# Bench‐Stable Boryl Thianthrenium Dication Enables Aziridinyl Boronate Synthesis via Metal‐Free Late‐Stage Aziridination with Diverse Nitrogen Nucleophiles

**DOI:** 10.1002/anie.202520969

**Published:** 2025-12-09

**Authors:** Veerabhadra R. Vulupala, Disni Gunasekera, Nagarjun R. Mallampudi, Ramy Yousef, Yusif I. Gyasi, Gopal R. Ramidi, Ifeoluwa Adedotun, Shiqing Xu

**Affiliations:** ^1^ Department of Chemistry Texas A&M University, College Station Texas 77843 USA; ^2^ Department of Pharmaceutical Sciences Irma Lerma Rangel College of Pharmacy, Texas A&M University, College Station Texas 77843 USA

**Keywords:** Aziridination, Boron, Electrochemistry, Late‐stage functionalization, Thianthrenation

## Abstract

Organoboron compounds are indispensable in modern organic synthesis and biomedical research. This study describes the synthesis of bench‐stable boryl thianthrenium dicationic compound via chemical or electrochemical thianthrenation of vinyl MIDA boronate. This unique boryl thianthrenium dication enables a transition‐metal‐free, chemo‐, and diastereoselective synthesis of aziridinyl boronates, utilizing a broad range of nitrogen nucleophiles. The method demonstrates generality, practicality, and functional group tolerance, as evidenced by its application to diverse substrates, including the late‐stage modification of drug molecules. Notably, the MIDA boryl group plays crucial roles in this approach including i) suppressing undesired deborylation, ii) promoting exclusive mono‐adduct formation via a formal [4 + 2] cycloaddition pathway, iii) directing regioselective vinyl boryl thianthrenium formation via selective deprotonation, and iv) enabling diastereoselective aziridination. The strategic significance of this approach is further highlighted through electrochemical one‐pot protocol, asymmetric synthesis using vinyl PIDA boronate, and diverse downstream transformations of aziridinyl boronates, offering new opportunities for synthetically challenging boron‐containing drug‐like scaffolds.

## Graphical abstract

A bench‐stable boryl thianthrenium dication is synthesized via thianthrenation of vinyl MIDA boronate. This novel dication enables metal‐free, chemo‐, and diastereoselective late‐stage aziridination with diverse nitrogen nucleophiles. The strategic significance is further showcased by one‐pot electrochemical and asymmetric protocols and versatile downstream transformations toward synthetically challenging boron‐containing drug‐like scaffolds.

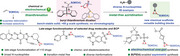

## Introduction

**Figure 1:**
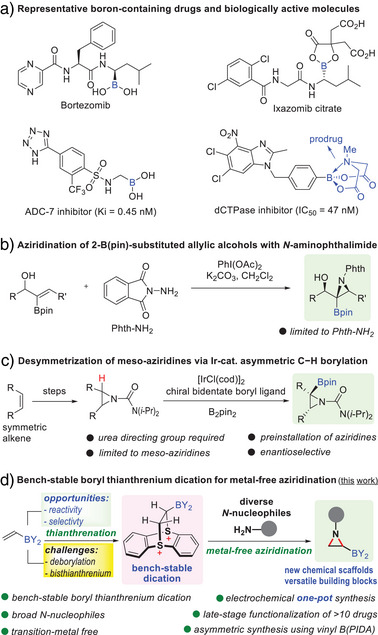
a) Representative boron‐containing drugs and biologically active molecules. Strategies for aziridinyl boronate synthesis: b) aziridination of 2‐B(pin)‐substituted allylic alcohols with *N*‐aminophthalimide; c) desymmetrization of meso‐aziridines via Ir‐catalzyed asymmetric C−H borylation; and d) bench‐stable boryl thianthrenium dication for metal‐free late‐stage aziridination.

Organoboron compounds are highly versatile building blocks for a wide range of synthetic transformations in modern organic synthesis.^[^ ^1^, ^2^, ^3^, ^4^, ^5^, ^6^, ^7^ ^]^ Beyond their synthetic utility, boron‐containing compounds are gaining recognition as a new frontier in biomedical research, exhibiting potential as imaging agents and therapeutic agents with anti‐cancer, antiviral, anti‐bacterial, and other disease‐specific activities.^[^ ^8^, ^9^, ^10^, ^11^, ^12^, ^13^, ^14^ ^]^ The unique chemical properties of boron, particularly its vacant p orbital, allow for reversible interactions with biological nucleophiles (e.g., hydroxyl and amine groups in enzyme residues, carbohydrates and nucleic acids), enabling the formation of anionic sp^3^‐hybridized complexes. This dynamic binding capability combined with the hydrogen‐bonding potential of boronic acids underscores boron's exceptional potential as a versatile anchoring element in drug discovery. Among boron‐based scaffolds, α‐aminoboronic acids and esters are particularly noteworthy as bioisosteres of amino acids and key pharmacophores in biologically active agents, probes, and FDA‐approved therapeutics.^[^ ^12^, ^15^, ^16^ ^]^ For example, bortezomib and ixazomib citrate have been approved for multiple myeloma treatment,^[^ ^12^, ^17^, ^18^ ^]^ and sulfonamide boronic acids serve as potent acinetobacter‐derived cephalosporinase‐7 (ADC‐7) inhibitors and β‐lactamase inhibitors.^[^ ^19^, ^20^, ^21^ ^]^ Furthermore, boronic esters, including *N*‐methyliminodiacetic acid (MIDA) boronates, have been employed as prodrug strategies to improve the solubility, stability, and permeability of boronic acids, further demonstrating their versatility and importance in advancing medicinal chemistry (Figure 1a).^[^ ^22^, ^23^, ^24^ ^]^ In addition, MIDA boronates are valuable in synthetic chemistry and automated iterative small‐molecule synthesis due to their enhanced stability, which arises from the steric protection and reduced Lewis acidity of the boron center conferred by the strongly coordinating *N*‐methyl of the MIDA group.^[^ ^25^, ^26^, ^27^ ^]^

Aziridines, on the other hand, represent valuable structural motifs prevalent in natural products and bioactive molecules and serve as versatile precursors of amine compounds via aziridine ring‐opening reactions.^[^ ^28^, ^29^, ^30^, ^31^, ^32^, ^33^, ^34^, ^35^, ^36^, ^37^ ^]^ Aziridinyl boronates, which integrate the distinct scaffold features of aziridines and α‐aminoboronates, offer significant promise for both synthetic chemistry and drug discovery. However, methods to synthesize aziridinyl boronates remain very limited. The Walsh Group first introduced a diastereoselective aziridination of 2‐boryl‐substituted allylic alcohols using *N*‐aminophthalimide and PhI(OAc)_2_, although the method is limited to *N*‐aminophthalimide (Figure 1b).^[^ ^38^ ^]^ The Yudin Group reported a single example of an aziridine MIDA boronate synthesized from an epoxide MIDA via epoxide ring opening, azidation with NaN_3_, and a subsequent Staudinger reaction with PPh_3_.^[^ ^39^ ^]^ Recently, Xu and co‐workers reported an Ir‐catalyzed asymmetric C(sp^3^)–H borylation of meso‐aziridines with urea as the directing group (Figure 1c).^[^ ^40^ ^]^ However, this method requires the preinstallation of meso‐aziridines and introduction of urea as the directing group. These approaches, while innovative, are constrained by substrate scope, functional group tolerance, and/or synthetic accessibility. As a consequence, there is a lack of synthetic methods for direct transformation of widely available and diverse nitrogen nucleophiles into aziridinyl boronates via the late‐stage aziridination approach, particularly for challenging aliphatic amines and amino acid derivatives.

Thianthrene chemistry has emerged as a powerful and broadly applicable synthetic strategy to functionalize arenes and alkenes.^[^ ^41^, ^42^, ^43^, ^44^, ^45^, ^46^, ^47^, ^48^, ^49^, ^50^ ^]^ Recent advances in alkene thianthrenation by Ritter, Wickens, and others have unveiled novel strategies for the generation of reactive electrophilic intermediates.^[^ ^44^, ^47^, ^49^, ^50^, ^51^, ^52^ ^]^ Notably, Wickens and co‐workers developed an elegant electrochemical method to convert alkenes into thianthrene‐based mono‐adduct and bis‐adduct dicationic electrophiles, enabling efficient aziridination with challenging aliphatic primary amines under mild conditions.^[^ ^30^ ^]^ Shu and collaborators described a related method to achieve aziridine synthesis via alkenyl thianthrenium salts as dielectrophiles.^[^ ^53^ ^]^ Inspired by these studies, we hypothesized that dicationic boryl‐thianthrenium intermediates derived from vinyl boronates could serve as versatile building blocks for the synthesis of aziridinyl boronates with diverse nitrogen nucleophiles. However, the inherent susceptibility of boryl intermediates to deborylation presents a significant synthetic challenge, as evidenced by previous studies on alkenyl and aryl boronic acid derivatives undergoing ipso‐substitution,^[^ ^54^, ^55^ ^]^ or Cu‐mediated thianthrenation and phenoxathiination.^[^ ^56^ ^]^ Importantly, boryl substituents offer new opportunities to tune reactivity and selectivity via their inherent electronic and steric effects.^[^ ^57^ ^]^

Herein, we address this challenge by introducing the synthesis of bench‐stable boryl thianthrenium dication intermediates via chemical or electrochemical thianthrenation of vinyl MIDA boronate. The boryl thianthrenium dication unlocks a general, metal‐free approach for the efficient and diastereoselective synthesis of aziridinyl boronates with diverse nitrogen nucleophiles (Figure 1d). Key features of this strategy include: i) excellent selectivity for the mono‐thianthrenium formation; ii) broad substrate compatibility of nitrogen nucleophiles, including sulfonamides, sulfamates, alkyl amines, amino esters, and hydrazides; iii) late‐stage functionalization of drug molecules with excellent chemoselectivity and functional group tolerance; iv) high diasteroselectivity of boryl thianthrenium dication‐enabled metal‐free aziridination; v) distinct regioselectivity of deprotonation of boryl thianthrenium dication; and v) asymmetric synthesis using chiral vinyl boronate. This approach will significantly broaden the range of aziridinyl boronates that are otherwise very challenging to access.

## Results and Discussion

Our investigation began with thianthrenation of vinylboronic acid pinacol ester **1a** using Ritter's conditions:^[^ ^51^ ^]^ **1a** (0.3 mmol, 1.0 equiv), thianthrene oxide (TTO) **2** (1.03 equiv), trifluoroacetic anhydride (TFAA) (3 equiv), triflic acid (HOTf) (1.2 equiv) in MeCN 3 mL at 0 °C for 1 h. The reaction did not yield the desired boryl thianthrenium dication **3a** but instead afforded the deborylated vinyl thianthrenium product **4** in 90% NMR yield and 80% isolated yield (Table 1 **, entry 1**). The same reaction conditions were applied to the more sterically hindered vinyl boronic ester, 1,1,2,2‐tetraethylethylene glycol ester (BEpin) **1b**,^[^ ^58^ ^]^ but this substrate similarly led to the formation of the deborylated byproduct **4** in 80% yield (**entry 2**). The deborylation process likely proceeds through a pathway similar to 1,2‐dehaloboration, wherein nucleophilic attack at the boron center facilitates alkene formation.^[^ ^59^, ^60^, ^61^ ^]^ To overcome this deborylation issue, we turned our attention to sp^3^‐hybridized MIDA boronates, which are known for their enhanced stability due to a strong B–N dative bond of *N*‐methyl of MIDA group to the boron center. This coordination effectively attenuates susceptibility toward nucleophilic attack and underlies the broad utility of MIDA boronates in organic synthesis and medicinal chemistry.^[^ ^25^, ^27^ ^]^ Then, we investigated the thianthrenation of vinyl MIDA boronate **1c** under similar reaction conditions. Remarkably, this substrate **1c** completely suppressed the deborylation, furnishing the desired boryl thianthrenium dicationic adduct **3c** in an excellent 90% isolated yield (**entry 3**). Notably, this reaction is highly scalable; **3c** was synthesized on a preparative scale, yielding over 60 g of product in a single step through simple precipitation, without requiring column chromatography (**entry 4**). The structure of **3c** was unambiguously confirmed via X‐ray crystallography (CCDC 2414383). The boryl thianthrenium dication **3c** was obtained as a white solid, which is bench‐stable and can be stored for several months without decomposition. This high stability, combined with its straightforward preparation and handling, highlights the practicality of **3c** for broader synthetic applications.

**Table 1:** Thianthrenation of vinyl boronates to boryl thianthrenium dication **3**.

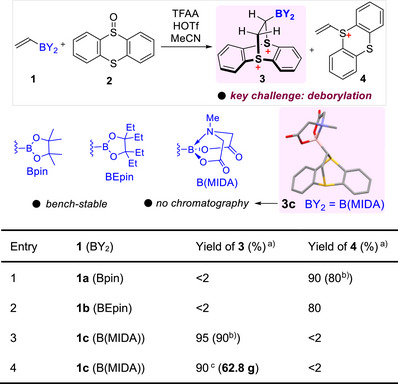

The reaction was conducted using **1** (0.3 mmol, 1.0 equiv), TTO **2** (1.03 equiv), TFAA (3 equiv), and HOTf (1.2 equiv) in MeCN (3 mL) at 0 °C for 1 h.

^a)^NMR yield;

^b)^Isolated yield;

^c)^Multiple gram‐scale synthesis using HOTf (2 equiv).

To evaluate the synthetic utility of boryl thianthrenium dication **3c**, we initiated our investigation by employing celecoxib and **3c** as the model substrates (Table 2). To our delight, desired *N*‐tosyl aziridinyl MIDA boronate **5** was formed in excellent 94% yield using potassium carbonate (5.0 equiv) as the base in dichloromethane (DCM, 0.1 M) at room temperature (Table 2 **, entry 1**). Encouraged by this result, we explored alternative bases and solvents. Substituting potassium carbonate with cesium carbonate or potassium phosphate yielded **5** with comparable yields of 93% and 90%, respectively (**entries 2 and 3**). Employing a weaker base such as sodium bicarbonate resulted in a significantly reduced yield of 23% (**entry 4**). On the other hand, using a stronger base like sodium *tert*‐butoxide yielded **5** in only 18% (**entry 5**). We next evaluated the effect of solvents on the reaction. The transformation proceeded smoothly in acetonitrile, THF, and DMF, delivering the desired product **5** in yields ranging from 75%–90% (**entries 6–8**). However, when DMSO was used as the solvent, the yield dropped significantly to 33%. To demonstrate the scalability of this transformation, we conducted a gram‐scale synthesis of **5** under the optimized conditions (potassium carbonate as base in DCM), affording 3.62 g of **5** in 92% isolated yield (**entry 10**).

**Table 2:** Optimization of reaction conditions.

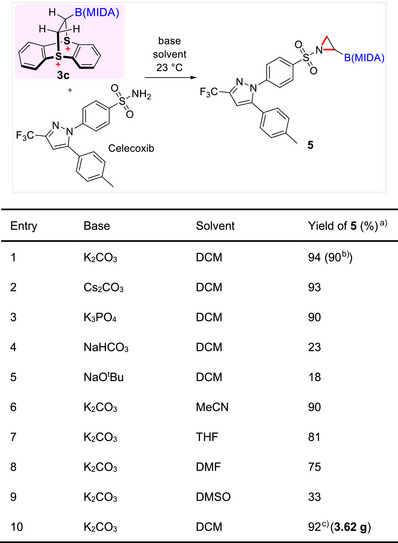

The reaction was conducted using **3c** (0.2 mmol, 1.0 equiv), celecoxib (1.2 equiv), and base (5 equiv) in solvent 2 mL at 23 °C for 14 h.

^a)^NMR yield;

^b)^Isolated yield;

^c)^Gram‐scale synthesis.

**Figure 2:**
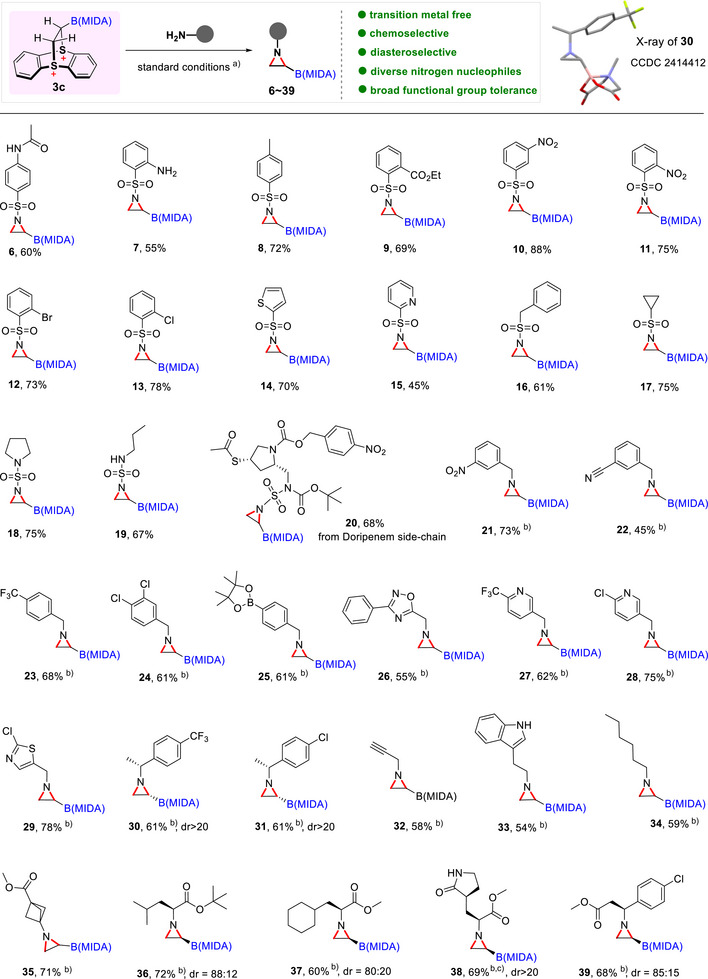
Scope of the metal‐free aziridination of boryl thianthrenium dication **3c**. ^a)^ The reaction, unless otherwise noted, was conducted using **3c** (0.3 mmol, 1.0 equiv), RNH_2_ (1.2 equiv), and K_2_CO_3_ (5 equiv) in DCM 3 mL at 23 °C for 14 h. ^b)^ The reaction was conducted using **3c** (0.3 mmol, 1.0 equiv), RNH_2_ (1.2 equiv), and Cs_2_CO_3_ (3 equiv) in MeCN 3 mL at 23 °C for 14 h. All yields are for isolated aziridinyl boronate products. ^c)^ The dr ratio refers specifically to the newly formed C–B stereocenter.

**Figure 3:**
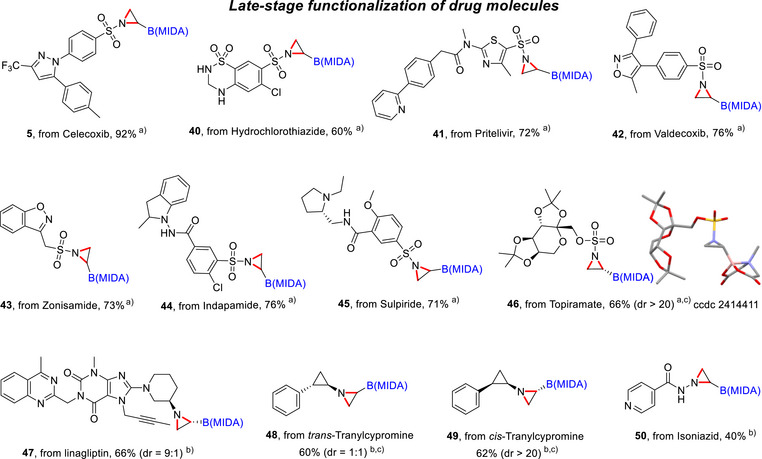
Late‐stage functionalization of drug molecules. ^a)^ The reaction was conducted using **3c** (0.3 mmol, 1.0 equiv), RNH_2_ (1.2 equiv), and K_2_CO_3_ (5 equiv) in DCM 3 mL at 23 °C for 14 h. ^b)^ The reaction was conducted using **3c** (0.3 mmol, 1.0 equiv), RNH_2_ (1.2 equiv), and Cs_2_CO_3_ (3 equip) in MeCN 3 mL at 23 °C for 14 h. ^c)^ The dr ratio refers specifically to the newly formed C–B stereocenter.

With optimized conditions in hand, we evaluated the versatility and robustness of this metal‐free aziridination protocol by synthesizing over 40 aziridinyl boronates using a variety of nitrogen nucleophiles (Figures 2 and 3). We began by examining the reactivity of a broad range of sulfonamides as both sulfonamides^[^ ^62^, ^63^ ^]^ and sulfonamide boronic acids^[^ ^19^, ^20^, ^21^ ^]^ constitute important classes of drugs and bioactive molecules. Aryl sulfonamides bearing electron‐donating groups, such as acetamide **6**, amino **7**, and methyl **8**, as well as electron‐withdrawing groups, including ester **9**, nitro **10–11**, bromide **12**, and chloride **13**, were all compatible with the transformation, affording the corresponding aziridinyl boronates in good to excellent yields. Heteroaryl sulfonamides, such as **14** and **15**, as well as aliphatic sulfonamides **16** and **17**, were similarly well‐suited to this transformation, delivering aziridinyl boronates. Furthermore, sulfamides, including **18**, **19**, and doripenem sidechain **20**, underwent aziridination to afford aziridinyl boronates in good yields, demonstrating the broad applicability of the reaction.

We next investigated the expansion of nitrogen nucleophiles to amines and amino acids. Gratifyingly, *N*‐alkyl aziridinyl boronates were readily synthesized from a wide range of amine nucleophiles by using Cs_2_CO_3_ as a base in MeCN at room temperature. Benzyl amines, heterobenzylic amines, propargyl amine, and linear aliphatic amines underwent efficient aziridination, furnishing desired aziridines **21**–**35**. Enantioenriched aziridines were readily obtained by the aziridination reaction of commercially available chiral amines, affording **30** and **31** in 61% yield with excellent > 20:1 diastereomeric ratio (dr) values. The absolute configuration of **30** was unambiguously confirmed via X‐ray crystallographic analysis (CCDC 2414412). Notably, tryptamine, containing a potentially competing unprotected indole group, underwent successful aziridination to yield **33**. Additionally, sterically congested bicyclo [1.1.1]pentane (BCP) amine, a bioisostere of *para*‐substituted benzene with growing relevance in medicinal chemistry,^[^ ^64^, ^65^ ^]^ proceeded smoothly to produce the corresponding BCP aziridine **35** in 71% yield. To the best of our knowledge, this is the first report of BCP aziridines, offering novel scaffolds and building blocks for BCP‐based drug discovery. Furthermore, amino acid derivatives, including α‐amino esters and β‐amino esters, were also well tolerated under these conditions, delivering aziridinyl boronates **36**–**39**. Notably, substrates with additional nucleophilic groups such as aniline (**7**), unprotected indole (**33**), *NH*‐amides (**6, 38**), *NH*‐sulfamides (**19**), and terminal alkynes **(32**), were efficiently converted into the corresponding aziridinyl boronates, underscoring the mildness of the protocol and its broad functional‐group compatibility. Despite this versatility, certain substrates proved challenging under the current conditions (Figure S3). Phosphoramidate and sulfinamide failed to undergo the desired aziridination, possibly due to their reduced nucleophilicity arising from relatively high pKa values. Aniline produced a diamine product instead of the desired aziridine. In addition, 1,1‐disubstituted alkenyl B(MIDA) substrates did not undergo thianthrenation presumably due to steric hindrance, while 1,2‐disubstituted analogs proceeded thianthrenation but exhibited divergent reactivity with nitrogen nucleophiles, favoring allylic C─N bond formation over aziridination (Figure S3).

### Late‐Stage Functionalization of Drug Molecules

The synthetic utility of this method was further showcased through the late‐stage functionalization of various drug molecules. The mild conditions, coupled with excellent functional group tolerance, allowed the successful modification of a wide range of sulfonamide and sulfamate drugs, including celecoxib, hydrochlorothiazide, pritelivir, valdecoxib, indapamide, sulpiride, zonisamide, and topiramate, affording corresponding boryl aziridine derivatives **5**, **40**–**46** in yields ranging from 60% to 92% (Figure 3). Notably, the aziridination of topiramate, a widely used anti‐convulsant and nerve pain drug, yielded **46**, and its absolute configuration was confirmed via X‐ray crystallography (CCDC 2414411). In addition to sulfonamide‐based drugs, this method also proved effective for aliphatic primary amine drugs. For example, linagliptin and tranylcypromine were readily converted to their boryl aziridine analogs **47**–**49**. Interestingly, isoniazid, a first‐line tuberculosis treatment containing a hydrazide motif, underwent successful aziridination to yield **50**. It should be noted that heterocyclic compounds are ubiquitous in biologically active molecules, but their susceptibility to oxidation and their potential to poison transition‐metal catalysts often complicate their functionalization in traditional aziridination reactions. In contrast, this boryl thianthrenium dication‐enabled methodology offers several distinct advantages: i) transition‐metal‐free, ii) oxidant‐free in the aziridination step, iii) broad functional group tolerance, and iv) excellent chemo‐ and diastereoselectivity. This robustness enabled the selective conversion of commercially available diverse nitrogen nucleophiles bearing heteroaromatic or saturated heterocyclic groups into aziridinyl boronates in a single step, providing an attractive strategy for late‐stage functionalization and the development of potential boron‐containing therapeutic candidates.

**Figure 4:**
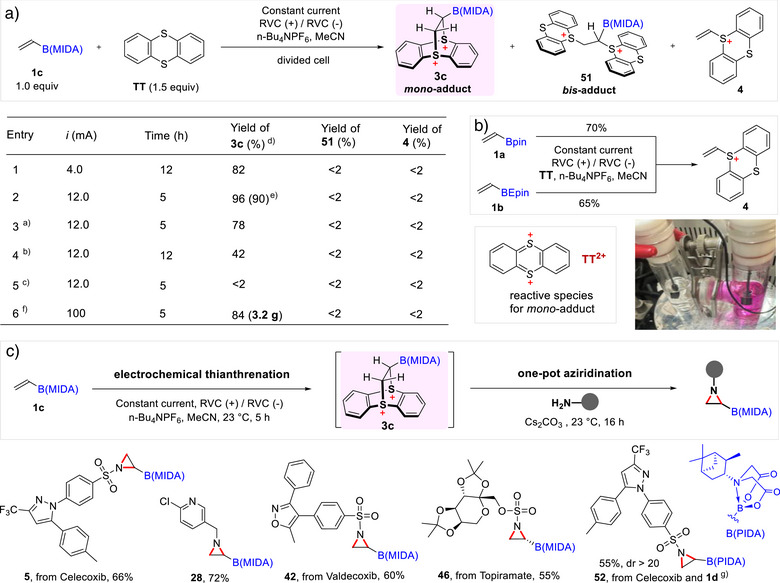
a) Optimization of electrochemical thianthrenation of vinyl MIDA boronate. Reaction scale: Anode (RVC): 0.3 mmol of **1c** (1 equiv), 0.45 mmol of TT (1.5 equiv) in 4 mL MeCN (0.2 M n‐Bu_4_NPF_6_); Cathode (RVC): TFA (0.8 mmol) in 4 mL MeCN (0.2 M n‐Bu_4_NPF_6_); ^a)^ Ni form as the cathode; ^b)^ ElectraSyn as the electrochemical cell; ^c)^ RVC electrode connected by a wire on the anode side; ^d)^ NMR yield; ^e)^ isolated yield; ^f)^ Reaction scale: Anode (RVC): 5.5 mmol of **1c** (1 equiv), 8.25 mmol of TT (1.5 equiv) in 20 mL MeCN (2.2 equiv n‐Bu_4_NPF_6_); Cathode (RVC): TFA (2.5 equiv) in 20 mL MeCN (2.2 equiv n‐Bu_4_NPF_6_); b) Standard electrochemical thianthrena‐tion of **1a** and **1b** led to deborylated product **4**; c) One‐pot electrochemical aziridination. Electrochemical conditions are the same as A using 0.3 mmol of **1c**. Then RNH_2_ (0.2 mmol) and Cs_2_CO_3_ (1.0 mmol) were added to the anode cell, stirred for 16 h. ^g)^ 0.3 mmol of vinyl PIDA boronate **1d** instead of **1c**.

Building on the successful development of chemical thianthrenation of vinyl B(MIDA) under acidic conditions and the metal‐free aziridination, we next turned our attention to a more sustainable and acid‐free electrochemical thianthrenation approach. Electrochemistry has emerged as a cornerstone of sustainable organic synthesis, offering an environmentally friendly alternative to traditional chemical methods for oxidizing or reducing organic compounds.^[^ ^66^, ^67^, ^68^, ^69^, ^70^, ^71^, ^72^, ^73^, ^74^ ^]^ By leveraging electrons from a power supply to drive redox reactions, electrochemical methods reduce the need for stoichiometric chemical reagents, lower production costs, and promote sustainability by minimizing waste and energy consumption. Wickens group demonstrated the utility of anodic oxidation of inexpensive thianthrene (TT) to form dicationic thianthrene adducts with alkenes, enabling diverse in situ transformations.^[^ ^30^, ^75^, ^76^, ^77^ ^]^ These methods generate a mixture of mono‐ and bis‐adducts through two reactive species, thianthrene radical cation (TT^+•^) and thianthrene dication (TT^2+^), and have been primarily limited to alkyl‐substituted alkenes. Notably, Wickens and co‐workers recently reported *Z*‐alkenyl thianthrenium formation via *Z*‐selective elimination of electrogenerated bis‐TT adducts.^[^ ^78^ ^]^ To expand the scope of electrochemical thianthrenation, we investigated the reactivity of vinyl B(MIDA) **1c** using a divided cell setup with RVC electrodes RVC(+)/RVC(‐) (Figure 4a). In our experiments, we found that applying a current of 4 mA afforded desired product **3c** with [PF_6_]^−^ as anions in a good yield of 82% in 12 h (**entry 1**). Remarkably, only the mono‐adduct **3c** was formed, with no detectable bis‐adduct **51** or deborylation by‐product **4**. This selectivity contrasts with Wickens's findings, where terminal alkenes afforded mixtures of mono‐ and bis‐adducts.^[^ ^30^ ^]^ Increasing the current to 12 mA further improved the yield of **3c** to 96% and reduced the reaction time to 5 h, still with no bis‐adduct formation (**entry 2**). Substituting the cathode with a nickel form electrode resulted in a reduced yield of 78% (**entry 3**), while changing the connection from pencil/RVC to stainless steel wire/RVC led to no product formation (**entry 5**), highlighting the importance of a proper connection to the RVC for successful reaction progression. When the reaction was performed in an undivided cell using an ElectraSyn apparatus (**entry 4**), the yield dropped significantly to 42%, likely due to potential competing reduction process of **3c** at the cathode. Similar to chemical thianthrenation, electro‐chemical thianthrenation of vinyl Bpin **1a** and vinyl BEpin **1b** led to deborylated vinyl thianthrenium product **4** in 70% and 65% NMR yield, respectively (Figure 4b). Furthermore, we explored the scalability of the electrochemical process by employing vinyl B(MIDA) **1c** (5.5 mmol), resulting in the successful synthesis of **3c** (3.2 g) in an 84% yield (**entry 6**). This result demonstrates the practicality and efficiency of this electrochemical method.

The acid‐free nature of the electrochemical synthesis of **3c** is particularly advantageous for developing a one‐pot aziridination protocol under basic conditions, eliminating the need for intermediate workup or purification. To showcase the feasibility of this one‐pot process, we evaluated its applicability to the late‐stage aziridination of drug molecules containing amine groups. In this protocol, the dicationic boryl thianthrenium salt **3c** was first generated in the divided cell under optimized conditions. Subsequently, amine‐containing substrates and Cs_2_CO_3_ were added directly to the anode compartment to afford the desired aziridinyl boronates **5**, **28**, **42**, and **46**. A pinene‐derived iminodiacetic acid (PIDA) ligand has previously been shown to function as an effective chiral auxiliary, enabling highly diastereoselective epoxidation of alkenylboronates to furnish chiral oxiranyl PIDA boronates.^[^ ^79^ ^]^ Motivated by these precedents, we investigated the reactivity of vinyl B(PIDA) **1d** in thiathrenation and aziridination transformation. Gratifyingly, this one‐pot electrochemical process proceeded with high levels of stereocontrol, providing access to chiral aziridinyl boronate **52** in 55% yield with excellent diastereoselectivity (Figure 4c). The acid‐free conditions and high selectivity make this strategy particularly attractive for the sustainable synthesis of boron‐containing compounds with pharmaceutical relevance.

**Figure 5:**
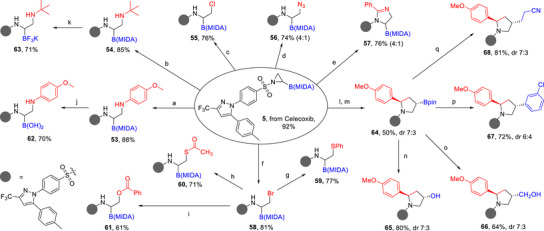
Diverse transformations of aziridinyl boronate **5**. Reaction conditions: a) **5** (1.0 equiv), p‐anisidine (3.0 equiv), DMF, 60 °C, 14 h; b) **5** (1.0 equiv), *tert*‐butylamine, MeCN, 1 h; c) **5** (1.0 equiv), benzyltriethylammonium chloride (1.5 equiv), BF_3_·Et_2_O (1.2 equiv), DCM, 0 °C, 5 h; d) **5** (1.0 equiv), NaN_3_ (3.0 equiv), DMF, 75 °C, 10 h; e) **5** (1.0 equiv), BF_3_·Et_2_O (1.2 equiv), PhCN, 1 h; f) **5** (1.0 equiv), ben‐zyltriethylammonium bromide (1.5 equiv), BF_3_·Et_2_O (1.2 equiv), DCM, 0 °C, 1 h; g) **57** (1.0 equiv), PhSNa (1.5 equiv), NaI (1.5 equiv), acetone, 55 °C, 12 h; h) **58** (1.0 equiv), potassium ethanethioate (1.5 equiv), NaI (1.5 equiv), DMF, 60 °C, 12 h; i) **58** (1.0 equiv), sodium benzoate (1.5 equiv), NaI (1.5 equiv), DMF, 60 °C, 12 h; j) **52** (1.0 equiv), 3 N HCl (2.5 equiv), MeOH, 4 h; k) **53** (1.0 equiv), KHF_2_ (5 equiv), MeCN/MeOH = 4:1, 16 h; l) **5** (1.0 equiv), 4‐vinylanisole (1.5 equiv); 4‐CzlPN (4 mol%), (4,4′‐dtbbpy)NiCl_2_ (8 mol%), NaI (2.0 equiv), Et_3_N (3.0 equiv), blue LEDs (456 nm), THF, 70 °C, 48 h; m) pinacol (1.2 equiv), DCM/MeOH = 1:1, 45 °C, 12 h; n) **64** (1.0 equiv), NaBO_3_ (5.0 equiv), THF/H_2_O = 4:1, 3 h; o) **64** (1.0 equiv), CH_2_Br_2_ (4 equiv), nBuLi (3.9 equiv), THF, ‐78∼23 °C, 21 h; H_2_O_2_, NaOH; p) **64** (1.3 equiv), 1‐bromo‐3‐chlorobenzene (1.0 equiv), morpholine (1.5 equiv), [Ir(dF(CF_3_)ppy)_2_(dtbbpy)]PF_6_ (1 mol%), NiCl_2_·glyme (5 mol%), dtbbpy (5 mol%), DMF, blue LEDs (456 nm), 2 h; and q) **64** (1.3 equiv), acrylonitrile (4.0 equiv), DMAP (1.5 equiv), [Ir(dF(CF_3_)ppy)_2_(dtbbpy)]PF_6_ (2 mol%), acetone/MeOH 1:1, blue LEDs (456 nm).

We further explored the downstream derivatization of aziridinyl boronates to demonstrate their versatility in accessing a broad range of α‐ and β‐aminoboronic esters, important classes of bioactive molecules with significant medicinal and synthetic applications. Aziridinyl MIDA boronate **5**, derived from a nonsteroidal anti‐inflammatory drug celecoxib in 92% yield, was chosen as a representative substrate (Figure 5). Initial investigations focused on regioselective aziridine ring‐opening reactions using various nucleophiles to generate α‐aminoboronic esters. For instance, the treatment of aziridine **5** with p‐anisidine and t‐butylamine resulted in the formation of regioselective ring‐opening products **53** and **54** in excellent yields of 88% and 85%, respectively. We further evaluated ring‐openingreactions using benzyl triethylammonium halides (Cl and Br) in combination with BF_3_·Et_2_O at 0 °C, which successfully afforded halogenated derivatives **55** and **58** in 76% and 81% yields, respectively. Additionally, the reaction of aziridine **5** with sodium azide in DMF at 75 °C produced the azido sulfonamide **56** with a regioselectivity ratio of 4:1. Beyond traditional ring‐opening reactions, aziridine **5** displayed reactivity in cycloaddition chemistry.^[^ ^80^, ^81^ ^]^ Treatment of **5** with BF_3_·Et_2_O and benzonitrile enabled a [3 + 2] cycloaddition to form the dihydroimidazole derivative **57** in 76% yield with 4:1 regioselectivity. This transformation highlights the potential of aziridinyl boronates to access nitrogen‐containing heterocycles in a straightforward manner. We further explored nucleophilic substitution of the bromo derivative **58**, which was prepared via regioselective azirdine ring‐opening reaction. The bromo intermediate **58** was treated with various nucleophiles, including sodium benzoate, sodium benzenethiolate, and potassium ethanethioate in the presence of NaI, yielding derivatives **59**–**61** in good yields. The B(MIDA) group can be further converted into the corresponding B(OH)_2_ and BF_3_K derivatives, affording compounds **62** and **63** in good yields.

After synthesizing various α‐aminoboronic esters, we next directed our efforts toward the preparation of cyclic β‐aminoboronic ester **64** via a photoredox [3 + 2] cycloaddition reaction of aziridine **5** and 4‐vinylanisole.^[^ ^82^ ^]^ This reaction afforded a disubstituted sulfonyl pyrrolidine MIDA boronate, which was subsequently converted to the corresponding pinacol ester **64** in 50% overall yield. To further showcase the synthetic utility of organoboron, **64** was oxidized to afford alcohol **65** in 80% yield. Boron homologation of **64** via a 1,2‐boronate rearrangement followed by in situ oxidation provided **66** in 64% yield.^[^ ^83^ ^]^ In addition, C(sp^2^)–C(sp^3^) coupling reaction enabled by dual photoredox/nickel catalysis afforded **67** in 72% yield.^[^ ^84^ ^]^ Furthermore, Ir photocatalyst was used to oxidize C–Bpin bond of **64** in the presence of dimethylaminopyridine (DMAP) to generate a stabilized alkyl radical intermediate, which underwent radical addition to acrylonitrile to furnish **68** in 81% yield.^[^ ^85^ ^]^ Collectively, these results highlight aziridinyl boronates as versatile and robust intermediates for constructing a wide array of functionalized molecules containing the α‐ and β‐aminoboronic ester moiety. The ability to perform late‐stage modifications with excellent functional group tolerance and broad reactivity of organoboron compounds further underscores their potential as valuable building blocks in medicinal chemistry and complex molecule synthesis.

**Figure 6:**
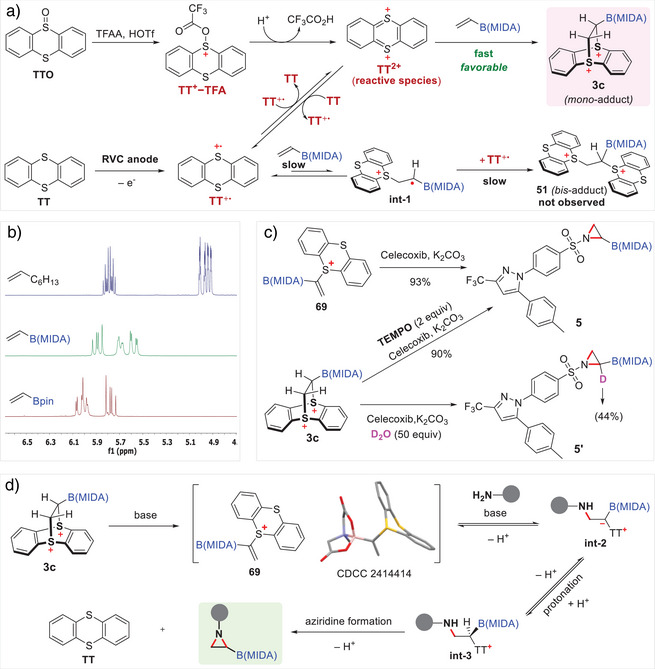
a) Proposed mechanism of the chemical or electrochemical thianthrenation of vinyl MIDA boronate; b) Comparative ^1^H NMR analysis of 1‐octene, vinyl Bpin, and vinyl MIDA boronate; c) Control experiments for aziridination; d) Proposed mechanism of transition‐metal‐free aziridination.

For the mechanism for thianthrenation of vinyl MIDA boronate **1c** using TTO, TFAA, and HOTf, the thianthrene dication TT^2+^ is proposed as the reactive species based on the experimental results of exclusive formation of mono‐adduct **3c** and Ritter's studies.^[^ ^51^, ^86^ ^]^ Activation of TTO with TFAA and Brønsted acid HOTf generates trifluoroacetylated derivative TT^+^–TFA, which undergoes further electron transfer under acidic conditions to form TT^2+^. This highly reactive species TT^2+^ facilitates a fast inverse‐electron demand [4 + 2] cycloaddition with vinyl MIDA boronate **1c**, leading exclusively to mono‐thianthrenium diatonic adduct **3c**. This is consistent with Ritter's studies on the stereoselective thianthrenation of olefins.^[^ ^51^ ^]^ In contrast, the mechanism of electrochemical thianthrenation, as elucidated by the Wickens Group, involves two distinct reactive species thianthrene radical cation TT^+•^ and dication TT^2+^.^[^ ^30^, ^75^, ^76^, ^77^ ^]^ Anodic oxidation of TT generates TT^+•^, which reacts with olefins to initially form bis‐adducts through radical addition followed by sequential radical trapping. At lower TT concentrations, TT^+•^ undergoes disproportionation to yield TT^2^⁺, leading to mono‐adduct via the rapid [4 + 2] cycloaddition. This dual‐species mechanism results in a mixture of mono‐ and bis‐adducts, each formed with high selectivity at different stages of the reaction due to the involvement of two distinct reactive species TT^+•^ and TT^2+^.^[^ ^30^ ^]^ Notably, the electro‐chemical thianthrenation of vinyl MIDA boronate **1c** differs from Wickens's studies. Exclusively the mono‐adduct **3c** is produced, with no bis‐adduct **51** observed by ^1^H NMR and HRMS analysis. This unprecedented selectivity likely arises from the unique electronic and steric effects of the MIDA boryl group compared to carbon‐based substituents. Unlike the tricoordinate pinacol boryl Bpin group, which is known for its electron‐withdrawing characteristics due to a partially vacant boron orbital, the electronic nature of the MIDA boryl group whether electron‐withdrawing or electron‐donating is less clearly defined.^[^ ^57^, ^87^, ^88^ ^]^ The stable sp^3^‐hybridized tetracoordinate boron complex in B(MIDA), featuring intramolecular B─N coordination, moderates its electron‐withdrawing nature. To assess these electronic effects, we conducted comparative ^1^H NMR analysis of 1‐octene, vinyl Bpin, and vinyl MIDA boronate (Figure 6b). The results indicate that both Bpin and B(MIDA) are electron‐withdrawing, with Bpin exerting a stronger effect, as evidenced by the downfield chemical shifts of the C═C protons. Vinyl MIDA boronate exhibited no distinct oxidation peak in cyclic voltammetry (Figure S2). In radical cation addition, electron‐poor alkenes like vinyl MIDA boronate **1c** are less reactive toward electrophilic TT^+•^, slowing the radical addition pathway. Steric effects further contribute to this selectivity. The sp^3^‐hybridized boron center in B(MIDA) introduces steric hindrance and would further suppress radical trapping of the intermediate radical cation **int‐1** by the highly bulky TT^+•^. Collectively, these electronic and steric factors disfavor the TT^+•^ pathway and promote exclusive formation of the mono‐thianthrenium adduct **3c** via TT^2+^ under electrochemical conditions.

To gain insight into the reaction mechanism of boryl thianthrenium dication **3c**‐enabled metal‐free aziridination, we conducted several control experiments (Figure 6c). When **3c** was reacted with celecoxib in the presence of the radical scavenger TEMPO under standard conditions, the desired aziridine **5** was obtained in 90% yield, with no observable impact on the reaction efficiency. This finding strongly suggests that the aziridination does not proceed through a radical pathway. During the aziridination process, we observed the rapid consumption of **3c**, leading to the formation of an alkenyl thianthrenium salt intermediate **69**. This intermediate was isolated and structurally characterized using NMR and X‐ray crystallography (CCDC 2414414). In contrast to alkenyl thianthrenium salt formation with carbon‐substituted olefins reported by Ritter,^[^ ^51^ ^]^ the distinct regioselectivity of **69** is likely due to the electron‐withdrawing effect of boron outweighing steric influences which would preferentially facilitate the deprotonation of the α‐proton of **3c** adjacent to B(MIDA). To confirm the role of compound **69** in the aziridination pathway, we subjected the isolated intermediate to standard aziridination conditions. The reaction yielded the desired aziridinyl boronate **5** in 93% yield, providing compelling evidence that **69** serves as a key intermediate in the reaction pathway. Additionally, a deuterium‐labeling experiment was performed using D_2_O as the deuterium source under standard conditions. The resulting formation of the α‐deuterated aziridine **5′** suggests the involvement of a protonation step in the mechanism. Based on these experimental findings and relevant literature,^[^ ^30^, ^51^, ^53^ ^]^ we propose the following plausible mechanism depicted in Figure 6d: 1) dicationic adduct **3c** rapidly transforms into alkenyl thianthrenium salt **69** via B(MIDA)‐directed selective deprotonation; 2) a nitrogen nucleophile undergoes nucleophilic conjugate addition to alkenylthianthrenium salt **68**, generating a stabilized thianthrenium ylide **int‐2**; 3) protonation of ylide **int‐2** produces cationic **int‐3**, where the proton originates from the amine nucleophile itself, similar to a Michael‐type addition;^[^ ^30^, ^53^ ^]^ and 4) **int‐3** subsequently undergoes intramolecular nucleophilic substitution to form the desired aziridine product, releasing the thianthrene.

## Conclusion

In conclusion, we have developed two readily scalable methods for preparing bench‐stable boryl thianthrenium dication **3c** by either chemical or electrochemical thianthrenation of vinyl MIDA boronate. This unique boryl thianthrenium dication serves a versatile building block to enable a transition‐metal‐free, chemo‐ and diastereoselective synthesis of aziridinyl boronates, utilizing a broad range of nitrogen nucleophiles. Notably, the MIDA boryl group plays crucial roles in this approach including i) suppressing undesired deborylation, ii) promoting exclusive mono‐adduct formation via a formal [4 + 2] cycloaddition pathway with thianthrene dication TT^2+^, iii) directing regioselective vinyl boryl thianthrenium formation via selective deprotonation of **3c**; and iv) enabling diastereoselective aziridination. The approach demonstrates generality, practicality, and functional group tolerance, as evidenced by its application to diverse substrates, including the late‐stage modification of over ten drug molecules. The strategic significance of this approach is further highlighted through an electrochemical one‐pot protocol, asymmetric synthesis using vinyl B(PIDA), and diverse downstream transformations of aziridinyl boronates and organoboron compounds. This approach significantly enhances the synthetic accessibility of aziridinyl boronates that were previously inaccessible, offering new opportunities for synthetically challenging boron‐containing drug‐like scaffolds.

## Supporting Information

The Supporting Information is available free of charge online. Experimental procedures and characterization of all compounds (PDF).

## Accession Codes

CCDC 2414383 (**3c**), CCDC 2414412 (**30**), CCDC 2414411 (**46**), and CCDC 2414414 (**69**) contain the supplementary crystallo‐graphic data for this paper. These data can be obtained free of charge via www.ccdc.cam.ac.uk/data_request/cif, or by emailing data_request@ccdc.cam.ac.uk.

## Conflict of Interests

The authors declare no conflict of interest.

## Supporting information



Supporting Information

## Data Availability

The data that support the findings of this study are available in the Supporting Information of this article.
