# Supplementary material for: Bench‐Stable Boryl Thianthrenium Dication Enables Aziridinyl Boronate Synthesis via Metal‐Free Late‐Stage Aziridination with Diverse Nitrogen Nucleophiles
Source: Angew Chem Int Ed Engl. 2025 Dec 9;65(4):e20969. doi: 10.1002/anie.202520969 (PMC12828481; doi:10.1002/anie.202520969)
Supplement: Supplementary file 1 — Supporting Information [file ANIE-65-e20969-s001.pdf]

Supporting Information

Bench-Stable Boryl Thianthrenium Dication Enables Aziridinyl Boronate Synthesis via Metal-Free Late-Stage Aziridination with Diverse Nitrogen Nucleophiles

Veerabhadra R. Vulupala,[a] Disni Gunasekera,[a] Nagarjun R. Mallampudi,[a] Ramy Yousef,[a] Yusif I. Gyasi,[a] Gopal R. Ramidi,[a] Ifeoluwa Adedotun,[a] and Shiqing Xu*,[a,b]

[a] Department of Chemistry, Texas A&M University, College Station, Texas 77843, United States

[b] Department of Pharmaceutical Sciences, Irma Lerma Rangel College of Pharmacy, Texas A&M University, College Station, Texas77843, United States

Contents

1. General Information 3

2. General procedures for chemical synthesis 4

2A. General procedure for boryl thianthrenium dication formation 4

2B. General procedure for the metal-free aziridination of boryl dication 5

3. General experimental procedures for electrolysis 5

3A. General procedure and experimental setup for boryl thianthrenium dication formation (divided cell) 5

3B. General procedure and experimental setup for boryl thianthrenium dication formation (Undivided cell) 6

3C. General procedure and experimental setup for gram-scale boryl thianthrenium dication formation (divided cell) 6

3D. General procedure for electrochemical one-pot aziridination reaction 7

3E. General procedures for cyclic voltammetry 8

4. Unsuccessful substrates 9

5. Experimental and characterization data 10

6. X-Ray crystallography data (compounds 3c, 30, 46, 69) 45

7. References 89

8. Copies of ^{1}H, ^{13}C, ^{11}B and ^{19}F NMR Spectra 90

1. General Information

Analytical thin layer chromatography (TLC) was carried out using silica gel 60 F254 pre-coated plates. Visualization was accomplished with UV lamp or I2 stain. Silica gel 230-400 mesh size was used for flash column chromatography using the combination of ethyl acetate and petroleum ether as eluent. Unless noted, all reactions were carried out in oven-dried glassware under an atmosphere of nitrogen/argon using anhydrous solvents. All commercial reagents were used as received. ^{1}H NMR, ^{13}C NMR, ^{11}B NMR and ^{19}F NMR spectra were recorded on Varian-Inova-400, Bruker-ARX-400 or Bruker Avance-III-800 spectrometer at ambient temperature. Multiplicities are indicated as s (singlet), d (doublet), t (triplet), m (multiplet) and br (broad). Mass spectra (MS) were obtained using ESI mass spectrometers.

All experiments with divided cell were carried out using a DC power supply (HM305P), and RVC electrodes (35 mm × 5 mm × 3 mm) was purchased from IKA and (6.0″ × 6.0″ × 0.25″) was purchased from ERG Aerospace Corporation. Undivided cell experiments were carried out using IKA ElectraSyn 2.0. Divided H-cells were fabricated in-house. A glass frit (Chemglass, catalog no. 202-05) was used to separate the anodic and cathodic chambers. The anode and cathode (RVC, electrodes) were assembled by affixing them using 2B graphite rods (JuneGold, 2 mm) to ensure good electrical contact.

2. General procedures for chemical synthesis

2A. General procedure for boryl thianthrenium dication **3c** formation

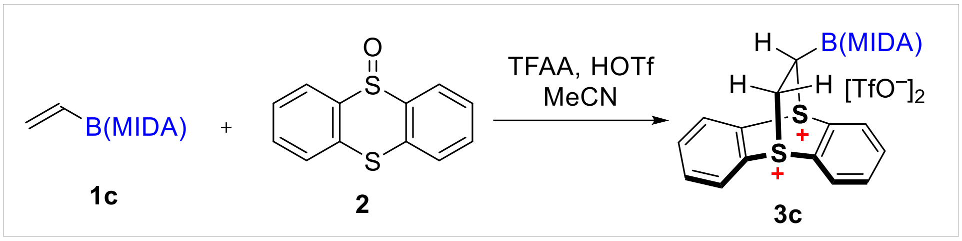

Under nitrogen atmosphere a 50 mL Schlenk vial equipped with a magnetic stir bar was charged with **1c** (1.0 mmol, 1.0 equiv), thianthrene 5-oxide (1.03 equiv), and MeCN (10.0 mL, c = 0.10 M). After cooling to 0 °C, trifluoroacetic anhydride (630 mg, 3.0 mmol, 3.0 equiv) was added dropwise, followed by dropwise addition of TfOH (180 mg, 1.2 mmol, 1.2 equiv). After stirring the mixture at 0 °C for 1 h followed by addition 35mL of ether was added. The resulting slurry was filtered, and the solid was washed with a 10 mL Et_2O and then dried in a vacuum at 23 °C. The solid was **3c** used without further purification.

Representative procedure for large scale synthesis of **3c**: Under nitrogen atmosphere a 1000 mL three neck equipped with a magnetic stir bar was charged with **1c** (18.3 g, 100 mmol, 1.0 equiv), thianthrene 5-oxide (23.92 g, 103 mmol, 1.03 equiv), and MeCN (200 mL, c = 0.5 M). After cooling to 0 °C, trifluoroacetic anhydride (63 g, 300.0 mmol, 3.0 equiv) was added dropwise within 10 minutes, followed by dropwise addition of TfOH (30 g, 200 mmol, 2.0 equiv) by 10 minutes. After stirring the mixture at 0 °C for 1 h followed by an addition 100 mL of dichloromethane and 600 mL of ether was added. The resulting slurry was filtered, and the solid was washed with a 200 mL Et_2O and then dried in a vacuum at 23 °C to afford **3c** as white solid (62.8 g, 90%).

2B. General procedure for the metal-free aziridination

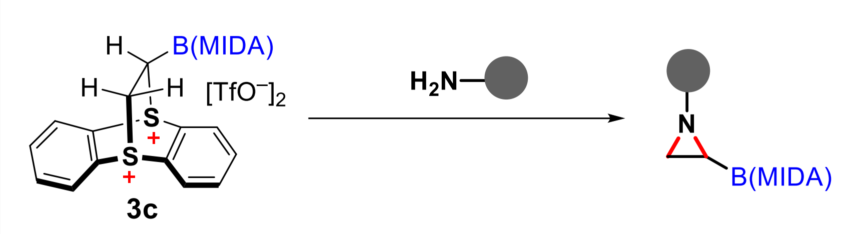

General Procedure A: Sulfonamide (1.2 equiv), K_2CO_3 (5.0 equiv), and **3c** (1.0 equiv) were placed in a 10 mL Schlenk tube which equipped with a magnetic stir bar. After back-filled with nitrogen (this process was repeated three times), DCM (0.1 M) was added. The vial was sealed and at room temperature with stirring until TLC indicated (typically 14-16 h). The mixture was filtered, the filter cake was washed with Dichloromethane (2 × 10 mL), and then the filtrates were combined and concentrated and purified directly by column chromatography to afford the product.

General Procedure B: Amine (or HCL salt) (1.2 equiv), Cs_2CO_3 (3-5.0 equiv), and **3c** (1.0 equiv) were placed in a 10 mL Schlenk tube which equipped with a magnetic stir bar. After back-filled with nitrogen (this process was repeated three times), Acetonitrile (0.1 M) was added. The vial was sealed and at room temperature with stirring until TLC indicated (typically 14-16 h). The mixture was filtered, the filter cake was washed with Dichloromethane (2 × 10 mL), and then the filtrates were combined and concentrated and purified directly by column chromatography to afford the product.

3. General experimental procedures for electrolysis

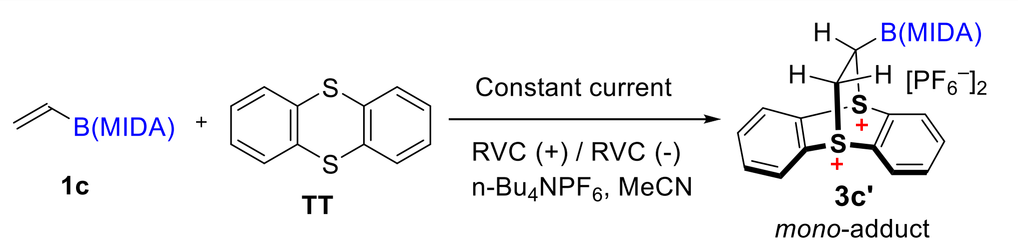

3A. General procedure and experimental setup for boryl thianthrenium dication formation (divided cell)

To an oven dried H-cell (divided cell) containing magnetic stir bars was charged with **1c** (0.3 mmol, 1 equiv), **TT** (0.45 mmol, 1.5 equiv) in 4 mL anhydrous acetonitrile (0.2 M n-Bu_4NPF_6) to the anode compartment and trifluoroacitic acid (0.8 mmol) in 4 mL anhydrous acetonitrile (0.1 M n-Bu_4NPF_6) to the cathode compartment under argon atmosphere. Using stainless steel wire/RVC (12 mm × 5 mm × 3 mm) cathode and pencil/RVC (10 mm × 5 mm × 3 mm) anode, the reaction mixture was electrolyzed under constant current (12 mA) for 5 hours (**Figure S1A**). After the reaction 50 μL of the reaction mixture was transferred

to an NMR tube and diluted with DMSO-d6. The conversion was determined via H^1 NMR using 1,3,5-trimethoxylbenzene as the internal standard. Then, the electrodes were removed, and the reaction mixture was concentrated under reduced pressure. The precipitate was formed by adding diethyl ether to the rection mixture and filtered the precipitate and washed the precipitate with DCM to afford the **3c'**.

3B. General procedure and experimental setup for boryl thianthrenium dication formation (Undivided cell)

To an oven-dried ElectraSyn reaction vial (5 mL) containing magnetic stir bar was charged with **1c** (0.3 mmol, 1 equiv), **TT** (0.45 mmol, 1.5 equiv) and trifluoroacetic acid (0.5 mmol) in 4 mL anhydrous acetonitrile (0.2 M *n*-Bu_4NPF_6) under argon atmosphere. Using a RVC anode and cathode, the reaction mixture was electrolyzed under a constant current of 12 mA for 12 hours (**Figure S1B**). After the reaction 50 μL of the reaction mixture was transferred to an NMR tube and diluted with DMSO-d6. The conversion was determined via H^1 NMR using 1,3,5 trimethoxylbenzene as the internal standard.

3C. General procedure and experimental setup for gram scale boryl thianthrenium dication formation (divided cell)

To an oven dried H-cell (divided cell) containing magnetic stir bars was charged with **1c** (5.5 mmol, 1 equiv), **TT** (8.25 mmol, 1.5 equiv) in 20 mL anhydrous acetonitrile (12.1 mmol, 2.2 equiv, *n*-Bu_4NPF_6) to the anode compartment and trifluoroacitic acid (13.75 mmol, 2.5 equiv) in 20 mL anhydrous acetonitrile (12.1 mmol, 2.2 equiv, *n*-Bu_4NPF_6) to the cathode compartment under argon atmosphere. Using pencil/RVC (3 cm × 5 cm × 6 mm) cathode and pencil/RVC (3.5 cm × 5 cm × 6 mm) anode, the reaction mixture was electrolyzed under constant current (100 mA) for 5 hours. After the reaction 50 μL of the reaction mixture was transferred to an NMR tube and diluted with DMSO-d6. The conversion was determined via ^1H NMR using 1,3,5-trimethoxylbenzene as the internal standard. Then, the electrodes were removed, and the reaction mixture was concentrated under reduced pressure. The precipitate was formed by adding diethyl ether to the rection mixture and filtered the precipitate and washed the precipitate with DCM to afford the **3c'**.

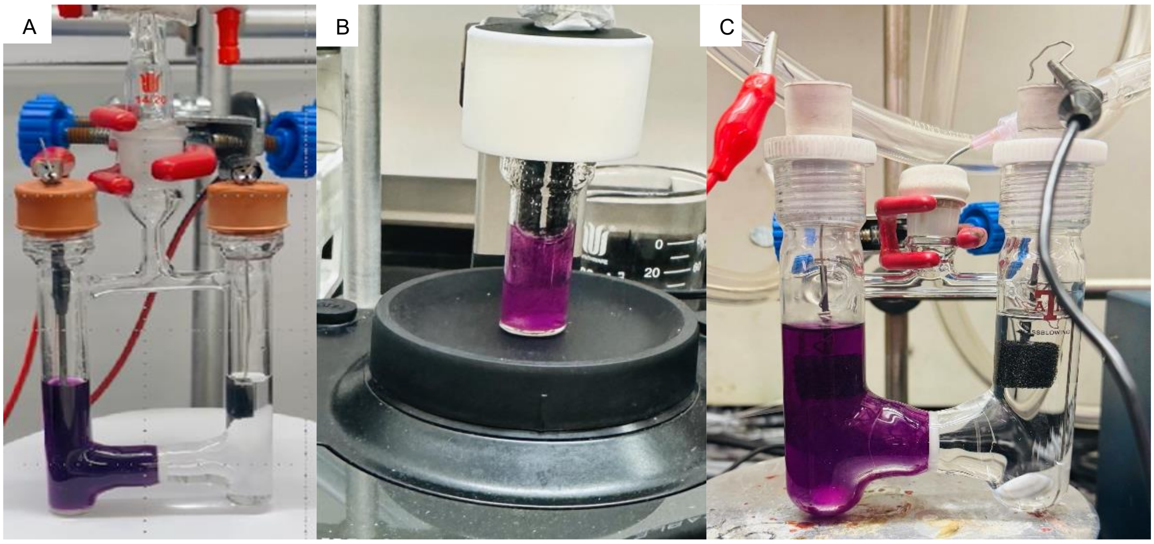

Figure S1. Photographs of the experimental setups for (**A**) divided cell (**B**) commercial IKA ElectraSyn 2.0 and (**C**) divided cell for gram scale reactions.

3D. General procedure for electrochemical one-pot aziridination reaction

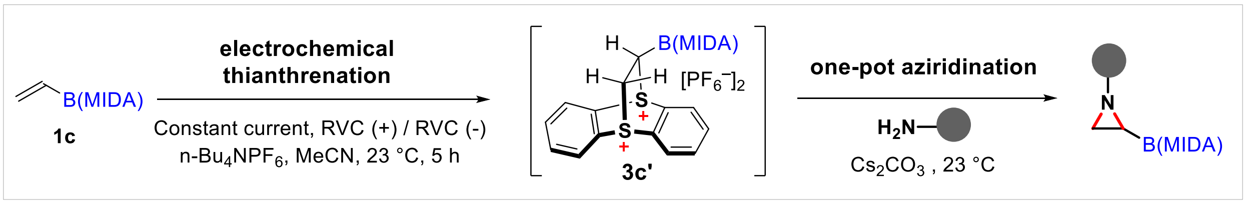

To an oven dried H-cell (divided cell) containing magnetic stir bars was charged with **1c** (0.3 mmol, 1 equiv), **TT** (0.45 mmol, 1.5 equiv) in 4 mL anhydrous acetonitrile (0.2 M *n*-Bu_4NPF_6) to the anode compartment and trifluoroacitic acid (0.8 mmol) in 4 mL anhydrous acetonitrile (0.2 M *n*-Bu_4NPF_6) to the cathode compartment under argon atmosphere. Using stainless steel wire/RVC (12 mm × 5 mm × 3 mm) cathode and pencil/RVC (10 mm × 5 mm × 3 mm) anode, the reaction mixture was electrolyzed under constant current (12 mA) for 5 hours. At the completion of the electrolysis, the electrode on the anode side was removed and Cs_2CO_3 (1 mmol), amine (0.2 mmol) was added to the anode compartment. The anodic compartment was equipped with septa with a needle to prevent pressurizing and after pressure equilibrium, the needle was removed, and cathode solution was removed from the cell using a pipette. The anodic solution was stirred in the cell for 16 hours. The mixture was filtered, the filter cake was washed with dichloromethane (2 × 10 mL), and then the filtrates were combined and concentrated and purified directly by column chromatography to afford the product.

3E. General procedures for cyclic voltammetry

All cyclic voltammograms (CVs) were collected using an electrochemical cell fitted with a 3-mm-diameter glassy carbon disk electrode as the working electrode, a glassy carbon plate as the counter electrode, and an Ag/Ag^{+} electrode as the reference electrode. Following the general procedure, cyclic voltammetry was performed with **1a** (5 mM), **1c** (5 mM), and 1-octene (5mM) with $TBAPF_6$ (0.1M) in anhydrous ACN. The scan rate used here was 0.2 V/s.

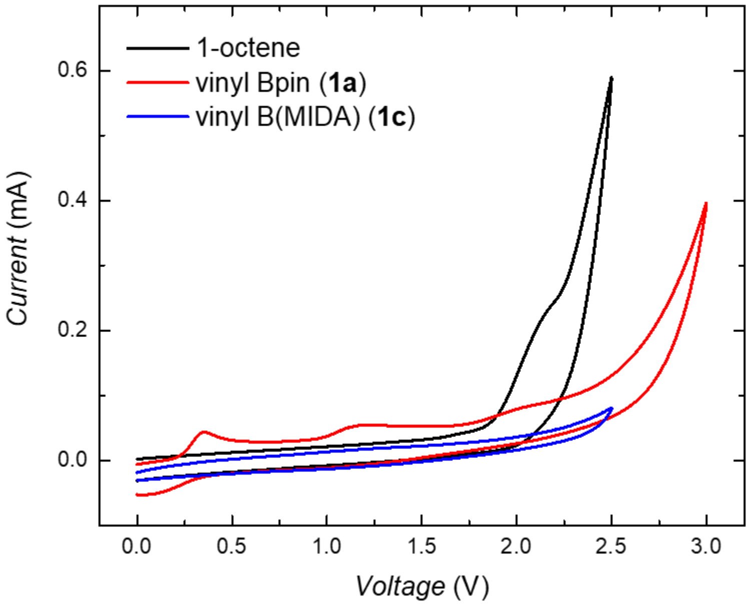

Figure S2. Cyclic voltammograms (CVs) of **1a**, **1c**, and 1-octene.

4. Unsuccessful Substrates

(A) Alkenyl B(MIDA) substrates

(i) Thianthrenation did not occur

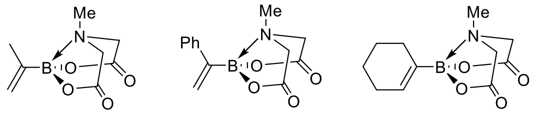

(ii) Thianthrenation proceeded efficiently, but exhibited divergent reactivity with nitrogen nucleophiles, favoring allylic C–N bond formation over aziridination

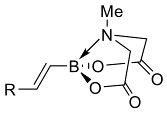

(B) Nitrogen nucleophiles did not undergo aziridination

(i) no reaction

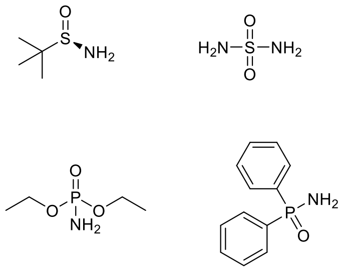

(ii) diamination

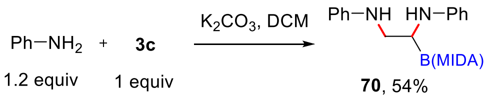

Figure S3. Unsuccessful substrates.

5. Experimental and characterization data

12-(6-methyl-4,8-dioxo-1,3,6,2-dioxazaborocan-2-yl)-5,10-ethanothianthrene-5,10-diium (3c):

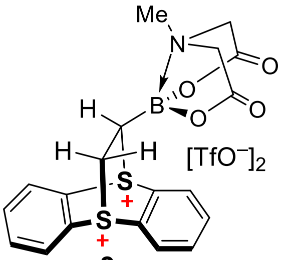

3c

^{1}H NMR (400 MHz, DMSO) δ 8.62 – 8.42 (m, 4H), 8.24 – 8.09 (m, 4H), 4.50 – 4.28 (m, 4H), 4.18 – 4.00 (m, 3H), 2.97 (s, 3H). ^{19}F NMR (377 MHz, DMSO) δ -77.73. ^{11}B NMR (128 MHz, DMSO) δ 9.93. ^{13}C NMR (101 MHz, DMSO) δ 166.14, 165.36, 133.97, 133.79, 133.44, 133.29, 133.09, 132.74, 132.53, 126.43, 126.21, 125.16, 124.29, 120.63, 117.43, 116.44, 61.24, 60.84, 45.24.

12-(6-methyl-4,8-dioxo-1,3,6,2-dioxazaborocan-2-yl)-5,10-ethanothianthrene-5,10-diium (3c'):

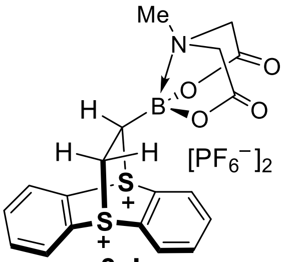

3c'

^{1}H NMR (400 MHz, DMSO) δ 8.56 – 8.44 (m, 4H), 8.19 – 8.11 (m, 4H), 4.46 – 4.33 (m, 4H), 4.13 – 4.02 (m, 3H), 2.96 (s, 3H). ^{19}F NMR (377 MHz, DMSO) δ -69.32, -71.48. ^{11}B NMR (128 MHz, DMSO) δ 9.04. HRMS- ESI (m/z) $[M]^{2+}$ calc'd for $C_{19}H_{18}BNO_4S_2^{2+}$, 199.5380, found 199.5380.

6-methyl-2-(1-((4-(4-(*p*-tolyl)-3-(trifluoromethyl)-1*H*-pyrazol-1-yl)phenyl)sulfonyl)aziridin-2-yl)-1,3,6,2-dioxazaborocane-4,8-dione (5):

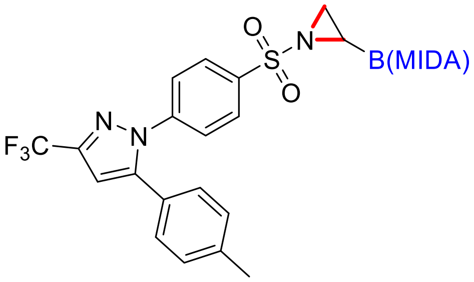

General procedure A with **3c** (210 mg, 0.3 mmol), Celecoxib (137.8 mg, 0.36 mmol, 1.2 equiv.,) and K_2CO_3 (207 mg, 1.5 mmol, 5.0 equiv) to afford a white solid (155 mg, 0.162 mmol, 92% yield). Rf = 0.4 (hexanes:ethyl acetate=4:6). ^{1}H NMR (400 MHz, DMSO) δ 8.09 – 8.00 (m, 2H), 7.70 – 7.61 (m, 2H), 7.33 – 7.22 (m, 5H), 4.36 (dd, *J* = 24.9, 17.1 Hz, 2H), 4.16 (d, *J* = 17.1 Hz, 1H), 3.98 (d, *J* = 17.0 Hz, 1H), 3.11

(s, 3H), 2.48 (d, *J* = 8.4 Hz, 1H), 2.37 (s, 3H), 2.31 (dd, *J* = 8.3, 5.5 Hz, 1H), 2.17 – 2.11 (m, 1H). ^{19}F NMR (377 MHz, DMSO) δ -60.96. ^{11}B NMR (128 MHz, DMSO) δ 12.91. ^{13}C NMR (101 MHz, DMSO) δ 169.38, 168.73, 145.90, 143.23, 143.16, 142.78, 139.73, 137.12, 129.99, 129.66, 129.29, 126.61, 125.72, 123.05, 120.38, 106.94, 62.54, 62.49, 46.71, 30.80, 21.30. HRMS- ESI (m/z) [M+H]+ calc'd for $C_{24}H_{23}BF_3N_4O_6S^+$, 563.1383, found 563.1395.

***N*-(4-((2-(6-methyl-4,8-dioxo-1,3,6,2-dioxazaborocan-2-yl)aziridin-1-yl)sulfonyl)phenyl)acetamide (6):**

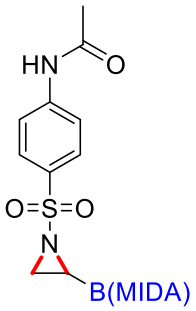

General procedure A with **3c** (210 mg, 0.3 mmol), *N*-(4-sulfamoylphenyl)acetamide (77.3 mg, 0.36 mmol, 1.2 equiv.,) and K_2CO_3 (207 mg, 1.5 mmol, 5.0 equiv) to afford a white solid (71 mg, 60% yield). Rf = 0.3 (hexanes:Acetone 1:1).

^{1}H NMR (400 MHz, DMSO) δ 7.82 (d, *J* = 1.2 Hz, 4H), 4.30 (dd, *J* = 28.2, 17.0 Hz, 2H), 4.09 (d, *J* = 17.1 Hz, 1H), 3.88 (d, *J* = 17.0 Hz, 1H), 3.05 (s, 3H), 2.28 (d, *J* = 8.3 Hz, 1H), 2.12 – 2.07 (m, 4H), 2.00 (d, *J* = 5.3 Hz, 1H). ^{11}B NMR (128 MHz, DMSO) δ 2.12. ^{13}C NMR (101 MHz, DMSO) δ 169.69, 169.46, 168.68, 144.61, 130.02, 129.84, 122.75, 119.55, 119.04, 62.46, 62.41, 46.63, 30.10, 24.66. HRMS- ESI (m/z) [M+H]+ calc'd for $C_{15}H_{19}BN_3O_7S^+$, 396.1037, found 396.1019.

2-(1-((2-aminophenyl)sulfonyl)aziridin-2-yl)-6-methyl-1,3,6,2-dioxazaborocane-4,8-dione (7):

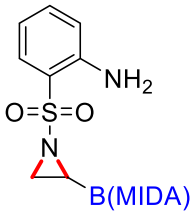

General procedure A with **3c** (210 mg, 0.3 mmol), 2-aminobenzenesulfonamide (62 mg, 0.36 mmol, 1.2 equiv.,) and K_2CO_3 (207 mg, 1.5 mmol, 5.0 equiv) to afford a white solid (58 mg, 55% yield). Rf = 0.2 (hexanes:ethyl acetate=1:9).

^{1}H NMR (400 MHz, DMSO) δ 7.48 (dd, *J* = 8.1, 1.6 Hz, 1H), 7.39 (ddd, *J* = 8.5, 7.1, 1.6 Hz, 1H), 6.90 (dd, *J* = 8.4, 1.1 Hz, 1H), 6.67 (ddd, *J* = 8.2, 7.0, 1.1 Hz, 1H), 6.15 (s, 2H), 4.37 (d, *J* = 17.3 Hz, 1H), 4.26 (d, *J* = 16.9 Hz, 1H), 4.09 (d, *J* = 17.3 Hz, 1H), 3.90 (d, *J* = 16.9 Hz, 1H), 3.02 (s, 3H), 2.34 (d, *J* = 8.3 Hz, 1H),

2.11 – 1.99 (m, 2H). ^{11}B NMR (128 MHz, DMSO) δ 9.21. ^{13}C NMR (101 MHz, DMSO) δ 169.65, 169.54, 168.53, 148.42, 135.51, 130.16, 122.74, 119.54, 117.64, 116.12, 115.71, 62.37, 62.32, 46.46, 29.62. HRMS- ESI (m/z) [M+H]+ calc'd for $C_{13}H_{17}BN_3O_6S^+$, 354.0931, found 354.0928.

6-methyl-2-(1-tosylaziridin-2-yl)-1,3,6,2-dioxazaborocane-4,8-dione (8):

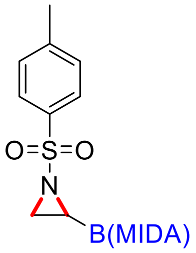

General procedure A with **3c** (210 mg, 0.3 mmol), 4-methylbenzenesulfonamide (62 mg, 0.36 mmol, 1.2 equiv.,) and K_2CO_3 (207 mg, 1.5 mmol, 5.0 equiv) to afford a white solid (76 mg, 72% yield). Rf = 0.4 (hexanes:ethyl acetate=3:7).

^{1}H NMR (400 MHz, DMSO) δ 7.90 – 7.76 (m, 2H), 7.51 (d, *J* = 8.1 Hz, 2H), 4.36 (dd, *J* = 30.4, 17.1 Hz, 2H), 4.15 (d, *J* = 17.2 Hz, 1H), 3.94 (d, *J* = 16.9 Hz, 1H), 3.12 (s, 3H), 2.47 (s, 3H), 2.37 (d, *J* = 8.3 Hz, 1H), 2.20 (dd, *J* = 8.3, 5.4 Hz, 1H), 2.06 (d, *J* = 5.3 Hz, 1H). ^{11}B NMR (128 MHz, DMSO) δ 8.45. ^{13}C NMR (101 MHz, DMSO) δ 167.33, 166.54, 142.93, 132.06, 128.23, 126.33, 60.34, 60.30, 44.50, 28.20, 19.44. HRMS- ESI (m/z) [M+H]+ calc'd for $C_{14}H_{18}BN_2O_6S^+$, 353.0979, found 353.0972.

ethyl 2-((2-(6-methyl-4,8-dioxo-1,3,6,2-dioxazaborocan-2-yl)aziridin-1-yl)sulfonyl)benzoate (9):

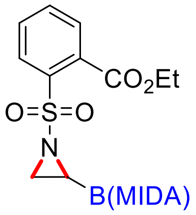

General procedure A with **3c** (210 mg, 0.3 mmol), ethyl 2-sulfamoylbenzoate (83 mg, 0.36 mmol, 1.2 equiv.,) and K_2CO_3 (207 mg, 1.5 mmol, 5.0 equiv) to afford a white solid (85 mg, 69% yield). Rf = 0.4 (hexanes:ethyl acetate=4:6).

^{1}H NMR (400 MHz, DMSO) δ 8.02 (dd, *J* = 7.9, 1.4 Hz, 1H), 7.82 (td, *J* = 7.5, 1.4 Hz, 1H), 7.76 (td, *J* = 7.7, 1.5 Hz, 1H), 7.66 (dd, *J* = 7.5, 1.4 Hz, 1H), 4.39 – 4.23 (m, 4H), 4.08 (d, *J* = 17.1 Hz, 1H), 3.92 (d, *J* = 16.9 Hz, 1H), 3.06 (s, 3H), 2.29 (dd, *J* = 8.3, 5.5 Hz, 1H), 2.10 (d, *J* = 5.5 Hz, 1H), 1.28 (t, *J* = 7.1 Hz, 3H). ^{11}B NMR (128 MHz, DMSO) δ 8.45. ^{13}C NMR (101 MHz, DMSO) δ 169.44, 168.60, 167.24, 134.56, 134.50, 133.89, 131.42, 130.20, 129.02, 62.37, 62.34, 62.32, 46.49, 31.64, 14.24. HRMS- ESI (m/z) [M+H]+ calc'd for $C_{16}H_{20}BN_2O_8S^+$, 411.1033, found 411.1030.

6-methyl-2-(1-((3-nitrophenyl)sulfonyl)aziridin-2-yl)-1,3,6,2-dioxazaborocane-4,8-dione (10):

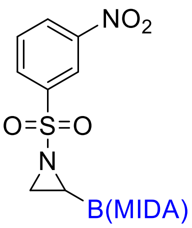

General procedure A with **3c** (210 mg, 0.3 mmol), 3-nitrobenzenesulfonamide (73 mg, 0.36 mmol, 1.2 equiv.,) and K_2CO_3 (207 mg, 1.5 mmol, 5.0 equiv) to afford a white solid (100 mg, 88% yield). Rf = 0.4 (hexanes:ethyl acetate=4:6).

^{1}H NMR (400 MHz, DMSO) δ 8.59 (ddd, *J* = 8.2, 2.3, 1.0 Hz, 1H), 8.55 (t, *J* = 2.0 Hz, 1H), 8.36 (dt, *J* = 8.0, 1.3 Hz, 1H), 7.97 (t, *J* = 8.0 Hz, 1H), 4.30 (dd, *J* = 22.7, 17.1 Hz, 2H), 4.11 (d, *J* = 17.1 Hz, 1H), 3.99 (d, *J* = 17.0 Hz, 1H), 3.08 (s, 3H), 2.54 (d, *J* = 8.4 Hz, 1H), 2.35 (dd, *J* = 8.4, 5.6 Hz, 1H), 2.13 (d, *J* = 5.6 Hz, 1H). ^{13}C NMR (101 MHz, DMSO) δ 168.99, 168.37, 148.07, 138.54, 133.96, 131.74, 128.67, 122.63, 122.33, 119.13, 62.19, 62.17, 46.36, 38.89, 30.83.^{11}B NMR (128 MHz, DMSO-d6). HRMS-ESI (m/z) [M+H]+ calc'd for $C_{13}H_{15}O_8N_3BS$ 384.0667; found 384.0666.

6-methyl-2-(1-((2-nitrophenyl)sulfonyl)aziridin-2-yl)-1,3,6,2-dioxazaborocane-4,8-dione (11):

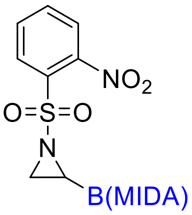

General procedure A with **3c** (210 mg, 0.3 mmol), 2-nitrobenzenesulfonamide (73 mg, 0.36 mmol, 1.2 equiv.,) and K_2CO_3 (207 mg, 1.5 mmol, 5.0 equiv) to afford a white solid (87 mg, 75% yield). Rf = 0.4 (hexanes:ethyl acetate=3:7).

^{1}H NMR (400 MHz, DMSO) δ 8.16 (dd, *J* = 7.8, 1.4 Hz, 1H), 8.06 (dd, *J* = 8.0, 1.4 Hz, 1H), 8.00 (td, *J* = 7.7, 1.4 Hz, 1H), 7.92 (td, *J* = 7.7, 1.4 Hz, 1H), 4.34 (dd, *J* = 30.2, 17.0 Hz, 2H), 4.11 (d, *J* = 17.2 Hz, 1H), 3.89 (d, *J* = 16.9 Hz, 1H), 3.10 (s, 3H), 2.60 (d, *J* = 8.4 Hz, 1H), 2.46 (dd, *J* = 8.4, 5.6 Hz, 1H), 2.23 (d, *J* = 5.6 Hz, 1H). ^{13}C NMR (101 MHz, DMSO) δ 168.98, 167.96, 148.01, 135.88, 132.74, 130.83, 129.23, 124.56, 61.89, 61.84, 46.06, 32.21. ^{11}B NMR (128 MHz, DMSO-d6). HRMS-ESI (m/z) [M+H]+ calc'd for $C_{13}H_{15}O_8N_3BS$ 384.0667; found 384.0674.

2-(1-((2-bromophenyl)sulfonyl)aziridin-2-yl)-6-methyl-1,3,6,2-dioxazaborocane-4,8-dione (12):

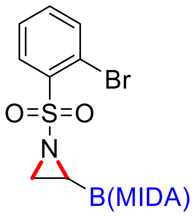

General procedure A with **3c** (210 mg, 0.3 mmol), 2-bromobenzenesulfonamide (85 mg, 0.36 mmol, 1.2 equiv.,) and K_2CO_3 (207 mg, 1.5 mmol, 5.0 equiv) to afford a white solid (91 mg, 73% yield). Rf = 0.4 (hexanes:ethyl acetate=4:6).

^{1}H NMR (400 MHz, DMSO) δ 8.04 (dd, *J* = 6.0, 3.5 Hz, 1H), 7.95 – 7.90 (m, 1H), 7.69 – 7.59 (m, 2H), 4.32 (dd, *J* = 45.9, 17.1 Hz, 2H), 4.10 (d, *J* = 17.2 Hz, 1H), 3.90 (d, *J* = 16.9 Hz, 1H), 3.11 (s, 3H), 2.52 (s, 1H), 2.39 (dd, *J* = 8.3, 5.5 Hz, 1H), 2.10 (d, *J* = 5.4 Hz, 1H). ^{13}C NMR (101 MHz, DMSO) δ 169.09, 167.93, 137.10, 135.84, 135.38, 131.19, 128.43, 120.56, 61.98, 61.87, 46.12, 31.56. ^{11}B NMR (128 MHz, DMSO-d6). **HRMS- ESI** (m/z) [M+H]+ calc'd for $C_{13}H_{15}O_6N_2BBrS$ 416.9922; found 416.9922.

2-(1-((2-chlorophenyl)sulfonyl)aziridin-2-yl)-6-methyl-1,3,6,2-dioxazaborocane-4,8-dione (13):

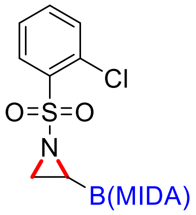

General procedure A with **2** (210 mg, 0.3 mmol), 2-chlorobenzenesulfonamide (70 mg, 0.36 mmol, 1.2 equiv.,) and K_2CO_3 (207 mg, 1.5 mmol, 5.0 equiv) to afford a white solid (87 mg, 78% yield). Rf = 0.4 (hexanes:ethyl acetate=4:6).

^{1}H NMR (400 MHz, DMSO) δ 8.03 (dt, *J* = 7.9, 1.1 Hz, 1H), 7.79 – 7.71 (m, 2H), 7.65 – 7.54 (m, 1H), 4.32 (dd, *J* = 41.5, 17.0 Hz, 2H), 4.10 (d, *J* = 17.2 Hz, 1H), 3.85 (d, *J* = 16.9 Hz, 1H), 3.09 (s, 3H), 2.53 (d, *J* = 8.4 Hz, 1H), 2.39 (dd, *J* = 8.4, 5.6 Hz, 1H), 2.13 (d, *J* = 5.5 Hz, 1H). ^{13}C NMR (101 MHz, DMSO) δ 169.06, 167.95, 135.47, 135.37, 132.34, 131.96, 130.99, 127.96, 61.85, 61.81, 46.05, 31.57. ^{11}B NMR (128 MHz, DMSO-d6). HRMS-ESI (m/z) [M+H]+ calc'd for $C_{13}H_{15}O_6N_2BClS$ 373.0427; found 373.0428.

6-methyl-2-(1-(thiophen-2-ylsulfonyl)aziridin-2-yl)-1,3,6,2-dioxazaborocane-4,8-dione (14):

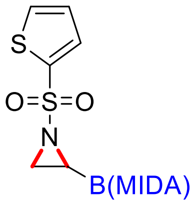

General procedure A with **3c** (210 mg, 0.3 mmol), thiophene-2-sulfonamide (59 mg, 0.36 mmol, 1.2 equiv.,) and K_2CO_3 (207 mg, 1.5 mmol, 5.0 equiv) to afford a white solid (73 mg, 70% yield). Rf = 0.4 (hexanes:ethyl acetate=4:6).

^1H NMR (400 MHz, DMSO) δ 8.14 (dd, J = 5.0, 1.3 Hz, 1H), 7.79 (dd, J = 3.8, 1.4 Hz, 1H), 7.29 (dd, J = 5.0, 3.8 Hz, 1H), 4.31 (dd, J = 24.2, 17.1 Hz, 2H), 4.10 (d, J = 17.1 Hz, 1H), 3.90 (d, J = 17.0 Hz, 1H), 3.05 (s, 3H), 2.34 (d, J = 8.2 Hz, 1H), 2.21 – 2.09 (m, 2H). ^{13}C NMR (101 MHz, DMSO) δ 169.40, 168.69, 136.12, 135.92, 135.30, 128.68, 62.55, 62.50, 55.38, 46.72, 30.64, 29.48. ^{11}B NMR (128 MHz, DMSO-d6). HRMS-ESI (m/z) [M+H]+ calc'd for $C_{11}H_{14}O_6N_2BS_2$ 345.0381; found 345.0382.

6-methyl-2-(1-(pyridin-2-ylsulfonyl)aziridin-2-yl)-1,3,6,2-dioxazaborocane-4,8-dione (15):

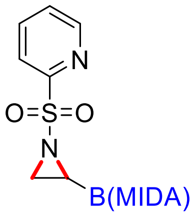

General procedure A with **3c** (210 mg, 0.3 mmol), pyridine-2-sulfonamide (57.1 mg, 0.36 mmol, 1.2 equiv.,) and K_2CO_3 (207 mg, 1.5 mmol, 5.0 equiv) to afford a white solid (45 mg, 45% yield). Rf = 0.4 (hexanes:Acetone 3:7).

^1H NMR (400 MHz, DMSO) δ 8.86 (ddd, J = 4.7, 1.7, 0.9 Hz, 1H), 8.22 (td, J = 7.7, 1.7 Hz, 1H), 8.13 (dt, J = 7.9, 1.1 Hz, 1H), 7.84 (ddd, J = 7.6, 4.7, 1.2 Hz, 1H), 4.36 (dd, J = 29.9, 17.1 Hz, 2H), 4.17 (d, J = 17.1 Hz, 1H), 3.96 (d, J = 17.0 Hz, 1H), 3.16 (s, 3H), 2.64 (d, J = 8.3 Hz, 1H), 2.38 (dd, J = 8.3, 5.6 Hz, 1H), 2.21 (d, J = 5.5 Hz, 1H). ^{11}B NMR (128 MHz, DMSO) δ 8.44. ^{13}C NMR (101 MHz, DMSO) δ 169.42, 168.69, 155.21, 150.87, 139.55, 128.69, 123.61, 62.47, 46.71, 31.31. HRMS-ESI (m/z) [M+H]+ calc'd for $C_{12}H_{15}BN_3O_6S^+$, 340.0775, found 340.0772.

2-(1-(benzylsulfonyl)aziridin-2-yl)-6-methyl-1,3,6,2-dioxazaborocane-4,8-dione (16):

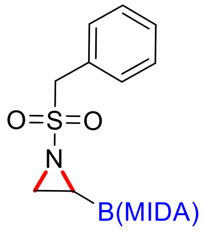

General procedure A with **3c** (210 mg, 0.3 mmol), phenylmethanesulfonamide (62 mg, 0.36 mmol, 1.2 equiv.,) and K_2CO_3 (207 mg, 1.5 mmol, 5.0 equiv) to afford a white solid (120 mg, 61% yield). Rf = 0.45 (hexanes:ethyl acetate=1:9).

^1H NMR (400 MHz, DMF) δ 7.65 – 7.51 (m, 5H), 4.81 (s, 2H), 4.50 (d, J = 17.2 Hz, 1H), 4.39 (d, J = 16.8 Hz, 1H), 4.24 (d, J = 17.1 Hz, 1H), 3.95 (d, J = 16.9 Hz, 1H), 3.22 (s, 3H), 2.42 (d, J = 8.2 Hz, 1H), 2.30

(dd, J = 8.2, 5.5 Hz, 1H), 2.20 (d, J = 5.4 Hz, 1H). ^{11}B NMR (128 MHz, DMSO) δ 9.67. ^{13}C NMR (101 MHz, DMF) δ 169.94, 169.07, 131.94, 129.83, 129.24, 129.22, 62.77, 57.83, 46.94, 31.82. HRMS-ESI (m/z) [M+H]+ calc'd for $C_{14}H_{18}BN_2O_6S^+$, 353.0979, found 353.2078.

2-(1-(cyclopropylsulfonyl)aziridin-2-yl)-6-methyl-1,3,6,2-dioxazaborocane-4,8-dione (17):

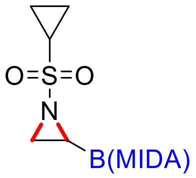

General procedure A with **3c** (210 mg, 0.3 mmol), cyclopropanesulfonamide (40 mg, 0.36 mmol, 1.2 equiv.,) and K_2CO_3 (207 mg, 1.5 mmol, 5.0 equiv) to afford a white solid (68 mg, 75% yield). Rf = 0.4 (hexanes:ethyl acetate=4:6).

^{1}H NMR (400 MHz, DMSO) δ 4.34 (dd, J = 31.2, 17.1 Hz, 2H), 4.13 (d, J = 17.1 Hz, 1H), 3.95 (d, J = 16.9 Hz, 1H), 3.12 (s, 3H), 2.84 (tt, J = 7.9, 4.9 Hz, 1H), 2.44 – 2.38 (m, 1H), 2.15 – 2.11 (m, 2H), 1.15 – 1.01 (m, 4H). ^{11}B NMR (128 MHz, DMSO) δ 8.19. ^{13}C NMR (101 MHz, DMSO) δ 167.40, 166.66, 60.33, 60.28, 28.06, 26.27, 3.30, 3.07. HRMS-ESI (m/z) [M+H]+ calc'd for $C_{10}H_{15}BN_2O_6S^+$, 303.0822, found 303.0808.

6-methyl-2-(1-(pyrrolidin-1-ylsulfonyl)aziridin-2-yl)-1,3,6,2-dioxazaborocane-4,8-dione (18):

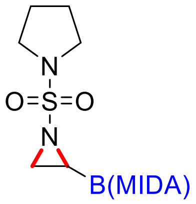

General procedure A with **3c** (210 mg, 0.3 mmol), pyrrolidine-1-sulfonamide (54.2mg, 0.36 mmol, 1.2 equiv.,) and K_2CO_3 (207 mg, 1.5 mmol, 5.0 equiv) to afford a white solid (74 mg, 75% yield). Rf = 0.5 (hexanes:ethyl acetate = 2:8).

^{1}H NMR (400 MHz, DMSO) δ 4.28 (d, J = 17.2 Hz, 1H), 4.17 (d, J = 16.8 Hz, 1H), 4.00 (d, J = 17.2 Hz, 1H), 3.83 (d, J = 16.9 Hz, 1H), 3.25 – 3.20 (m, 4H), 3.00 (s, 3H), 2.29 (d, J = 8.4 Hz, 1H), 1.97 (d, J = 5.3 Hz, 1H), 1.85 – 1.75 (m, 5H). ^{11}B NMR (128 MHz, DMSO) δ 8.77. ^{13}C NMR (101 MHz, DMSO) δ 169.70, 168.57, 62.24, 62.20, 46.51, 45.52, 29.43, 23.27, 11.60. HRMS-ESI (m/z) [M+H]+ calc'd for $C_{11}H_{20}BN_3O_6S^+$, 332.1088, found 332.1084.

2-(6-methyl-4,8-dioxo-1,3,6,2-dioxazaborocan-2-yl)-*N*-propylaziridine-1-sulfonamide (19):

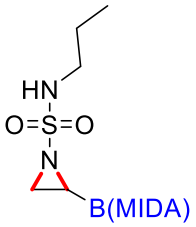

General procedure A with **3c** (210 mg, 0.3 mmol), propane-1-sulfonamide (44 mg, 0.36 mmol, 1.2 equiv) and K_2CO_3 (207 mg, 1.5 mmol, 5.0 equiv) to afford a white solid (60 mg, 67% yield). Rf = 0.4 (hexanes:ethyl acetate=4:6).

^{1}H NMR (400 MHz, DMSO) δ 7.32 (t, *J* = 5.8 Hz, 1H), 4.29 (d, *J* = 17.2 Hz, 1H), 4.17 (d, *J* = 16.8 Hz, 1H), 4.00 (d, *J* = 17.2 Hz, 1H), 3.75 (d, *J* = 16.8 Hz, 1H), 3.02 (s, 3H), 2.90 (td, *J* = 7.2, 5.9 Hz, 2H), 2.15 (d, *J* = 8.3 Hz, 1H), 1.88 (d, *J* = 5.1 Hz, 1H), 1.78 (dd, *J* = 8.1, 5.2 Hz, 1H), 1.40 (h, *J* = 7.3 Hz, 2H), 0.79 (t, *J* = 7.4 Hz, 3H). ^{11}B NMR (128 MHz, DMSO) δ 9.21. ^{13}C NMR (101 MHz, DMSO) δ 169.70, 168.57, 62.24, 62.20, 46.51, 45.52, 29.43, 23.27, 11.60. HRMS-ESI (m/z) [M+H]+ calc'd for $C_{10}H_{19}BN_3O_6S^+$, 320.1088, found 320.1081.

4-nitrobenzyl (2*S*,4*S*)-4-(acetylthio)-2-(((*N*-(*tert*-butoxycarbonyl)-2-(6-methyl-4,8-dioxo-1,3,6,2-dioxazaborocan-2-yl)aziridine)-1-sulfonamido)methyl)pyrrolidine-1-carboxylate (20):

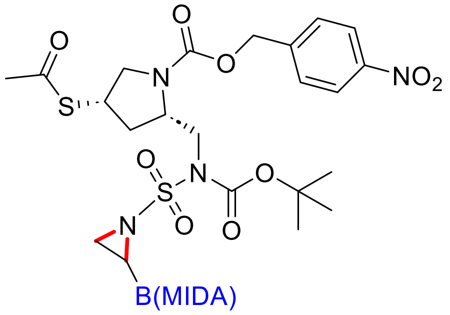

General procedure A with **3c** (210 mg, 0.3 mmol), Doripenem side chain (192.3 mg, 0.36 mmol, 1.2 equiv) and K_2CO_3 (207 mg, 1.5 mmol, 5.0 equiv) to afford a white solid (145 mg, 68% yield). Rf = 0.3 (Dichloromrthane:Methanol=9:1).

^{1}H NMR (400 MHz, DMSO) δ 8.20 (d, *J* = 8.2 Hz, 2H), 7.62 (d, *J* = 8.5 Hz, 2H), 5.42 – 4.92 (m, 2H), 4.49 – 3.63 (m, 9H), 3.08 (s, 4H), 2.67 (d, *J* = 8.0 Hz, 1H), 2.49 – 2.41 (m, 1H), 2.33 (d, *J* = 1.5 Hz, 3H), 2.29 – 2.14 (m, 2H), 1.69 (s, 1H), 1.41 (t, *J* = 10.6 Hz, 9H). ^{11}B NMR (128 MHz, DMSO) δ 9.21. ^{13}C NMR (101 MHz, DMSO) δ 195.54, 195.51, 169.44, 168.57, 154.22, 151.00, 150.97, 147.41, 145.32, 128.66, 123.95, 84.58, 65.50, 62.40, 52.27, 46.67, 33.41, 30.95, 30.93, 27.90, 1.63. HRMS-ESI (m/z) [M+H]+ calc'd for $C_{27}H_{37}BN_5O_{13}S_2^+$, 714.1922, found 714.1923.

6-methyl-2-(1-(3-nitrobenzyl)aziridin-2-yl)-1,3,6,2-dioxazaborocane-4,8-dione (21):

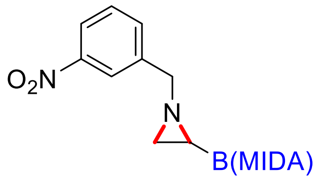

General procedure B with **3c** (210 mg, 0.3 mmol), (3,4-dichlorophenyl)methanamine (63.2 mg, 0.36 mmol, 1.2 equiv) and Cs_2CO_3 (295 mg, 0.9 mmol, 3.0 equiv) to afford a white solid (77 mg, 73% yield). Rf = 0.2 (Dichloromethane:MeOH=9:1).

^{1}H NMR (400 MHz, DMSO) δ 8.23 (t, *J* = 2.0 Hz, 1H), 8.13 (ddd, *J* = 8.2, 2.4, 1.1 Hz, 1H), 7.82 (dt, *J* = 7.7, 1.4 Hz, 1H), 7.63 (t, *J* = 7.9 Hz, 1H), 4.27 (d, *J* = 17.2 Hz, 1H), 4.14 (d, *J* = 16.8 Hz, 1H), 4.01 (d, *J* = 17.2 Hz, 1H), 3.96 (d, *J* = 13.6 Hz, 1H), 3.89 (d, *J* = 16.8 Hz, 1H), 2.99 (d, *J* = 13.6 Hz, 1H), 2.92 (s, 3H), 1.54 (dd, *J* = 4.3, 1.6 Hz, 1H), 1.40 (dd, *J* = 7.4, 1.7 Hz, 1H), 0.82 (dd, *J* = 7.5, 4.3 Hz, 1H). ^{13}C NMR (101 MHz, DMSO) δ 169.49, 168.49, 147.73, 142.39, 134.96, 129.64, 122.63, 121.83, 63.91, 61.90, 61.68, 48.59, 46.20, 30.67. ^{11}B NMR (128 MHz, DMSO) δ 13.26.). HRMS-ESI (m/z) [M+H]+ calc'd for $C_{14}H_{16}O_6N_3B$ 334.1205; found 334.1207.

3-((2-(6-methyl-4,8-dioxo-1,3,6,2-dioxazaborocan-2-yl)aziridin-1-yl)methyl)benzonitrile (22):

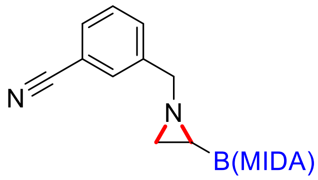

General procedure B with **3c** (210 mg, 0.3 mmol), 3-(aminomethyl)benzonitrile (47.7 mg, 0.36 mmol, 1.2 equiv) and Cs_2CO_3 (295 mg, 0.9 mmol, 3.0 equiv) to afford a white solid (42 mg, 45% yield). Rf = 0.5 (Dichloromethane:MeOH=9:1).

^{1}H NMR (400 MHz, DMSO) δ 7.74 (s, 1H), 7.65 (t, *J* = 7.5 Hz, 2H), 7.48 (t, *J* = 7.7 Hz, 1H), 4.20 (d, *J* = 17.2 Hz, 1H), 4.07 (d, *J* = 16.8 Hz, 1H), 3.94 (d, *J* = 17.2 Hz, 1H), 3.85 – 3.73 (m, 2H), 2.86 (d, *J* = 14.4 Hz, 4H), 1.46 (d, *J* = 4.1 Hz, 1H), 1.35 – 1.28 (m, 1H), 0.73 (dd, *J* = 7.4, 4.3 Hz, 1H).^{13}C NMR (101 MHz, DMSO) δ 169.51, 168.48, 141.67, 133.23, 131.73, 130.69, 129.39, 118.92, 111.11, 64.07, 61.90, 61.66, 54.91, 46.13, 30.73, 30.70. ^{11}B NMR (128 MHz, DMSO) δ 11.73. HRMS-ESI (m/z) [M+H]+ calc'd for $C_{15}H_{17}O_4N_3B$ 314.1307; found 312.1297.

6-methyl-2-(1-(4-(trifluoromethyl)benzyl)aziridin-2-yl)-1,3,6,2-dioxazaborocane-4,8-dione (23):

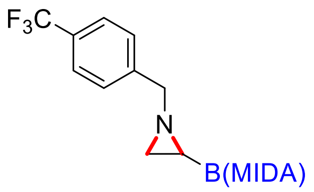

General procedure B with **3c** (210 mg, 0.3 mmol), (4-(trifluoromethyl)phenyl)methanamine (63.3 mg, 0.36 mmol, 1.2 equiv) and Cs_2CO_3 (295 mg, 0.9 mmol, 3.0 equiv) to afford a white solid (73 mg, 68% yield). Rf = 0.5 (Dichloromethane:MeOH=9:1).

^{1}H NMR (400 MHz, MeOD) δ 7.64 (d, *J* = 8.2 Hz, 2H), 7.59 (d, *J* = 8.2 Hz, 2H), 4.13 (dd, *J* = 20.7, 17.0 Hz, 2H), 4.00 (d, *J* = 17.1 Hz, 1H), 3.86 (d, *J* = 16.8 Hz, 1H), 3.64 (d, *J* = 13.6 Hz, 1H), 3.42 (d, *J* = 13.7 Hz, 1H), 2.85 (s, 3H), 1.86 (d, *J* = 4.5 Hz, 1H), 1.60 (d, *J* = 7.6 Hz, 1H), 0.97 (dd, *J* = 7.7, 4.5 Hz, 1H). 19F NMR (376 MHz, MeOD) δ -63.87. ^{11}B NMR (128 MHz, MeOD) δ 10.34. 13C NMR (101 MHz, DMSO) δ 169.97, 168.90, 145.34, 129.76, 129.30, 128.14, 127.83, 127.51, 126.21, 125.88, 125.49, 125.46, 125.42, 125.38, 123.51, 64.94, 62.33, 62.11, 46.50, 31.21.. HRMS-ESI (m/z) [M+H]+ calc'd for $C_{15}H_{17}O_4N_2BF_3$ 357.1228; found 357.1222.

2-(1-(3,4-dichlorobenzyl)aziridin-2-yl)-6-methyl-1,3,6,2-dioxazaborocane-4,8-dione (24):

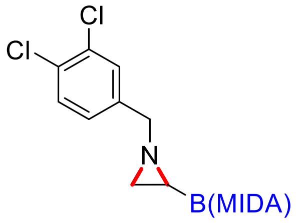

General procedure B with **3c** (210 mg, 0.3 mmol), (3,4-dichlorophenyl)methanamine (63.2 mg, 0.36 mmol, 1.2 equiv.,) and Cs_2CO_3 (295 mg, 0.9 mmol, 3.0 equiv) to afford a white solid (65 mg, 61% yield). Rf = 0.2 (Dichloromethane:MeOH=9:1).

^{1}H NMR (400 MHz, DMSO) δ 7.57 – 7.49 (m, 2H), 7.29 (dt, *J* = 8.3, 1.7 Hz, 1H), 4.20 (dd, *J* = 17.2, 1.3 Hz, 1H), 4.07 (dd, *J* = 16.8, 1.3 Hz, 1H), 3.99 – 3.93 (m, 1H), 3.86 – 3.69 (m, 2H), 2.85 (s, 3H), 2.79 (d, *J* = 13.5 Hz, 1H), 1.49 – 1.43 (m, 1H), 1.28 (dd, *J* = 7.4, 1.6 Hz, 1H), 0.71 (dd, *J* = 7.5, 4.3 Hz, 1H). ^{11}B NMR (128 MHz, DMSO) δ 9.92. ^{13}C NMR (101 MHz, DMSO) δ 169.97, 168.94, 141.75, 131.19, 130.76, 130.54, 129.76, 129.00, 64.06, 62.36, 62.13, 46.61, 31.12. HRMS- ESI (m/z) [M+H]+ calc'd for $C_{14}H_{16}BCl_2N_2O_4$+, 357.0580, found 357.0576.

6-methyl-2-(1-(4-(4,4,5,5-tetramethyl-1,3,2-dioxaborolan-2-yl)benzyl)aziridin-2-yl)-1,3,6,2-dioxazaborocane-4,8-dione (25):

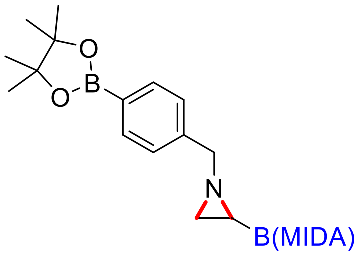

General procedure B with **3c** (210 mg, 0.3 mmol), (4-(4,4,5,5-tetramethyl-1,3,2-dioxaborolan-2-yl)phenyl)methanamine (84.3 mg, 0.36 mmol, 1.2 equiv) and Cs_2CO_3 (295 mg, 0.9 mmol, 3.0 equiv) to afford a white solid (76 mg, 61% yield). Rf = 0.5 (Dichloromethane:MeOH=9:1).

^{1}H NMR (400 MHz, DMSO) δ 7.56 (d, *J* = 7.7 Hz, 2H), 7.29 (d, *J* = 7.7 Hz, 2H), 4.18 (d, *J* = 17.3 Hz, 1H), 4.04 (d, *J* = 16.7 Hz, 1H), 3.91 (dd, *J* = 17.1, 8.0 Hz, 1H), 3.70 (d, *J* = 16.7 Hz, 1H), 3.61 (d, *J* = 13.3 Hz, 1H), 2.96 (d, *J* = 13.4 Hz, 1H), 2.73 (s, 3H), 1.50 – 1.42 (m, 1H), 1.22 (s, 13H), 0.67 (dd, *J* = 7.5, 4.2 Hz, 1H). ^{11}B NMR (128 MHz, DMSO) δ 13.72, 1.14. ^{13}C NMR (101 MHz, DMSO) δ 169.98, 169.64, 168.86, 143.83, 134.84, 128.27, 84.04, 65.79, 62.27, 62.06, 46.35, 31.13, 25.16, 25.14. HRMS- ESI (m/z) [M+H]+ calc'd for $C_{20}H_{29}O_6N_2B_2$ 415.2212; found 415.2174.

6-methyl-2-(1-((3-phenyl-1,2,4-oxadiazol-5-yl)methyl)aziridin-2-yl)-1,3,6,2-dioxazaborocane-4,8-dione (26):

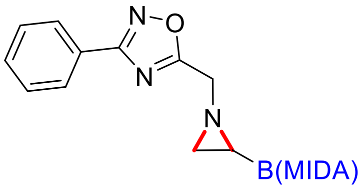

General procedure B with **3c** (210 mg, 0.3 mmol), (3-phenyl-1,2,4-oxadiazol-5-yl)methanamine (63.3 mg, 0.36 mmol, 1.2 equiv) and Cs_2CO_3 (295 mg, 0.9 mmol, 3.0 equiv) to afford a white solid (59 mg, 55% yield). Rf = 0.6 (Dichloromethane:MeOH=9:1).

^{1}H NMR (400 MHz, DMSO) δ 8.07 – 7.95 (m, 2H), 7.65 – 7.51 (m, 3H), 4.29 (d, *J* = 17.2 Hz, 1H), 4.19 (d, *J* = 16.7 Hz, 1H), 4.10 (d, *J* = 15.2 Hz, 1H), 4.03 (d, *J* = 12.5 Hz, 1H), 3.99 (d, *J* = 12.0 Hz, 1H), 3.50 (d, *J* = 15.3 Hz, 1H), 3.00 (s, 3H), 1.66 (dd, *J* = 4.5, 1.3 Hz, 1H), 1.60 (dd, *J* = 7.7, 1.2 Hz, 1H), 0.96 (dd, *J* = 7.6, 4.4 Hz, 1H). ^{13}C NMR (101 MHz, DMSO) δ 177.92, 169.46, 168.64, 168.48, 167.55, 131.65, 129.35, 127.05, 126.11, 61.72, 61.70, 61.40, 55.42, 48.62, 46.06, 30.58. 11B NMR (128 MHz, DMSO) δ 5.20. HRMS- ESI (m/z) [M+H]+ calc'd for $C_{16}H_{18}O_5N_4B$ 357.1365; found 357.1364.

6-methyl-2-(1-((6-(trifluoromethyl)pyridin-3-yl)methyl)aziridin-2-yl)-1,3,6,2-dioxazaborocane-4,8-dione (27):

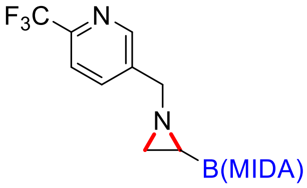

General procedure B with **3c** (210 mg, 0.3 mmol), (6-(trifluoromethyl)pyridin-3-yl)methanamine (63.6 mg, 0.36 mmol, 1.2 equiv) and Cs_2CO_3 (295 mg, 0.9 mmol, 3.0 equiv) to afford a white solid (66 mg, 62% yield). Rf = 0.2 (Dichloromethane:MeOH=9:1). f

^{1}H NMR (400 MHz, DMSO) δ 8.66 (dd, *J* = 5.3, 2.0 Hz, 1H), 8.00 (td, *J* = 7.9, 2.1 Hz, 1H), 7.81 (d, *J* = 8.2 Hz, 1H), 4.22 (d, *J* = 17.3 Hz, 1H), 4.09 (d, *J* = 16.8 Hz, 1H), 3.97 (d, *J* = 17.2 Hz, 1H), 3.91 – 3.80 (m, 2H), 2.92 (d, *J* = 20.3 Hz, 4H), 1.48 (dd, *J* = 4.3, 1.6 Hz, 1H), 1.35 (dd, *J* = 7.4, 1.6 Hz, 1H), 0.79 (dd, *J* = 7.5, 4.3 Hz, 1H). ^{19}F NMR (376 MHz, DMSO) δ -66.19. ^{11}B NMR (128 MHz, DMSO) δ 13.89. ^{13}C NMR (101 MHz, DMSO) δ 169.94, 168.98, 150.30, 149.86, 145.72, 145.39, 140.01, 138.17, 137.25, 62.40, 62.14, 55.35, 46.68, 31.13. HRMS (ESI-MS) m/z: $[M+H]^+$ Calcd for $C_{14}H_{16}O_4N_3BF_3$ 358.1186; found 358.1175.

2-(1-((6-chloropyridin-3-yl)methyl)aziridin-2-yl)-6-methyl-1,3,6,2-dioxazaborocane-4,8-dione (28):

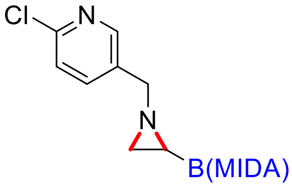

General procedure B with **3c** (210 mg, 0.3 mmol), (6-chloropyridin-3-yl)methanamine (52 mg, 0.36 mmol, 1.2 equiv) and Cs_2CO_3 (295 mg, 0.9 mmol, 3.0 equiv) to afford a white solid (76 mg, 75% yield). Rf = 0.2 (Dichloromethane:MeOH=9:1).

^{1}H NMR (400 MHz, DMSO) δ 7.98 (d, *J* = 2.4 Hz, 1H), 7.47 (dd, *J* = 8.2, 2.5 Hz, 1H), 7.09 (d, *J* = 8.2 Hz, 1H), 3.81 (dd, *J* = 49.9, 17.0 Hz, 2H), 3.63 (d, *J* = 17.2 Hz, 1H), 3.55 – 3.36 (m, 2H), 2.54 (s, 3H), 1.12 (dd, *J* = 4.2, 1.6 Hz, 1H), 0.99 (dd, *J* = 7.5, 1.6 Hz, 1H), 0.42 (dd, *J* = 7.4, 4.3 Hz, 1H). ^{11}B NMR (128 MHz, DMSO) δ 12.24. ^{13}C NMR (101 MHz, DMSO) δ 169.95, 168.96, 149.93, 149.27, 140.22, 135.47, 124.37, 62.38, 62.14, 55.37, 46.65, 31.04. HRMS- ESI (m/z) [M+H]+ calc’d for $C_{13}H_{16}O_4N_3BCl$ 324.0922; found 324.0891.

2-(1-((2-chlorothiazol-5-yl)methyl)aziridin-2-yl)-6-methyl-1,3,6,2-dioxazaborocane-4,8-dione (29):

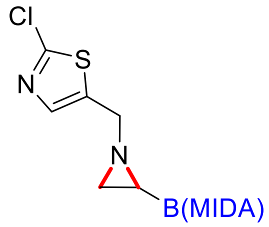

General procedure B with **3c** (210 mg, 0.3 mmol), (2-chlorothiazol-5-yl)methanamine (54 mg, 0.36 mmol, 1.2 equiv) and Cs_2CO_3 (295 mg, 0.9 mmol, 3.0 equiv) to afford a white solid (77 mg, 78% yield). Rf = 0.4 (Dichloromethane:MeOH=95:5).

^{1}H NMR (400 MHz, DMSO) δ 7.58 (s, 1H), 4.32 (d, *J* = 17.2 Hz, 1H), 4.21 (d, *J* = 16.8 Hz, 1H), 4.14 – 3.88 (m, 4H), 3.21 – 3.14 (m, 1H), 2.99 (s, 3H), 1.57 (dd, *J* = 4.4, 1.4 Hz, 1H), 1.45 (dd, *J* = 7.5, 1.5 Hz, 1H), 0.90 (dd, *J* = 7.6, 4.4 Hz, 1H). ^{13}C NMR (101 MHz, DMSO) δ 169.87, 168.93, 149.80, 141.33, 139.07, 62.34, 62.14, 57.25, 46.59, 30.98. HRMS- ESI (m/z) [M+H]+ calc'd for $C_{11}H_{14}BClN_3O_4S$, 330.0487, found 330.0482.

6-methyl-2-((*R*)-1-((*R*)-1-(4-(trifluoromethyl)phenyl)ethyl)aziridin-2-yl)-1,3,6,2-dioxazaborocane-4,8-dione (30):

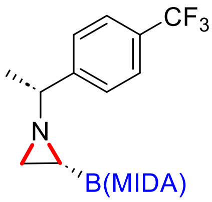

General procedure B with **3c** (210 mg, 0.3 mmol), (*R*)-1-(4-(trifluoromethyl)phenyl)ethan-1-amine HCl salt (81.3 mg, 0.36 mmol, 1.2 equiv.,) and Cs_2CO_3 (490 mg, 1.5 mmol, 5.0 equiv) to afford a white solid (68 mg, 61% yield). Rf = 0.45 (Dichloromethane:MeOH=95:5).

^{1}H NMR (400 MHz, DMSO) δ 8.26 (d, *J* = 8.2 Hz, 2H), 8.16 (d, *J* = 8.0 Hz, 2H), 4.52 (d, *J* = 33.9 Hz, 1H), 4.35 (s, 1H), 4.20 (d, *J* = 17.1 Hz, 1H), 3.91 (d, *J* = 16.5 Hz, 1H), 3.03 (q, *J* = 6.5 Hz, 1H), 2.70 (s, 3H), 2.29 (dd, *J* = 4.3, 1.3 Hz, 1H), 2.06 – 2.02 (m, 1H), 2.00 (d, *J* = 6.5 Hz, 3H), 1.29 (dd, *J* = 7.7, 4.2 Hz, 1H).^{11}B NMR (128 MHz, DMSO) δ 10.82. ^{13}C NMR (101 MHz, CD_3CN) δ 167.80, 166.84, 166.56, 149.40, 128.36, 128.04, 127.37, 126.31, 124.95, 124.67, 122.26, 70.29, 60.71, 60.61, 60.47, 46.95, 43.42, 28.83, 21.52. HRMS- ESI (m/z) [M+H]+ calc'd for $C_{16}H_{19}BF_3N_2O_4$, 371.1390, found 371.1383.

2-((*R*)-1-((*R*)-1-(4-chlorophenyl)ethyl)aziridin-2-yl)-6-methyl-1,3,6,2-dioxazaborocane-4,8-dione (31):

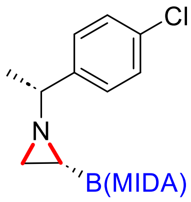

General procedure B with **3c** (210 mg, 0.3 mmol), (*R*)-1-(4-chlorophenyl)ethan-1-amine HCl salt (56 mg, 0.36 mmol, 1.2 equiv.,) and Cs_2CO_3 (490 mg, 1.5 mmol, 5.0 equiv) to afford a white solid (61 mg, 61% yield). Rf = 0.3 (Dichloromethane:MeOH=9:1).

^{1}H NMR (400 MHz, DMSO) δ 7.32 (s, 4H), 4.08 (d, *J* = 17.3 Hz, 1H), 3.97 – 3.87 (m, 1H), 3.73 (d, *J* = 17.2 Hz, 1H), 3.29 (d, *J* = 16.5 Hz, 2H), 2.27 (q, *J* = 6.5 Hz, 1H), 2.09 (s, 3H), 1.51 (dd, *J* = 4.2, 1.5 Hz, 1H), 1.31 (dd, *J* = 7.6, 1.5 Hz, 1H), 1.25 (d, *J* = 6.5 Hz, 3H), 0.59 (dd, *J* = 7.6, 4.2 Hz, 1H). ^{11}B NMR (128 MHz, DMSO) δ 5.20. ^{13}C NMR (101 MHz, DMSO) δ 169.84, 168.48, 144.79, 132.12, 129.61, 128.85, 70.60, 61.90, 61.84, 61.66, 44.86, 30.10, 23.67. HRMS- ESI (m/z) [M+H]+ calc'd for $C_{15}H_{19}BClN_2O_4$, 337.1120, found 337.1116.

6-methyl-2-(1-(prop-2-yn-1-yl)aziridin-2-yl)-1,3,6,2-dioxazaborocane-4,8-dione (32):

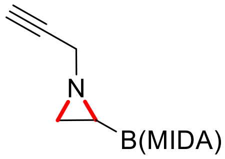

General procedure B with **3c** (210 mg, 0.3 mmol), prop-2-yn-1-amine (20 mg, 0.36 mmol, 1.2 equiv) and Cs_2CO_3 (295 mg, 0.9 mmol, 3.0 equiv) to afford a white solid (41 mg, 58% yield). Rf = 0.4 (Dichloromethane:MeOH=9:1).

^{1}H NMR (400 MHz, DMSO) δ 4.27 (d, *J* = 17.3 Hz, 1H), 4.14 (d, *J* = 16.7 Hz, 1H), 4.00 (d, *J* = 17.3 Hz, 1H), 3.85 (d, *J* = 16.6 Hz, 1H), 3.24 (dd, *J* = 16.5, 2.5 Hz, 1H), 3.17 (t, *J* = 2.5 Hz, 1H), 3.08 (d, *J* = 2.5 Hz, 1H), 3.03 (s, 3H), 1.44 (dd, *J* = 4.3, 1.5 Hz, 1H), 1.34 (dd, *J* = 7.6, 1.5 Hz, 1H), 0.73 (dd, *J* = 7.6, 4.3 Hz, 1H).) δ ^{13}C NMR (101 MHz, DMSO) δ 169.55, 168.32, 80.85, 75.09, 61.83, 61.59, 54.91, 48.11, 46.23, 28.88. ^{11}B NMR (128 MHz, DMSO) δ 8.49. HRMS- ESI (m/z) [M+H]+ calc'd for $C_{10}H_{14}O_4N_2B$ 237.1041; found 237.1037.

2-(1-(2-(1*H*-indol-3-yl)ethyl)aziridin-2-yl)-6-methyl-1,3,6,2-dioxazaborocane-4,8-dione (33):

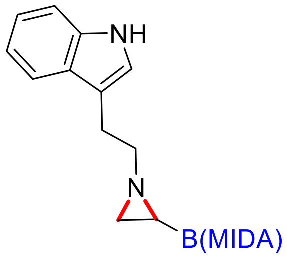

General procedure B with **3c** (210 mg, 0.3 mmol), Tryptamine (58 mg, 0.36 mmol, 1.2 equiv.,) and Cs_2CO_3 (295 mg, 0.9 mmol, 3.0 equiv) to afford a white solid (56 mg, 54% yield). Rf = 0.2 (Dichloromethane:MeOH=85:15).

^{1}H NMR (400 MHz, CD_3CN) δ 9.29 (s, 1H), 7.52 (d, J = 8.0 Hz, 1H), 7.35 (d, J = 8.1 Hz, 1H), 7.15 – 7.06 (m, 2H), 7.00 (ddd, J = 8.0, 6.9, 1.1 Hz, 1H), 4.12 – 3.91 (m, 4H), 3.69 (s, 1H), 3.45 – 3.36 (m, 1H), 3.16 – 3.12 (m, 2H), 3.00 (s, 3H), 2.69 – 2.62 (m, 2H), 2.32 (dd, J = 9.2, 8.2 Hz, 1H). ^{11}B NMR (128 MHz, CD_3CN) δ 8.94. ^{13}C NMR (101 MHz, CD_3CN) δ 168.13, 167.76, 137.18, 127.43, 124.28, 122.39, 119.72, 118.77, 112.18, 109.76, 63.81, 63.58, 55.82, 47.80, 33.39, 23.12. HRMS- ESI (m/z) [M+H]+ calc'd for $C_{17}H_{21}BN_3O_4$ 342.1625; found 342.1619.

2-(1-hexylaziridin-2-yl)-6-methyl-1,3,6,2-dioxazaborocane-4,8-dione (34):

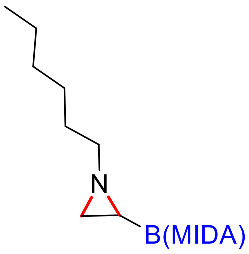

General procedure B with **3c** (210 mg, 0.3 mmol), hexylamine (36 mg, 0.36 mmol, 1.2 equiv) and Cs_2CO_3 (295 mg, 0.9 mmol, 3.0 equiv) to afford a white solid (50 mg, 59% yield). Rf = 0.2 (Dichloromethane:MeOH=85:15).

^{1}H NMR (400 MHz, $CDCl_3$) δ 3.93 (d, J = 17.7 Hz, 2H), 3.86 – 3.75 (m, 2H), 3.03 (s, 3H), 2.61 (dt, J = 11.2, 7.3 Hz, 1H), 1.68 (d, J = 4.0 Hz, 1H), 1.61 (dd, J = 12.8, 5.7 Hz, 1H), 1.46 (t, J = 5.0 Hz, 1H), 1.25 – 1.16 (m, 8H), 0.81 (t, J = 6.6 Hz, 3H), 0.49 (dd, J = 7.4, 4.1 Hz, 1H). ^{13}C NMR (126 MHz, $CDCl_3$) δ 168.72, 167.54, 62.34, 61.99, 46.08, 32.12, 31.79, 30.07, 29.69, 27.12, 22.61, 14.06. HRMS- ESI (m/z) [M+H]+ calc'd for $C_{13}H_{24}BN_2O_4$ 283.1829; found 283.1823.

Methyl 3-(2-(6-methyl-4,8-dioxo-1,3,6,2-dioxazaborocan-2-yl)aziridin-1-yl)bicyclo[1.1.1]pentane-1-carboxylate (35):

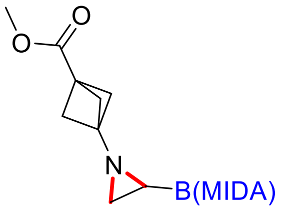

general procedure B with **3c** (210 mg, 0.3 mmol), Methyl 3-aminobicyclo[1.1.1]pentane-1-carboxylate hydrochloride (64 mg, 0.36 mmol, 1.2 equiv) and Cs_2CO_3 (295 mg, 0.9 mmol, 3.0 equiv) to afford a white solid (68 mg, 71% yield). Rf = 0.3 (Dichloromethane:MeOH=95:5).

^{1}H NMR (400 MHz, DMSO) δ 4.20 (d, J = 17.1 Hz, 1H), 4.10 (d, J = 16.9 Hz, 1H), 3.98 (d, J = 17.2 Hz, 1H), 3.77 (d, J = 16.8 Hz, 1H), 3.52 (s, 3H), 2.91 (s, 3H), 1.81 (s, 6H), 1.35 – 1.31 (m, 2H), 0.84 – 0.63 (m, 1H). ^{11}B NMR (128 MHz, DMSO) δ 13.41. ^{13}C NMR (101 MHz, DMSO) δ 170.59, 169.82, 168.94, 62.59, 62.19, 58.44, 51.97, 50.82, 46.84, 33.18, 27.30. HRMS- ESI (m/z) [M]+ calc'd for $C_{14}H_{20}BN_2O_6$, 323.1414, found 323.1408.

***Tert*-butyl (*S*)-4-methyl-2-((*S*)-2-(6-methyl-4,8-dioxo-1,3,6,2-dioxazaborocan-2-yl)aziridin-1-yl)pentanoate (36):**

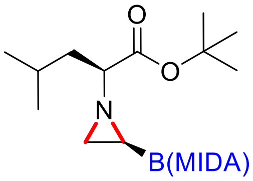

General procedure B with **3c** (210 mg, 0.3 mmol), *tert*-butyl *L*-leucinate (67.6 mg, 0.36 mmol, 1.2 equiv) and Cs_2CO_3 (295 mg, 0.9 mmol, 3.0 equiv) to afford a white solid (79 mg, 72% yield). Rf = 0.3 (Dichloromethane:MeOH=9:1).

^{1}H NMR (400 MHz, DMSO) δ 4.30 – 4.23 (m, 1H), 4.13 (d, J = 16.2 Hz, 1H), 3.97 (dd, J = 20.7, 16.8 Hz, 2H), 2.92 (s, 3H), 1.98 (dd, J = 8.3, 4.8 Hz, 1H), 1.62 (ddt, J = 18.9, 13.2, 6.0 Hz, 2H), 1.48 (dd, J = 4.3, 1.4 Hz, 1H), 1.41 (s, 10H), 1.25 (dd, J = 7.6, 1.5 Hz, 1H), 0.87 (dd, J = 8.1, 6.3 Hz, 6H), 0.74 (dd, J = 7.6, 4.2 Hz, 1H). ^{11}B NMR (128 MHz, DMSO) δ 10.15. ^{13}C NMR (101 MHz, DMSO) δ 173.44, 170.06, 168.65, 81.03, 72.91, 61.85, 61.48, 45.42, 42.90, 28.84, 28.11, 25.12, 23.81, 22.60. HRMS- ESI (m/z) [M+H]+ calc'd for $C_{17}H_{30}BN_2O_6$, 369.2197, found 369.2192.

Methyl (*S*)-3-cyclohexyl-2-((*S*)-2-(6-methyl-4,8-dioxo-1,3,6,2-dioxazaborocan-2-yl)aziridin-1-yl)propanoate (37):

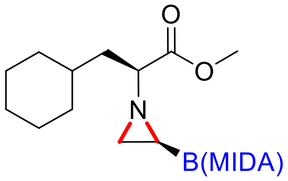

General procedure B with **3c** (210 mg, 0.3 mmol), L-CHA-OMe-HCl salt (80 mg, 0.36 mmol, 1.2 equiv) and Cs_2CO_3 (490 mg, 1.5 mmol, 5.0 equiv) to afford a white solid (66 mg, 60% yield). Rf = 0.6 (Dichloromethane:MeOH=9:1).

^{1}H NMR (400 MHz, DMSO) δ 4.14 (dd, *J* = 17.0, 4.9 Hz, 2H), 3.92 (dd, *J* = 17.0, 7.3 Hz, 2H), 3.57 (d, *J* = 2.7 Hz, 3H), 2.89 (s, 3H), 2.19 (dd, *J* = 8.9, 4.8 Hz, 1H), 1.60 – 1.51 (m, 6H), 1.41 (dt, *J* = 10.2, 5.0 Hz, 1H), 1.36 (dd, *J* = 7.5, 4.6 Hz, 1H), 1.28 (d, *J* = 7.7 Hz, 2H), 1.07 (td, *J* = 11.9, 5.2 Hz, 3H), 0.82 – 0.71 (m, 2H), 0.58 (dd, *J* = 7.6, 4.5 Hz, 1H). ^{11}B NMR (128 MHz, DMSO) δ 14.71. ^{13}C NMR (101 MHz, DMSO) δ 173.24, 169.58, 169.28, 71.20, 62.32, 51.89, 51.84, 46.46, 34.28, 33.76, 32.97, 29.25, 26.47, 26.13, 26.05. HRMS-ESI (m/z) [M+H]+ calc'd for $C_{17}H_{28}BN_2O_6$, 367.2040, found 367.2033.

Methyl (*S*)-2-((*S*)-2-(6-methyl-4,8-dioxo-1,3,6,2-dioxazaborocan-2-yl)aziridin-1-yl)-3-((*S*)-2-oxopyrrolidin-3-yl)propanoate (38):

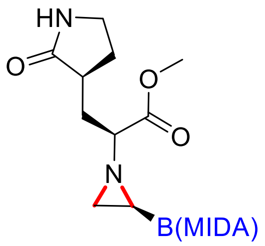

General procedure B with **3c** (210 mg, 0.3 mmol), Methyl (*S*)-2-amino-3-((*S*)-2-oxopyrrolidin-3-yl)propanoate HCl salt (67 mg, 0.36 mmol, 1.2 equiv.,) and Cs_2CO_3 (490 mg, 1.5 mmol, 5.0 equiv) to afford a white solid (76 mg, 69% yield). Rf = 0.4 (Dichloromethane:MeOH=9:1).

^{1}H NMR (400 MHz, DMSO) δ 7.52 (s, 1H), 4.19 (d, *J* = 17.2 Hz, 1H), 4.05 (d, *J* = 16.3 Hz, 1H), 3.90 (dd, *J* = 16.7, 11.8 Hz, 2H), 3.57 (s, 3H), 3.03 (dd, *J* = 11.3, 7.8 Hz, 2H), 2.82 (s, 3H), 2.31 (dd, *J* = 6.9, 4.5 Hz, 1H), 2.08 (ddt, *J* = 17.8, 8.8, 4.6 Hz, 3H), 1.59 – 1.48 (m, 2H), 1.44 (dd, *J* = 4.3, 1.4 Hz, 1H), 1.28 (dd, *J* = 7.6, 1.5 Hz, 1H), 0.72 (dd, *J* = 7.6, 4.3 Hz, 1H). ^{11}B NMR (128 MHz, DMSO) δ 8.19. ^{13}C NMR (101 MHz, DMSO) δ 178.52, 174.14, 170.10, 168.66, 71.85, 61.89, 61.42, 55.36, 52.14, 45.22, 38.29, 35.08, 28.98, 28.89. HRMS- ESI (m/z) [M]+ calc'd for $C_{15}H_{23}BN_3O_7$, 368.1629, found 368.1632.

Methyl (*S*)-3-(4-chlorophenyl)-3-((*S*)-2-(6-methyl-4,8-dioxo-1,3,6,2-dioxazaborocan-2-yl)aziridin-1-yl)propanoate (39):

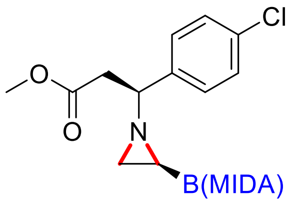

General procedure B with **3c** (210 mg, 0.3 mmol), methyl (*S*)-3-amino-3-(4-chlorophenyl)propanoate HCl salt (77 mg, 0.36 mmol, 1.2 equiv) and Cs_2CO_3 (490 mg, 1.5 mmol, 5.0 equiv) to afford a white solid (80 mg, 68% yield). Rf = 0.3 (Dichloromethane:MeOH=9:1).

^{1}H NMR (400 MHz, DMSO) δ 7.31 (d, *J* = 6.0 Hz, 4H), 4.16 (dd, *J* = 17.0, 5.1 Hz, 2H), 3.96 (dd, *J* = 17.0, 11.6 Hz, 2H), 3.41 (s, 3H), 2.95 (d, *J* = 9.1 Hz, 3H), 2.82 (dd, *J* = 13.0, 3.3 Hz, 1H), 2.72 – 2.63 (m, 2H), 1.24 (dd, *J* = 4.4, 1.4 Hz, 1H), 1.14 (dd, *J* = 7.5, 1.4 Hz, 1H), 0.82 (dd, *J* = 7.5, 4.4 Hz, 1H). ^{11}B NMR (128 MHz, DMSO) δ 15.65. ^{13}C NMR (101 MHz, DMSO) δ 171.36, 169.63, 169.33, 141.60, 132.24, 129.94, 128.52, 71.15, 62.37, 62.33, 51.71, 46.50, 42.07, 29.62. HRMS- ESI (m/z) [M+H]+ calc'd for $C_{17}H_{21}BClN_2O_6$, 395.1181, found 395.1168.

2-(1-((7-chloro-1,1-dioxido-3,4-dihydro-2*H*-benzo[*e*][1,2,4]thiadiazin-6-yl)sulfonyl)aziridin-2-yl)-6-methyl-1,3,6,2-dioxazaborocane-4,8-dione (40):

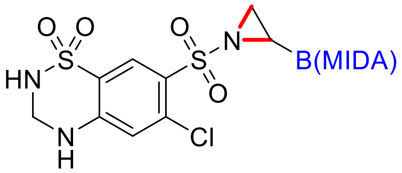

General procedure A with **3c** (210 mg, 0.3 mmol), Hydrochlorothiazide (107.3 mg, 0.36 mmol, 1.2 equiv.,) and K_2CO_3 (207 mg, 1.5 mmol, 5.0 equiv) to afford a white solid (86 mg, 60% yield). Rf = 0.3 (hexanes:ethyl acetate=2:8).^{1}H NMR (400 MHz, DMSO) δ 8.39 (t, *J* = 2.7 Hz, 1H), 8.07 (t, *J* = 7.9 Hz, 1H), 8.00 (s, 1H), 7.10 (s, 1H), 4.81 (dd, *J* = 7.9, 2.7 Hz, 2H), 4.42 (d, *J* = 17.2 Hz, 1H), 4.31 (d, *J* = 16.8 Hz, 1H), 4.15 (d, *J* = 17.2 Hz, 1H), 3.89 (d, *J* = 16.8 Hz, 1H), 3.16 (s, 3H), 2.52 (d, *J* = 8.3 Hz, 1H), 2.39 (dd, *J* = 8.3, 5.4 Hz, 1H), 2.13 (d, *J* = 5.4 Hz, 1H).^{11}B NMR (128 MHz, DMSO) δ 8.58. 13C NMR (101 MHz, DMSO) δ 172.16, 169.58, 168.36, 151.31, 147.42, 139.36, 139.03, 129.08, 122.86, 62.30, 62.27, 46.77, 27.68. HRMS- ESI (m/z) [M+H]+ calc'd for $C_{14}H_{17}BClN_4O_8S_2$+, 479.0269, found 479.0249.

***N*-methyl-*N*-(4-methyl-5-((2-(6-methyl-4,8-dioxo-1,3,6,2-dioxazaborocan-2-yl)aziridin-1-yl)sulfonyl)thiazol-2-yl)-2-(4-(pyridin-2-yl)phenyl)acetamide (41)**:

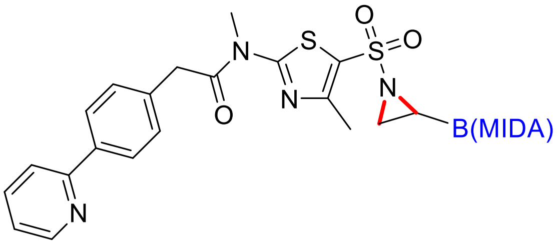

General procedure A with **3c** (210 mg, 0.3 mmol), Pritelivir (145 mg, 0.36 mmol, 1.2 equiv) and K_2CO_3 (207 mg, 1.5 mmol, 5.0 equiv) to afford a white solid (126 mg, 72% yield). Rf = 0.4 (100 % ethyl acetate).

^{1}H NMR (400 MHz, DMSO) δ 8.60 (dd, *J* = 4.9, 1.8 Hz, 1H), 8.00 (d, *J* = 8.0 Hz, 2H), 7.94 – 7.76 (m, 2H), 7.39 – 7.22 (m, 3H), 4.36 – 4.14 (m, 4H), 4.03 (d, *J* = 17.2 Hz, 1H), 3.82 (d, *J* = 17.0 Hz, 1H), 3.68 (s, 3H), 3.03 (s, 3H), 2.47 (s, 3H), 2.28 (d, *J* = 8.3 Hz, 1H), 2.12 (dd, *J* = 8.2, 5.4 Hz, 1H), 2.05 (d, *J* = 5.4 Hz, 1H). ^{13}C NMR (101 MHz, DMSO) δ 172.84, 169.49, 168.58, 161.49, 156.27, 153.91, 150.01, 137.87, 137.70, 135.32, 130.68, 126.99, 123.02, 120.61, 119.98, 62.46, 62.43, 46.66, 34.93, 30.60, 16.96. HRMS- ESI (m/z) [M+H]+ calc'd for $C_{25}H_{27}BN_5O_7S_2$+, 584.1439, found 584.1439

6-methyl-2-(1-((4-(5-methyl-3-phenylisoxazol-4-yl)phenyl)sulfonyl)aziridin-2-yl)-1,3,6,2-dioxazaborocane-4,8-dione (42):

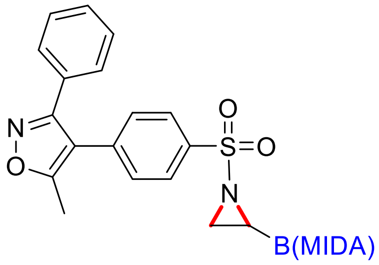

General procedure A with **3c** (210 mg, 0.3 mmol), Valdecoxib (113.5 mg, 0.36 mmol, 1.2 equiv.,) and K_2CO_3 (207 mg, 1.5 mmol, 5.0 equiv) to afford a white solid (113 mg, 76% yield). Rf = 0.5 (hexanes:ethyl acetate=4:6).

^{1}H NMR (400 MHz, DMSO) δ 7.93 – 7.80 (m, 2H), 7.48 – 7.34 (m, 5H), 7.29 (dq, *J* = 6.6, 2.3 Hz, 2H), 4.24 (dd, *J* = 27.7, 17.1 Hz, 2H), 4.04 (d, *J* = 17.1 Hz, 1H), 3.84 (d, *J* = 17.0 Hz, 1H), 2.98 (s, 3H), 2.44 (s, 3H), 2.38 (d, *J* = 8.4 Hz, 1H), 2.18 (dd, *J* = 8.3, 5.5 Hz, 1H), 2.02 (d, *J* = 5.5 Hz, 1H). ^{11}B NMR (128 MHz, DMSO) δ 8.72. ^{13}C NMR (101 MHz, DMSO) δ 167.29, 166.58, 166.35, 159.04, 134.53, 133.82, 128.74, 128.20, 127.26, 126.63, 126.60, 126.57, 112.31, 60.37, 60.33, 44.52, 28.60, 9.81. HRMS- ESI (m/z) [M+H]+ calc'd for $C_{23}H_{22}BN_3O_7S$+, 496.1350, found 496.1353.

2-(1-((benzo[*d*]isoxazol-3-ylmethyl)sulfonyl)aziridin-2-yl)-6-methyl-1,3,6,2-dioxazaborocane-4,8-dione (43):

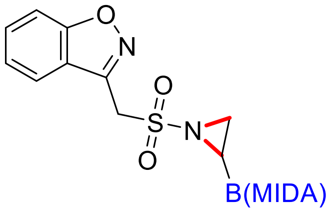

General procedure A with **3c** (210 mg, 0.3 mmol), Zonisamide (76.6 mg, 0.36 mmol, 1.2 equiv.,) and K_2CO_3 (207 mg, 1.5 mmol, 5.0 equiv) to afford a white solid (86 mg, 73% yield). Rf = 0.4 (hexanes:ethyl acetate=2:8).^{1}H NMR (400 MHz, DMSO) δ 8.01 (dt, *J* = 8.1, 1.1 Hz, 1H), 7.80 (t, *J* = 9.4 Hz, 1H), 7.74 – 7.67 (m, 1H), 7.44 (t, *J* = 7.5 Hz, 1H), 5.31 (d, *J* = 1.6 Hz, 2H), 4.39 – 4.20 (m, 2H), 4.09 (d, *J* = 17.1 Hz,

1H), 3.87 (d, J = 16.9 Hz, 1H), 3.06 (s, 3H), 2.38 (d, J = 8.4 Hz, 1H), 2.26 (dd, J = 8.3, 5.6 Hz, 1H), 2.14 – 2.03 (m, 1H). ^{11}B NMR (128 MHz, DMSO) δ 9.06. ^{13}C NMR (101 MHz, DMSO) δ 169.65, 169.43, 168.65, 163.28, 150.06, 131.23, 129.13, 124.60, 123.66, 121.23, 110.24, 62.45, 52.45, 48.41, 47.17, 46.67, 32.08. HRMS- ESI (m/z) [M+H]+ calc'd for $C_{15}H_{17}BFN_3O_7S^+$, 394.0850, found 394.0858.

3-chloro-4-((2-(6-methyl-4,8-dioxo-1,3,6,2-dioxazaborocan-2-yl)aziridin-1-yl)sulfonyl)-*N*-(2-methylindolin-1-yl)benzamide (44):

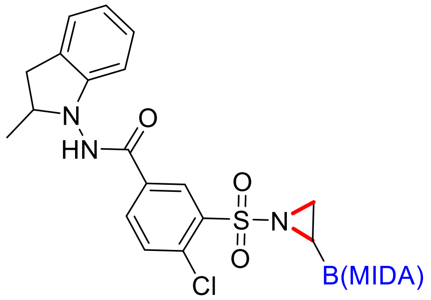

General procedure A with **3c** (210 mg, 0.3 mmol), Indapamide (132 mg, 0.36 mmol, 1.2 equiv.,) and K_2CO_3 (207 mg, 1.5 mmol, 5.0 equiv) to afford a white solid (125 mg, 76% yield). Rf = 0.4 (hexanes:ethyl acetate=1:9).

^{1}H NMR (400 MHz, DMSO) δ 10.52 (s, 1H), 8.43 (dd, J = 4.0, 2.2 Hz, 1H), 8.16 (dt, J = 8.3, 2.0 Hz, 1H), 7.85 (d, J = 8.3 Hz, 1H), 7.13 – 6.89 (m, 2H), 6.80 – 6.62 (m, 1H), 6.48 (d, J = 7.8 Hz, 1H), 4.27 (dd, J = 38.0, 17.0 Hz, 2H), 4.05 (d, J = 17.2 Hz, 1H), 3.85 (t, J = 16.8 Hz, 2H), 3.16 – 2.94 (m, 5H), 2.54 (dd, J = 9.7, 6.3 Hz, 2H), 2.43 – 2.37 (m, 1H), 2.09 (dd, J = 5.6, 2.0 Hz, 1H), 1.24 (d, J = 6.1 Hz, 3H). ^{11}B NMR (128 MHz, DMSO) δ 9.28. ^{13}C NMR (101 MHz, DMSO) δ 167.41, 166.34, 162.01, 161.95, 149.76, 134.04, 133.64, 132.24, 131.08, 130.84, 130.80, 128.28, 125.43, 122.77, 118.42, 116.45, 60.25, 60.19, 44.47, 33.79, 30.26, 16.89. HRMS- ESI (m/z) [M+H]+ calc'd for $C_{23}H_{25}BClN_4O_7S^+$, 547.1226, found 547.1234.

***N*-((1-ethylpyrrolidin-2-yl)methyl)-2-methoxy-5-((2-(6-methyl-4,8-dioxo-1,3,6,2-dioxazaborocan-2-yl)aziridin-1-yl)sulfonyl)benzamide (45):**

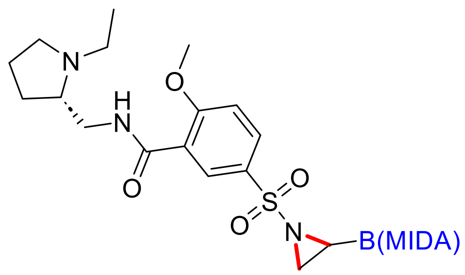

General procedure A with **3c** (210 mg, 0.3 mmol), (*S*)-Sulpiride (123.3 mg, 0.36 mmol, 1.2 equiv.,) and K_2CO_3 (207 mg, 1.5 mmol, 5.0 equiv) to afford a white solid (134 mg, 71% yield). Rf = 0.3 (hexanes:ethyl acetate=0:100).

^{1}H NMR (400 MHz, DMSO) δ 8.04 (dd, *J* = 6.0, 3.5 Hz, 1H), 7.95 – 7.90 (m, 1H), 7.69 – 7.59 (m, 2H), 4.32 (dd, *J* = 45.9, 17.1 Hz, 2H), 4.10 (d, *J* = 17.2 Hz, 1H), 3.90 (d, *J* = 16.9 Hz, 1H), 3.11 (s, 3H), 2.52 (s, 1H), 2.39 (dd, *J* = 8.3, 5.5 Hz, 1H), 2.10 (d, *J* = 5.4 Hz, 1H). ^{13}C NMR (101 MHz, DMSO) δ 169.09, 167.93, 137.10, 135.84, 135.38, 131.19, 128.43, 120.56, 61.98, 61.87, 46.12, 31.56. ^{11}B NMR (128 MHz, DMSO-d6). HRMS- ESI (m/z) [M+H]+ calc'd for $C_{22}H_{32}BN_4O_8S$ 523.2034; found 523.2007.

((3a*S*,5a*R*,8a*R*,8b*S*)-2,2,7,7-tetramethyltetrahydro-3a*H*-bis([1,3]dioxolo)[4,5-*b*:4',5'-*d*]pyran-3a-yl)methyl (*R*)-2-(6-methyl-4,8-dioxo-1,3,6,2-dioxazaborocan-2-yl)aziridine-1-sulfonate (46):

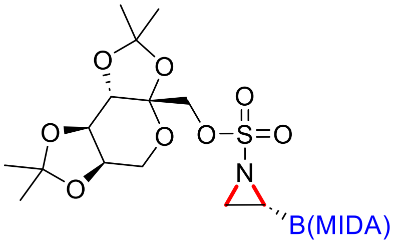

General procedure A with **3c** (210 mg, 0.3 mmol), Topiramate (102.1 mg, 0.36 mmol, 1.2 equiv.,) and K_2CO_3 (207 mg, 1.5 mmol, 5.0 equiv) to afford a white solid (103 mg, 66% yield). Rf = 0.4 (hexanes:ethyl acetate=3:7).

^{1}H NMR (400 MHz, DMSO) δ 4.66 (dt, *J* = 7.9, 2.4 Hz, 1H), 4.45 – 4.27 (m, 6H), 4.18 (dd, *J* = 17.1, 1.6 Hz, 1H), 3.99 (dd, *J* = 17.0, 8.5 Hz, 1H), 3.81 (dt, *J* = 13.1, 2.1 Hz, 1H), 3.70 (dd, *J* = 12.9, 2.7 Hz, 1H), 3.13 (s, 3H), 2.61 – 2.56 (m, 1H), 2.33 – 2.25 (m, 2H), 1.53 (s, 3H), 1.41 (dd, *J* = 11.0, 2.4 Hz, 6H), 1.34 (s, 3H). ^{11}B NMR (128 MHz, DMSO) δ 8.96. ^{13}C NMR (101 MHz, DMSO) δ 169.36, 168.71, 168.66, 109.06, 108.71, 100.52, 72.71, 72.46, 70.43, 70.25, 69.53, 62.58, 61.04, 46.81, 31.35, 26.63, 26.10, 25.29, 24.38. HRMS-ESI (m/z) [M+H]+ calc'd for $C_{19}H_{30}BN_2O_{12}S$+, 521.1613, found 521.1608.

7-(but-2-yn-1-yl)-3-methyl-8-((*R*)-3-((*R*)-2-(6-methyl-4-oxo-1,3,6,2-dioxazaborocan-2-yl)aziridin-1-yl)piperidin-1-yl)-1-((4-methylquinazolin-2-yl)methyl)-3,7-dihydro-1*H*-purine-2,6-dione (47):

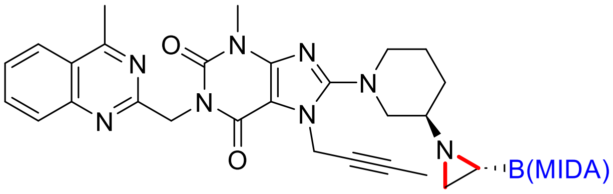

***General procedure* B** with **3c** (210 mg, 0.3 mmol), Linagliptin (170.7 mg, 0.36 mmol, 1.2 equiv.,) and Cs_2CO_3 (294.4 mg, 0.9 mmol, 3.0 equiv) to afford a white solid (127 mg, 66% yield). Rf = 0.4 (Dichloromethane:Methanol=9:1).

^{1}H NMR (400 MHz, DMSO) δ 8.19 – 8.16 (m, 1H), 7.87 – 7.82 (m, 1H), 7.76 – 7.72 (m, 1H), 7.62 – 7.58 (m, 1H), 4.87 – 4.77 (m, 2H), 4.19 – 4.10 (m, 2H), 4.01 – 3.79 (m, 3H), 3.73 – 3.51 (m, 3H), 3.39 – 3.33 (m, 6H), 2.81 (d, *J* = 1.5 Hz, 4H), 1.90 (d, *J* = 10.4 Hz, 1H), 1.75 – 1.68 (m, 5H), 1.59 – 1.48 (m, 1H), 1.40 (ddd,

J = 11.2, 6.8, 3.8 Hz, 3H), 1.26 (t, J = 7.0 Hz, 1H), 0.67 (dt, J = 7.8, 4.0 Hz, 1H). ^{13}C NMR (101 MHz, DMSO) δ 169.65, 169.62, 169.32, 169.19, 161.47, 161.44, 156.32, 156.24, 153.72, 153.66, 151.40, 149.51, 148.12, 148.01, 134.57, 128.34, 127.63, 126.22, 122.97, 103.85, 103.70, 81.72, 74.55, 74.05, 62.41, 46.03, 29.90, 29.84, 22.05, 3.58. HRMS- ESI (m/z) [M+H]+ calc'd for $C_{32}H_{37}BN_9O_6$ 654.2960; found 654.2980.

6-methyl-2-((*R*)-1-((1*R*,2*S*)-2-phenylcyclopropyl)aziridin-2-yl)-1,3,6,2-dioxazaborocane-4,8-dione (1:1 isomers) (48):

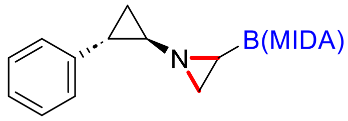

***General procedure* B** with **3c** (210 mg, 0.3 mmol), Tranylcypromine HCl (61.1 mg, 0.36 mmol, 1.2 equiv.,) and Cs_2CO_3 (490 mg, 1.5 mmol, 5.0 equiv) to afford a white solid (56 mg, 60% yield). Rf = 0.25 (Dichloromethane:Methanol=9:1).

^1H NMR (400 MHz, DMSO) δ 7.19 (dd, J = 8.1, 6.9 Hz, 2H), 7.12 – 7.06 (m, 1H), 7.02 – 6.97 (m, 2H), 4.25 (dd, J = 17.3, 10.2 Hz, 1H), 4.18 – 3.80 (m, 3H), 3.04 (d, J = 12.3 Hz, 3H), 2.09 (dddd, J = 58.9, 9.4, 5.9, 3.3 Hz, 1H), 1.59 – 1.37 (m, 3H), 1.30 – 1.07 (m, 1H), 0.96 – 0.76 (m, 2H). ^{11}B NMR (128 MHz, DMSO) δ 9.48. ^{13}C NMR (101 MHz, DMSO) δ 170.06, 170.00, 168.94, 168.83, 142.56, 142.51, 128.68, 128.62, 126.06, 125.95, 125.80, 125.74, 62.55, 62.42, 62.13, 62.07, 53.12, 52.94, 46.61, 46.55, 30.21, 29.73, 24.08, 22.70, 16.93, 15.53. HRMS- ESI (m/z) [M+H]+ calc'd for $C_{16}H_{20}BN_2O_4$ 315.1509; found 315.1509.

6-methyl-2-((*R*)-1-((1*R*,2*R*)-2-phenylcyclopropyl)aziridin-2-yl)-1,3,6,2-dioxazaborocane-4,8-dione (49):

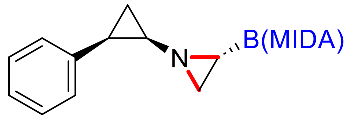

***General procedure* B** with **3c** (210 mg, 0.3 mmol), *Cis*-Tranylcypromine HCl (61.1 mg, 0.36 mmol, 1.2 equiv.,) and Cs_2CO_3 (490 mg, 1.5 mmol, 5.0 equiv) to afford a white solid (58 mg, 62% yield). Rf= 0.25 (Dichloromethane:Methanol=9:1).

^1H NMR (400 MHz, DMSO) δ 7.25 – 7.19 (m, 2H), 7.10 (dd, J = 8.2, 6.5 Hz, 2H), 7.07 – 7.00 (m, 1H), 4.21 (dd, J = 17.2, 9.2 Hz, 2H), 4.04 – 3.98 (m, 2H), 3.83 (d, J = 16.7 Hz, 1H), 3.02 (s, 3H), 1.82 (dt, J = 9.3, 7.1 Hz, 1H), 1.47 (td, J = 7.2, 4.5 Hz, 1H), 1.22 – 1.13 (m, 2H), 1.07 – 0.98 (m, 2H), 0.79 (dd, J = 7.4, 4.3 Hz, 1H). ^{11}B NMR (128 MHz, DMSO) δ 5.90.^{13}C NMR (101 MHz, DMSO) δ 170.19, 168.53, 139.81, 128.89, 127.79, 125.49, 62.37, 61.85, 49.76, 48.26, 46.52, 30.32, 22.15, 14.51. HRMS- ESI (m/z) [M+H]+ calc'd for $C_{16}H_{20}BN_2O_4$ 315.1509; found 315.1509.

***N*-(2-(6-methyl-4,8-dioxo-1,3,6,2-dioxazaborocan-2-yl)aziridin-1-yl)isonicotinamide (50):**

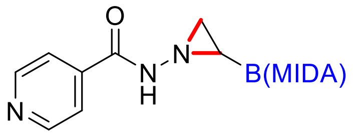

***General procedure* B** with **3c** (210 mg, 0.3 mmol), Isoniazid (49.5 mg, 0.36 mmol, 1.2 equiv.,) and Cs_2CO_3 (294 mg, 0.9 mmol, 3.0 equiv) to afford a white solid (38 mg, 40% yield). Rf = 0.1 (Dichloromethane:Methanol=9:1).

^{1}H NMR (400 MHz, DMSO) δ 10.19 (s, 1H), 8.69 (d, *J* = 5.0 Hz, 2H), 7.78 – 7.52 (m, 2H), 4.38 (dd, *J* = 16.7, 11.9 Hz, 2H), 4.15 (d, *J* = 16.0 Hz, 1H), 3.97 (d, *J* = 17.3 Hz, 1H), 3.17 (s, 3H), 2.32 (d, *J* = 8.9 Hz, 1H), 1.89 (d, *J* = 6.0 Hz, 1H), 1.30 (dd, *J* = 9.0, 6.0 Hz, 1H). ^{11}B NMR (128 MHz, DMSO) δ 12.87. ^{13}C NMR (101 MHz, DMSO) δ 170.37, 168.60, 163.87, 150.59, 140.93, 121.62, 61.76, 61.52, 49.07, 45.55, 32.22. HRMS- ESI (m/z) [M+H]+ calc'd for $C_{13}H_{16}BN_4O_5$ 319.1214; found 319.1212.

2-(1-((4-(5-(*p*-tolyl)-3-(trifluoromethyl)-1*H*-pyrazol-1-yl)phenyl)sulfonyl)aziridin-2-yl)-6-((1*R*,2*R*,3*R*,5*S*)-2,6,6-trimethylbicyclo[3.1.1]heptan-3-yl)-1,3,6,2-dioxazaborocane-4,8-dione (52):

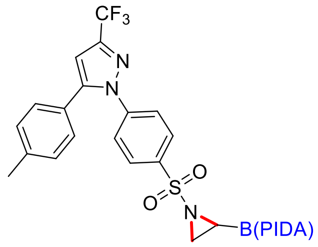

To an oven dried H-cell (divided cell) containing magnetic stir bars was charged with vinyl B(PIDA) [1] **1d** (91 mg, 0.3 mmol, 1 equiv), TT (97 mg, 0.45 mmol, 1.5 equiv) in 4 mL anhydrous acetonitrile (0.2 M n-Bu_4NPF_6) to the anode compartment, and trifluoroacitic acid (0.8 mmol) in 4 mL anhydrous acetonitrile (0.2 M n-Bu_4NPF_6) to the cathode compartment under argon atmosphere. Using stainless steel wire/RVC (12 mm × 5 mm × 3 mm) cathode and pencil/RVC (10 mm × 5 mm × 3 mm) anode, the reaction mixture was electrolyzed under constant current (12 mA) for 5 h. At the completion of the electrolysis, the electrode on the anode side was removed and Cs_2CO_3 (1 mmol), Celecoxib (0.2 mmol) was added to the anode compartment. The anodic compartment was equipped with septa with a needle to prevent pressurizing and after pressure equilibrium, the needle was removed, and cathode solution was removed from the cell using a pipette. The anodic solution was stirred in the cell for 16 h. The mixture was filtered, the filter cake was washed with dichloromethane (2 × 10 mL), and then the filtrates were combined and concentrated and purified directly by column chromatography to afford the product **52** (75 mg, 55%, dr > 20).

^{1}H NMR (400 MHz, DMSO) δ 7.94 (d, *J* = 8.7 Hz, 2H), 7.55 (d, *J* = 8.7 Hz, 2H), 7.16 (d, *J* = 7.6 Hz, 5H), 4.42 – 4.32 (m, 1H), 4.30 – 4.17 (m, 2H), 4.07 (d, *J* = 18.3 Hz, 1H), 3.97 (d, *J* = 15.6 Hz, 1H), 2.52 – 2.41 (m, 2H), 2.37 – 2.20 (m, 6H), 2.07 (d, *J* = 5.2 Hz, 1H), 1.90 (tq, *J* = 5.8, 2.4 Hz, 1H), 1.78 (td, *J* = 5.9, 2.2

Hz, 1H), 1.58 – 1.48 (m, 1H), 1.25 – 1.11 (m, 6H), 1.02 – 0.86 (m, 4H). ^{11}B NMR (128 MHz, DMSO) δ 9.13. ^{19}F NMR (377 MHz, DMSO) δ -60.96. ^{13}C NMR (101 MHz, DMSO) δ 170.67, 167.35, 145.92, 143.33, 143.16, 142.78, 139.70, 137.06, 129.98, 129.59, 129.30, 126.69, 125.71, 123.05, 120.38, 66.78, 60.89, 54.86, 49.15, 38.95, 37.79, 31.42, 30.20, 27.53, 23.21, 22.99, 21.30. HRMS- ESI (m/z) [M+H]+ calc'd for $C_{33}H_{37}BF_3N_4O_6S^+$, 685.2479, found 685.2464.

***N*-(2-((4-methoxyphenyl)amino)-1-(6-methyl-4,8-dioxo-1,3,6,2-dioxazaborocan-2-yl)ethyl)-4-(5-(*p*-tolyl)-3-(trifluoromethyl)-1*H*-pyrazol-1-yl)benzenesulfonamide (53):**

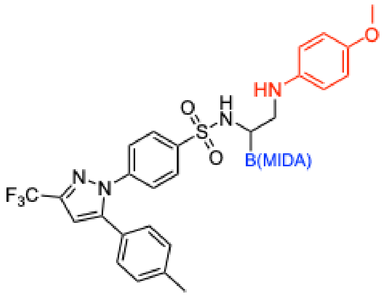

Aziridine **5** (100 mg, 0.17 mmol) and P-anisidine (65 mg, 0.51 mmol) were dissolved in anhydrous DMF (6 mL). The resulting solution was stirred at 60 °C for 14 h, and then concentrated in vacuo at 60 °C. The crude residue was purified by flash column chromatography (Rf = 0.4 in Hexane:ethyl acetate, 3:7) affording **53** (106 mg, 88%) as a green solid.

^{1}H NMR (400 MHz, DMSO) δ 7.72 – 7.65 (m, 2H), 7.34 – 7.30 (m, 2H), 7.21 (t, *J* = 5.5 Hz, 1H), 7.12 (d, *J* = 8.3 Hz, 3H), 7.07 – 7.04 (m, 2H), 6.54 (s, 4H), 4.46 (d, *J* = 11.0 Hz, 1H), 4.18 (dd, *J* = 28.2, 17.1 Hz, 2H), 4.03 – 3.81 (m, 2H), 3.44 (s, 3H), 3.06 (dt, *J* = 10.5, 4.7 Hz, 1H), 2.82 (s, 3H), 2.74 (t, *J* = 5.3 Hz, 2H), 2.24 (s, 3H). ^{19}F NMR (376 MHz, DMSO) δ -60.92. ^{13}C NMR (101 MHz, DMSO) δ 169.50, 169.35, 151.44, 145.65, 142.62, 141.72, 139.98, 139.60, 129.86, 129.15, 128.33, 126.22, 125.73, 123.10, 114.95, 114.86, 62.85, 62.73, 55.54, 46.37, 43.72, 21.26. HRMS- ESI (m/z) [M+H]+ calc'd for $C_{31}H_{32}BF_3N_5O_7S$, 686.2068, found 686.2103.

***N*-(2-(*tert*-butylamino)-1-(6-methyl-4,8-dioxo-1,3,6,2-dioxazaborocan-2-yl)ethyl)-4-(5-(*p*-tolyl)-3-(trifluoromethyl)-1*H*-pyrazol-1-yl)benzenesulfonamide (54):**

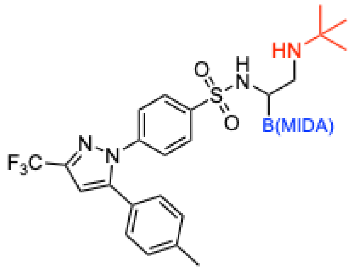

Tert-Butylamine (0.05 mL, 0.51 mmol) was added in one portion to a stirred, room-temperature solution of aziridine **5** (100 mg, 0.17 mmol) in acetonitrile (3 mL). After 1 h the reaction was complete (Rf = 0.3 in 75%

EtOAc/Hexane), and the volatiles were removed in vacuo. The crude residue was purified by flash column chromatography to afford **54** (96 mg, 85%) as a white solid.

^{1}H NMR (400 MHz, DMSO) δ 7.82 – 7.77 (m, 2H), 7.49 – 7.45 (m, 2H), 7.15 – 7.09 (m, 5H), 4.20 (d, *J* = 17.2 Hz, 1H), 4.05 (d, *J* = 16.5 Hz, 1H), 3.83 (dd, *J* = 37.9, 16.8 Hz, 2H), 2.90 (s, 3H), 2.85 (dd, *J* = 11.6, 2.9 Hz, 1H), 2.75 (dd, *J* = 11.7, 4.6 Hz, 1H), 2.41 (s, 1H), 2.24 (s, 3H), 0.79 (s, 9H). ^{19}F NMR (376 MHz, DMSO) δ -60.88. ^{13}C NMR (101 MHz, DMSO) δ 169.68, 169.28, 145.76, 142.86, 142.49, 141.98, 140.06, 139.48, 129.86, 129.20, 128.61, 126.48, 125.79, 123.10, 120.43, 106.61, 62.97, 62.70, 45.96, 45.66, 30.65, 21.28. HRMS-ESI (m/z) [M+H]+ calc'd for $C_{28}H_{34}BF_3N_5O_6S$, 636.2275, found 636.2294.

N-(2-chloro-1-(6-methyl-4,8-dioxo-1,3,6,2-dioxazaborocan-2-yl)ethyl)-4-(5-(p-tolyl)-3-(trifluoromethyl)-1H-pyrazol-1-yl)benzenesulfonamide (55):

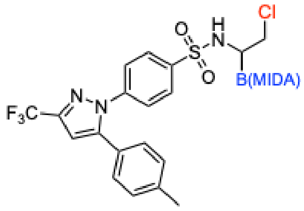

To a stirred solution of compound **5** (50 mg, 0.089 mmol, 1.0 eq) in DCM, was added benzyl triethylammonium chloride (30 mg, 0.134 mmol, 1.5 eq) under nitrogen atmosphere. The reaction mixture was cooled to 0 °C and added $BF_3.Et_2O$ (13 μL, 0.105 mmol) dropwise and allowed to stir for 5h at 0 °C. After the complete consumption of starting material (monitored by TLC), saturated $NaHCO_3$ solution was added and allowed to stir for 20 min at room temperature. The layers were separated, and the aqueous layer was further extracted with DCM and the combined organic layers were dried over anhydrous Na_2SO_4, filtered and the solvents were removed under vacuum. The obtained crude was purified by using flash Column chromatography to get the compound **55** (40 mg, 76% yield).

^{1}H NMR (400 MHz, CD_3CN) δ 7.91 – 7.83 (m, 2H), 7.54 – 7.46 (m, 2H), 7.27 – 7.13 (m, 4H), 6.92 (s, 1H), 6.08 – 6.01 (m, 1H), 4.00 (dd, *J* = 17.0, 4.3 Hz, 2H), 3.88 (t, *J* = 16.9 Hz, 2H), 3.23 – 3.11 (m, 3H), 2.99 (s, 3H), 2.34 (s, 3H). ^{11}B NMR (128 MHz, CD_3CN) δ 9.48. ^{19}F NMR (376 MHz, CD_3CN) δ -62.82. ^{13}C NMR (101 MHz, CD_3CN) δ 168.87, 168.27, 146.67, 143.84, 143.46, 140.87, 140.81, 130.45, 129.90, 128.95, 127.12, 126.71, 106.91, 63.43, 63.23, 46.90, 45.68, 21.30. HRMS- ESI (m/z) [M+H]+ calc'd for $C_{24}H_{24}BClF_3N_4O_6S$ (M+H$^+$): 599.1150; found: 599.1168.

***N*-(2-azido-1-(6-methyl-4,8-dioxo-1,3,6,2-dioxazaborocan-2-yl)ethyl)-4-(5-(*p*-tolyl)-3-(trifluoromethyl)-1*H*-pyrazol-1-yl)benzenesulfonamide (56):**

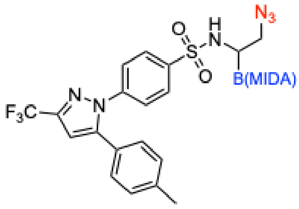

To a stirred solution of compound **5** (50 mg, 0.089 mmol, 1.0 eq) in DMF, was added sodium azide (17mg, 0.267 mmol, 3.0 eq) under nitrogen atmosphere. The reaction mixture was heated to 75 °C allowed to stir for 10h at same temperature. After the complete consumption of starting material (monitored by TLC), the reaction mixture was allowed to cool to room temperature and diluted with cold water and ethyl acetate. The layers were separated, and the aqueous layer was further extracted wit ethyl acetate, and the combined organic layers were dried over anhydrous Na_2SO_4, filtered and the solvents were removed under vacuum. The obtained crude was purified by using flash column chromatography to afford the compound **56** (38 mg, 74% yield).

^{1}H NMR (400 MHz, CD_3CN) δ 7.92 – 7.82 (m, 2H), 7.53 – 7.45 (m, 2H), 7.24 – 7.12 (m, 4H), 6.92 (s, 1H), 6.03 (t, *J* = 6.1 Hz, 1H), 4.06 – 3.81 (m, 4H), 3.25 – 3.11 (m, 3H), 2.99 (s, 3H), 2.34 (s, 3H). ^{11}B NMR (128 MHz, CD_3CN) δ 9.54. ^{19}F NMR (376 MHz, CD_3CN) δ -62.84. ^{13}C NMR (101 MHz, CD_3CN) δ 168.91, 168.32, 146.68, 144.23, 143.85, 143.45, 140.89, 140.82, 130.45, 129.91, 128.95, 127.12, 126.72, 123.87, 121.21, 106.92, 63.44, 63.24, 46.90, 45.68, 21.30. HRMS- ESI (m/z) [M+H]+ calc'd for $C_{24}H_{24}BF_3N_7O_6S$ (M+H$^+$): 606.1548; found: 606.1565.

6-methyl-2-(2-phenyl-1-((4-(5-(*p*-tolyl)-3-(trifluoromethyl)-1*H*-pyrazol-1-yl)phenyl)sulfonyl)-4,5-dihydro-1*H*-imidazol-5-yl)-1,3,6,2-dioxazaborocane-4,8-dione (57):

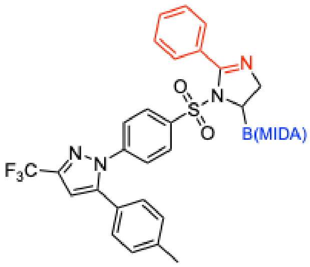

To a stirred solution of the corresponding aziridine **5** (120 mg, 0.21 mmol) in the benzo nitrile (3 mL) was added $BF_3.Et_2O$ (0.04 mL, 0.3 mmol) at room temperature. After stirring at room temperature for 1 h, an aqueous saturated solution of sodium bicarbonate (5 mL) was added, and the mixture was stirred at room temperature for 5 min. Then, the aqueous phase was extracted with Dichloromethane (3 × 10 mL), and the combined organic layers were dried over anhydrous Na_2SO_4, filtered, and concentrated in vacuo. Flash column chromatography on silica gel (Hexane:Ethyleacetate 20:80) to afford **57** (107 mg, 76%) as a white solid.

^{1}H NMR (400 MHz, DMSO) δ 7.73 – 7.65 (m, 2H), 7.64 – 7.58 (m, 2H), 7.55 – 7.51 (m, 2H), 7.47 – 7.43 (m, 1H), 7.35 (t, *J* = 7.6 Hz, 2H), 7.19 – 7.15 (m, 3H), 7.10 (d, *J* = 8.1 Hz, 2H), 4.37 (d, *J* = 17.2 Hz, 1H), 4.25 (d, *J* = 16.9 Hz, 1H), 4.12 – 4.01 (m, 3H), 3.66 (dd, *J* = 16.0, 1.8 Hz, 1H), 3.17 (s, 3H), 2.81 (dd, *J* = 15.9, 9.0 Hz, 1H), 2.25 (d, *J* = 9.1 Hz, 4H). ^{19}F NMR (377 MHz, DMSO) δ -60.92. ^{13}C NMR (101 MHz, DMSO) δ 169.22, 168.58, 160.03, 146.00, 143.40, 139.71, 137.03, 131.66, 129.91, 129.79, 129.34, 129.27, 128.20, 127.09, 63.24, 62.46, 57.15, 46.44, 21.31. HRMS-ESI (m/z) [M+H]+ calc'd for $C_{31}H_{28}BF_3N_5O_6S$, 666.1800, found 666.1840.

N-(2-bromo-1-(6-methyl-4,8-dioxo-1,3,6,2-dioxazaborocan-2-yl)ethyl)-4-(5-(p-tolyl)-3 (trifluoromethyl)-1H-pyrazol-1-yl)benzenesulfonamide (58):

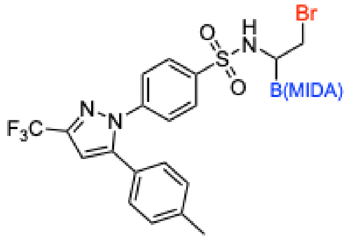

To a stirred solution of compound **5** (68 mg, 0.122 mmol, 1.0 equiv) in DCM, was added benzyl triethylammonium bromide (36 mg, 0.134 mmol, 1.1 equiv) under nitrogen atmosphere. The reaction mixture was cooled to 0 °C and added $BF_3 \cdot Et_2O$ (7 μL, 0.15 mmol) dropwise and allowed to stir for 1h at 0 °C. After the complete consumption of starting material (monitored by TLC), saturated $NaHCO_3$ solution was added and allowed to stir for 20 min at room temperature. The layers were separated, and the aqueous layer was further extracted with DCM and the combined organic layers were dried over anhydrous Na_2SO_4, filtered and the solvents were removed under vacuum. The obtained crude was purified by using flash Column chromatography to get compound **58** (63 mg, 81%).

^{1}H NMR (400 MHz, CD_3CN) δ 7.93 (d, *J* = 8.7 Hz, 2H), 7.48 (d, *J* = 8.7 Hz, 2H), 7.23 – 7.13 (m, 4H), 6.93 (s, 1H), 5.86 (d, *J* = 9.8 Hz, 1H), 4.00 (dd, *J* = 17.0, 1.8 Hz, 2H), 3.86 (t, *J* = 16.9 Hz, 2H), 3.65 – 3.56 (m, 1H), 3.38 (dd, *J* = 10.9, 3.9 Hz, 1H), 3.24 (dd, *J* = 10.9, 3.9 Hz, 1H), 3.01 (s, 3H), 2.34 (s, 3H). ^{11}B NMR (128 MHz, CD_3CN) δ 9.73. ^{19}F NMR (376 MHz, CD_3CN) δ -62.84. ^{13}C NMR (101 MHz, DMSO) δ 168.86, 168.23, 145.29, 142.79, 142.41, 142.26, 142.04, 141.66, 141.41, 139.08, 129.44, 128.72, 127.92, 125.98, 125.32, 125.28, 122.65, 119.97, 118.04, 106.06, 62.32, 62.24, 45.59, 36.57, 20.83. HRMS- ESI (m/z) [M+H]+ calc'd for $C_{24}H_{24}BBrF_3N_4O_6S$ ($M+H^+$): 643.0640; found: 643.0662.

***N*-(1-(6-methyl-4,8-dioxo-1,3,6,2-dioxazaborocan-2-yl)-2-(phenylthio)ethyl)-4-(5-(*p*-tolyl)-3-(trifluoromethyl)-1*H*-pyrazol-1-yl)benzenesulfonamide (59):**

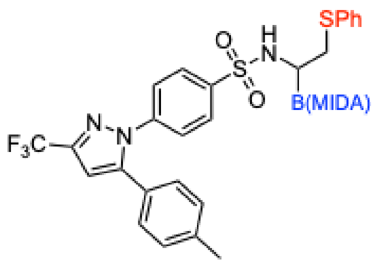

To a stirred solution of compound **58** (250 mg, 0.388 mmol, 1.0 equiv), was added sodium benzenethiolate (77 mg, 0.582 mmol, 1.5 equiv) and sodium iodide (88 mg, 0.582 mmol, 1.5 equiv) in acetone and the reaction mixture was heated to 55 °C. The reaction mixture was stirred for 12h, after completion of starting material (monitored by TLC) the reaction mixture was cooled to room temperature and the solvents were removed under reduced pressure. This crude product was purified by silica gel flash chromatography to afford compound **59** (201 mg, 77% yield).

^{1}H NMR (400 MHz, CD_3CN) δ 7.84 – 7.76 (m, 2H), 7.33 – 7.26 (m, 2H), 7.20 – 7.09 (m, 6H), 7.09 – 7.02 (m, 3H), 6.90 (s, 1H), 5.82 (d, *J* = 10.0 Hz, 1H), 4.03 (dd, *J* = 17.0, 5.3 Hz, 2H), 3.89 (dd, *J* = 17.0, 12.4 Hz, 2H), 3.45 (dt, *J* = 10.1, 5.2 Hz, 1H), 3.05 (s, 3H), 2.92 – 2.79 (m, 2H), 2.29 (s, 3H). ^{11}B NMR (128 MHz, CD_3CN) δ 9.79. ^{19}F NMR (376 MHz, CD_3CN) δ -62.80. ^{13}C NMR (101 MHz, CD_3CN) δ 168.69, 168.63, 146.46, 144.14, 143.76, 143.38, 143.15, 142.76, 140.71, 137.95, 130.44, 129.88, 129.86, 129.75, 128.73, 126.75, 126.52, 123.89, 121.22, 107.03, 63.65, 63.50, 46.86, 37.03, 21.29. HRMS- ESI (m/z) [M+H]+ calc'd for $C_{30}H_{29}BF_3N_4O_6S_2$ (M+H$^+$): 673.1568; found: 673.1614.

***S*-(2-(6-methyl-4,8-dioxo-1,3,6,2-dioxazaborocan-2-yl)-2-((4-(5-(*p*-tolyl)-3-(trifluoromethyl)-1*H*-pyrazol-1-yl)phenyl)sulfonamido)ethyl) ethanethioate (60):**

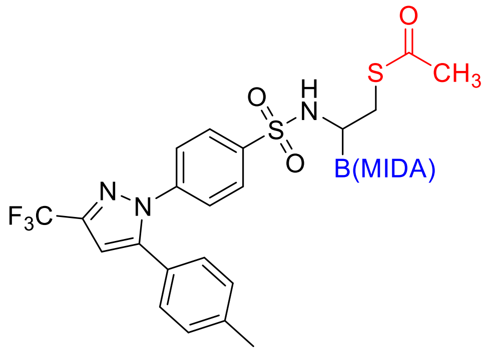

To a stirred solution of compound **58** (150 mg, 0.233 mmol, 1.0 equiv), was added potassium ethanethioate (40 mg, 0.350 mmol, 1.5 equiv) and sodium iodide (52 mg, 0.350 mmol, 1.5 equiv) in DMF and the reaction mixture was heated to 60 °C. The reaction mixture was stirred for 12 h, after completion of starting material (monitored by TLC) the reaction mixture was cooled to room temperature and the solvents were removed under reduced pressure. This crude product was purified by silica gel flash chromatography to afford compound **60** (105 mg, 71% yield).

^{1}H NMR (400 MHz, CD_3CN) δ 7.89 – 7.82 (m, 2H), 7.49 – 7.42 (m, 2H), 7.20 (s, 4H), 6.92 (s, 1H), 5.71 (d, *J* = 9.8 Hz, 1H), 4.02 (dd, *J* = 17.0, 5.0 Hz, 2H), 3.87 (t, *J* = 16.9 Hz, 2H), 3.40 (ddd, *J* = 9.8, 6.2, 5.0 Hz, 1H), 3.06 (s, 3H), 2.95 (dd, *J* = 13.9, 5.0 Hz, 1H), 2.74 (dd, *J* = 13.9, 6.2 Hz, 1H), 2.34 (s, 3H), 2.14 (s, 3H). ^{19}F NMR (376 MHz, CD_3CN) δ -62.82. ^{13}C NMR (101 MHz, CD_3CN) δ 196.23, 168.62, 168.57, 146.60, 144.57, 144.19, 143.81, 143.44, 143.32, 142.82, 140.71, 130.44, 129.95, 128.98, 126.89, 126.75, 123.86, 121.20, 106.96, 63.59, 63.42, 46.77, 32.00, 30.74, 21.33. HRMS- ESI (m/z) [M+H]+ calc'd for $C_{26}H_{27}BF_3N_4O_7S_2$ ($M+H^+$): 639.1361; found: 639.1399.

2-(6-methyl-4,8-dioxo-1,3,6,2-dioxazaborocan-2-yl)-2-((4-(5-(*p*-tolyl)-3-(trifluoromethyl)-1*H*-pyrazol-1-yl)phenyl)sulfonamido)ethyl benzoate (61):

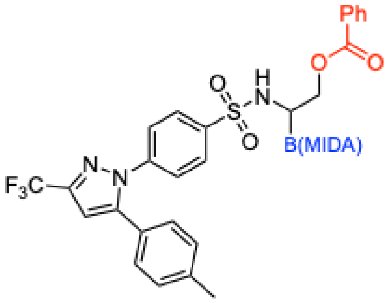

To a stirred solution of compound **58** (150 mg, 0.233 mmol, 1.0 equiv), was added sodium benzoate (50 mg, 0.350 mmol, 1.5 equiv) and sodium iodide (52 mg, 0.350 mmol, 1.5 equiv) in DMF and the reaction mixture was heated to 60 °C. The reaction mixture was stirred for 12h, after completion of starting material (monitored by TLC) the reaction mixture was cooled to room temperature and diluted with chilled water and ethyl acetate. The aqueous layer was further extracted with ethyl acetate (2 x 10 mL) and the combined organic layers were dried over anhydrous Na_2SO_4, evaporated under reduced pressure. This crude product was purified by silica gel flash chromatography to afford compound **61** (97 mg, 61% yield).

^{1}H NMR (400 MHz, CD_3CN) δ 8.02 (dt, *J* = 7.0, 1.4 Hz, 2H), 7.89 – 7.81 (m, 2H), 7.62 – 7.55 (m, 1H), 7.45 (t, *J* = 7.8 Hz, 2H), 7.41 – 7.32 (m, 2H), 7.21 – 7.12 (m, 4H), 6.91 (d, *J* = 3.7 Hz, 1H), 6.03 (d, *J* = 10.2 Hz, 1H), 4.15 – 3.99 (m, 3H), 3.96 – 3.83 (m, 3H), 3.52 (dt, *J* = 10.3, 3.2 Hz, 1H), 3.11 (s, 3H), 2.28 (s, 3H). ^{11}B NMR (128 MHz, CD_3CN) δ 10.45. ^{19}F NMR (377 MHz, CD_3CN) δ -62.85. ^{13}C NMR (101 MHz, CD_3CN) δ 168.76, 168.68, 167.05, 146.63, 146.57, 144.22, 143.84, 143.30, 142.88, 142.83, 140.72, 134.07, 131.06, 130.65, 130.46, 129.97, 129.89, 129.35, 128.71, 126.91, 126.70, 123.87, 121.21, 107.00, 65.62, 63.52, 63.40, 47.02, 21.28. HRMS- ESI (m/z) [M+H]+ calc'd for $C_{31}H_{29}BF_3N_4O_8S$ ($M+H^+$): 685.1746; found: 685.1778.

(2-((4-methoxyphenyl)amino)-1-((4-(5-(*p*-tolyl)-3-(trifluoromethyl)-1*H*-pyrazol-1-yl)phenyl)sulfonamido)ethyl)boronic acid (62):

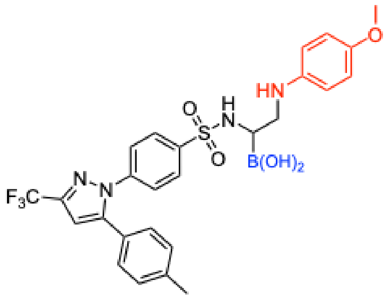

To an oven dried 25 mL round bottle flask with a magnetic stir bar was charged with **53** (0.2 mmol, 137 mg) in 5 mL of MeOH 3.0 N HCl (2.5 equiv.) was then added dropwise to the solution at room temperature and stirred for 4 h. Upon concentration at < 25 °C, the resulting residue was dissolved in 1:1 MeCN:H_2O and lyophilized to afford the title compound **62** as a powder (80 mg, 70% yield, observed along with MIDA acid). ^{1}H NMR (400 MHz, DMSO) δ 7.95 (tt, *J* = 6.5, 3.3 Hz, 2H), 7.67 – 7.60 (m, 2H), 7.45 – 7.41 (m, 1H), 7.25 (td, *J* = 7.5, 3.5 Hz, 6H), 7.09 – 6.96 (m, 2H), 3.84 – 3.69 (m, 4H), 3.31 – 3.18 (m, 2H), 2.36 (d, *J* = 3.3 Hz, 3H). ^{19}F NMR (376 MHz, DMSO) δ -60.92. ^{11}B NMR (128 MHz, DMSO) δ 18.81. ^{13}C NMR (101 MHz, DMSO) δ 145.81, 145.79, 142.27, 139.63, 129.93, 129.19, 128.60, 128.40, 126.77, 126.71, 125.67, 115.36, 115.06, 106.63, 55.77, 42.74, 21.27. HRMS-ESI (m/z) [M+H]+ calc'd for C_{26}H27B$F_3N_4O_5$S, 575.1747, found 575.1743.

***N*-(2-(*tert*-butylamino)-1-(potassiumtrifluoro-λ4-boraneyl)ethyl)-4-(5-(*p*-tolyl)-3-(trifluoromethyl)-1*H*-pyrazol-1-yl)benzenesulfonamide (63):**

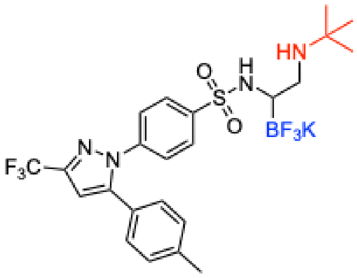

To a stirred solution of compound **54** (250 mg, 0.39 mmol, 1.0 equiv) in 3 mL of MeCN:MeOH (4:1), was added 4.5 M aqueous solution of KHF_2 (0.44 mL, 5.0 equiv) and allowed to stir for 16 h at room temperature. After completion of starting material, the volatiles were removed under reduced pressure. The mixture was dissolved in acetonitrile and stirred for 10-15 min and then filtered followed by washing the solid with additional acetonitrile for two times. The combined filtrate was evaporated under reduced pressure and the obtained crude compound was triturated with Et_2O followed by hexanes. The white precipitate formed was filtered and dried to afford white solid **63** (164 mg, 71%).

^{1}H NMR (400 MHz, CD_3CN) δ 7.84 (d, *J* = 8.7 Hz, 2H), 7.48 (d, *J* = 8.7 Hz, 2H), 7.22 – 7.12 (m, 5H), 6.91 (s, 1H), 3.11 (dd, *J* = 14.8, 4.0 Hz, 1H), 2.95 (dd, *J* = 14.8, 8.6 Hz, 1H), 2.46 – 2.36 (m, 1H), 2.33 (s, 3H), 1.33 (s, 9H) ppm. ^{11}B NMR (128 MHz, DMSO) δ 2.43 ppm. ^{13}C NMR (101 MHz, CD_3CN) δ 146.66, 143.94

(q, J = 38.0 Hz), 143.50, 140.77, 140.66, 130.45, 129.90, 128.95, 127.09, 126.73, 123.87, 121.20, 106.96, 59.37, 44.69, 26.77, 21.32 ppm. HRMS- ESI (m/z) [M+H]+ calc'd for $C_{23}H_{27}BF_6KN_4O_2S$, 587.1484, found 587.1519.

1-(4-((2-(4-methoxyphenyl)-4-(4,4,5,5-tetramethyl-1,3,2-dioxaborolan-2-yl)pyrrolidin-1-yl)sulfonyl)phenyl)-5-(*p*-tolyl)-3-(trifluoromethyl)-1*H*-pyrazole (64):

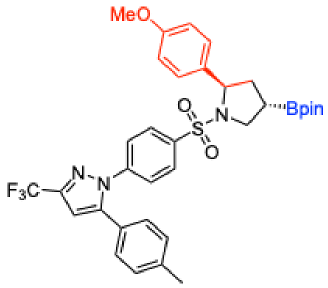

A 25 mL round-bottom flask was charged with compound **5** (281 mg, 0.5 mmol, 1.0 equiv), 1-methoxy-4-vinylbenzene (99 mg, 0.75 mmol, 1.5 equiv), NaI (149 mg, 1.0 mmol, 2.0 equiv), 4CzIPN (15.8 mg, 0.02 mmol, 4 mol%), and Ni(dtbbpy)Cl_2 (7.7 mg, 0.025 mmol, 8 mol%). The solids were dissolved in THF (5 mL, 0.08 M), and Et_3N (210 μL, 1.5 mmol, 3.0 equiv) was added. The reaction mixture was degassed for 10 min, sealed with a cone-lined cap, and stirred under blue LED irradiation (456 nm) at 70 °C for 48 h. After completion, the reaction was diluted with EtOAc (20 mL), quenched with saturated aqueous NH_4Cl (15 mL), and extracted with EtOAc (2 × 25 mL). The combined organic extracts were dried over Na_2SO_4, filtered, and concentrated. The crude residue was dissolved in CH_2Cl_2/MeOH (1:1, 20 mL), treated with pinacol (64 mg, 0.55 mmol, 1.1 equiv), and stirred at 45 °C for 12 h. After solvent removal under reduced pressure, the crude product was purified by Florisil silica gel flash chromatography to afford compound **64** (166 mg, 50% yield, 7:3 dr).

^{1}H NMR (400 MHz, $CDCl_3$) δ 7.73 – 7.52 (m, 2H), 7.36 – 7.27 (m, 2H), 7.13 – 7.03 (m, 4H), 7.00 (dd, J = 8.3, 3.1 Hz, 2H), 6.76 – 6.65 (m, 3H), 4.81 – 4.46 (m, 1H), 3.69 (s, 4H), 3.46 – 3.13 (m, 1H), 2.30 (s, 4H), 1.79 (ddd, J = 12.5, 5.8, 2.6 Hz, 1H), 1.27 (tt, J = 11.4, 7.4 Hz, 1H), 1.13 – 1.06 (m, 12H). ^{19}F NMR (377 MHz, $CDCl_3$) δ -62.40. ^{11}B NMR (128 MHz, $CDCl_3$) δ 32.68. ^{13}C NMR (101 MHz, $CDCl_3$) δ 158.89, 145.27, 145.22, 142.13, 139.77, 139.74, 138.66, 138.10, 134.71, 134.01, 129.74, 129.72, 128.70, 128.44, 128.24, 127.79, 127.18, 125.70, 125.66, 125.48, 125.36, 113.74, 113.64, 83.74, 64.85, 55.30, 55.27, 52.13, 40.02, 24.74, 24.68, 21.32. HRMS- ESI (m/z) [M+Na] calc'd for $C_{34}H_{37}BF_3N_3O_5SNa$, 690.2397, found 690.2343.

5-(4-methoxyphenyl)-1-((4-(5-(*p*-tolyl)-3-(trifluoromethyl)-1*H*-pyrazol-1-yl)phenyl)sulfonyl)pyrrolidin-3-ol (65):

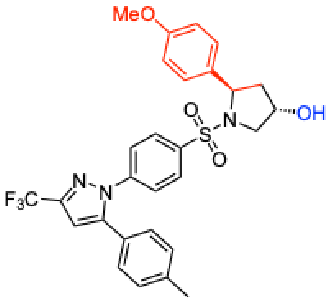

To a solution of compound **64** (66.7 mg, 0.1 mmol, 1.0 equiv) in THF/H_2O (1:1, 4.0 mL) was added $NaBO_3 \cdot 4H_2O$ (77 mg, 0.5 mmol, 5.0 equiv). The mixture was stirred at room temperature for 3 h, diluted with H_2O, and extracted with EtOAc. The combined organic extracts were dried over Na_2SO_4, filtered, and concentrated under reduced pressure. The residue was purified by flash column chromatography to afford compound **65** as a white solid (44 mg, 80% yield, 7:3 dr).

^{1}H NMR (400 MHz, $CDCl_3$) δ 7.59 – 7.52 (m, 2H), 7.29 (dd, *J* = 8.2, 6.3 Hz, 2H), 7.15 (d, *J* = 8.3 Hz, 2H), 7.11 – 6.98 (m, 5H), 6.74 – 6.64 (m, 3H), 4.74 – 4.65 (m, 1H), 4.34 – 4.23 (m, 1H), 3.69 (d, *J* = 1.7 Hz, 3H), 3.63 – 3.57 (m, 1H), 3.43 (dd, *J* = 10.9, 4.0 Hz, 1H), 2.29 (s, 4H), 1.89 (dt, *J* = 13.7, 5.3 Hz, 1H). ^{19}F NMR (376 MHz, $CDCl_3$) δ -62.40. ^{13}C NMR (101 MHz, $CDCl_3$) δ 158.98, 145.28, 142.40, 139.83, 137.85, 133.72, 129.75, 129.71, 128.70, 128.32, 127.95, 125.62, 125.41, 113.91, 113.86, 70.24, 62.15, 56.64, 55.27, 44.01, 21.32. HRMS- ESI (m/z) [M+Na] calc'd for $C_{28}H_{26}F_3N_3O_4SNa$, 580.1494, found 580.1454.

(5-(4-methoxyphenyl)-1-((4-(5-(*p*-tolyl)-3-(trifluoromethyl)-1*H*-pyrazol-1-yl)phenyl)sulfonyl)pyrrolidin-3-yl)methanol (66):

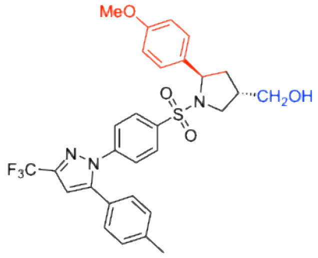

An oven-dried 10 mL vial with a magnetic stir bar was charged with compound **64** (66.7 mg, 0.1 mmol). The vial was sealed with a polypropylene open-top cap with PTFE/silicone septum, and evacuated and refilled with argon for three cycles, then CH_2Br_2 (35 μL, 0.4 mmol) and anhydrous THF (4 mL) was added. The mixture was cooled to −78 ℃, and then *n*-BuLi (0.16 mL, 0.39 mmol) was added dropwise to the solution for 5 min and stirred for 10 min. The solution was then warmed to room temperature and stirred for 21 h. Slowly add a premix solution of NaOH (2 M, aq.) and 35% H_2O_2 (2:1, 1.5 mL) at 0°C. The mixture was stirred at room temperature for an additional 3 h. The reaction was quenched with 10 mL of water. Extract the resulting mixture twice with 10 mL of EtOAc. Dry the combine organic layers over anhydrous Na_2SO_4.

Removing the solvent under reduced pressure. Purify the residue by flash column chromatography to afford compound **66** as a white solid (37 mg, 64%, 7:3 dr).

^{1}H NMR (400 MHz, $CDCl_3$) δ 7.56 – 7.49 (m, 2H), 7.32 – 7.26 (m, 2H), 7.11 – 7.04 (m, 4H), 7.00 (d, *J* = 8.2 Hz, 2H), 6.73 – 6.65 (m, 3H), 4.55 (dd, *J* = 8.9, 7.3 Hz, 1H), 3.77 (dd, *J* = 10.8, 7.5 Hz, 1H), 3.69 (d, *J* = 2.3 Hz, 3H), 3.58 – 3.42 (m, 2H), 3.29 – 3.22 (m, 1H), 2.32 – 2.23 (m, 4H), 2.17 – 2.04 (m, 1H), 1.63 – 1.58 (m, 1H). ^{19}F NMR (377 MHz, $CDCl_3$) δ -62.39. ^{13}C NMR (101 MHz, $CDCl_3$) δ 158.99, 145.28, 142.26, 139.81, 138.21, 133.70, 129.74, 128.70, 128.28, 127.83, 125.64, 125.39, 113.82, 63.88, 63.58, 55.27, 52.42, 40.65, 39.58, 21.32. HRMS- ESI (m/z) [M+Na] calc'd for $C_{29}H_{28}F_3N_3O_4SNa$, 594.1651, found 594.1613.

1-(4-((4-(3-chlorophenyl)-2-(4-methoxyphenyl)pyrrolidin-1-yl)sulfonyl)phenyl)-5-(*p*-tolyl)-3-(trifluoromethyl)-1*H*-pyrazole (67):

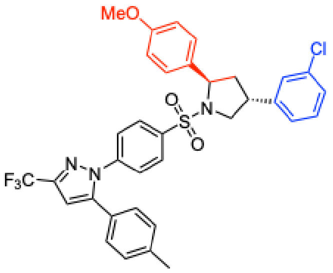

A 4 mL glass vial equipped with a magnetic stir bar was charged with [Ir(dF(CF_3)ppy)$_2$(dtbbpy)]PF_6 (1.1 mg, 1 mol %), 1-bromo-3-chlorobenzene (14.3 mg, 0.075 mmol), and compound **64** (66 mg, 0.1 mmol, 1.3 equiv) in DMF (1 mL). Morpholine (9 mg, 0.30 mmol, 1.5 equiv) was added. In a separate vial, $NiCl_2$·glyme (1.1 mg, 5 mol%) and dtbbpy (1.3 mg, 5 mol%) were dissolved in DMF (1 mL), sonicated for 30 s, and briefly heated to 100 °C until a clear green solution formed.[2] The two solutions were combined and irradiated with blue LEDs (450 nm) for 2 h. The reaction mixture was poured into brine and extracted with cold EtOAc (3 × 10 mL). The combined organic layers were dried over Na_2SO_4, filtered, and concentrated. Purification by flash column chromatography afforded compound **67** as a white solid (34 mg, 72%, 6:4 dr).

^{1}H NMR (400 MHz, $CDCl_3$) δ 7.71 – 7.49 (m, 2H), 7.41 – 7.26 (m, 2H), 7.17 – 7.04 (m, 6H), 7.04 – 6.85 (m, 4H), 6.79 – 6.75 (m, 1H), 6.75 – 6.69 (m, 1H), 6.67 (d, *J* = 2.8 Hz, 1H), 4.99 – 4.66 (m, 1H), 4.15 – 3.88 (m, 1H), 3.71 (dd, *J* = 6.9, 1.1 Hz, 3H), 3.46 – 3.34 (m, 1H), 3.31 – 2.97 (m, 1H), 2.29 (s, 3H), 2.11 – 1.92 (m, 2H). ^{19}F NMR (376 MHz, $CDCl_3$) δ -62.40. ^{13}C NMR (101 MHz, $CDCl_3$) δ 158.97, 145.29, 142.58, 141.46, 139.84, 137.50, 134.59, 134.14, 130.04, 129.75, 129.73, 128.71, 128.42, 128.17, 127.93, 127.29, 127.24, 127.22, 125.67, 125.53, 125.40, 125.28, 125.06, 113.94, 113.91, 64.14, 62.66, 55.41, 55.33, 55.30, 54.75, 44.17, 43.23, 42.11, 41.18, 21.33. HRMS- ESI (m/z) [M+Na] calc'd for $C_{34}H_{29}ClF_3N_3O_3SNa$, 674.1468, found 674.1419.

3-(5-(4-methoxyphenyl)-1-((4-(5-(*p*-tolyl)-3-(trifluoromethyl)-1*H*-pyrazol-1-yl)phenyl)sulfonyl)pyrrolidin-3-yl)propanenitrile (68):

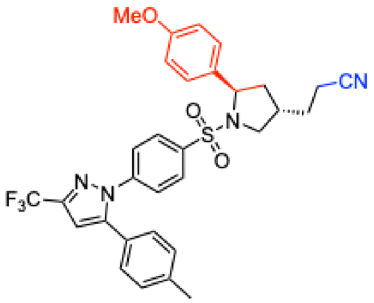

A 5 mL glass vial equipped with a magnetic stir bar was charged with compound **64** (66.7 mg, 0.1 mmol), the photo catalyst Ir(dF(CF$_3$)ppy$_2$)(dtbpy)]PF$_6$ (2.2 mg, 2 mol%) and DMAP (18.3 mg, 0.15 mmol, 1.5 equiv). The vial was then sealed with a rubber septum and evacuated/backfilled with argon three times. The acrylonitrile (21 mg, 0.4 mmol, 4.0 equiv) was then added followed by 2 mL of a degassed acetone/methanol (1:1) mixture.[3] This solution was then stirred while irradiated under blue LED (450 nm) for 16 hours at 30 °C. The content of the vial was then concentrated and purified by flash column chromatography afforded compound **68** as a white solid (48 mg, 81%, 7:3 dr).

^{1}H NMR (400 MHz, CDCl$_3$) δ 7.63 – 7.48 (m, 2H), 7.36 – 7.26 (m, 2H), 7.14 – 6.97 (m, 6H), 6.76 – 6.63 (m, 3H), 4.82 – 4.53 (m, 1H), 3.69 (d, *J* = 2.1 Hz, 4H), 3.13 – 2.91 (m, 1H), 2.44 – 2.16 (m, 6H), 2.10 – 1.86 (m, 1H), 1.74 – 1.45 (m, 3H). ^{19}F NMR (377 MHz, CDCl$_3$) δ -62.40. ^{13}C NMR (101 MHz, CDCl$_3$) δ 158.98, 145.29, 142.49, 139.86, 137.77, 133.92, 129.76, 129.74, 128.71, 128.31, 127.27, 125.64, 125.45, 118.79, 113.90, 62.45, 55.30, 55.28, 53.47, 41.20, 36.13, 28.20, 21.33. HRMS- ESI (m/z) [M+H]$^+$ calc'd for $C_{31}H_{30}F_3N_4O_3S$, 595.1991, found 595.1944.

5-(1-(6-methyl-4,8-dioxo-1,3,6,2-dioxazaborocan-2-yl)vinyl)-5*H*-thianthren-5-ium trifluoromethane sulfonate (69):

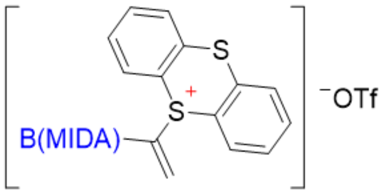

In an oven-dried 100 mL Schlenk vial equipped with a magnetic stir bar was charged with **3c** (0.3 mmol, 1.0 equiv) and Cs_2CO_3 (0.52 equiv), followed by the solvent acetonitrile (0.1 M) were added and the mixture was stirred at 23 °C for 14 h. The reaction mixtures were filtered through a celite and washed with MeCN. The volatiles were removed *in vacuo*. Dichloromethane was added to the crude reaction product, the resulting slurry was filtered, and the solid was washed with a 3 mL DCM and then dried and afforded the product **69** in 80% yield as a white solid.

^1H NMR (400 MHz, DMSO) δ 8.25 (dd, *J* = 7.9, 1.4 Hz, 2H), 8.16 – 8.01 (m, 2H), 8.01 – 7.72 (m, 4H), 6.66 (d, *J* = 3.6 Hz, 1H), 5.51 (d, *J* = 3.5 Hz, 1H), 4.37 (d, *J* = 17.4 Hz, 2H), 3.99 (d, *J* = 17.3 Hz, 2H), 2.54 (s, 3H). ^{19}F NMR (376 MHz, DMSO) δ -77.69. ^{11}B NMR (128 MHz, DMSO) δ 7.60. ^{13}C NMR (101 MHz, DMSO) δ 168.42, 158.67, 137.10, 135.83, 135.03, 130.81, 130.22, 122.73, 119.53, 116.83, 62.64, 47.61.

2-(1,2-bis(phenylamino)ethyl)-6-methyl-1,3,6,2-dioxazaborocane-4,8-dione (70):

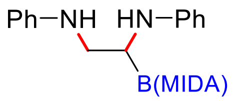

General procedure A with **3c** (210 mg, 0.3 mmol), aniline (32 mg, 0.36 mmol, 1.2 equiv) and K_2CO_3 (207 mg, 1.5 mmol, 5.0 equiv) to afford a white solid (36 mg, 54% yield). Rf = 0.4 (hexanes:ethyl acetate=3:7). ^1H NMR (500 MHz, $CDCl_3$) δ 7.12 (dt, *J* = 16.2, 7.6 Hz, 4H), 6.82 (dd, *J* = 21.5, 7.7 Hz, 5H), 6.73 (t, *J* = 7.3 Hz, 1H), 4.94 (s, 2H), 4.25 (t, *J* = 19.4 Hz, 2H), 3.97 (d, *J* = 17.2 Hz, 1H), 3.83 (d, *J* = 16.7 Hz, 1H), 3.43 (d, *J* = 17.2 Hz, 2H), 3.27 (d, *J* = 10.5 Hz, 1H), 2.89 (s, 3H). ^{13}C NMR (126 MHz, $CDCl_3$) δ 169.37, 169.22, 146.17, 143.81, 129.63, 129.57, 119.05, 117.06, 114.61, 63.02, 48.10, 46.52, 29.72. HRMS- ESI (m/z) $[M+H]^+$ calc'd for $C_{19}H_{23}BN_3O_4$, 368.1782, found 368.1776.

6. X-Ray crystallography data (3c, 30, 46, 69)

Experimental

Boryl thianthrenium dication (**3c**) was crystallized from Acetonitrile. The atoms are depicted with 50% probability ellipsoids. The crystallographic data are summarized in the following table

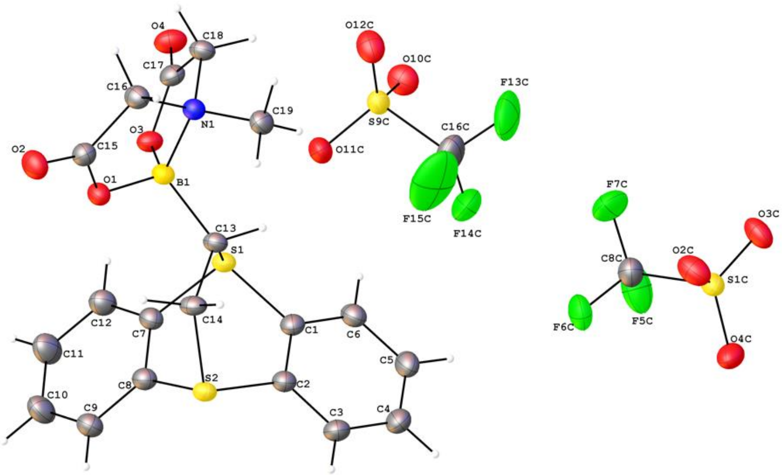

Figure S4: X-ray structure of boryl thianthrenium dication (**3c**)

Crystal data and structure refinement

Compound	3c
Formula	$C_{23}H_{21}BF_6N_2O_{10}S_4$
$D_{calc.}$/ g cm^{-3}	1.642
μ/mm^{-1}	3.800
Formula Weight	738.47
Colour	colourless
Shape	block-shaped
Size/mm^3	0.25×0.15×0.10
T/K	101(2)
Crystal System	triclinic
Space Group	P-1
a/Å	9.9502(2)
b/Å	11.4299(2)
c/Å	13.9995(3)
α/°	77.086(2)
β/°	74.258(2)
γ/°	86.353(2)
V/Å^3	1493.68(5)
Z	2
Z'	1
Wavelength/Å	1.54184
Radiation type	Cu K$_\alpha$
Θ_{min}/°	3.358
Θ_{max}/°	74.485
Measured Refl's.	29241
Indep't Refl's	6096
Refl's I≥2 σ(I)	5926
$R_{\rm int}$	0.0239
Parameters	438
Restraints	132
Largest Peak	1.145
Deepest Hole	-0.745
GooF	1.026
wR_2 (all data)	0.0949
wR_2	0.0945
R_1 (all data)	0.0386
R_1	0.0380

Structure Quality Indicators

**Reflections:**	d min (CuKα) 2Θ=149.0° 0.80	I/σ(I) 60.9	Rint m=4.80 2.39%	Full 135.4° 99.9
**Refinement:**	Shift -0.001	Max Peak 1.1	Min Peak -0.8	GooF 1.026

Bond lengths [Å] and angles [°].

Atom	x	y	z	U_{eq}
S(1)	3969.3(4)	5956.4(4)	3164.1(3)	21.14(10)
S(2)	1373.0(4)	7000.5(4)	2413.5(3)	21.79(10)

Atom	x	y	z	U_{eq}
O(1)	5607.8(13)	7929.0(12)	586.4(9)	23.8(3)
O(2)	6277.1(15)	9284.8(13)	-872.6(10)	31.5(3)
O(3)	6585.4(13)	6648.8(11)	1860.6(10)	23.5(3)
O(4)	8509.3(14)	6155.7(13)	2403.2(11)	31.2(3)
N(1)	6614.2(15)	8822.8(13)	1666.9(11)	20.9(3)
C(1)	2372.3(19)	6123.9(17)	4076.9(14)	23.4(4)
C(2)	1228.7(19)	6619.2(17)	3734.8(14)	24.0(4)
C(3)	-28(2)	6796.4(18)	4413.2(15)	28.1(4)
C(4)	-109(2)	6477(2)	5446.6(15)	31.1(4)
C(5)	1035(2)	5996.4(19)	5787.7(15)	30.1(4)
C(6)	2293(2)	5805.5(17)	5102.0(14)	26.4(4)
C(7)	3369.4(19)	5244.4(17)	2352.2(14)	23.5(4)
C(8)	2157.5(19)	5663.6(17)	2062.0(14)	24.0(4)
C(9)	1635(2)	5093.9(18)	1464.7(15)	28.5(4)
C(10)	2358(2)	4107(2)	1148.7(18)	36.4(5)
C(11)	3578(2)	3710(2)	1418.4(18)	38.0(5)
C(12)	4094(2)	4274.8(18)	2030.5(16)	30.0(4)
C(13)	4135.5(18)	7514.2(16)	2436.2(14)	21.2(3)
C(14)	2953.7(18)	7913.7(17)	1924.8(14)	23.7(4)
C(15)	6287.5(19)	8910.5(17)	2.4(14)	23.7(4)
C(16)	7033(2)	9492.5(17)	574.2(14)	24.8(4)
C(17)	7713.6(18)	6903.9(17)	2119.3(14)	24.2(4)
C(18)	7819.6(18)	8232.6(17)	2047.9(14)	23.4(4)
C(19)	5867(2)	9666.2(17)	2322.3(15)	25.6(4)
B(1)	5703(2)	7683.5(18)	1635.2(15)	21.2(4)
S(1C)	680.0(5)	7966.0(4)	9529.5(3)	26.02(11)
F(5C)	1363(2)	5783.8(13)	9273.0(12)	59.0(4)
F(6C)	986.1(15)	6974.5(13)	7966.0(9)	44.6(3)
F(7C)	2875.7(15)	7146.4(18)	8384.1(14)	65.0(5)
O(2C)	852.6(17)	9102.9(13)	8816.5(12)	37.8(3)
O(3C)	1463.6(17)	7839.9(14)	10274.7(11)	35.5(3)
O(4C)	-733.4(15)	7540.1(16)	9928.2(12)	40.5(4)
C(8C)	1517(2)	6904(2)	8752.2(15)	35.6(5)
S(9C)	6043.5(8)	7391.3(7)	4938.1(5)	24.18(17)
F(13C)	4924(3)	8564(2)	6356.9(16)	72.6(7)
F(14C)	3617(2)	7242(2)	6240.5(16)	71.7(7)
F(15C)	3973(3)	8911(3)	5114(2)	108.4(11)
O(10C)	6508(2)	6476.9(16)	5667.9(14)	34.6(4)
O(11C)	5480(2)	6958(3)	4237.0(16)	37.3(6)
O(12C)	7001(2)	8375.0(16)	4460.4(13)	35.7(4)
C(16C)	4561(3)	8068(3)	5701(2)	51.3(8)
C(1A)	6117(4)	8567(3)	5383(3)	35.6(18)
S(6)	5584(4)	7117(3)	5271(3)	26.6(9)
F(3)	6305(7)	8509(5)	6296(4)	49(2)
F(4)	5146(6)	9397(3)	5254(5)	43.0(19)
F(5)	7296(5)	8937(5)	4691(5)	42(2)
O(7)	6747(5)	6339(4)	5394(5)	34.6(4)
O(8)	4330(5)	6861(4)	6086(4)	36(2)
O(9)	5371(7)	7366(5)	4265(4)	26(3)

Table S1: Anisotropic Displacement Parameters (×10^4) for **3c** The anisotropic displacement factor exponent takes the form: $-2p^2[h^2a^{*2} \times U_{11} + ... + 2hka^* \times b^* \times U_{12}]$

Atom	U_{11}	U_{22}	U_{33}	U_{23}	U_{13}	U_{12}
S(1)	16.93(19)	25.6(2)	21.2(2)	-3.69(16)	-6.08(15)	-2.91(15)
S(2)	16.3(2)	27.4(2)	22.9(2)	-5.82(16)	-6.36(15)	-2.09(16)
O(1)	23.3(6)	28.2(7)	20.4(6)	-5.8(5)	-5.1(5)	-5.9(5)
O(2)	37.1(8)	35.4(7)	23.1(7)	-3.3(6)	-11.0(6)	-4.8(6)
O(3)	17.8(6)	24.5(6)	28.5(7)	-6.6(5)	-5.5(5)	-1.9(5)
O(4)	21.7(6)	31.3(7)	39.1(8)	-2.9(6)	-9.6(6)	2.1(5)
N(1)	18.5(7)	24.4(7)	20.6(7)	-5.1(6)	-5.7(6)	-1.9(6)
C(1)	19.8(8)	26.0(9)	23.7(9)	-5.6(7)	-3.6(7)	-5.1(7)
C(2)	19.1(8)	29.8(9)	24.2(9)	-7.3(7)	-5.2(7)	-4.0(7)
C(3)	19.3(9)	36.1(10)	29.5(10)	-8.7(8)	-5.2(7)	-3.5(7)
C(4)	24.5(9)	40.9(11)	26.5(10)	-9.9(8)	-0.8(8)	-6.6(8)
C(5)	30.0(10)	37.6(11)	22.2(9)	-6.4(8)	-3.7(8)	-9.5(8)
C(6)	25.0(9)	30.3(9)	25.0(9)	-4.8(7)	-8.2(7)	-6.0(7)
C(7)	21.9(8)	26.5(9)	23.1(9)	-5.5(7)	-6.3(7)	-4.5(7)
C(8)	20.5(8)	27.7(9)	23.9(9)	-4.9(7)	-5.9(7)	-3.1(7)
C(9)	25.1(9)	34.1(10)	29.1(10)	-9.1(8)	-9.4(8)	-3.4(8)
C(10)	35.7(11)	40.7(12)	41.7(12)	-20.0(10)	-15.5(9)	-0.9(9)
C(11)	38.1(12)	37.2(11)	46.0(13)	-21.1(10)	-14.9(10)	5.9(9)
C(12)	26.8(9)	31.9(10)	33.8(10)	-10.2(8)	-9.8(8)	1.4(8)
C(13)	17.7(8)	23.9(8)	22.8(8)	-3.8(7)	-7.3(7)	-2.6(6)
C(14)	17.9(8)	26.5(9)	25.8(9)	-0.9(7)	-6.8(7)	-5.8(7)
C(15)	21.1(8)	26.8(9)	22.9(9)	-5.6(7)	-4.9(7)	-1.0(7)
C(16)	24.5(9)	27.6(9)	21.7(9)	-2.3(7)	-6.3(7)	-5.2(7)
C(17)	18.0(8)	28.9(9)	23.9(9)	-4.4(7)	-2.5(7)	-3.5(7)
C(18)	18.7(8)	27.3(9)	25.4(9)	-4.3(7)	-8.6(7)	-2.6(7)
C(19)	24.2(9)	27.3(9)	27.7(9)	-10.5(7)	-7.0(7)	-0.8(7)
B(1)	18.8(9)	24.4(9)	21.5(9)	-6.0(8)	-5.7(7)	-3.3(7)
S(1C)	24.5(2)	31.7(2)	23.3(2)	-8.13(18)	-5.37(17)	-6.56(17)
F(5C)	99.4(13)	38.2(8)	47.1(8)	-17.3(7)	-28.0(9)	10.1(8)
F(6C)	52.8(8)	58.3(8)	27.2(6)	-18.3(6)	-9.9(6)	-4.3(7)
F(7C)	28.2(7)	98.8(13)	73.3(11)	-46.1(10)	-0.7(7)	5.5(8)
O(2C)	45.2(9)	34.3(8)	37.9(8)	-2.2(6)	-20.0(7)	-8.7(7)
O(3C)	46.2(9)	37.3(8)	28.5(7)	-5.4(6)	-18.7(7)	-8.6(7)
O(4C)	28.3(7)	54.1(10)	39.8(8)	-22.6(7)	2.8(6)	-13.6(7)
C(8C)	32.9(11)	46.8(12)	29.7(10)	-14.6(9)	-7.4(8)	0.4(9)
S(9C)	23.0(4)	28.3(3)	23.6(3)	-9.6(3)	-7.2(3)	1.5(3)
F(13C)	90.0(16)	73.0(14)	56.0(12)	-47.1(11)	6.1(11)	-2.7(12)
F(14C)	37.0(10)	127(2)	53.2(12)	-47.9(13)	10.3(9)	-16.3(11)
F(15C)	88.0(19)	131(2)	91.7(19)	-25.3(17)	-16.5(15)	79.6(19)
O(10C)	39.6(9)	30.5(8)	32.7(10)	0.1(7)	-13.3(8)	-0.7(7)
O(11C)	29.1(10)	59.3(17)	29.5(10)	-21.2(9)	-7.0(8)	-4.9(10)
O(12C)	46.8(11)	30.7(9)	29.2(9)	-4.7(7)	-8.8(8)	-8.0(8)
C(16C)	43.7(16)	67(2)	43.1(16)	-24.7(15)	-4.0(13)	16.3(15)
C(1A)	34(3)	31(3)	43(3)	-12(3)	-10(3)	-1(3)
S(6)	25(2)	27.6(18)	30(2)	-9.5(15)	-8.9(15)	-2.6(14)
F(3)	65(5)	45(4)	45(4)	-17(3)	-17(4)	-11(4)
F(4)	39(4)	30(3)	59(4)	-9(3)	-12(3)	5(3)
F(5)	36(4)	40(4)	49(4)	-16(4)	-1(3)	-11(3)
O(7)	39.6(9)	30.5(8)	32.7(10)	0.1(7)	-13.3(8)	-0.7(7)

Atom	U_{11}	U_{22}	U_{33}	U_{23}	U_{13}	U_{12}
O(8)	29(4)	54(5)	32(4)	-19(4)	-14(4)	6(4)
O(9)	35(5)	26(5)	21(4)	-7(3)	-14(4)	1(4)

Table S2: Bond Lengths in Å for **3c**

Atom	Atom	Length/Å
S(1)	C(1)	1.7786(19)
S(1)	C(7)	1.7759(18)
S(1)	C(13)	1.8361(18)
S(2)	C(2)	1.7708(19)
S(2)	C(8)	1.7783(19)
S(2)	C(14)	1.8268(18)
O(1)	C(15)	1.331(2)
O(1)	B(1)	1.460(2)
O(2)	C(15)	1.207(2)
O(3)	C(17)	1.336(2)
O(3)	B(1)	1.456(2)
O(4)	C(17)	1.204(2)
N(1)	C(16)	1.507(2)
N(1)	C(18)	1.504(2)
N(1)	C(19)	1.503(2)
N(1)	B(1)	1.649(2)
C(1)	C(2)	1.392(3)
C(1)	C(6)	1.381(3)
C(2)	C(3)	1.384(3)
C(3)	C(4)	1.392(3)
C(4)	C(5)	1.387(3)
C(5)	C(6)	1.394(3)
C(7)	C(8)	1.398(3)
C(7)	C(12)	1.377(3)
C(8)	C(9)	1.384(3)
C(9)	C(10)	1.390(3)
C(10)	C(11)	1.387(3)
C(11)	C(12)	1.394(3)

Atom	Atom	Length/Å
C(13)	C(14)	1.532(2)
C(13)	B(1)	1.649(3)
C(15)	C(16)	1.502(3)
C(17)	C(18)	1.508(3)
S(1C)	O(2C)	1.4388(15)
S(1C)	O(3C)	1.4411(14)
S(1C)	O(4C)	1.4373(15)
S(1C)	C(8C)	1.822(2)
F(5C)	C(8C)	1.320(3)
F(6C)	C(8C)	1.330(2)
F(7C)	C(8C)	1.333(2)
S(9C)	O(10C)	1.4380(18)
S(9C)	O(11C)	1.440(2)
S(9C)	O(12C)	1.4400(19)
S(9C)	C(16C)	1.812(3)
F(13C)	C(16C)	1.317(3)
F(14C)	C(16C)	1.323(3)
F(15C)	C(16C)	1.335(3)
C(1A)	S(6)	1.8237
C(1A)	F(3)	1.3286
C(1A)	F(4)	1.3291
C(1A)	F(5)	1.3246
S(6)	O(7)	1.4389
S(6)	O(8)	1.4379
S(6)	O(9)	1.4426
F(4)	F(4)[1]	1.454(8)

[1] 1-x,2-y,1-z

Table S3: Bond Angles in ° for **3c**

Atom	Atom	Atom	Angle/°
C(1)	S(1)	C(13)	97.75(8)
C(7)	S(1)	C(1)	100.63(9)
C(7)	S(1)	C(13)	100.94(8)
C(2)	S(2)	C(8)	99.81(9)
C(2)	S(2)	C(14)	102.23(8)
C(8)	S(2)	C(14)	96.50(9)
C(15)	O(1)	B(1)	113.95(14)
C(17)	O(3)	B(1)	114.08(14)
C(16)	N(1)	B(1)	103.41(13)
C(18)	N(1)	C(16)	113.62(14)
C(18)	N(1)	B(1)	103.70(13)
C(19)	N(1)	C(16)	109.24(14)
C(19)	N(1)	C(18)	110.18(14)

Atom	Atom	Atom	Angle/°
C(19)	N(1)	B(1)	116.61(14)
C(2)	C(1)	S(1)	118.64(14)
C(6)	C(1)	S(1)	120.24(15)
C(6)	C(1)	C(2)	121.07(17)
C(1)	C(2)	S(2)	119.52(14)
C(3)	C(2)	S(2)	119.52(15)
C(3)	C(2)	C(1)	120.95(18)
C(2)	C(3)	C(4)	118.04(18)
C(5)	C(4)	C(3)	121.04(18)
C(4)	C(5)	C(6)	120.70(18)
C(1)	C(6)	C(5)	118.19(18)
C(8)	C(7)	S(1)	119.58(14)
C(12)	C(7)	S(1)	119.45(15)

Atom	Atom	Atom	Angle/°
C(12)	C(7)	C(8)	120.96(17)
C(7)	C(8)	S(2)	118.12(14)
C(9)	C(8)	S(2)	121.02(15)
C(9)	C(8)	C(7)	120.74(18)
C(8)	C(9)	C(10)	118.31(19)
C(11)	C(10)	C(9)	120.80(19)
C(10)	C(11)	C(12)	120.9(2)
C(7)	C(12)	C(11)	118.29(19)
C(14)	C(13)	S(1)	113.55(12)
C(14)	C(13)	B(1)	113.39(15)
B(1)	C(13)	S(1)	108.32(12)
C(13)	C(14)	S(2)	116.30(13)
O(1)	C(15)	C(16)	111.62(15)
O(2)	C(15)	O(1)	123.42(17)
O(2)	C(15)	C(16)	124.95(17)
C(15)	C(16)	N(1)	106.97(14)
O(3)	C(17)	C(18)	111.29(15)
O(4)	C(17)	O(3)	123.70(17)
O(4)	C(17)	C(18)	124.97(17)
N(1)	C(18)	C(17)	106.95(14)
O(1)	B(1)	N(1)	103.55(14)
O(1)	B(1)	C(13)	110.76(14)
O(3)	B(1)	O(1)	112.27(15)
O(3)	B(1)	N(1)	103.42(13)
O(3)	B(1)	C(13)	110.90(15)
N(1)	B(1)	C(13)	115.61(14)
O(2C)	S(1C)	O(3C)	114.49(9)
O(2C)	S(1C)	C(8C)	103.54(10)
O(3C)	S(1C)	C(8C)	103.68(10)
O(4C)	S(1C)	O(2C)	115.04(10)
O(4C)	S(1C)	O(3C)	114.74(10)
O(4C)	S(1C)	C(8C)	103.17(9)
F(5C)	C(8C)	S(1C)	112.00(14)

Atom	Atom	Atom	Angle/°
F(5C)	C(8C)	F(6C)	107.01(17)
F(5C)	C(8C)	F(7C)	108.9(2)
F(6C)	C(8C)	S(1C)	111.59(15)
F(6C)	C(8C)	F(7C)	107.55(17)
F(7C)	C(8C)	S(1C)	109.68(15)
O(10C)	S(9C)	O(11C)	115.25(15)
O(10C)	S(9C)	O(12C)	114.38(12)
O(10C)	S(9C)	C(16C)	103.89(13)
O(11C)	S(9C)	C(16C)	104.29(14)
O(12C)	S(9C)	O(11C)	113.57(14)
O(12C)	S(9C)	C(16C)	103.58(13)
F(13C)	C(16C)	S(9C)	111.7(2)
F(13C)	C(16C)	F(14C)	106.5(2)
F(13C)	C(16C)	F(15C)	108.3(3)
F(14C)	C(16C)	S(9C)	110.4(2)
F(14C)	C(16C)	F(15C)	109.1(3)
F(15C)	C(16C)	S(9C)	110.8(2)
F(3)	C(1A)	S(6)	111.1
F(3)	C(1A)	F(4)	107.4
F(4)	C(1A)	S(6)	110.8
F(5)	C(1A)	S(6)	111.3
F(5)	C(1A)	F(3)	108.4
F(5)	C(1A)	F(4)	107.8
O(7)	S(6)	C(1A)	104.0
O(7)	S(6)	O(9)	114.7
O(8)	S(6)	C(1A)	102.9
O(8)	S(6)	O(7)	115.0
O(8)	S(6)	O(9)	114.8
O(9)	S(6)	C(1A)	103.1
C(1A)	F(4)	F(4)[1]	146.3(8)

[1] 1-x,2-y,1-z

Table S4: Torsion Angles in ° for **3c**

Atom	Atom	Atom	Atom	Angle/°
S(1)	C(1)	C(2)	S(2)	2.9(2)
S(1)	C(1)	C(2)	C(3)	-178.39(15)
S(1)	C(1)	C(6)	C(5)	177.54(14)
S(1)	C(7)	C(8)	S(2)	6.6(2)
S(1)	C(7)	C(8)	C(9)	-177.28(15)
S(1)	C(7)	C(12)	C(11)	178.11(17)
S(1)	C(13)	C(14)	S(2)	17.90(19)
S(1)	C(13)	B(1)	O(1)	114.48(14)
S(1)	C(13)	B(1)	O(3)	-10.87(17)
S(1)	C(13)	B(1)	N(1)	-128.16(13)
S(2)	C(2)	C(3)	C(4)	179.44(15)
S(2)	C(8)	C(9)	C(10)	174.99(16)
O(1)	C(15)	C(16)	N(1)	-6.1(2)
O(2)	C(15)	C(16)	N(1)	172.73(18)
O(3)	C(17)	C(18)	N(1)	-1.9(2)

Atom	Atom	Atom	Atom	Angle/°
O(4)	C(17)	C(18)	N(1)	-179.66(17)
C(1)	S(1)	C(7)	C(8)	44.00(17)
C(1)	S(1)	C(7)	C(12)	-135.00(16)
C(1)	S(1)	C(13)	C(14)	-62.88(14)
C(1)	S(1)	C(13)	B(1)	170.22(12)
C(1)	C(2)	C(3)	C(4)	0.7(3)
C(2)	S(2)	C(8)	C(7)	-52.53(16)
C(2)	S(2)	C(8)	C(9)	131.40(16)
C(2)	S(2)	C(14)	C(13)	37.54(16)
C(2)	C(1)	C(6)	C(5)	-0.2(3)
C(2)	C(3)	C(4)	C(5)	0.0(3)
C(3)	C(4)	C(5)	C(6)	-0.9(3)
C(4)	C(5)	C(6)	C(1)	0.9(3)
C(6)	C(1)	C(2)	S(2)	-179.38(14)
C(6)	C(1)	C(2)	C(3)	-0.7(3)
C(7)	S(1)	C(1)	C(2)	-49.30(17)
C(7)	S(1)	C(1)	C(6)	132.94(16)
C(7)	S(1)	C(13)	C(14)	39.60(15)
C(7)	S(1)	C(13)	B(1)	-87.31(13)
C(7)	C(8)	C(9)	C(10)	-1.0(3)
C(8)	S(2)	C(2)	C(1)	47.45(17)
C(8)	S(2)	C(2)	C(3)	-131.30(16)
C(8)	S(2)	C(14)	C(13)	-64.01(15)
C(8)	C(7)	C(12)	C(11)	-0.9(3)
C(8)	C(9)	C(10)	C(11)	-0.5(3)
C(9)	C(10)	C(11)	C(12)	1.3(4)
C(10)	C(11)	C(12)	C(7)	-0.6(3)
C(12)	C(7)	C(8)	S(2)	-174.38(15)
C(12)	C(7)	C(8)	C(9)	1.7(3)
C(13)	S(1)	C(1)	C(2)	53.44(16)
C(13)	S(1)	C(1)	C(6)	-124.32(16)
C(13)	S(1)	C(7)	C(8)	-56.14(16)
C(13)	S(1)	C(7)	C(12)	124.86(16)
C(14)	S(2)	C(2)	C(1)	-51.47(17)
C(14)	S(2)	C(2)	C(3)	129.78(16)
C(14)	S(2)	C(8)	C(7)	51.12(16)
C(14)	S(2)	C(8)	C(9)	-124.95(16)
C(14)	C(13)	B(1)	O(1)	-12.5(2)
C(14)	C(13)	B(1)	O(3)	-137.86(16)
C(14)	C(13)	B(1)	N(1)	104.84(17)
C(15)	O(1)	B(1)	O(3)	-108.59(17)
C(15)	O(1)	B(1)	N(1)	2.30(19)
C(15)	O(1)	B(1)	C(13)	126.83(16)
C(16)	N(1)	C(18)	C(17)	-105.96(16)
C(16)	N(1)	B(1)	O(1)	-5.71(17)
C(16)	N(1)	B(1)	O(3)	111.57(15)
C(16)	N(1)	B(1)	C(13)	-127.02(16)
C(17)	O(3)	B(1)	O(1)	117.68(16)
C(17)	O(3)	B(1)	N(1)	6.70(19)
C(17)	O(3)	B(1)	C(13)	-117.82(16)
C(18)	N(1)	C(16)	C(15)	118.64(16)
C(18)	N(1)	B(1)	O(1)	-124.53(14)

Atom	Atom	Atom	Atom	Angle/°
C(18)	N(1)	B(1)	O(3)	-7.26(17)
C(18)	N(1)	B(1)	C(13)	114.15(16)
C(19)	N(1)	C(16)	C(15)	-117.89(16)
C(19)	N(1)	C(18)	C(17)	131.08(15)
C(19)	N(1)	B(1)	O(1)	114.18(16)
C(19)	N(1)	B(1)	O(3)	-128.55(15)
C(19)	N(1)	B(1)	C(13)	-7.1(2)
B(1)	O(1)	C(15)	O(2)	-176.69(18)
B(1)	O(1)	C(15)	C(16)	2.2(2)
B(1)	O(3)	C(17)	O(4)	174.36(18)
B(1)	O(3)	C(17)	C(18)	-3.4(2)
B(1)	N(1)	C(16)	C(15)	6.92(18)
B(1)	N(1)	C(18)	C(17)	5.58(17)
B(1)	C(13)	C(14)	S(2)	142.10(13)
O(2C)	S(1C)	C(8C)	F(5C)	-175.06(15)
O(2C)	S(1C)	C(8C)	F(6C)	-55.12(17)
O(2C)	S(1C)	C(8C)	F(7C)	63.95(18)
O(3C)	S(1C)	C(8C)	F(5C)	65.11(17)
O(3C)	S(1C)	C(8C)	F(6C)	-174.95(14)
O(3C)	S(1C)	C(8C)	F(7C)	-55.88(18)
O(4C)	S(1C)	C(8C)	F(5C)	-54.84(18)
O(4C)	S(1C)	C(8C)	F(6C)	65.10(17)
O(4C)	S(1C)	C(8C)	F(7C)	-175.83(16)
O(10C)	S(9C)	C(16C)	F(13C)	62.0(2)
O(10C)	S(9C)	C(16C)	F(14C)	-56.4(2)
O(10C)	S(9C)	C(16C)	F(15C)	-177.3(3)
O(11C)	S(9C)	C(16C)	F(13C)	-177.0(2)
O(11C)	S(9C)	C(16C)	F(14C)	64.7(2)
O(11C)	S(9C)	C(16C)	F(15C)	-56.2(3)
O(12C)	S(9C)	C(16C)	F(13C)	-57.9(2)
O(12C)	S(9C)	C(16C)	F(14C)	-176.2(2)
O(12C)	S(9C)	C(16C)	F(15C)	62.9(3)
S(6)	C(1A)	F(4)	F(4)[1]	-132.0(14)
F(3)	C(1A)	S(6)	O(7)	-62.5
F(3)	C(1A)	S(6)	O(8)	57.8
F(3)	C(1A)	S(6)	O(9)	177.5
F(3)	C(1A)	F(4)	F(4)[1]	106.5(14)
F(4)	C(1A)	S(6)	O(7)	178.2
F(4)	C(1A)	S(6)	O(8)	-61.5
F(4)	C(1A)	S(6)	O(9)	58.2
F(5)	C(1A)	S(6)	O(7)	58.3
F(5)	C(1A)	S(6)	O(8)	178.6
F(5)	C(1A)	S(6)	O(9)	-61.7
F(5)	C(1A)	F(4)	F(4)[1]	-10.1(14)

[1]1-x,2-y,1-z

Table S5: Hydrogen Fractional Atomic Coordinates (×10[4]) and Equivalent Isotropic Displacement Parameters (Å[2]×10[3]) for **3c** U_{eq} is defined as 1/3 of the trace of the orthogonalised U_{ij}.

**Atom**	**x**	**y**	**z**	U_{eq}
H(3)	-811.68	7126.16	4180.26	34
H(4)	-961.31	6590.62	5925.94	37
H(5)	960.31	5794.94	6496.23	36
H(6)	3074.24	5465.62	5333.48	32
H(9)	803.6	5370.18	1275.53	34
H(10)	2012.22	3699.3	743.02	44
H(11)	4067.56	3044.63	1183.04	46
H(12)	4924.67	3997.87	2220.67	36
H(13)	4089.88	8038.14	2928.64	25
H(14A)	3318.9	7924.49	1190.89	28
H(14B)	2692.78	8748.04	1990.42	28
H(16A)	6760.81	10348.17	521.76	30
H(16B)	8055.99	9444.87	292.24	30
H(18A)	7769.39	8392.04	2723.39	28
H(18B)	8716.23	8547.55	1572.59	28
H(19A)	6493.65	10322.66	2260.43	38
H(19B)	5579.76	9230.58	3032.34	38
H(19C)	5040.26	9995.52	2101.59	38

Table S6: Atomic Occupancies for all atoms that are not fully occupied in **3c**.

**Atom**	**Occupancy**	**Atom**	**Occupancy**	**Atom**	**Occupancy**
S(9C)	0.8485(19)	O(12C)	0.8485(19)	F(5)	0.1515(19)
F(13C)	0.8485(19)	C(16C)	0.8485(19)	O(7)	0.1515(19)
F(14C)	0.8485(19)	C(1A)	0.1515(19)	O(8)	0.1515(19)
F(15C)	0.8485(19)	S(6)	0.1515(19)	O(9)	0.1515(19)
O(10C)	0.8485(19)	F(3)	0.1515(19)		
O(11C)	0.8485(19)	F(4)	0.1515(19)		

Table S7: Solvent masking (PLATON/SQUEEZE) information for **3c**

**No**	**x**	**y**	**z**	**V**	**e**	**Content**
1	0.000	0.000	0.500	175.4	49.3	2acetonitrile

Experimental

Compound **30** was crystallized from acetonitrile. The crystallographic data are summarized in the following tables.

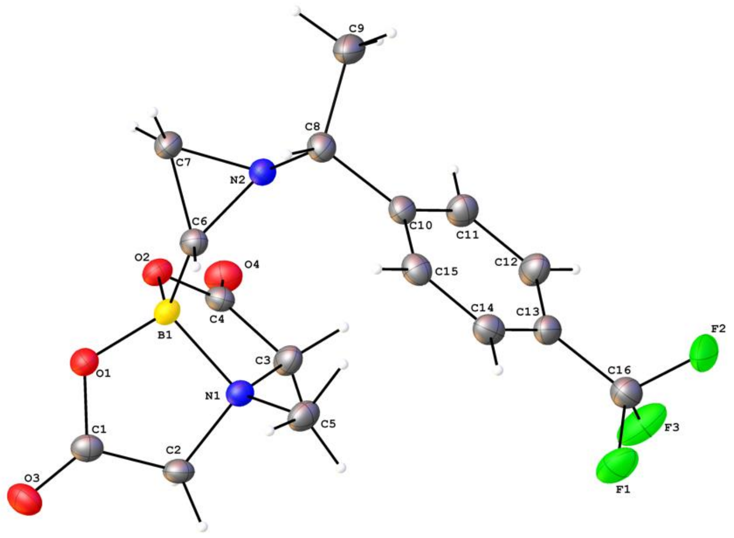

Figure S5: X-ray structure of compound **30**.

Crystal data and structure refinement

**Compound**	**30**
Formula	$C_{16}H_{18}BF_3N_2O_4$
$D_{calc.}$/ g cm^{-3}	1.451
μ/mm^{-1}	1.074
Formula Weight	370.13
Color	yellow
Shape	block-shaped
Size/mm^3	0.20×0.10×0.06
T/K	104(6)
Crystal System	triclinic
Flack Parameter	-0.03(4)
Hooft Parameter	-0.02(3)
Space Group	P1
a/Å	7.56920(10)
b/Å	7.59130(10)
c/Å	14.9391(2)
α/°	95.8240(10)
β/°	96.5830(10)
γ/°	92.281(2)
V/Å^3	847.19(2)
Z	2
Z'	2
Wavelength/Å	1.54184
Radiation type	Cu K$_\alpha$
Θ_{min}/°	2.995
Θ_{max}/°	74.455
Measured Refl's.	29818
Indep't Refl's	6144
Refl's I≥2 σ(I)	6035
R_{int}	0.0305
Parameters	498
Restraints	199
Largest Peak	0.186
Deepest Hole	-0.180
GooF	1.036
wR_2 (all data)	0.0660
wR_2	0.0657
R_1 (all data)	0.0255
R_1	0.0250

Structure Quality Indicators

Reflections: d min (CuKα) 2Θ=148.9° 0.80 | I/σ(I) 55.4 | Rint m=4.85 3.05% | Full 135.4° 99% to 148.9° 99.8

Refinement: Shift 0.000 | Max Peak 0.2 | Min Peak -0.2 | GooF 1.036 | Hooft -.02(3)

Bond lengths [Å] and angles [°] (45):

Table S8: Fractional Atomic Coordinates (×10^4) and Equivalent Isotropic Displacement Parameters (Å^2×10^3) for **30**. U_{eq} is defined as 1/3 of the trace of the orthogonalized U_{ij}.

Atom	x	y	z	U_{eq}
F1B	131.2(17)	-534.6(16)	6692.2(10)	33.2(3)
F2B	-853.4(19)	377(2)	5427.5(10)	40.5(3)
F3B	-1816.4(18)	1438.5(17)	6650.5(12)	39.8(4)

Atom	x	y	z	U_{eq}
O1B	5336.4(17)	8221.2(18)	9992.4(9)	20.5(3)
O2B	3304.0(17)	9821.4(17)	9039.2(9)	19.2(3)
O3B	5045.4(19)	7637(2)	11411.9(10)	26.3(3)
O4B	530.6(18)	10757.7(19)	8836.2(10)	25.9(3)
N1B	2574(2)	6766(2)	9218.7(11)	18.2(3)
N2B	4638(2)	7949(2)	7359.9(11)	19.8(3)
C1B	4454(2)	7605(2)	10628.2(14)	20.3(4)
C2B	2620(2)	6846(3)	10228.8(13)	19.4(4)
C3B	994(2)	7617(3)	8791.1(14)	20.6(4)
C4B	1541(2)	9584(3)	8891.3(13)	18.7(4)
C5B	2702(3)	4902(3)	8820.6(14)	23.2(4)
C6B	5374(2)	7649(3)	8300.7(13)	19.1(4)
C7B	6173(3)	9059(3)	7812.6(13)	21.6(4)
C8B	5110(3)	6590(3)	6653.3(14)	24.0(4)
C9B	5196(3)	7423(3)	5771.0(15)	34.5(5)
C10B	3717(3)	5067(3)	6556.1(13)	21.4(4)
C11B	1928(3)	5407(3)	6328.2(14)	23.4(4)
C12B	622(3)	4058(3)	6261.8(14)	22.7(4)
C13B	1091(3)	2358(3)	6427.4(13)	19.9(4)
C14B	2860(3)	2001(3)	6659.9(14)	22.8(4)
C15B	4170(3)	3371(3)	6722.0(14)	22.9(4)
C16B	-344(3)	925(3)	6315.0(15)	24.1(4)
B1B	4250(3)	8148(3)	9104.4(15)	18.6(4)
O1	8716.9(18)	4826.8(17)	5.5(10)	20.9(3)
O2	10770.8(16)	3224.9(17)	946.5(10)	19.8(3)
O3	7489(2)	3888(2)	-1410.8(10)	27.1(3)
O4	11813.9(19)	547.3(19)	1136.0(11)	26.6(3)
N1	7631(2)	2412(2)	789.8(11)	17.8(3)
N2	9648(2)	5270(2)	2634.0(11)	19.4(3)
C1	7808(2)	3651(3)	-626.3(14)	20.3(4)
C2	7248(2)	2008(3)	-219.4(13)	20.2(4)
C3	8693(3)	1031(3)	1211.7(14)	22.0(4)
C4	10610(2)	1539(3)	1092.5(13)	19.9(4)
C5	5954(2)	2710(3)	1202.5(14)	23.5(4)
C6	8938(2)	5614(2)	1698.6(14)	19.0(4)
C7	10590(3)	6588(3)	2189.7(13)	21.7(4)
C8	8638(3)	6069(3)	3352.5(14)	22.0(4)
C9	9914(3)	6526(3)	4217.8(15)	29.9(5)
C10	7142(3)	4765(3)	3474.5(13)	20.4(4)
C11	7540(3)	3111(3)	3760.7(15)	25.2(4)
C12	6194(3)	1873(3)	3843.2(15)	25.6(4)
C13	4417(3)	2271(3)	3631.9(14)	22.1(4)
C14	4005(3)	3909(3)	3354.5(14)	22.8(4)
C15	5367(3)	5151(3)	3279.2(14)	22.3(4)
B1	9066(3)	4137(3)	892.6(15)	18.2(4)
F1	1429(4)	1132(5)	3249(3)	36.9(6)
F2	2665(5)	891(7)	4594.4(16)	56.7(12)
F3	3421(3)	-755(3)	3472(3)	43.4(8)
C16	3002(5)	898(5)	3732(3)	26.5(6)
F1A	1690(30)	930(40)	3082(16)	36.9(6)
F2A	2070(20)	1760(30)	4464(11)	44(4)
F3A	3460(20)	-440(30)	3910(20)	47(4)
C16A	2920(30)	1120(30)	3783(19)	26.5(6)

Table S9: Anisotropic Displacement Parameters (×10[4]) for **30.** The anisotropic displacement factor exponent takes the form: $-2\pi^2[h^2a^{*2} \times U_{11} + ... +2hka^* \times b^* \times U_{12}]$

Atom	U_{11}	U_{22}	U_{33}	U_{23}	U_{13}	U_{12}
F1B	32.5(7)	22.1(6)	45.6(8)	9.1(6)	3.6(6)	-0.8(5)
F2B	44.0(8)	42.4(8)	30.5(7)	0.1(6)	-4.0(6)	-20.0(6)
F3B	27.5(6)	28.2(7)	67.9(10)	7.2(7)	21.6(6)	1.1(5)
O1B	18.0(6)	24.1(7)	19.3(7)	3.5(5)	1.6(5)	-2.4(5)
O2B	18.4(6)	17.2(6)	22.0(7)	2.3(5)	3.2(5)	-1.6(5)
O3B	24.8(7)	34.0(8)	20.1(8)	6.4(6)	-0.5(6)	3.0(6)
O4B	24.2(7)	24.9(7)	29.2(8)	5.1(6)	2.8(6)	5.8(6)
N1B	17.9(7)	17.7(7)	19.1(8)	1.7(6)	3.2(6)	0.0(6)
N2B	20.1(8)	20.6(8)	18.0(8)	1.6(6)	1.3(6)	-3.7(6)
C1B	21.0(9)	18.2(9)	22.4(10)	3.4(7)	4.0(7)	3.4(7)
C2B	20.3(9)	21.9(9)	16.7(9)	3.5(7)	4.0(7)	1.7(7)
C3B	17.3(8)	21.4(9)	22.9(10)	4.6(8)	0.1(7)	-1.6(7)
C4B	19.5(9)	21.4(9)	15.4(9)	2.9(7)	2.6(7)	0.0(7)
C5B	26.0(10)	18.4(9)	25.2(10)	-0.3(8)	7.2(8)	-1.8(7)
C6B	17.5(8)	20.1(9)	19.3(9)	2.6(7)	1.0(7)	-1.4(7)
C7B	20.8(9)	24.3(10)	19.0(9)	1.6(8)	3.1(7)	-5.5(7)
C8B	22.8(9)	26.6(10)	21.6(10)	-1.9(8)	4.0(8)	-4.4(8)
C9B	42.9(13)	37.6(13)	21.5(11)	-1.9(9)	7.4(9)	-14.8(10)
C10B	23.4(9)	23.5(9)	16.5(9)	-1.9(8)	3.6(7)	-1.1(8)
C11B	26.2(10)	21.1(9)	22.6(10)	3.8(8)	0.3(8)	1.1(8)
C12B	20.7(9)	23.9(10)	22.7(10)	1.8(8)	-0.3(8)	2.1(7)
C13B	22.8(9)	20.3(9)	16.5(9)	0.1(7)	3.7(7)	0.0(7)
C14B	25.5(10)	20.8(9)	22.2(10)	0.3(8)	3.9(8)	4.1(8)
C15B	19.3(9)	27.5(10)	21.1(10)	-1.4(8)	2.7(7)	2.3(7)
C16B	23.6(9)	22.8(10)	26.5(11)	3.5(8)	4.5(8)	2.7(8)
B1B	16.9(9)	17.2(10)	21.2(11)	2.7(8)	1.4(8)	-3.4(8)
O1	21.9(6)	20.2(6)	20.0(7)	2.3(5)	1.1(5)	-2.9(5)
O2	16.4(6)	19.9(6)	22.5(7)	0.7(5)	2.3(5)	-1.0(5)
O3	31.3(8)	28.0(7)	21.1(8)	4.3(6)	-2.5(6)	3.5(6)
O4	22.5(7)	25.1(7)	31.5(8)	1.5(6)	0.0(6)	4.8(6)
N1	15.5(7)	19.2(7)	18.3(8)	0.6(6)	2.1(6)	-0.1(6)
N2	18.6(7)	21.7(8)	17.5(8)	1.7(7)	2.2(6)	-2.4(6)
C1	16.4(8)	21.9(9)	21.8(10)	0.0(8)	0.6(7)	2.6(7)
C2	18.5(9)	22.3(9)	18.1(10)	-1.8(7)	-0.7(7)	-1.5(7)
C3	20.2(9)	20.6(9)	25.6(10)	6.4(8)	1.0(8)	-1.0(7)
C4	20.3(9)	21.3(9)	16.8(9)	1.3(7)	-1.2(7)	-1.3(7)
C5	18.2(9)	25.1(10)	26.9(10)	-1.3(8)	6.6(8)	-2.1(7)
C6	18.7(9)	19.0(9)	19.3(9)	3.0(7)	1.1(7)	0.1(7)
C7	22.1(9)	23.0(9)	19.3(9)	0.8(8)	3.1(7)	-4.8(7)
C8	24.5(10)	22.2(10)	19.3(10)	0.5(8)	4.6(8)	-1.0(7)
C9	30.5(11)	35.5(12)	22.1(11)	-1.5(9)	4.3(9)	-10.6(9)
C10	22.0(9)	22.9(9)	16.3(9)	0.7(7)	4.1(7)	-1.1(7)
C11	19.5(9)	29.0(10)	27.5(11)	5.4(8)	3.0(8)	1.3(8)
C12	23.1(10)	24.5(10)	29.6(11)	7.9(8)	0.9(8)	-0.8(8)
C13	20.8(9)	26.9(10)	18.7(9)	1.9(8)	4.3(7)	-2.0(8)
C14	18.0(8)	29.1(10)	21.5(10)	0.5(8)	3.9(7)	3.4(8)
C15	26.2(10)	22.2(9)	19.0(9)	1.0(8)	4.9(8)	4.3(8)
B1	15.6(9)	19.2(10)	19.6(10)	2.9(8)	1.9(8)	-2.7(8)
F1	19.9(11)	37.9(12)	51.7(16)	6.7(10)	-0.9(9)	-4.2(8)
F2	64.6(18)	78(2)	24.3(10)	1.2(11)	13.7(10)	-44.4(18)
F3	28.3(9)	24.4(9)	76(2)	-1.8(11)	7.4(12)	-3.7(7)
C16	24.8(11)	31.3(16)	22.9(12)	2.4(11)	2.6(8)	-1.0(10)
F1A	19.9(11)	37.9(12)	51.7(16)	6.7(10)	-0.9(9)	-4.2(8)
F2A	45(7)	59(9)	29(6)	8(6)	15(5)	-23(6)
F3A	38(6)	29(7)	75(11)	26(7)	-5(7)	-14(5)
C16A	24.8(11)	31.3(16)	22.9(12)	2.4(11)	2.6(8)	-1.0(10)

Table S10: Bond Lengths in Å for **30**.

Atom	Atom	Length/Å
F1B	C16B	1.337(3)
F2B	C16B	1.356(3)
F3B	C16B	1.330(2)
O1B	C1B	1.333(2)
O1B	B1B	1.473(3)
O2B	C4B	1.330(2)
O2B	B1B	1.489(3)
O3B	C1B	1.202(2)
O4B	C4B	1.200(2)
N1B	C2B	1.500(2)
N1B	C3B	1.492(2)
N1B	C5B	1.489(2)
N1B	B1B	1.649(3)
N2B	C6B	1.494(3)
N2B	C7B	1.464(2)
N2B	C8B	1.484(2)
C1B	C2B	1.514(3)
C3B	C4B	1.521(3)
C6B	C7B	1.501(3)
C6B	B1B	1.574(3)
C8B	C9B	1.525(3)
C8B	C10B	1.518(3)
C10B	C11B	1.399(3)
C10B	C15B	1.384(3)
C11B	C12B	1.383(3)
C12B	C13B	1.391(3)
C13B	C14B	1.388(3)
C13B	C16B	1.489(3)
C14B	C15B	1.397(3)
O1	C1	1.339(2)
O1	B1	1.474(3)
O2	C4	1.323(2)
O2	B1	1.486(2)
O3	C1	1.202(3)
O4	C4	1.205(2)
N1	C2	1.500(2)
N1	C3	1.493(3)
N1	C5	1.489(2)
N1	B1	1.651(2)
N2	C6	1.491(3)
N2	C7	1.461(3)
N2	C8	1.483(2)
C1	C2	1.508(3)
C3	C4	1.521(3)
C6	C7	1.501(2)
C6	B1	1.574(3)
C8	C9	1.523(3)
C8	C10	1.513(3)
C10	C11	1.400(3)
C10	C15	1.391(3)
C11	C12	1.383(3)
C12	C13	1.398(3)
C13	C14	1.386(3)
C13	C16	1.495(4)
C13	C16A	1.46(2)
C14	C15	1.391(3)
F1	C16	1.347(4)
F2	C16	1.343(4)
F3	C16	1.337(4)
F1A	C16A	1.31(2)
F2A	C16A	1.32(2)
F3A	C16A	1.30(2)

Table S11: Bond Angles in ° for **30**.

Atom	Atom	Atom	Angle/°
C1B	O1B	B1B	113.12(15)
C4B	O2B	B1B	113.95(14)
C2B	N1B	B1B	102.42(14)
C3B	N1B	C2B	112.31(14)
C3B	N1B	B1B	103.24(15)
C5B	N1B	C2B	110.22(16)
C5B	N1B	C3B	112.42(15)
C5B	N1B	B1B	115.72(15)
C7B	N2B	C6B	60.94(12)
C7B	N2B	C8B	113.25(15)
C8B	N2B	C6B	113.68(16)
O1B	C1B	C2B	110.94(16)
O3B	C1B	O1B	124.38(18)
O3B	C1B	C2B	124.68(19)
N1B	C2B	C1B	106.51(15)
N1B	C3B	C4B	104.62(15)
O2B	C4B	C3B	110.34(16)
O4B	C4B	O2B	124.60(18)
O4B	C4B	C3B	125.06(17)
N2B	C6B	C7B	58.54(12)
N2B	C6B	B1B	119.13(16)
C7B	C6B	B1B	120.94(17)
N2B	C7B	C6B	60.52(12)
N2B	C8B	C9B	109.31(18)
N2B	C8B	C10B	107.61(16)
C10B	C8B	C9B	112.39(17)
C11B	C10B	C8B	118.97(18)
C15B	C10B	C8B	121.51(18)
C15B	C10B	C11B	119.47(18)
C12B	C11B	C10B	120.34(19)
C11B	C12B	C13B	119.73(19)
C12B	C13B	C16B	118.18(18)
C14B	C13B	C12B	120.64(18)
C14B	C13B	C16B	121.15(18)
C13B	C14B	C15B	119.17(19)
C10B	C15B	C14B	120.64(18)
F1B	C16B	F2B	105.40(16)
F1B	C16B	C13B	113.95(17)
F2B	C16B	C13B	111.32(18)
F3B	C16B	F1B	106.97(18)
F3B	C16B	F2B	105.53(18)
F3B	C16B	C13B	113.03(16)
O1B	B1B	O2B	109.72(15)
O1B	B1B	N1B	102.37(16)

Atom	Atom	Atom	Angle/°
O1B	B1B	C6B	111.82(16)
O2B	B1B	N1B	100.32(14)
O2B	B1B	C6B	114.33(17)
C6B	B1B	N1B	117.12(15)
C1	O1	B1	112.89(15)
C4	O2	B1	114.12(15)
C2	N1	B1	102.14(14)
C3	N1	C2	112.15(15)
C3	N1	B1	103.12(14)
C5	N1	C2	110.71(15)
C5	N1	C3	112.30(16)
C5	N1	B1	115.85(14)
C7	N2	C6	61.13(12)
C7	N2	C8	113.15(15)
C8	N2	C6	114.28(15)
O1	C1	C2	110.87(17)
O3	C1	O1	124.02(19)
O3	C1	C2	125.11(17)
N1	C2	C1	106.86(14)
N1	C3	C4	104.42(16)
O2	C4	C3	110.48(16)
O4	C4	O2	124.89(18)
O4	C4	C3	124.62(18)
N2	C6	C7	58.47(12)
N2	C6	B1	118.73(16)
C7	C6	B1	120.52(16)
N2	C7	C6	60.40(12)
N2	C8	C9	108.69(16)
N2	C8	C10	108.44(15)
C10	C8	C9	112.35(18)
C11	C10	C8	119.74(18)
C15	C10	C8	121.31(19)
C15	C10	C11	118.91(18)
C12	C11	C10	120.72(19)
C11	C12	C13	119.7(2)
C12	C13	C16	118.0(2)
C12	C13	C16A	123.3(12)
C14	C13	C12	120.19(19)
C14	C13	C16	121.8(2)
C14	C13	C16A	116.1(11)
C13	C14	C15	119.73(18)
C10	C15	C14	120.78(19)
O1	B1	O2	109.85(16)
O1	B1	N1	102.42(14)
O1	B1	C6	111.80(16)
O2	B1	N1	100.27(14)
O2	B1	C6	114.40(16)
C6	B1	N1	116.95(16)
F1	C16	C13	113.2(3)
F2	C16	C13	112.2(3)
F2	C16	F1	105.8(3)
F3	C16	C13	113.5(3)
F3	C16	F1	105.9(3)
F3	C16	F2	105.5(3)
F1A	C16A	C13	112(2)
F1A	C16A	F2A	104(2)
F2A	C16A	C13	113.4(18)
F3A	C16A	C13	109.6(19)
F3A	C16A	F1A	107(2)
F3A	C16A	F2A	110(2)

Table S12: Torsion Angles in ° for **30**.

Atom	Atom	Atom	Atom	Angle/°
O1B	C1B	C2B	N1B	9.9(2)
O3B	C1B	C2B	N1B	-169.48(17)
N1B	C3B	C4B	O2B	19.6(2)
N1B	C3B	C4B	O4B	-161.04(19)
N2B	C6B	B1B	O1B	-164.87(15)
N2B	C6B	B1B	O2B	-39.4(2)
N2B	C6B	B1B	N1B	77.5(2)
N2B	C8B	C10B	C11B	58.2(2)
N2B	C8B	C10B	C15B	-119.3(2)
C1B	O1B	B1B	O2B	89.38(19)
C1B	O1B	B1B	N1B	-16.49(19)
C1B	O1B	B1B	C6B	-142.67(16)
C2B	N1B	C3B	C4B	83.12(18)
C2B	N1B	B1B	O1B	20.93(17)
C2B	N1B	B1B	O2B	-92.09(16)
C2B	N1B	B1B	C6B	143.60(17)
C3B	N1B	C2B	C1B	-128.67(16)
C3B	N1B	B1B	O1B	137.75(15)
C3B	N1B	B1B	O2B	24.73(17)
C3B	N1B	B1B	C6B	-99.58(19)
C4B	O2B	B1B	O1B	-121.25(17)
C4B	O2B	B1B	N1B	-14.0(2)
C4B	O2B	B1B	C6B	112.21(18)
C5B	N1B	C2B	C1B	105.14(17)
C5B	N1B	C3B	C4B	-151.89(16)

Atom	Atom	Atom	Atom	Angle/°
C5B	N1B	B1B	O1B	-99.00(18)
C5B	N1B	B1B	O2B	147.98(16)
C5B	N1B	B1B	C6B	23.7(2)
C6B	N2B	C8B	C9B	-150.69(17)
C6B	N2B	C8B	C10B	86.99(19)
C7B	N2B	C6B	B1B	110.43(19)
C7B	N2B	C8B	C9B	-83.6(2)
C7B	N2B	C8B	C10B	154.11(17)
C7B	C6B	B1B	O1B	-96.1(2)
C7B	C6B	B1B	O2B	29.3(2)
C7B	C6B	B1B	N1B	146.21(17)
C8B	N2B	C6B	C7B	104.45(17)
C8B	N2B	C6B	B1B	-145.12(17)
C8B	N2B	C7B	C6B	-105.15(18)
C8B	C10B	C11B	C12B	-178.02(19)
C8B	C10B	C15B	C14B	177.73(18)
C9B	C8B	C10B	C11B	-62.2(2)
C9B	C8B	C10B	C15B	120.3(2)
C10B	C11B	C12B	C13B	0.3(3)
C11B	C10B	C15B	C14B	0.3(3)
C11B	C12B	C13B	C14B	0.1(3)
C11B	C12B	C13B	C16B	-177.71(19)
C12B	C13B	C14B	C15B	-0.3(3)
C12B	C13B	C16B	F1B	-164.56(17)
C12B	C13B	C16B	F2B	76.4(2)
C12B	C13B	C16B	F3B	-42.1(3)
C13B	C14B	C15B	C10B	0.1(3)
C14B	C13B	C16B	F1B	17.7(3)
C14B	C13B	C16B	F2B	-101.3(2)
C14B	C13B	C16B	F3B	140.1(2)
C15B	C10B	C11B	C12B	-0.5(3)
C16B	C13B	C14B	C15B	177.42(19)
B1B	O1B	C1B	O3B	-175.39(18)
B1B	O1B	C1B	C2B	5.2(2)
B1B	O2B	C4B	O4B	178.33(19)
B1B	O2B	C4B	C3B	-2.4(2)
B1B	N1B	C2B	C1B	-18.55(18)
B1B	N1B	C3B	C4B	-26.49(18)
B1B	C6B	C7B	N2B	-107.37(19)
O1	C1	C2	N1	10.0(2)
O3	C1	C2	N1	-170.02(17)
N1	C3	C4	O2	20.1(2)
N1	C3	C4	O4	-160.76(18)
N2	C6	B1	O1	-166.27(15)
N2	C6	B1	O2	-40.6(2)
N2	C6	B1	N1	76.1(2)
N2	C8	C10	C11	62.3(2)
N2	C8	C10	C15	-115.5(2)
C1	O1	B1	O2	88.97(18)
C1	O1	B1	N1	-16.90(19)
C1	O1	B1	C6	-142.91(16)
C2	N1	C3	C4	82.56(17)
C2	N1	B1	O1	21.32(17)
C2	N1	B1	O2	-91.84(16)
C2	N1	B1	C6	143.90(17)
C3	N1	C2	C1	-128.62(16)
C3	N1	B1	O1	137.82(15)
C3	N1	B1	O2	24.66(17)
C3	N1	B1	C6	-99.60(19)
C4	O2	B1	O1	-121.02(17)
C4	O2	B1	N1	-13.70(19)

Atom	Atom	Atom	Atom	Angle/˚
C4	O2	B1	C6	112.31(19)
C5	N1	C2	C1	105.08(17)
C5	N1	C3	C4	-152.01(15)
C5	N1	B1	O1	-99.10(18)
C5	N1	B1	O2	147.74(16)
C5	N1	B1	C6	23.5(2)
C6	N2	C8	C9	-149.48(17)
C6	N2	C8	C10	88.1(2)
C7	N2	C6	B1	110.06(19)
C7	N2	C8	C9	-82.0(2)
C7	N2	C8	C10	155.58(16)
C7	C6	B1	O1	-97.9(2)
C7	C6	B1	O2	27.7(3)
C7	C6	B1	N1	144.48(18)
C8	N2	C6	C7	104.11(17)
C8	N2	C6	B1	-145.83(17)
C8	N2	C7	C6	-105.96(17)
C8	C10	C11	C12	-177.50(19)
C8	C10	C15	C14	177.04(18)
C9	C8	C10	C11	-57.8(2)
C9	C8	C10	C15	124.3(2)
C10	C11	C12	C13	0.5(3)
C11	C10	C15	C14	-0.8(3)
C11	C12	C13	C14	-1.0(3)
C11	C12	C13	C16	-179.9(2)
C11	C12	C13	C16A	-173.7(13)
C12	C13	C14	C15	0.6(3)
C12	C13	C16	F1	-159.5(3)
C12	C13	C16	F2	80.7(4)
C12	C13	C16	F3	-38.7(4)
C12	C13	C16A	F1A	-134.6(19)
C12	C13	C16A	F2A	107.8(19)
C12	C13	C16A	F3A	-16(3)
C13	C14	C15	C10	0.4(3)
C14	C13	C16	F1	21.6(4)
C14	C13	C16	F2	-98.1(4)
C14	C13	C16	F3	142.4(3)
C14	C13	C16A	F1A	52(2)
C14	C13	C16A	F2A	-65(2)
C14	C13	C16A	F3A	171.1(18)
C15	C10	C11	C12	0.4(3)
B1	O1	C1	O3	-174.51(18)
B1	O1	C1	C2	5.5(2)
B1	O2	C4	O4	178.00(18)
B1	O2	C4	C3	-2.8(2)
B1	N1	C2	C1	-18.85(18)
B1	N1	C3	C4	-26.60(18)
B1	C6	C7	N2	-107.0(2)
C16	C13	C14	C15	179.4(2)
C16A	C13	C14	C15	173.8(12)

Table S13: Hydrogen Fractional Atomic Coordinates (×10[4]) and Equivalent Isotropic Displacement Parameters (Å^2×10^3) for **30.** U_{eq} is defined as 1/3 of the trace of the orthogonalized U_{ij}.

Atom	x	y	z	U_{eq}
H2BA	2408.8	5645.79	10412.18	23
H2BB	1691.51	7614.17	10440.63	23
H3BA	-62.28	7398.64	9106.31	25
H3BB	714.8	7155.86	8144.17	25

Atom	x	y	z	U_{eq}
H5BA	1685.69	4175.33	8955.02	35
H5BB	3811.45	4435.48	9082.32	35
H5BC	2692.35	4869.47	8162.61	35
H6B	6077.05	6568.63	8351.9	23
H7BA	5976.56	10310.4	8016.92	26
H7BB	7345.9	8856.27	7598.27	26
H8B	6302.89	6147.96	6854.02	29
H9BA	4039.27	7886.87	5579.88	52
H9BB	5490.92	6523.22	5300.56	52
H9BC	6114.13	8392.63	5865.49	52
H11B	1608.2	6570.2	6218.53	28
H12B	-591.83	4291.86	6103.77	27
H14B	3175.71	839.24	6775.24	27
H15B	5383.63	3137.68	6879.68	27
H2A	5962.99	1707.32	-396.79	24
H2B	7927.19	992.05	-432.86	24
H3A	8329.25	-164.72	900.63	26
H3B	8540.99	1042.6	1861.68	26
H5A	5159.98	1641.56	1066.19	35
H5B	5366.33	3710.59	952.36	35
H5C	6231.49	2974.92	1860.65	35
H6	7845.97	6313.39	1649.13	23
H7A	11746.1	6278.56	1983.19	26
H7B	10508.97	7853.04	2410.37	26
H8	8118.79	7180.84	3158.57	26
H9A	10504.99	5459.09	4381.98	45
H9B	9247.54	6982.06	4709.18	45
H9C	10810.26	7430.82	4118.84	45
H11	8747.72	2836.76	3899.88	30
H12	6476.02	756.92	4042.46	31
H14	2796.7	4182.45	3216.26	27
H15	5081.8	6274.93	3092.24	27

Experimental

Compound **46** was crystallized from dichloromethane/Et_2O. The crystallographic data are summarized in the following tables.

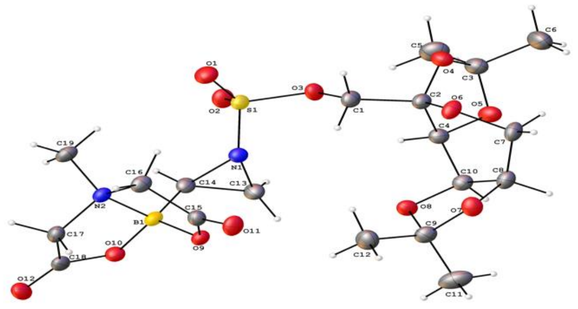

Figure S6: X-ray structure of compound **46**.

Crystal data and structure refinement

**Compound**	**46**
Formula	$C_{19}H_{29}BN_2O_{12}S$
$D_{calc.}$/ g cm^{-3}	1.440
μ/mm^{-1}	1.788
Formula Weight	520.31
Colour	colourless
Shape	plate-shaped
Size/mm^3	0.16×0.07×0.01
T/K	100.01(10)
Crystal System	monoclinic
Flack Parameter	0.013(11)
Hooft Parameter	0.020(5)
Space Group	$P2_1$
a/Å	10.02940(10)
b/Å	6.24760(10)
c/Å	19.1570(3)
α/°	90
β/°	90.7440(10)
γ/°	90
V/Å^3	1200.27(3)
Z	2
Z'	1
Wavelength/Å	1.54184
Radiation type	Cu K$_\alpha$
Θ_{min}/°	2.307
Θ_{max}/°	74.468
Measured Refl's.	23814
Indep't Refl's	4898
Refl's I≥2 σ(I)	4763
R_{int}	0.0366

Parameters	321
Restraints	1
Largest Peak	0.241
Deepest Hole	-0.312
GooF	1.055
wR_2 (all data)	0.0757
wR_2	0.0751
R_1 (all data)	0.0304
R_1	0.0294

Reflections: d min (CuKα) 2Θ=148.9° 0.80 | I/σ(I) 36.2 | Rint m=4.86 3.66% | Full 135.4° 100

Refinement: Shift 0.000 | Max Peak 0.2 | Min Peak -0.3 | GooF 1.055 | Hooft .020(5)

Bond lengths [Å] and angles [°] (46):

Table S14: Fractional Atomic Coordinates (×10[4]) and Equivalent Isotropic Displacement Parameters (Å[2]×10[3]) for **46.** U_{eq} is defined as 1/3 of the trace of the orthogonalised U_{ij}.

Atom	x	y	z	U_{eq}
S(1)	3763.0(4)	3129.6(9)	3084.9(3)	19.99(13)
O(1)	3373.8(18)	1055(3)	2852.3(10)	30.7(4)
O(2)	2869.5(15)	4303(3)	3512.3(9)	27.4(4)
O(3)	5086.5(14)	3035(3)	3527.8(8)	23.6(3)
O(4)	7392.1(16)	2077(3)	4339.8(9)	25.6(4)
O(5)	8071.0(17)	5527(3)	4488.7(8)	25.4(4)
O(6)	8562.1(15)	2145(3)	3305.4(9)	24.0(3)
O(7)	9623.2(16)	5311(3)	2373.9(9)	25.3(4)
O(8)	7697.5(15)	6919(3)	2674.3(8)	22.3(3)
O(9)	4597.7(14)	5740(3)	934.1(8)	19.1(3)
O(10)	2447.2(15)	7624(2)	938.6(8)	20.6(3)
O(11)	5984.9(14)	3312(3)	477.8(8)	26.0(4)
O(12)	1033.9(14)	8275(3)	48.6(9)	28.0(4)
N(1)	4251.5(18)	4429(3)	2388.3(10)	19.6(4)
N(2)	2560.2(17)	3762(3)	846.8(10)	18.2(4)
C(1)	6299(2)	2204(4)	3213.9(12)	21.9(5)
C(2)	7461.4(19)	2978(4)	3667.6(11)	20.8(4)
C(3)	7572(2)	3720(4)	4860.8(13)	28.4(5)
C(4)	7524(2)	5401(4)	3804.8(12)	20.2(4)
C(5)	6242(3)	4205(6)	5197.2(15)	38.2(6)
C(6)	8625(3)	3014(6)	5385.4(15)	41.2(7)
C(7)	9794(2)	3270(4)	3425.6(12)	24.7(5)
C(8)	9711(2)	5481(4)	3119.3(12)	22.3(5)
C(9)	8644(2)	6800(4)	2124.4(12)	23.5(5)
C(10)	8414(2)	6672(4)	3320.8(12)	20.3(4)
C(11)	9279(3)	8949(5)	1987.4(16)	38.4(7)
C(12)	7952(3)	5855(5)	1492.9(13)	32.0(6)
C(13)	4352(2)	6780(4)	2461.3(13)	23.2(5)
C(14)	3220(2)	5858(4)	2049.5(12)	20.0(4)
C(15)	4902(2)	3817(4)	692.6(11)	20.0(5)
C(16)	3714(2)	2316(4)	721.2(13)	21.8(5)
C(17)	2018(2)	4746(4)	186.2(12)	22.3(5)
C(18)	1757(2)	7067(4)	364.8(12)	21.6(5)
C(19)	1469(2)	2696(4)	1239.9(12)	23.0(5)
B(1)	3235(2)	5851(4)	1223.7(13)	18.4(5)

Table S15: Anisotropic Displacement Parameters (×10[4]) for **46.** The anisotropic displacement factor exponent takes the form: $-2\pi^2[h^2a^{*2} \times U_{11} + ... +2hka^* \times b^* \times U_{12}]$

Atom	U_{11}	U_{22}	U_{33}	U_{23}	U_{13}	U_{12}
S(1)	14.0(2)	21.9(3)	24.0(2)	2.3(2)	0.09(17)	0.1(2)
O(1)	27.0(9)	24.9(10)	40.0(10)	3.0(7)	-3.0(7)	-3.6(7)
O(2)	16.9(7)	38.4(11)	27.0(8)	0.0(8)	3.9(6)	2.7(7)
O(3)	13.7(6)	33.1(9)	24.0(7)	3.1(8)	0.5(5)	2.7(7)
O(4)	26.4(8)	23.6(9)	26.7(8)	5.0(7)	-3.4(6)	-0.3(7)
O(5)	28.9(8)	26.3(9)	21.0(8)	0.1(7)	-0.3(6)	-3.1(7)
O(6)	14.3(7)	20.7(8)	37.1(9)	-6.1(7)	1.2(6)	1.2(6)
O(7)	19.4(7)	32.7(10)	23.8(8)	-4.7(7)	1.7(6)	3.6(7)
O(8)	16.7(7)	27.8(9)	22.5(8)	3.6(7)	3.4(6)	1.8(6)
O(9)	13.5(7)	20.6(8)	23.4(7)	0.3(6)	1.1(5)	-1.8(6)
O(10)	17.1(7)	18.7(9)	26.1(7)	1.4(6)	0.4(6)	0.8(6)
O(11)	14.4(6)	34.2(10)	29.5(8)	-5.2(8)	4.2(6)	2.1(7)
O(12)	16.0(6)	34.5(10)	33.6(8)	9.1(8)	-0.7(6)	4.4(8)
N(1)	15.8(8)	19.1(10)	23.8(9)	1.4(8)	0.5(7)	1.1(7)
N(2)	13.6(8)	18.9(10)	22.2(9)	-1.1(7)	2.3(6)	-1.5(7)
C(1)	14.6(10)	23.0(11)	28.2(11)	-0.7(10)	1.0(8)	2.4(9)
C(2)	15.2(9)	21.0(11)	26.1(10)	1.2(10)	0.5(7)	0.8(9)
C(3)	30.2(12)	29.3(14)	25.5(12)	4.2(10)	-2.4(9)	-3.3(10)
C(4)	18.5(10)	20.2(12)	21.9(10)	0.2(9)	1.4(8)	1.8(8)
C(5)	39.3(14)	45.1(18)	30.4(13)	0.9(13)	9.1(11)	-3.3(13)
C(6)	43.4(14)	45.7(17)	34.1(13)	10.9(14)	-13.1(11)	-8.8(15)
C(7)	13.5(8)	25.9(12)	34.8(11)	-4.7(11)	-1.4(8)	0.5(10)
C(8)	15.4(9)	25.9(12)	25.7(11)	-5.1(10)	1.2(8)	-1.3(9)
C(9)	20.6(10)	26.2(13)	23.9(11)	-1.4(10)	4.9(9)	-0.4(9)
C(10)	18.9(10)	20.1(11)	22.1(11)	-2.2(9)	1.9(8)	0.3(9)
C(11)	46.9(16)	32.0(15)	36.5(14)	-2.7(12)	16.6(12)	-8.5(12)
C(12)	28.6(12)	42.4(16)	25.0(12)	-0.9(11)	-1.9(9)	-2.9(11)
C(13)	24.6(11)	19.5(12)	25.3(11)	-1.3(9)	-3.5(9)	-1.8(9)
C(14)	15.7(9)	17.2(11)	27.3(11)	0.2(9)	-0.2(8)	2.7(8)
C(15)	15.6(9)	24.4(12)	20.0(10)	0.6(8)	-0.4(8)	0.3(8)
C(16)	15.2(10)	20.9(11)	29.5(11)	-2.8(9)	4.4(8)	2.1(8)
C(17)	15.7(9)	27.3(12)	23.7(11)	0.4(9)	-1.7(8)	-3.9(9)
C(18)	11.8(9)	26.9(12)	26.3(11)	5.7(10)	2.8(8)	-0.9(9)
C(19)	17.0(9)	23.1(13)	29.0(11)	-0.2(9)	5.8(8)	-4.1(8)
B(1)	12.4(10)	16.9(12)	26.0(12)	0.2(10)	1.9(8)	-1.2(9)

Table S16: Bond Lengths in Å for **46.**

Atom	Atom	Length/Å
S(1)	O(1)	1.423(2)
S(1)	O(2)	1.4248(17)
S(1)	O(3)	1.5672(15)
S(1)	N(1)	1.6421(19)
O(3)	C(1)	1.459(2)
O(4)	C(2)	1.408(3)
O(4)	C(3)	1.442(3)
O(5)	C(3)	1.429(3)
O(5)	C(4)	1.416(3)
O(6)	C(2)	1.411(3)
O(6)	C(7)	1.437(3)
O(7)	C(8)	1.433(3)
O(7)	C(9)	1.430(3)
O(8)	C(9)	1.429(3)
O(8)	C(10)	1.432(3)
O(9)	C(15)	1.325(3)

Atom	Atom	Length/Å
O(9)	B(1)	1.483(3)
O(10)	C(18)	1.338(3)
O(10)	B(1)	1.462(3)
O(11)	C(15)	1.208(3)
O(12)	C(18)	1.205(3)
N(1)	C(13)	1.479(3)
N(1)	C(14)	1.508(3)
N(2)	C(16)	1.491(3)
N(2)	C(17)	1.502(3)
N(2)	C(19)	1.493(3)
N(2)	B(1)	1.634(3)
C(1)	C(2)	1.524(3)
C(2)	C(4)	1.537(3)
C(3)	C(5)	1.520(4)
C(3)	C(6)	1.514(4)
C(4)	C(10)	1.520(3)
C(7)	C(8)	1.503(4)

Atom	Atom	Length/Å
C(8)	C(10)	1.552(3)
C(9)	C(11)	1.510(4)
C(9)	C(12)	1.507(3)
C(13)	C(14)	1.490(3)

Atom	Atom	Length/Å
C(14)	B(1)	1.582(3)
C(15)	C(16)	1.517(3)
C(17)	C(18)	1.513(4)

Table S17: Bond Angles in ° for **46.**

Atom	Atom	Atom	Angle/°
O(1)	S(1)	O(2)	118.46(11)
O(1)	S(1)	O(3)	111.22(11)
O(1)	S(1)	N(1)	106.20(11)
O(2)	S(1)	O(3)	104.07(10)
O(2)	S(1)	N(1)	114.15(11)
O(3)	S(1)	N(1)	101.49(9)
C(1)	O(3)	S(1)	119.66(14)
C(2)	O(4)	C(3)	109.93(19)
C(4)	O(5)	C(3)	106.48(19)
C(2)	O(6)	C(7)	114.66(18)
C(9)	O(7)	C(8)	108.53(17)
C(9)	O(8)	C(10)	107.51(16)
C(15)	O(9)	B(1)	112.98(17)
C(18)	O(10)	B(1)	112.38(18)
C(13)	N(1)	S(1)	115.77(16)
C(13)	N(1)	C(14)	59.85(15)
C(14)	N(1)	S(1)	115.61(14)
C(16)	N(2)	C(17)	112.65(18)
C(16)	N(2)	C(19)	112.73(18)
C(16)	N(2)	B(1)	103.75(16)
C(17)	N(2)	B(1)	100.88(17)
C(19)	N(2)	C(17)	110.37(17)
C(19)	N(2)	B(1)	115.83(17)
O(3)	C(1)	C(2)	106.71(18)
O(4)	C(2)	O(6)	110.51(19)
O(4)	C(2)	C(1)	110.36(19)
O(4)	C(2)	C(4)	103.88(18)
O(6)	C(2)	C(1)	101.54(18)
O(6)	C(2)	C(4)	114.63(19)
C(1)	C(2)	C(4)	116.1(2)
O(4)	C(3)	C(5)	109.4(2)
O(4)	C(3)	C(6)	109.4(2)
O(5)	C(3)	O(4)	105.01(18)
O(5)	C(3)	C(5)	111.6(2)
O(5)	C(3)	C(6)	108.4(2)

Atom	Atom	Atom	Angle/°
C(6)	C(3)	C(5)	112.6(2)
O(5)	C(4)	C(2)	103.16(19)
O(5)	C(4)	C(10)	108.17(19)
C(10)	C(4)	C(2)	115.67(19)
O(6)	C(7)	C(8)	110.11(18)
O(7)	C(8)	C(7)	108.9(2)
O(7)	C(8)	C(10)	104.04(18)
C(7)	C(8)	C(10)	112.72(18)
O(7)	C(9)	C(11)	110.3(2)
O(7)	C(9)	C(12)	108.7(2)
O(8)	C(9)	O(7)	104.31(18)
O(8)	C(9)	C(11)	111.6(2)
O(8)	C(9)	C(12)	107.95(19)
C(12)	C(9)	C(11)	113.6(2)
O(8)	C(10)	C(4)	106.95(17)
O(8)	C(10)	C(8)	104.43(17)
C(4)	C(10)	C(8)	113.70(19)
N(1)	C(13)	C(14)	61.02(15)
N(1)	C(14)	B(1)	114.41(18)
C(13)	C(14)	N(1)	59.13(14)
C(13)	C(14)	B(1)	120.88(19)
O(9)	C(15)	C(16)	111.32(18)
O(11)	C(15)	O(9)	124.6(2)
O(11)	C(15)	C(16)	124.1(2)
N(2)	C(16)	C(15)	104.04(19)
N(2)	C(17)	C(18)	105.27(18)
O(10)	C(18)	C(17)	110.25(19)
O(12)	C(18)	O(10)	123.5(2)
O(12)	C(18)	C(17)	126.3(2)
O(9)	B(1)	N(2)	100.13(17)
O(9)	B(1)	C(14)	113.26(18)
O(10)	B(1)	O(9)	113.06(18)
O(10)	B(1)	N(2)	102.80(17)
O(10)	B(1)	C(14)	111.04(19)
C(14)	B(1)	N(2)	115.77(18)

Table S18: Torsion Angles in ° for **46.**

Atom	Atom	Atom	Atom	Angle/°
S(1)	O(3)	C(1)	C(2)	161.83(17)
S(1)	N(1)	C(13)	C(14)	-105.94(16)
S(1)	N(1)	C(14)	C(13)	106.20(19)
S(1)	N(1)	C(14)	B(1)	-141.13(16)
O(1)	S(1)	O(3)	C(1)	60.0(2)
O(1)	S(1)	N(1)	C(13)	163.85(16)
O(1)	S(1)	N(1)	C(14)	96.63(18)
O(2)	S(1)	O(3)	C(1)	-171.41(18)
O(2)	S(1)	N(1)	C(13)	31.47(19)
O(2)	S(1)	N(1)	C(14)	-35.8(2)

Atom	Atom	Atom	Atom	Angle/°
O(3)	S(1)	N(1)	C(13)	-79.81(18)
O(3)	S(1)	N(1)	C(14)	-147.03(16)
O(3)	C(1)	C(2)	O(4)	64.5(2)
O(3)	C(1)	C(2)	O(6)	-178.25(18)
O(3)	C(1)	C(2)	C(4)	-53.3(2)
O(4)	C(2)	C(4)	O(5)	25.2(2)
O(4)	C(2)	C(4)	C(10)	143.09(18)
O(5)	C(4)	C(10)	O(8)	-167.49(18)
O(5)	C(4)	C(10)	C(8)	77.8(2)
O(6)	C(2)	C(4)	O(5)	-95.5(2)
O(6)	C(2)	C(4)	C(10)	22.4(3)
O(6)	C(7)	C(8)	O(7)	-66.9(2)
O(6)	C(7)	C(8)	C(10)	48.0(2)
O(7)	C(8)	C(10)	O(8)	3.7(2)
O(7)	C(8)	C(10)	C(4)	119.9(2)
O(9)	C(15)	C(16)	N(2)	13.9(2)
O(11)	C(15)	C(16)	N(2)	-166.3(2)
N(1)	S(1)	O(3)	C(1)	-52.6(2)
N(1)	C(13)	C(14)	B(1)	-101.7(2)
N(1)	C(14)	B(1)	O(9)	-37.8(3)
N(1)	C(14)	B(1)	O(10)	-166.31(18)
N(1)	C(14)	B(1)	N(2)	77.0(2)
N(2)	C(17)	C(18)	O(10)	19.2(2)
N(2)	C(17)	C(18)	O(12)	-160.4(2)
C(1)	C(2)	C(4)	O(5)	146.52(18)
C(1)	C(2)	C(4)	C(10)	-95.6(2)
C(2)	O(4)	C(3)	O(5)	-13.7(2)
C(2)	O(4)	C(3)	C(5)	106.3(2)
C(2)	O(4)	C(3)	C(6)	-129.9(2)
C(2)	O(6)	C(7)	C(8)	-67.2(2)
C(2)	C(4)	C(10)	O(8)	77.5(2)
C(2)	C(4)	C(10)	C(8)	-37.3(3)
C(3)	O(4)	C(2)	O(6)	116.4(2)
C(3)	O(4)	C(2)	C(1)	-132.1(2)
C(3)	O(4)	C(2)	C(4)	-7.0(2)
C(3)	O(5)	C(4)	C(2)	-34.4(2)
C(3)	O(5)	C(4)	C(10)	-157.38(19)
C(4)	O(5)	C(3)	O(4)	30.6(2)
C(4)	O(5)	C(3)	C(5)	-87.9(2)
C(4)	O(5)	C(3)	C(6)	147.4(2)
C(7)	O(6)	C(2)	O(4)	-87.0(2)
C(7)	O(6)	C(2)	C(1)	155.9(2)
C(7)	O(6)	C(2)	C(4)	30.0(3)
C(7)	C(8)	C(10)	O(8)	-114.1(2)
C(7)	C(8)	C(10)	C(4)	2.1(3)
C(8)	O(7)	C(9)	O(8)	-31.4(2)
C(8)	O(7)	C(9)	C(11)	88.5(2)
C(8)	O(7)	C(9)	C(12)	-146.4(2)
C(9)	O(7)	C(8)	C(7)	137.34(18)
C(9)	O(7)	C(8)	C(10)	16.9(2)
C(9)	O(8)	C(10)	C(4)	-143.80(19)
C(9)	O(8)	C(10)	C(8)	-23.0(2)
C(10)	O(8)	C(9)	O(7)	33.8(2)
C(10)	O(8)	C(9)	C(11)	-85.3(3)
C(10)	O(8)	C(9)	C(12)	149.3(2)
C(13)	N(1)	C(14)	B(1)	112.7(2)
C(13)	C(14)	B(1)	O(9)	29.5(3)
C(13)	C(14)	B(1)	O(10)	-99.0(2)
C(13)	C(14)	B(1)	N(2)	144.4(2)
C(15)	O(9)	B(1)	O(10)	-128.7(2)
C(15)	O(9)	B(1)	N(2)	-20.0(2)

Atom	Atom	Atom	Atom	Angle/°
C(15)	O(9)	B(1)	C(14)	103.9(2)
C(16)	N(2)	C(17)	C(18)	-137.20(18)
C(16)	N(2)	B(1)	O(9)	27.0(2)
C(16)	N(2)	B(1)	O(10)	143.69(17)
C(16)	N(2)	B(1)	C(14)	-95.1(2)
C(17)	N(2)	C(16)	C(15)	83.5(2)
C(17)	N(2)	B(1)	O(9)	-89.75(18)
C(17)	N(2)	B(1)	O(10)	26.90(19)
C(17)	N(2)	B(1)	C(14)	148.12(18)
C(18)	O(10)	B(1)	O(9)	90.1(2)
C(18)	O(10)	B(1)	N(2)	-17.0(2)
C(18)	O(10)	B(1)	C(14)	-141.36(18)
C(19)	N(2)	C(16)	C(15)	-150.78(18)
C(19)	N(2)	C(17)	C(18)	95.8(2)
C(19)	N(2)	B(1)	O(9)	151.12(17)
C(19)	N(2)	B(1)	O(10)	-92.2(2)
C(19)	N(2)	B(1)	C(14)	29.0(3)
B(1)	O(9)	C(15)	O(11)	-174.6(2)
B(1)	O(9)	C(15)	C(16)	5.2(3)
B(1)	O(10)	C(18)	O(12)	179.4(2)
B(1)	O(10)	C(18)	C(17)	-0.3(2)
B(1)	N(2)	C(16)	C(15)	-24.7(2)
B(1)	N(2)	C(17)	C(18)	-27.17(19)

Table S19: Hydrogen Fractional Atomic Coordinates (×10^4) and Equivalent Isotropic Displacement Parameters (Å^2×10^3) for **46.** U_{eq} is defined as 1/3 of the trace of the orthogonalised U_{ij}.

Atom	x	y	z	U_{eq}
H(1A)	6386.12	2744.75	2730.89	26
H(1B)	6277.35	620.25	3200.49	26
H(4)	6603.41	6015.74	3796.67	24
H(5A)	6343.09	5427.51	5514.12	57
H(5B)	5944.77	2951.97	5460.6	57
H(5C)	5579.22	4549.26	4833.71	57
H(6A)	8731.02	4113.54	5746.75	62
H(6B)	9474.3	2806.53	5147.84	62
H(6C)	8349.59	1665.13	5601.32	62
H(7A)	10535.04	2473.88	3209.07	30
H(7B)	9977.29	3367.02	3933.59	30
H(8)	10511.36	6339.68	3260.39	27
H(10)	8629.47	8102.52	3527.93	24
H(11A)	8590.24	9974.12	1841.7	58
H(11B)	9937.69	8804.14	1616.97	58
H(11C)	9721.04	9462.99	2414.76	58
H(12A)	7621.56	4422	1606.49	48
H(12B)	8582.96	5756.51	1107.91	48
H(12C)	7201.45	6771.55	1353.93	48
H(13A)	5064.01	7519.23	2201.71	28
H(13B)	4151.79	7409.2	2922.59	28
H(14)	2323.62	5898.79	2272.19	24
H(16A)	3599.17	1530.92	275.51	26
H(16B)	3821.33	1268.62	1105.78	26
H(17A)	1183.77	4024.98	36.31	27
H(17B)	2674.8	4633.16	-193.61	27
H(19A)	1067.27	1569.57	949.92	35
H(19B)	788.01	3754.85	1358.6	35
H(19C)	1836.55	2062.83	1669.16	35

Atom	Occupancy
F1	0.867(11)
F2	0.867(11)
F3	0.867(11)
C16	0.867(11)
F1A	0.133(11)
F2A	0.133(11)
F3A	0.133(11)
C16A	0.133(11)

Experimental

Compound **69** was crystallized from acetonitrile. The atoms are depicted with 50% probability ellipsoids. The crystallographic data are summarized in the following tables.

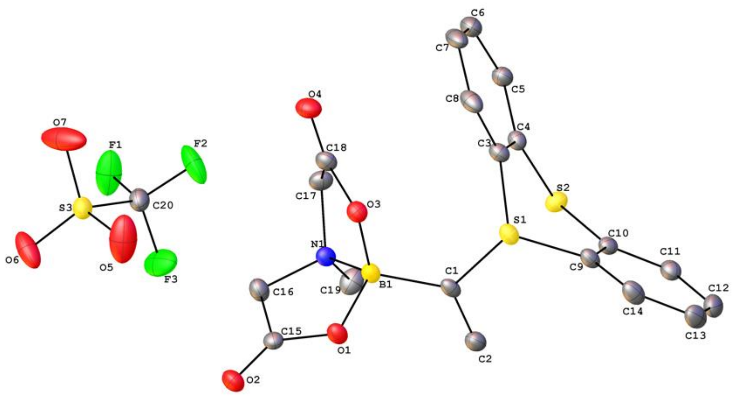

Figure S7: X-ray structure of Compound **69**.

**Compound**	**69**
Formula	$C_{20}H_{17}BF_3NO_7S_3$
$D_{calc.}$/ g cm^{-3}	1.640
μ/mm^{-1}	3.710
Formula Weight	547.34
Colour	colourless
Shape	block-shaped
Size/mm^3	0.44×0.29×0.15
T/K	100.0(3)
Crystal System	monoclinic
Space Group	$P2_1/n$
a/Å	12.75819(5)
b/Å	16.13758(7)
c/Å	43.24849(18)
α/°	90
β/°	95.3159(4)
γ/°	90
V/Å^3	8865.97(6)
Z	16
Z'	4
Wavelength/Å	1.54184
Radiation type	Cu K$_\alpha$
Θ_{min}/°	2.924
Θ_{max}/°	74.499
Measured Refl's.	231518
Indep't Refl's	18116
Refl's I≥2 σ(I)	16879
R_{int}	0.0392
Parameters	1376
Restraints	706
Largest Peak	0.506
Deepest Hole	-0.402
GooF	1.077
wR_2 (all data)	0.1113
wR_2	0.1100
R_1 (all data)	0.0399
R_1	0.0382

Structure Quality Indicators

**Reflections:**	d min (CuKα) 2Θ=149.0° 0.80	I/σ(I) 67.8	Rint m=13.00 3.92%	Full 135.4° 99.9
**Refinement:**	Shift -0.003	Max Peak 0.5	Min Peak -0.4	GooF 1.077

Reflection Statistics

Total reflections (after filtering)	235447	Unique reflections	18116
Completeness	0.999	Mean I/σ	37.53
hkl$_{max}$ collected	(15, 20, 54)	hkl$_{min}$ collected	(-15, -18, -54)
hkl$_{max}$ used	(15, 20, 54)	hkl$_{min}$ used	(-15, 0, 0)
Lim d$_{max}$ collected	100.0	Lim d$_{min}$ collected	0.77
d$_{max}$ used	16.14	d$_{min}$ used	0.8
Friedel pairs	23609	Friedel pairs merged	1
Inconsistent equivalents	8	R$_{int}$	0.0392
R$_{sigma}$	0.0147	Intensity transformed	0

Omitted reflections	0	Omitted by user (OMIT hkl)	111
Multiplicity	(11120, 11709, 8789, 6892, 5524, 4689, 3696, 2685, 1731, 1097, 709, 380, 200, 98, 41, 18, 5)	Maximum multiplicity	34
Removed systematic absences	3818	Filtered off (Shel/OMIT)	0

Table S21: Fractional Atomic Coordinates (×10[4]) and Equivalent Isotropic Displacement Parameters (Å[2]×10[3]) for **69.** U_{eq} is defined as 1/3 of the trace of the orthogonalised U_{ij}.

Atom	x	y	z	U_{eq}
S(1)	2263.4(3)	8957.8(2)	4657.1(2)	15.05(9)
S(2)	657.2(3)	10326.8(2)	4306.1(2)	15.99(9)
O(1)	2746.5(8)	7224.1(7)	4060.5(2)	17.6(2)
O(2)	2917.3(9)	6140.6(7)	3744.8(3)	22.6(2)
O(3)	1476.7(8)	7268.9(7)	4445.9(2)	16.3(2)
O(4)	-45.5(9)	6727.0(7)	4578.0(3)	23.5(2)
N(1)	918.7(10)	7584.3(8)	3911.0(3)	14.0(2)
C(1)	2273.8(11)	8631.7(10)	4257.7(3)	15.0(3)
C(2)	2712.0(12)	9128.3(10)	4061.0(4)	19.4(3)
C(3)	909.7(12)	9002.4(9)	4717.1(3)	15.8(3)
C(4)	229.6(12)	9565.5(9)	4556.3(3)	15.2(3)
C(5)	-844.2(12)	9519.4(10)	4593.9(4)	18.7(3)
C(6)	-1210.8(14)	8922.3(10)	4789.3(4)	23.0(3)
C(7)	-514.4(14)	8391.7(10)	4960.1(4)	24.3(4)
C(8)	553.8(14)	8437.3(10)	4926.4(4)	21.2(3)
C(9)	2585.9(12)	10019.7(10)	4648.7(3)	16.4(3)
C(10)	1902.2(12)	10584.7(10)	4490.5(3)	15.0(3)
C(11)	2229.3(13)	11406.3(10)	4471.6(4)	18.4(3)
C(12)	3206.9(14)	11644.8(11)	4608.3(4)	23.1(3)
C(13)	3870.2(13)	11082.2(11)	4772.5(4)	24.0(4)
C(14)	3560.3(13)	10261.7(11)	4793.6(4)	22.2(3)
C(15)	2388.3(12)	6668.7(9)	3847.4(3)	15.5(3)
C(16)	1218.1(13)	6799.6(10)	3758.0(4)	19.9(3)
C(17)	-5.6(12)	7471.6(10)	4093.6(4)	19.5(3)
C(18)	440.3(12)	7095.5(10)	4398.5(4)	17.8(3)
C(19)	719.6(13)	8260.9(10)	3675.3(4)	22.5(3)
B(1)	1898.3(13)	7695.5(10)	4183.0(4)	14.1(3)
S(1B)	2779.7(3)	3922.8(2)	2851.4(2)	15.13(8)
S(2B)	4353.6(3)	5318.4(2)	3203.6(2)	17.82(9)
O(1B)	2291.1(8)	2183.5(7)	3448.5(3)	18.6(2)
O(2B)	2119.6(9)	1113.3(7)	3770.2(3)	23.5(3)
O(3B)	3552.9(8)	2224.3(7)	3060.8(2)	17.0(2)
O(4B)	5067.1(9)	1676.0(8)	2926.5(3)	23.6(2)
N(1B)	4119.0(10)	2543.1(8)	3594.7(3)	14.2(2)
C(1B)	2761.9(11)	3589.7(10)	3249.6(3)	15.1(3)
C(2B)	2318.2(12)	4080.0(10)	3447.3(4)	20.4(3)
C(3B)	4136.4(12)	3981.4(10)	2796.9(3)	15.8(3)
C(4B)	4801.0(12)	4558.9(9)	2955.8(3)	15.7(3)
C(5B)	5875.7(12)	4524.9(10)	2920.5(4)	19.4(3)
C(6B)	6260.6(13)	3927.1(10)	2729.9(4)	21.9(3)
C(7B)	5584.2(14)	3379.0(10)	2562.1(4)	22.6(3)
C(8B)	4513.8(13)	3411.2(10)	2592.8(4)	19.5(3)
C(9B)	2434.0(12)	4978.9(10)	2861.0(4)	17.1(3)
C(10B)	3102.4(12)	5555.3(10)	3019.9(3)	16.9(3)
C(11B)	2750.1(13)	6369.6(10)	3041.1(4)	20.3(3)
C(12B)	1766.4(14)	6593.0(11)	2904.6(4)	25.0(4)
C(13B)	1119.6(13)	6021.8(12)	2739.2(4)	26.1(4)
C(14B)	1453.1(13)	5205.6(11)	2717.9(4)	22.8(3)
C(15B)	2649.6(12)	1637.0(9)	3663.8(3)	16.1(3)
C(16B)	3824.9(13)	1762.7(10)	3750.4(4)	21.0(3)
C(17B)	5042.2(12)	2426.2(10)	3410.7(4)	20.7(3)

Atom	x	y	z	U_{eq}
C(18B)	4585.6(12)	2047.7(10)	3106.6(4)	17.9(3)
C(19B)	4326.1(13)	3224.2(10)	3828.7(4)	23.5(3)
B(1B)	3136.6(13)	2653.7(11)	3323.8(4)	14.9(3)
S(1D)	2059.8(3)	8838.3(2)	7183.1(2)	15.42(8)
S(2D)	596.0(3)	10253.8(2)	6804.0(2)	16.60(9)
O(1D)	2593.6(8)	6990.7(7)	6640.3(3)	18.2(2)
O(2D)	3076.0(9)	6228.1(7)	6246.3(3)	23.5(2)
O(3D)	1217.4(8)	7202.1(7)	6981.4(2)	16.6(2)
O(4D)	-250.9(9)	6502.5(7)	7063.9(3)	22.1(2)
N(1D)	875.5(9)	7434.2(8)	6421.8(3)	13.8(2)
C(1D)	2164.1(11)	8471.0(9)	6792.0(3)	15.3(3)
C(2D)	2670.6(13)	8932.4(10)	6600.5(4)	20.8(3)
C(3D)	687.7(12)	8924.5(10)	7215.4(4)	17.3(3)
C(4D)	77.8(12)	9516.3(9)	7048.1(3)	15.9(3)
C(5D)	-1008.8(13)	9517.6(10)	7069.7(4)	21.3(3)
C(6D)	-1458.5(14)	8928.3(11)	7250.7(4)	25.6(4)
C(7D)	-835.2(15)	8353.9(11)	7421.7(4)	27.4(4)
C(8D)	245.5(14)	8358.9(10)	7409.6(4)	23.2(3)
C(9D)	2450.3(12)	9885.7(9)	7172.2(3)	15.9(3)
C(10D)	1828.3(12)	10473.4(10)	7003.4(3)	15.3(3)
C(11D)	2211.3(13)	11280.5(10)	6985.1(4)	17.9(3)
C(12D)	3183.3(14)	11480.9(11)	7134.6(4)	21.9(3)
C(13D)	3784.5(13)	10898.8(11)	7309.7(4)	23.4(3)
C(14D)	3416.8(13)	10090.7(11)	7330.1(4)	20.7(3)
C(15D)	2465.8(12)	6692.9(9)	6351.6(4)	16.1(3)
C(16D)	1488.8(12)	7065.1(11)	6178.3(4)	20.9(3)
C(17D)	111.4(12)	6819.7(10)	6539.4(4)	19.5(3)
C(18D)	312.6(12)	6819.8(10)	6890.4(4)	16.8(3)
C(19D)	295.1(13)	8195.4(10)	6310.8(4)	24.9(4)
B(1D)	1750.9(13)	7539.2(11)	6723.8(4)	14.7(3)
S(1F)	2953.2(3)	3801.2(2)	5324.2(2)	14.88(8)
S(2F)	4380.7(3)	5238.6(2)	5706.3(2)	17.71(9)
O(1F)	2390.9(8)	1950.1(7)	5864.0(3)	17.5(2)
O(2F)	1895.7(9)	1198.4(7)	6259.4(3)	23.4(2)
O(3F)	3772.3(8)	2162.7(7)	5525.3(2)	16.2(2)
O(4F)	5230.2(9)	1448.3(7)	5444.1(3)	22.2(2)
N(1F)	4107.0(9)	2391.1(8)	6084.5(3)	13.7(2)
C(1F)	2830.3(11)	3431.1(9)	5713.6(3)	14.8(3)
C(2F)	2313.9(13)	3890.1(10)	5903.7(4)	20.7(3)
C(3F)	4326.0(12)	3898.4(9)	5299.4(3)	16.1(3)
C(4F)	4919.8(12)	4500.3(9)	5466.4(4)	16.1(3)
C(5F)	6008.8(13)	4510.8(10)	5449.0(4)	21.0(3)
C(6F)	6474.3(14)	3925.1(11)	5270.8(4)	25.1(4)
C(7F)	5868.6(14)	3341.5(11)	5099.0(4)	25.1(4)
C(8F)	4786.1(14)	3334.4(10)	5109.2(4)	21.4(3)
C(9F)	2545.9(12)	4843.7(9)	5333.3(3)	15.8(3)
C(10F)	3149.1(12)	5439.9(10)	5503.2(3)	16.3(3)
C(11F)	2742.3(13)	6241.0(10)	5519.5(4)	19.6(3)
C(12F)	1768.9(14)	6424.8(11)	5368.1(4)	24.0(4)
C(13F)	1189.3(13)	5832.9(12)	5191.3(4)	25.7(4)
C(14F)	1578.2(13)	5033.9(11)	5173.1(4)	22.0(3)
C(15F)	2511.7(12)	1660.4(9)	6154.0(4)	16.2(3)
C(16F)	3483.7(12)	2033.0(10)	6328.4(4)	20.2(3)
C(17F)	4864.2(12)	1770.7(10)	5968.6(4)	18.7(3)
C(18F)	4669.7(12)	1770.9(10)	5616.7(4)	16.6(3)
C(19F)	4694.4(13)	3149.0(10)	6196.3(4)	23.3(3)
B(1F)	3238.2(13)	2499.2(11)	5782.1(4)	14.6(3)
S(3)	1515.4(3)	3600.7(2)	4353.8(2)	19.40(9)
C(20)	983.3(13)	4617.7(10)	4239.0(4)	22.3(3)
F(1)	96(3)	4551.2(19)	4082.8(11)	44.0(10)
F(2)	915(4)	5082(2)	4486.6(7)	39.6(9)

Atom	x	y	z	U_{eq}
F(3)	1684(3)	4979.5(15)	4071.9(10)	41.3(9)
O(5)	2528(3)	3754(2)	4534.8(13)	51.6(13)
O(6)	1631(4)	3162(2)	4078.0(7)	37.0(10)
O(7)	767(4)	3254(3)	4547.3(15)	58.0(15)
F(1A)	-98(2)	4655(3)	4231.9(11)	39.7(9)
F(2A)	1317(4)	5248(2)	4410.8(13)	50.4(13)
F(3A)	1103(4)	4843.9(18)	3939.8(8)	40.9(12)
O(5A)	2588(2)	3749(2)	4315.4(15)	45.3(13)
O(6A)	996(5)	3106(2)	4104.4(10)	46.1(13)
O(7A)	1186(4)	3500(3)	4645.4(8)	42.3(12)
S(3B)	3503.7(3)	-1438.8(3)	3136.8(2)	21.87(10)
C(20B)	3989.6(13)	-400.3(11)	3237.8(4)	24.3(3)
F(1B)	4872.8(15)	-430.5(10)	3409.6(6)	43.4(5)
F(2B)	4124.7(19)	10.3(11)	2979.1(4)	47.8(5)
F(3B)	3284.1(13)	-7.7(10)	3382.7(5)	41.0(5)
O(5B)	2566(2)	-1326.5(14)	2933.0(6)	59.3(8)
O(6B)	3322.2(19)	-1812.8(11)	3429.2(4)	34.5(5)
O(7B)	4346(2)	-1840.9(13)	2992.6(7)	59.7(8)
F(1C)	5051(3)	-251(3)	3220.0(14)	41.5(13)
F(2C)	3537(4)	288(3)	3122.9(15)	52.3(18)
F(3C)	4032(5)	-198(3)	3562.1(9)	47.8(16)
O(5C)	2464(4)	-1295(4)	3224.5(18)	52.1(19)
O(6C)	4182(7)	-1887(3)	3344.0(18)	70(3)
O(7C)	3646(6)	-1384(4)	2822.5(11)	51.5(19)
S(3D)	1691.9(3)	3574.8(2)	6808.8(2)	17.45(9)
F(1D)	1523.5(9)	5169.2(7)	6924.4(3)	35.4(3)
F(2D)	1436.3(11)	4873.8(7)	6440.7(3)	42.2(3)
F(3D)	122.3(8)	4624.6(7)	6699.9(3)	31.3(2)
O(5D)	1198.0(11)	3077.0(8)	6558.3(3)	28.2(3)
O(6D)	1360.5(13)	3415.4(9)	7109.2(3)	37.9(3)
O(7D)	2806.2(10)	3674.6(9)	6792.2(4)	37.7(3)
C(20D)	1166.0(13)	4609.2(10)	6715.3(4)	18.8(3)
S(3F)	3249.7(3)	-1444.6(2)	5682.5(2)	20.46(9)
F(1F)	3420.2(10)	136.8(8)	5546.6(3)	46.3(3)
F(2F)	3508.6(13)	-116.2(8)	6031.0(3)	53.8(4)
F(3F)	4818.2(8)	-391.3(7)	5776.5(3)	34.8(3)
O(5F)	3736.3(11)	-1918.4(8)	5937.9(3)	31.8(3)
O(6F)	3555.6(16)	-1625.1(11)	5383.4(4)	56.5(5)
O(7F)	2132.3(11)	-1336.1(10)	5702.2(4)	46.7(4)
C(20F)	3781.0(13)	-407.0(10)	5760.3(4)	21.2(3)

Table S22: Anisotropic Displacement Parameters (×10[4]) for **69**. The anisotropic displacement factor exponent takes the form: $-2\pi^2[h^2a^{*2} \times U_{11}+ ... +2hka^* \times b^* \times U_{12}]$

Atom	U_{11}	U_{22}	U_{33}	U_{23}	U_{13}	U_{12}
S(1)	16.18(17)	15.27(17)	13.21(17)	-0.84(12)	-1.28(13)	4.56(13)
S(2)	13.68(17)	16.96(18)	16.74(18)	4.73(13)	-1.75(13)	0.61(13)
O(1)	14.3(5)	18.8(5)	19.4(5)	-3.7(4)	0.3(4)	3.4(4)
O(2)	23.4(6)	20.3(6)	24.8(6)	-4.9(5)	6.5(5)	3.5(5)
O(3)	18.3(5)	16.2(5)	14.2(5)	1.2(4)	-0.1(4)	2.7(4)
O(4)	25.0(6)	24.5(6)	22.1(6)	2.9(5)	8.6(5)	1.4(5)
N(1)	13.0(6)	15.8(6)	12.9(6)	0.4(5)	-1.0(5)	1.9(5)
C(1)	11.5(7)	19.2(7)	13.9(7)	-3.5(5)	-0.2(5)	4.4(5)
C(2)	17.3(7)	20.9(8)	20.3(7)	-3.6(6)	3.8(6)	0.2(6)
C(3)	17.5(7)	16.5(7)	13.8(7)	-1.0(6)	2.6(5)	2.6(6)
C(4)	18.4(7)	13.5(7)	13.7(7)	-0.4(5)	2.1(5)	1.4(6)
C(5)	17.7(7)	18.7(7)	20.2(7)	-2.2(6)	4.0(6)	1.4(6)
C(6)	24.5(8)	21.0(8)	24.7(8)	-4.7(6)	9.7(7)	-1.8(6)
C(7)	34.5(9)	17.5(8)	23.7(8)	-0.7(6)	16.8(7)	-0.4(7)

Atom	U_{11}	U_{22}	U_{33}	U_{23}	U_{13}	U_{12}
C(8)	32.4(9)	16.1(7)	16.1(7)	2.0(6)	7.8(6)	5.9(6)
C(9)	14.9(7)	19.0(7)	15.2(7)	-4.5(6)	0.8(5)	3.0(6)
C(10)	15.3(7)	17.7(7)	12.3(6)	-2.3(5)	2.5(5)	-0.7(6)
C(11)	21.8(8)	18.0(8)	15.9(7)	-0.8(6)	4.3(6)	0.4(6)
C(12)	26.6(8)	21.9(8)	21.4(8)	-7.7(6)	4.5(6)	-6.0(7)
C(13)	17.8(8)	30.8(9)	23.0(8)	-10.8(7)	-1.2(6)	-2.6(7)
C(14)	17.9(8)	27.5(9)	20.5(8)	-7.9(6)	-2.0(6)	4.5(6)
C(15)	17.4(7)	14.8(7)	14.8(7)	1.2(6)	4.2(5)	-1.4(6)
C(16)	21.2(8)	18.2(8)	20.2(7)	-4.6(6)	1.0(6)	1.6(6)
C(17)	14.1(7)	23.4(8)	20.9(8)	0.9(6)	1.7(6)	0.3(6)
C(18)	16.2(7)	18.6(7)	18.8(7)	-1.3(6)	2.5(6)	3.8(6)
C(19)	24.8(8)	21.0(8)	20.3(8)	6.1(6)	-5.2(6)	-0.6(6)
B(1)	12.6(7)	14.8(8)	14.5(7)	-1.6(6)	-1.4(6)	3.0(6)
S(1B)	14.54(17)	16.98(18)	13.41(17)	1.21(13)	-1.17(13)	-4.05(13)
S(2B)	15.36(18)	18.37(18)	19.04(18)	-6.01(14)	-2.02(14)	-0.45(13)
O(1B)	13.7(5)	20.7(5)	21.2(5)	5.1(4)	0.3(4)	-3.1(4)
O(2B)	25.7(6)	20.5(6)	25.4(6)	3.1(5)	8.3(5)	-4.3(5)
O(3B)	17.8(5)	17.6(5)	15.1(5)	-1.5(4)	-0.5(4)	-2.3(4)
O(4B)	25.1(6)	25.6(6)	21.4(6)	-2.4(5)	8.6(5)	0.4(5)
N(1B)	13.1(6)	16.0(6)	13.1(6)	-0.4(5)	-1.5(5)	-1.4(5)
C(1B)	10.6(6)	19.8(7)	14.6(7)	3.2(6)	0.2(5)	-3.5(5)
C(2B)	18.2(7)	22.8(8)	20.6(8)	4.9(6)	4.3(6)	1.4(6)
C(3B)	15.8(7)	18.1(7)	13.7(7)	0.5(6)	1.8(5)	-3.0(6)
C(4B)	17.0(7)	14.9(7)	15.2(7)	0.1(5)	1.6(5)	-1.8(6)
C(5B)	17.0(7)	19.7(8)	21.6(8)	1.7(6)	2.3(6)	-3.6(6)
C(6B)	21.0(8)	22.2(8)	23.6(8)	4.9(6)	7.9(6)	1.4(6)
C(7B)	29.3(9)	20.1(8)	20.5(8)	1.5(6)	13.4(6)	0.8(7)
C(8B)	28.4(8)	16.5(7)	14.3(7)	-1.4(6)	5.2(6)	-4.4(6)
C(9B)	14.7(7)	20.1(8)	16.4(7)	4.1(6)	1.6(5)	-1.6(6)
C(10B)	16.8(7)	20.0(8)	14.2(7)	2.1(6)	2.5(5)	1.5(6)
C(11B)	26.0(8)	18.7(8)	16.9(7)	1.6(6)	5.8(6)	1.4(6)
C(12B)	28.6(9)	25.5(8)	21.9(8)	10.8(7)	7.9(7)	8.8(7)
C(13B)	19.3(8)	34.0(10)	25.1(8)	14.9(7)	2.3(6)	5.1(7)
C(14B)	17.4(8)	30.2(9)	20.4(8)	10.1(7)	0.1(6)	-1.6(6)
C(15B)	18.9(7)	14.7(7)	15.4(7)	-1.9(6)	4.7(6)	0.7(6)
C(16B)	21.7(8)	20.5(8)	20.3(8)	4.6(6)	-0.1(6)	-1.6(6)
C(17B)	14.5(7)	24.8(8)	23.0(8)	-1.2(6)	1.7(6)	-0.7(6)
C(18B)	15.3(7)	19.9(8)	18.6(7)	1.5(6)	2.8(6)	-2.8(6)
C(19B)	25.0(8)	21.4(8)	22.6(8)	-6.5(6)	-6.7(6)	0.9(6)
B(1B)	11.7(7)	17.5(8)	15.2(7)	1.5(6)	-1.1(6)	-2.3(6)
S(1D)	17.45(18)	13.99(17)	14.37(17)	0.53(13)	-0.96(13)	3.71(13)
S(2D)	15.00(17)	15.62(18)	18.43(18)	5.02(13)	-2.47(13)	0.02(13)
O(1D)	14.4(5)	18.6(5)	21.1(5)	-3.5(4)	-0.8(4)	3.9(4)
O(2D)	22.8(6)	22.7(6)	26.0(6)	-2.7(5)	7.6(5)	4.3(5)
O(3D)	17.9(5)	15.7(5)	15.7(5)	0.9(4)	-1.0(4)	-0.6(4)
O(4D)	20.8(6)	25.3(6)	20.6(6)	5.4(5)	4.9(4)	-0.4(5)
N(1D)	11.0(6)	16.0(6)	14.3(6)	1.6(5)	0.1(5)	0.5(5)
C(1D)	12.2(7)	16.4(7)	17.1(7)	-3.0(6)	0.2(5)	3.6(5)
C(2D)	19.7(8)	20.9(8)	22.7(8)	-3.5(6)	6.0(6)	-1.3(6)
C(3D)	20.4(7)	15.7(7)	16.2(7)	0.1(6)	4.1(6)	3.2(6)
C(4D)	17.9(7)	13.8(7)	16.2(7)	-0.8(5)	2.7(6)	0.4(6)
C(5D)	18.7(8)	20.2(8)	25.1(8)	-3.0(6)	3.6(6)	1.3(6)
C(6D)	21.9(8)	25.3(9)	31.5(9)	-8.4(7)	12.4(7)	-4.5(7)
C(7D)	38.0(10)	19.2(8)	28.0(9)	-2.0(7)	19.6(8)	-3.6(7)
C(8D)	33.9(9)	16.5(7)	20.6(8)	1.4(6)	10.0(7)	4.7(7)
C(9D)	16.2(7)	15.6(7)	15.9(7)	-1.9(6)	1.9(5)	0.3(6)
C(10D)	16.2(7)	17.6(7)	12.3(7)	-0.7(5)	1.9(5)	0.1(6)
C(11D)	23.0(8)	16.1(7)	15.0(7)	-0.1(6)	4.0(6)	-1.0(6)
C(12D)	25.2(8)	22.6(8)	18.3(7)	-6.1(6)	4.7(6)	-8.1(6)
C(13D)	18.8(8)	31.1(9)	19.9(8)	-8.0(7)	-0.2(6)	-5.4(7)
C(14D)	17.7(7)	25.6(8)	18.1(7)	-3.9(6)	-2.0(6)	1.7(6)

Atom	U_{11}	U_{22}	U_{33}	U_{23}	U_{13}	U_{12}
C(15D)	16.9(7)	14.3(7)	17.6(7)	-0.2(6)	5.0(6)	-2.6(6)
C(16D)	18.6(7)	26.0(8)	18.2(7)	-2.7(6)	2.7(6)	0.2(6)
C(17D)	16.0(7)	22.9(8)	19.7(7)	2.9(6)	1.3(6)	-4.5(6)
C(18D)	13.4(7)	18.2(7)	18.9(7)	1.6(6)	1.9(6)	3.2(6)
C(19D)	22.6(8)	20.7(8)	29.9(9)	4.8(7)	-6.0(7)	4.0(6)
B(1D)	13.7(7)	15.4(8)	14.5(8)	-0.3(6)	-0.9(6)	2.1(6)
S(1F)	15.48(17)	14.87(17)	13.85(17)	-0.30(12)	-1.05(13)	-3.44(13)
S(2F)	16.23(18)	16.56(18)	19.57(18)	-5.78(13)	-2.53(14)	-0.36(13)
O(1F)	13.3(5)	18.4(5)	20.3(5)	3.1(4)	-0.5(4)	-3.6(4)
O(2F)	23.9(6)	22.0(6)	25.6(6)	2.3(5)	8.4(5)	-4.2(5)
O(3F)	17.1(5)	15.4(5)	15.5(5)	-0.2(4)	-1.1(4)	1.0(4)
O(4F)	21.2(6)	25.4(6)	20.8(6)	-4.2(5)	5.8(4)	1.7(5)
N(1F)	11.1(6)	16.4(6)	13.5(6)	-1.0(5)	0.1(4)	0.4(5)
C(1F)	11.4(6)	16.3(7)	16.4(7)	3.1(5)	0.1(5)	-2.9(5)
C(2F)	20.1(8)	20.4(8)	22.6(8)	3.8(6)	6.8(6)	2.2(6)
C(3F)	17.2(7)	15.7(7)	15.5(7)	-0.1(6)	2.7(6)	-2.5(6)
C(4F)	16.8(7)	14.9(7)	16.7(7)	0.3(6)	2.1(5)	-1.1(6)
C(5F)	17.5(8)	20.0(8)	25.6(8)	2.2(6)	2.1(6)	-3.0(6)
C(6F)	20.1(8)	24.5(8)	32.0(9)	8.0(7)	10.3(7)	2.9(6)
C(7F)	33.5(9)	19.0(8)	25.5(8)	2.1(6)	16.2(7)	2.0(7)
C(8F)	30.0(9)	17.5(7)	17.7(7)	-1.5(6)	7.7(6)	-3.6(6)
C(9F)	15.9(7)	15.2(7)	16.3(7)	3.1(5)	2.2(5)	0.5(6)
C(10F)	16.7(7)	18.3(7)	14.1(7)	1.4(6)	2.3(5)	0.7(6)
C(11F)	25.7(8)	16.1(7)	18.1(7)	1.9(6)	7.4(6)	2.5(6)
C(12F)	28.4(9)	23.8(8)	20.6(8)	8.4(6)	7.4(7)	9.6(7)
C(13F)	19.7(8)	35.3(10)	22.3(8)	11.9(7)	2.3(6)	7.2(7)
C(14F)	17.3(7)	28.6(9)	19.4(8)	5.1(6)	-1.5(6)	-0.4(6)
C(15F)	17.8(7)	14.2(7)	17.5(7)	0.2(6)	5.7(6)	3.2(6)
C(16F)	19.1(7)	25.2(8)	16.5(7)	1.5(6)	2.6(6)	0.7(6)
C(17F)	15.5(7)	20.9(8)	19.5(7)	-2.0(6)	0.9(6)	4.3(6)
C(18F)	13.5(7)	18.2(7)	18.3(7)	-0.9(6)	2.7(6)	-2.1(6)
C(19F)	20.1(8)	19.7(8)	28.5(8)	-4.9(6)	-5.8(6)	-3.8(6)
B(1F)	12.4(7)	16.2(8)	14.9(8)	0.2(6)	0.3(6)	-2.1(6)
S(3)	22.6(2)	16.27(18)	19.42(19)	-2.39(14)	2.34(15)	-1.32(14)
C(20)	29.1(9)	18.6(8)	19.8(8)	-2.9(6)	5.0(6)	-3.2(6)
F(1)	42.7(18)	25.3(12)	58(2)	-2.4(15)	-27.8(17)	5.6(12)
F(2)	69(3)	23.0(16)	26.5(13)	-8.0(11)	0.8(13)	16.9(15)
F(3)	47.5(18)	20.9(11)	59(2)	15.6(12)	24.1(16)	2.7(11)
O(5)	48(2)	29.8(16)	70(3)	-6.5(17)	-30(2)	8.7(13)
O(6)	70(3)	21.3(14)	19.5(13)	-2.8(10)	2.9(16)	12.0(17)
O(7)	64(3)	37(2)	82(4)	31(2)	52(3)	23(2)
F(1A)	26.6(13)	39.8(18)	53(2)	22.9(17)	3.3(14)	7.6(11)
F(2A)	64(3)	17.5(16)	64(3)	-16.0(17)	-27(2)	5.5(16)
F(3A)	71(3)	23.6(13)	32.5(16)	13.8(11)	28.5(17)	19.7(15)
O(5A)	20.7(15)	27.1(16)	89(4)	12.4(19)	10.8(18)	3.2(12)
O(6A)	77(3)	16.4(14)	38(2)	-4.2(13)	-35(2)	1.4(19)
O(7A)	63(3)	45(3)	20.1(16)	16.0(15)	11.9(16)	26(2)
S(3B)	24.0(2)	24.9(2)	16.50(19)	0.91(15)	0.47(15)	0.51(15)
C(20B)	27.5(9)	21.7(8)	23.9(8)	6.0(6)	3.6(7)	5.4(7)
F(1B)	35.4(10)	25.4(8)	64.2(14)	4.4(9)	-23.1(10)	-7.6(7)
F(2B)	88.8(16)	26.1(9)	29.1(8)	7.8(7)	8.5(9)	-17.7(9)
F(3B)	40.3(9)	21.5(8)	63.7(12)	-14.4(8)	17.9(9)	1.1(7)
O(5B)	64.6(16)	36.5(12)	67.3(17)	5.2(11)	-44.3(14)	-10.9(11)
O(6B)	60.5(14)	23.6(9)	20.5(9)	-1.6(7)	9.4(8)	-12.7(9)
O(7B)	65.3(16)	28.9(11)	94(2)	-24.0(12)	57.5(15)	-15.5(11)
F(1C)	28(2)	39(3)	58(3)	-24(3)	4(2)	-4.4(19)
F(2C)	50(3)	16(2)	85(4)	17(2)	-26(3)	1(2)
F(3C)	90(4)	24(2)	33(2)	-11.5(18)	22(3)	-25(2)
O(5C)	44(3)	29(3)	89(5)	-19(3)	34(3)	-15(2)
O(6C)	109(6)	16(3)	71(5)	1(3)	-66(5)	3(3)
O(7C)	72(4)	61(4)	24(3)	-23(3)	13(3)	-38(4)

Atom	U_{11}	U_{22}	U_{33}	U_{23}	U_{13}	U_{12}
S(3D)	17.95(18)	15.61(18)	18.28(18)	-2.26(13)	-1.12(14)	1.87(13)
F(1D)	37.6(6)	22.3(5)	44.4(7)	-16.2(5)	-6.8(5)	4.3(4)
F(2D)	71.5(9)	23.3(5)	36.5(6)	9.8(5)	30.1(6)	11.0(5)
F(3D)	19.4(5)	26.4(5)	47.4(7)	4.6(5)	-0.8(4)	4.6(4)
O(5D)	36.5(7)	17.9(6)	28.5(6)	-4.5(5)	-6.7(5)	-1.1(5)
O(6D)	57.8(9)	34.8(7)	22.5(6)	9.1(6)	10.6(6)	17.8(7)
O(7D)	18.4(6)	27.9(7)	66.6(10)	-11.7(7)	3.1(6)	1.8(5)
C(20D)	20.3(8)	17.2(7)	19.3(7)	-2.1(6)	3.7(6)	-0.2(6)
S(3F)	23.8(2)	18.99(19)	17.76(19)	2.13(14)	-2.44(14)	-4.87(14)
F(1F)	37.8(7)	32.0(6)	66.5(9)	29.0(6)	-8.4(6)	-6.2(5)
F(2F)	87.4(11)	25.3(6)	55.9(8)	-15.3(6)	44.7(8)	-16.0(6)
F(3F)	19.7(5)	27.2(6)	56.7(7)	-0.2(5)	0.3(5)	-4.5(4)
O(5F)	37.1(7)	19.2(6)	36.9(7)	6.4(5)	-9.2(6)	-0.7(5)
O(6F)	92.5(14)	50.7(10)	29.2(8)	-16.5(7)	21.4(8)	-33.5(10)
O(7F)	23.1(7)	36.6(8)	79.3(12)	22.8(8)	-1.5(7)	-4.1(6)
C(20F)	22.1(8)	17.0(8)	25.0(8)	2.9(6)	5.3(6)	0.1(6)

Table S23: Bond Lengths in Å for **69.**

Atom	Atom	Length/Å
S(1)	C(1)	1.8070(15)
S(1)	C(3)	1.7714(16)
S(1)	C(9)	1.7635(16)
S(2)	C(4)	1.7572(15)
S(2)	C(10)	1.7600(15)
O(1)	C(15)	1.3357(19)
O(1)	B(1)	1.4616(19)
O(2)	C(15)	1.1976(19)
O(3)	C(18)	1.3485(19)
O(3)	B(1)	1.473(2)
O(4)	C(18)	1.196(2)
N(1)	C(16)	1.4949(19)
N(1)	C(17)	1.4898(19)
N(1)	C(19)	1.4992(19)
N(1)	B(1)	1.645(2)
C(1)	C(2)	1.329(2)
C(1)	B(1)	1.609(2)
C(3)	C(4)	1.397(2)
C(3)	C(8)	1.390(2)
C(4)	C(5)	1.396(2)
C(5)	C(6)	1.391(2)
C(6)	C(7)	1.395(3)
C(7)	C(8)	1.386(2)
C(9)	C(10)	1.396(2)
C(9)	C(14)	1.395(2)
C(10)	C(11)	1.395(2)
C(11)	C(12)	1.384(2)
C(12)	C(13)	1.390(3)
C(13)	C(14)	1.387(3)
C(15)	C(16)	1.522(2)
C(17)	C(18)	1.514(2)
S(1B)	C(1B)	1.8063(15)
S(1B)	C(3B)	1.7709(15)
S(1B)	C(9B)	1.7619(16)
S(2B)	C(4B)	1.7578(16)
S(2B)	C(10B)	1.7581(16)
O(1B)	C(15B)	1.3323(19)
O(1B)	B(1B)	1.4621(19)
O(2B)	C(15B)	1.200(2)

Atom	Atom	Length/Å
O(3B)	C(18B)	1.3447(19)
O(3B)	B(1B)	1.472(2)
O(4B)	C(18B)	1.197(2)
N(1B)	C(16B)	1.4922(19)
N(1B)	C(17B)	1.4934(19)
N(1B)	C(19B)	1.5007(19)
N(1B)	B(1B)	1.644(2)
C(1B)	C(2B)	1.329(2)
C(1B)	B(1B)	1.608(2)
C(3B)	C(4B)	1.397(2)
C(3B)	C(8B)	1.391(2)
C(4B)	C(5B)	1.395(2)
C(5B)	C(6B)	1.388(2)
C(6B)	C(7B)	1.392(2)
C(7B)	C(8B)	1.385(2)
C(9B)	C(10B)	1.399(2)
C(9B)	C(14B)	1.393(2)
C(10B)	C(11B)	1.395(2)
C(11B)	C(12B)	1.384(2)
C(12B)	C(13B)	1.390(3)
C(13B)	C(14B)	1.390(3)
C(15B)	C(16B)	1.525(2)
C(17B)	C(18B)	1.516(2)
S(1D)	C(1D)	1.8086(15)
S(1D)	C(3D)	1.7745(16)
S(1D)	C(9D)	1.7640(16)
S(2D)	C(4D)	1.7599(16)
S(2D)	C(10D)	1.7577(16)
O(1D)	C(15D)	1.3337(19)
O(1D)	B(1D)	1.4635(19)
O(2D)	C(15D)	1.201(2)
O(3D)	C(18D)	1.3356(19)
O(3D)	B(1D)	1.463(2)
O(4D)	C(18D)	1.201(2)
N(1D)	C(16D)	1.4930(19)
N(1D)	C(17D)	1.5112(19)
N(1D)	C(19D)	1.4907(19)
N(1D)	B(1D)	1.647(2)
C(1D)	C(2D)	1.326(2)

Atom	Atom	Length/Å
C(1D)	B(1D)	1.612(2)
C(3D)	C(4D)	1.392(2)
C(3D)	C(8D)	1.394(2)
C(4D)	C(5D)	1.398(2)
C(5D)	C(6D)	1.389(2)
C(6D)	C(7D)	1.389(3)
C(7D)	C(8D)	1.385(3)
C(9D)	C(10D)	1.398(2)
C(9D)	C(14D)	1.393(2)
C(10D)	C(11D)	1.396(2)
C(11D)	C(12D)	1.383(2)
C(12D)	C(13D)	1.392(3)
C(13D)	C(14D)	1.391(2)
C(15D)	C(16D)	1.518(2)
C(17D)	C(18D)	1.516(2)
S(1F)	C(1F)	1.8075(15)
S(1F)	C(3F)	1.7716(16)
S(1F)	C(9F)	1.7623(16)
S(2F)	C(4F)	1.7609(16)
S(2F)	C(10F)	1.7579(16)
O(1F)	C(15F)	1.3338(19)
O(1F)	B(1F)	1.4664(19)
O(2F)	C(15F)	1.203(2)
O(3F)	C(18F)	1.3353(19)
O(3F)	B(1F)	1.4604(19)
O(4F)	C(18F)	1.1995(19)
N(1F)	C(16F)	1.4947(19)
N(1F)	C(17F)	1.5089(19)
N(1F)	C(19F)	1.4914(19)
N(1F)	B(1F)	1.644(2)
C(1F)	C(2F)	1.326(2)
C(1F)	B(1F)	1.610(2)
C(3F)	C(4F)	1.392(2)
C(3F)	C(8F)	1.392(2)
C(4F)	C(5F)	1.399(2)
C(5F)	C(6F)	1.387(2)
C(6F)	C(7F)	1.389(3)
C(7F)	C(8F)	1.386(2)
C(9F)	C(10F)	1.397(2)
C(9F)	C(14F)	1.393(2)
C(10F)	C(11F)	1.397(2)
C(11F)	C(12F)	1.381(2)
C(12F)	C(13F)	1.392(3)

Atom	Atom	Length/Å
C(13F)	C(14F)	1.386(3)
C(15F)	C(16F)	1.515(2)
C(17F)	C(18F)	1.519(2)
S(3)	C(20)	1.8271(18)
S(3)	O(5)	1.468(3)
S(3)	O(6)	1.407(3)
S(3)	O(7)	1.440(3)
S(3)	O(5A)	1.414(3)
S(3)	O(6A)	1.452(3)
S(3)	O(7A)	1.375(4)
C(20)	F(1)	1.268(3)
C(20)	F(2)	1.316(3)
C(20)	F(3)	1.335(3)
C(20)	F(1A)	1.379(3)
C(20)	F(2A)	1.308(4)
C(20)	F(3A)	1.367(3)
S(3B)	C(20B)	1.8258(19)
S(3B)	O(5B)	1.430(2)
S(3B)	O(6B)	1.4395(17)
S(3B)	O(7B)	1.446(2)
S(3B)	O(5C)	1.432(5)
S(3B)	O(6C)	1.390(5)
S(3B)	O(7C)	1.391(4)
C(20B)	F(1B)	1.292(2)
C(20B)	F(2B)	1.325(2)
C(20B)	F(3B)	1.307(2)
C(20B)	F(1C)	1.384(4)
C(20B)	F(2C)	1.326(4)
C(20B)	F(3C)	1.436(4)
S(3D)	O(5D)	1.4455(12)
S(3D)	O(6D)	1.4266(14)
S(3D)	O(7D)	1.4390(14)
S(3D)	C(20D)	1.8302(17)
F(1D)	C(20D)	1.3290(19)
F(2D)	C(20D)	1.3367(19)
F(3D)	C(20D)	1.3273(19)
S(3F)	O(5F)	1.4362(13)
S(3F)	O(6F)	1.4156(15)
S(3F)	O(7F)	1.4466(15)
S(3F)	C(20F)	1.8260(17)
F(1F)	C(20F)	1.325(2)
F(2F)	C(20F)	1.337(2)
F(3F)	C(20F)	1.319(2)

Table S24: Bond Angles in ° for **69.**

Atom	Atom	Atom	Angle/°
C(3)	S(1)	C(1)	104.20(7)
C(9)	S(1)	C(1)	103.94(7)
C(9)	S(1)	C(3)	101.31(7)
C(4)	S(2)	C(10)	101.83(7)
C(15)	O(1)	B(1)	112.45(12)
C(18)	O(3)	B(1)	113.75(12)
C(16)	N(1)	C(19)	110.50(12)
C(16)	N(1)	B(1)	101.57(11)
C(17)	N(1)	C(16)	112.24(12)
C(17)	N(1)	C(19)	110.89(12)
C(17)	N(1)	B(1)	102.75(11)
C(19)	N(1)	B(1)	118.45(12)

Atom	Atom	Atom	Angle/°
C(2)	C(1)	S(1)	118.49(12)
C(2)	C(1)	B(1)	124.94(14)
B(1)	C(1)	S(1)	115.93(11)
C(4)	C(3)	S(1)	121.52(12)
C(8)	C(3)	S(1)	116.76(12)
C(8)	C(3)	C(4)	121.70(15)
C(3)	C(4)	S(2)	123.20(12)
C(5)	C(4)	S(2)	118.22(12)
C(5)	C(4)	C(3)	118.58(14)
C(6)	C(5)	C(4)	119.76(15)
C(5)	C(6)	C(7)	120.89(16)
C(8)	C(7)	C(6)	119.74(15)

Atom	Atom	Atom	Angle/°
C(7)	C(8)	C(3)	119.13(15)
C(10)	C(9)	S(1)	120.55(12)
C(14)	C(9)	S(1)	117.56(12)
C(14)	C(9)	C(10)	121.83(15)
C(9)	C(10)	S(2)	124.34(12)
C(11)	C(10)	S(2)	117.43(12)
C(11)	C(10)	C(9)	118.22(14)
C(12)	C(11)	C(10)	120.12(15)
C(11)	C(12)	C(13)	121.21(16)
C(14)	C(13)	C(12)	119.55(15)
C(13)	C(14)	C(9)	119.02(16)
O(1)	C(15)	C(16)	110.31(12)
O(2)	C(15)	O(1)	124.39(14)
O(2)	C(15)	C(16)	125.30(14)
N(1)	C(16)	C(15)	106.86(12)
N(1)	C(17)	C(18)	104.98(12)
O(3)	C(18)	C(17)	109.47(13)
O(4)	C(18)	O(3)	124.36(14)
O(4)	C(18)	C(17)	126.11(15)
O(1)	B(1)	O(3)	111.84(12)
O(1)	B(1)	N(1)	102.90(11)
O(1)	B(1)	C(1)	110.18(12)
O(3)	B(1)	N(1)	101.15(11)
O(3)	B(1)	C(1)	114.02(12)
C(1)	B(1)	N(1)	116.01(12)
C(3B)	S(1B)	C(1B)	103.98(7)
C(9B)	S(1B)	C(1B)	103.89(7)
C(9B)	S(1B)	C(3B)	101.62(7)
C(4B)	S(2B)	C(10B)	102.04(7)
C(15B)	O(1B)	B(1B)	112.57(12)
C(18B)	O(3B)	B(1B)	113.86(12)
C(16B)	N(1B)	C(17B)	112.18(12)
C(16B)	N(1B)	C(19B)	110.48(12)
C(16B)	N(1B)	B(1B)	101.89(11)
C(17B)	N(1B)	C(19B)	110.71(12)
C(17B)	N(1B)	B(1B)	102.75(11)
C(19B)	N(1B)	B(1B)	118.41(12)
C(2B)	C(1B)	S(1B)	118.67(12)
C(2B)	C(1B)	B(1B)	124.56(14)
B(1B)	C(1B)	S(1B)	116.12(11)
C(4B)	C(3B)	S(1B)	121.75(12)
C(8B)	C(3B)	S(1B)	116.68(12)
C(8B)	C(3B)	C(4B)	121.55(14)
C(3B)	C(4B)	S(2B)	123.26(12)
C(5B)	C(4B)	S(2B)	118.18(12)
C(5B)	C(4B)	C(3B)	118.55(14)
C(6B)	C(5B)	C(4B)	119.86(15)
C(5B)	C(6B)	C(7B)	121.00(15)
C(8B)	C(7B)	C(6B)	119.68(15)
C(7B)	C(8B)	C(3B)	119.20(15)
C(10B)	C(9B)	S(1B)	120.82(12)
C(14B)	C(9B)	S(1B)	117.41(13)
C(14B)	C(9B)	C(10B)	121.70(15)
C(9B)	C(10B)	S(2B)	124.34(12)
C(11B)	C(10B)	S(2B)	117.38(12)
C(11B)	C(10B)	C(9B)	118.26(15)
C(12B)	C(11B)	C(10B)	120.15(16)
C(11B)	C(12B)	C(13B)	121.22(16)
C(14B)	C(13B)	C(12B)	119.45(16)
C(13B)	C(14B)	C(9B)	119.18(16)
O(1B)	C(15B)	C(16B)	110.47(13)

Atom	Atom	Atom	Angle/°
O(2B)	C(15B)	O(1B)	124.39(15)
O(2B)	C(15B)	C(16B)	125.13(14)
N(1B)	C(16B)	C(15B)	106.50(12)
N(1B)	C(17B)	C(18B)	104.74(12)
O(3B)	C(18B)	C(17B)	109.57(13)
O(4B)	C(18B)	O(3B)	124.41(15)
O(4B)	C(18B)	C(17B)	125.96(15)
O(1B)	B(1B)	O(3B)	111.82(13)
O(1B)	B(1B)	N(1B)	102.75(11)
O(1B)	B(1B)	C(1B)	110.20(12)
O(3B)	B(1B)	N(1B)	101.17(11)
O(3B)	B(1B)	C(1B)	114.19(13)
C(1B)	B(1B)	N(1B)	115.93(12)
C(3D)	S(1D)	C(1D)	104.95(7)
C(9D)	S(1D)	C(1D)	104.11(7)
C(9D)	S(1D)	C(3D)	102.13(7)
C(10D)	S(2D)	C(4D)	102.17(7)
C(15D)	O(1D)	B(1D)	114.83(12)
C(18D)	O(3D)	B(1D)	113.43(12)
C(16D)	N(1D)	C(17D)	111.84(12)
C(16D)	N(1D)	B(1D)	103.84(11)
C(17D)	N(1D)	B(1D)	102.24(11)
C(19D)	N(1D)	C(16D)	112.25(12)
C(19D)	N(1D)	C(17D)	109.41(12)
C(19D)	N(1D)	B(1D)	116.80(12)
C(2D)	C(1D)	S(1D)	118.85(12)
C(2D)	C(1D)	B(1D)	125.44(14)
B(1D)	C(1D)	S(1D)	115.21(11)
C(4D)	C(3D)	S(1D)	121.40(12)
C(4D)	C(3D)	C(8D)	121.63(15)
C(8D)	C(3D)	S(1D)	116.95(12)
C(3D)	C(4D)	S(2D)	123.57(12)
C(3D)	C(4D)	C(5D)	118.46(15)
C(5D)	C(4D)	S(2D)	117.96(12)
C(6D)	C(5D)	C(4D)	120.02(16)
C(7D)	C(6D)	C(5D)	120.73(16)
C(8D)	C(7D)	C(6D)	119.93(16)
C(7D)	C(8D)	C(3D)	119.10(16)
C(10D)	C(9D)	S(1D)	121.05(12)
C(14D)	C(9D)	S(1D)	117.00(12)
C(14D)	C(9D)	C(10D)	121.92(15)
C(9D)	C(10D)	S(2D)	123.92(12)
C(11D)	C(10D)	S(2D)	117.53(12)
C(11D)	C(10D)	C(9D)	118.54(14)
C(12D)	C(11D)	C(10D)	119.68(15)
C(11D)	C(12D)	C(13D)	121.46(15)
C(12D)	C(13D)	C(14D)	119.68(15)
C(13D)	C(14D)	C(9D)	118.67(15)
O(1D)	C(15D)	C(16D)	110.09(13)
O(2D)	C(15D)	O(1D)	123.55(15)
O(2D)	C(15D)	C(16D)	126.27(15)
N(1D)	C(16D)	C(15D)	105.64(12)
N(1D)	C(17D)	C(18D)	106.39(12)
O(3D)	C(18D)	C(17D)	110.90(13)
O(4D)	C(18D)	O(3D)	124.32(14)
O(4D)	C(18D)	C(17D)	124.76(14)
O(1D)	B(1D)	N(1D)	101.66(11)
O(1D)	B(1D)	C(1D)	111.97(13)
O(3D)	B(1D)	O(1D)	111.54(13)
O(3D)	B(1D)	N(1D)	103.68(11)
O(3D)	B(1D)	C(1D)	112.01(13)

Atom	Atom	Atom	Angle/°
C(1D)	B(1D)	N(1D)	115.32(12)
C(3F)	S(1F)	C(1F)	104.91(7)
C(9F)	S(1F)	C(1F)	104.08(7)
C(9F)	S(1F)	C(3F)	102.24(7)
C(10F)	S(2F)	C(4F)	102.16(7)
C(15F)	O(1F)	B(1F)	114.54(12)
C(18F)	O(3F)	B(1F)	113.49(12)
C(16F)	N(1F)	C(17F)	112.13(12)
C(16F)	N(1F)	B(1F)	103.82(11)
C(17F)	N(1F)	B(1F)	102.20(11)
C(19F)	N(1F)	C(16F)	111.87(12)
C(19F)	N(1F)	C(17F)	109.51(12)
C(19F)	N(1F)	B(1F)	116.91(12)
C(2F)	C(1F)	S(1F)	118.95(12)
C(2F)	C(1F)	B(1F)	125.34(14)
B(1F)	C(1F)	S(1F)	115.18(11)
C(4F)	C(3F)	S(1F)	121.59(12)
C(4F)	C(3F)	C(8F)	121.58(15)
C(8F)	C(3F)	S(1F)	116.82(12)
C(3F)	C(4F)	S(2F)	123.67(12)
C(3F)	C(4F)	C(5F)	118.42(14)
C(5F)	C(4F)	S(2F)	117.89(12)
C(6F)	C(5F)	C(4F)	120.02(15)
C(5F)	C(6F)	C(7F)	120.89(16)
C(8F)	C(7F)	C(6F)	119.69(16)
C(7F)	C(8F)	C(3F)	119.29(15)
C(10F)	C(9F)	S(1F)	121.33(12)
C(14F)	C(9F)	S(1F)	116.67(12)
C(14F)	C(9F)	C(10F)	121.96(15)
C(9F)	C(10F)	S(2F)	123.96(12)
C(11F)	C(10F)	S(2F)	117.69(12)
C(11F)	C(10F)	C(9F)	118.33(14)
C(12F)	C(11F)	C(10F)	119.77(16)
C(11F)	C(12F)	C(13F)	121.42(16)
C(14F)	C(13F)	C(12F)	119.64(15)
C(13F)	C(14F)	C(9F)	118.81(16)
O(1F)	C(15F)	C(16F)	110.31(13)
O(2F)	C(15F)	O(1F)	123.43(15)
O(2F)	C(15F)	C(16F)	126.18(15)
N(1F)	C(16F)	C(15F)	105.46(12)
N(1F)	C(17F)	C(18F)	106.41(12)
O(3F)	C(18F)	C(17F)	110.65(12)
O(4F)	C(18F)	O(3F)	124.43(14)
O(4F)	C(18F)	C(17F)	124.89(14)
O(1F)	B(1F)	N(1F)	101.63(11)
O(1F)	B(1F)	C(1F)	112.08(12)
O(3F)	B(1F)	O(1F)	111.42(13)
O(3F)	B(1F)	N(1F)	103.76(11)
O(3F)	B(1F)	C(1F)	111.91(13)
C(1F)	B(1F)	N(1F)	115.36(12)
O(5)	S(3)	C(20)	106.25(14)
O(6)	S(3)	C(20)	106.71(14)
O(6)	S(3)	O(5)	112.4(2)
O(6)	S(3)	O(7)	115.1(3)
O(7)	S(3)	C(20)	104.91(18)
O(7)	S(3)	O(5)	110.7(3)
O(5A)	S(3)	C(20)	98.91(15)
O(5A)	S(3)	O(6A)	112.7(3)
O(6A)	S(3)	C(20)	99.18(16)
O(7A)	S(3)	C(20)	102.6(2)
O(7A)	S(3)	O(5A)	120.6(3)

Atom	Atom	Atom	Angle/°
O(7A)	S(3)	O(6A)	117.3(3)
F(1)	C(20)	S(3)	111.08(17)
F(1)	C(20)	F(2)	111.0(3)
F(1)	C(20)	F(3)	111.0(2)
F(2)	C(20)	S(3)	109.9(2)
F(2)	C(20)	F(3)	106.8(2)
F(3)	C(20)	S(3)	106.83(16)
F(1A)	C(20)	S(3)	113.04(18)
F(2A)	C(20)	S(3)	116.8(2)
F(2A)	C(20)	F(1A)	104.6(3)
F(2A)	C(20)	F(3A)	105.7(3)
F(3A)	C(20)	S(3)	115.21(15)
F(3A)	C(20)	F(1A)	99.6(2)
O(5B)	S(3B)	C(20B)	106.09(11)
O(5B)	S(3B)	O(6B)	113.66(16)
O(5B)	S(3B)	O(7B)	113.84(18)
O(6B)	S(3B)	C(20B)	104.67(9)
O(6B)	S(3B)	O(7B)	112.17(15)
O(7B)	S(3B)	C(20B)	105.39(10)
O(5C)	S(3B)	C(20B)	95.2(2)
O(6C)	S(3B)	C(20B)	98.4(2)
O(6C)	S(3B)	O(5C)	116.8(6)
O(6C)	S(3B)	O(7C)	122.2(5)
O(7C)	S(3B)	C(20B)	95.9(3)
O(7C)	S(3B)	O(5C)	117.2(5)
F(1B)	C(20B)	S(3B)	111.21(13)
F(1B)	C(20B)	F(2B)	109.25(19)
F(1B)	C(20B)	F(3B)	110.19(19)
F(2B)	C(20B)	S(3B)	108.96(13)
F(3B)	C(20B)	S(3B)	109.02(13)
F(3B)	C(20B)	F(2B)	108.15(17)
F(1C)	C(20B)	S(3B)	117.3(2)
F(1C)	C(20B)	F(3C)	93.8(3)
F(2C)	C(20B)	S(3B)	123.5(2)
F(2C)	C(20B)	F(1C)	103.1(4)
F(2C)	C(20B)	F(3C)	98.7(4)
F(3C)	C(20B)	S(3B)	115.04(19)
O(5D)	S(3D)	C(20D)	102.46(7)
O(6D)	S(3D)	O(5D)	115.91(9)
O(6D)	S(3D)	O(7D)	116.30(10)
O(6D)	S(3D)	C(20D)	103.43(8)
O(7D)	S(3D)	O(5D)	112.89(9)
O(7D)	S(3D)	C(20D)	103.33(8)
F(1D)	C(20D)	S(3D)	111.97(11)
F(1D)	C(20D)	F(2D)	106.65(14)
F(2D)	C(20D)	S(3D)	111.61(11)
F(3D)	C(20D)	S(3D)	111.95(11)
F(3D)	C(20D)	F(1D)	107.51(13)
F(3D)	C(20D)	F(2D)	106.84(14)
O(5F)	S(3F)	O(7F)	112.17(9)
O(5F)	S(3F)	C(20F)	102.70(8)
O(6F)	S(3F)	O(5F)	117.19(11)
O(6F)	S(3F)	O(7F)	115.80(12)
O(6F)	S(3F)	C(20F)	103.17(9)
O(7F)	S(3F)	C(20F)	103.26(9)
F(1F)	C(20F)	S(3F)	112.31(12)
F(1F)	C(20F)	F(2F)	106.04(15)
F(2F)	C(20F)	S(3F)	111.16(11)
F(3F)	C(20F)	S(3F)	112.39(11)
F(3F)	C(20F)	F(1F)	107.85(14)
F(3F)	C(20F)	F(2F)	106.72(15)

Table S25: Torsion Angles in ° for **69**.

Atom	Atom	Atom	Atom	Angle/°
S(1)	C(1)	B(1)	O(1)	-119.71(12)
S(1)	C(1)	B(1)	O(3)	7.00(16)
S(1)	C(1)	B(1)	N(1)	123.94(12)
S(1)	C(3)	C(4)	S(2)	-5.10(19)
S(1)	C(3)	C(4)	C(5)	174.43(12)
S(1)	C(3)	C(8)	C(7)	-173.92(12)
S(1)	C(9)	C(10)	S(2)	3.49(19)
S(1)	C(9)	C(10)	C(11)	-175.34(11)
S(1)	C(9)	C(14)	C(13)	175.50(13)
S(2)	C(4)	C(5)	C(6)	179.75(12)
S(2)	C(10)	C(11)	C(12)	-179.11(12)
O(1)	C(15)	C(16)	N(1)	7.61(17)
O(2)	C(15)	C(16)	N(1)	-173.01(15)
N(1)	C(17)	C(18)	O(3)	22.08(16)
N(1)	C(17)	C(18)	O(4)	-160.54(15)
C(1)	S(1)	C(3)	C(4)	-64.53(14)
C(1)	S(1)	C(3)	C(8)	114.04(13)
C(1)	S(1)	C(9)	C(10)	65.91(14)
C(1)	S(1)	C(9)	C(14)	-111.42(13)
C(2)	C(1)	B(1)	O(1)	50.93(19)
C(2)	C(1)	B(1)	O(3)	177.64(14)
C(2)	C(1)	B(1)	N(1)	-65.42(19)
C(3)	S(1)	C(1)	C(2)	119.97(13)
C(3)	S(1)	C(1)	B(1)	-68.75(12)
C(3)	S(1)	C(9)	C(10)	-41.99(14)
C(3)	S(1)	C(9)	C(14)	140.67(13)
C(3)	C(4)	C(5)	C(6)	0.2(2)
C(4)	S(2)	C(10)	C(9)	35.42(14)
C(4)	S(2)	C(10)	C(11)	-145.74(12)
C(4)	C(3)	C(8)	C(7)	4.6(2)
C(4)	C(5)	C(6)	C(7)	3.0(2)
C(5)	C(6)	C(7)	C(8)	-2.5(3)
C(6)	C(7)	C(8)	C(3)	-1.3(2)
C(8)	C(3)	C(4)	S(2)	176.40(12)
C(8)	C(3)	C(4)	C(5)	-4.1(2)
C(9)	S(1)	C(1)	C(2)	14.24(14)
C(9)	S(1)	C(1)	B(1)	-174.49(10)
C(9)	S(1)	C(3)	C(4)	43.17(14)
C(9)	S(1)	C(3)	C(8)	-138.26(13)
C(9)	C(10)	C(11)	C(12)	-0.2(2)
C(10)	S(2)	C(4)	C(3)	-34.25(14)
C(10)	S(2)	C(4)	C(5)	146.22(12)
C(10)	C(9)	C(14)	C(13)	-1.8(2)
C(10)	C(11)	C(12)	C(13)	-1.6(2)
C(11)	C(12)	C(13)	C(14)	1.6(3)
C(12)	C(13)	C(14)	C(9)	0.0(2)
C(14)	C(9)	C(10)	S(2)	-179.29(12)
C(14)	C(9)	C(10)	C(11)	1.9(2)
C(15)	O(1)	B(1)	O(3)	86.73(15)
C(15)	O(1)	B(1)	N(1)	-21.06(15)
C(15)	O(1)	B(1)	C(1)	-145.36(12)
C(16)	N(1)	C(17)	C(18)	80.82(15)
C(16)	N(1)	B(1)	O(1)	23.72(14)
C(16)	N(1)	B(1)	O(3)	-92.01(12)
C(16)	N(1)	B(1)	C(1)	144.09(13)
C(17)	N(1)	C(16)	C(15)	-127.94(13)
C(17)	N(1)	B(1)	O(1)	139.99(12)

Atom	Atom	Atom	Atom	Angle/°
C(17)	N(1)	B(1)	O(3)	24.26(14)
C(17)	N(1)	B(1)	C(1)	-99.65(14)
C(18)	O(3)	B(1)	O(1)	-120.83(13)
C(18)	O(3)	B(1)	N(1)	-11.93(15)
C(18)	O(3)	B(1)	C(1)	113.33(14)
C(19)	N(1)	C(16)	C(15)	107.71(14)
C(19)	N(1)	C(17)	C(18)	-155.05(12)
C(19)	N(1)	B(1)	O(1)	-97.44(15)
C(19)	N(1)	B(1)	O(3)	146.83(13)
C(19)	N(1)	B(1)	C(1)	22.92(19)
B(1)	O(1)	C(15)	O(2)	-169.75(15)
B(1)	O(1)	C(15)	C(16)	9.64(17)
B(1)	O(3)	C(18)	O(4)	177.29(15)
B(1)	O(3)	C(18)	C(17)	-5.27(17)
B(1)	N(1)	C(16)	C(15)	-18.85(15)
B(1)	N(1)	C(17)	C(18)	-27.53(15)
S(1B)	C(1B)	B(1B)	O(1B)	-120.59(12)
S(1B)	C(1B)	B(1B)	O(3B)	6.24(16)
S(1B)	C(1B)	B(1B)	N(1B)	123.28(12)
S(1B)	C(3B)	C(4B)	S(2B)	-4.1(2)
S(1B)	C(3B)	C(4B)	C(5B)	174.84(12)
S(1B)	C(3B)	C(8B)	C(7B)	-174.29(12)
S(1B)	C(9B)	C(10B)	S(2B)	3.7(2)
S(1B)	C(9B)	C(10B)	C(11B)	-174.83(12)
S(1B)	C(9B)	C(14B)	C(13B)	175.49(12)
S(2B)	C(4B)	C(5B)	C(6B)	179.19(12)
S(2B)	C(10B)	C(11B)	C(12B)	-179.21(12)
O(1B)	C(15B)	C(16B)	N(1B)	8.31(17)
O(2B)	C(15B)	C(16B)	N(1B)	-172.70(15)
N(1B)	C(17B)	C(18B)	O(3B)	22.16(17)
N(1B)	C(17B)	C(18B)	O(4B)	-160.62(15)
C(1B)	S(1B)	C(3B)	C(4B)	-65.88(14)
C(1B)	S(1B)	C(3B)	C(8B)	112.73(13)
C(1B)	S(1B)	C(9B)	C(10B)	66.49(14)
C(1B)	S(1B)	C(9B)	C(14B)	-110.45(13)
C(2B)	C(1B)	B(1B)	O(1B)	50.0(2)
C(2B)	C(1B)	B(1B)	O(3B)	176.84(14)
C(2B)	C(1B)	B(1B)	N(1B)	-66.1(2)
C(3B)	S(1B)	C(1B)	C(2B)	119.37(13)
C(3B)	S(1B)	C(1B)	B(1B)	-69.45(12)
C(3B)	S(1B)	C(9B)	C(10B)	-41.27(14)
C(3B)	S(1B)	C(9B)	C(14B)	141.79(13)
C(3B)	C(4B)	C(5B)	C(6B)	0.2(2)
C(4B)	S(2B)	C(10B)	C(9B)	34.76(15)
C(4B)	S(2B)	C(10B)	C(11B)	-146.73(12)
C(4B)	C(3B)	C(8B)	C(7B)	4.3(2)
C(4B)	C(5B)	C(6B)	C(7B)	2.7(2)
C(5B)	C(6B)	C(7B)	C(8B)	-2.1(2)
C(6B)	C(7B)	C(8B)	C(3B)	-1.4(2)
C(8B)	C(3B)	C(4B)	S(2B)	177.33(12)
C(8B)	C(3B)	C(4B)	C(5B)	-3.7(2)
C(9B)	S(1B)	C(1B)	C(2B)	13.39(14)
C(9B)	S(1B)	C(1B)	B(1B)	-175.43(11)
C(9B)	S(1B)	C(3B)	C(4B)	41.81(14)
C(9B)	S(1B)	C(3B)	C(8B)	-139.59(13)
C(9B)	C(10B)	C(11B)	C(12B)	-0.6(2)
C(10B)	S(2B)	C(4B)	C(3B)	-34.23(15)
C(10B)	S(2B)	C(4B)	C(5B)	146.79(13)
C(10B)	C(9B)	C(14B)	C(13B)	-1.4(2)
C(10B)	C(11B)	C(12B)	C(13B)	-1.3(2)
C(11B)	C(12B)	C(13B)	C(14B)	1.9(3)

Atom	Atom	Atom	Atom	Angle/°
C(12B)	C(13B)	C(14B)	C(9B)	-0.5(2)
C(14B)	C(9B)	C(10B)	S(2B)	-179.53(12)
C(14B)	C(9B)	C(10B)	C(11B)	2.0(2)
C(15B)	O(1B)	B(1B)	O(3B)	87.37(15)
C(15B)	O(1B)	B(1B)	N(1B)	-20.36(16)
C(15B)	O(1B)	B(1B)	C(1B)	-144.49(13)
C(16B)	N(1B)	C(17B)	C(18B)	81.18(15)
C(16B)	N(1B)	B(1B)	O(1B)	23.57(14)
C(16B)	N(1B)	B(1B)	O(3B)	-92.10(13)
C(16B)	N(1B)	B(1B)	C(1B)	143.82(13)
C(17B)	N(1B)	C(16B)	C(15B)	-128.37(13)
C(17B)	N(1B)	B(1B)	O(1B)	139.88(12)
C(17B)	N(1B)	B(1B)	O(3B)	24.21(14)
C(17B)	N(1B)	B(1B)	C(1B)	-99.87(14)
C(18B)	O(3B)	B(1B)	O(1B)	-120.56(14)
C(18B)	O(3B)	B(1B)	N(1B)	-11.81(15)
C(18B)	O(3B)	B(1B)	C(1B)	113.45(14)
C(19B)	N(1B)	C(16B)	C(15B)	107.58(14)
C(19B)	N(1B)	C(17B)	C(18B)	-154.89(13)
C(19B)	N(1B)	B(1B)	O(1B)	-97.80(15)
C(19B)	N(1B)	B(1B)	O(3B)	146.54(13)
C(19B)	N(1B)	B(1B)	C(1B)	22.45(19)
B(1B)	O(1B)	C(15B)	O(2B)	-170.24(15)
B(1B)	O(1B)	C(15B)	C(16B)	8.76(17)
B(1B)	O(3B)	C(18B)	O(4B)	177.28(15)
B(1B)	O(3B)	C(18B)	C(17B)	-5.45(17)
B(1B)	N(1B)	C(16B)	C(15B)	-19.13(15)
B(1B)	N(1B)	C(17B)	C(18B)	-27.51(15)
S(1D)	C(1D)	B(1D)	O(1D)	-117.61(12)
S(1D)	C(1D)	B(1D)	O(3D)	8.55(16)
S(1D)	C(1D)	B(1D)	N(1D)	126.80(11)
S(1D)	C(3D)	C(4D)	S(2D)	-2.6(2)
S(1D)	C(3D)	C(4D)	C(5D)	176.12(12)
S(1D)	C(3D)	C(8D)	C(7D)	-174.41(13)
S(1D)	C(9D)	C(10D)	S(2D)	3.05(19)
S(1D)	C(9D)	C(10D)	C(11D)	-175.53(11)
S(1D)	C(9D)	C(14D)	C(13D)	175.73(12)
S(2D)	C(4D)	C(5D)	C(6D)	177.70(13)
S(2D)	C(10D)	C(11D)	C(12D)	-179.15(12)
O(1D)	C(15D)	C(16D)	N(1D)	-15.83(17)
O(2D)	C(15D)	C(16D)	N(1D)	167.46(15)
N(1D)	C(17D)	C(18D)	O(3D)	-13.53(17)
N(1D)	C(17D)	C(18D)	O(4D)	168.12(14)
C(1D)	S(1D)	C(3D)	C(4D)	-67.94(14)
C(1D)	S(1D)	C(3D)	C(8D)	110.43(13)
C(1D)	S(1D)	C(9D)	C(10D)	68.51(14)
C(1D)	S(1D)	C(9D)	C(14D)	-109.41(13)
C(2D)	C(1D)	B(1D)	O(1D)	54.1(2)
C(2D)	C(1D)	B(1D)	O(3D)	-179.74(15)
C(2D)	C(1D)	B(1D)	N(1D)	-61.5(2)
C(3D)	S(1D)	C(1D)	C(2D)	119.93(13)
C(3D)	S(1D)	C(1D)	B(1D)	-67.78(12)
C(3D)	S(1D)	C(9D)	C(10D)	-40.53(14)
C(3D)	S(1D)	C(9D)	C(14D)	141.56(13)
C(3D)	C(4D)	C(5D)	C(6D)	-1.1(2)
C(4D)	S(2D)	C(10D)	C(9D)	35.18(15)
C(4D)	S(2D)	C(10D)	C(11D)	-146.23(12)
C(4D)	C(3D)	C(8D)	C(7D)	4.0(2)
C(4D)	C(5D)	C(6D)	C(7D)	2.6(3)
C(5D)	C(6D)	C(7D)	C(8D)	-0.8(3)
C(6D)	C(7D)	C(8D)	C(3D)	-2.4(3)

Atom	Atom	Atom	Atom	Angle/°
C(8D)	C(3D)	C(4D)	S(2D)	179.08(12)
C(8D)	C(3D)	C(4D)	C(5D)	-2.2(2)
C(9D)	S(1D)	C(1D)	C(2D)	13.00(15)
C(9D)	S(1D)	C(1D)	B(1D)	-174.71(11)
C(9D)	S(1D)	C(3D)	C(4D)	40.45(15)
C(9D)	S(1D)	C(3D)	C(8D)	-141.18(13)
C(9D)	C(10D)	C(11D)	C(12D)	-0.5(2)
C(10D)	S(2D)	C(4D)	C(3D)	-35.28(15)
C(10D)	S(2D)	C(4D)	C(5D)	145.97(13)
C(10D)	C(9D)	C(14D)	C(13D)	-2.2(2)
C(10D)	C(11D)	C(12D)	C(13D)	-1.4(2)
C(11D)	C(12D)	C(13D)	C(14D)	1.5(2)
C(12D)	C(13D)	C(14D)	C(9D)	0.2(2)
C(14D)	C(9D)	C(10D)	S(2D)	-179.14(12)
C(14D)	C(9D)	C(10D)	C(11D)	2.3(2)
C(15D)	O(1D)	B(1D)	O(3D)	118.25(14)
C(15D)	O(1D)	B(1D)	N(1D)	8.31(16)
C(15D)	O(1D)	B(1D)	C(1D)	-115.33(14)
C(16D)	N(1D)	C(17D)	C(18D)	128.17(13)
C(16D)	N(1D)	B(1D)	O(1D)	-17.15(15)
C(16D)	N(1D)	B(1D)	O(3D)	-133.00(12)
C(16D)	N(1D)	B(1D)	C(1D)	104.19(14)
C(17D)	N(1D)	C(16D)	C(15D)	-89.99(14)
C(17D)	N(1D)	B(1D)	O(1D)	99.31(13)
C(17D)	N(1D)	B(1D)	O(3D)	-16.54(14)
C(17D)	N(1D)	B(1D)	C(1D)	-139.34(13)
C(18D)	O(3D)	B(1D)	O(1D)	-99.21(14)
C(18D)	O(3D)	B(1D)	N(1D)	9.42(16)
C(18D)	O(3D)	B(1D)	C(1D)	134.39(13)
C(19D)	N(1D)	C(16D)	C(15D)	146.59(13)
C(19D)	N(1D)	C(17D)	C(18D)	-106.82(14)
C(19D)	N(1D)	B(1D)	O(1D)	-141.30(13)
C(19D)	N(1D)	B(1D)	O(3D)	102.85(14)
C(19D)	N(1D)	B(1D)	C(1D)	-19.96(18)
B(1D)	O(1D)	C(15D)	O(2D)	-179.20(15)
B(1D)	O(1D)	C(15D)	C(16D)	3.98(18)
B(1D)	O(3D)	C(18D)	O(4D)	-179.73(15)
B(1D)	O(3D)	C(18D)	C(17D)	1.90(17)
B(1D)	N(1D)	C(16D)	C(15D)	19.53(15)
B(1D)	N(1D)	C(17D)	C(18D)	17.64(15)
S(1F)	C(1F)	B(1F)	O(1F)	-118.05(12)
S(1F)	C(1F)	B(1F)	O(3F)	7.97(16)
S(1F)	C(1F)	B(1F)	N(1F)	126.29(11)
S(1F)	C(3F)	C(4F)	S(2F)	-2.1(2)
S(1F)	C(3F)	C(4F)	C(5F)	176.47(12)
S(1F)	C(3F)	C(8F)	C(7F)	-175.21(13)
S(1F)	C(9F)	C(10F)	S(2F)	3.21(19)
S(1F)	C(9F)	C(10F)	C(11F)	-175.25(12)
S(1F)	C(9F)	C(14F)	C(13F)	175.53(12)
S(2F)	C(4F)	C(5F)	C(6F)	177.99(13)
S(2F)	C(10F)	C(11F)	C(12F)	-178.89(12)
O(1F)	C(15F)	C(16F)	N(1F)	-16.05(17)
O(2F)	C(15F)	C(16F)	N(1F)	167.18(15)
N(1F)	C(17F)	C(18F)	O(3F)	-13.38(17)
N(1F)	C(17F)	C(18F)	O(4F)	168.18(15)
C(1F)	S(1F)	C(3F)	C(4F)	-68.79(14)
C(1F)	S(1F)	C(3F)	C(8F)	110.09(13)
C(1F)	S(1F)	C(9F)	C(10F)	68.93(14)
C(1F)	S(1F)	C(9F)	C(14F)	-108.69(13)
C(2F)	C(1F)	B(1F)	O(1F)	53.5(2)
C(2F)	C(1F)	B(1F)	O(3F)	179.55(15)

Atom	Atom	Atom	Atom	Angle/˚
C(2F)	C(1F)	B(1F)	N(1F)	-62.1(2)
C(3F)	S(1F)	C(1F)	C(2F)	119.33(13)
C(3F)	S(1F)	C(1F)	B(1F)	-68.52(12)
C(3F)	S(1F)	C(9F)	C(10F)	-40.09(14)
C(3F)	S(1F)	C(9F)	C(14F)	142.29(13)
C(3F)	C(4F)	C(5F)	C(6F)	-0.6(2)
C(4F)	S(2F)	C(10F)	C(9F)	34.65(15)
C(4F)	S(2F)	C(10F)	C(11F)	-146.88(12)
C(4F)	C(3F)	C(8F)	C(7F)	3.7(2)
C(4F)	C(5F)	C(6F)	C(7F)	2.3(3)
C(5F)	C(6F)	C(7F)	C(8F)	-1.0(3)
C(6F)	C(7F)	C(8F)	C(3F)	-1.9(2)
C(8F)	C(3F)	C(4F)	S(2F)	179.09(12)
C(8F)	C(3F)	C(4F)	C(5F)	-2.4(2)
C(9F)	S(1F)	C(1F)	C(2F)	12.29(15)
C(9F)	S(1F)	C(1F)	B(1F)	-175.56(11)
C(9F)	S(1F)	C(3F)	C(4F)	39.60(14)
C(9F)	S(1F)	C(3F)	C(8F)	-141.53(13)
C(9F)	C(10F)	C(11F)	C(12F)	-0.3(2)
C(10F)	S(2F)	C(4F)	C(3F)	-35.14(15)
C(10F)	S(2F)	C(4F)	C(5F)	146.30(13)
C(10F)	C(9F)	C(14F)	C(13F)	-2.1(2)
C(10F)	C(11F)	C(12F)	C(13F)	-1.7(2)
C(11F)	C(12F)	C(13F)	C(14F)	1.9(3)
C(12F)	C(13F)	C(14F)	C(9F)	0.0(2)
C(14F)	C(9F)	C(10F)	S(2F)	-179.29(12)
C(14F)	C(9F)	C(10F)	C(11F)	2.2(2)
C(15F)	O(1F)	B(1F)	O(3F)	119.07(14)
C(15F)	O(1F)	B(1F)	N(1F)	9.09(16)
C(15F)	O(1F)	B(1F)	C(1F)	-114.64(14)
C(16F)	N(1F)	C(17F)	C(18F)	128.43(13)
C(16F)	N(1F)	B(1F)	O(1F)	-18.01(14)
C(16F)	N(1F)	B(1F)	O(3F)	-133.75(12)
C(16F)	N(1F)	B(1F)	C(1F)	103.48(14)
C(17F)	N(1F)	C(16F)	C(15F)	-89.38(14)
C(17F)	N(1F)	B(1F)	O(1F)	98.75(13)
C(17F)	N(1F)	B(1F)	O(3F)	-16.99(14)
C(17F)	N(1F)	B(1F)	C(1F)	-139.76(13)
C(18F)	O(3F)	B(1F)	O(1F)	-98.61(14)
C(18F)	O(3F)	B(1F)	N(1F)	10.00(16)
C(18F)	O(3F)	B(1F)	C(1F)	135.01(13)
C(19F)	N(1F)	C(16F)	C(15F)	147.13(13)
C(19F)	N(1F)	C(17F)	C(18F)	-106.77(14)
C(19F)	N(1F)	B(1F)	O(1F)	-141.71(13)
C(19F)	N(1F)	B(1F)	O(3F)	102.54(14)
C(19F)	N(1F)	B(1F)	C(1F)	-20.23(18)
B(1F)	O(1F)	C(15F)	O(2F)	-179.52(15)
B(1F)	O(1F)	C(15F)	C(16F)	3.61(17)
B(1F)	O(3F)	C(18F)	O(4F)	179.88(15)
B(1F)	O(3F)	C(18F)	C(17F)	1.43(17)
B(1F)	N(1F)	C(16F)	C(15F)	20.19(15)
B(1F)	N(1F)	C(17F)	C(18F)	17.83(15)
O(5)	S(3)	C(20)	F(1)	-179.3(3)
O(5)	S(3)	C(20)	F(2)	-56.0(3)
O(5)	S(3)	C(20)	F(3)	59.5(3)
O(6)	S(3)	C(20)	F(1)	60.6(3)
O(6)	S(3)	C(20)	F(2)	-176.2(3)
O(6)	S(3)	C(20)	F(3)	-60.6(2)
O(7)	S(3)	C(20)	F(1)	-62.0(3)
O(7)	S(3)	C(20)	F(2)	61.3(3)
O(7)	S(3)	C(20)	F(3)	176.8(3)

Atom	Atom	Atom	Atom	Angle/°
O(5A)	S(3)	C(20)	F(1A)	175.8(3)
O(5A)	S(3)	C(20)	F(2A)	-62.8(3)
O(5A)	S(3)	C(20)	F(3A)	62.2(3)
O(6A)	S(3)	C(20)	F(1A)	60.9(3)
O(6A)	S(3)	C(20)	F(2A)	-177.7(3)
O(6A)	S(3)	C(20)	F(3A)	-52.7(3)
O(7A)	S(3)	C(20)	F(1A)	-60.0(3)
O(7A)	S(3)	C(20)	F(2A)	61.4(3)
O(7A)	S(3)	C(20)	F(3A)	-173.6(3)
O(5B)	S(3B)	C(20B)	F(1B)	-176.6(2)
O(5B)	S(3B)	C(20B)	F(2B)	-56.1(2)
O(5B)	S(3B)	C(20B)	F(3B)	61.7(2)
O(6B)	S(3B)	C(20B)	F(1B)	62.95(19)
O(6B)	S(3B)	C(20B)	F(2B)	-176.57(17)
O(6B)	S(3B)	C(20B)	F(3B)	-58.74(17)
O(7B)	S(3B)	C(20B)	F(1B)	-55.5(2)
O(7B)	S(3B)	C(20B)	F(2B)	65.0(2)
O(7B)	S(3B)	C(20B)	F(3B)	-177.21(19)
O(5C)	S(3B)	C(20B)	F(1C)	173.9(5)
O(5C)	S(3B)	C(20B)	F(2C)	-55.7(5)
O(5C)	S(3B)	C(20B)	F(3C)	65.0(4)
O(6C)	S(3B)	C(20B)	F(1C)	55.8(5)
O(6C)	S(3B)	C(20B)	F(2C)	-173.8(6)
O(6C)	S(3B)	C(20B)	F(3C)	-53.1(5)
O(7C)	S(3B)	C(20B)	F(1C)	-68.1(5)
O(7C)	S(3B)	C(20B)	F(2C)	62.4(5)
O(7C)	S(3B)	C(20B)	F(3C)	-176.9(4)
O(5D)	S(3D)	C(20D)	F(1D)	179.30(12)
O(5D)	S(3D)	C(20D)	F(2D)	-61.22(14)
O(5D)	S(3D)	C(20D)	F(3D)	58.48(13)
O(6D)	S(3D)	C(20D)	F(1D)	58.45(14)
O(6D)	S(3D)	C(20D)	F(2D)	177.93(13)
O(6D)	S(3D)	C(20D)	F(3D)	-62.37(14)
O(7D)	S(3D)	C(20D)	F(1D)	-63.20(14)
O(7D)	S(3D)	C(20D)	F(2D)	56.28(14)
O(7D)	S(3D)	C(20D)	F(3D)	175.98(12)
O(5F)	S(3F)	C(20F)	F(1F)	-179.92(13)
O(5F)	S(3F)	C(20F)	F(2F)	-61.29(15)
O(5F)	S(3F)	C(20F)	F(3F)	58.25(14)
O(6F)	S(3F)	C(20F)	F(1F)	57.81(16)
O(6F)	S(3F)	C(20F)	F(2F)	176.44(15)
O(6F)	S(3F)	C(20F)	F(3F)	-64.02(16)
O(7F)	S(3F)	C(20F)	F(1F)	-63.14(15)
O(7F)	S(3F)	C(20F)	F(2F)	55.49(15)
O(7F)	S(3F)	C(20F)	F(3F)	175.03(13)

Table S26: Hydrogen Fractional Atomic Coordinates (×10⁴) and Equivalent Isotropic Displacement Parameters (Å^2×10^3) for **69**. U_{eq} is defined as 1/3 of the trace of the orthogonalised U_{ij}.

Atom	x	y	z	U_{eq}
H(2A)	2823.79	8936.5	3858.99	23
H(2B)	2913.44	9675.13	4122.39	23
H(5)	-1322.57	9894.66	4486.51	22
H(6)	-1945.22	8875.04	4806.61	28
H(7)	-771.43	8000.79	5098.87	29
H(8)	1036.55	8087.06	5044.78	25
H(11)	1780.36	11803.09	4364.71	22
H(12)	3428.68	12203.03	4589.5	28
H(13)	4531.02	11258.53	4869.56	29

Atom	x	y	z	U_{eq}
H(14)	4005.1	9870.35	4905.04	27
H(16A)	1076.61	6845.46	3529.64	24
H(16B)	808.14	6327.94	3830.02	24
H(17A)	-530.15	7096.44	3984.05	23
H(17B)	-346.64	8010.37	4128.46	23
H(19A)	1335.76	8322.76	3557.77	34
H(19B)	589.96	8782.77	3781.26	34
H(19C)	103.06	8119.63	3532.91	34
H(2BA)	2203.39	3882.65	3648.31	24
H(2BB)	2115.93	4627.8	3387.5	24
H(5B)	6343.34	4910.03	3026.75	23
H(6B)	6996.74	3891.44	2713.76	26
H(7B)	5855.39	2984.85	2427.13	27
H(8B)	4043.08	3048.36	2476.04	23
H(11B)	3186.18	6771.96	3149.52	24
H(12B)	1528.95	7146.76	2924.28	30
H(13B)	455.24	6187.99	2641.55	31
H(14B)	1017.22	4807.33	2607.16	27
H(16C)	3974.01	1811.55	3978.38	25
H(16D)	4229.49	1288.64	3677.83	25
H(17C)	5566.87	2050.33	3519.81	25
H(17D)	5384.64	2963.52	3374.42	25
H(19D)	3716.12	3286.22	3948.79	35
H(19E)	4448.14	3744.85	3720.99	35
H(19F)	4949.21	3086.41	3969	35
H(2DA)	2816.84	8717.75	6404.53	25
H(2DB)	2887.28	9477.81	6659.23	25
H(5D)	-1439.81	9921.77	6960.49	26
H(6D)	-2200.45	8917.94	7257.6	31
H(7D)	-1150.13	7958.04	7546.78	33
H(8D)	679.84	7982.07	7531.95	28
H(11D)	1806.02	11690.08	6870.45	21
H(12D)	3446.43	12028.01	7117.35	26
H(13D)	4442.84	11052.42	7414.69	28
H(14D)	3816.95	9686.95	7449.41	25
H(16E)	1683.56	7496.35	6031.2	25
H(16F)	1071.67	6631.94	6060.75	25
H(17E)	230.65	6260.24	6455.51	23
H(17F)	-622.95	6987.33	6475.03	23
H(19G)	792.54	8599.69	6239.32	37
H(19H)	-60.71	8434.73	6481.18	37
H(19I)	-228.25	8052.11	6138.79	37
H(2FA)	2157.48	3672.6	6098.33	25
H(2FB)	2100.55	4436.46	5845.31	25
H(5F)	6429.95	4919.06	5559.2	25
H(6F)	7217.34	3923.19	5266.13	30
H(7F)	6194.84	2949.13	4975.16	30
H(8F)	4362.57	2948.91	4987.7	26
H(11F)	3133.81	6657.9	5634.22	24
H(12F)	1488.87	6966.65	5384.9	29
H(13F)	530.98	5975.78	5083.81	31
H(14F)	1191.62	4623.44	5053.4	26
H(16G)	3286.29	2470.18	6472.76	24
H(16H)	3894.79	1602.03	6448.93	24
H(17G)	4735.71	1212.57	6052.3	22
H(17H)	5599.96	1932.23	6034.12	22
H(19J)	4198.19	3561.55	6262.79	35
H(19K)	5068.2	3378.58	6027.8	35
H(19L)	5202.93	3004.81	6371.94	35

Table S27: Atomic Occupancies for all atoms that are not fully occupied in **69**.

Atom	Occupancy
F(1)	0.521(6)
F(2)	0.521(6)
F(3)	0.521(6)
O(5)	0.521(6)
O(6)	0.521(6)
O(7)	0.521(6)
F(1A)	0.479(6)
F(2A)	0.479(6)
F(3A)	0.479(6)
O(5A)	0.479(6)
O(6A)	0.479(6)
O(7A)	0.479(6)
F(1B)	0.734(3)
F(2B)	0.734(3)
F(3B)	0.734(3)
O(5B)	0.734(3)
O(6B)	0.734(3)
O(7B)	0.734(3)
F(1C)	0.266(3)
F(2C)	0.266(3)
F(3C)	0.266(3)
O(5C)	0.266(3)
O(6C)	0.266(3)
O(7C)	0.266(3)

7. References

(1). Li, J.; Burke, M. D., Pinene-Derived Iminodiacetic Acid (PIDA): A Powerful Ligand for Stereoselective Synthesis and Iterative Cross-Coupling of C(sp3) Boronate Building Blocks. *Journal of the American Chemical Society* **2011,** *133* (35), 13774-13777.

(2). Speckmeier, E.; Maier, T. C., ART─An Amino Radical Transfer Strategy for C(sp2)–C(sp3) Coupling Reactions, Enabled by Dual Photo/Nickel Catalysis. *Journal of the American Chemical Society* **2022,** *144* (22), 9997-10005

(3). Lima, F.; Sharma, U. K.; Grunenberg, L.; Saha, D.; Johannsen, S.; Sedelmeier, J.; Van der Eycken, E. V.; Ley, S. V., A Lewis Base Catalysis Approach for the Photoredox Activation of Boronic Acids and Esters. *Angewandte Chemie International Edition* **2017,** *56* (47), 15136-15140.

8. Copies of ^{1}H, ^{13}C, ^{11}B and ^{19}F NMR Spectra

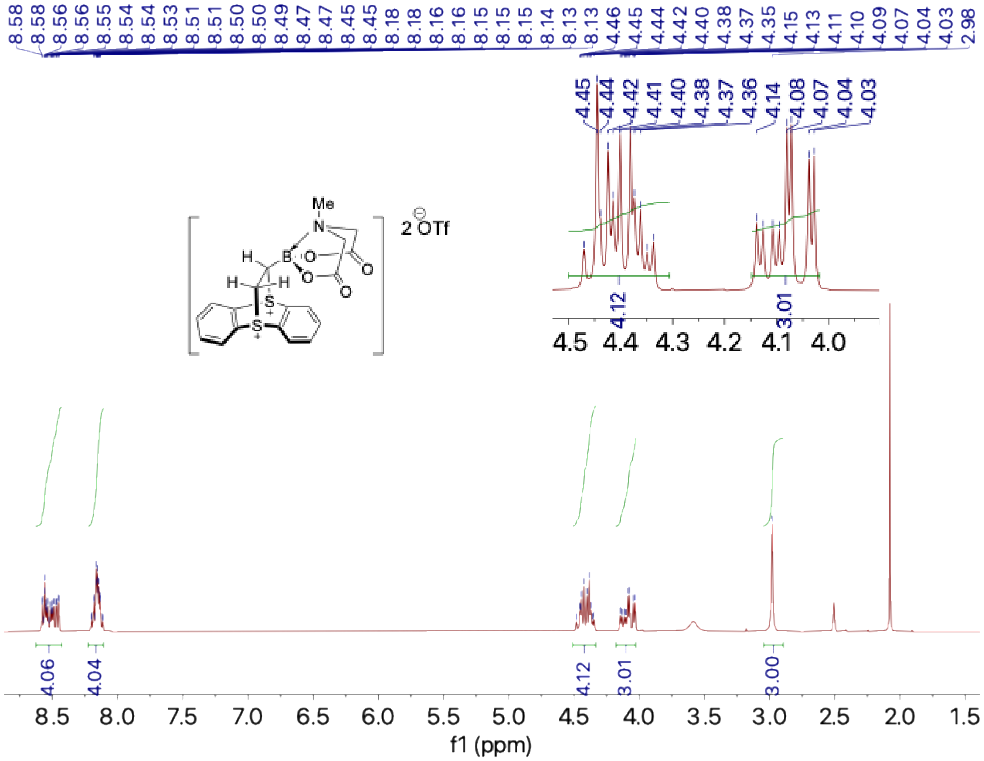

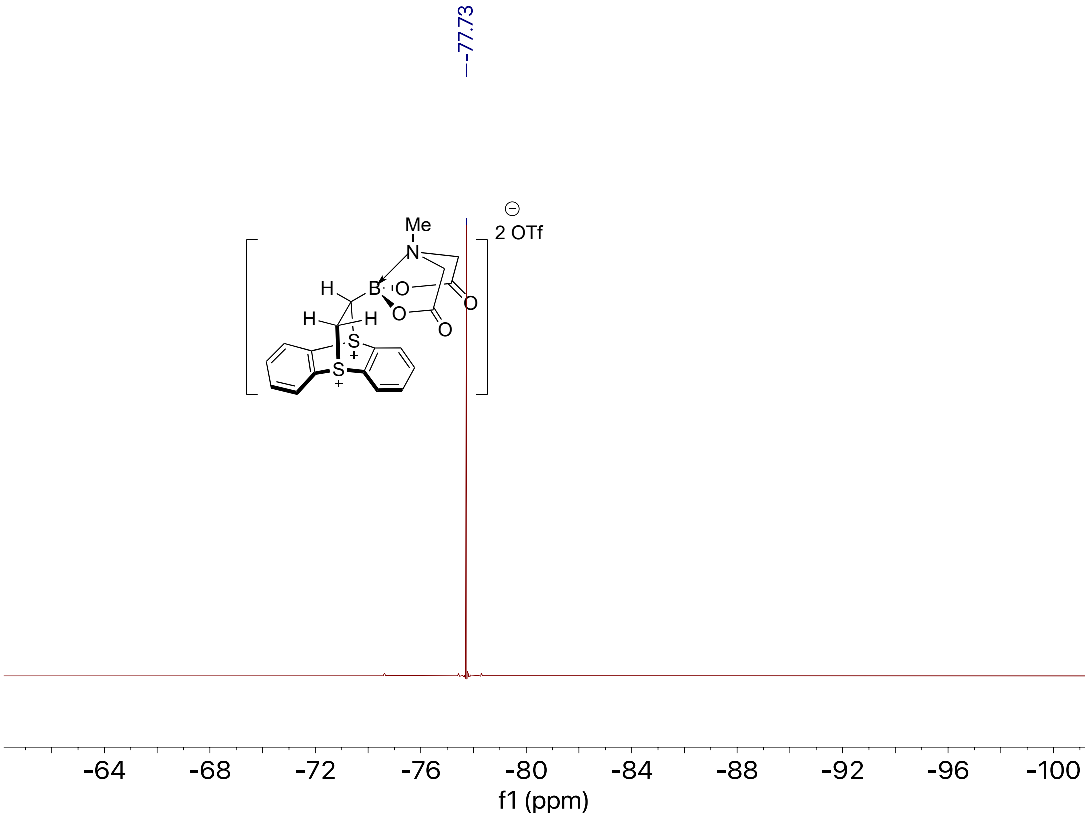

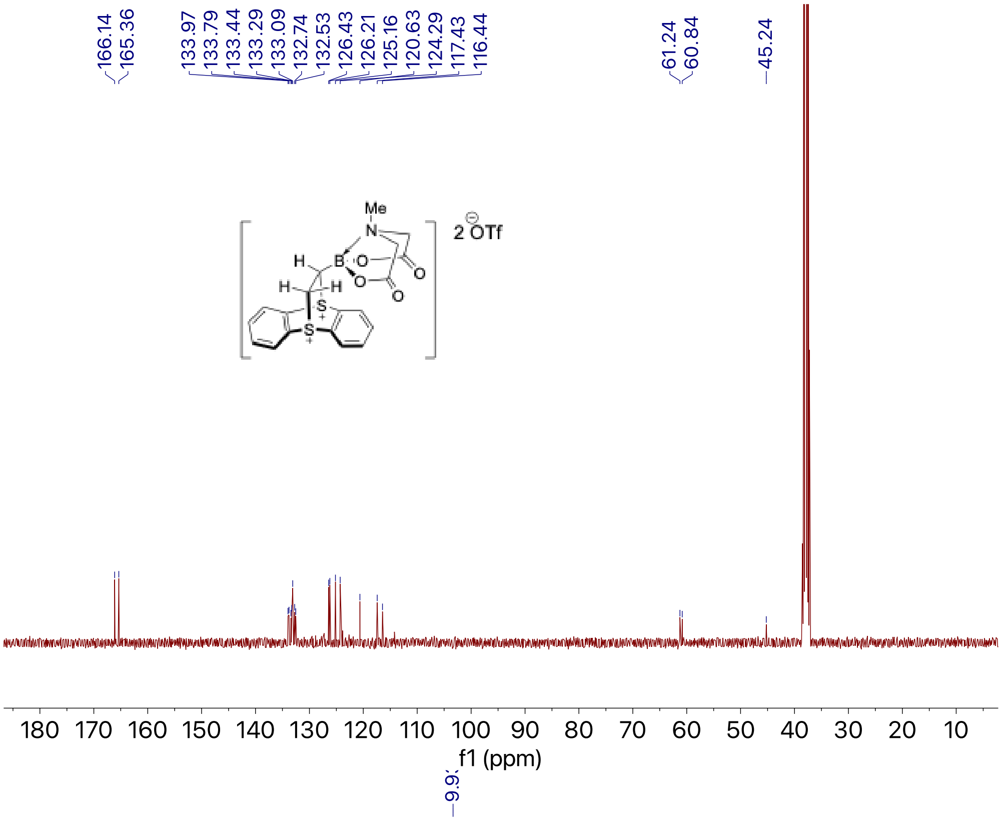
166.14
165.36
133.97
133.79
133.44
133.29
133.09
132.74
132.53
126.43
126.21
125.16
120.63
124.29
117.43
116.44
61.24
60.84
45.24
Me
N
H
B
O
O
O
O
H
H
S
S
2 OTf
180 170 160 150 140 130 120 110 100 90 80 70 60 50 40 30 20 10
f1 (ppm)

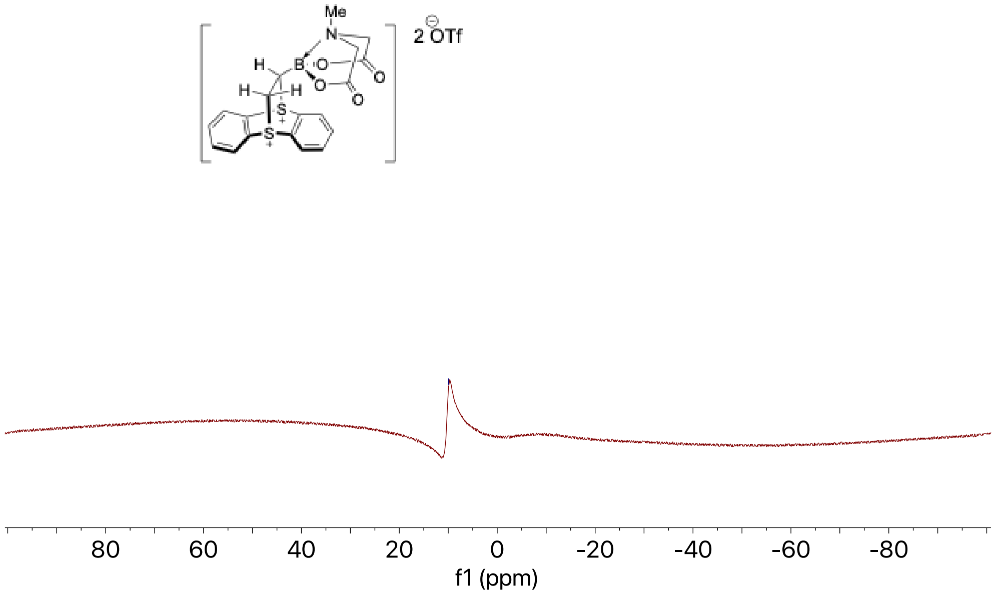
Me
N
H
B
O
O
O
O
H
H
S
S
2 OTf
80 60 40 20 0 -20 -40 -60 -80
f1 (ppm)

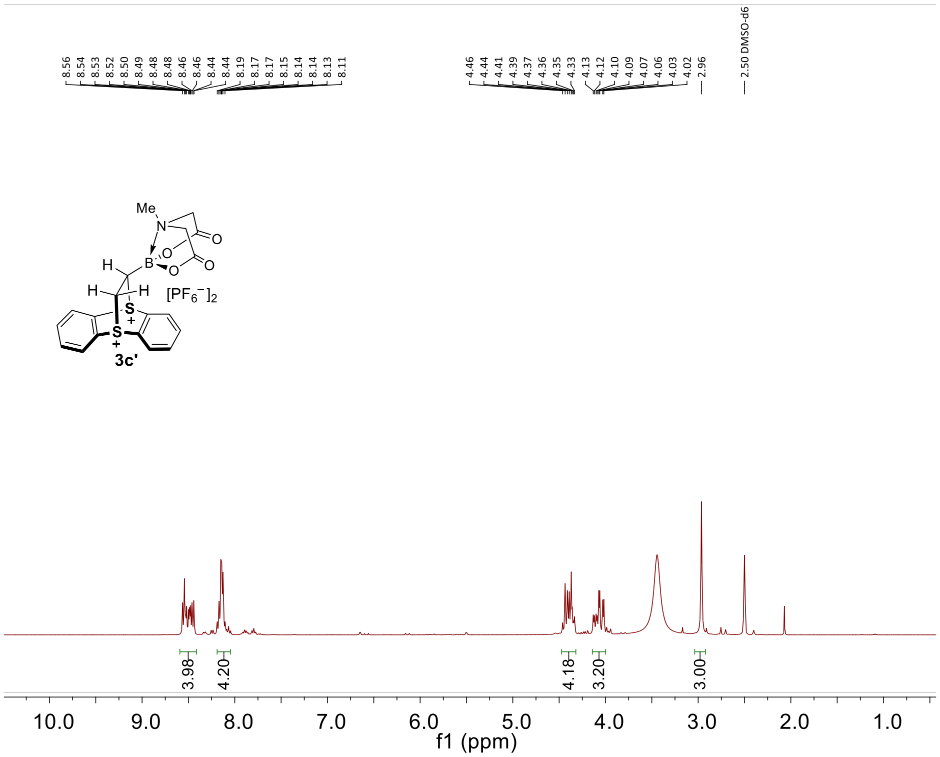
8.56
8.54
8.53
8.52
8.50
8.49
8.48
8.48
8.46
8.46
8.44
8.44
8.19
8.17
8.17
8.15
8.14
8.14
8.13
8.11
4.46
4.44
4.41
4.39
4.37
4.36
4.35
4.33
4.13
4.12
4.10
4.09
4.07
4.06
4.03
4.02
2.96
2.50 DMSO-d6
Me
N
O
B
O
O
O
H
H
H
[PF6−]2
S
+
S
+
3c'
3.98
4.20
4.18
3.20
3.00
10.0
9.0
8.0
7.0
6.0
5.0
4.0
3.0
2.0
1.0
f1 (ppm)

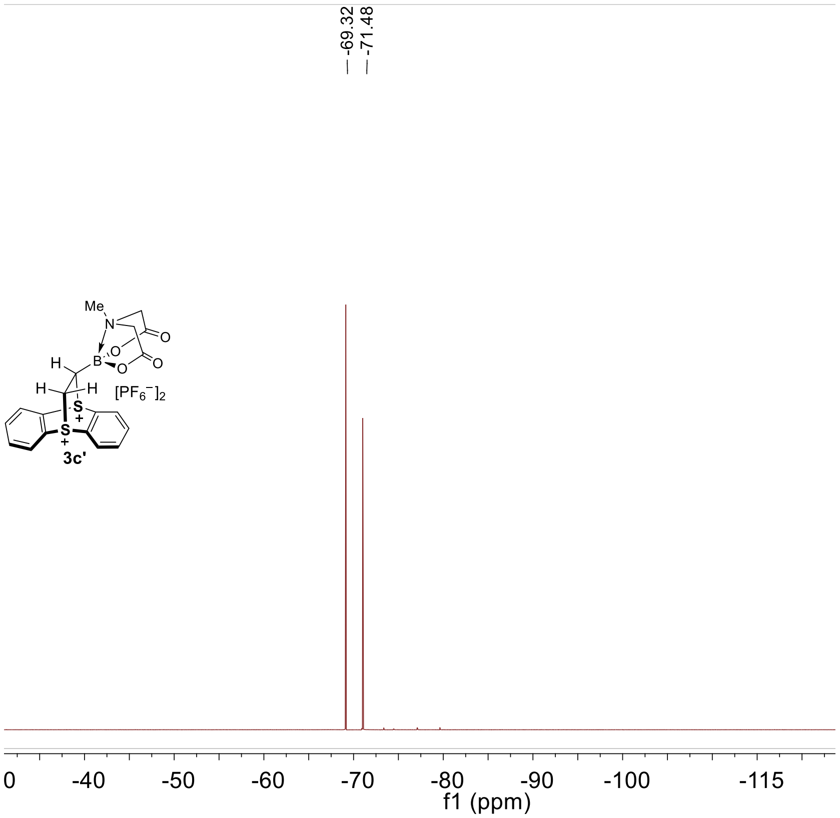
-69.32
-71.48
Me
N
O
B
O
O
O
H
H
H
[PF6−]2
S
+
S
+
3c'
0
-40
-50
-60
-70
-80
-90
-100
-115
f1 (ppm)

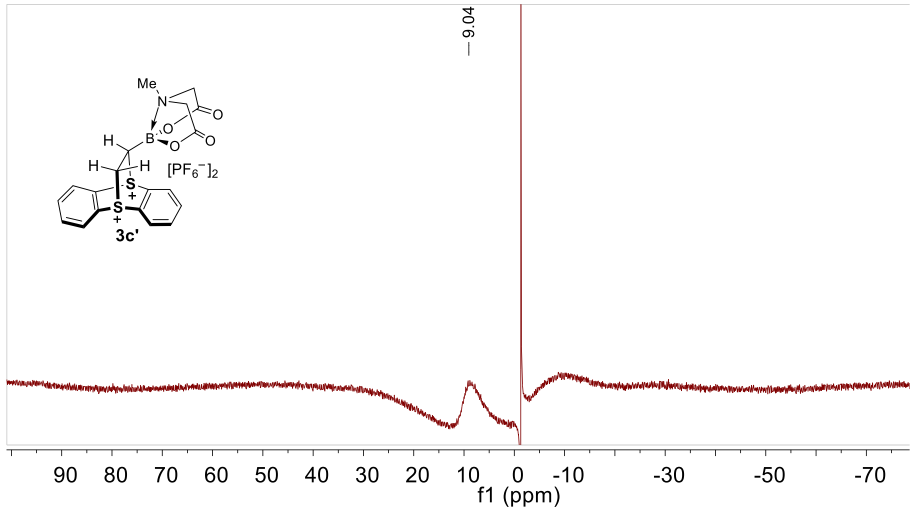

— 9.04
Me
N
O
O
H
B
O
O
H
H
[PF6⁻]2
S
+
S
+
3c'
90 80 70 60 50 40 30 20 10 0 -10 -30 -50 -70
f1 (ppm)

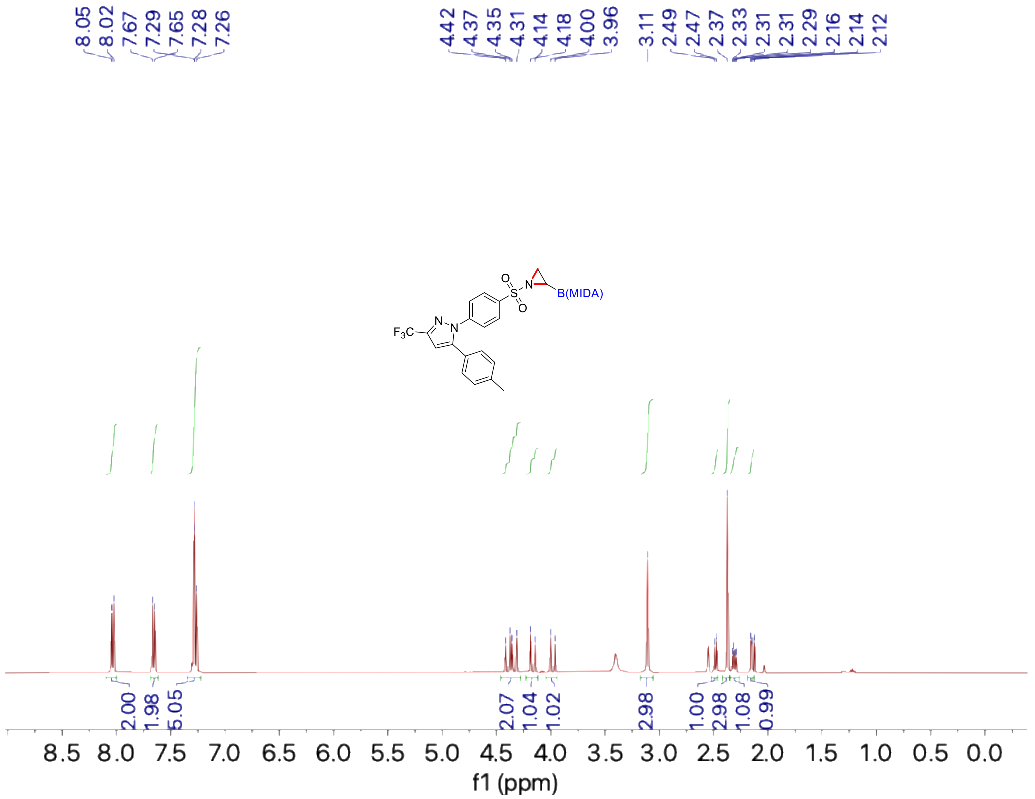

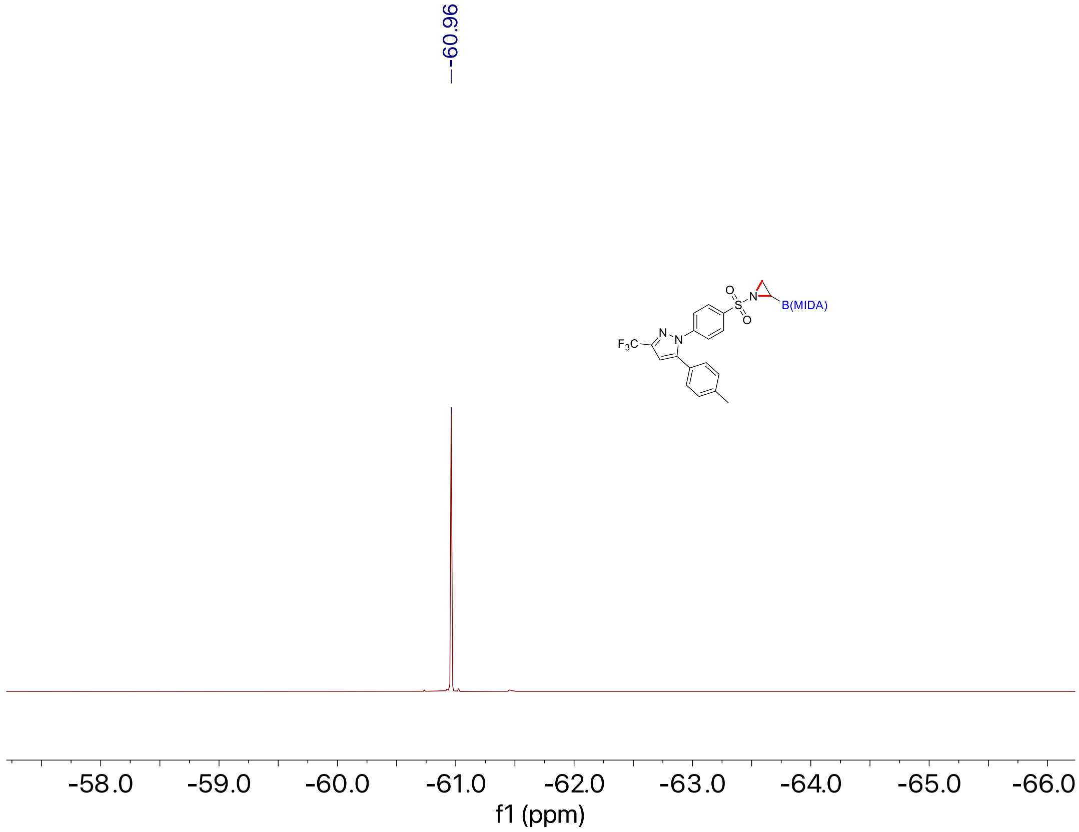

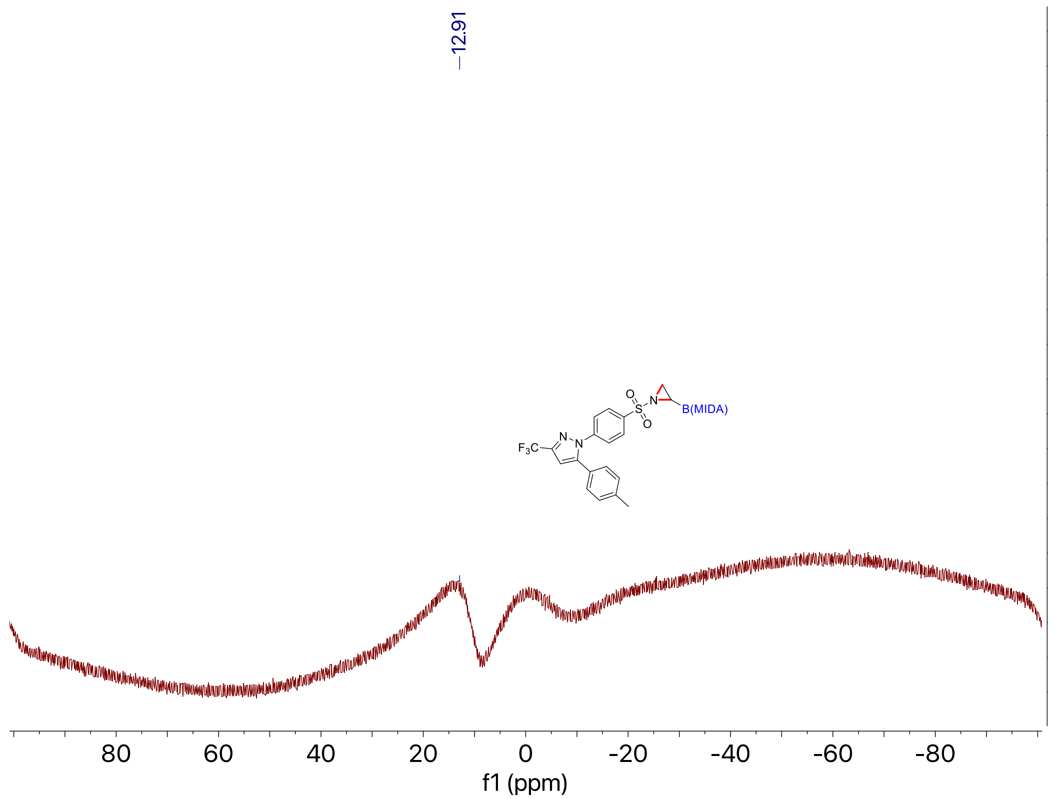

12.91
F3C
N
N
S
O
O
N
B(MIDA)
80 60 40 20 0 -20 -40 -60 -80
f1 (ppm)

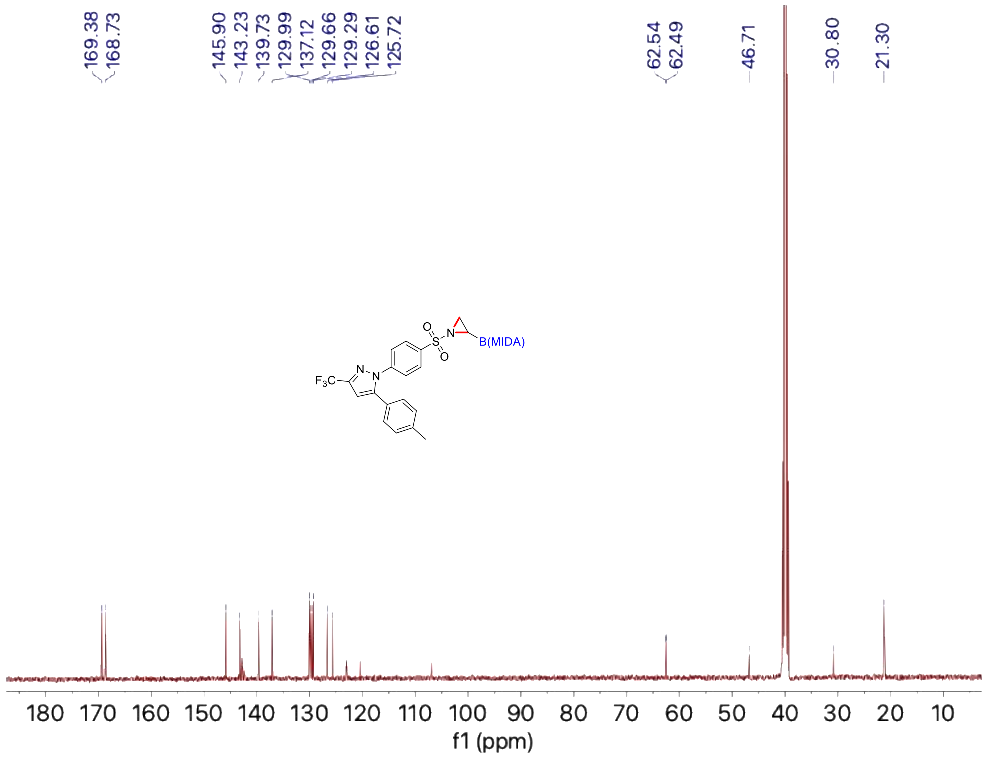

169.38
168.73
145.90
143.23
139.73
129.99
137.12
129.66
129.29
126.61
125.72
62.54
62.49
46.71
30.80
21.30
F3C
N
N
S
O
O
N
B(MIDA)
180 170 160 150 140 130 120 110 100 90 80 70 60 50 40 30 20 10
f1 (ppm)

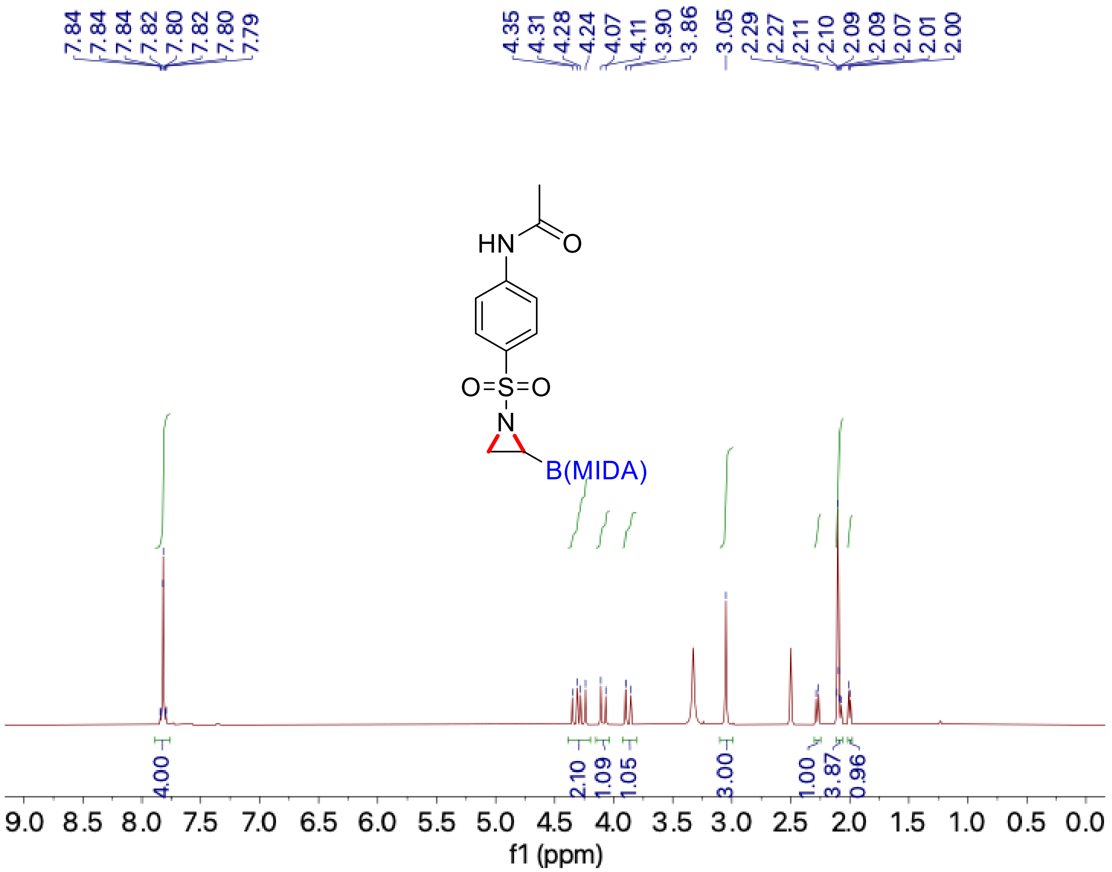
7.84
7.84
7.84
7.82
7.80
7.82
7.80
7.79
4.35
4.31
4.28
4.24
4.07
4.11
3.90
3.86
3.05
2.29
2.27
2.11
2.10
2.09
2.09
2.07
2.01
2.00
HN
O
O=S=O
N
B(MIDA)
4.00
2.10
1.09
1.05
3.00
1.00
3.87
0.96
9.0 8.5 8.0 7.5 7.0 6.5 6.0 5.5 5.0 4.5 4.0 3.5 3.0 2.5 2.0 1.5 1.0 0.5 0.0
f1 (ppm)

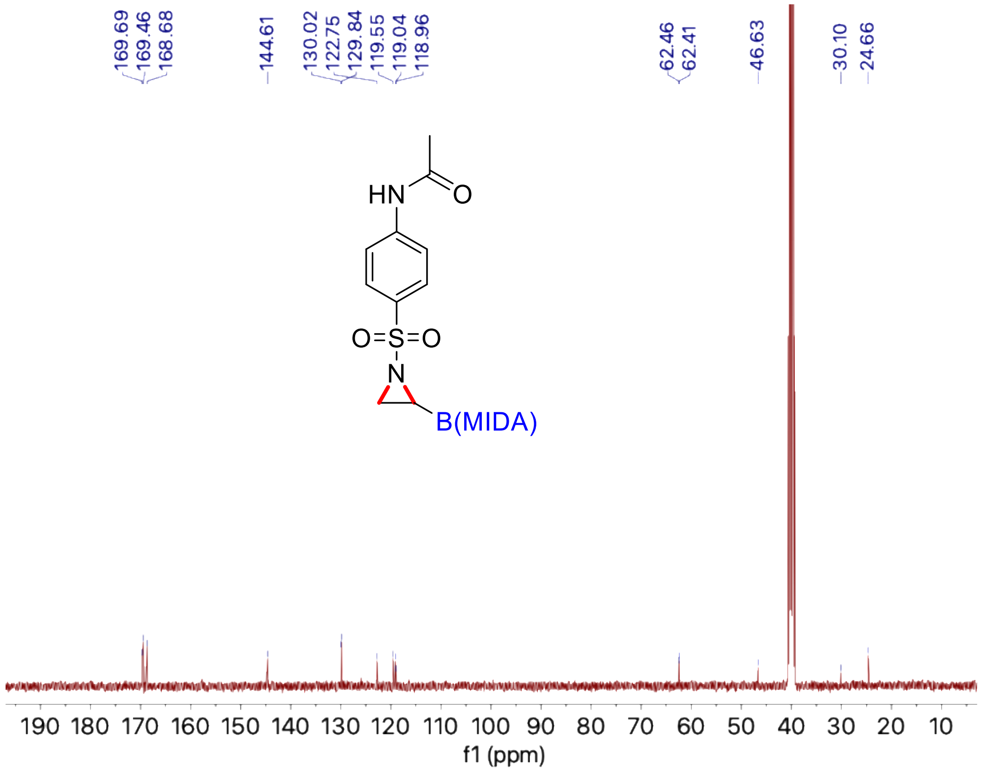
169.69
169.46
168.68
144.61
130.02
122.75
129.84
119.55
119.04
118.96
62.46
62.41
46.63
30.10
24.66
HN
O
O=S=O
N
B(MIDA)
190 180 170 160 150 140 130 120 110 100 90 80 70 60 50 40 30 20 10
f1 (ppm)

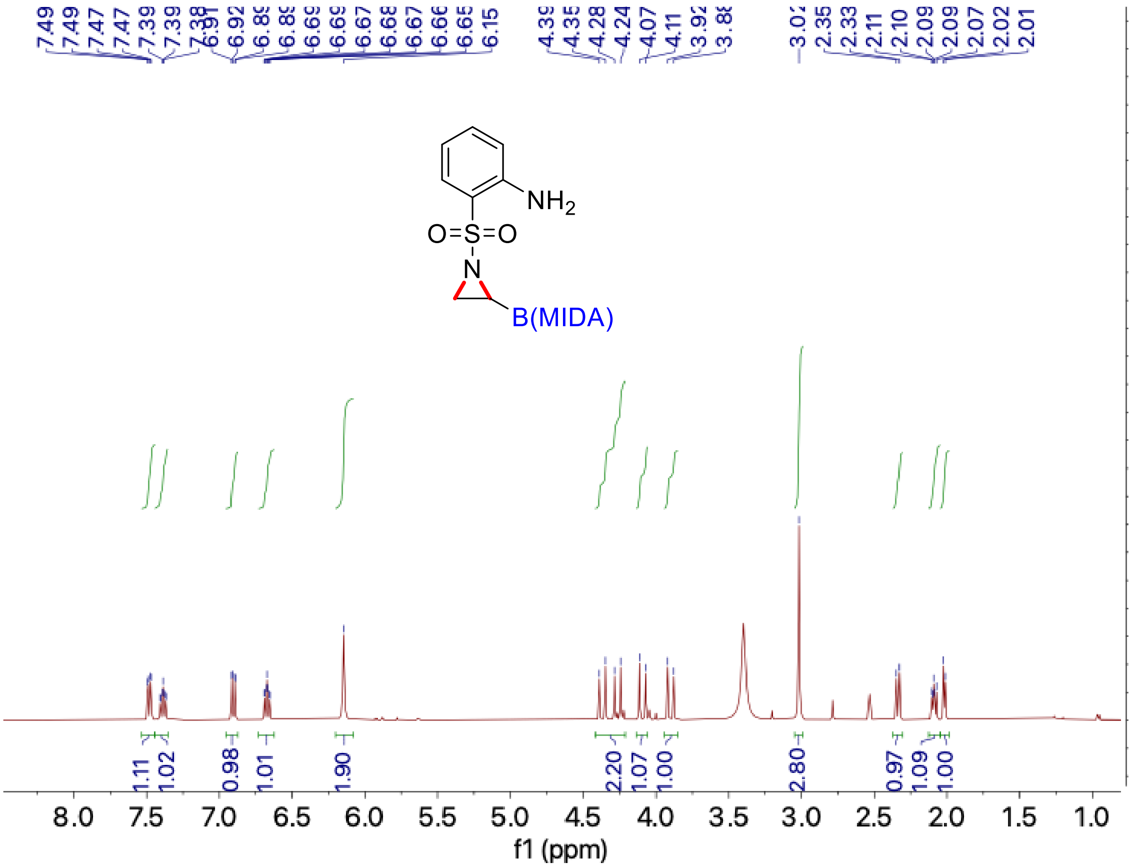
NH2
O=S=O
N
B(MIDA)
f1 (ppm)

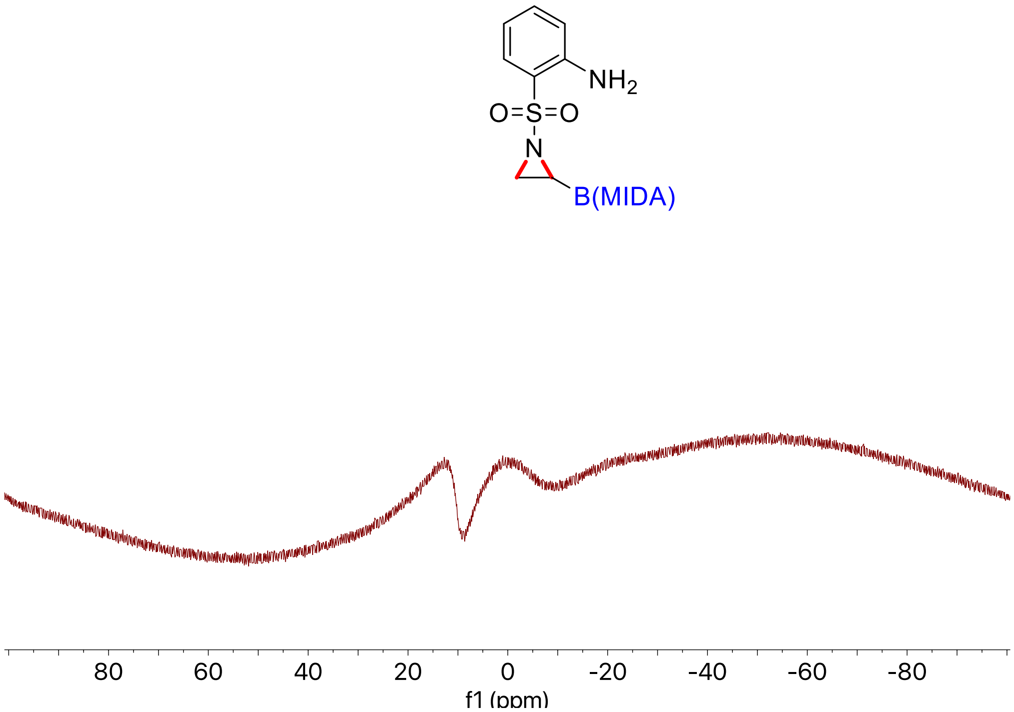
NH2
O=S=O
N
B(MIDA)
f1 (ppm)

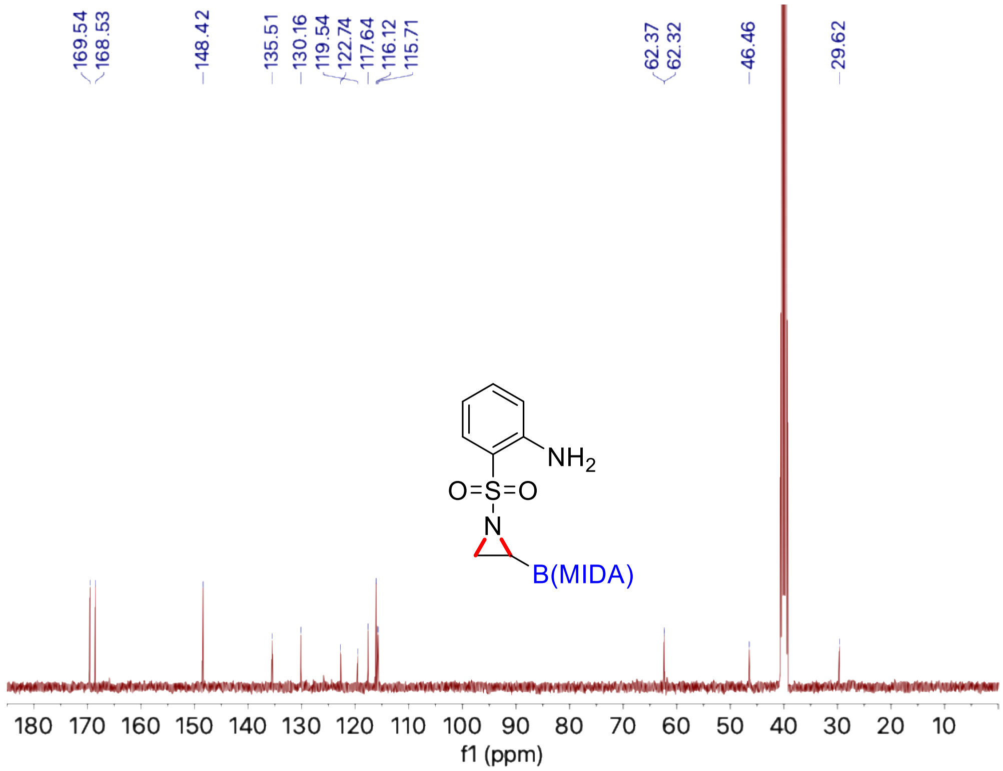

169.54
168.53
148.42
135.51
130.16
119.54
122.74
117.64
116.12
115.71
62.37
62.32
46.46
29.62
NH2
O=S=O
N
B(MIDA)
180 170 160 150 140 130 120 110 100 90 80 70 60 50 40 30 20 10
f1 (ppm)

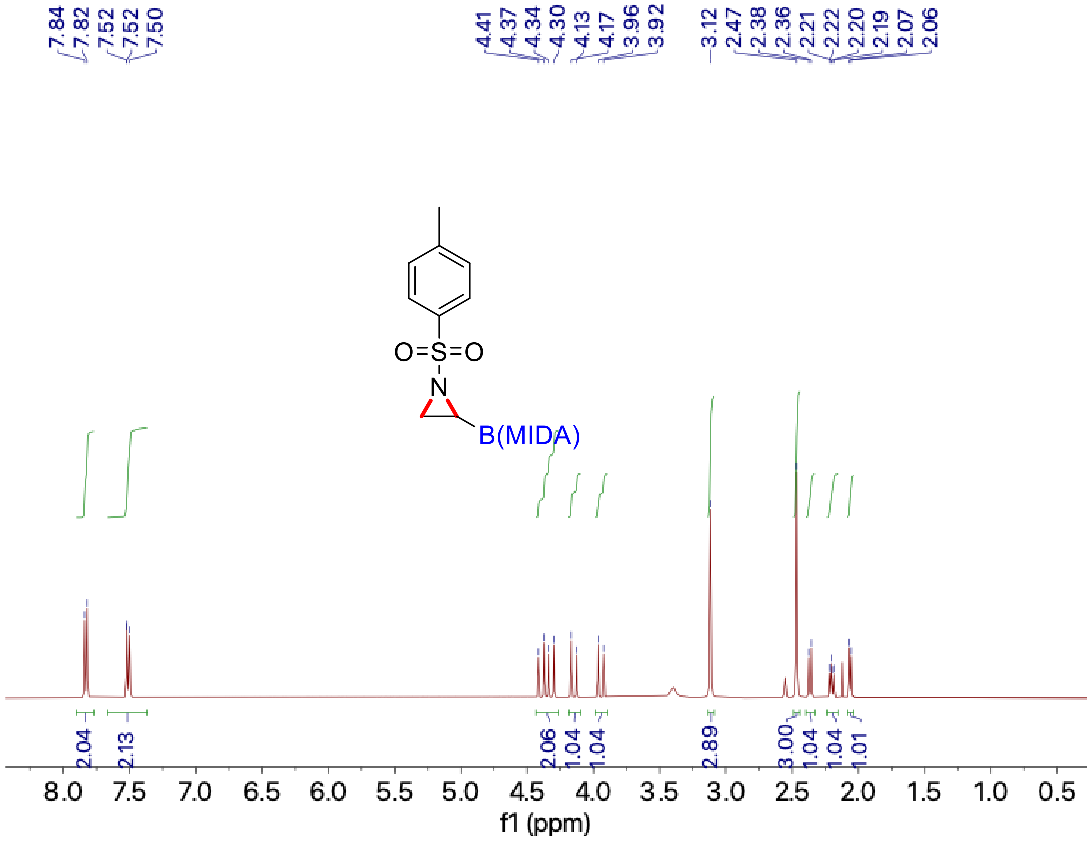
7.84
7.82
7.52
7.52
7.50
4.41
4.37
4.34
4.30
4.13
4.17
3.96
3.92
3.12
2.47
2.38
2.36
2.21
2.22
2.20
2.19
2.07
2.06
O=S=O
N
B(MIDA)
2.04
2.13
2.06
1.04
1.04
2.89
3.00
1.04
1.04
1.01
8.0 7.5 7.0 6.5 6.0 5.5 5.0 4.5 4.0 3.5 3.0 2.5 2.0 1.5 1.0 0.5
f1 (ppm)

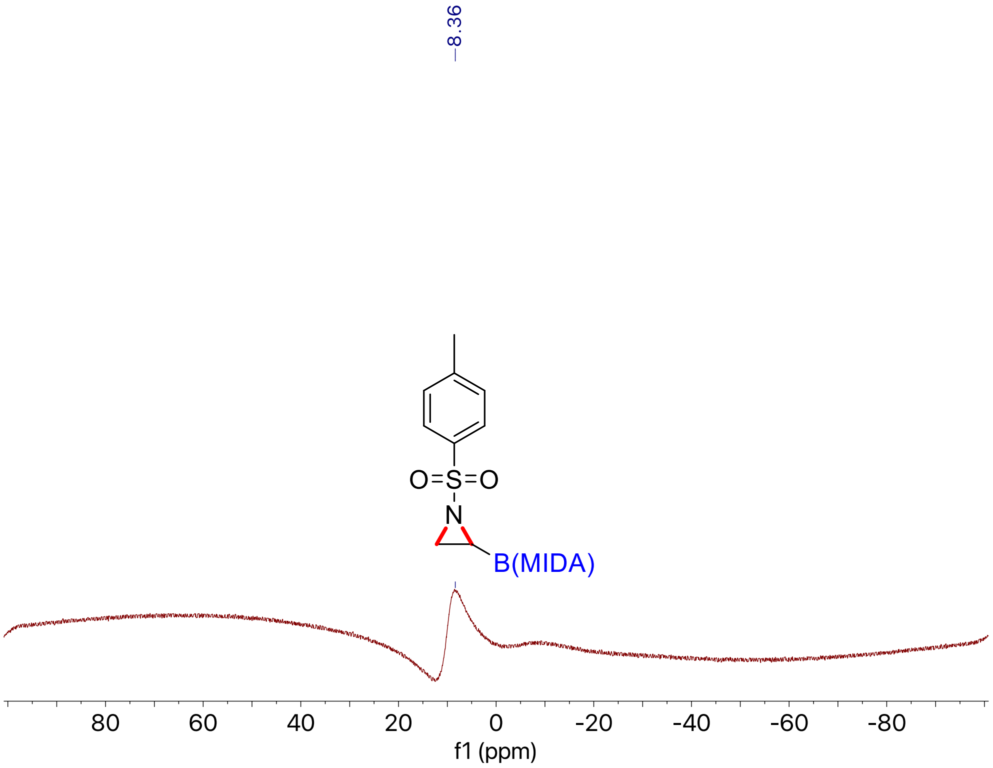
8.36
O=S=O
N
B(MIDA)
80 60 40 20 0 -20 -40 -60 -80
f1 (ppm)

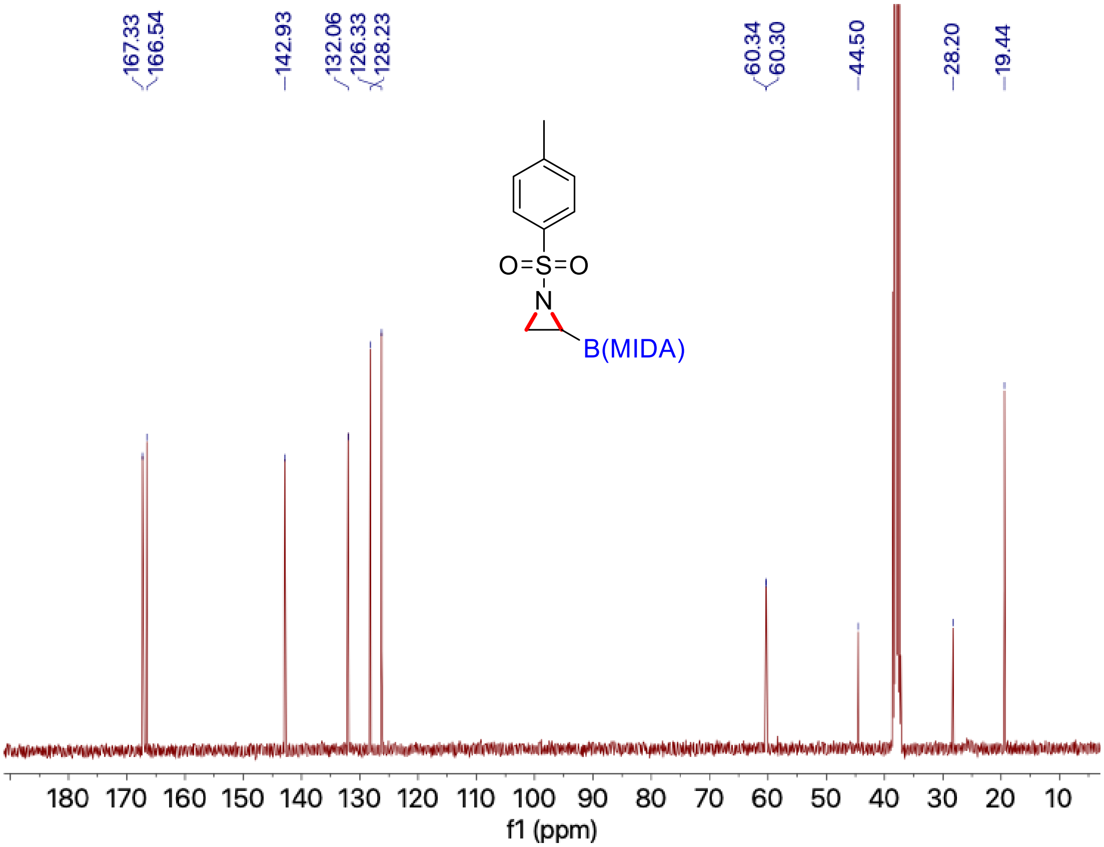

167.33
166.54
142.93
132.06
126.33
128.23
60.34
60.30
44.50
28.20
19.44
O=S=O
N
B(MIDA)
180 170 160 150 140 130 120 110 100 90 80 70 60 50 40 30 20 10
f1 (ppm)

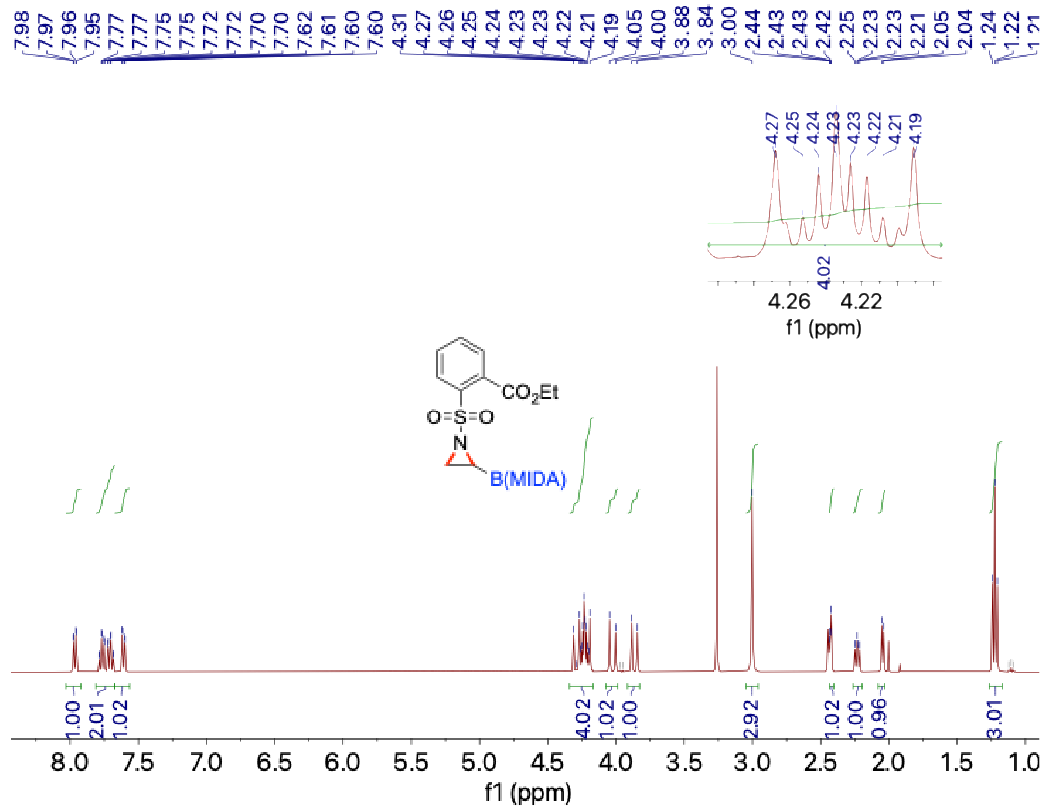
CO2Et
O=S=O
N
B(MIDA)
4.26
4.22
f1 (ppm)
8.0
7.5
7.0
6.5
6.0
5.5
5.0
4.5
4.0
3.5
3.0
2.5
2.0
1.5
1.0
f1 (ppm)

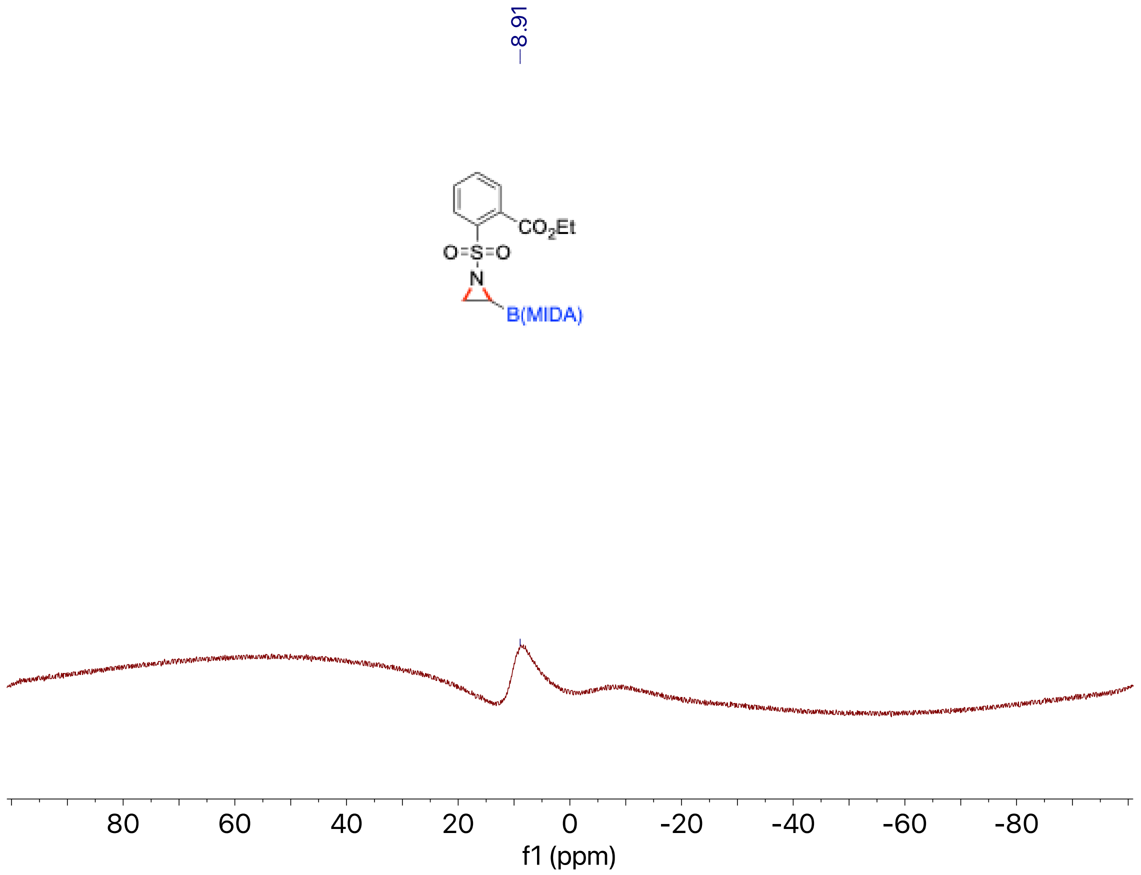
—8.91
CO2Et
O=S=O
N
B(MIDA)
80
60
40
20
0
-20
-40
-60
-80
f1 (ppm)

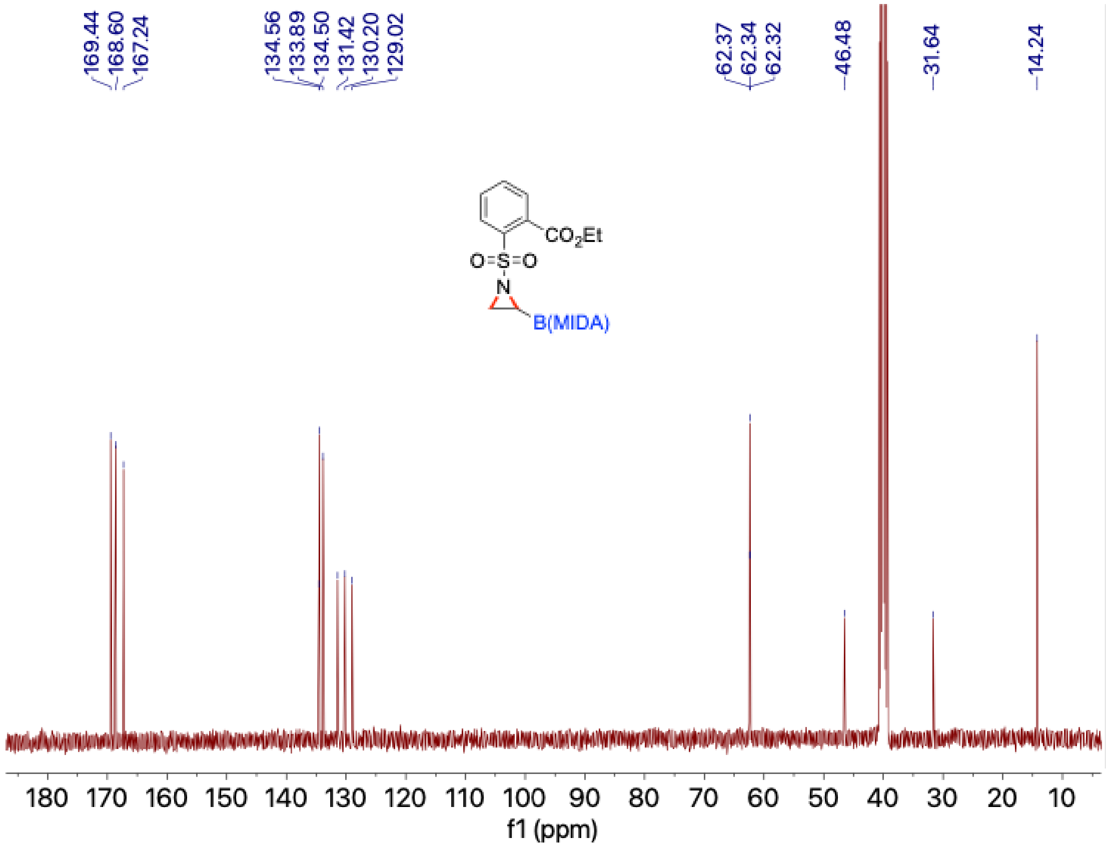
169.44
168.60
167.24
134.56
133.89
134.50
131.42
130.20
129.02
62.37
62.34
62.32
46.48
31.64
14.24
CO2Et
O=S=O
N
B(MIDA)
180 170 160 150 140 130 120 110 100 90 80 70 60 50 40 30 20 10
f1 (ppm)

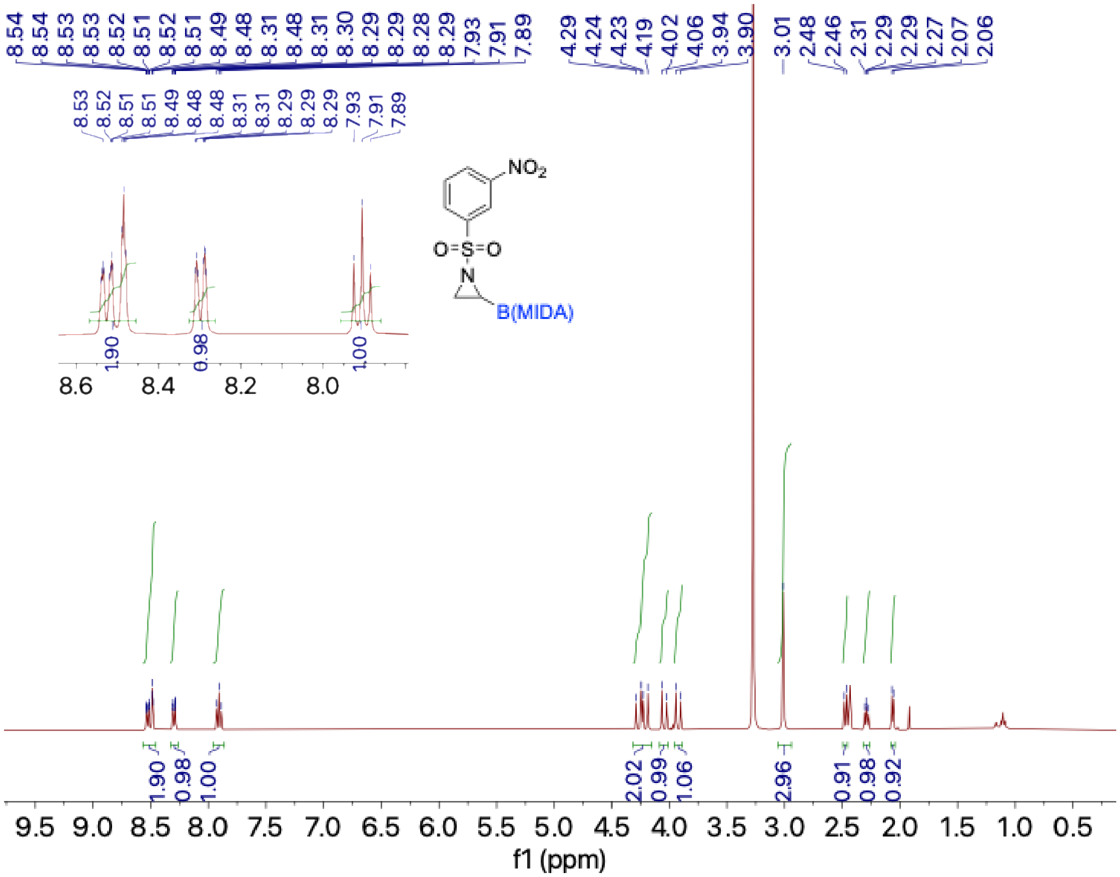

NO2
O=S=O
N
B(MIDA)
8.6
8.4
8.2
8.0
1.90
0.98
1.00
2.02
0.99
1.06
2.96
0.91
0.98
0.92
9.5 9.0 8.5 8.0 7.5 7.0 6.5 6.0 5.5 5.0 4.5 4.0 3.5 3.0 2.5 2.0 1.5 1.0 0.5
f1 (ppm)

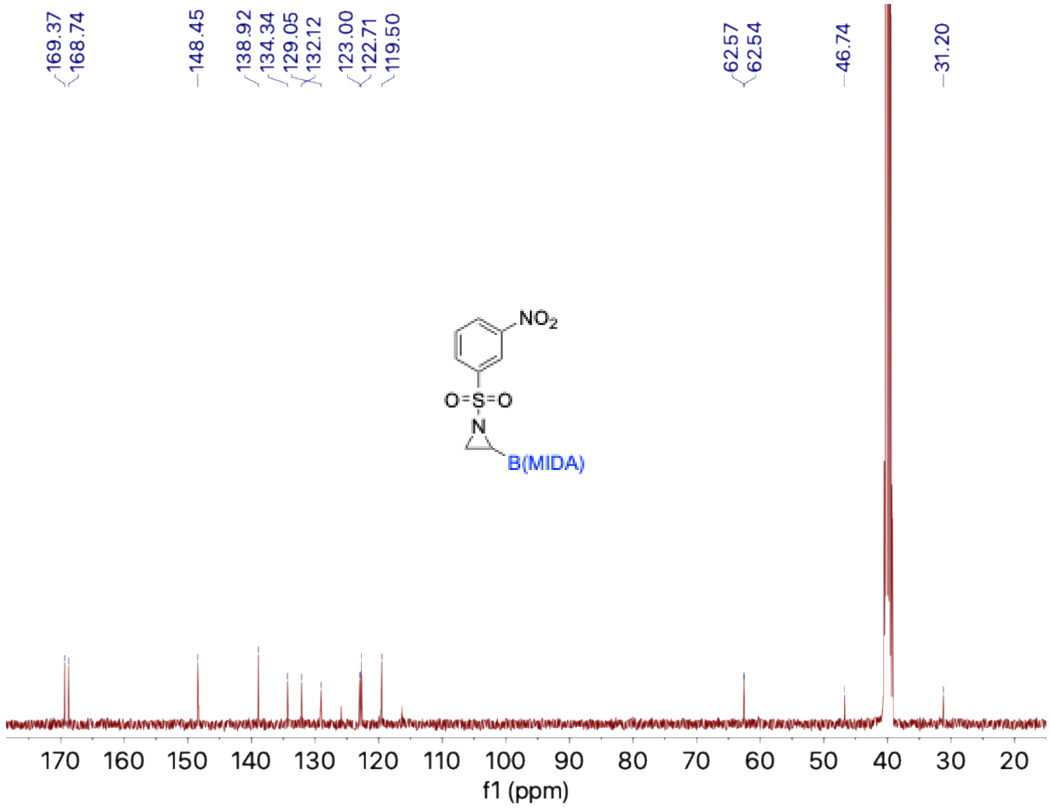
169.37
168.74
148.45
138.92
134.34
129.05
132.12
123.00
122.71
119.50
62.57
62.54
46.74
31.20
NO2
O=S=O
N
B(MIDA)
170 160 150 140 130 120 110 100 90 80 70 60 50 40 30 20
f1 (ppm)

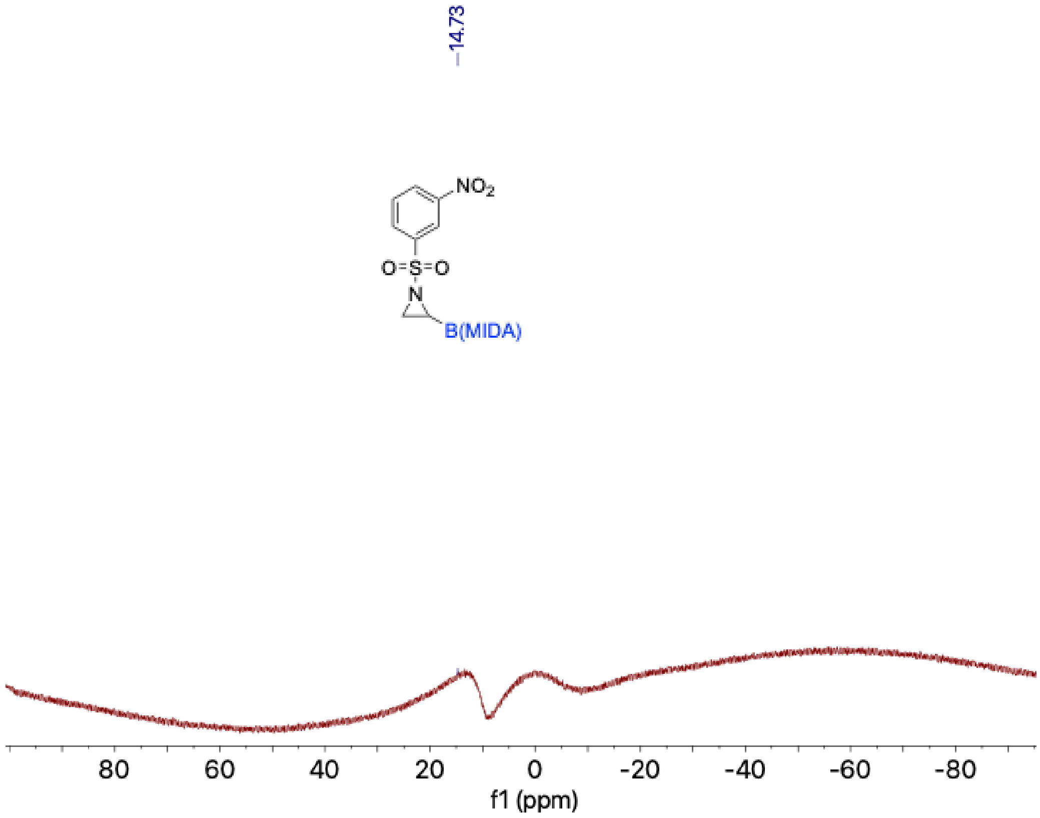
14.73
NO2
O=S=O
N
B(MIDA)
80 60 40 20 0 -20 -40 -60 -80
f1 (ppm)

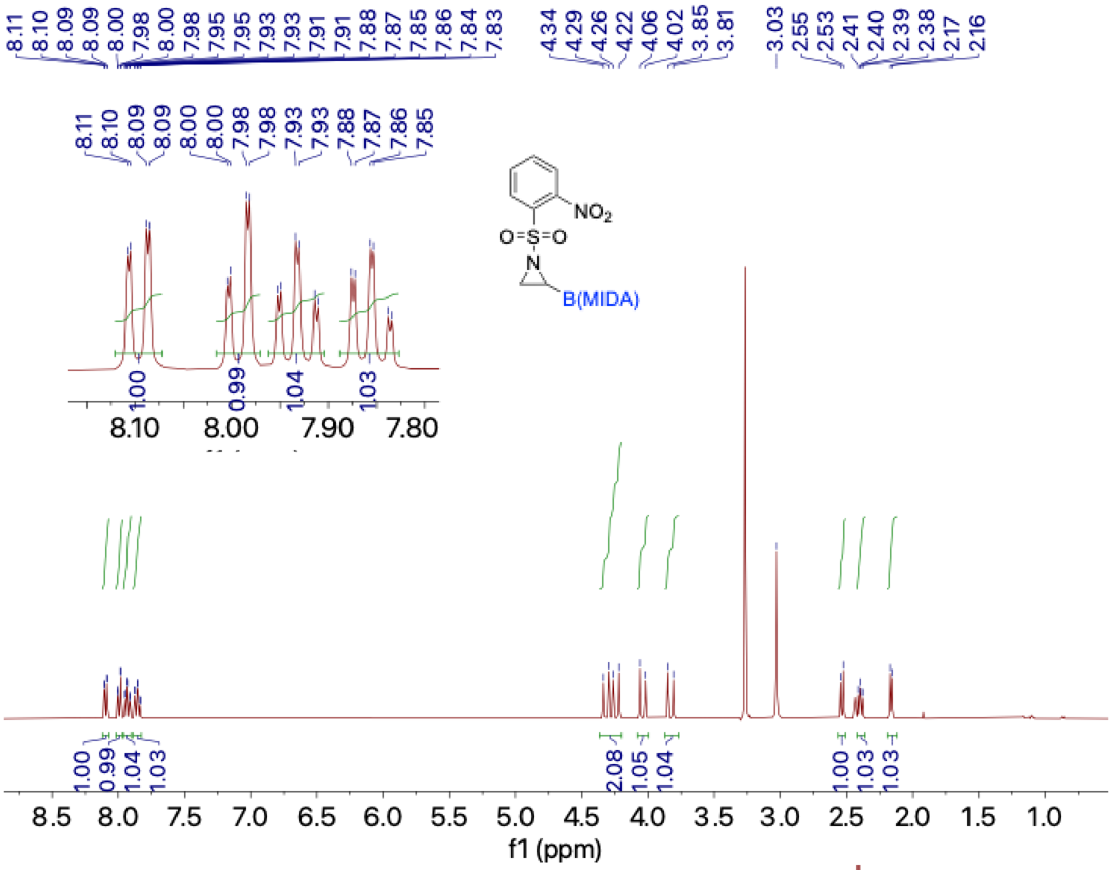
8.11
8.10
8.09
8.09
8.00
7.98
8.00
7.98
7.95
7.95
7.93
7.93
7.91
7.91
7.88
7.87
7.85
7.86
7.84
7.83
4.34
4.29
4.26
4.22
4.06
4.02
3.85
3.81
3.03
2.55
2.53
2.41
2.40
2.39
2.38
2.17
2.16
NO2
O=S=O
N
B(MIDA)
1.00
0.99
1.04
1.03
8.10
8.00
7.90
7.80
2.08
1.05
1.04
1.00
1.03
1.03
8.5
8.0
7.5
7.0
6.5
6.0
5.5
5.0
4.5
4.0
3.5
3.0
2.5
2.0
1.5
1.0
f1 (ppm)

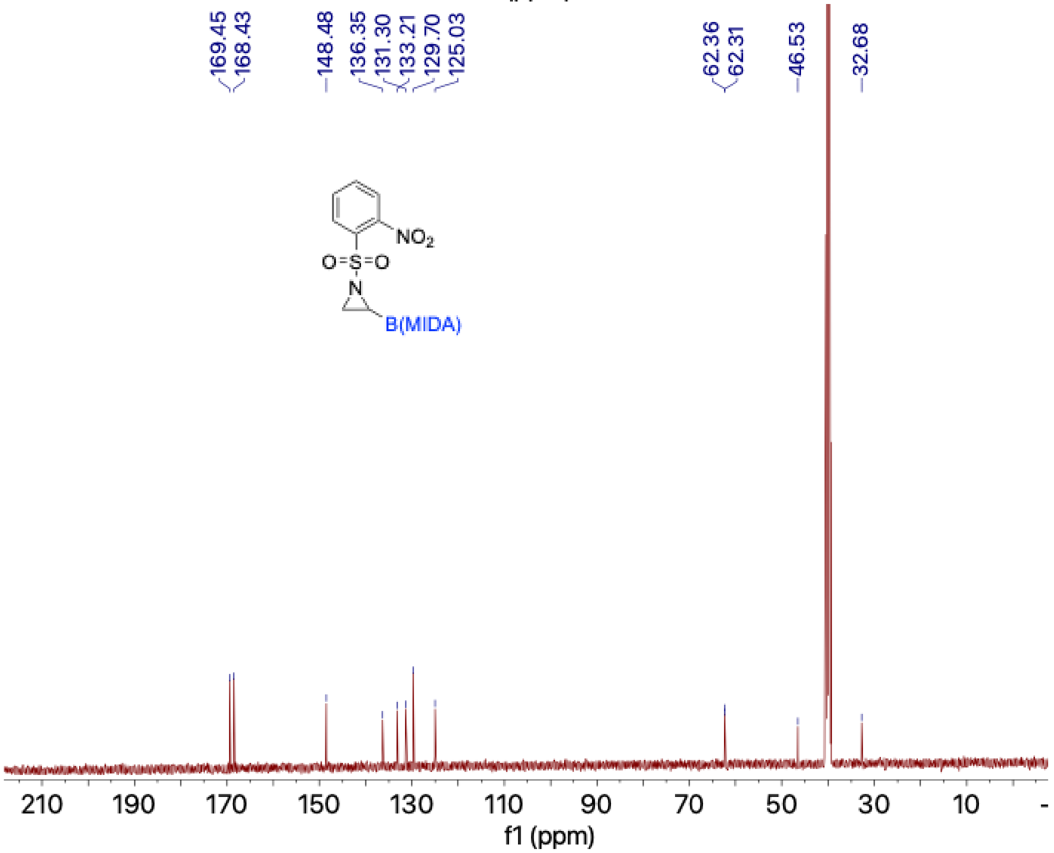
169.45
168.43
148.48
136.35
131.30
133.21
129.70
125.03
62.36
62.31
46.53
32.68
NO2
O=S=O
N
B(MIDA)
210
190
170
150
130
110
90
70
50
30
10
f1 (ppm)

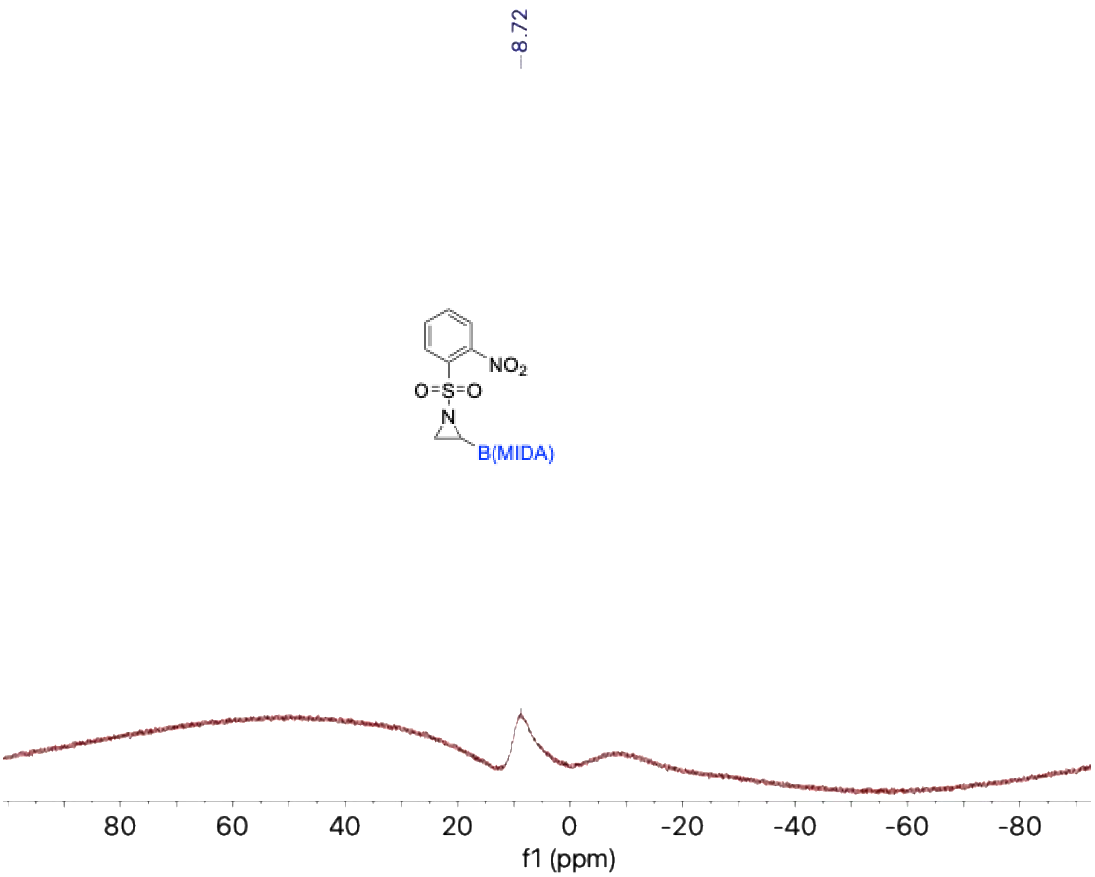

8.72
NO2
O=S=O
N
B(MIDA)
80
60
40
20
0
-20
-40
-60
-80
f1 (ppm)

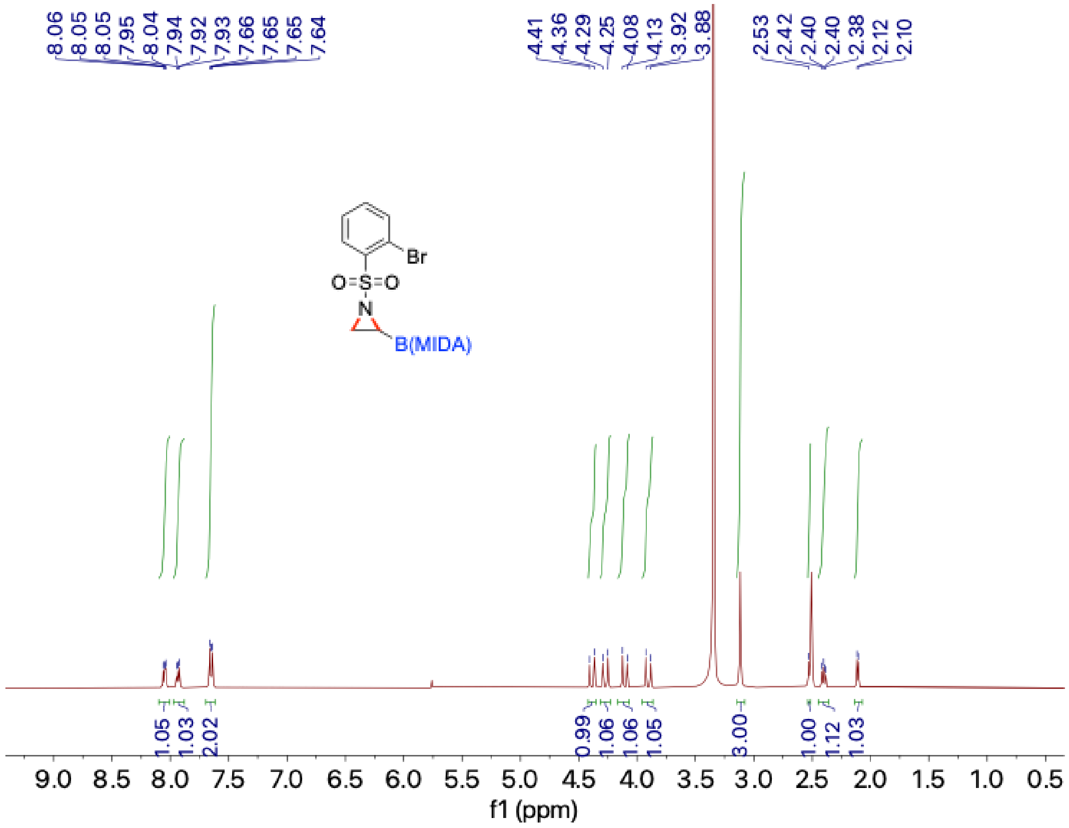
8.06
8.05
8.05
7.95
8.04
7.94
7.92
7.93
7.66
7.65
7.65
7.64
4.41
4.36
4.29
4.25
4.08
4.13
3.92
3.88
2.53
2.42
2.40
2.40
2.38
2.12
2.10
Br
O=S=O
N
B(MIDA)
1.05
1.03
2.02
0.99
1.06
1.06
1.05
3.00
1.00
1.12
1.03
9.0 8.5 8.0 7.5 7.0 6.5 6.0 5.5 5.0 4.5 4.0 3.5 3.0 2.5 2.0 1.5 1.0 0.5
f1 (ppm)

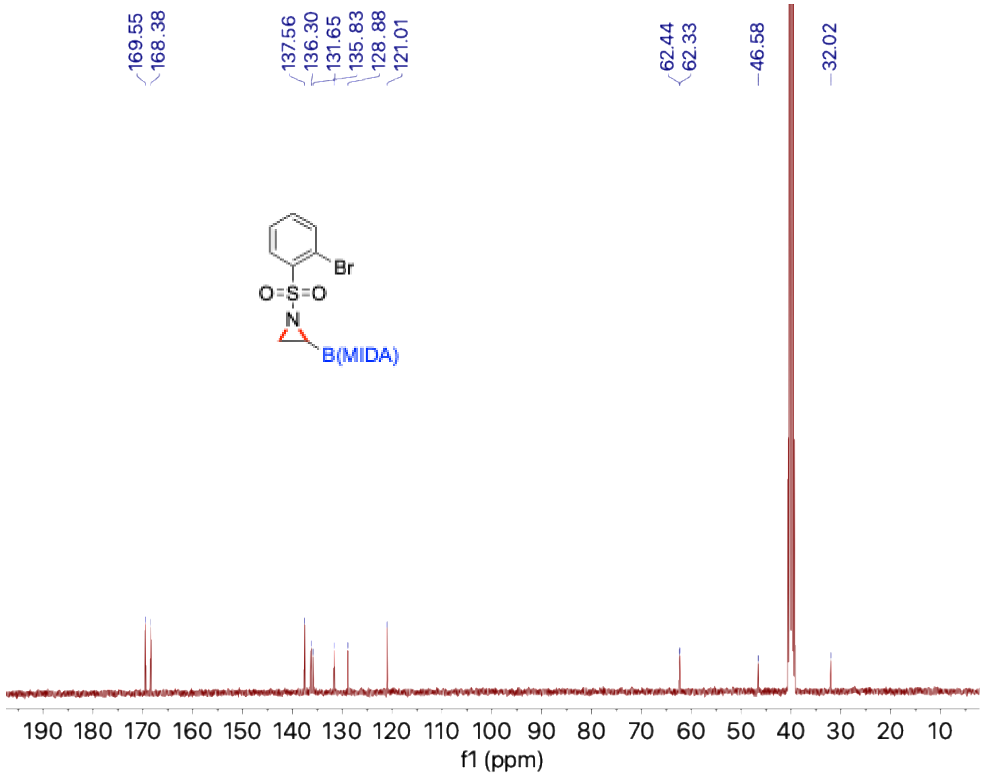
169.55
168.38
137.56
136.30
131.65
135.83
128.88
121.01
62.44
62.33
46.58
32.02
Br
O=S=O
N
B(MIDA)
190 180 170 160 150 140 130 120 110 100 90 80 70 60 50 40 30 20 10
f1 (ppm)

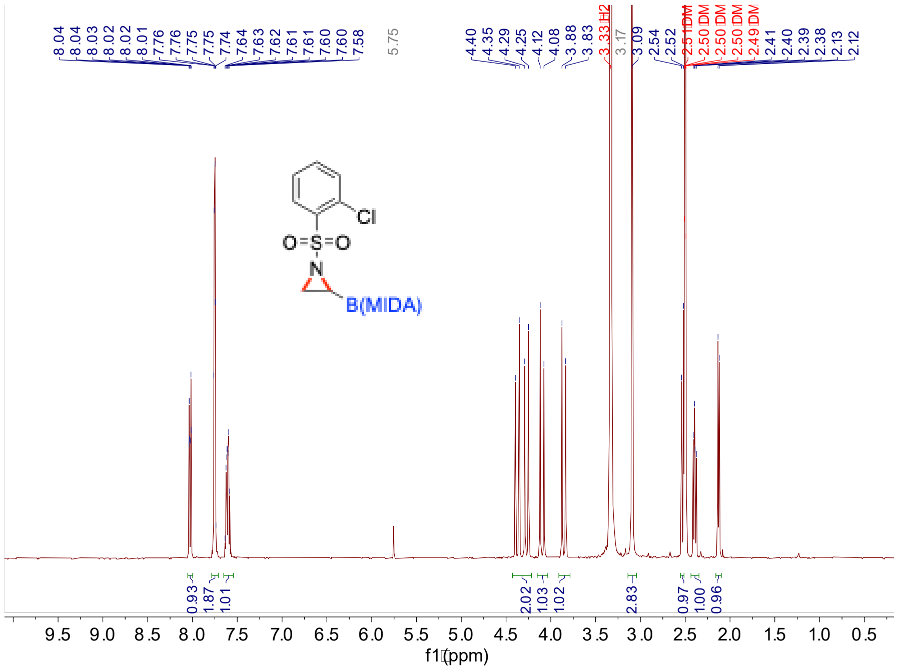
8.04
8.04
8.03
8.02
8.02
8.01
7.76
7.76
7.75
7.75
7.74
7.64
7.63
7.62
7.62
7.61
7.61
7.60
7.60
7.58
5.75
4.40
4.35
4.29
4.25
4.12
4.08
3.88
3.83
3.33 H2
3.17
3.09
2.54
2.52
2.51 DM
2.50 DM
2.50 DM
2.50 DM
2.49 DM
2.41
2.40
2.39
2.38
2.13
2.12
Cl
O=S=O
N
B(MIDA)
0.93
1.87
1.01
2.02
1.03
1.02
2.83
0.97
1.00
0.96
9.5 9.0 8.5 8.0 7.5 7.0 6.5 6.0 5.5 5.0 4.5 4.0 3.5 3.0 2.5 2.0 1.5 1.0 0.5
f1 (ppm)

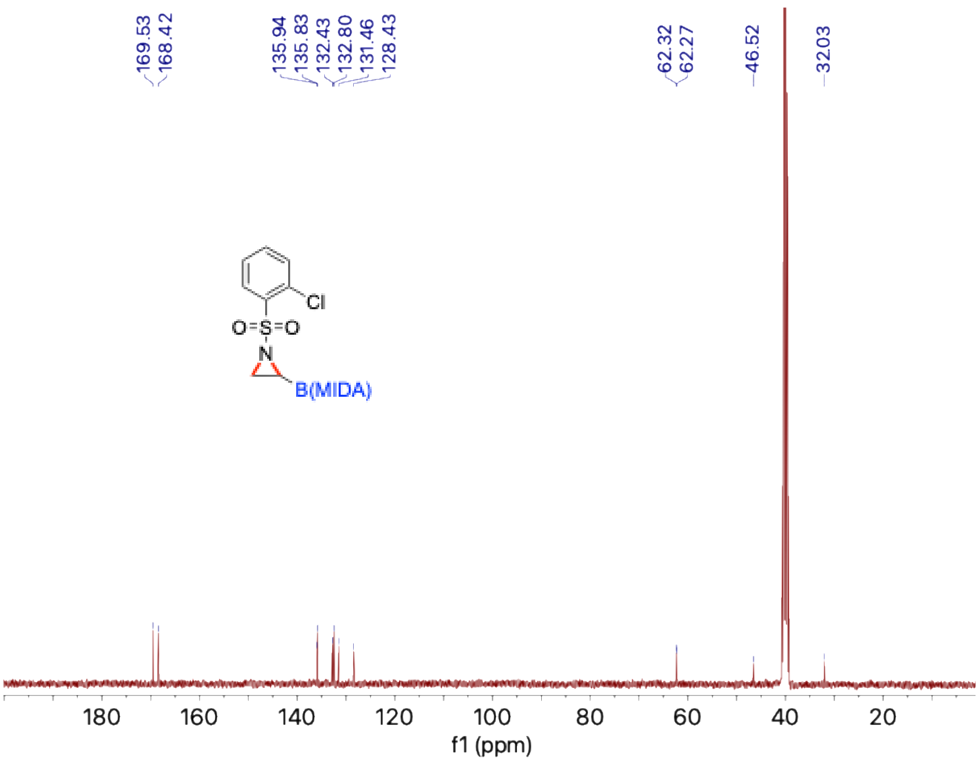
169.53
168.42
135.94
135.83
132.43
132.80
131.46
128.43
62.32
62.27
46.52
32.03
Cl
O=S=O
N
B(MIDA)
180 160 140 120 100 80 60 40 20
f1 (ppm)

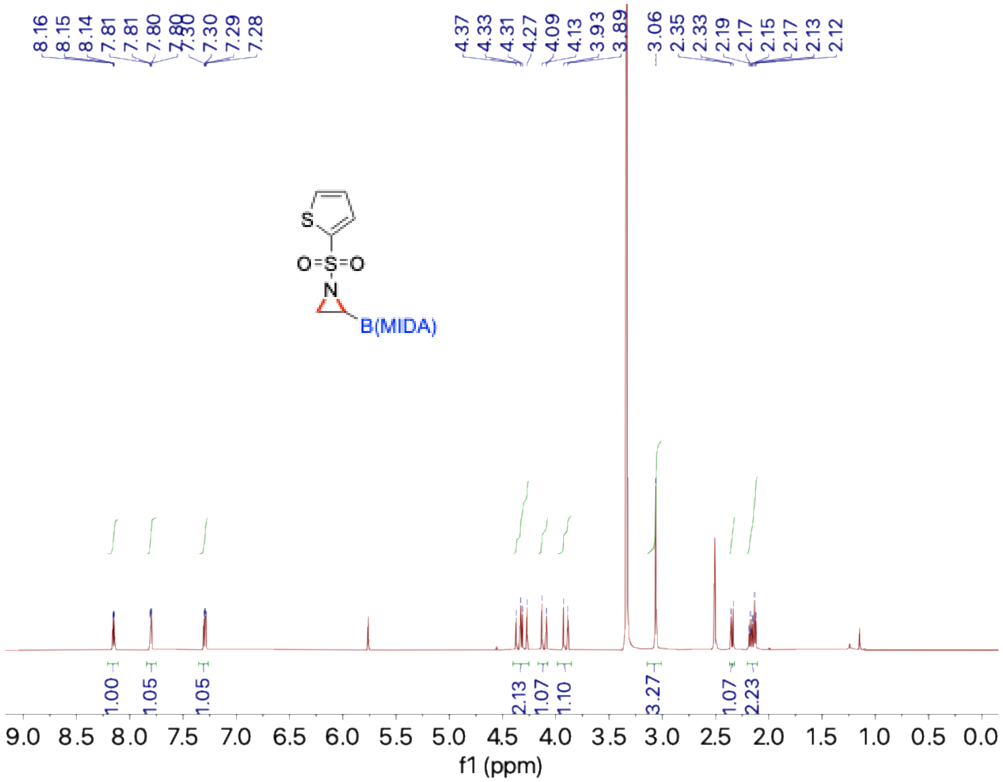
8.16
8.15
8.14
7.81
7.81
7.80
7.80
7.30
7.30
7.29
7.28
4.37
4.33
4.31
4.27
4.09
4.13
3.93
3.89
3.06
2.35
2.33
2.19
2.17
2.15
2.17
2.13
2.12
S
O=S=O
N
B(MIDA)
1.00
1.05
1.05
2.13
1.07
1.10
3.27
1.07
2.23
9.0 8.5 8.0 7.5 7.0 6.5 6.0 5.5 5.0 4.5 4.0 3.5 3.0 2.5 2.0 1.5 1.0 0.5 0.0
f1 (ppm)

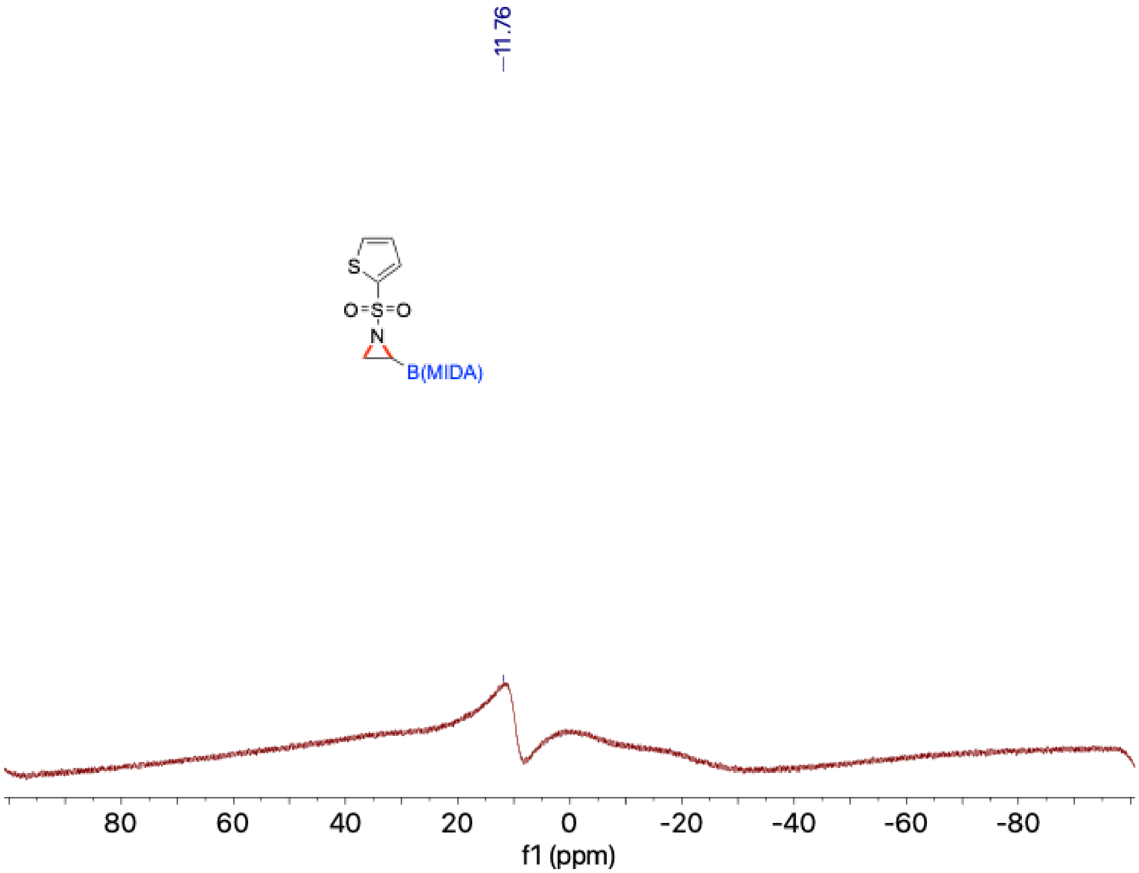
11.76
S
O=S=O
N
B(MIDA)
80 60 40 20 0 -20 -40 -60 -80
f1 (ppm)

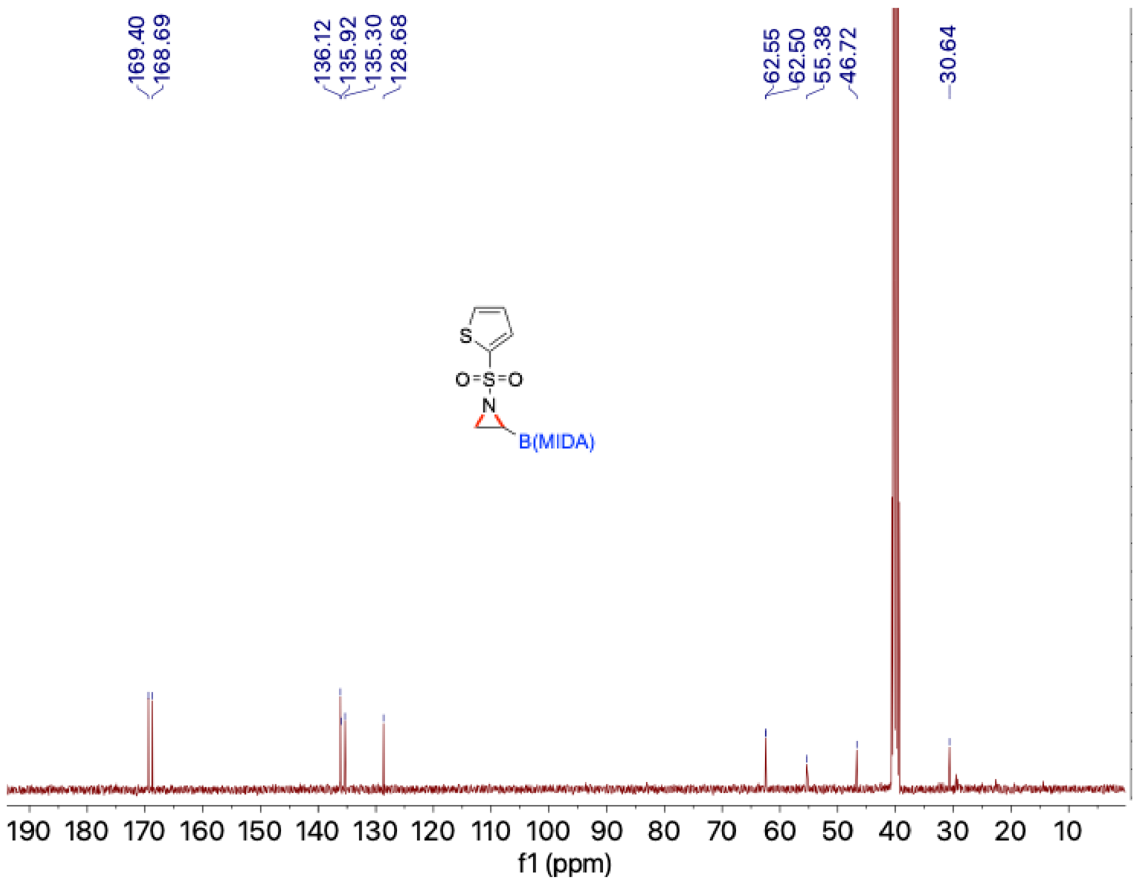
169.40
168.69
136.12
135.92
135.30
128.68
62.55
62.50
55.38
46.72
30.64
S
O=S=O
N
B(MIDA)
190 180 170 160 150 140 130 120 110 100 90 80 70 60 50 40 30 20 10
f1 (ppm)

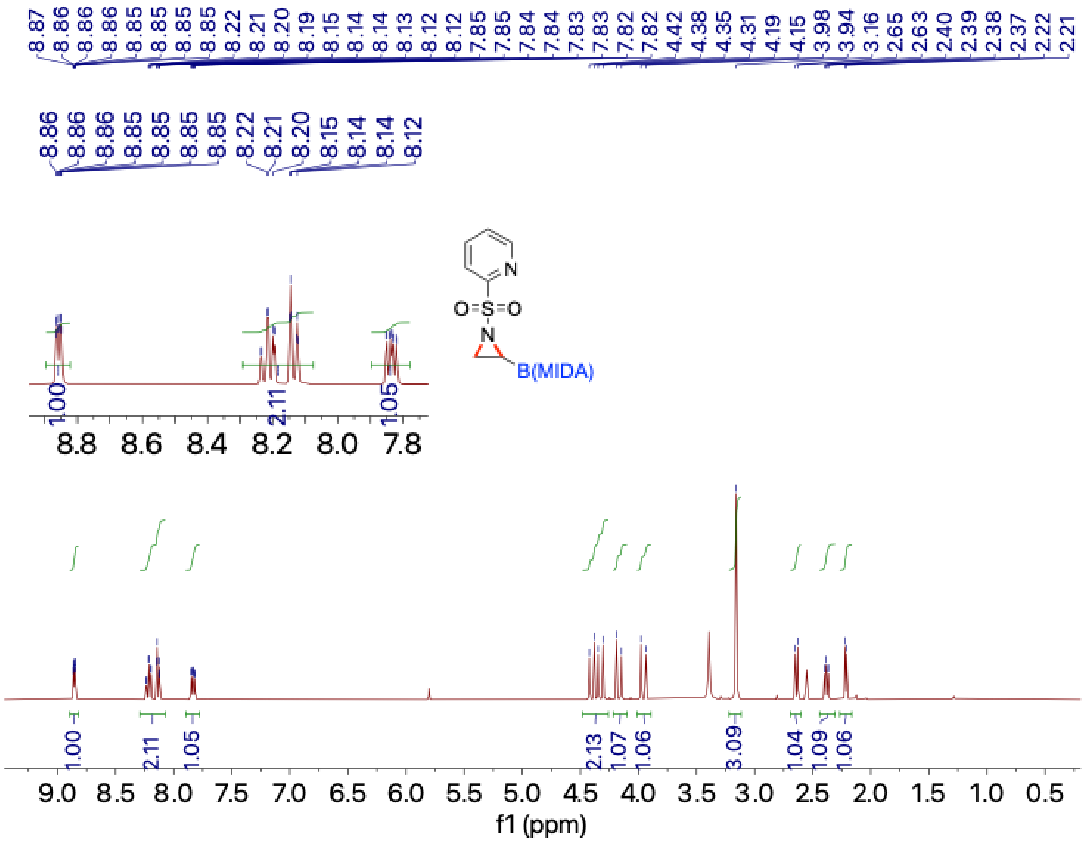
O=S=O
N
B(MIDA)
1.00
2.11
1.05
8.8 8.6 8.4 8.2 8.0 7.8
2.13
1.07
1.06
3.09
1.04
1.09
1.06
9.0 8.5 8.0 7.5 7.0 6.5 6.0 5.5 5.0 4.5 4.0 3.5 3.0 2.5 2.0 1.5 1.0 0.5
f1 (ppm)

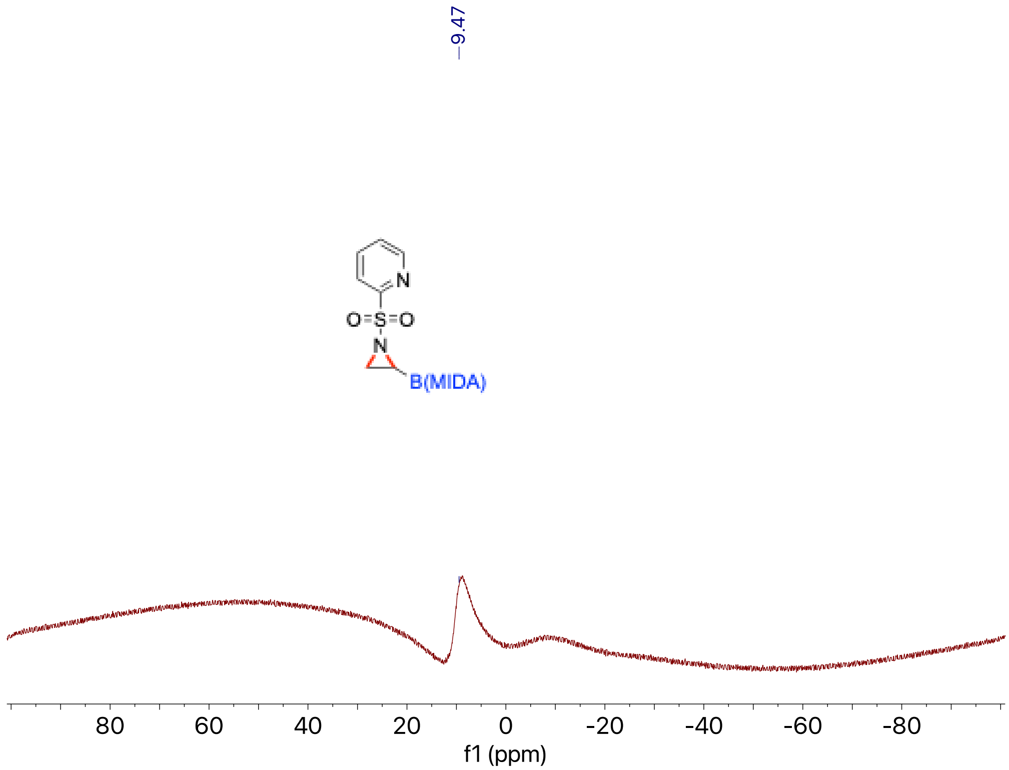
9.47
O=S=O
N
B(MIDA)
80 60 40 20 0 -20 -40 -60 -80
f1 (ppm)

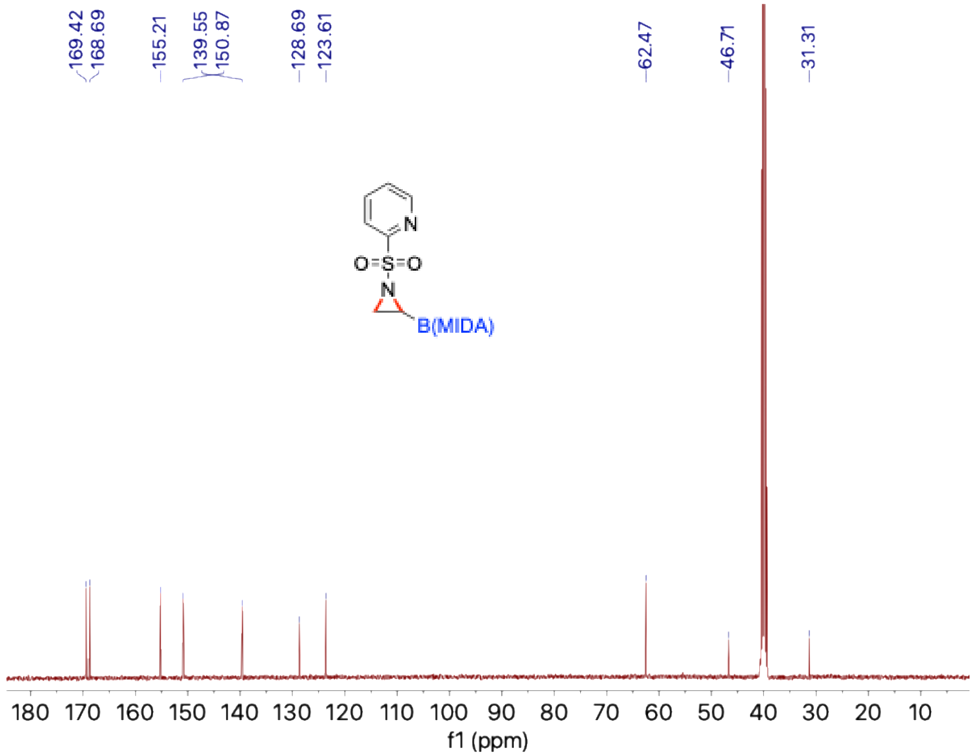
169.42
168.69
155.21
139.55
150.87
128.69
123.61
62.47
46.71
31.31
N
O=S=O
N
B(MIDA)
180 170 160 150 140 130 120 110 100 90 80 70 60 50 40 30 20 10
f1 (ppm)

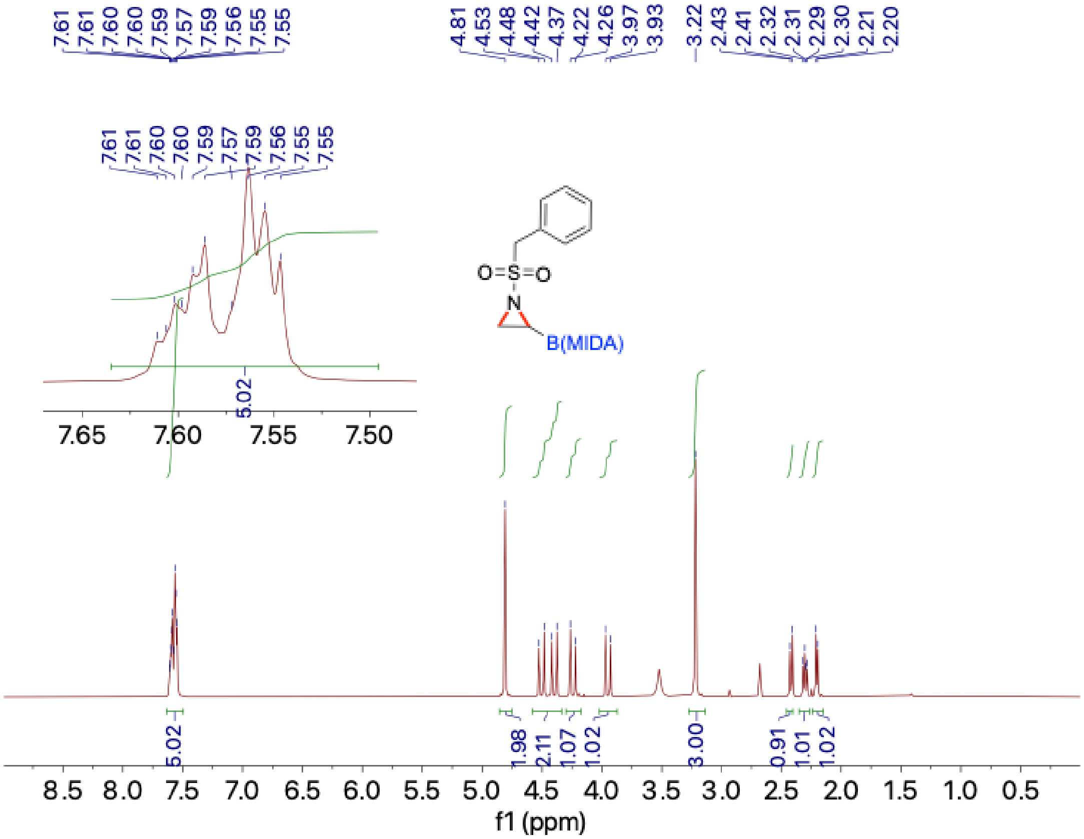
7.61
7.61
7.60
7.60
7.59
7.57
7.59
7.56
7.55
7.55
4.81
4.53
4.48
4.42
4.37
4.22
4.26
3.97
3.93
3.22
2.43
2.41
2.32
2.31
2.29
2.30
2.21
2.20
O=S=O
N
B(MIDA)
5.02
1.98
2.11
1.07
1.02
3.00
0.91
1.01
1.02
f1 (ppm)

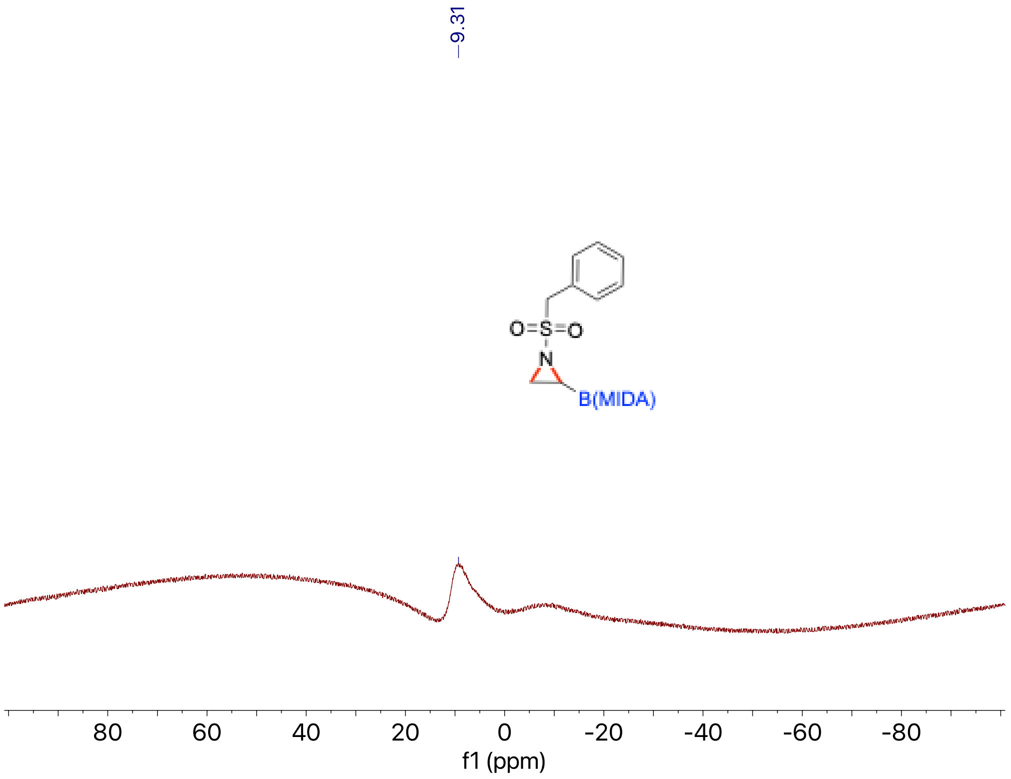
9.31
O=S=O
N
B(MIDA)
f1 (ppm)

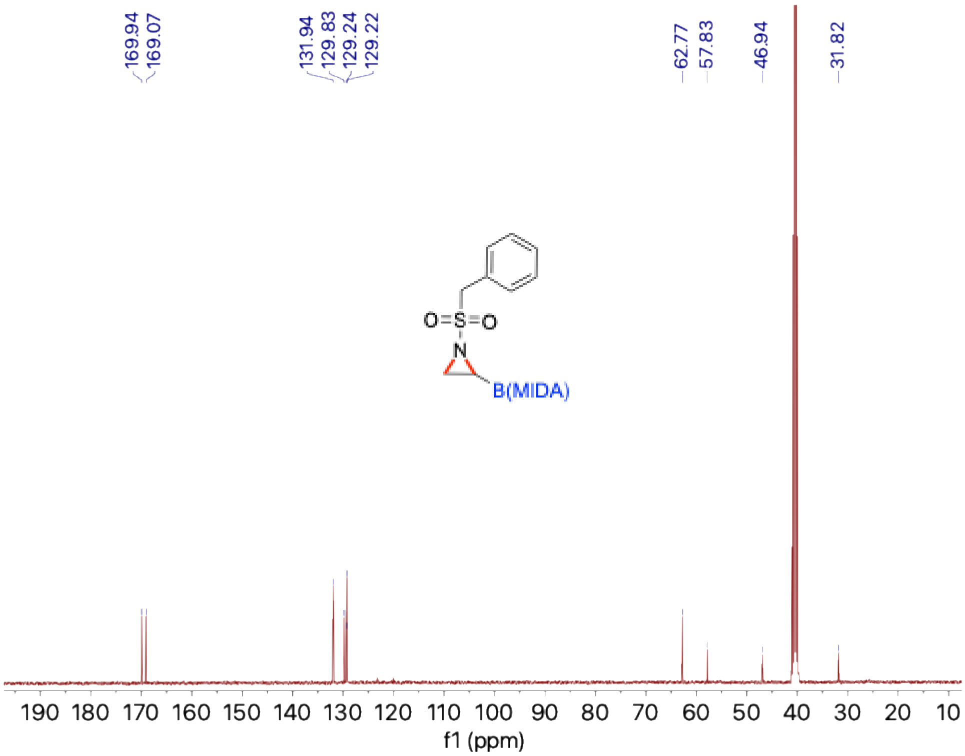
169.94
169.07
131.94
129.83
129.24
129.22
62.77
57.83
46.94
31.82
O=S=O
N
B(MIDA)
190 180 170 160 150 140 130 120 110 100 90 80 70 60 50 40 30 20 10
f1 (ppm)

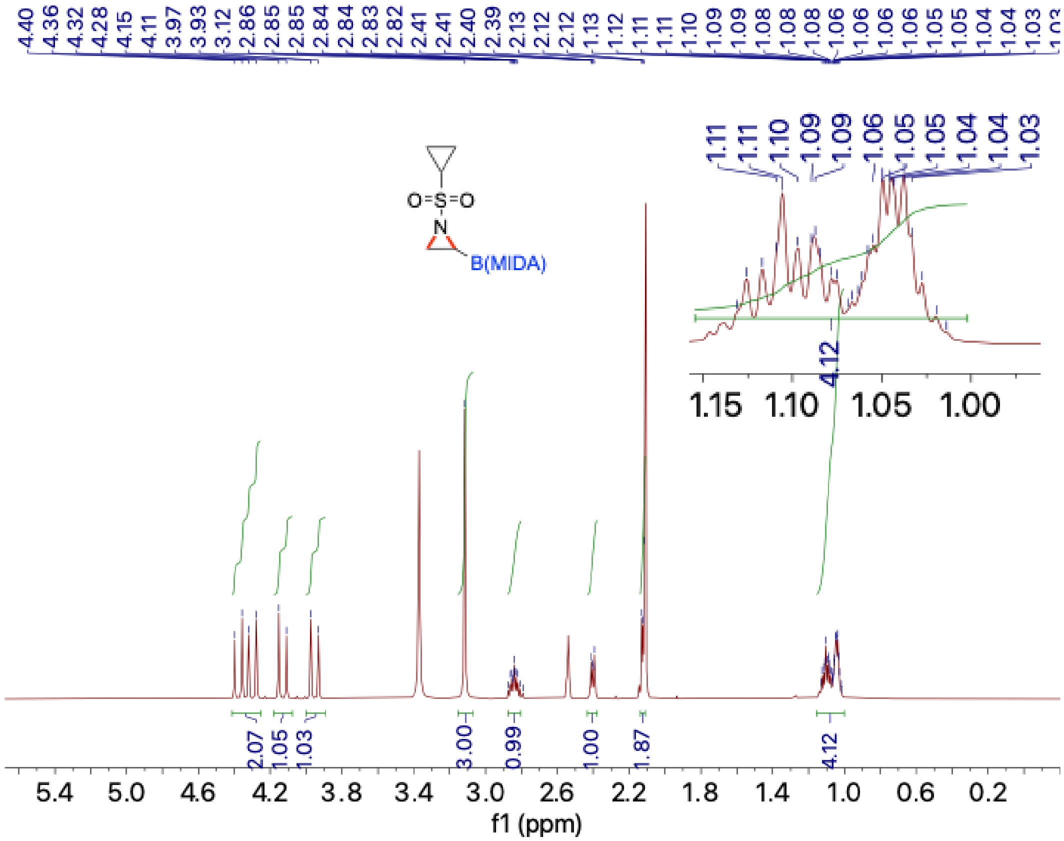
O=S=O
N
B(MIDA)
f1 (ppm)

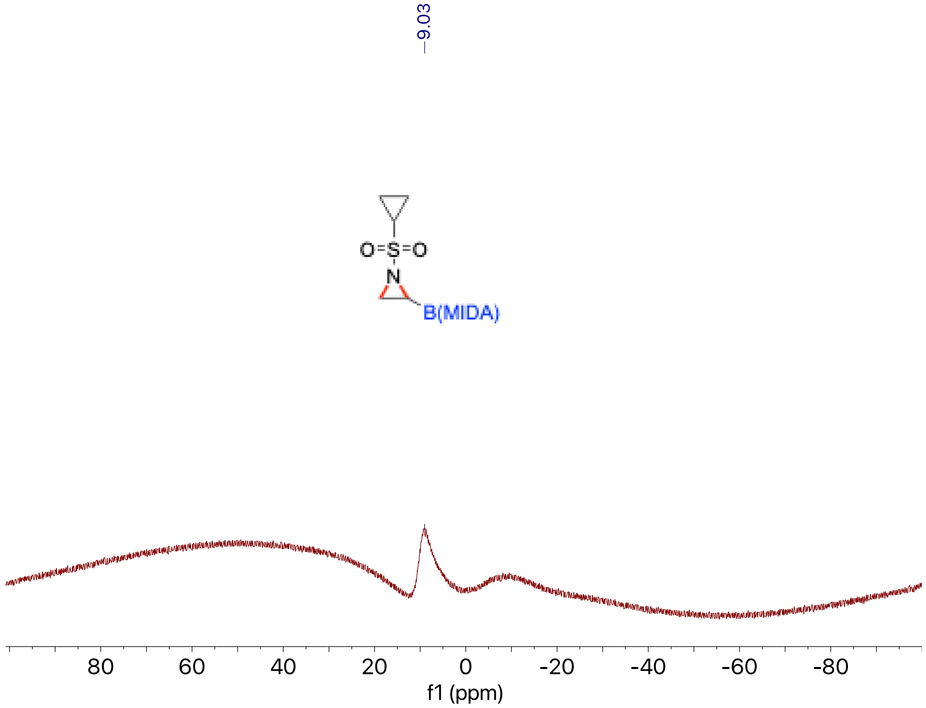
-9.03
O=S=O
N
B(MIDA)
f1 (ppm)

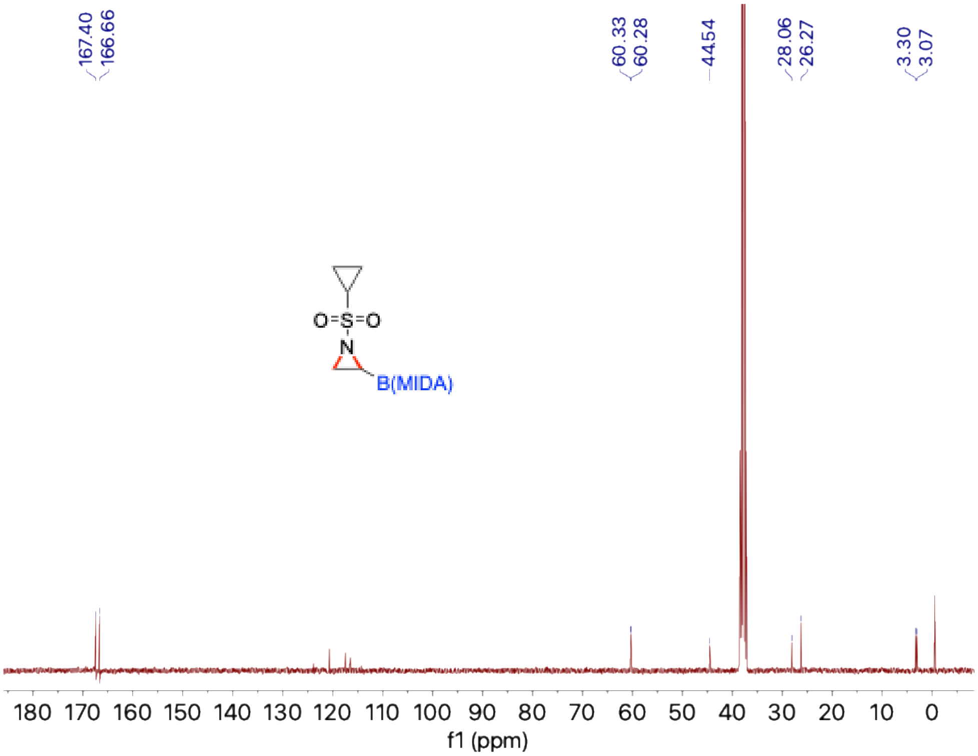
167.40
166.66
60.33
60.28
44.54
28.06
26.27
3.30
3.07
O=S=O
N
B(MIDA)
180 170 160 150 140 130 120 110 100 90 80 70 60 50 40 30 20 10 0
f1 (ppm)

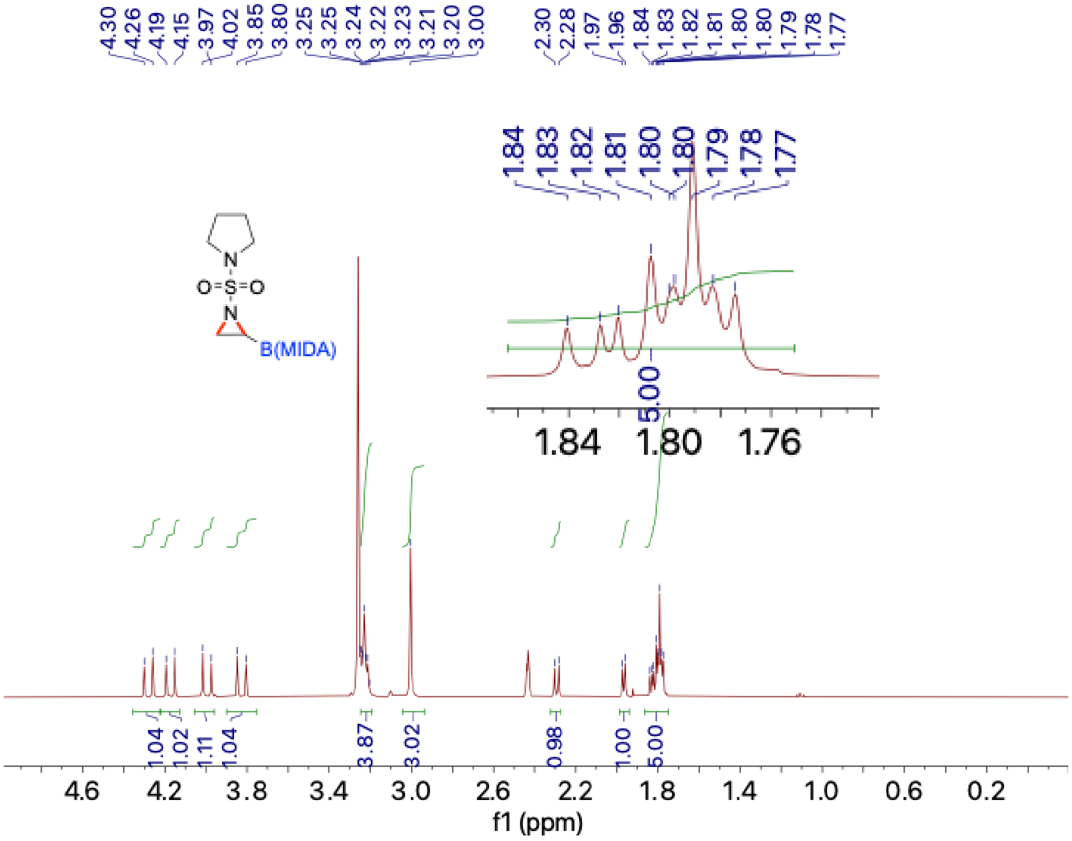

4.30
4.26
4.19
4.15
3.97
4.02
3.85
3.80
3.25
3.25
3.24
3.22
3.23
3.21
3.20
3.00
2.30
2.28
1.97
1.96
1.84
1.83
1.82
1.81
1.80
1.80
1.79
1.78
1.77
O=S=O
N
N
B(MIDA)
1.84
1.83
1.82
1.81
1.80
1.80
1.79
1.78
1.77
5.00
1.84
1.80
1.76
1.04
1.02
1.11
1.04
3.87
3.02
0.98
1.00
5.00
4.6
4.2
3.8
3.4
3.0
2.6
2.2
1.8
1.4
1.0
0.6
0.2
f1 (ppm)

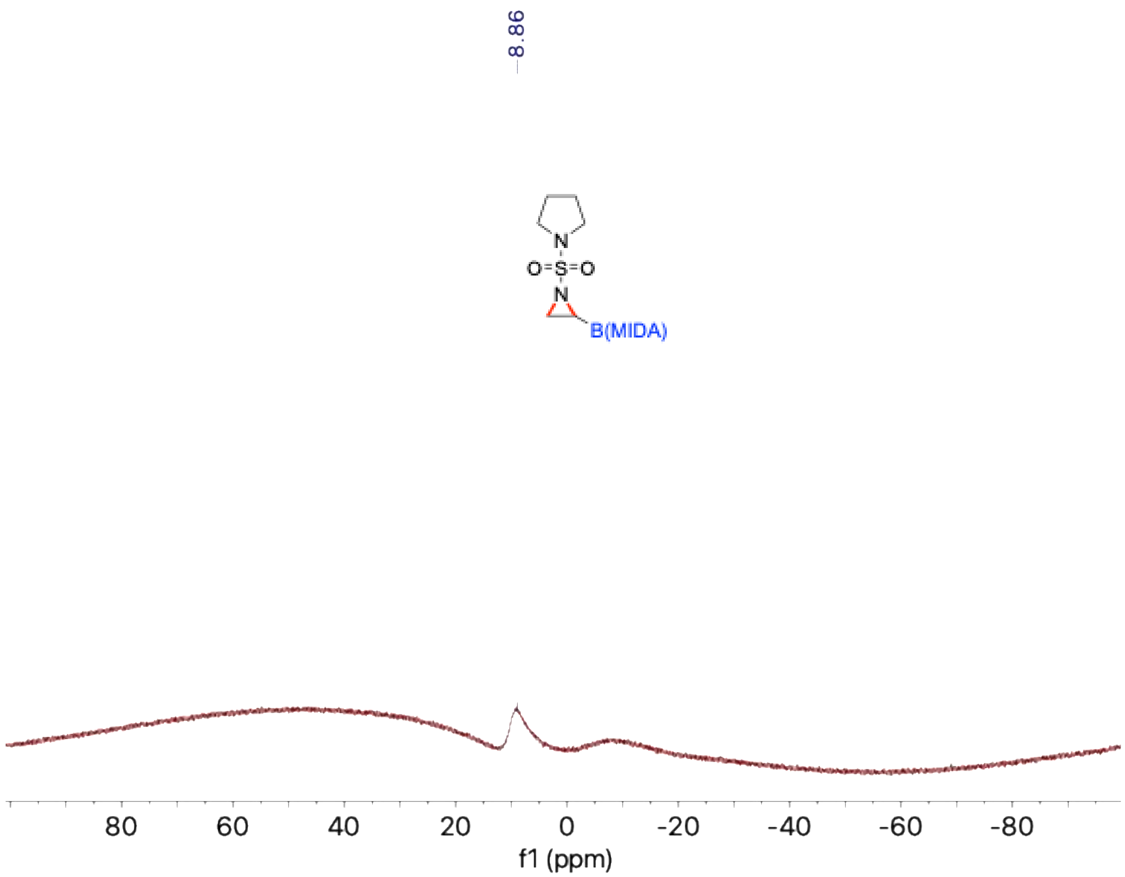
8.86
N
O=S=O
N
B(MIDA)
80 60 40 20 0 -20 -40 -60 -80
f1 (ppm)

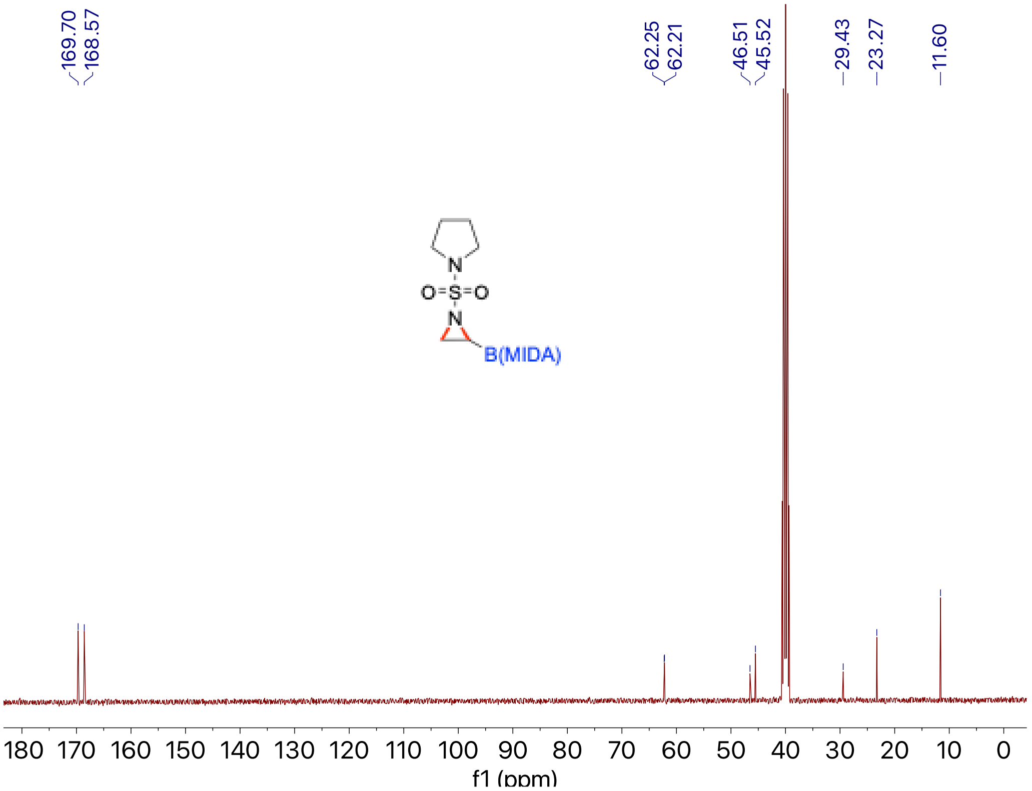
169.70
168.57
62.25
62.21
46.51
45.52
29.43
23.27
11.60
N
O=S=O
N
B(MIDA)
180 170 160 150 140 130 120 110 100 90 80 70 60 50 40 30 20 10 0
f1 (ppm)

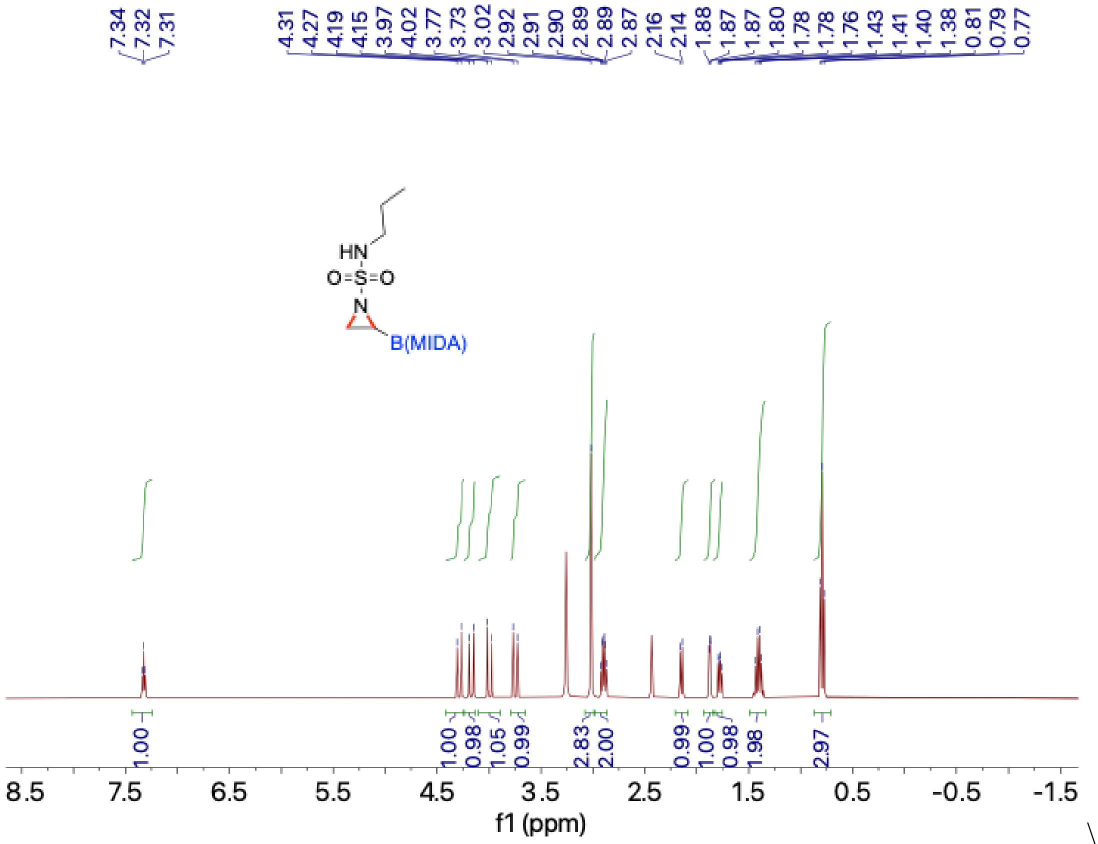

7.34
7.32
7.31
4.31
4.27
4.19
4.15
3.97
4.02
3.77
3.73
3.02
2.92
2.91
2.90
2.89
2.89
2.87
2.16
2.14
1.88
1.87
1.87
1.80
1.78
1.78
1.76
1.43
1.41
1.40
1.38
0.81
0.79
0.77
HN
O=S=O
N
B(MIDA)
1.00
1.00
0.98
1.05
0.99
2.83
2.00
0.99
1.00
0.98
1.98
2.97
8.5
7.5
6.5
5.5
4.5
3.5
2.5
1.5
0.5
-0.5
-1.5
f1 (ppm)

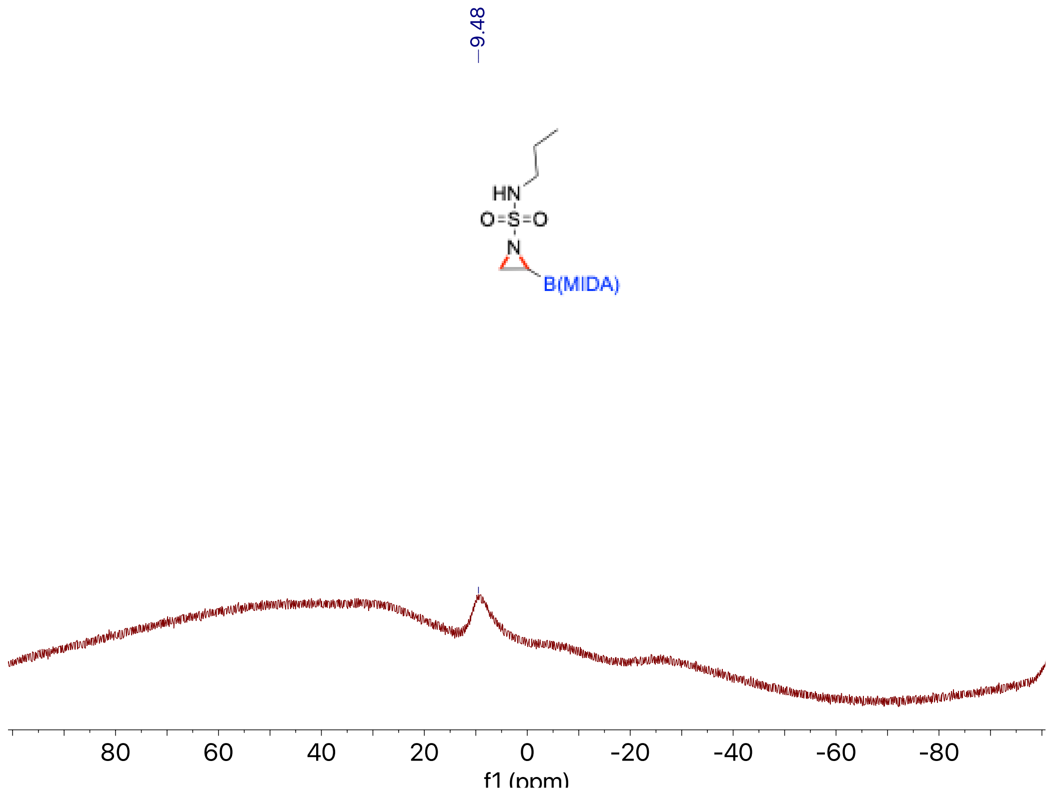

9.48
HN
O=S=O
N
B(MIDA)
80
60
40
20
0
-20
-40
-60
-80
f1 (ppm)

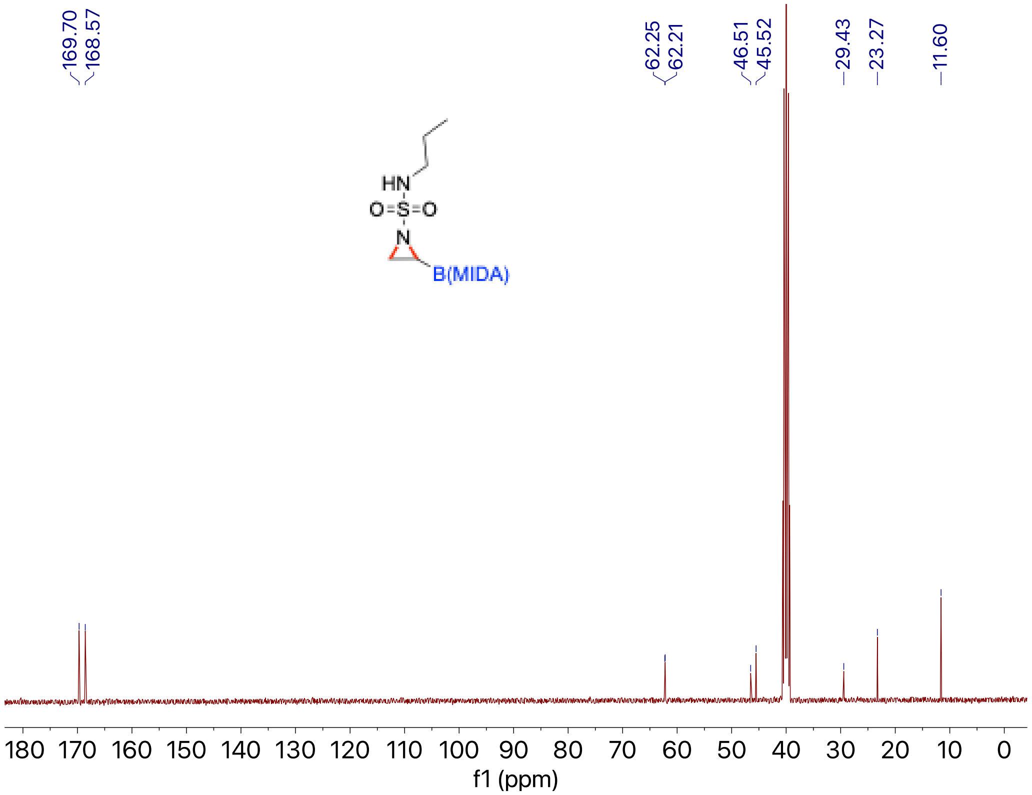

169.70
168.57
62.25
62.21
46.51
45.52
29.43
23.27
11.60
HN
O=S=O
N
B(MIDA)
180 170 160 150 140 130 120 110 100 90 80 70 60 50 40 30 20 10 0
f1 (ppm)

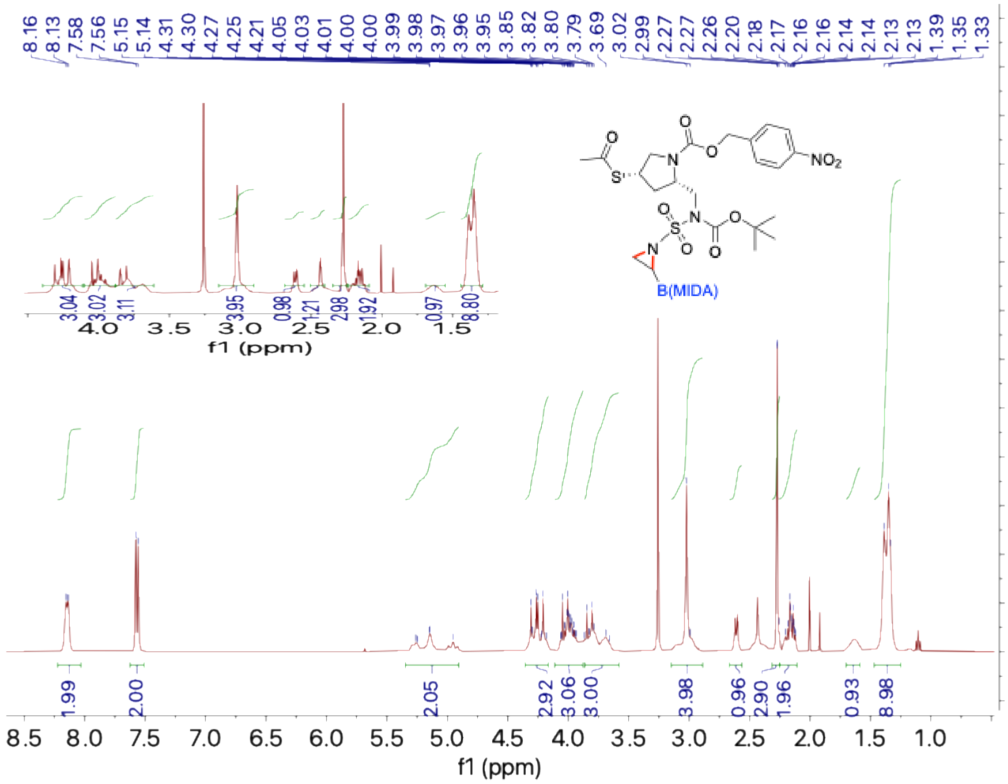

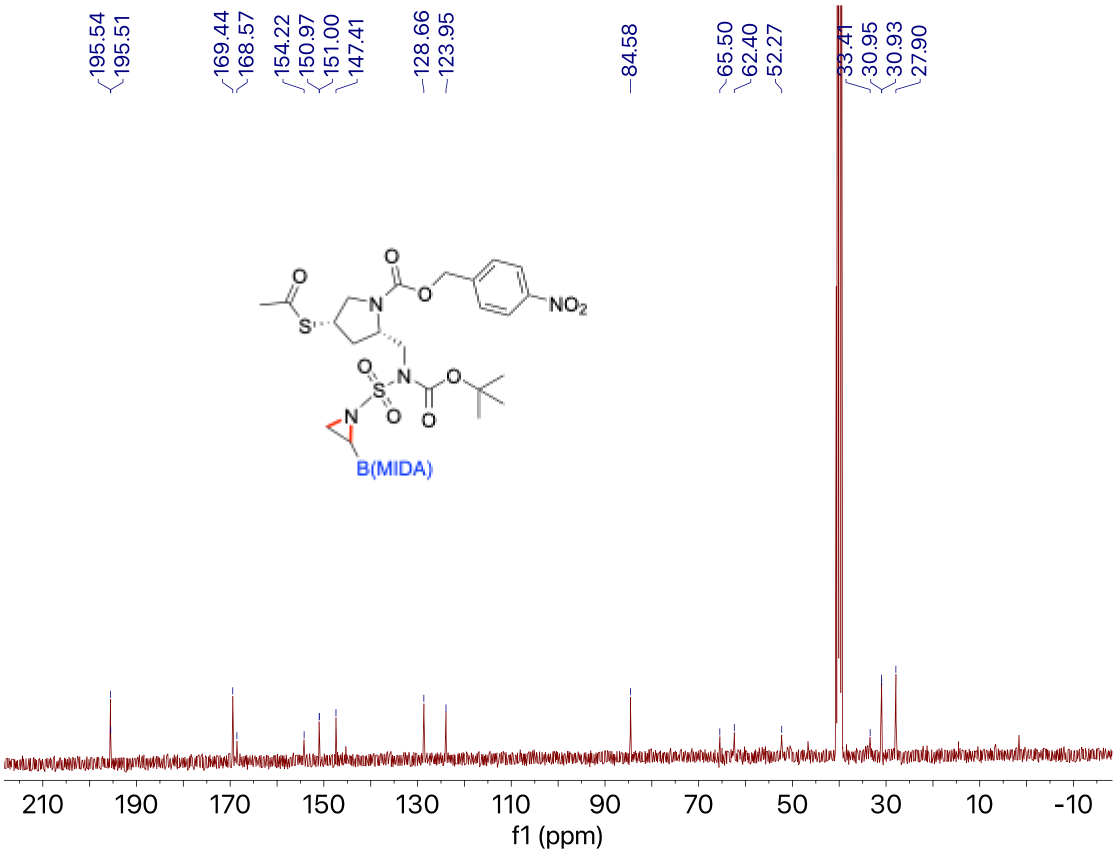

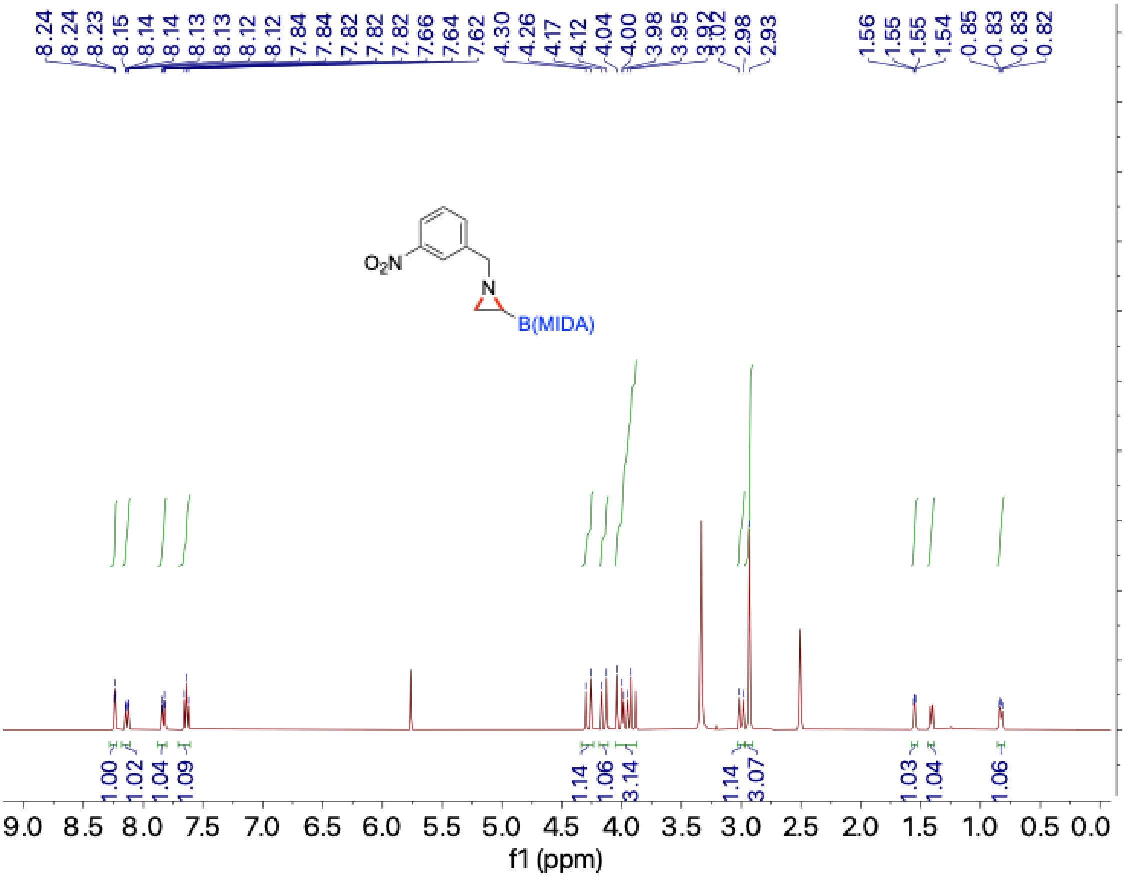
8.24
8.24
8.23
8.15
8.14
8.14
8.13
8.13
8.12
8.12
7.84
7.84
7.82
7.82
7.82
7.66
7.64
7.62
4.30
4.26
4.17
4.12
4.04
4.00
3.98
3.95
3.92
3.02
2.98
2.93
1.56
1.55
1.55
1.54
0.85
0.83
0.83
0.82
O2N
N
B(MIDA)
1.00
1.02
1.04
1.09
1.14
1.06
3.14
1.14
3.07
1.03
1.04
1.06
9.0 8.5 8.0 7.5 7.0 6.5 6.0 5.5 5.0 4.5 4.0 3.5 3.0 2.5 2.0 1.5 1.0 0.5 0.0
f1 (ppm)

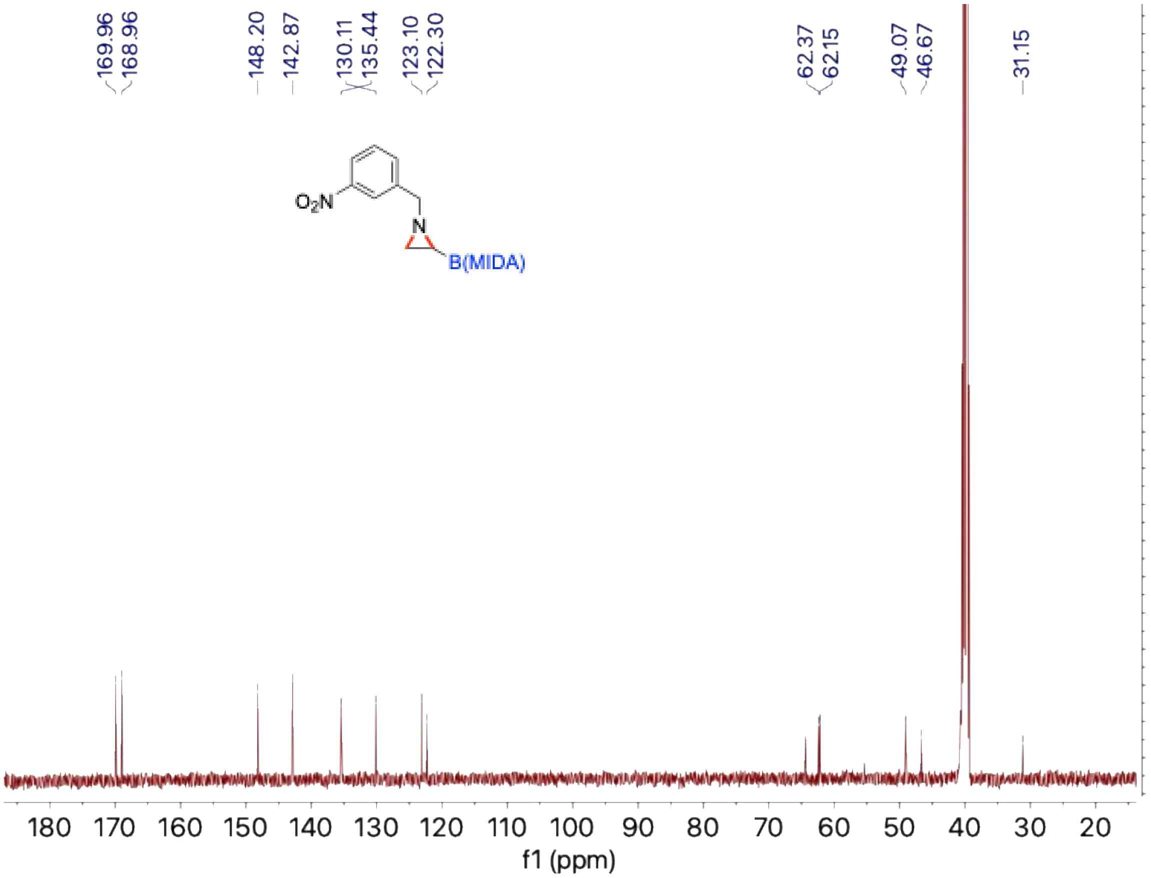

03-20-2024-RY-1-A63-Carbon.12.fid

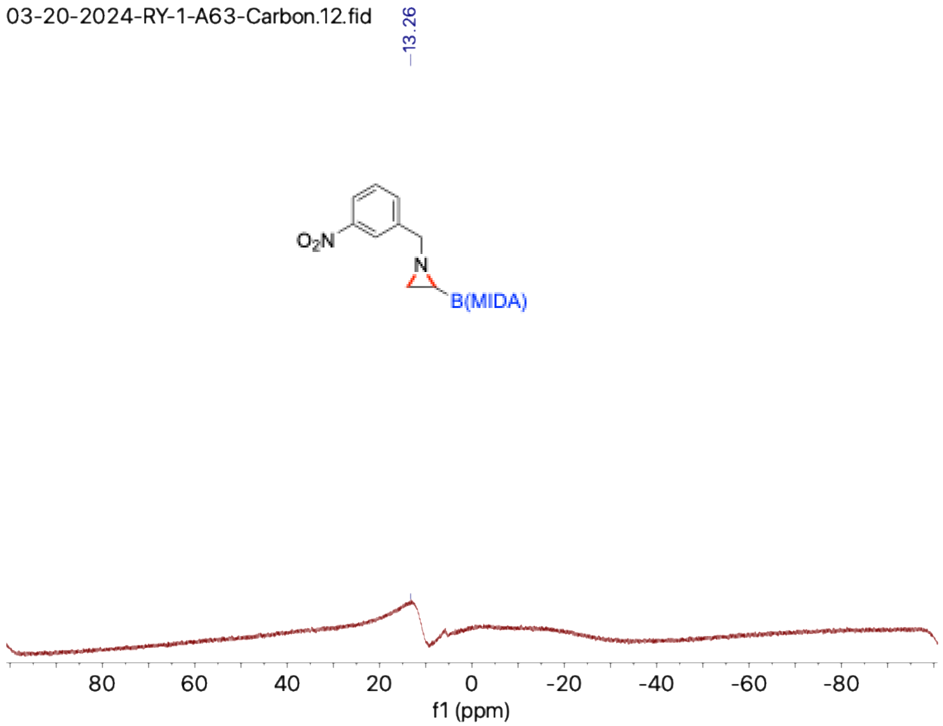

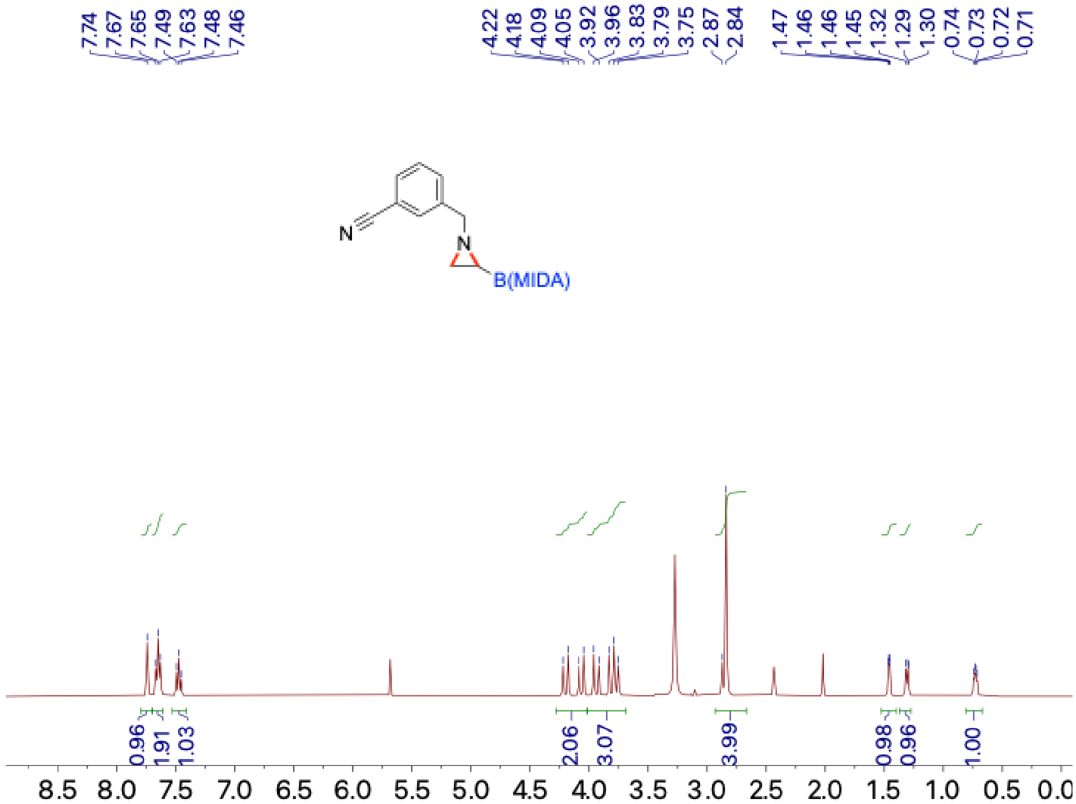
7.74
7.67
7.65
7.49
7.63
7.48
7.46
4.22
4.18
4.09
4.05
3.92
3.96
3.83
3.79
3.75
2.87
2.84
1.47
1.46
1.46
1.45
1.32
1.29
1.30
0.74
0.73
0.72
0.71
N
N
B(MIDA)
0.96
1.91
1.03
2.06
3.07
3.99
0.98
0.96
1.00
8.5 8.0 7.5 7.0 6.5 6.0 5.5 5.0 4.5 4.0 3.5 3.0 2.5 2.0 1.5 1.0 0.5 0.0

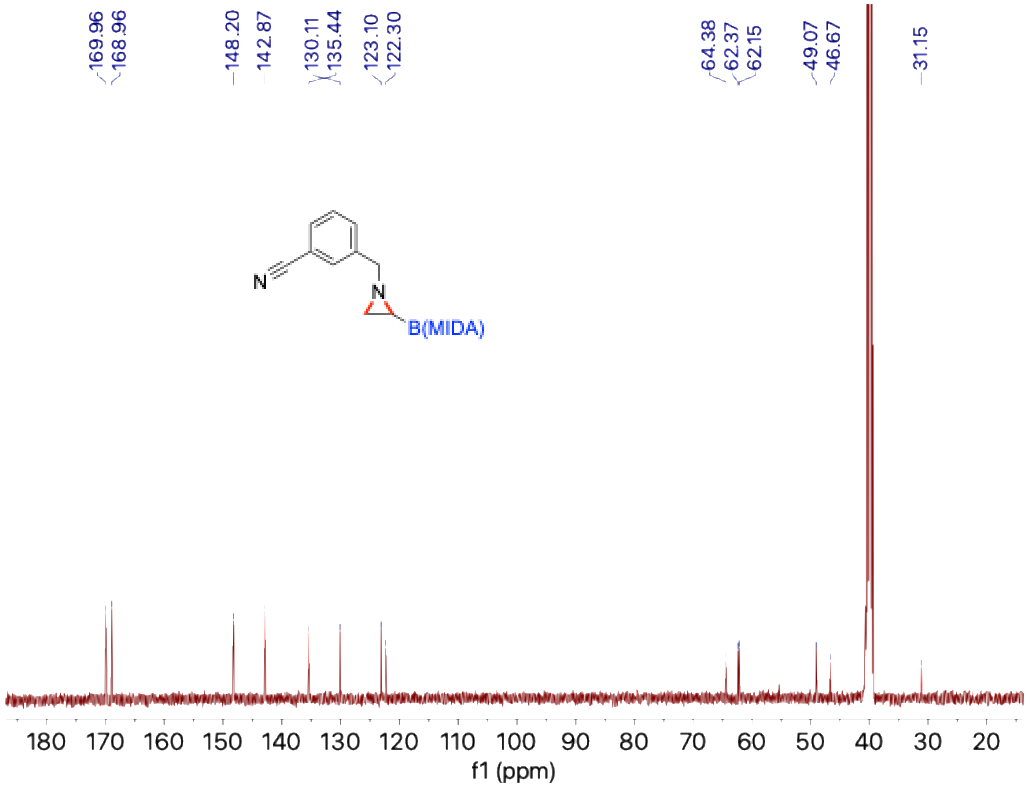
169.96
168.96
148.20
142.87
130.11
135.44
123.10
122.30
64.38
62.37
62.15
49.07
46.67
31.15
N
N
B(MIDA)
180 170 160 150 140 130 120 110 100 90 80 70 60 50 40 30 20
f1 (ppm)

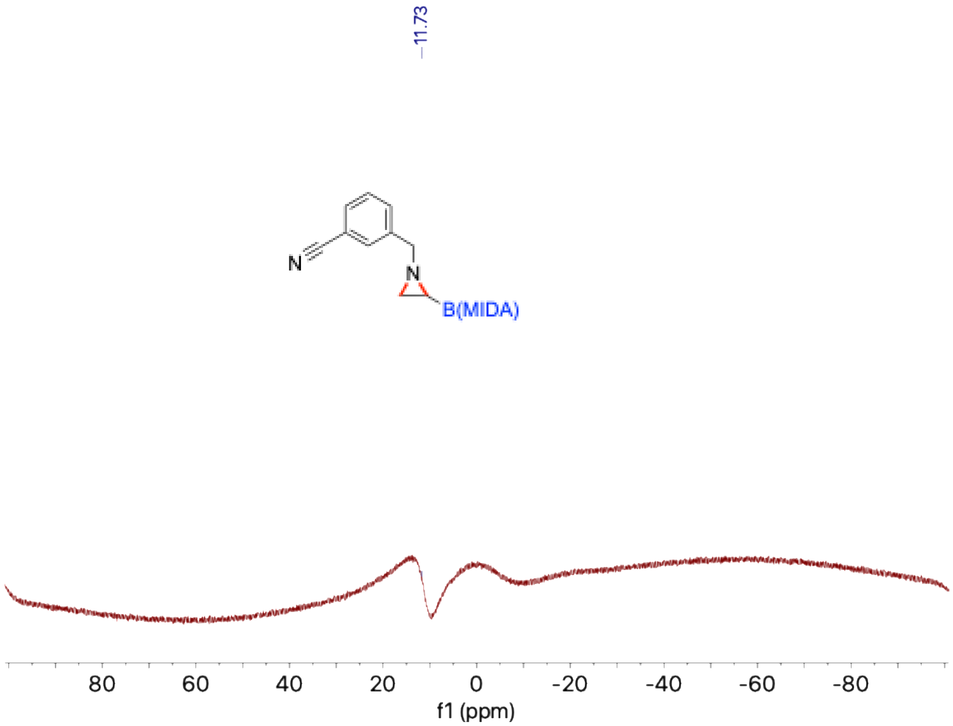
11.73
N
N
B(MIDA)
80 60 40 20 0 -20 -40 -60 -80
f1 (ppm)

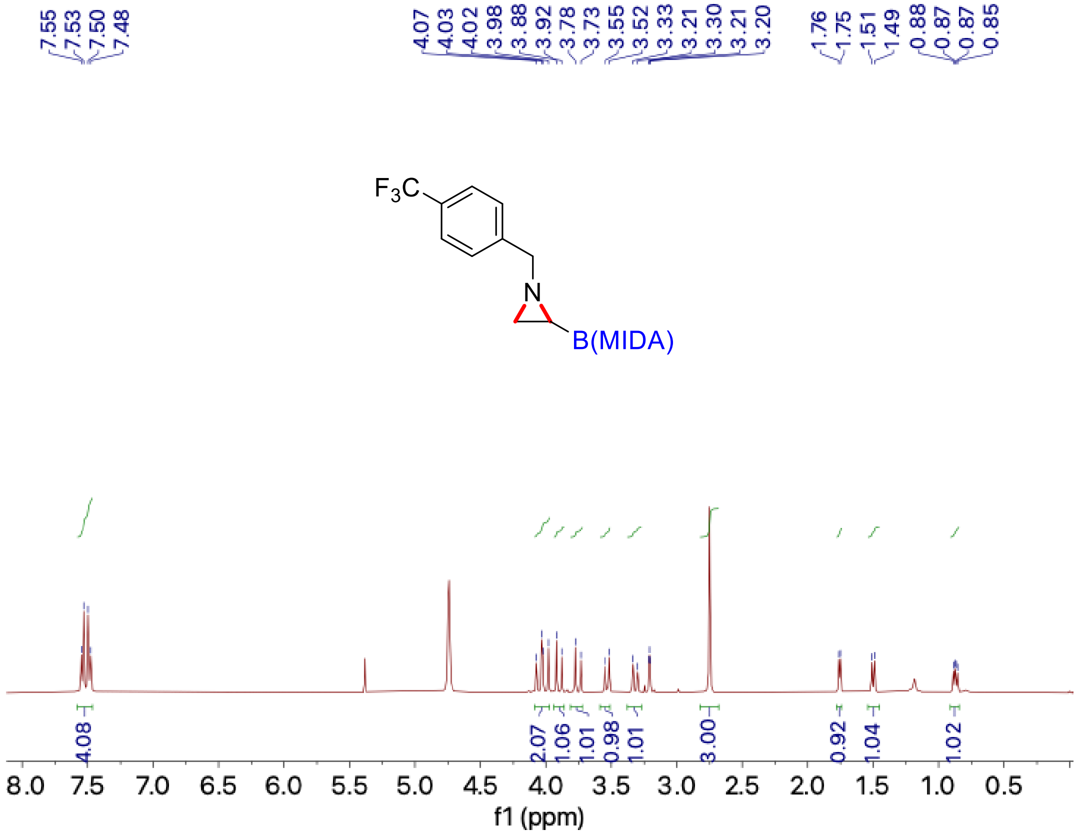
7.55
7.53
7.50
7.48
4.07
4.03
4.02
3.98
3.88
3.92
3.78
3.73
3.55
3.52
3.33
3.21
3.30
3.21
3.20
1.76
1.75
1.51
1.49
0.88
0.87
0.87
0.85
F3C
N
B(MIDA)
4.08
2.07
1.06
1.01
0.98
1.01
3.00
0.92
1.04
1.02
8.0 7.5 7.0 6.5 6.0 5.5 5.0 4.5 4.0 3.5 3.0 2.5 2.0 1.5 1.0 0.5
f1 (ppm)

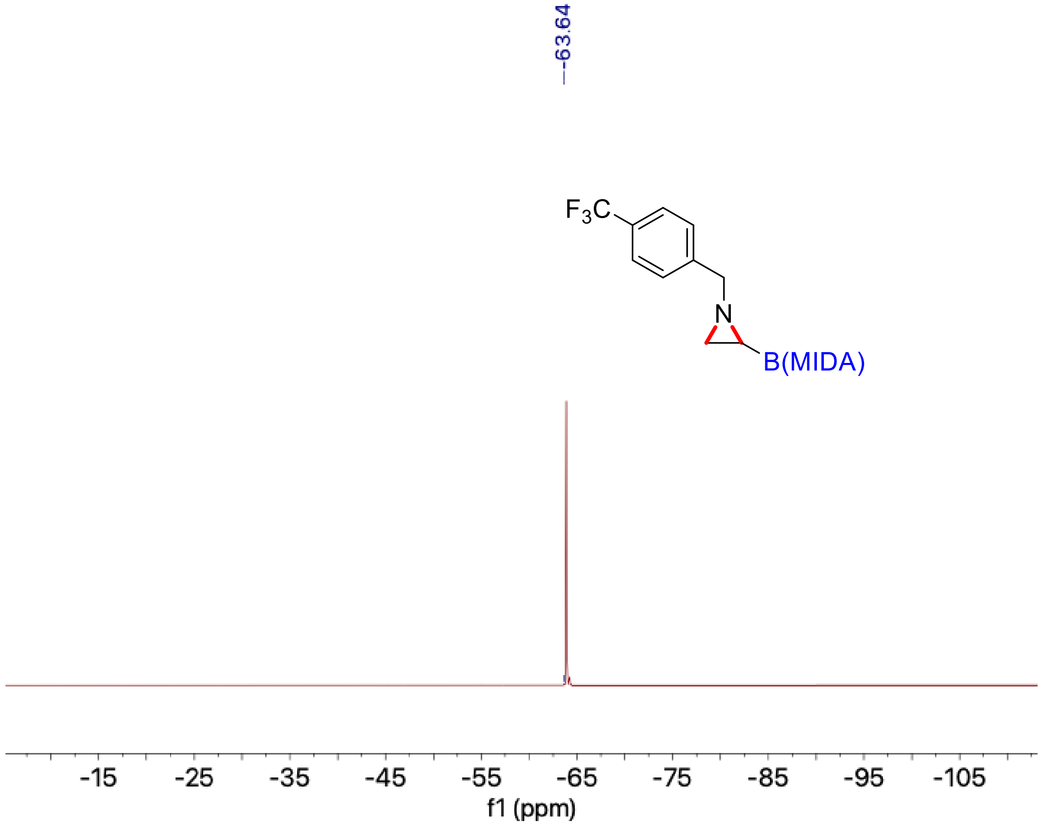
-63.64
F3C
N
B(MIDA)
-15 -25 -35 -45 -55 -65 -75 -85 -95 -105
f1 (ppm)

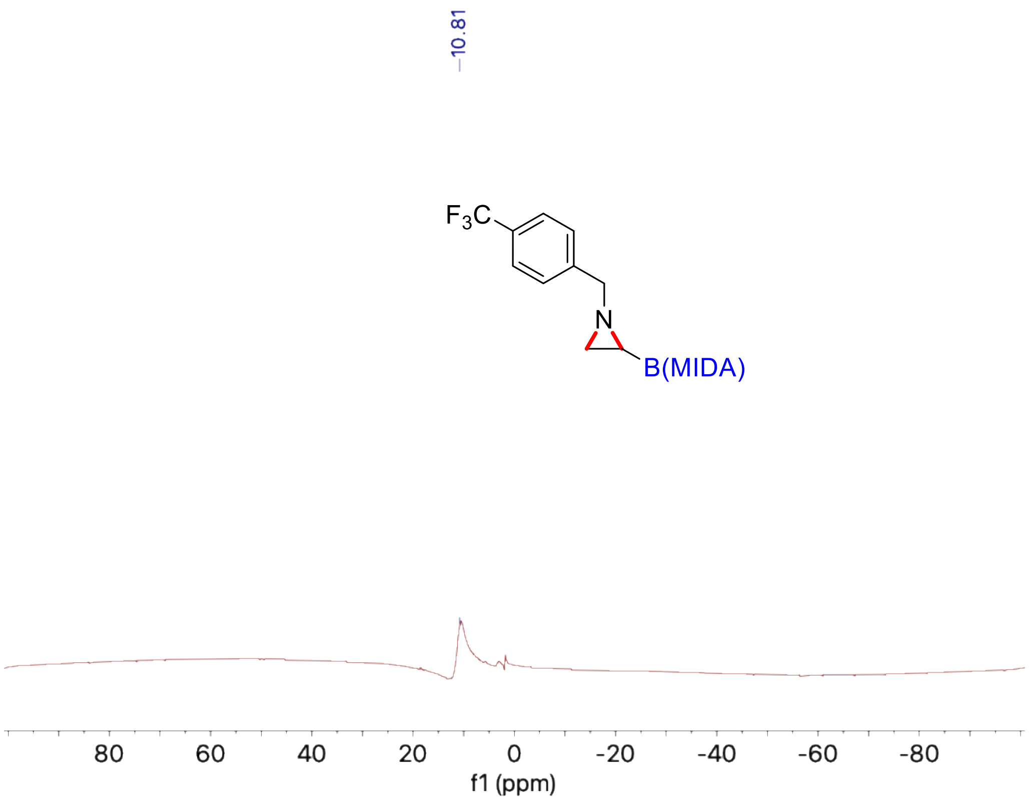

-10.81
F3C
N
B(MIDA)
80
60
40
20
0
-20
-40
-60
-80
f1 (ppm)

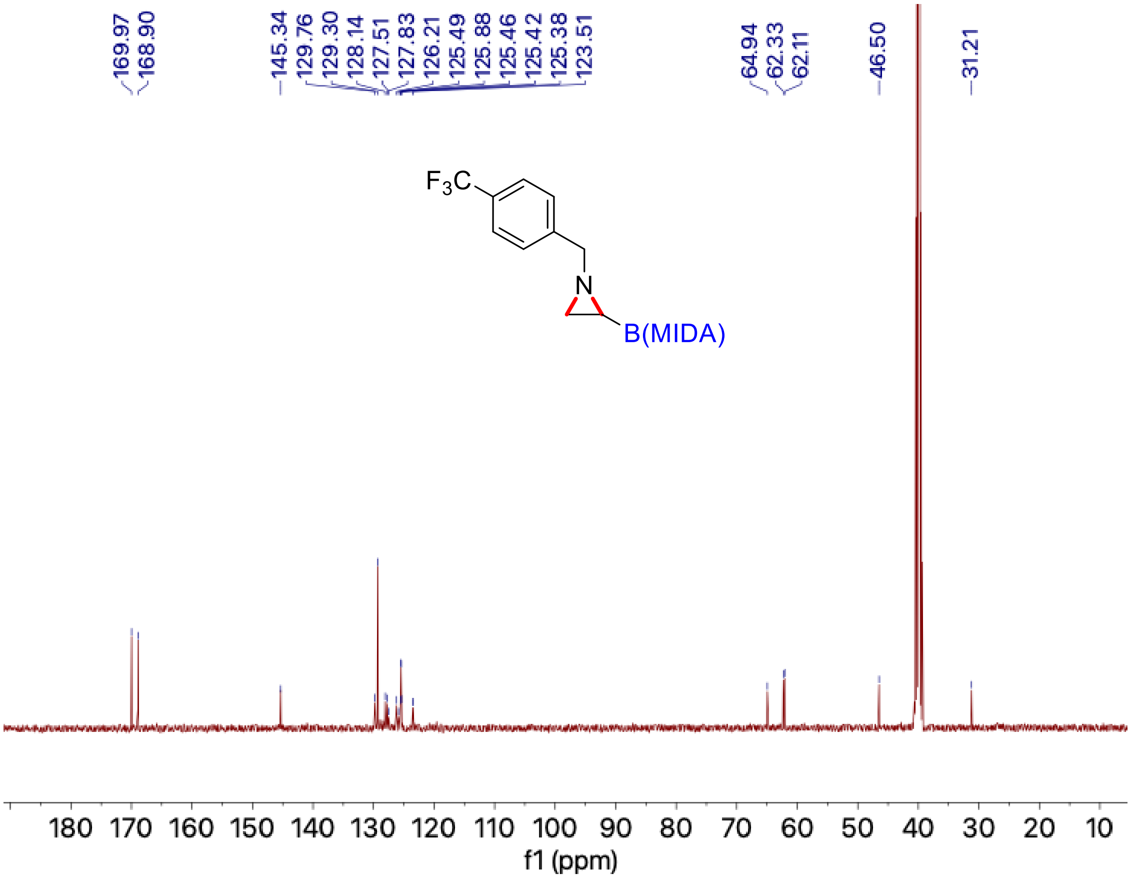

169.97
168.90
145.34
129.76
129.30
128.14
127.51
127.83
126.21
125.49
125.88
125.46
125.42
125.38
123.51
64.94
62.33
62.11
46.50
31.21
F3C
N
B(MIDA)
180
170
160
150
140
130
120
110
100
90
80
70
60
50
40
30
20
10
f1 (ppm)

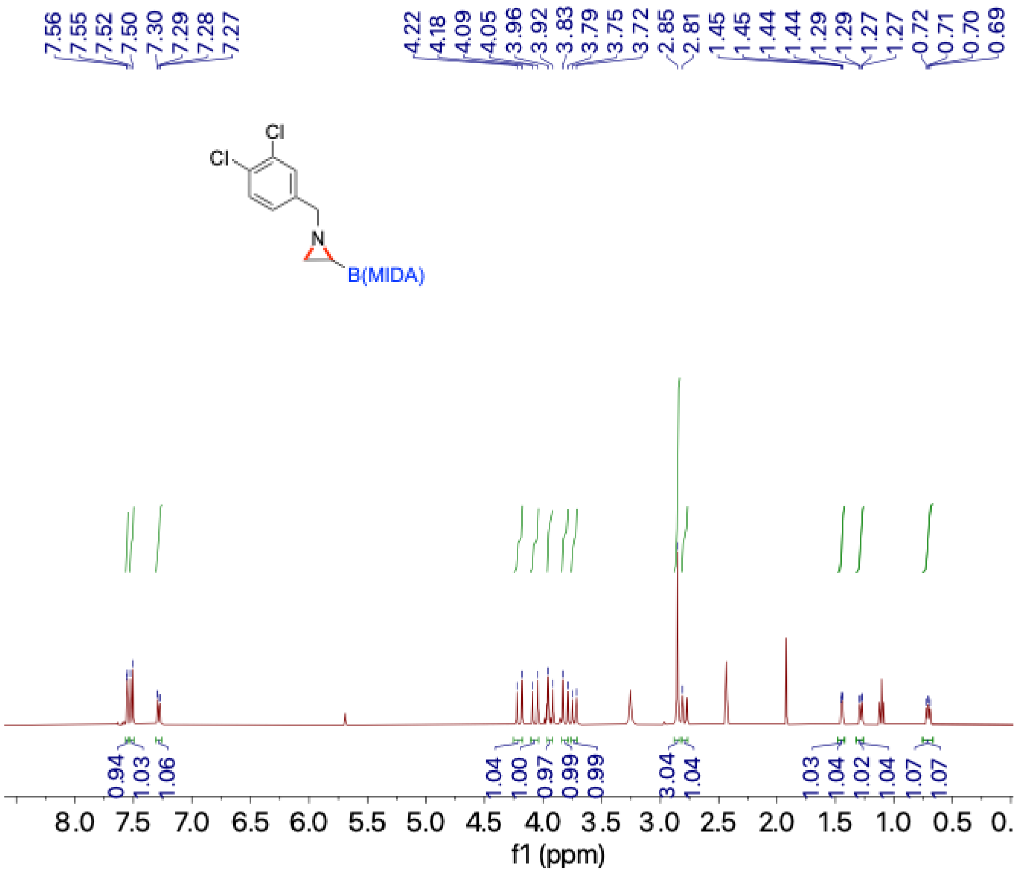

7.56 7.55 7.52 7.50 7.30 7.29 7.28 7.27
4.22 4.18 4.09 4.05 3.96 3.92 3.83 3.79 3.75 3.72 2.85 2.81 1.45 1.45 1.44 1.44 1.29 1.29 1.27 1.27 0.72 0.71 0.70 0.69
Cl
Cl
N
B(MIDA)
0.94 1.03 1.06
1.04 1.00 0.97 0.99 0.99
3.04 1.04
1.03 1.04 1.02 1.04 1.07 1.07
8.0 7.5 7.0 6.5 6.0 5.5 5.0 4.5 4.0 3.5 3.0 2.5 2.0 1.5 1.0 0.5 0.
f1 (ppm)

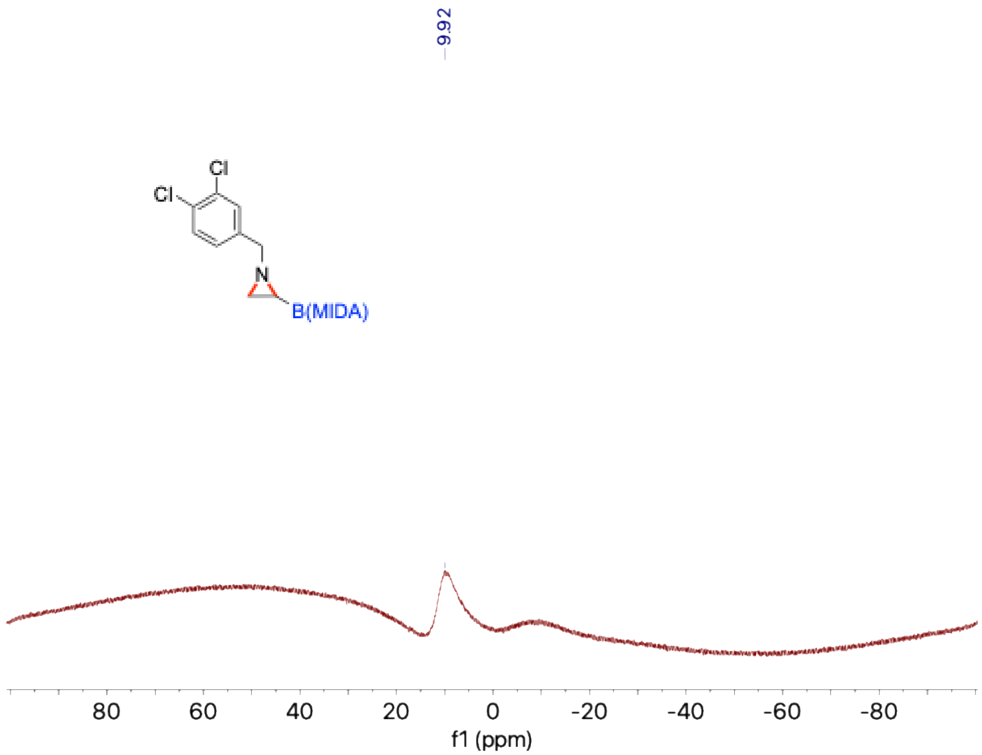
9.92
Cl
Cl
N
B(MIDA)
80
60
40
20
0
-20
-40
-60
-80
f1 (ppm)

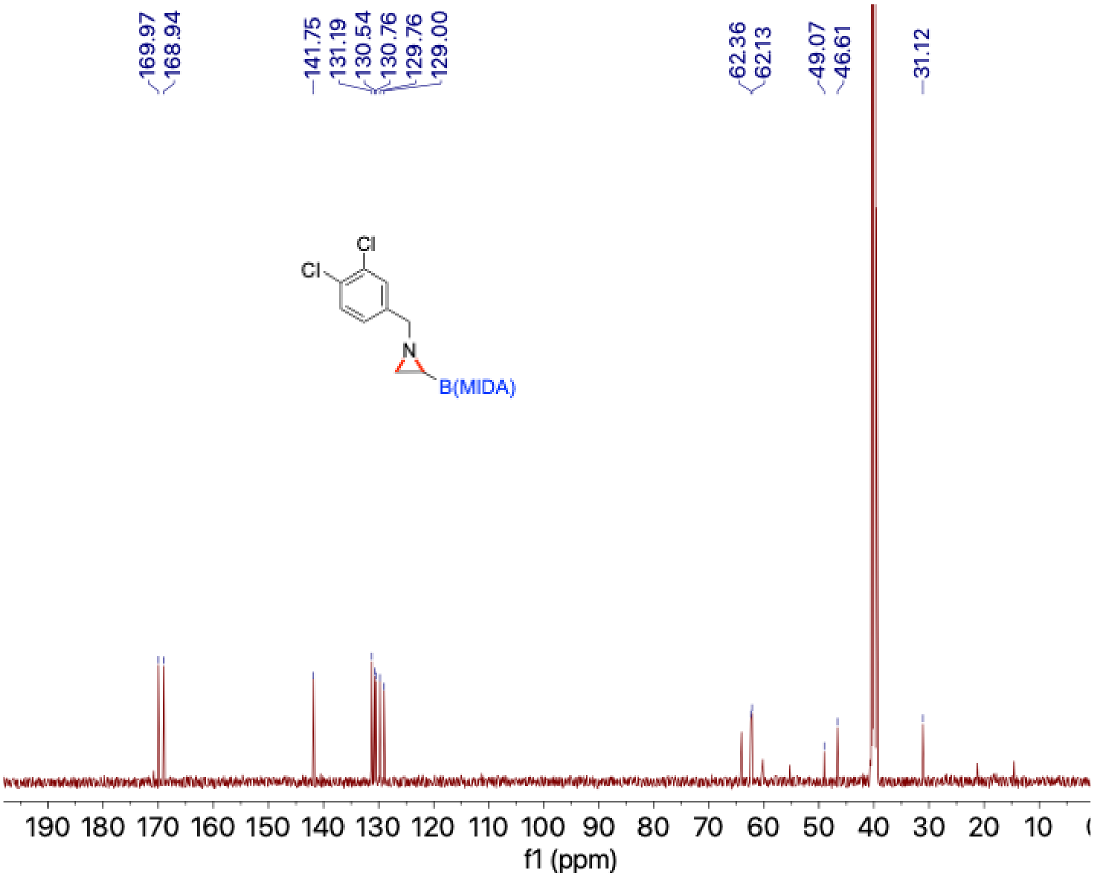
169.97
168.94
141.75
131.19
130.54
130.76
129.76
129.00
62.36
62.13
49.07
46.61
31.12
Cl
Cl
N
B(MIDA)
190
180
170
160
150
140
130
120
110
100
90
80
70
60
50
40
30
20
10
f1 (ppm)

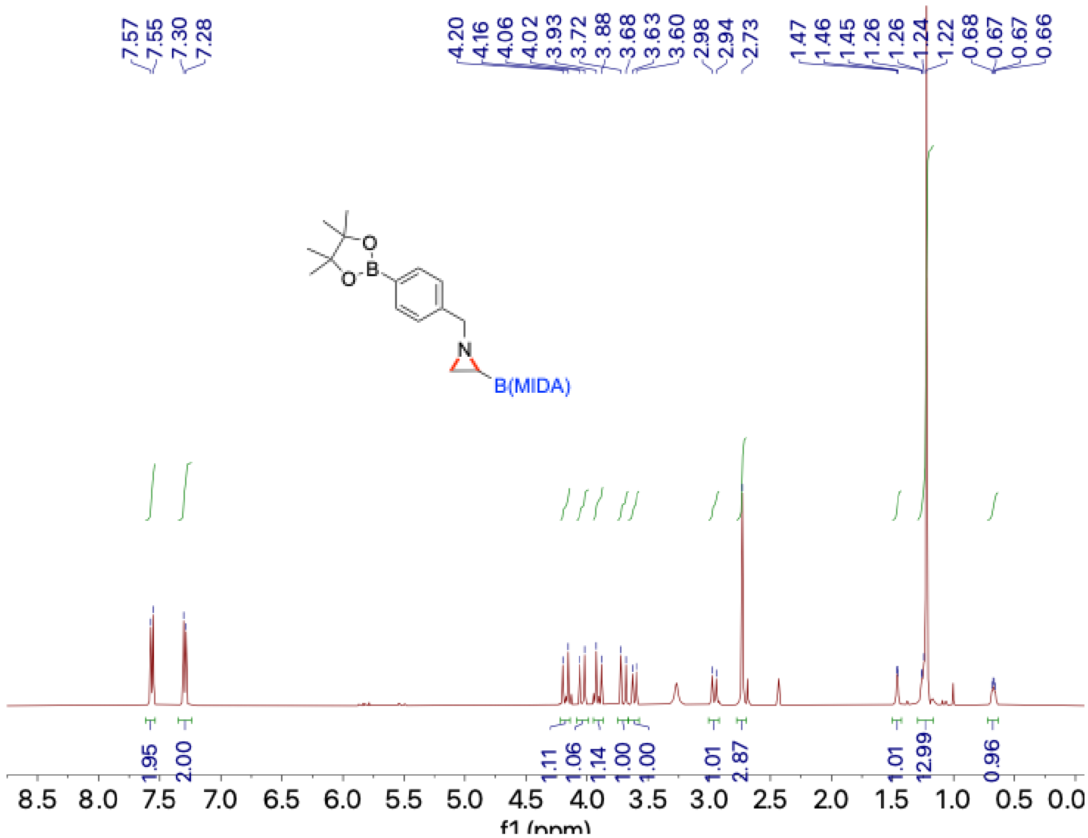

7.57
7.55
7.30
7.28
4.20
4.16
4.06
4.02
3.93
3.72
3.88
3.68
3.63
3.60
2.98
2.94
2.73
1.47
1.46
1.45
1.26
1.26
1.24
1.22
0.68
0.67
0.67
0.66
O
B
O
N
B(MIDA)
1.95
2.00
1.11
1.06
1.14
1.00
1.00
1.01
2.87
1.01
12.99
0.96
8.5 8.0 7.5 7.0 6.5 6.0 5.5 5.0 4.5 4.0 3.5 3.0 2.5 2.0 1.5 1.0 0.5 0.0
f1 (ppm)

VB-F-131-1.11.fid

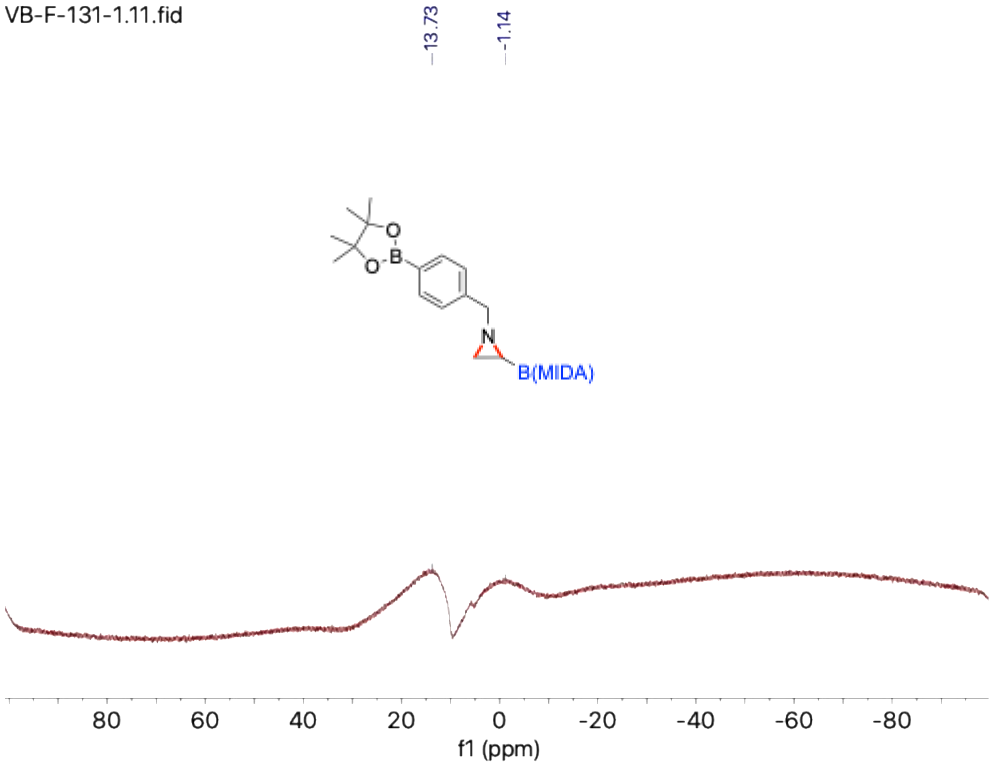

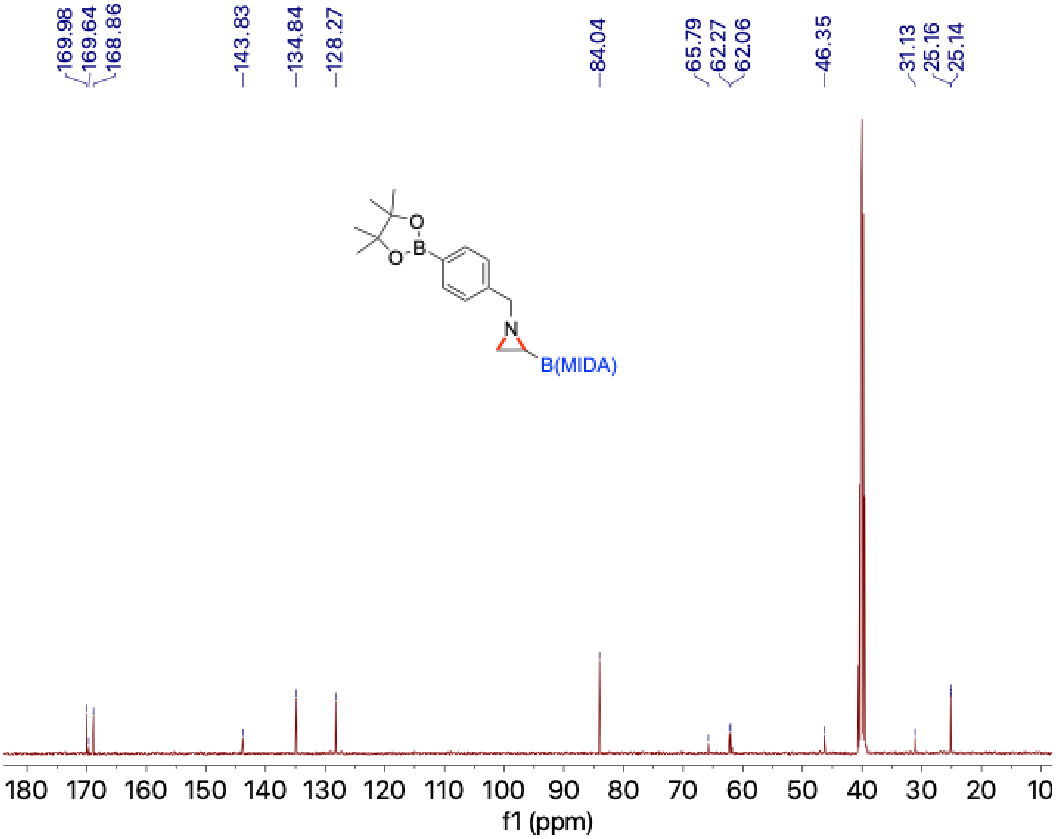

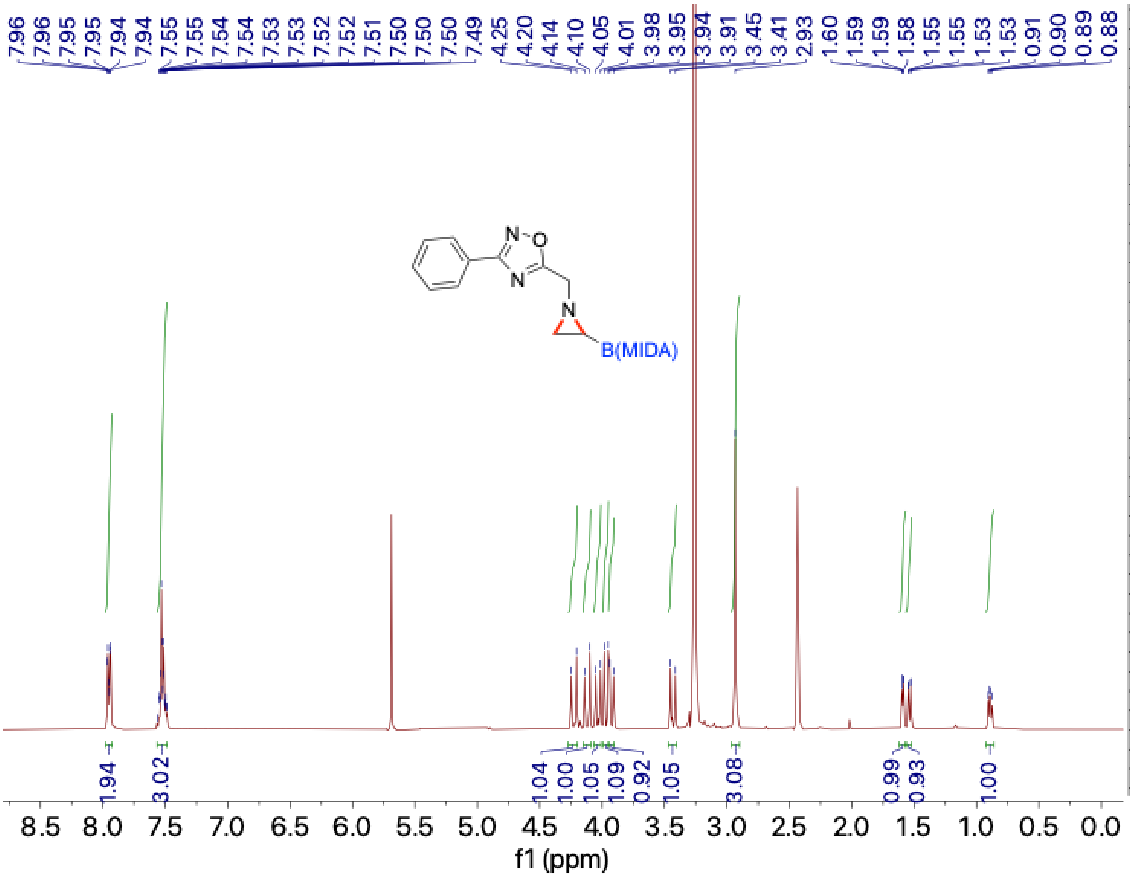

7.96
7.96
7.95
7.95
7.94
7.94
7.55
7.55
7.54
7.54
7.53
7.53
7.52
7.52
7.51
7.50
7.50
7.49
4.25
4.20
4.14
4.10
4.05
4.01
3.98
3.95
3.94
3.91
3.45
3.41
2.93
1.60
1.59
1.59
1.58
1.55
1.55
1.53
1.53
0.91
0.90
0.89
0.88
N
O
N
N
B(MIDA)
1.94
3.02
1.04
1.00
1.05
1.09
0.92
1.05
3.08
0.99
0.93
1.00
8.5
8.0
7.5
7.0
6.5
6.0
5.5
5.0
4.5
4.0
3.5
3.0
2.5
2.0
1.5
1.0
0.5
0.0
f1 (ppm)

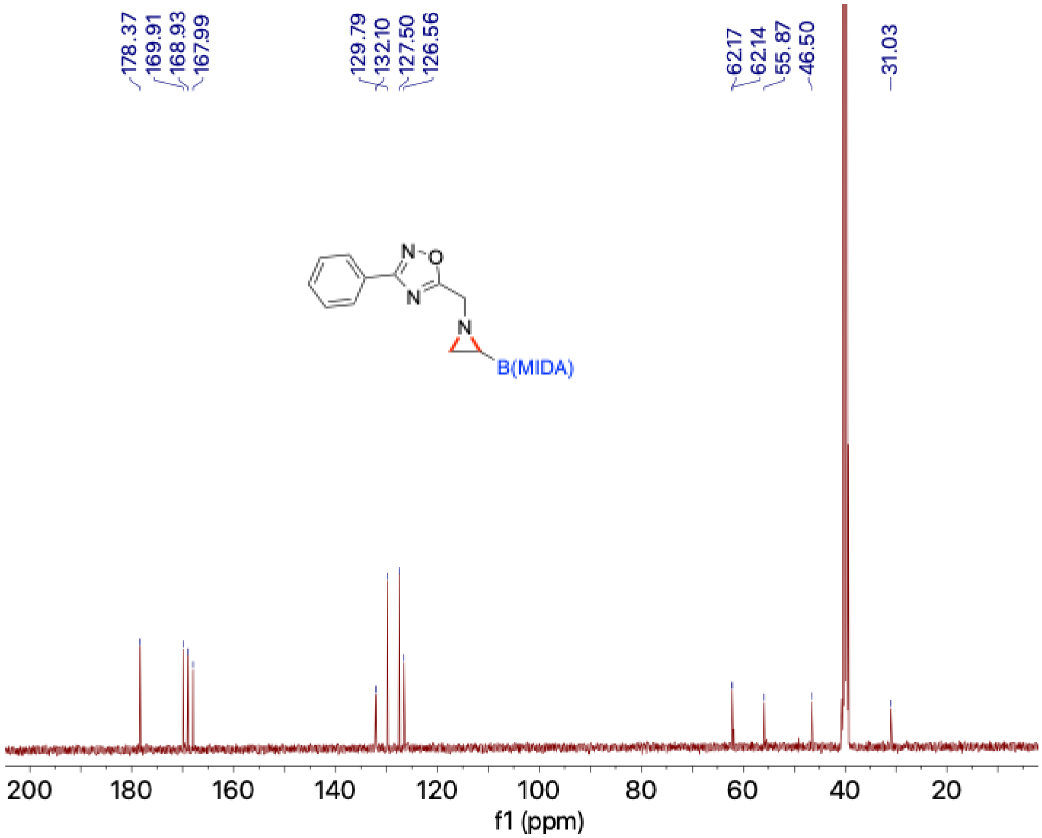
178.37
169.91
168.93
167.99
129.79
132.10
127.50
126.56
62.17
62.14
55.87
46.50
31.03
N
O
N
N
B(MIDA)
200
180
160
140
120
100
80
60
40
20
f1 (ppm)

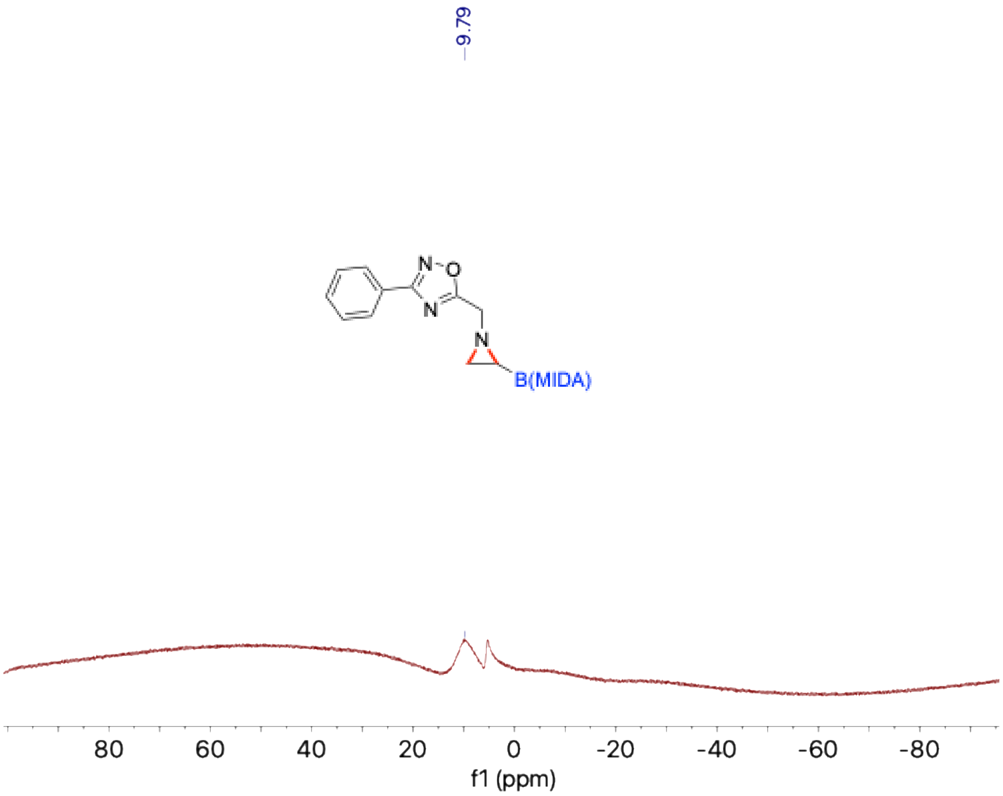
9.79
N
O
N
N
B(MIDA)
80
60
40
20
0
-20
-40
-60
-80
f1 (ppm)

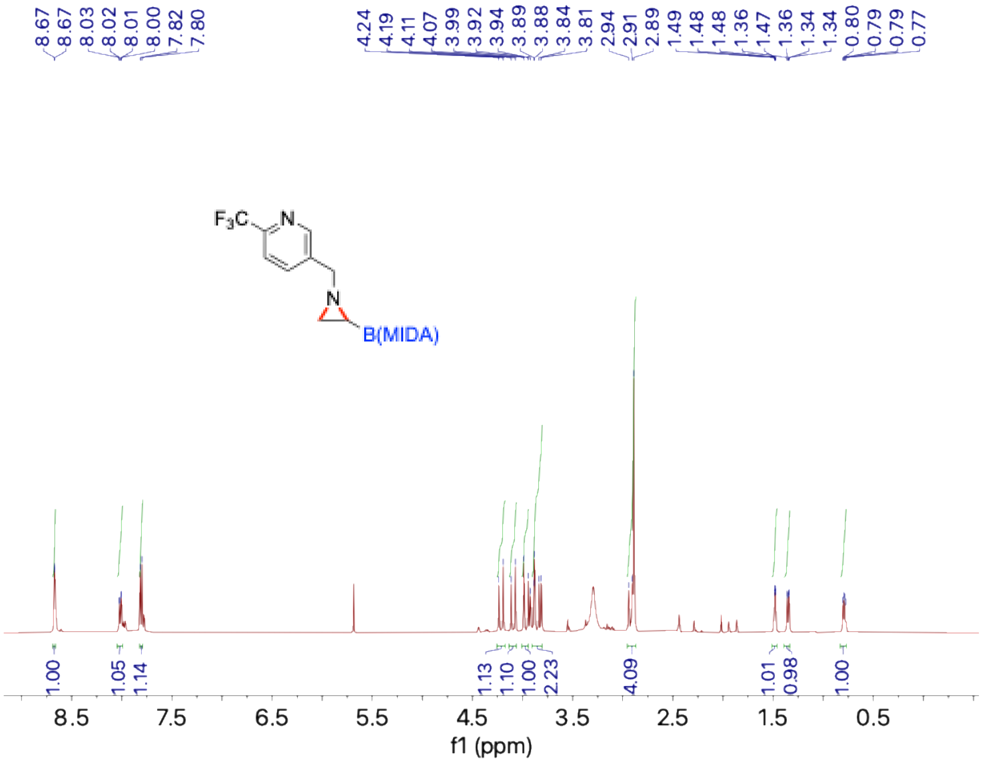

8.67 8.67 8.03 8.02 8.01 8.00 7.82 7.80
4.24 4.19 4.11 4.07 3.99 3.92 3.94 3.89 3.88 3.84 3.81 2.94 2.91 2.89 1.49 1.48 1.48 1.36 1.47 1.36 1.34 1.34 0.80 0.79 0.79 0.77
F3C
N
N
B(MIDA)
1.00
1.05
1.14
1.13
1.10
1.00
2.23
4.09
1.01
0.98
1.00
8.5
7.5
6.5
5.5
4.5
3.5
2.5
1.5
0.5
f1 (ppm)

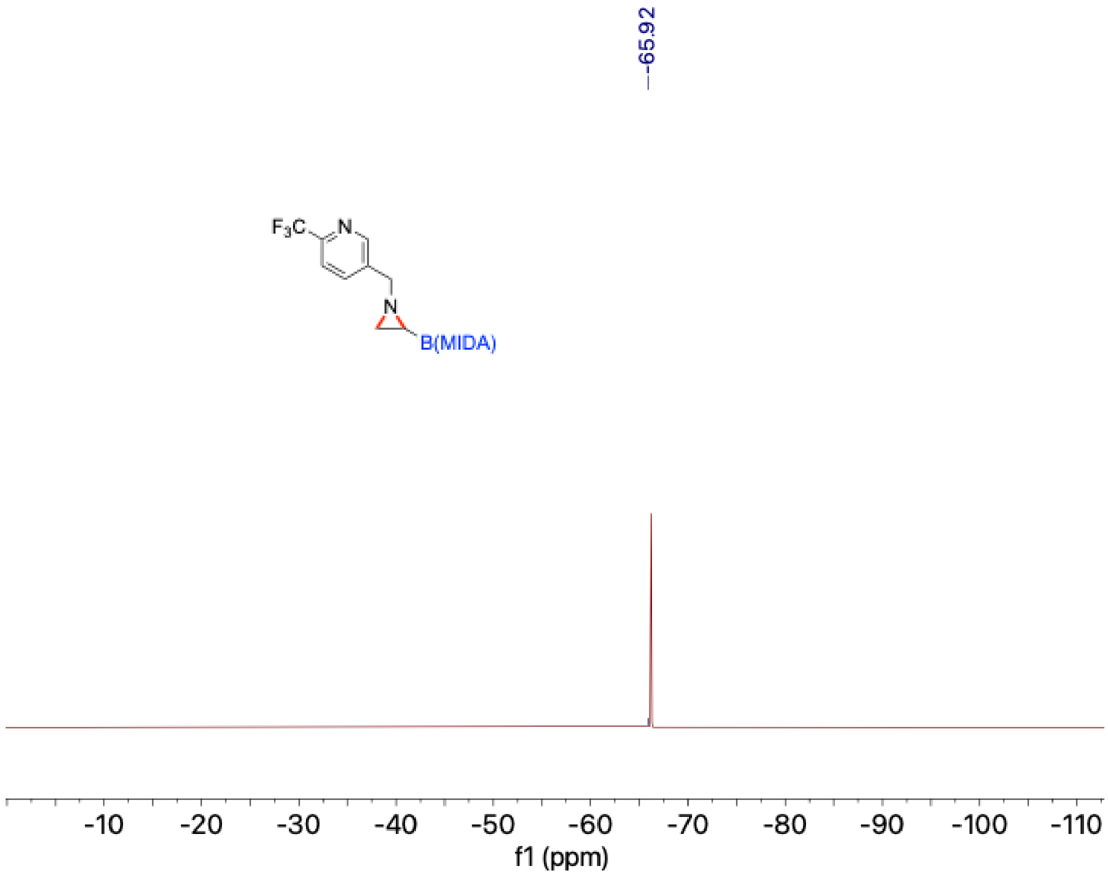

-65.92
F3C
N
N
B(MIDA)
-10
-20
-30
-40
-50
-60
-70
-80
-90
-100
-110
f1 (ppm)

VB-F-159-2-CF3-PY-CH2-NH2.12.fid

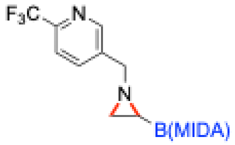

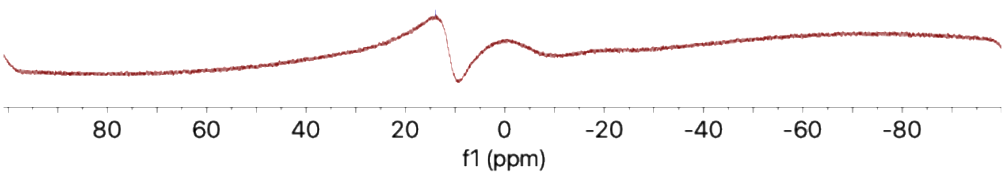

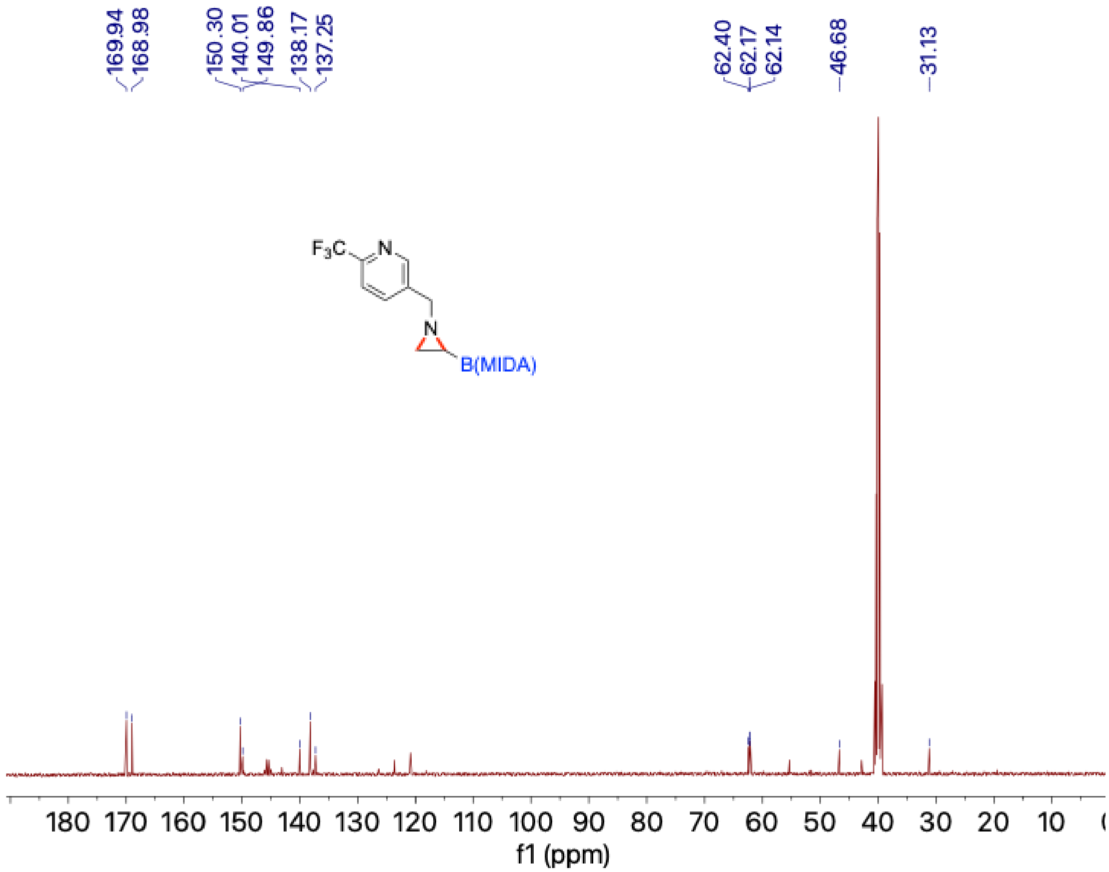

169.94
168.98
150.30
140.01
149.86
138.17
137.25
62.40
62.17
62.14
46.68
31.13
F3C
N
N
B(MIDA)
180 170 160 150 140 130 120 110 100 90 80 70 60 50 40 30 20 10
f1 (ppm)

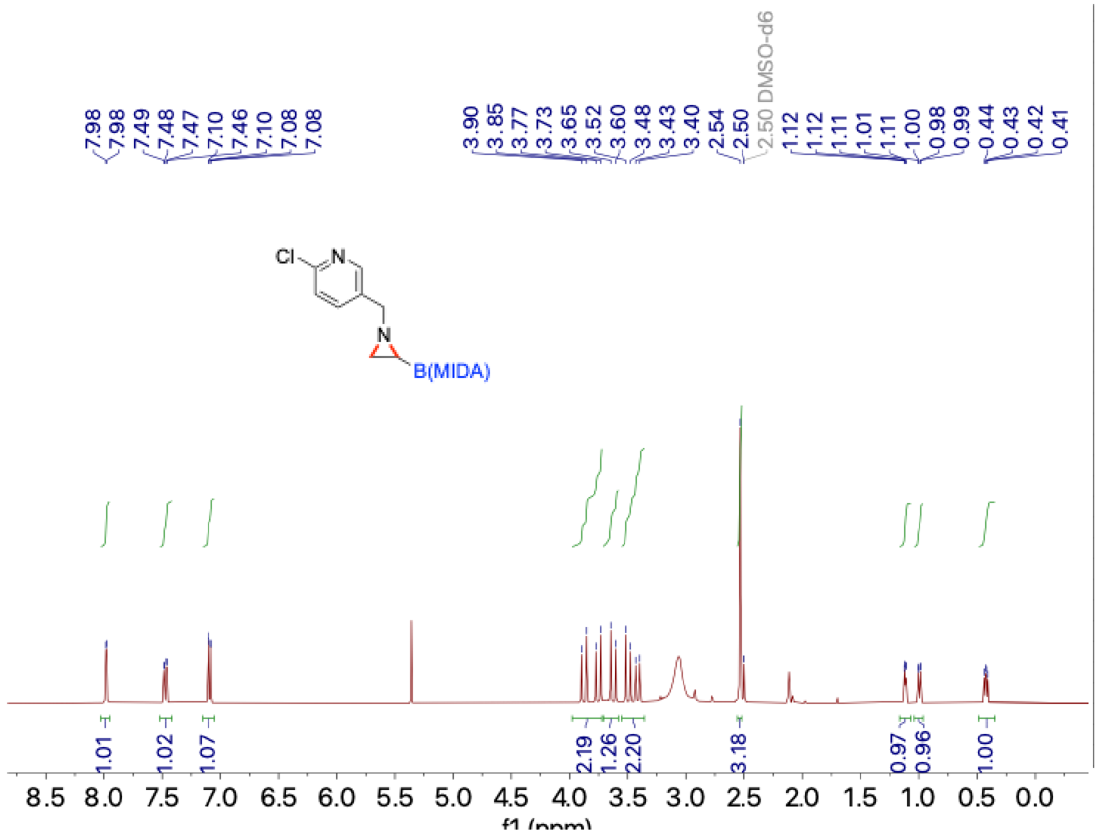

7.98
7.98
7.49
7.48
7.47
7.10
7.46
7.10
7.08
7.08
3.90
3.85
3.77
3.73
3.65
3.52
3.60
3.48
3.43
3.40
2.54
2.50
2.50 DMSO-d6
1.12
1.12
1.11
1.01
1.11
1.00
0.98
0.99
0.44
0.43
0.42
0.41
Cl
N
N
B(MIDA)
1.01
1.02
1.07
2.19
1.26
2.20
3.18
0.97
0.96
1.00
8.5 8.0 7.5 7.0 6.5 6.0 5.5 5.0 4.5 4.0 3.5 3.0 2.5 2.0 1.5 1.0 0.5 0.0
f1 (ppm)

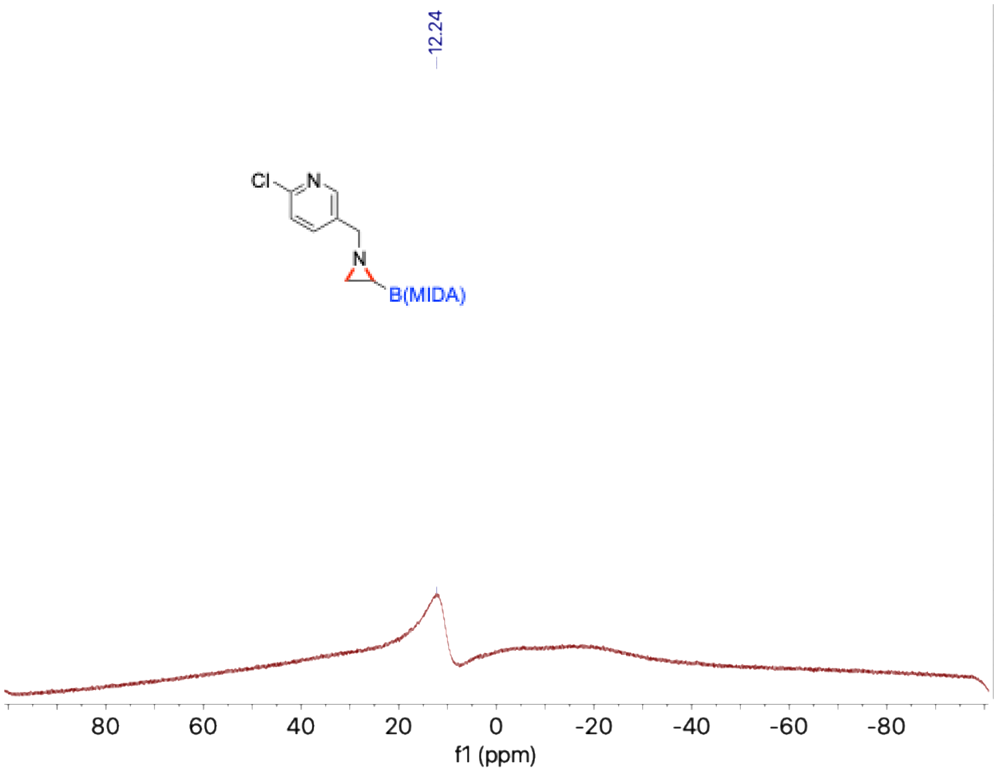

12.24
Cl
N
N
B(MIDA)
80 60 40 20 0 -20 -40 -60 -80
f1 (ppm)

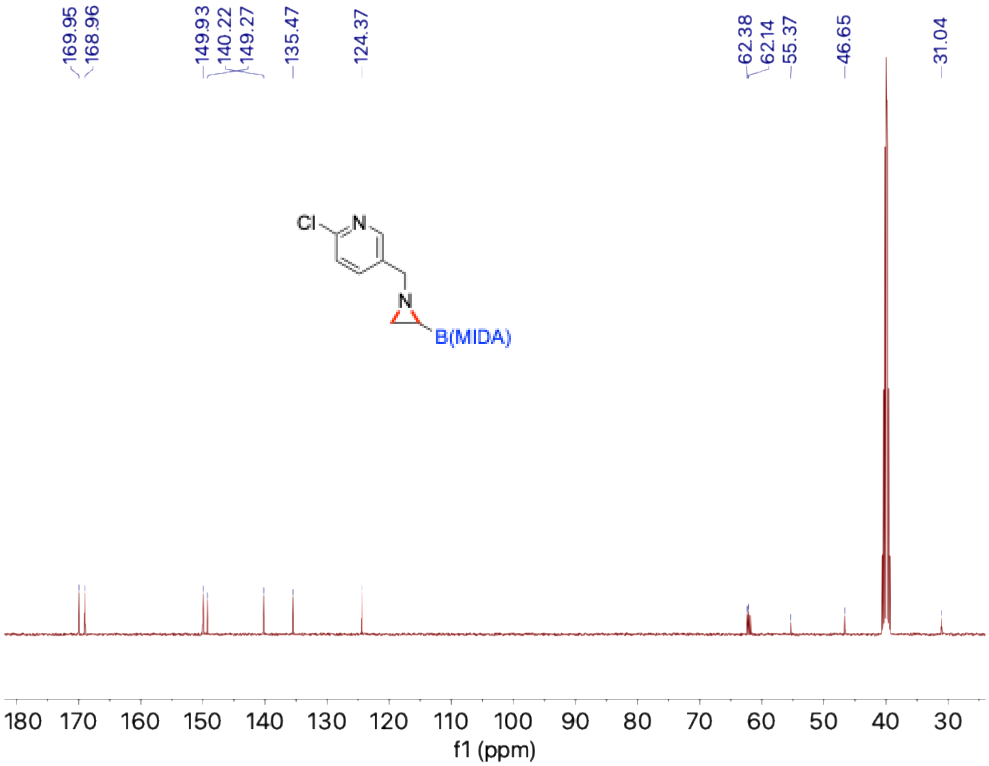

169.95
168.96
149.93
140.22
149.27
135.47
124.37
62.38
62.14
55.37
46.65
31.04
Cl
N
N
B(MIDA)
180 170 160 150 140 130 120 110 100 90 80 70 60 50 40 30
f1 (ppm)

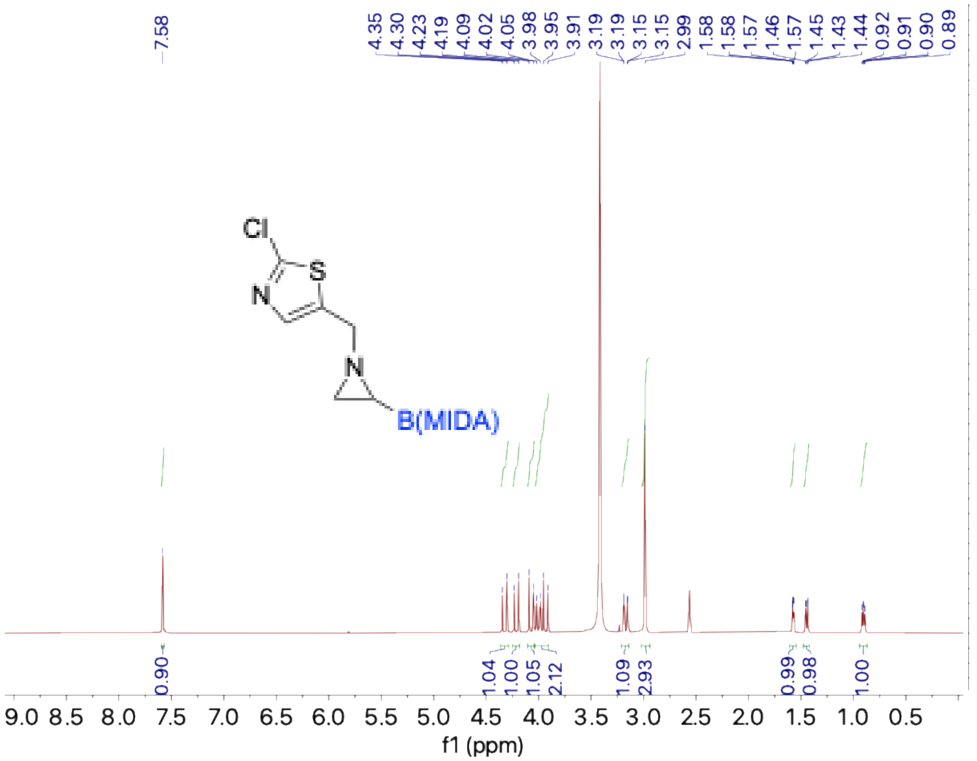

7.58
4.35
4.30
4.23
4.19
4.09
4.02
4.05
3.98
3.95
3.91
3.19
3.19
3.15
3.15
2.99
1.58
1.58
1.57
1.46
1.57
1.45
1.43
1.44
0.92
0.91
0.90
0.89
Cl
S
N
N
B(MIDA)
0.90
1.04
1.00
1.05
2.12
1.09
2.93
0.99
0.98
1.00
9.0 8.5 8.0 7.5 7.0 6.5 6.0 5.5 5.0 4.5 4.0 3.5 3.0 2.5 2.0 1.5 1.0 0.5
f1 (ppm)

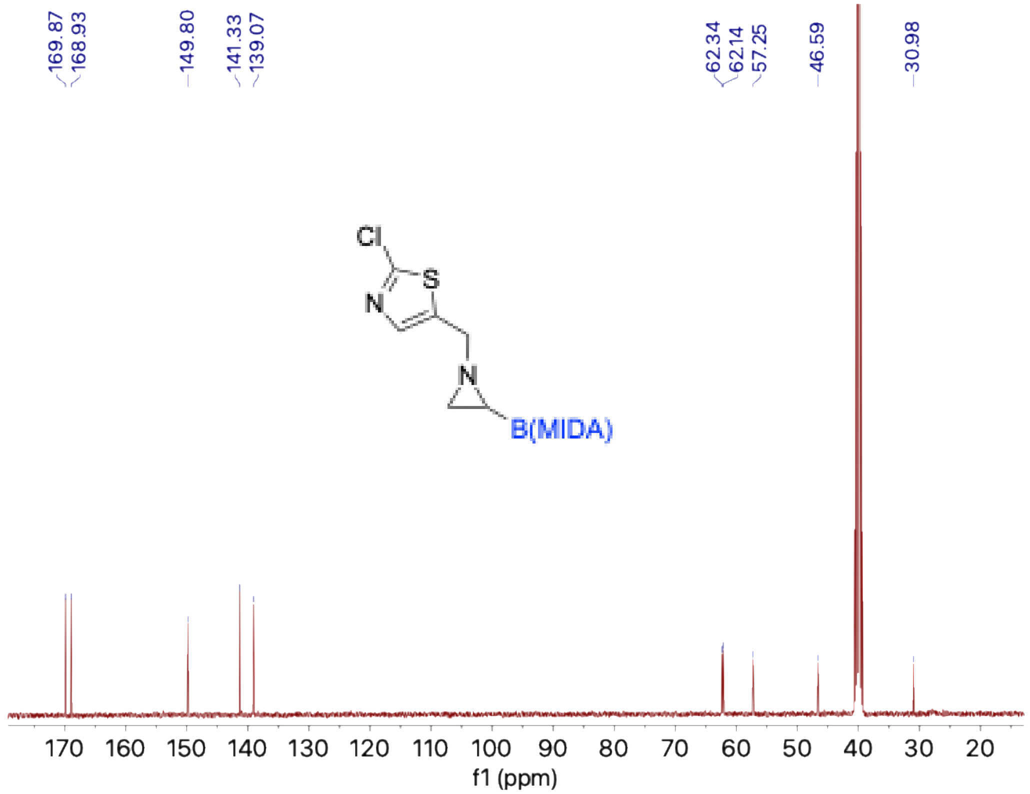
169.87
168.93
149.80
141.33
139.07
62.34
62.14
57.25
46.59
30.98
Cl
S
N
N
B(MIDA)
170 160 150 140 130 120 110 100 90 80 70 60 50 40 30 20
f1 (ppm)

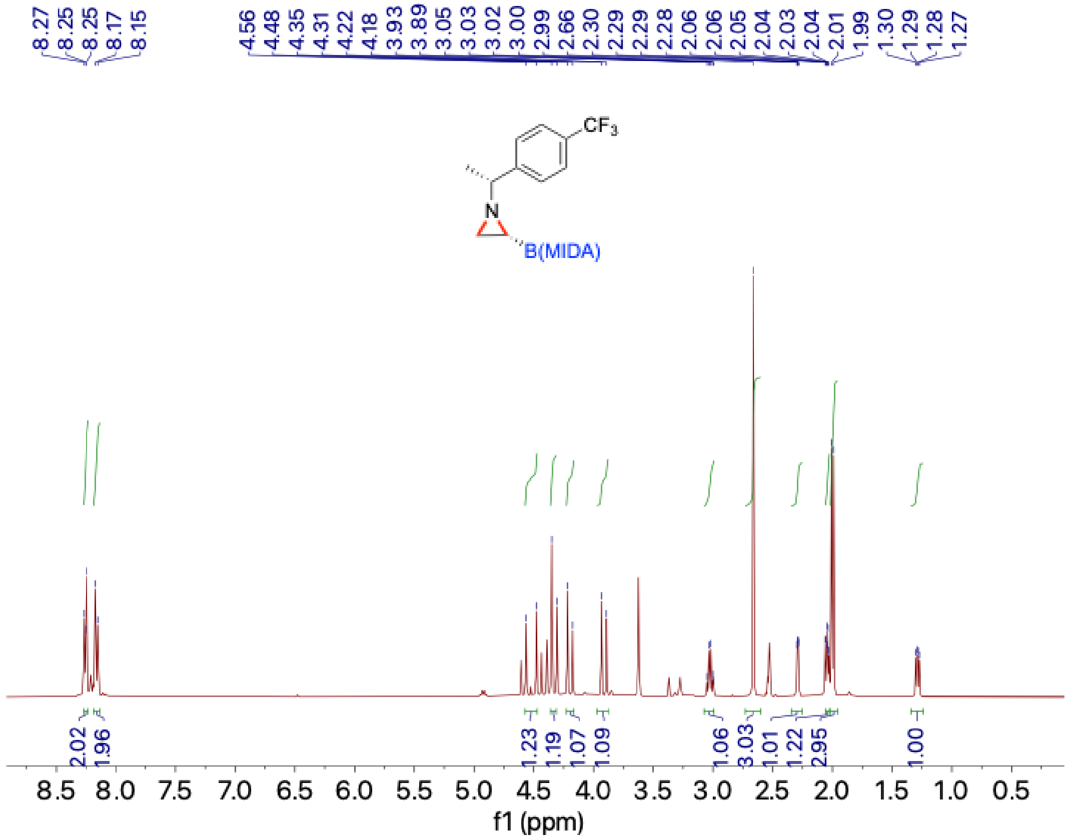
8.27
8.25
8.25
8.17
8.15
4.56
4.48
4.35
4.31
4.22
4.18
3.93
3.89
3.05
3.03
3.02
3.00
2.99
2.66
2.30
2.29
2.29
2.28
2.06
2.06
2.05
2.04
2.03
2.04
2.01
1.99
1.30
1.29
1.28
1.27
CF3
N
B(MIDA)
2.02
1.96
1.23
1.19
1.07
1.09
1.06
3.03
1.01
1.22
2.95
1.00
8.5 8.0 7.5 7.0 6.5 6.0 5.5 5.0 4.5 4.0 3.5 3.0 2.5 2.0 1.5 1.0 0.5
f1 (ppm)

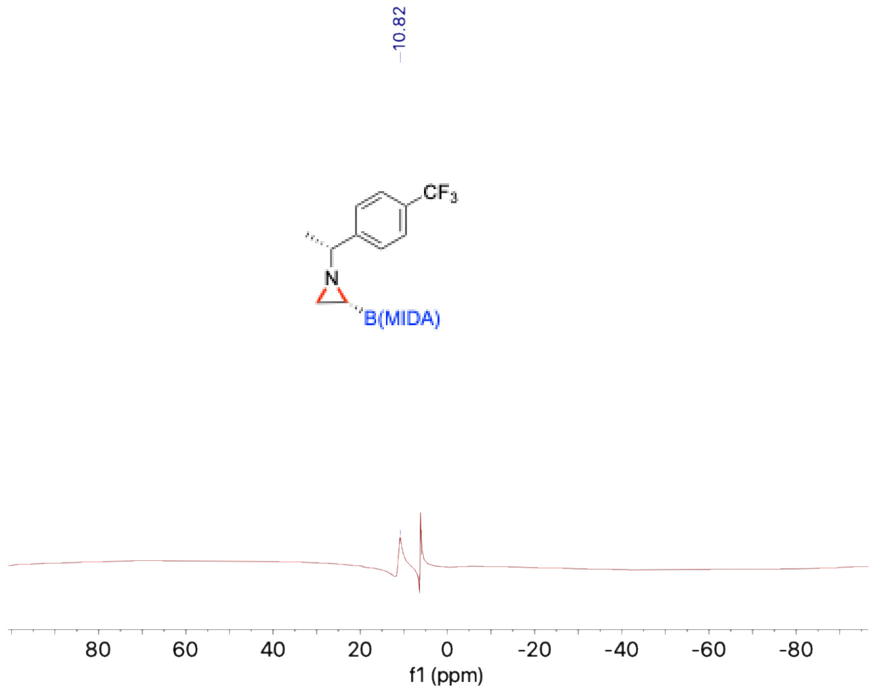
10.82
CF3
N
B(MIDA)
80
60
40
20
0
-20
-40
-60
-80
f1 (ppm)

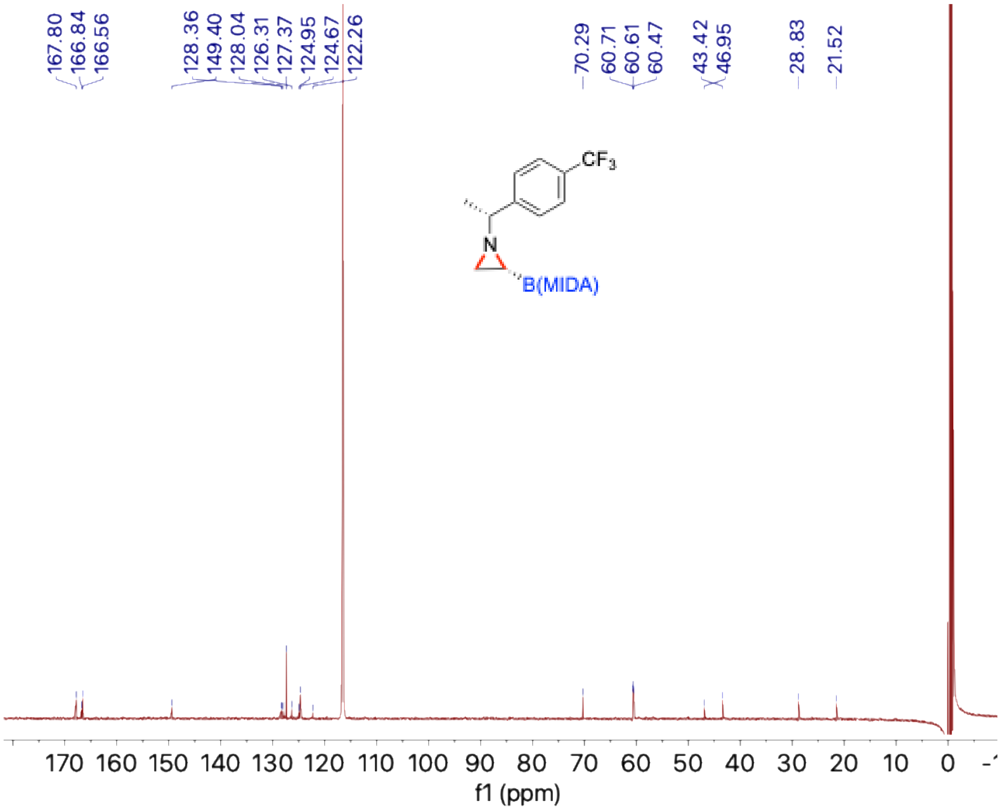
167.80
166.84
166.56
128.36
149.40
128.04
126.31
127.37
124.95
124.67
122.26
70.29
60.71
60.61
60.47
43.42
46.95
28.83
21.52
CF3
N
B(MIDA)
170
160
150
140
130
120
110
100
90
80
70
60
50
40
30
20
10
0
f1 (ppm)

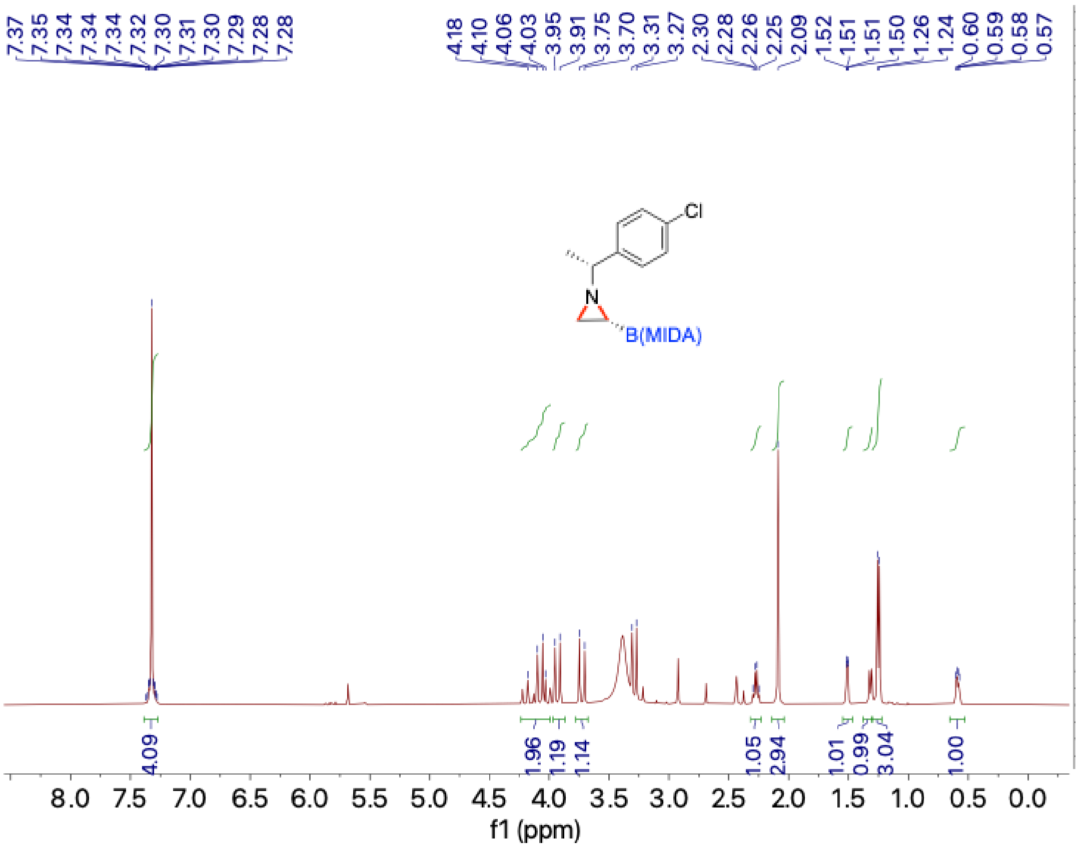

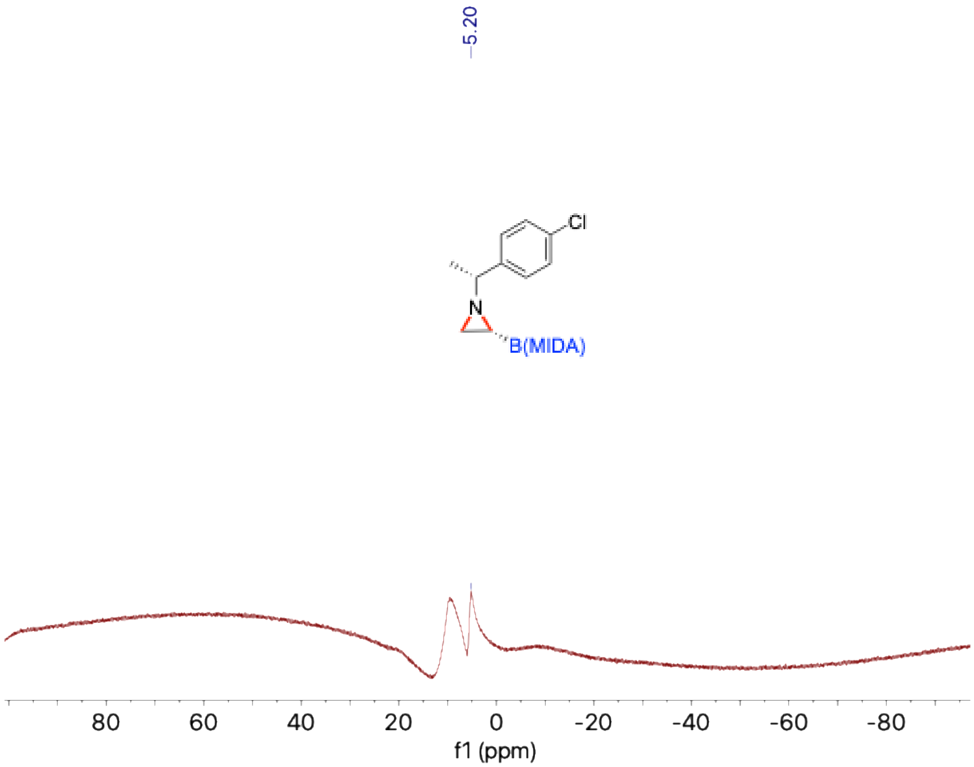

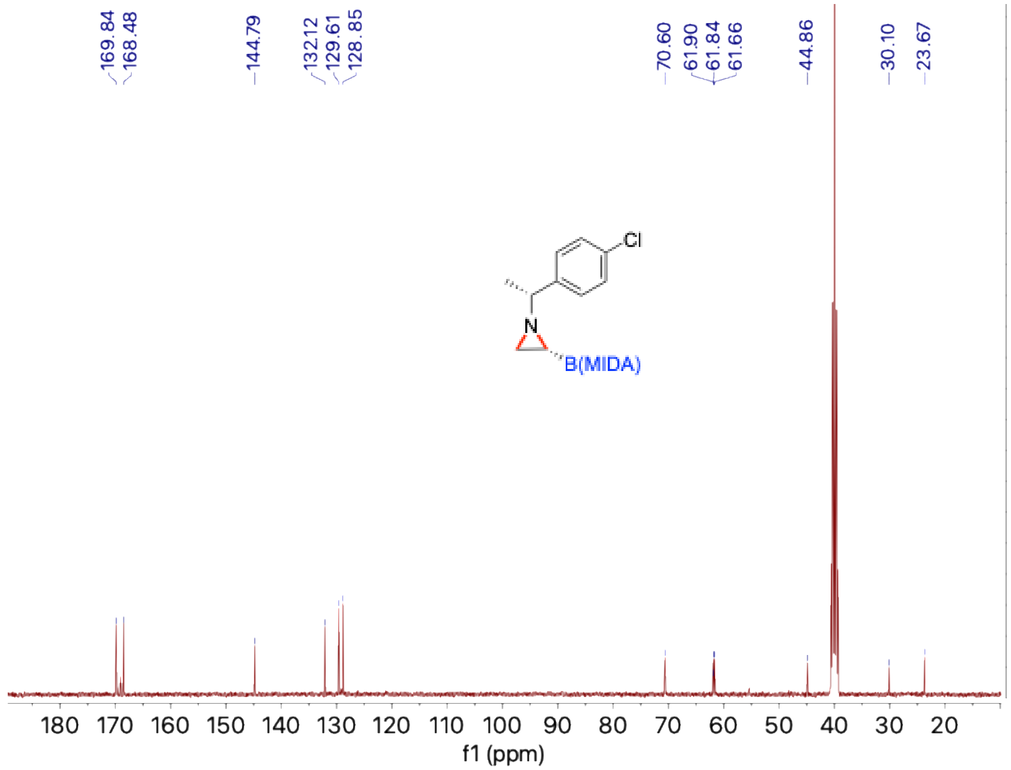
169.84
168.48
144.79
132.12
129.61
128.85
70.60
61.90
61.84
61.66
44.86
30.10
23.67
Cl
N
B(MIDA)
180 170 160 150 140 130 120 110 100 90 80 70 60 50 40 30 20
f1 (ppm)

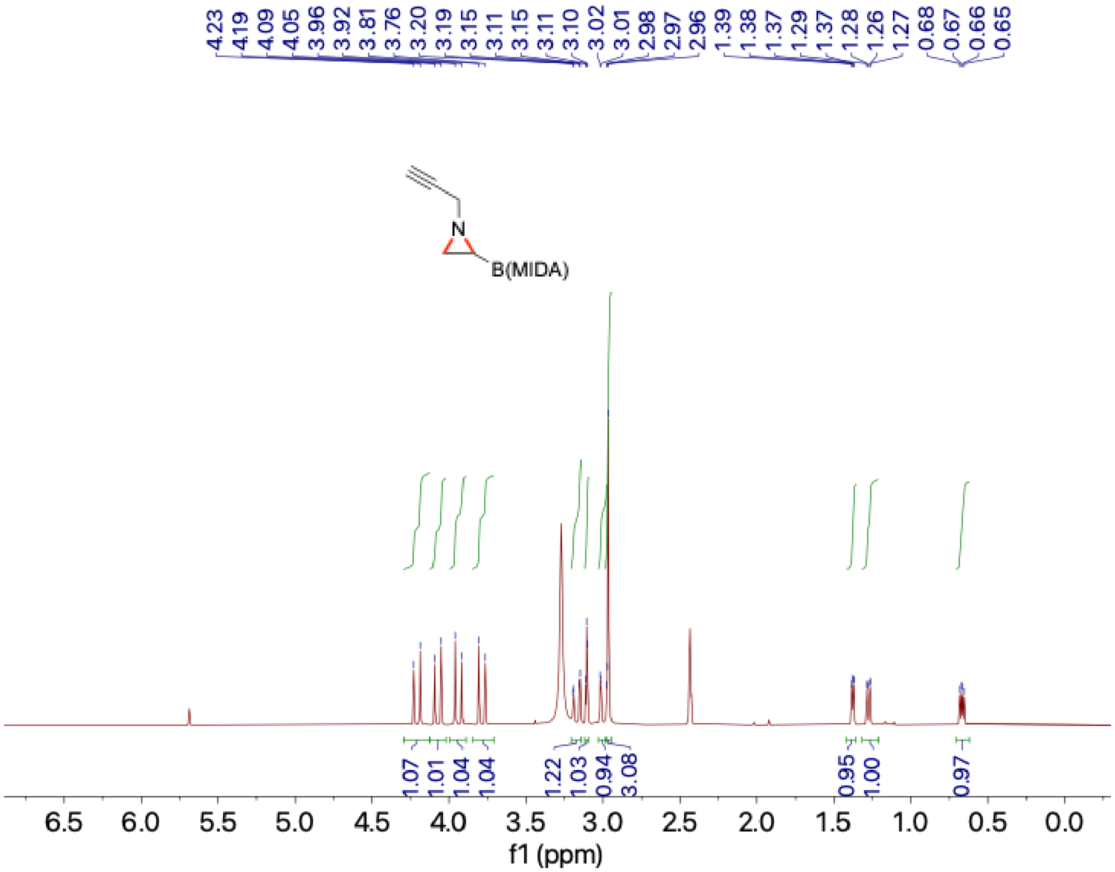
4.23
4.19
4.09
4.05
3.96
3.92
3.81
3.76
3.20
3.19
3.15
3.11
3.15
3.11
3.10
3.02
3.01
2.98
2.97
2.96
1.39
1.38
1.37
1.29
1.37
1.28
1.26
1.27
0.68
0.67
0.66
0.65
N
B(MIDA)
1.07
1.01
1.04
1.04
1.22
1.03
0.94
3.08
0.95
1.00
0.97
6.5 6.0 5.5 5.0 4.5 4.0 3.5 3.0 2.5 2.0 1.5 1.0 0.5 0.0
f1 (ppm)

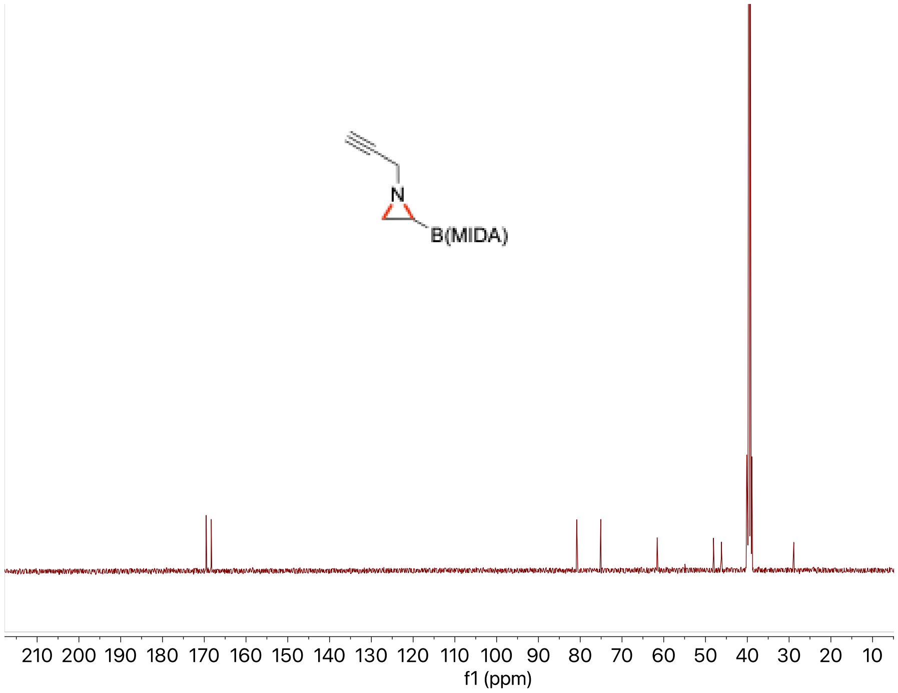
N
B(MIDA)
210 200 190 180 170 160 150 140 130 120 110 100 90 80 70 60 50 40 30 20 10
f1 (ppm)

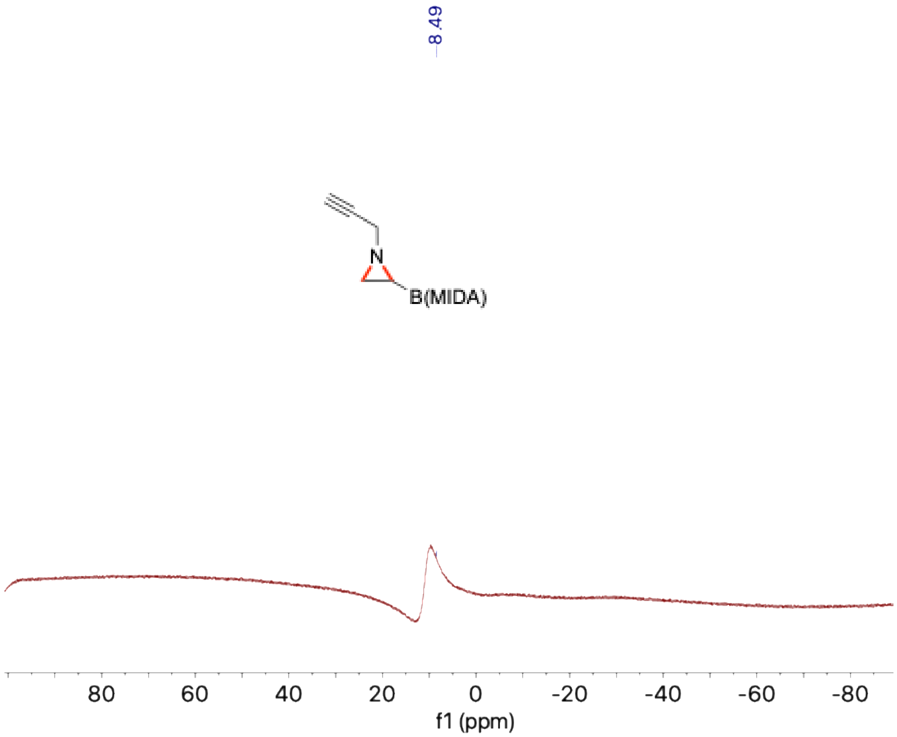

8.49
N
B(MIDA)
80
60
40
20
0
-20
-40
-60
-80
f1 (ppm)

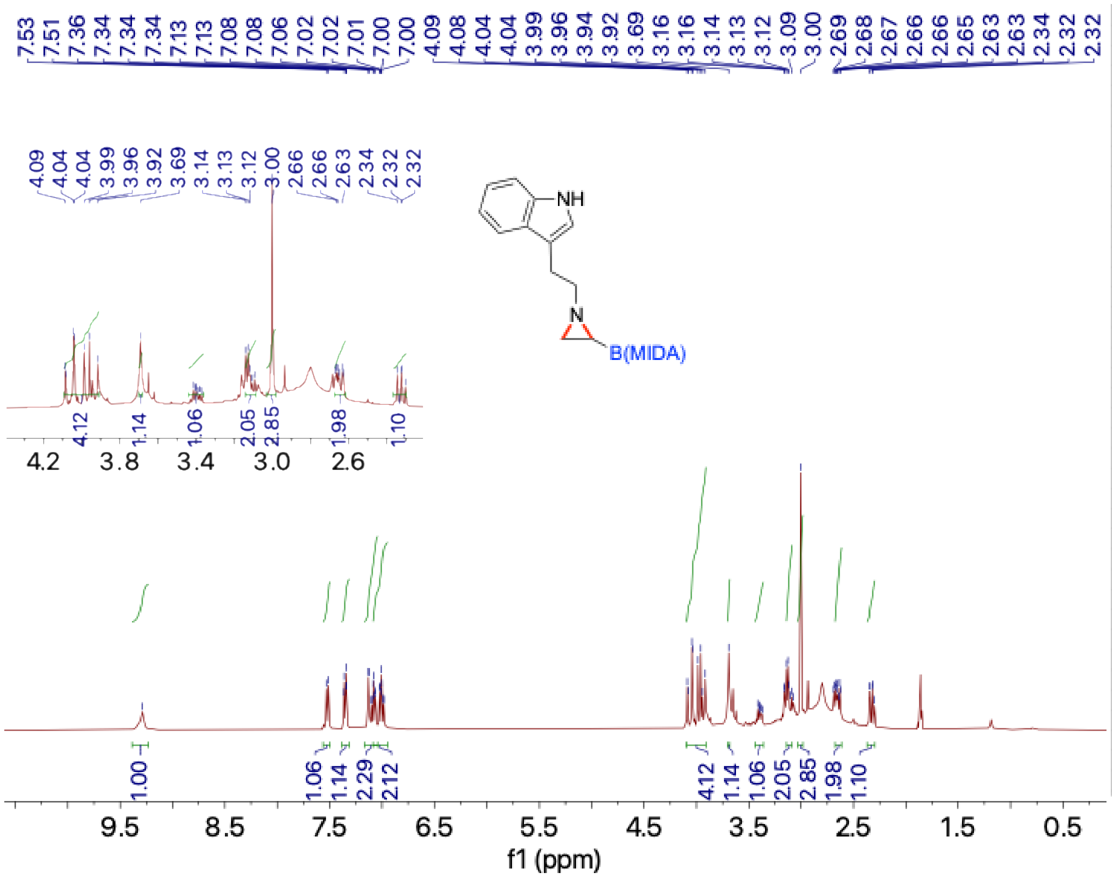
NH
N
B(MIDA)
f1 (ppm)

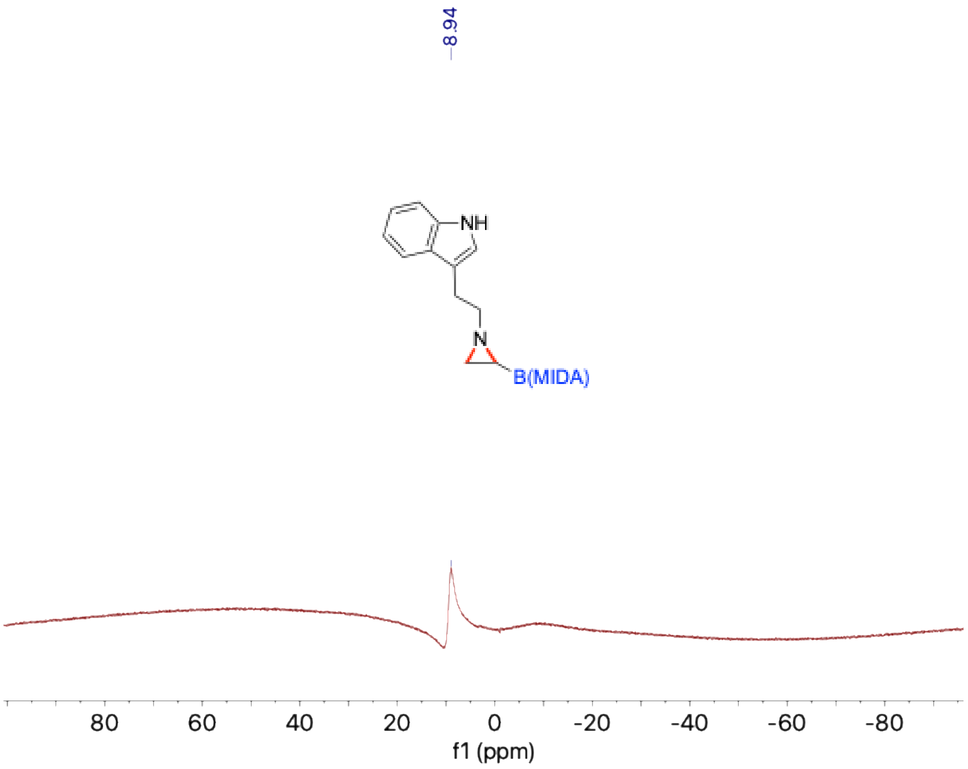
-8.94
NH
N
B(MIDA)
f1 (ppm)

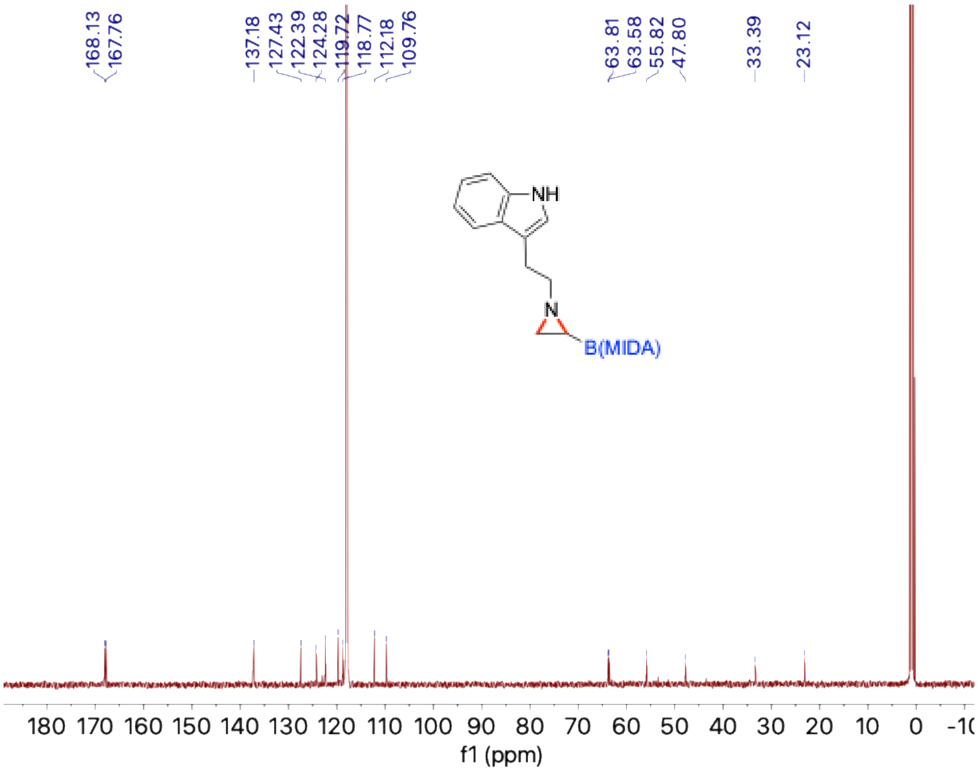
168.13
167.76
137.18
127.43
122.39
124.28
119.72
118.77
112.18
109.76
63.81
63.58
55.82
47.80
33.39
23.12
NH
N
B(MIDA)
180 170 160 150 140 130 120 110 100 90 80 70 60 50 40 30 20 10 0 -10
f1 (ppm)

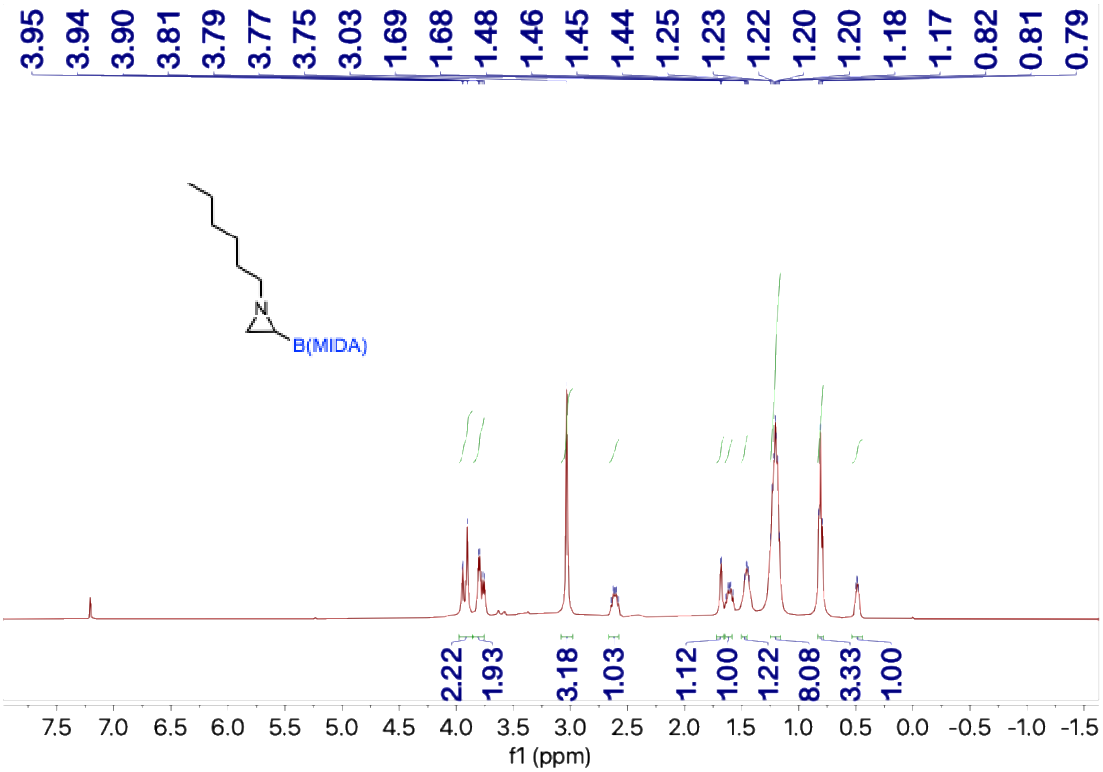
3.95
3.94
3.90
3.81
3.79
3.77
3.75
3.03
1.69
1.68
1.48
1.46
1.45
1.44
1.25
1.23
1.22
1.20
1.20
1.18
1.17
0.82
0.81
0.79
N
B(MIDA)
2.22
1.93
3.18
1.03
1.12
1.00
1.22
8.08
3.33
1.00
7.5 7.0 6.5 6.0 5.5 5.0 4.5 4.0 3.5 3.0 2.5 2.0 1.5 1.0 0.5 0.0 -0.5 -1.0 -1.5
f1 (ppm)

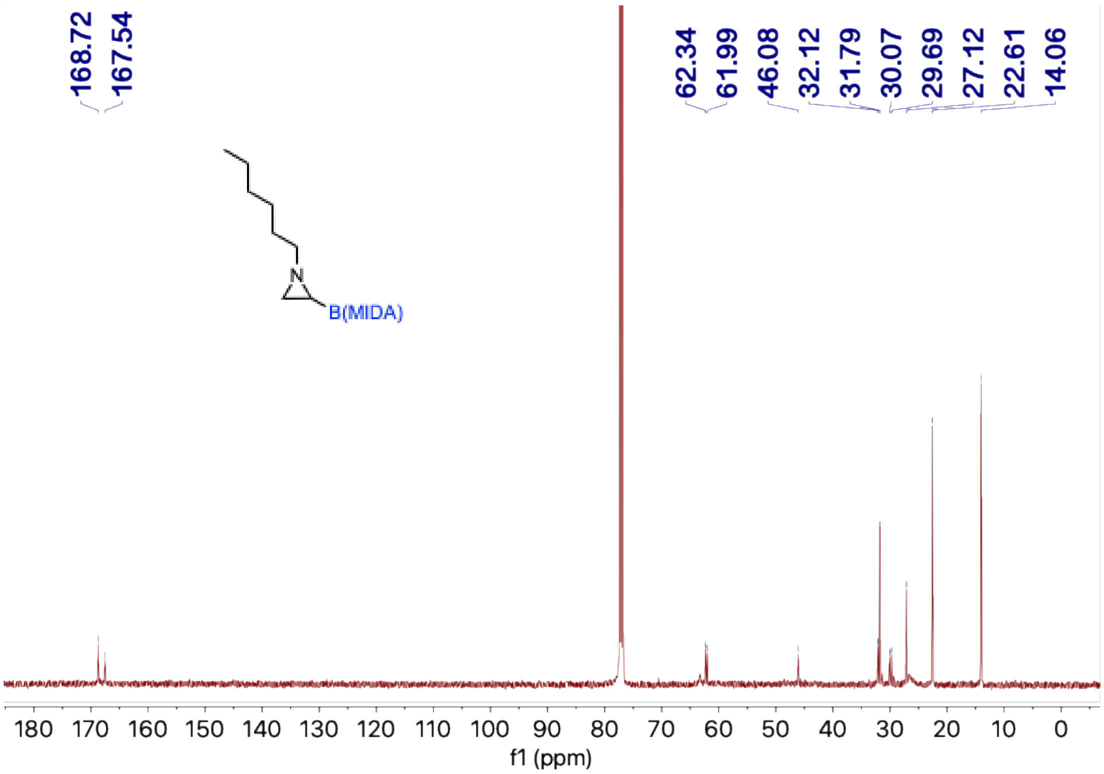
168.72
167.54
62.34
61.99
46.08
32.12
31.79
30.07
29.69
27.12
22.61
14.06
N
B(MIDA)
180 170 160 150 140 130 120 110 100 90 80 70 60 50 40 30 20 10 0
f1 (ppm)

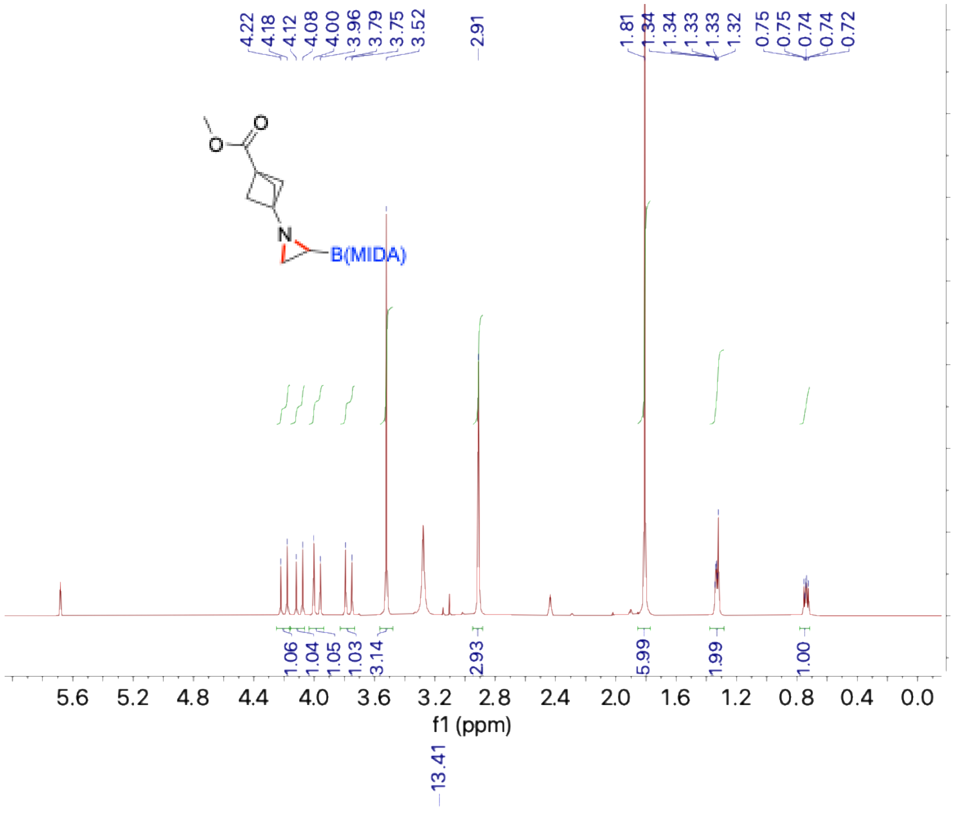
B(MIDA)
N
O
O
4.22
4.18
4.12
4.08
4.00
3.96
3.79
3.75
3.52
2.91
1.81
1.34
1.34
1.33
1.33
1.32
0.75
0.75
0.74
0.74
0.72
1.06
1.04
1.05
1.03
3.14
2.93
5.99
1.99
1.00
f1 (ppm)

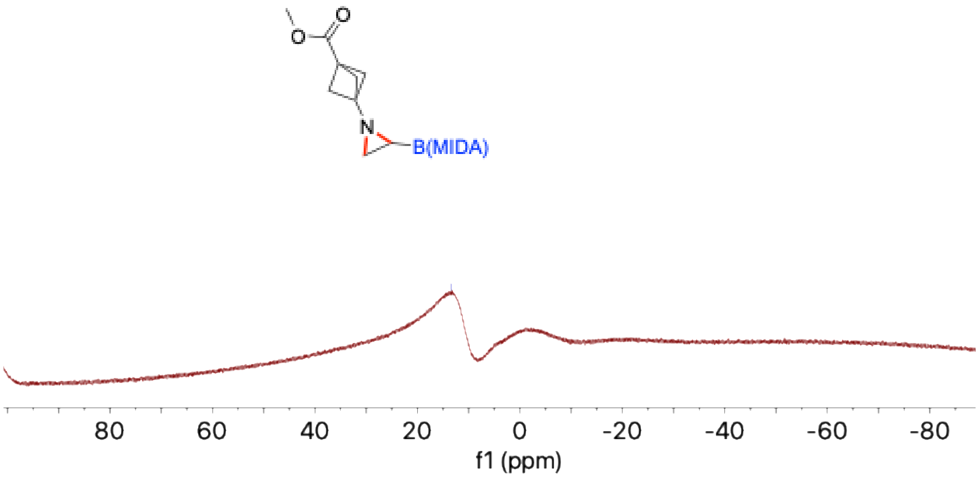
13.41
B(MIDA)
N
O
O
f1 (ppm)

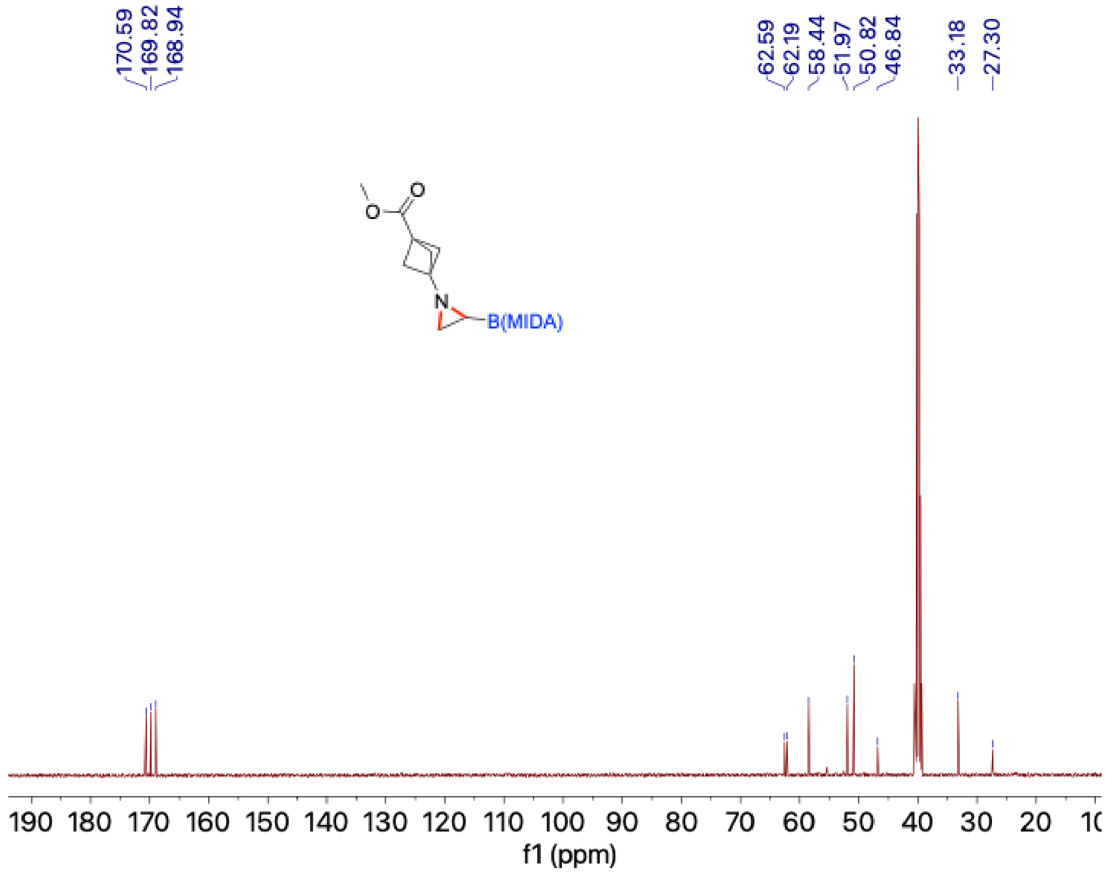

170.59
169.82
168.94
62.59
62.19
58.44
51.97
50.82
46.84
33.18
27.30
N
O
O
B(MIDA)
190 180 170 160 150 140 130 120 110 100 90 80 70 60 50 40 30 20 10
f1 (ppm)

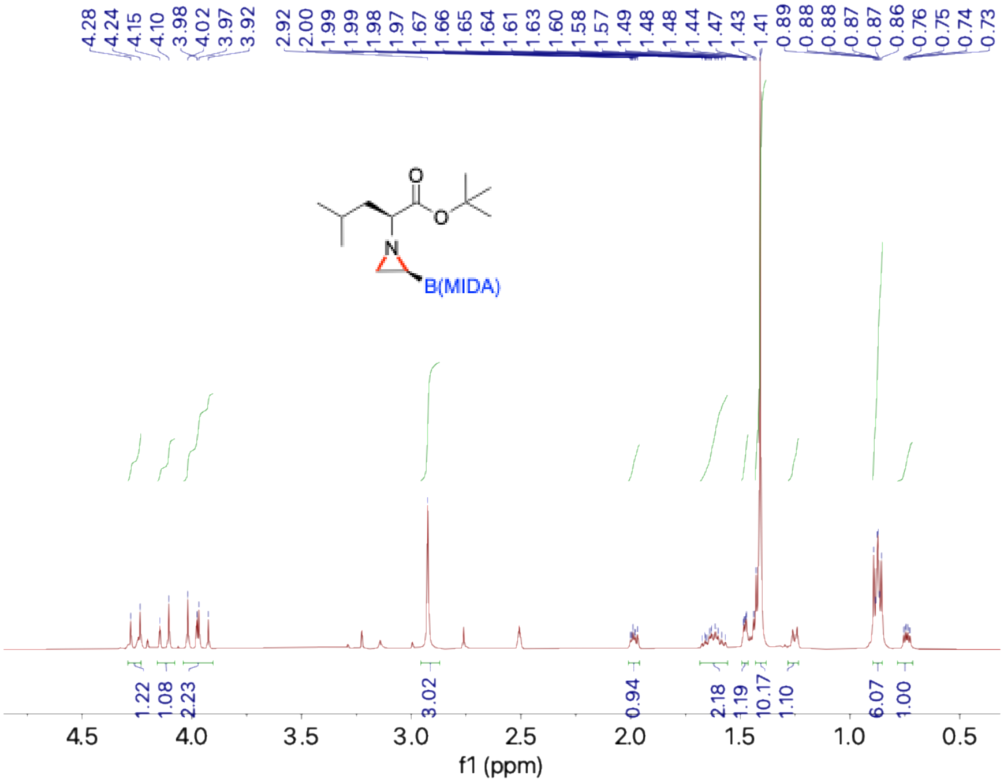
4.28 4.24 4.15 4.10 3.98 4.02 3.97 3.92 2.92 2.00 1.99 1.99 1.98 1.97 1.67 1.66 1.65 1.64 1.61 1.63 1.60 1.58 1.57 1.49 1.48 1.48 1.44 1.47 1.43 1.41 0.89 0.88 0.88 0.87 0.87 0.86 0.76 0.75 0.74 0.73
O
O
N
B(MIDA)
1.22 1.08 2.23 3.02 0.94 2.18 1.19 10.17 1.10 6.07 1.00
4.5 4.0 3.5 3.0 2.5 2.0 1.5 1.0 0.5
f1 (ppm)

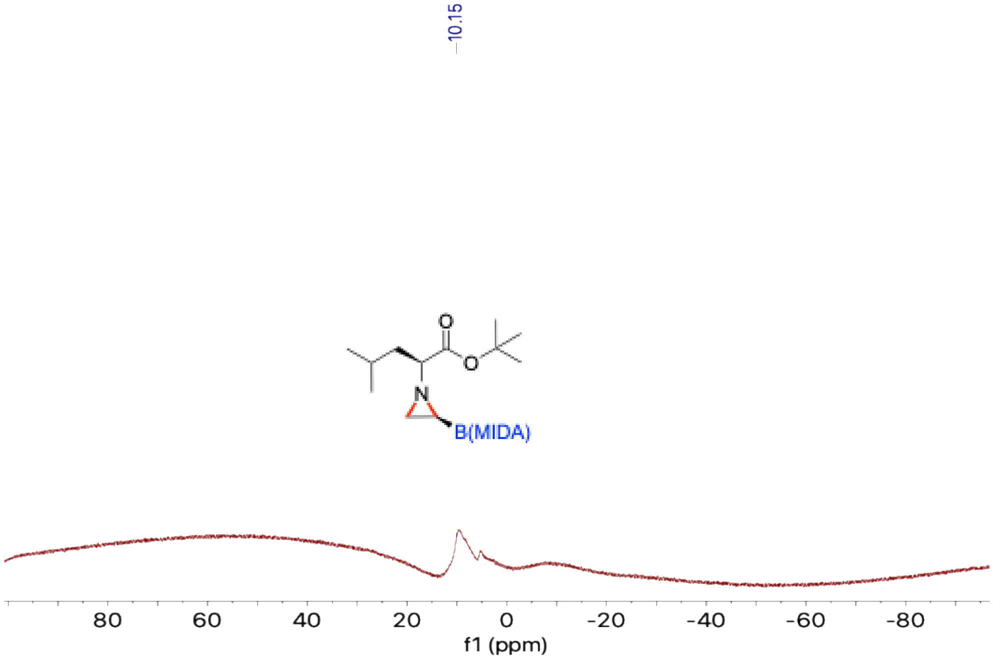
10.15
O
O
N
B(MIDA)
80 60 40 20 0 -20 -40 -60 -80
f1 (ppm)

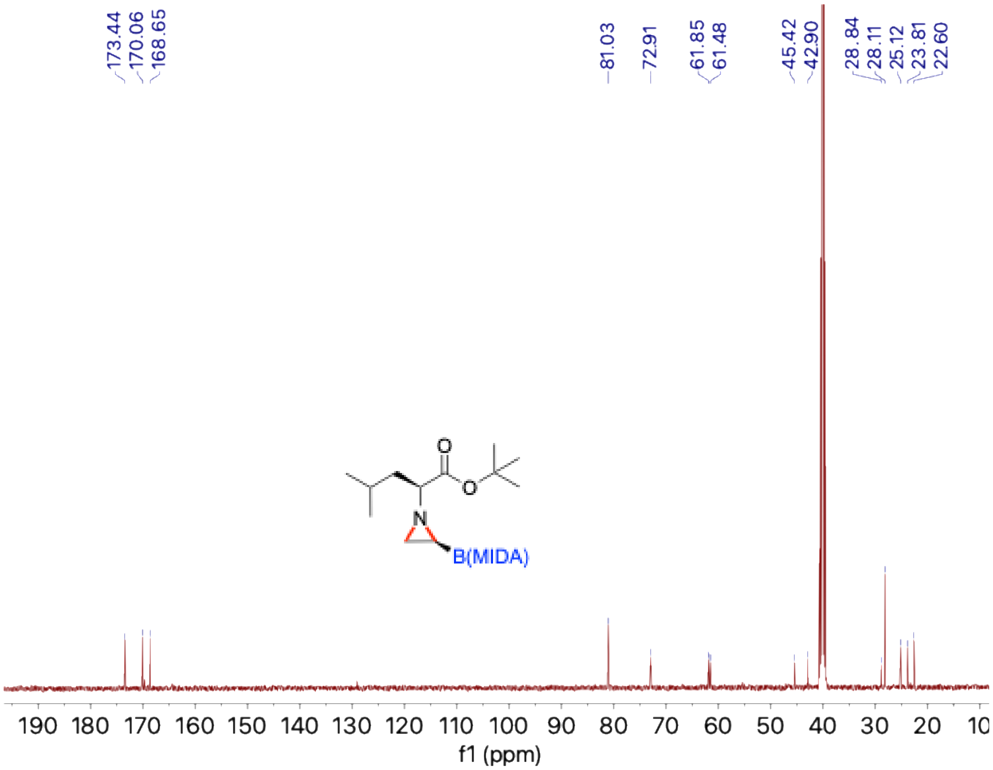
173.44
170.06
168.65
81.03
72.91
61.85
61.48
45.42
42.90
28.84
28.11
25.12
23.81
22.60
O
O
N
B(MIDA)
190 180 170 160 150 140 130 120 110 100 90 80 70 60 50 40 30 20 10
f1 (ppm)

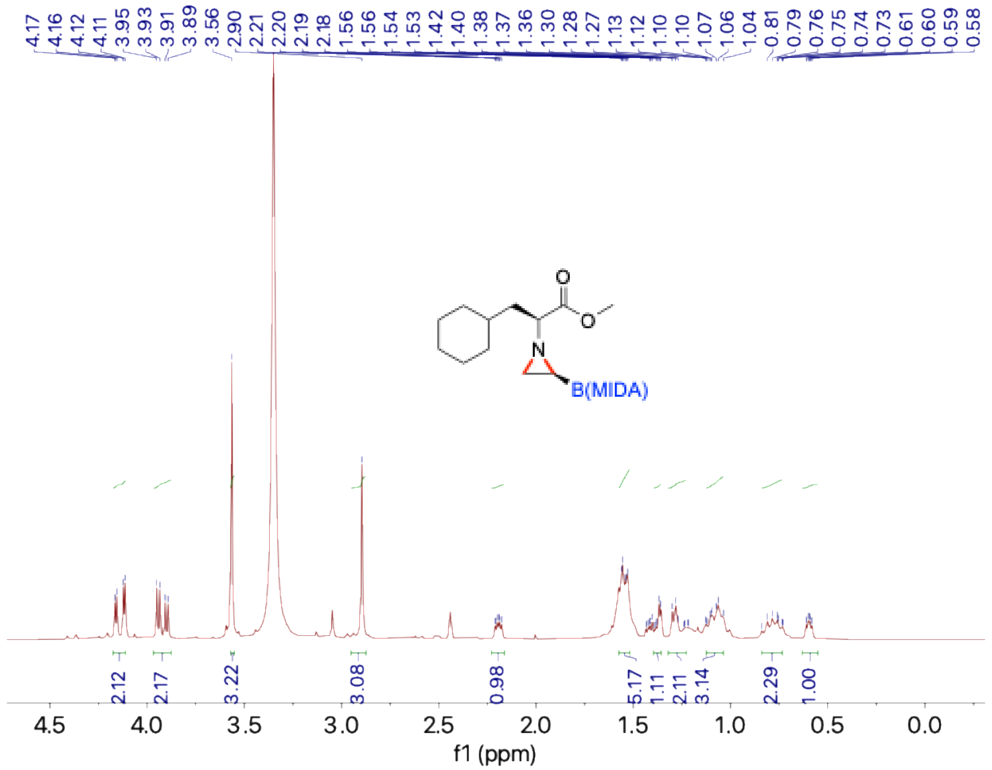
4.17
4.16
4.12
4.11
3.95
3.93
3.91
3.89
3.56
2.90
2.21
2.20
2.19
2.18
1.56
1.56
1.54
1.53
1.42
1.40
1.38
1.37
1.36
1.30
1.28
1.27
1.13
1.12
1.10
1.10
1.07
1.06
1.04
0.81
0.79
0.76
0.75
0.74
0.73
0.61
0.60
0.59
0.58
N
O
O
B(MIDA)
2.12
2.17
3.22
3.08
0.98
5.17
1.11
2.11
3.14
2.29
1.00
4.5
4.0
3.5
3.0
2.5
2.0
1.5
1.0
0.5
0.0
f1 (ppm)

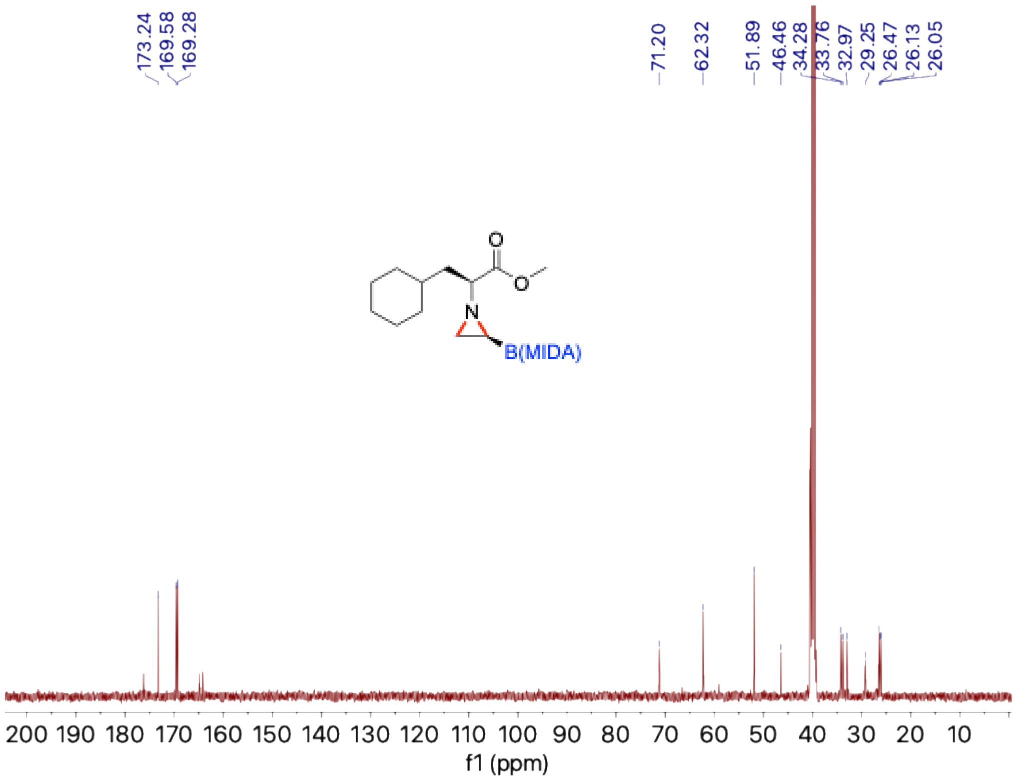
173.24
169.58
169.28
71.20
62.32
51.89
46.46
34.28
33.76
32.97
29.25
26.47
26.13
26.05
N
O
O
B(MIDA)
200 190 180 170 160 150 140 130 120 110 100 90 80 70 60 50 40 30 20 10
f1 (ppm)

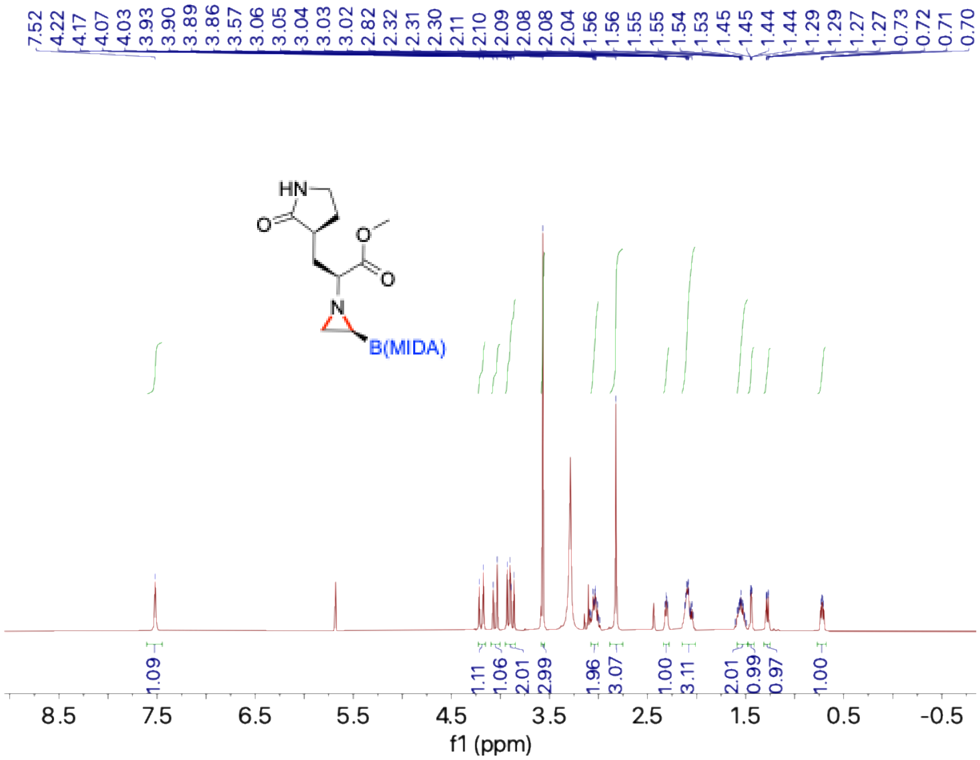
7.52 4.22 4.17 4.07 4.03 3.93 3.90 3.89 3.86 3.57 3.06 3.05 3.04 3.03 3.02 2.82 2.32 2.31 2.30 2.11 2.10 2.09 2.08 2.08 2.04 1.56 1.56 1.55 1.55 1.54 1.53 1.45 1.45 1.44 1.44 1.29 1.29 1.27 1.27 0.73 0.72 0.71 0.70
HN
O
O
O
N
B(MIDA)
1.09 1.11 1.06 2.01 2.99 1.96 3.07 1.00 3.11 2.01 0.99 0.97 1.00
8.5 7.5 6.5 5.5 4.5 3.5 2.5 1.5 0.5 -0.5
f1 (ppm)

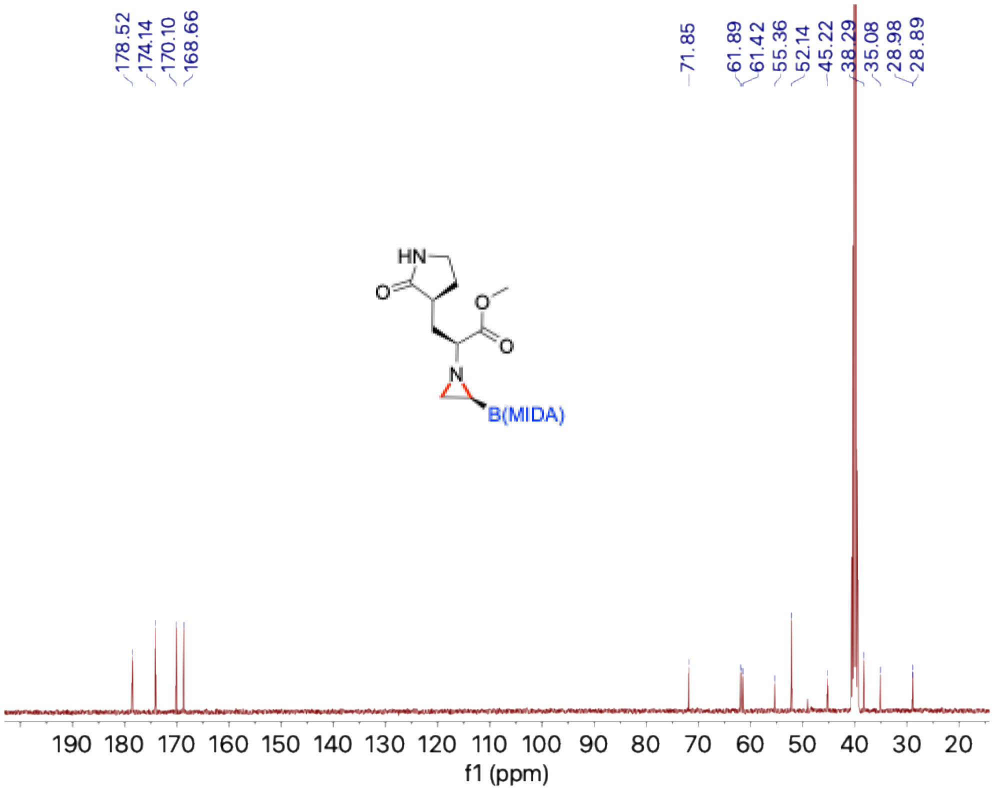
178.52 174.14 170.10 168.66
71.85
61.89 61.42 55.36 52.14 45.22 38.29 35.08 28.98 28.89
HN
O
O
O
N
B(MIDA)
190 180 170 160 150 140 130 120 110 100 90 80 70 60 50 40 30 20
f1 (ppm)

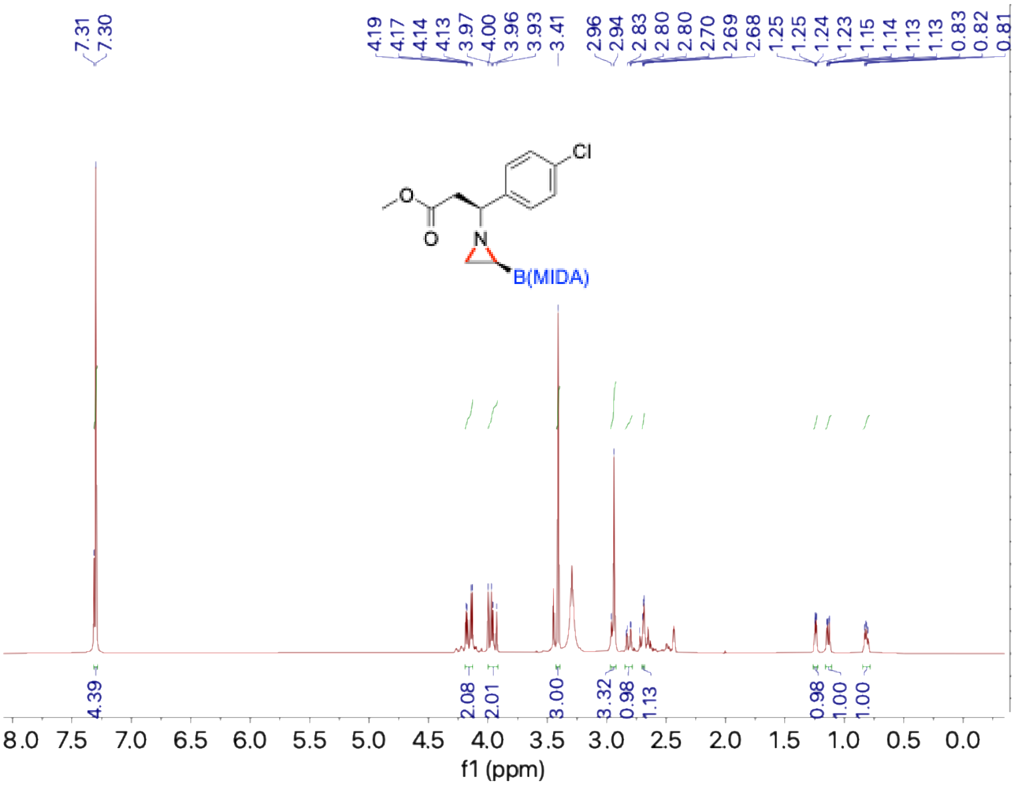

7.31
7.30
4.19
4.17
4.14
4.13
3.97
4.00
3.96
3.93
3.41
2.96
2.94
2.83
2.80
2.80
2.70
2.69
2.68
1.25
1.25
1.24
1.23
1.15
1.14
1.13
1.13
0.83
0.82
0.81
Cl
O
O
N
B(MIDA)
4.39
2.08
2.01
3.00
3.32
0.98
1.13
0.98
1.00
1.00
8.0 7.5 7.0 6.5 6.0 5.5 5.0 4.5 4.0 3.5 3.0 2.5 2.0 1.5 1.0 0.5 0.0
f1 (ppm)

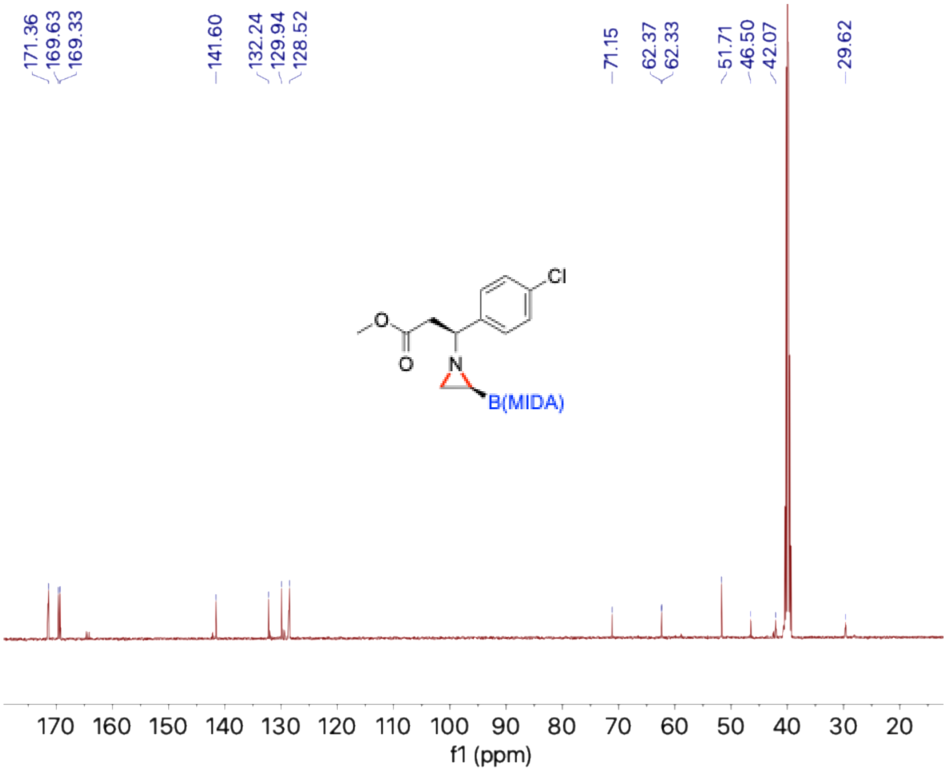

171.36
169.63
169.33
141.60
132.24
129.94
128.52
71.15
62.37
62.33
51.71
46.50
42.07
29.62
Cl
O
O
N
B(MIDA)
170 160 150 140 130 120 110 100 90 80 70 60 50 40 30 20
f1 (ppm)

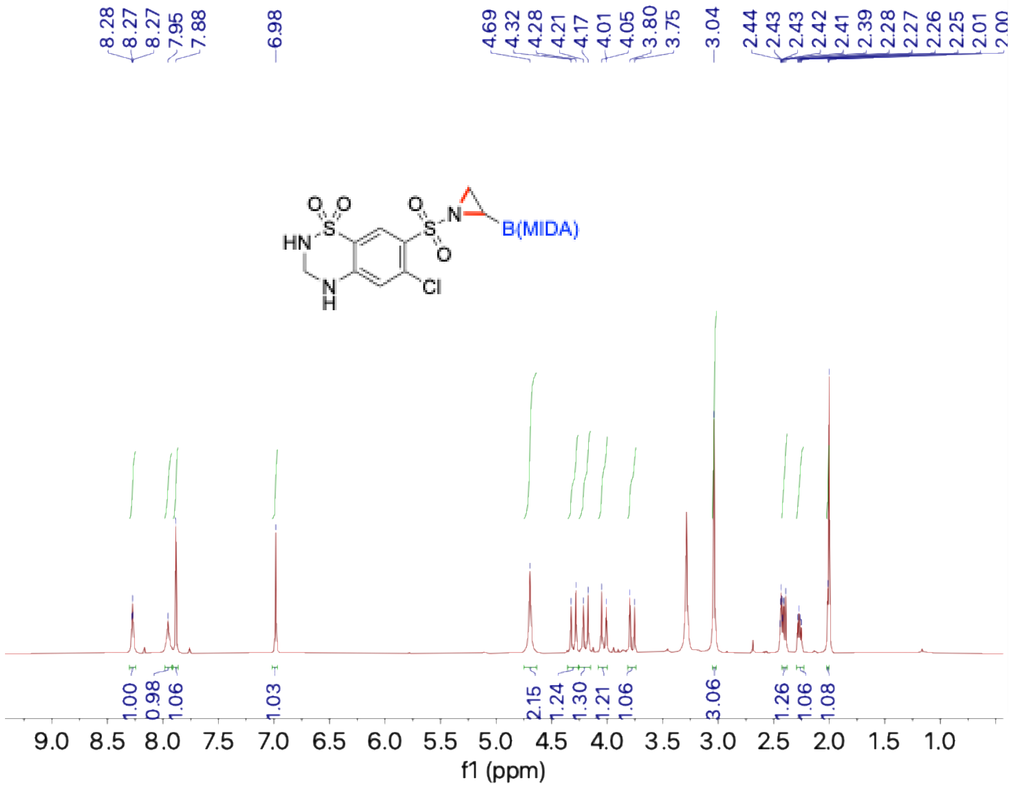

8.28
8.27
8.27
7.95
7.88
6.98
4.69
4.32
4.28
4.21
4.17
4.01
4.05
3.80
3.75
3.04
2.44
2.43
2.43
2.42
2.41
2.39
2.28
2.27
2.26
2.25
2.01
2.00
HN
S
O
O
O
O
S
N
B(MIDA)
N
H
Cl
1.00
0.98
1.06
1.03
2.15
1.24
1.30
1.21
1.06
3.06
1.26
1.06
1.08
9.0
8.5
8.0
7.5
7.0
6.5
6.0
5.5
5.0
4.5
4.0
3.5
3.0
2.5
2.0
1.5
1.0
f1 (ppm)

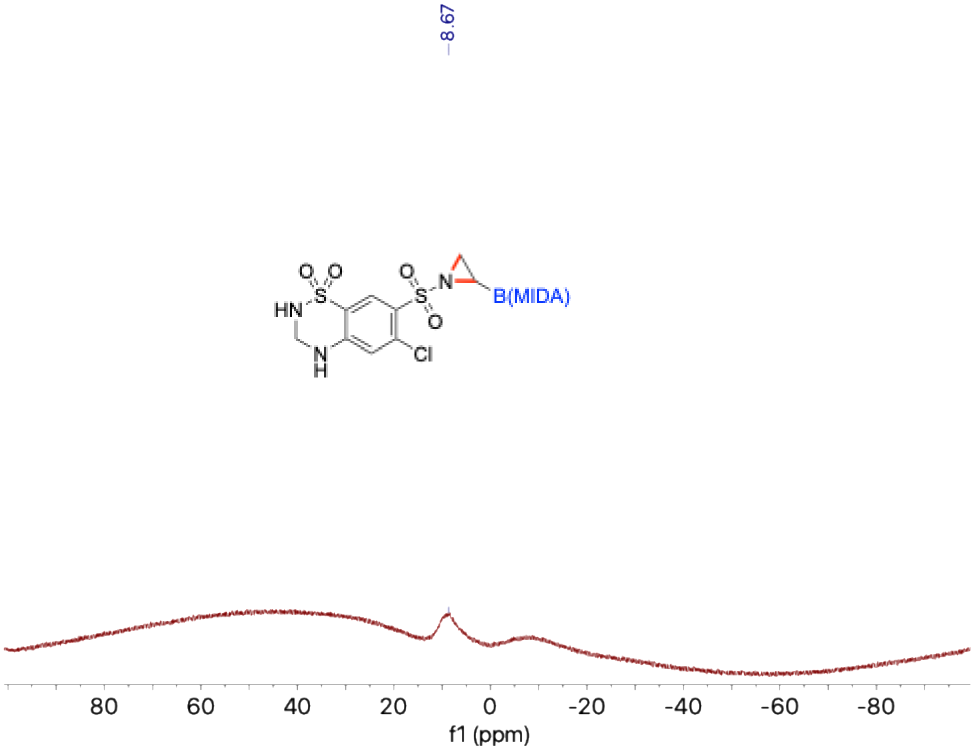

8.67
HN
S
O
O
O
O
S
N
B(MIDA)
N
H
Cl
80
60
40
20
0
-20
-40
-60
-80
f1 (ppm)

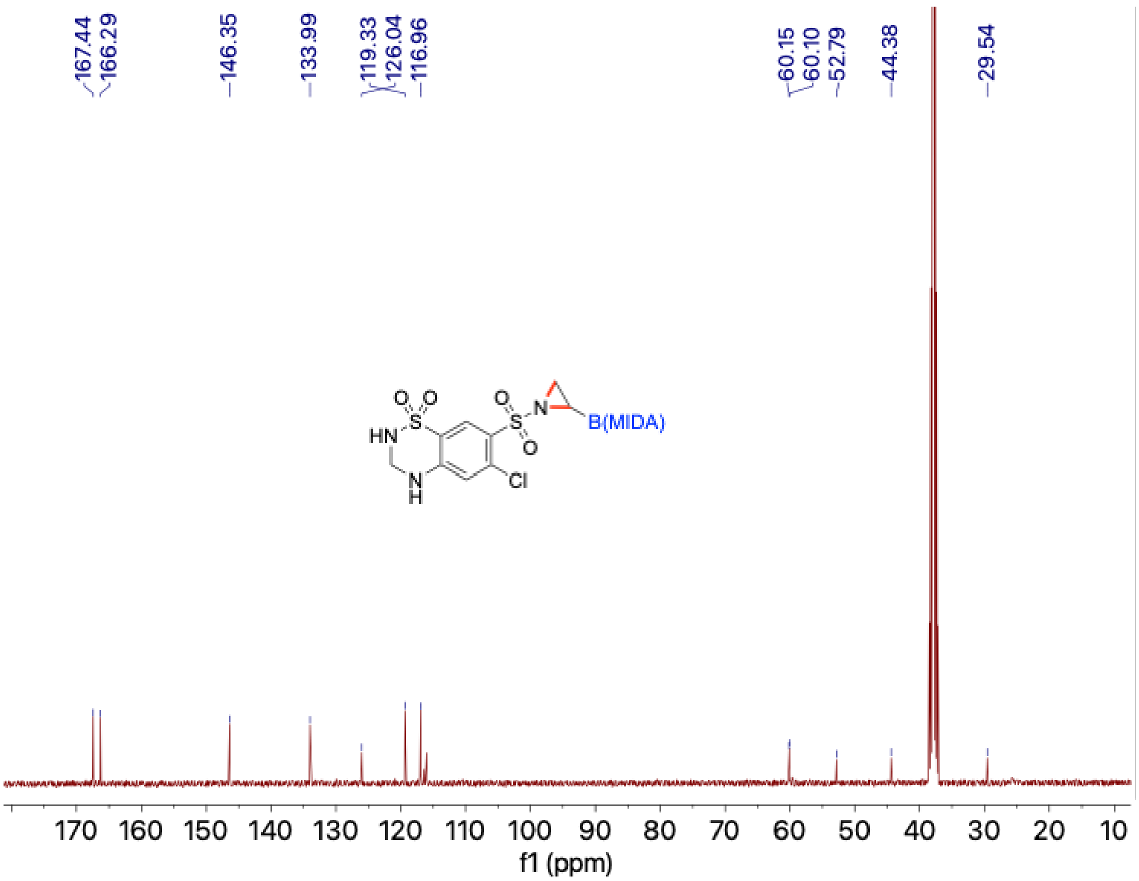
167.44
166.29
146.35
133.99
119.33
126.04
116.96
60.15
60.10
52.79
44.38
29.54
HN
S
O
N
H
Cl
N
B(MIDA)
170 160 150 140 130 120 110 100 90 80 70 60 50 40 30 20 10
f1 (ppm)

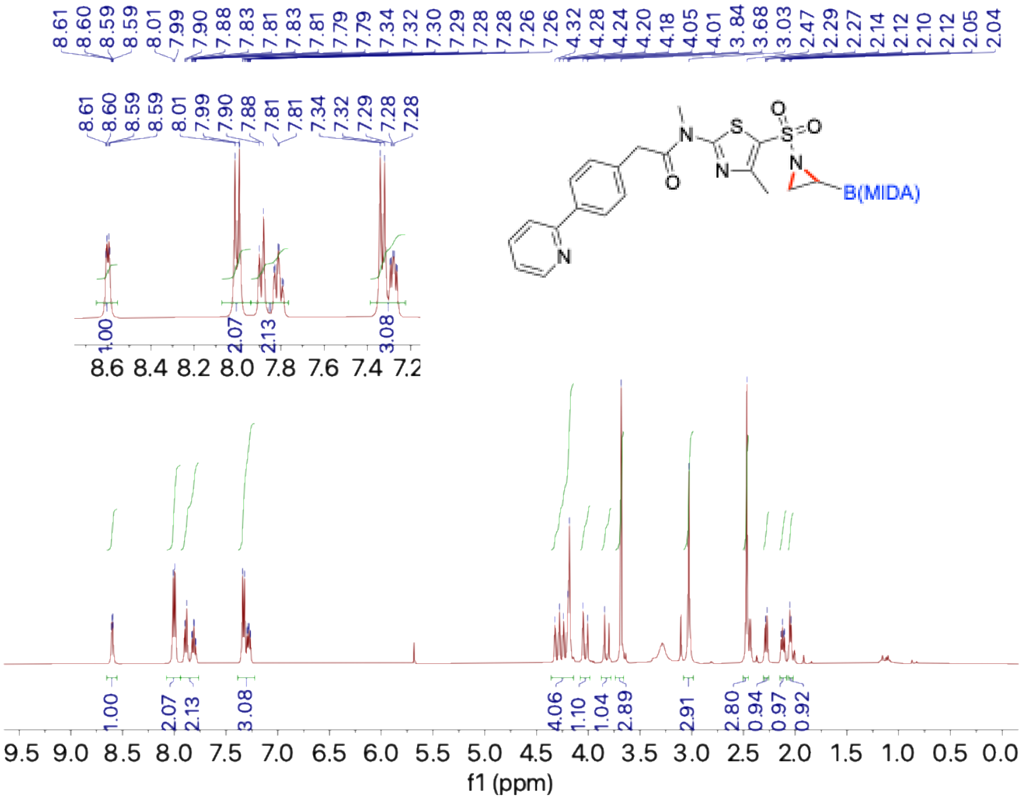

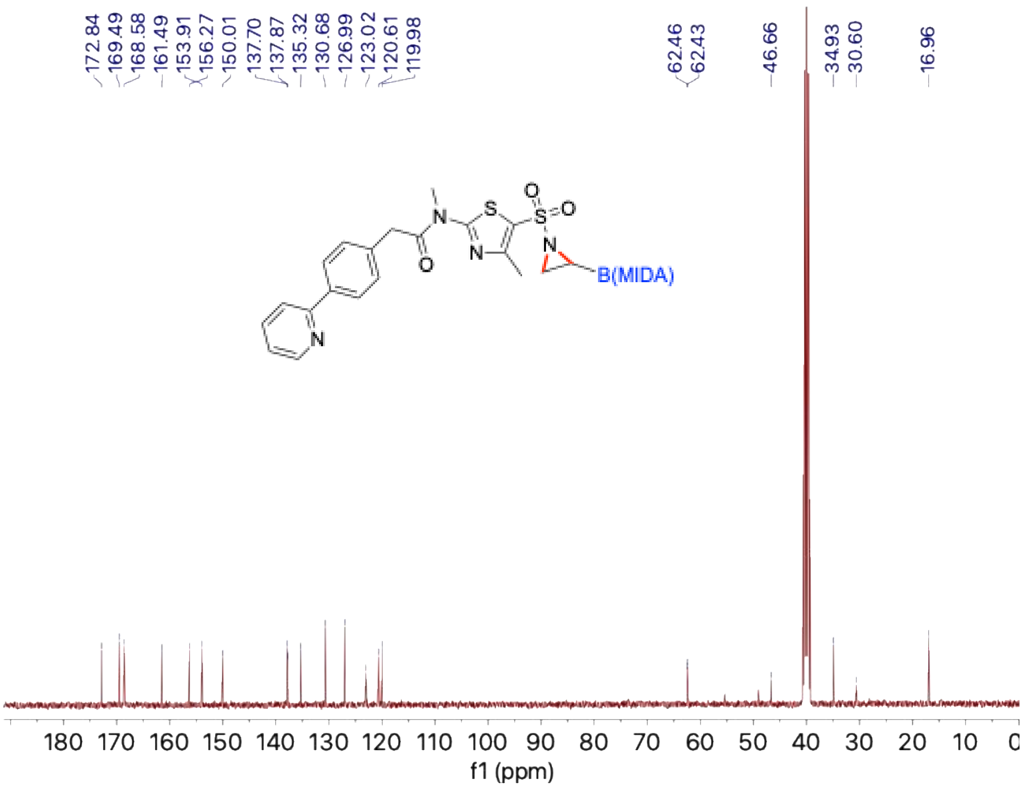

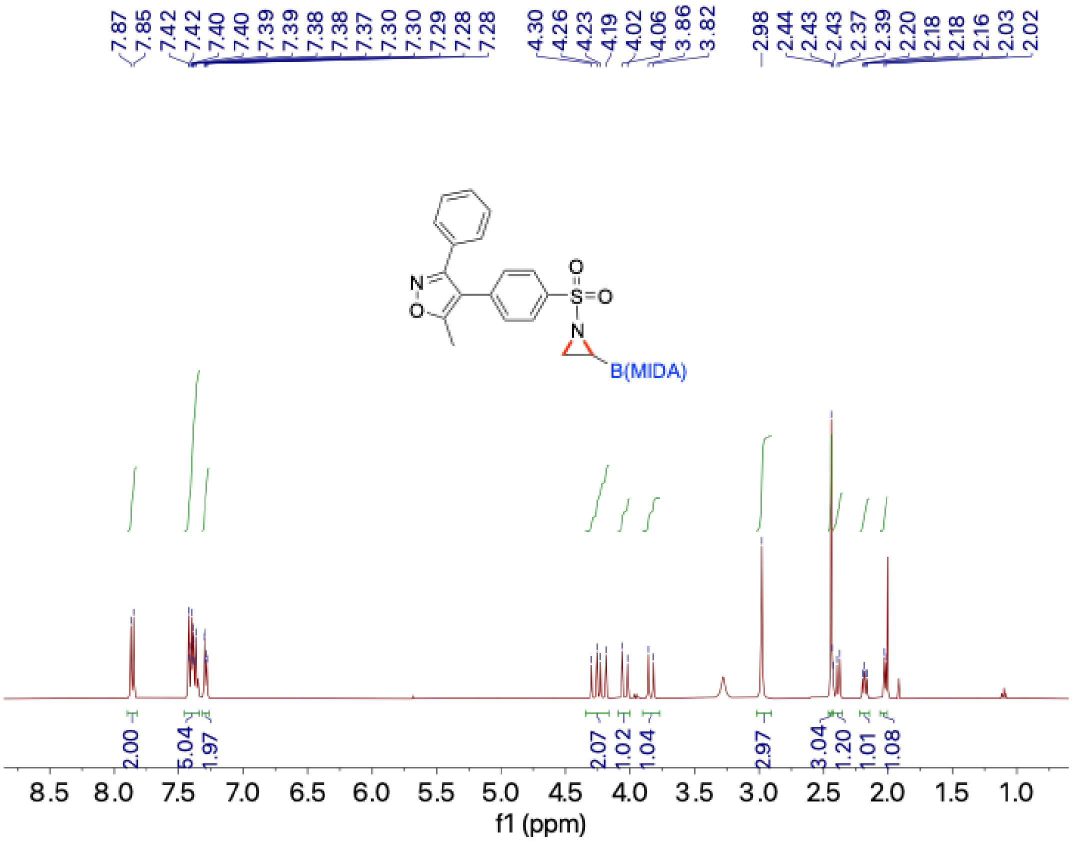

7.87
7.85
7.42
7.42
7.40
7.40
7.39
7.39
7.38
7.38
7.37
7.30
7.30
7.29
7.28
7.28
4.30
4.26
4.23
4.19
4.02
4.06
3.86
3.82
2.98
2.44
2.43
2.43
2.37
2.39
2.20
2.18
2.18
2.16
2.03
2.02
N
O
S
O
O
N
B(MIDA)
2.00
5.04
1.97
2.07
1.02
1.04
2.97
3.04
1.20
1.01
1.08
8.5
8.0
7.5
7.0
6.5
6.0
5.5
5.0
4.5
4.0
3.5
3.0
2.5
2.0
1.5
1.0
f1 (ppm)

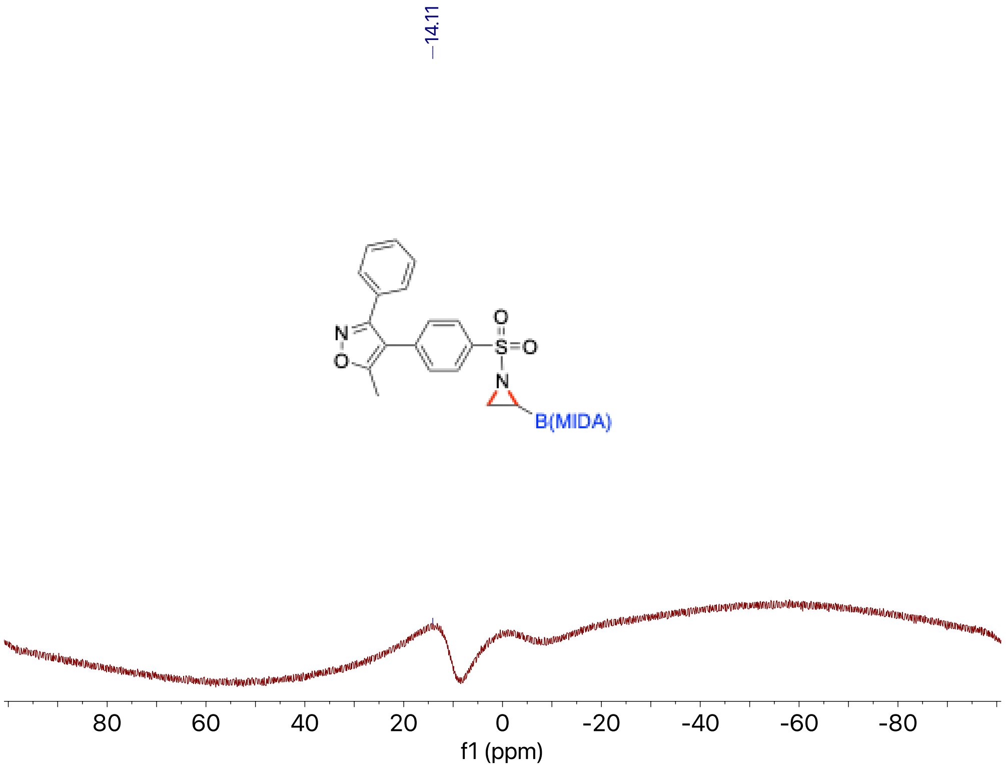
14.11
N
O
O
S
O
N
B(MIDA)
80 60 40 20 0 -20 -40 -60 -80
f1 (ppm)

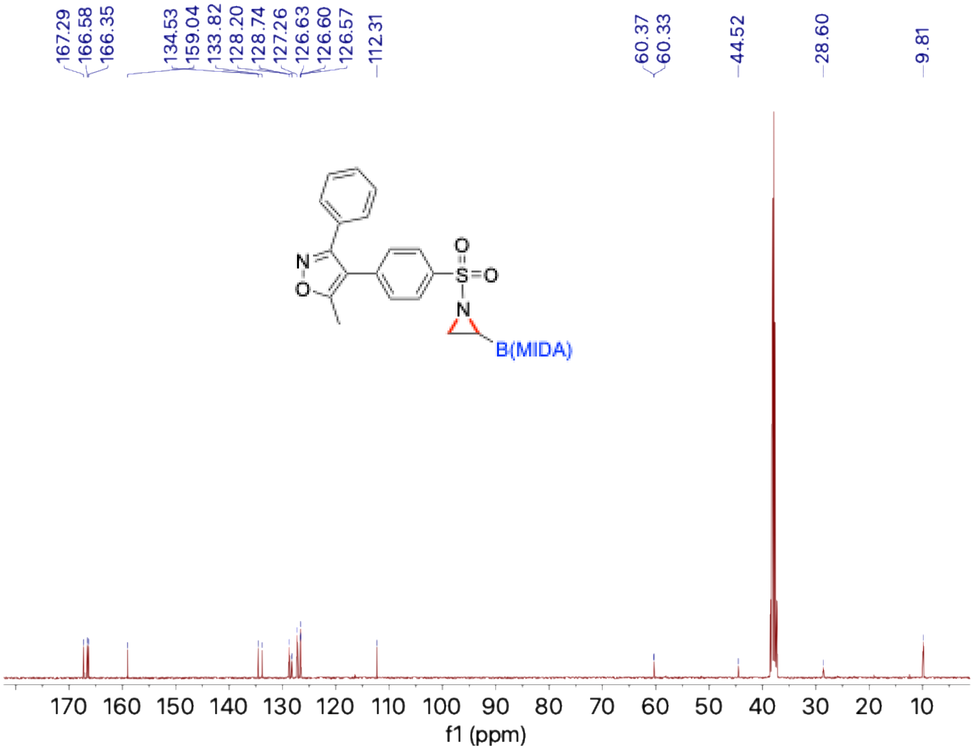
167.29
166.58
166.35
134.53
159.04
133.82
128.20
128.74
127.26
126.63
126.60
126.57
112.31
60.37
60.33
44.52
28.60
9.81
N
O
O
S
O
N
B(MIDA)
170 160 150 140 130 120 110 100 90 80 70 60 50 40 30 20 10
f1 (ppm)

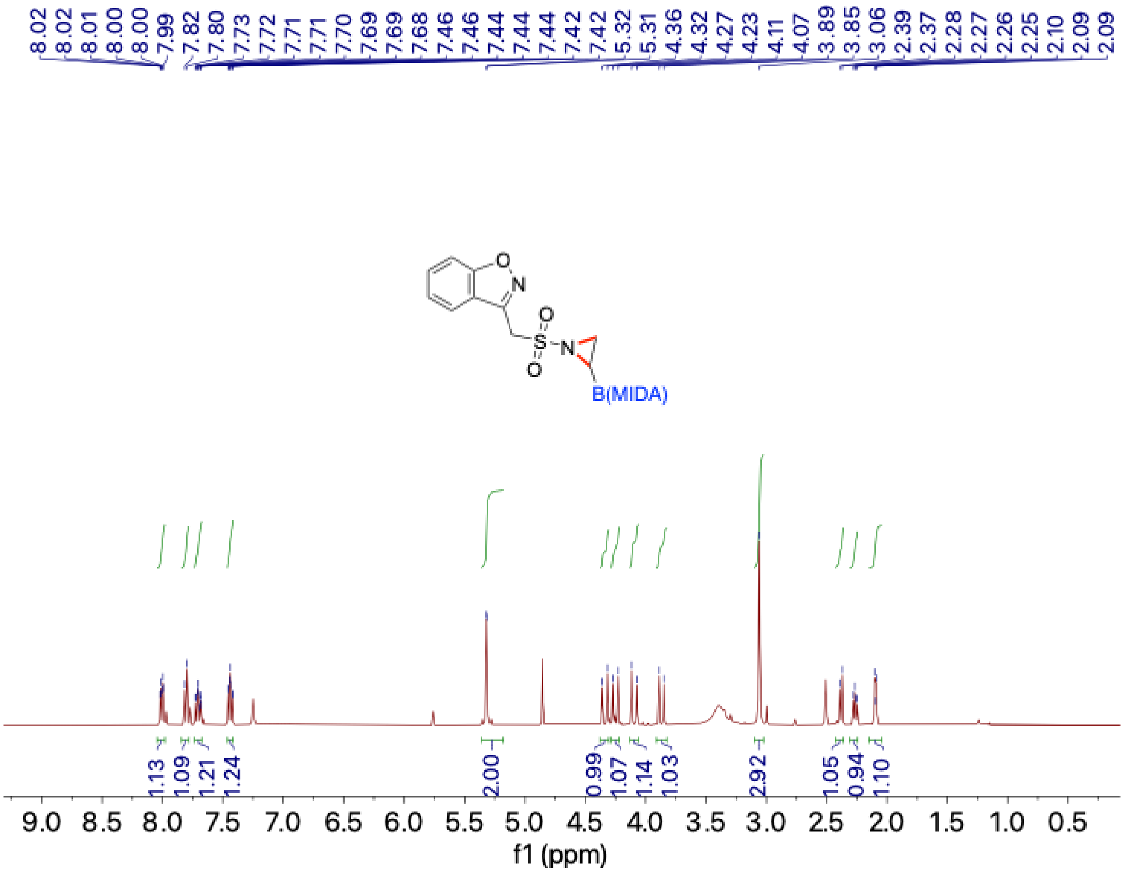
8.02
8.02
8.01
8.00
8.00
7.99
7.82
7.80
7.73
7.72
7.71
7.71
7.70
7.69
7.69
7.68
7.46
7.46
7.44
7.44
7.44
7.42
7.42
5.32
5.31
4.36
4.32
4.27
4.23
4.11
4.07
3.89
3.85
3.06
2.39
2.37
2.28
2.27
2.26
2.25
2.10
2.09
2.09
O
N
O
S
O
N
B(MIDA)
1.13
1.09
1.21
1.24
2.00
0.99
1.07
1.14
1.03
2.92
1.05
0.94
1.10
9.0 8.5 8.0 7.5 7.0 6.5 6.0 5.5 5.0 4.5 4.0 3.5 3.0 2.5 2.0 1.5 1.0 0.5
f1 (ppm)

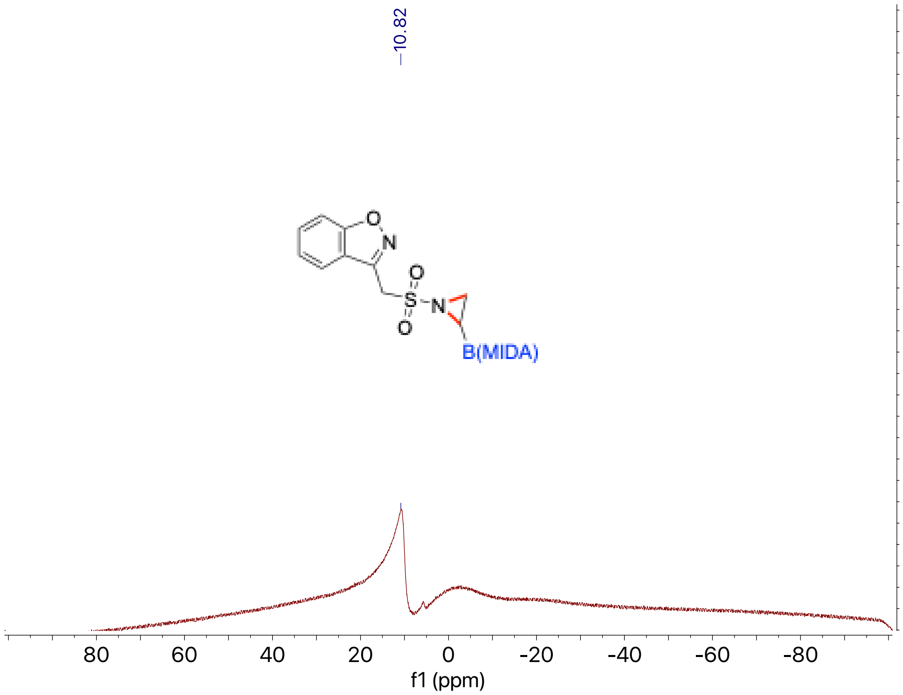
10.82
O
N
O
S
O
N
B(MIDA)
80 60 40 20 0 -20 -40 -60 -80
f1 (ppm)

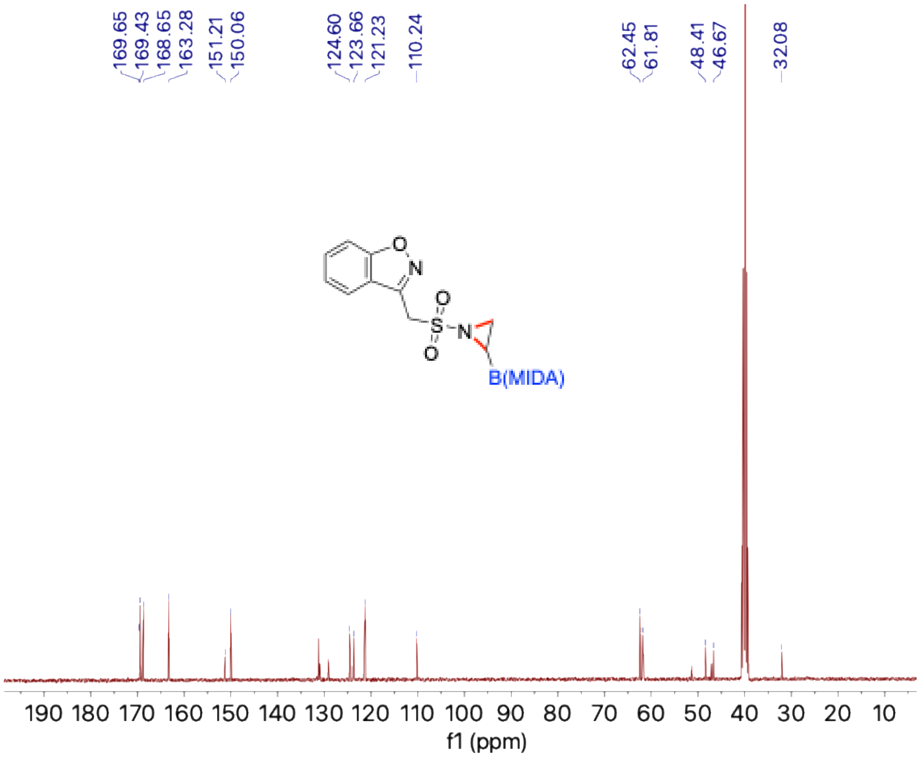
169.65
169.43
168.65
163.28
151.21
150.06
124.60
123.66
121.23
110.24
62.45
61.81
48.41
46.67
32.08
B(MIDA)
190 180 170 160 150 140 130 120 110 100 90 80 70 60 50 40 30 20 10
f1 (ppm)

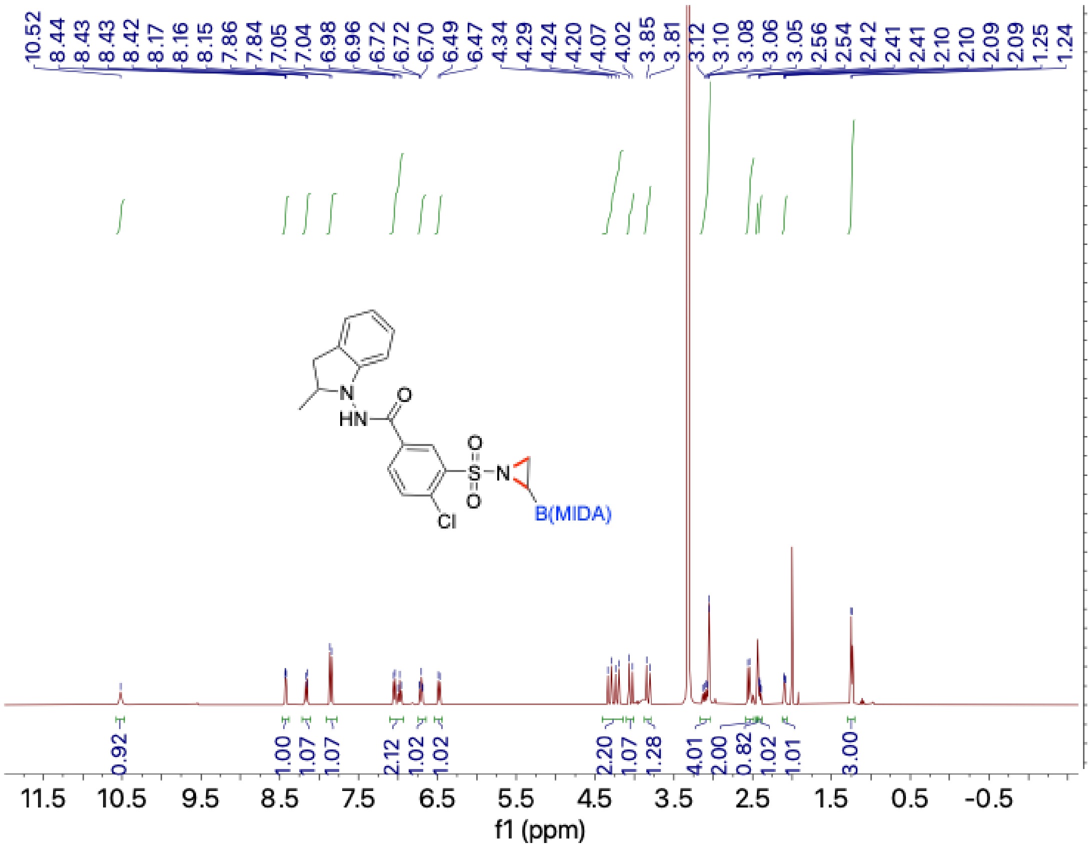
10.52 8.44 8.43 8.43 8.42 8.17 8.16 8.15 7.86 7.84 7.05 7.04 6.98 6.96 6.72 6.72 6.70 6.49 6.47 4.34 4.29 4.24 4.20 4.07 4.02 3.85 3.81 3.12 3.10 3.08 3.06 3.05 2.56 2.54 2.42 2.41 2.41 2.10 2.10 2.09 2.09 1.25 1.24
N
HN
O
O
S
O
N
Cl
B(MIDA)
0.92 1.00 1.07 1.07 2.12 1.02 1.02 2.20 1.07 1.28 4.01 2.00 0.82 1.02 1.01 3.00
11.5 10.5 9.5 8.5 7.5 6.5 5.5 4.5 3.5 2.5 1.5 0.5 -0.5
f1 (ppm)

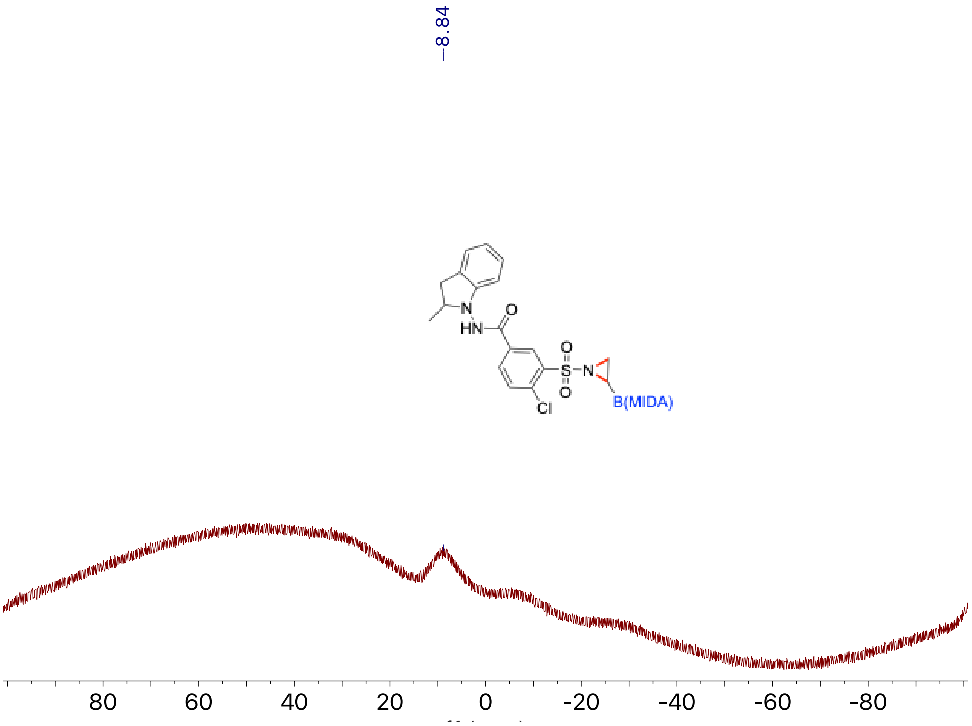
8.84
N
HN
O
O
S
O
N
Cl
B(MIDA)
80 60 40 20 0 -20 -40 -60 -80

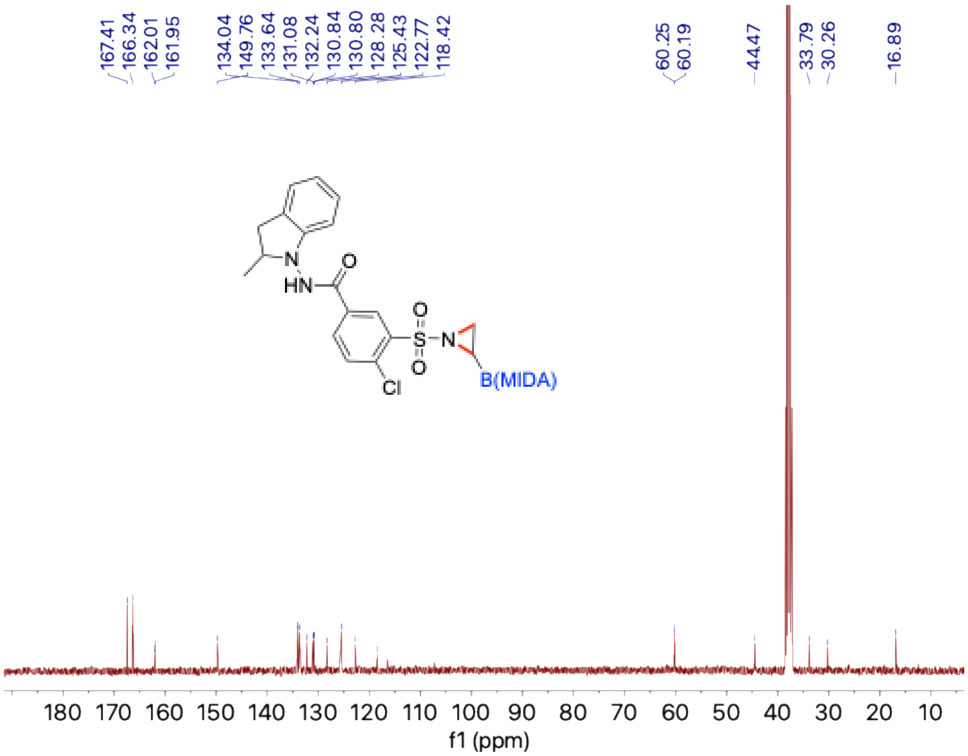
167.41
166.34
162.01
161.95
134.04
149.76
133.64
131.08
132.24
130.84
130.80
128.28
125.43
122.77
118.42
60.25
60.19
44.47
33.79
30.26
16.89
O
N
HN
O
S
O
N
Cl
B(MIDA)
180 170 160 150 140 130 120 110 100 90 80 70 60 50 40 30 20 10
f1 (ppm)

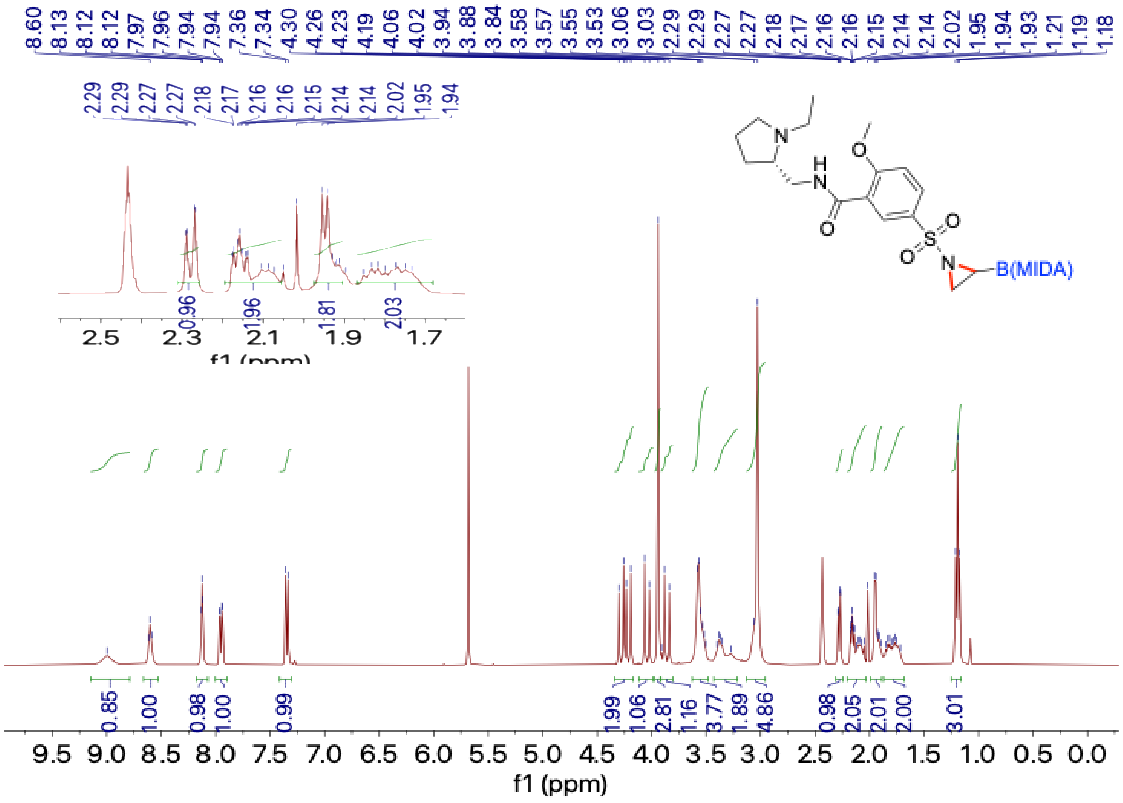

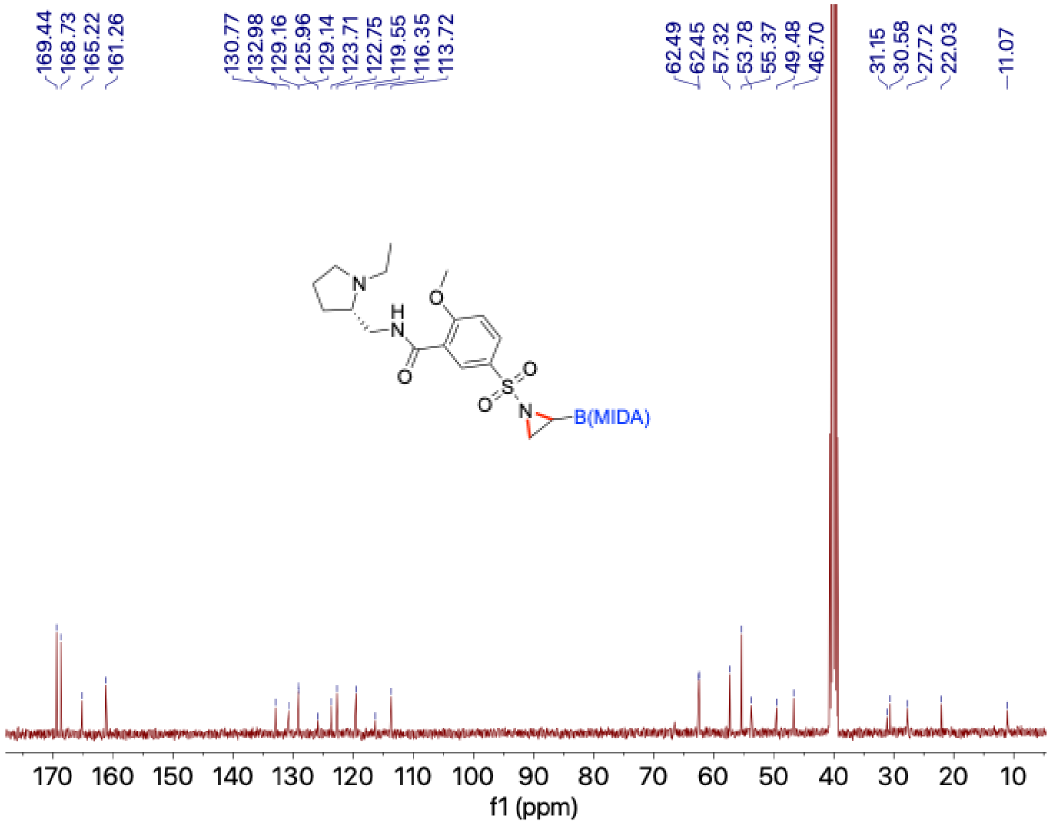

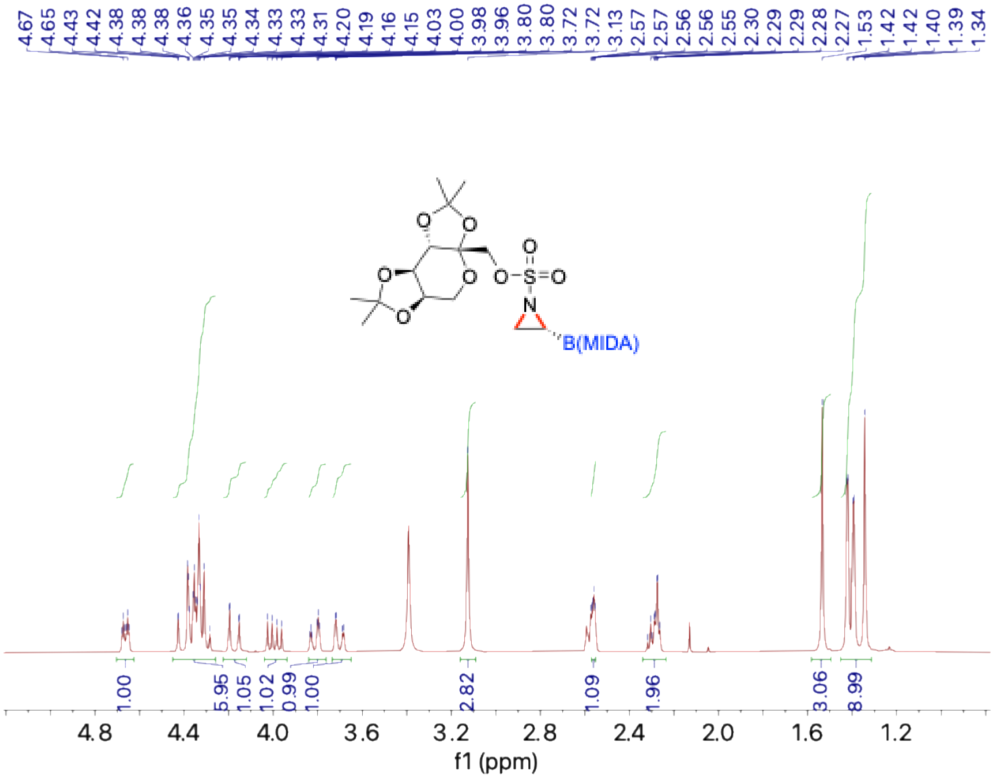

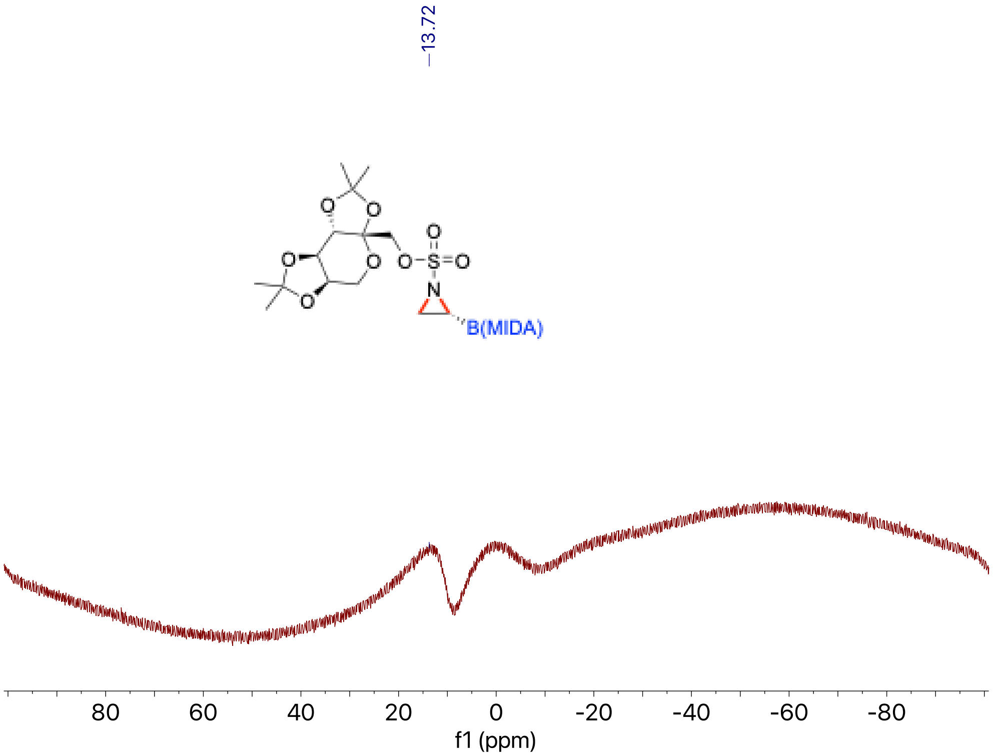

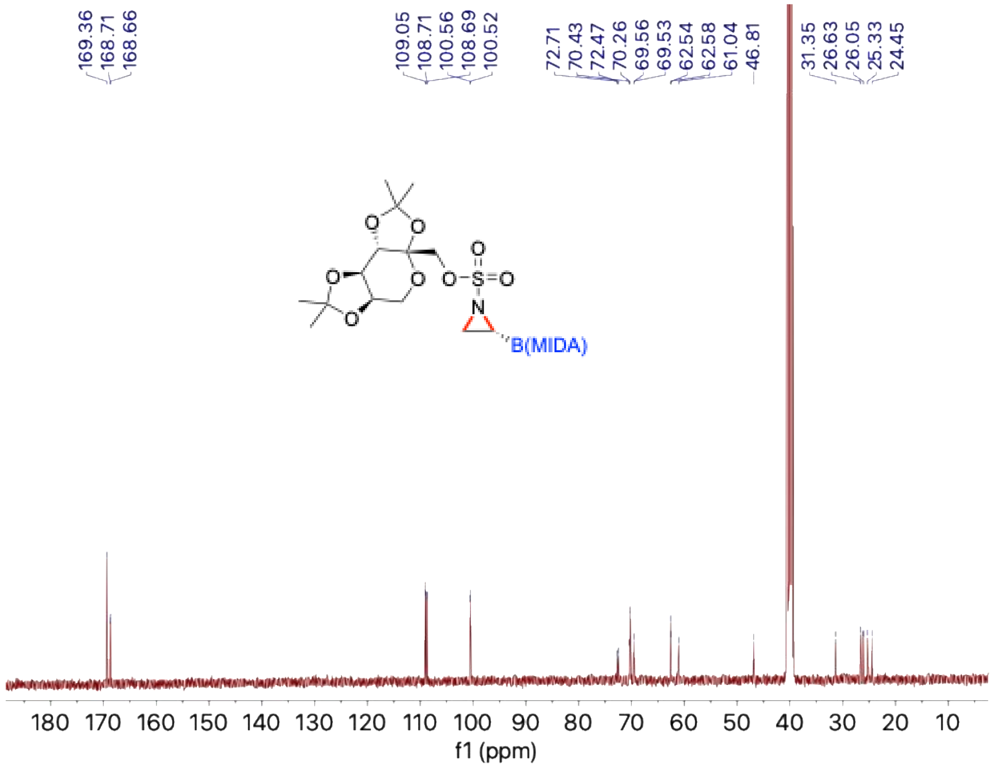
169.36
168.71
168.66
109.05
108.71
100.56
108.69
100.52
72.71
70.43
72.47
70.26
69.56
69.53
62.54
62.58
61.04
46.81
31.35
26.63
26.05
25.33
24.45
O
O
O
O
O
O
O
S
O
N
B(MIDA)
180 170 160 150 140 130 120 110 100 90 80 70 60 50 40 30 20 10
f1 (ppm)

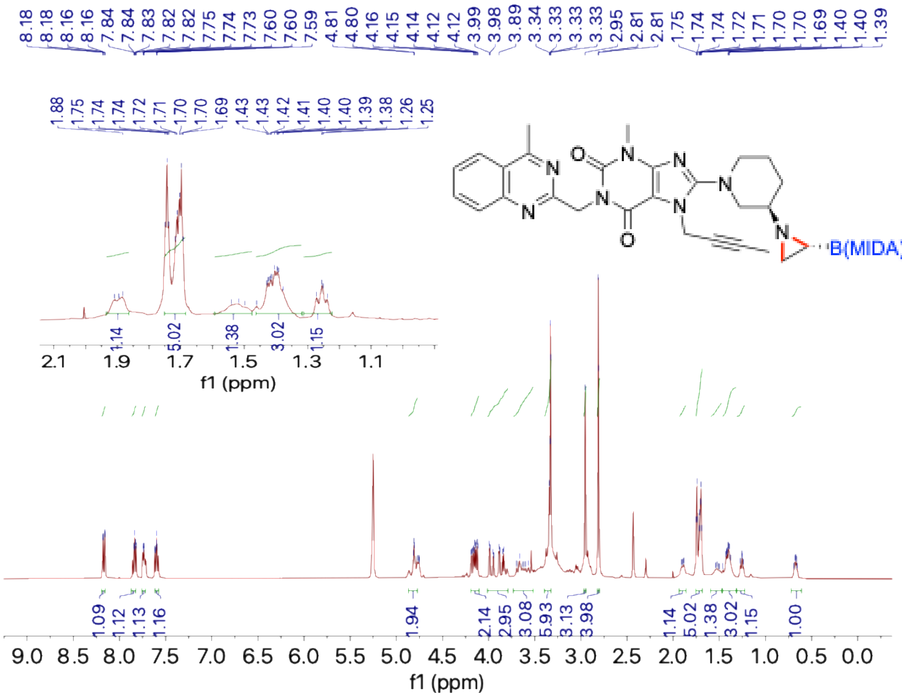
8.18 8.18 8.16 8.16 7.84 7.84 7.83 7.82 7.82 7.75 7.74 7.73 7.60 7.59 4.81 4.80 4.16 4.15 4.14 4.12 4.12 3.99 3.98 3.89 3.34 3.33 3.33 3.33 2.95 2.81 2.81 1.75 1.74 1.74 1.72 1.71 1.70 1.70 1.69 1.40 1.39
1.88 1.75 1.74 1.74 1.72 1.71 1.70 1.70 1.69 1.43 1.43 1.42 1.41 1.40 1.40 1.39 1.38 1.26 1.25
B(MIDA)
1.14 5.02 1.38 3.02 1.15
2.1 1.9 1.7 1.5 1.3 1.1
f1 (ppm)
1.09 1.12 1.13 1.16 1.94 2.14 2.95 3.08 5.93 3.13 3.98 1.14 5.02 1.38 3.02 1.15 1.00
9.0 8.5 8.0 7.5 7.0 6.5 6.0 5.5 5.0 4.5 4.0 3.5 3.0 2.5 2.0 1.5 1.0 0.5 0.0
f1 (ppm)

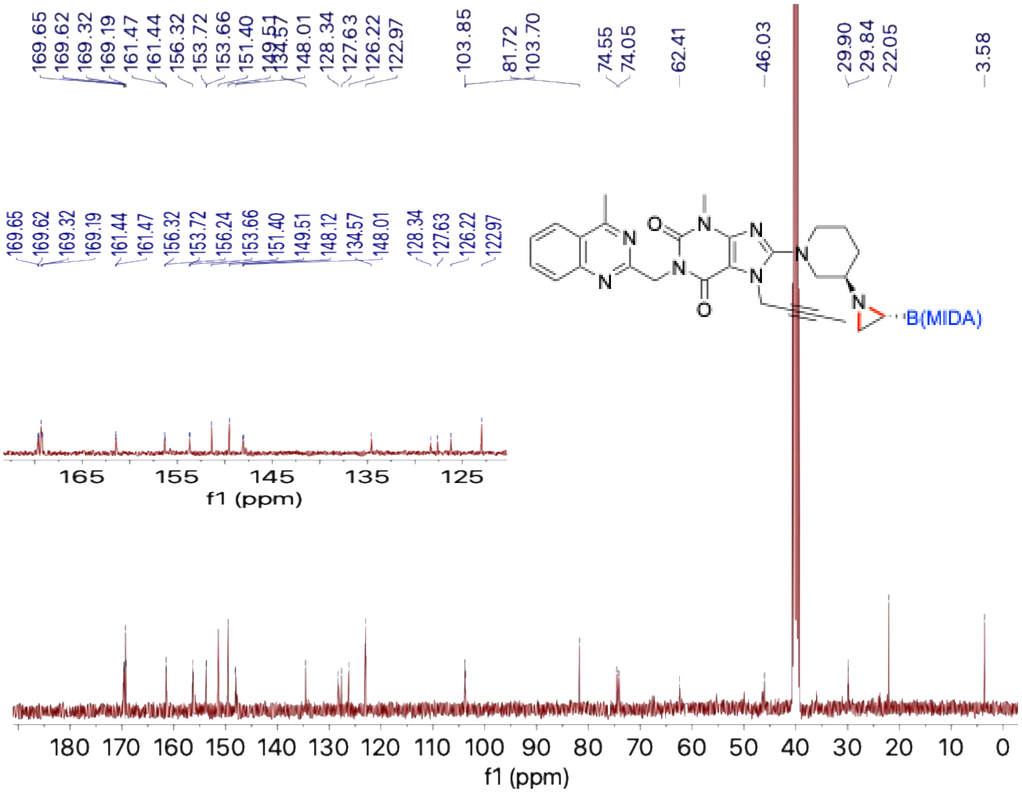
169.65 169.62 169.32 169.19 161.47 161.44 156.32 153.72 153.66 151.40 149.51 134.57 148.01 128.34 127.63 126.22 122.97 103.85 81.72 103.70 74.55 74.05 62.41 46.03 29.90 29.84 22.05 3.58
169.65 169.62 169.32 169.19 161.44 161.47 156.32 153.72 156.24 153.66 151.40 149.51 148.12 134.57 148.01 128.34 127.63 126.22 122.97
B(MIDA)
165 155 145 135 125
f1 (ppm)
180 170 160 150 140 130 120 110 100 90 80 70 60 50 40 30 20 10 0
f1 (ppm)

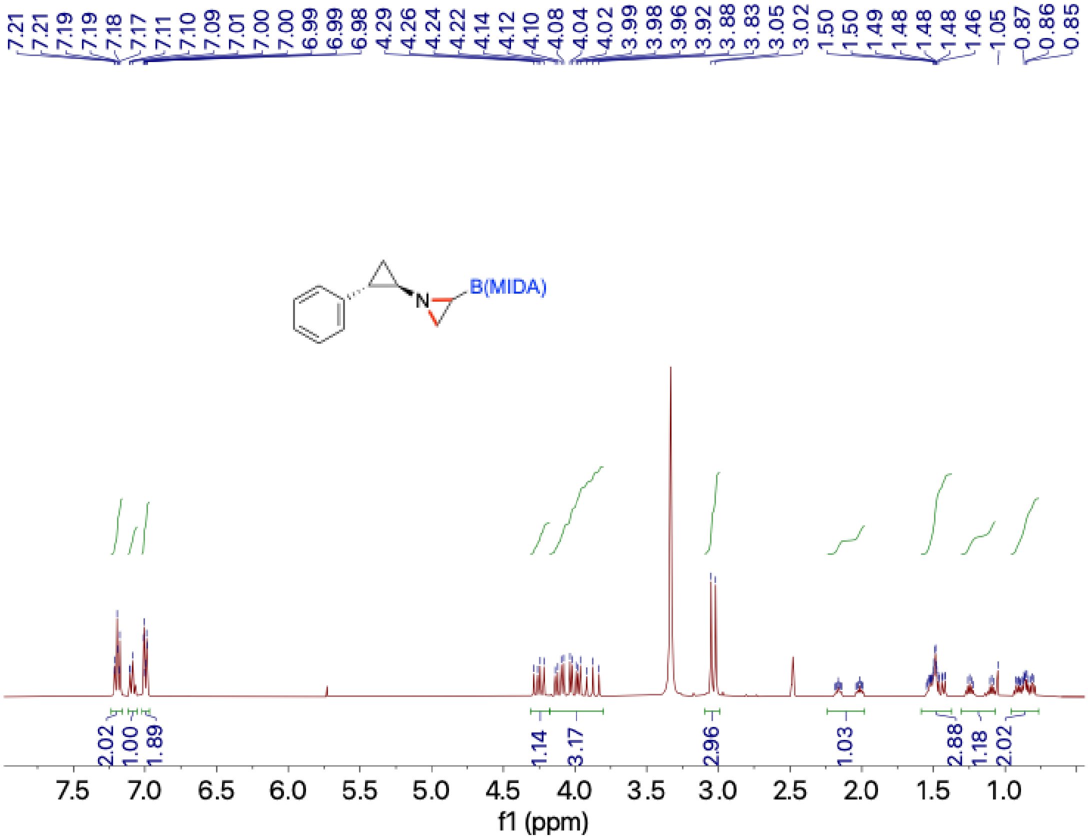
7.21
7.21
7.19
7.19
7.18
7.17
7.11
7.10
7.09
7.01
7.00
7.00
6.99
6.99
6.98
4.29
4.26
4.24
4.22
4.14
4.12
4.10
4.08
4.04
4.02
3.99
3.98
3.96
3.92
3.88
3.83
3.05
3.02
1.50
1.50
1.49
1.48
1.48
1.48
1.46
1.05
0.87
0.86
0.85
N
B(MIDA)
2.02
1.00
1.89
1.14
3.17
2.96
1.03
2.88
1.18
2.02
f1 (ppm)

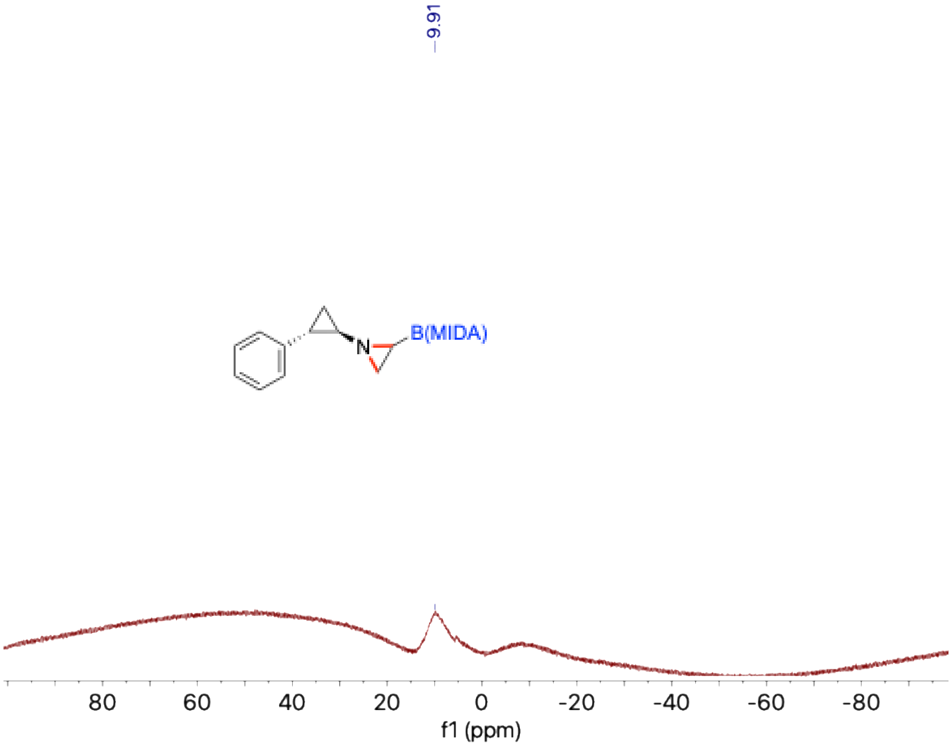
9.91
N
B(MIDA)
f1 (ppm)

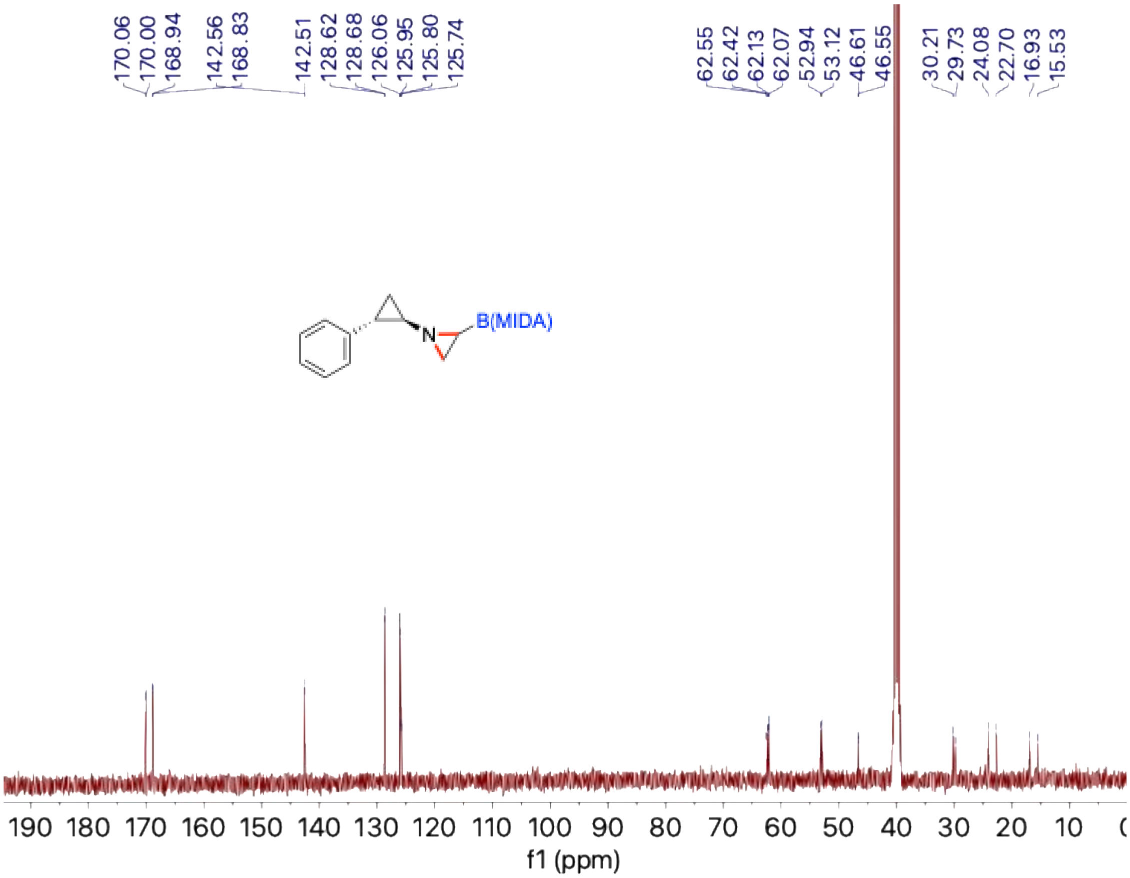
170.06
170.00
168.94
142.56
168.83
142.51
128.62
128.68
126.06
125.95
125.80
125.74
62.55
62.42
62.13
62.07
52.94
53.12
46.61
46.55
30.21
29.73
24.08
22.70
16.93
15.53
B(MIDA)
N
190 180 170 160 150 140 130 120 110 100 90 80 70 60 50 40 30 20 10
f1 (ppm)

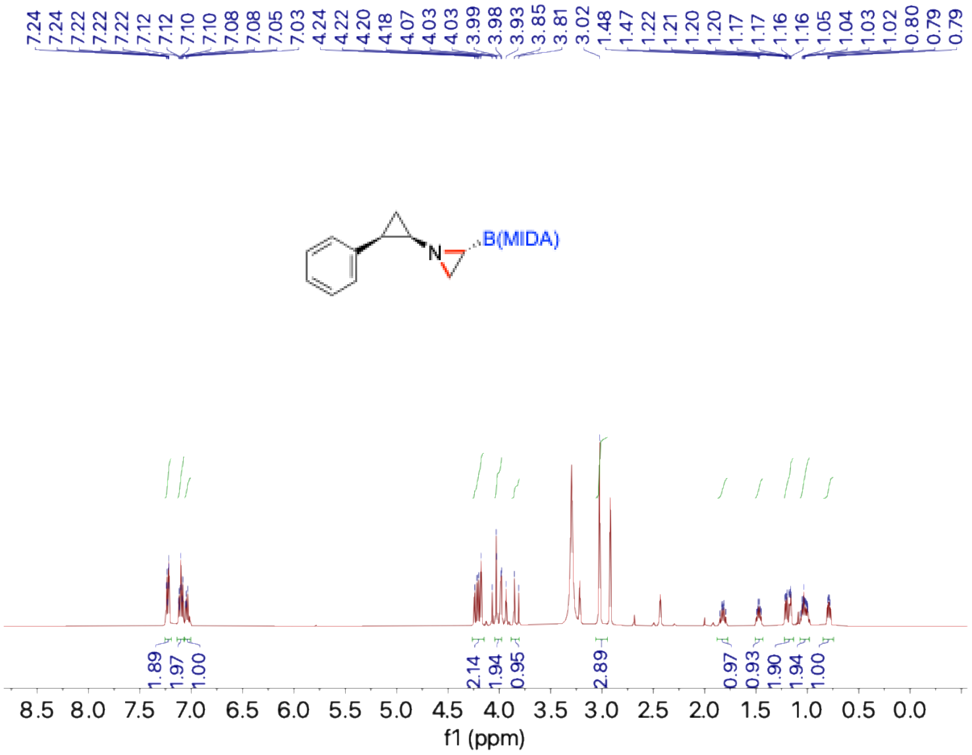
7.24 7.24 7.22 7.22 7.22 7.12 7.12 7.10 7.10 7.08 7.08 7.05 7.03 4.24 4.22 4.20 4.18 4.07 4.03 4.03 3.99 3.98 3.93 3.85 3.81 3.02 1.48 1.47 1.22 1.21 1.20 1.20 1.17 1.17 1.16 1.16 1.05 1.04 1.03 1.02 0.80 0.79 0.79
N
B(MIDA)
1.89 1.97 1.00 2.14 1.94 0.95 2.89 0.97 0.93 1.90 1.94 1.00
8.5 8.0 7.5 7.0 6.5 6.0 5.5 5.0 4.5 4.0 3.5 3.0 2.5 2.0 1.5 1.0 0.5 0.0
f1 (ppm)

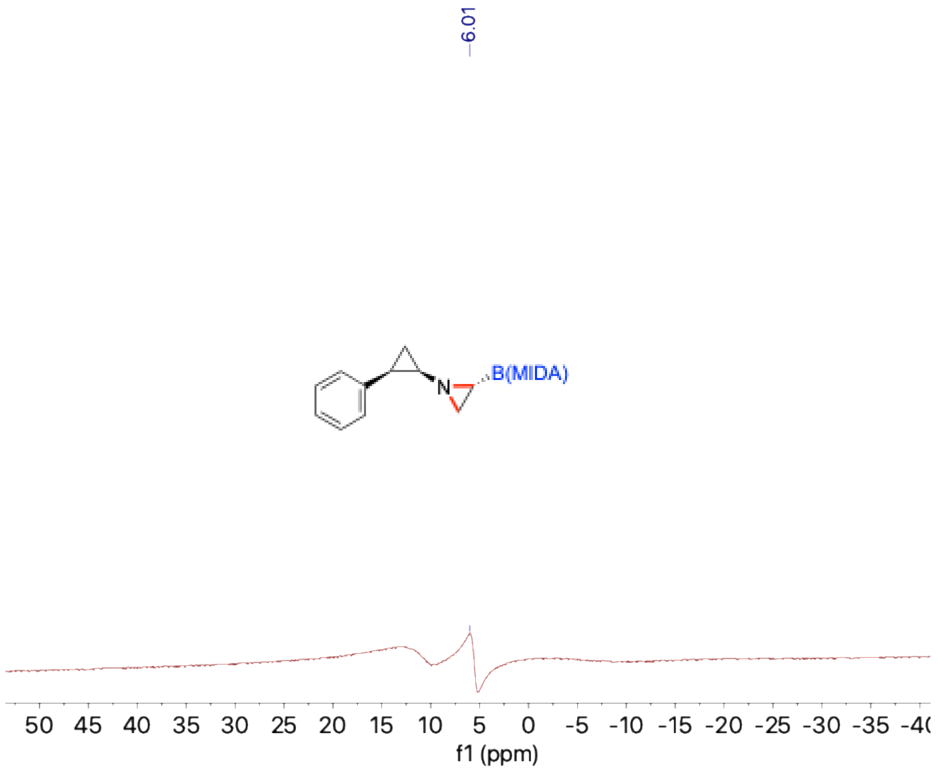
6.01
N
B(MIDA)
50 45 40 35 30 25 20 15 10 5 0 -5 -10 -15 -20 -25 -30 -35 -40
f1 (ppm)

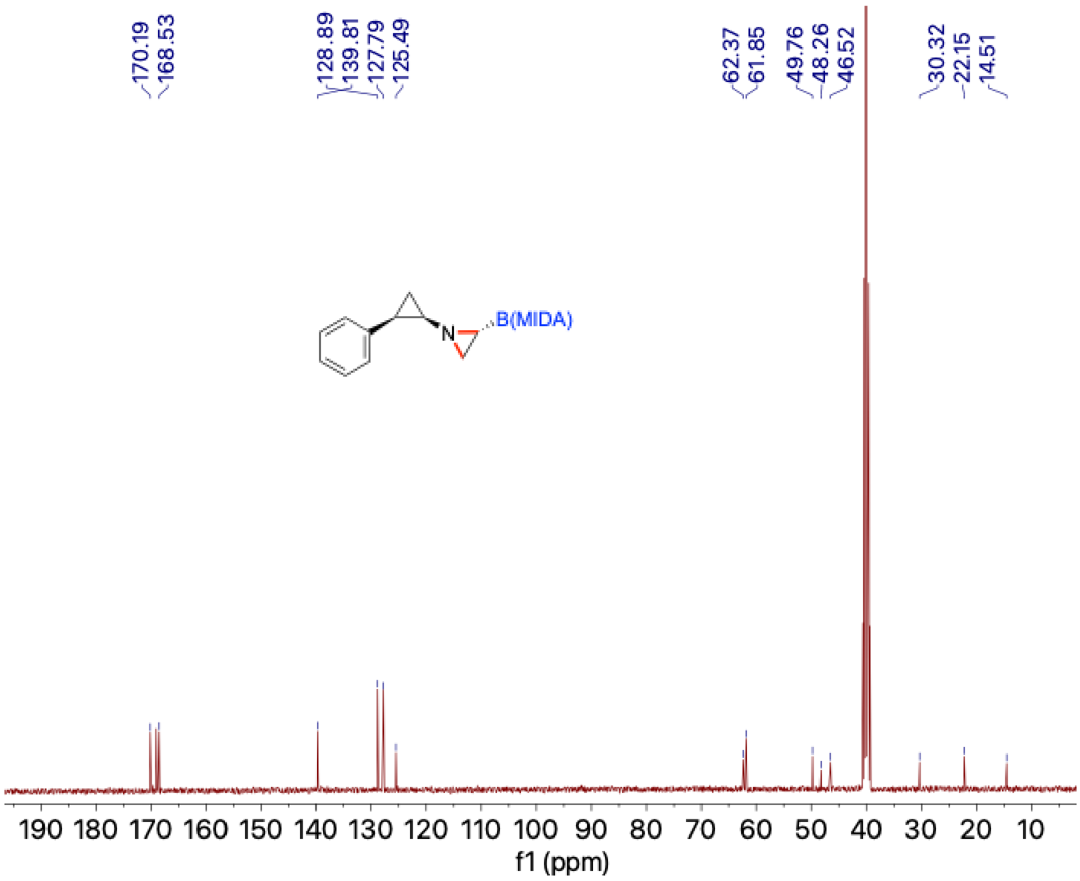
170.19
168.53
128.89
139.81
127.79
125.49
62.37
61.85
49.76
48.26
46.52
30.32
22.15
14.51
B(MIDA)
N
190 180 170 160 150 140 130 120 110 100 90 80 70 60 50 40 30 20 10
f1 (ppm)

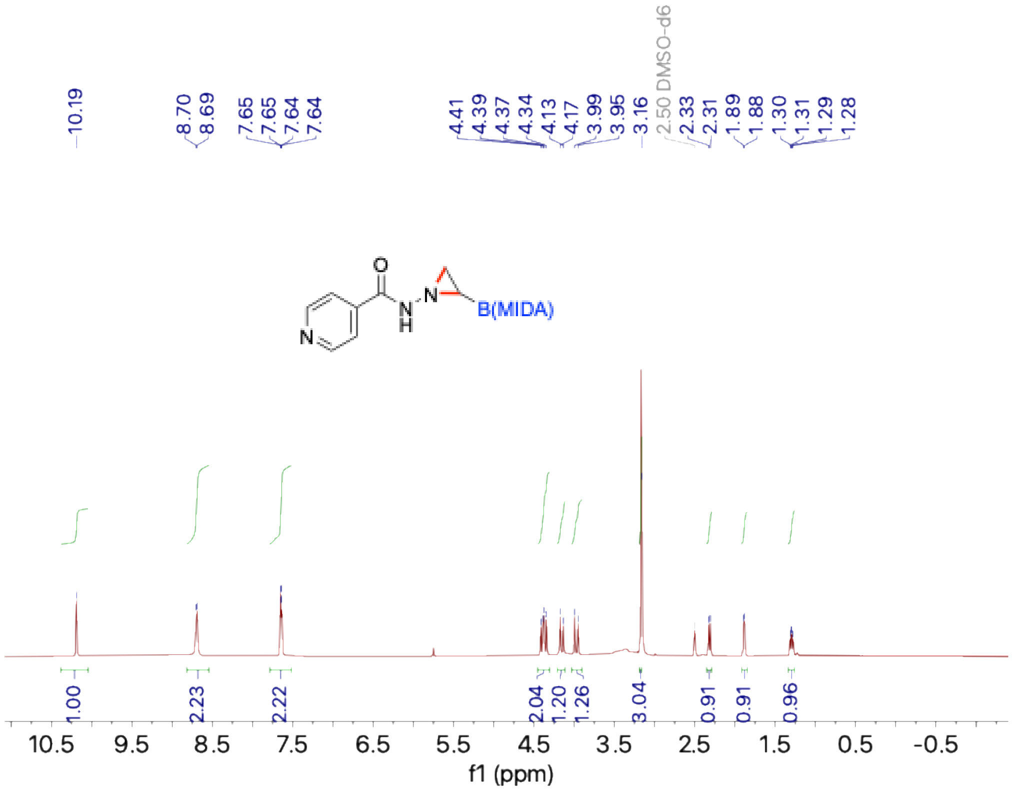
10.19
8.70
8.69
7.65
7.65
7.64
7.64
4.41
4.39
4.37
4.34
4.13
4.17
3.99
3.95
3.16
2.50 DMSO-d6
2.33
2.31
1.89
1.88
1.30
1.31
1.29
1.28
O
N
N
H
N
B(MIDA)
1.00
2.23
2.22
2.04
1.20
1.26
3.04
0.91
0.91
0.96
10.5 9.5 8.5 7.5 6.5 5.5 4.5 3.5 2.5 1.5 0.5 -0.5
f1 (ppm)

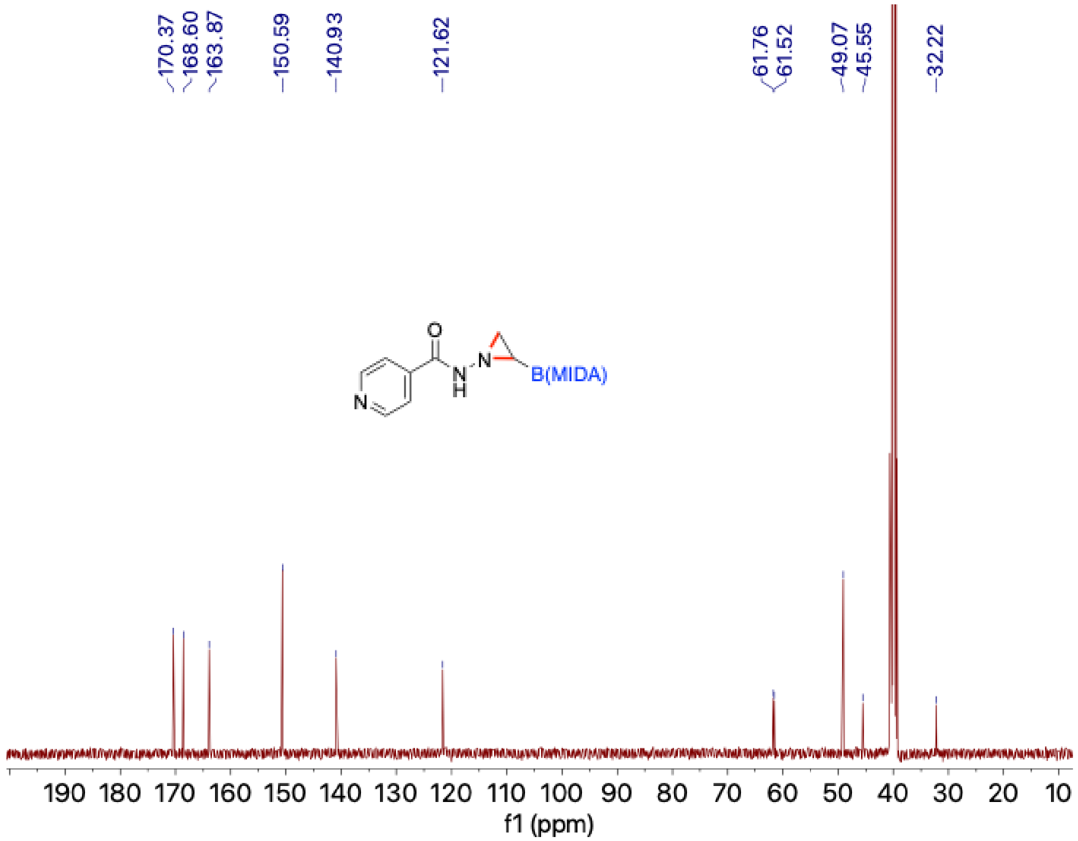
170.37
168.60
163.87
150.59
140.93
121.62
61.76
61.52
49.07
45.55
32.22
O
N
N
H
N
B(MIDA)
190 180 170 160 150 140 130 120 110 100 90 80 70 60 50 40 30 20 10
f1 (ppm)

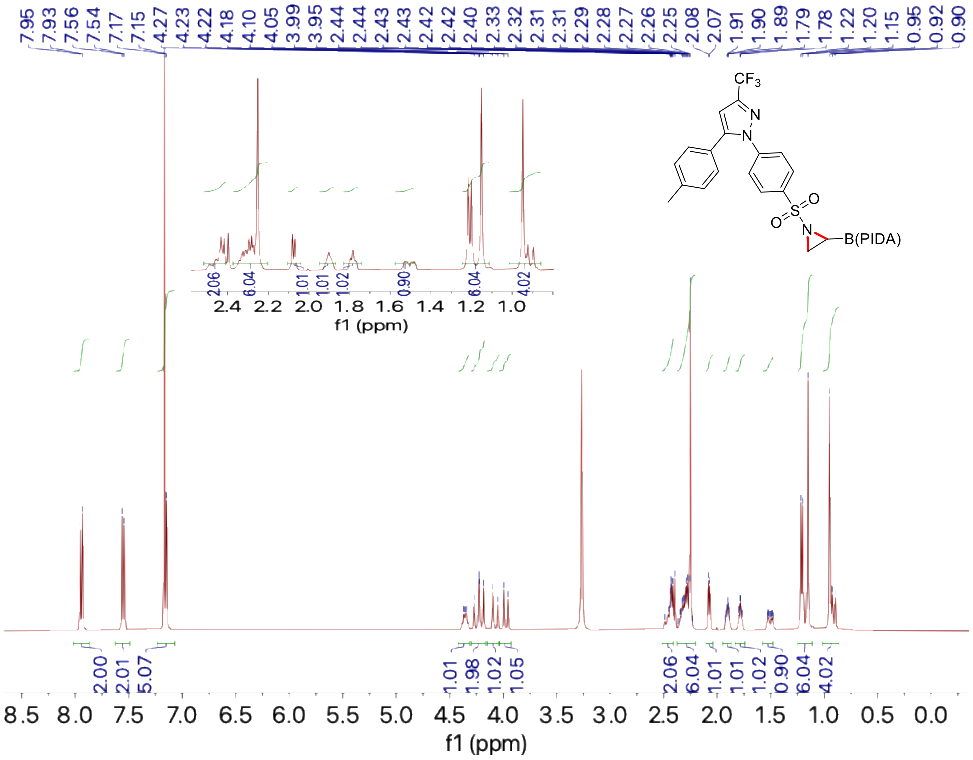
7.95
7.93
7.56
7.54
7.17
7.15
4.27
4.23
4.22
4.18
4.10
4.05
3.99
3.95
2.44
2.44
2.43
2.43
2.42
2.42
2.40
2.33
2.32
2.31
2.31
2.29
2.28
2.27
2.26
2.25
2.08
2.07
1.91
1.90
1.89
1.79
1.78
1.22
1.20
1.15
0.95
0.92
0.90
CF3
N
N
O
S
O
N
B(PIDA)
2.00
2.01
5.07
1.01
1.98
1.02
1.05
2.06
6.04
1.01
1.01
1.02
0.90
6.04
4.02
f1 (ppm)

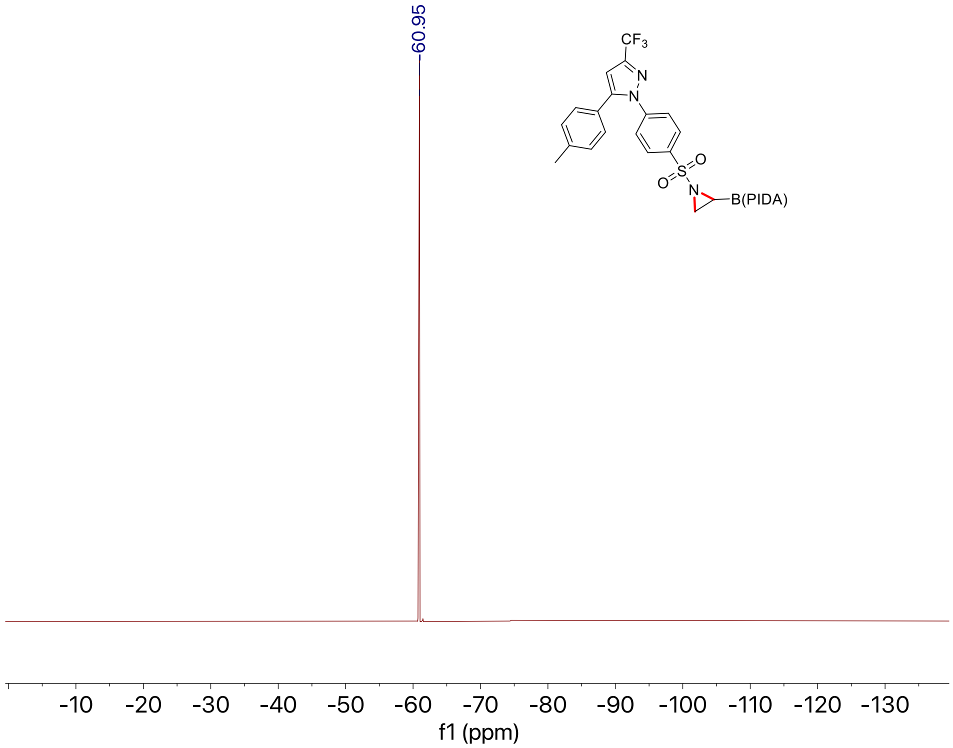
-60.95
CF3
N
N
O
S
O
N
B(PIDA)
f1 (ppm)

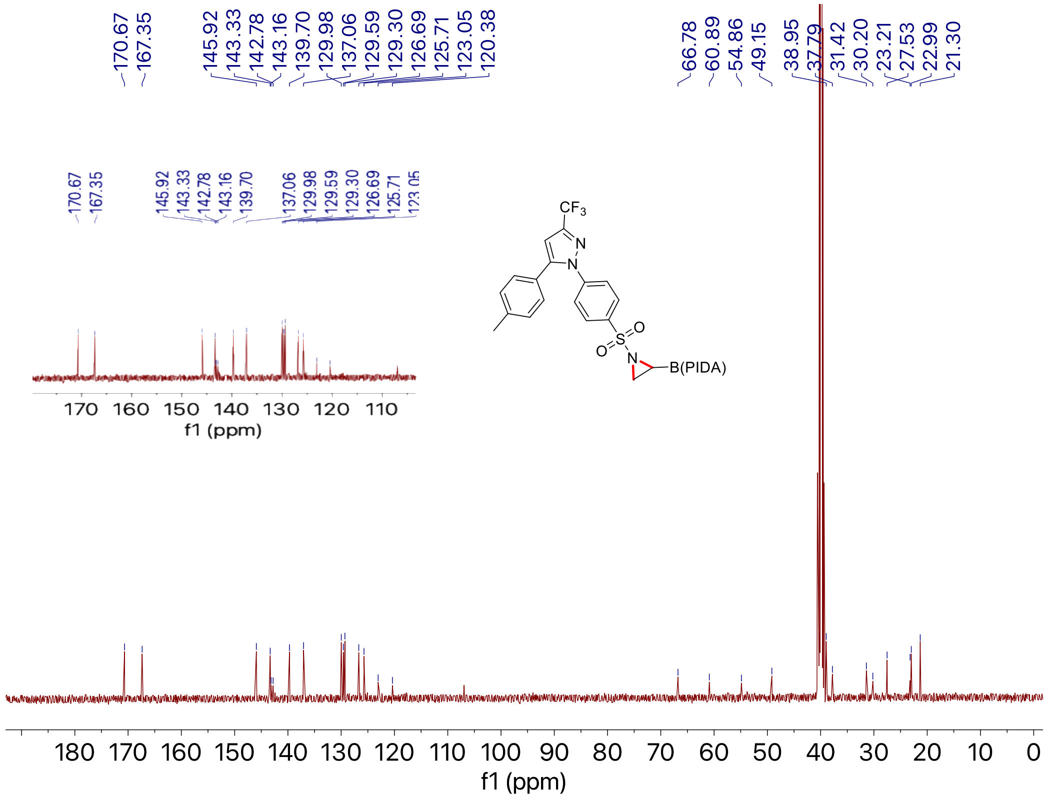
170.67
167.35
145.92
143.33
142.78
143.16
139.70
129.98
137.06
129.59
129.30
126.69
125.71
123.05
120.38
66.78
60.89
54.86
49.15
38.95
37.79
31.42
30.20
23.21
27.53
22.99
21.30
CF3
N
N
S
O
O
B(PIDA)
f1 (ppm)

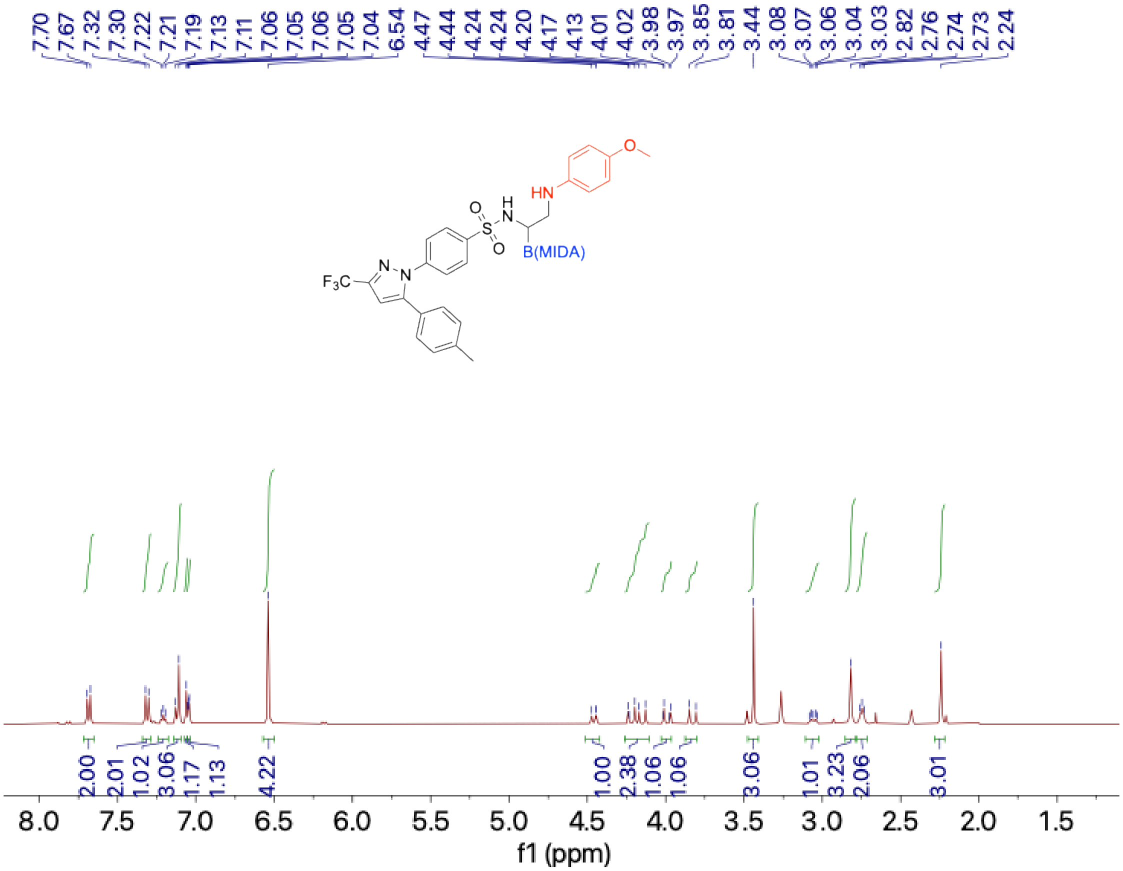
7.70
7.67
7.32
7.30
7.22
7.21
7.19
7.13
7.11
7.06
7.05
7.06
7.05
7.04
6.54
4.47
4.44
4.24
4.24
4.20
4.17
4.13
4.01
4.02
3.98
3.97
3.85
3.81
3.44
3.08
3.07
3.06
3.04
3.03
2.82
2.76
2.74
2.73
2.24
HN
O
H
N
S
O
O
B(MIDA)
F_3C
N
N
2.00
2.01
1.02
3.06
1.17
1.13
4.22
1.00
2.38
1.06
1.06
3.06
1.01
3.23
2.06
3.01
8.0 7.5 7.0 6.5 6.0 5.5 5.0 4.5 4.0 3.5 3.0 2.5 2.0 1.5
f1 (ppm)

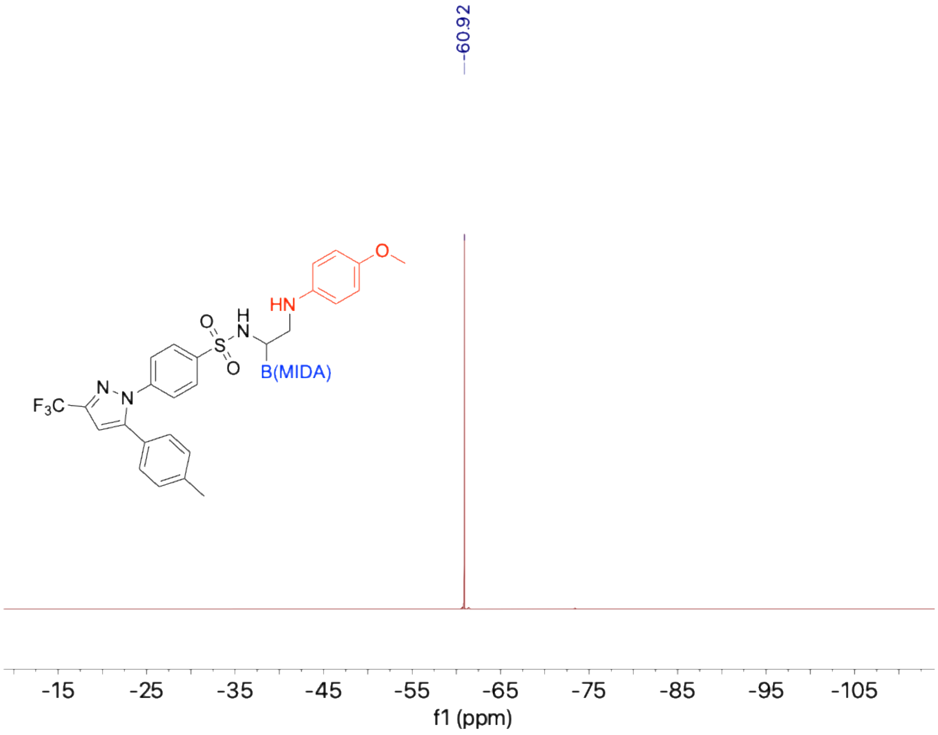
-60.92
HN
O
H
N
S
O
O
B(MIDA)
F_3C
N
N
-15 -25 -35 -45 -55 -65 -75 -85 -95 -105
f1 (ppm)

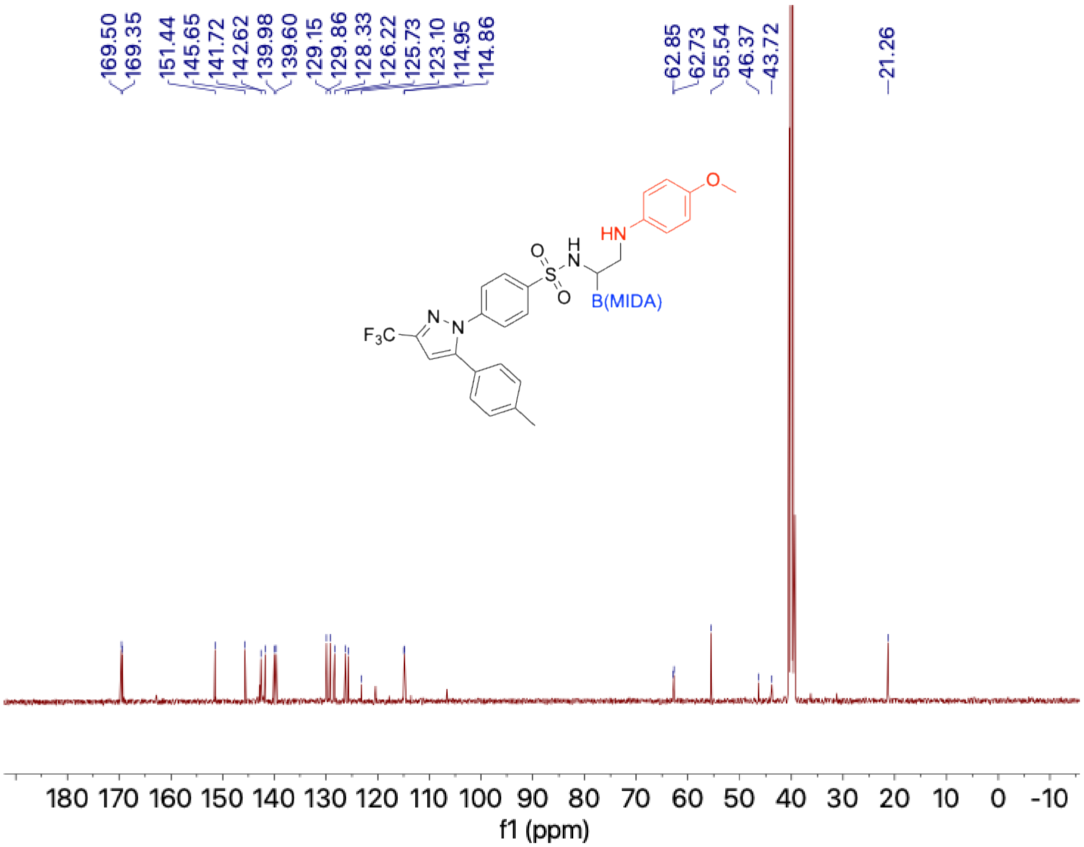
169.50
169.35
151.44
145.65
141.72
14262
139.98
139.60
129.15
129.86
128.33
126.22
125.73
123.10
114.95
114.86
62.85
62.73
55.54
46.37
43.72
21.26
O
HN
H
N
O
S
O
B(MIDA)
N
N
F3C
180 170 160 150 140 130 120 110 100 90 80 70 60 50 40 30 20 10 0 -10
f1 (ppm)

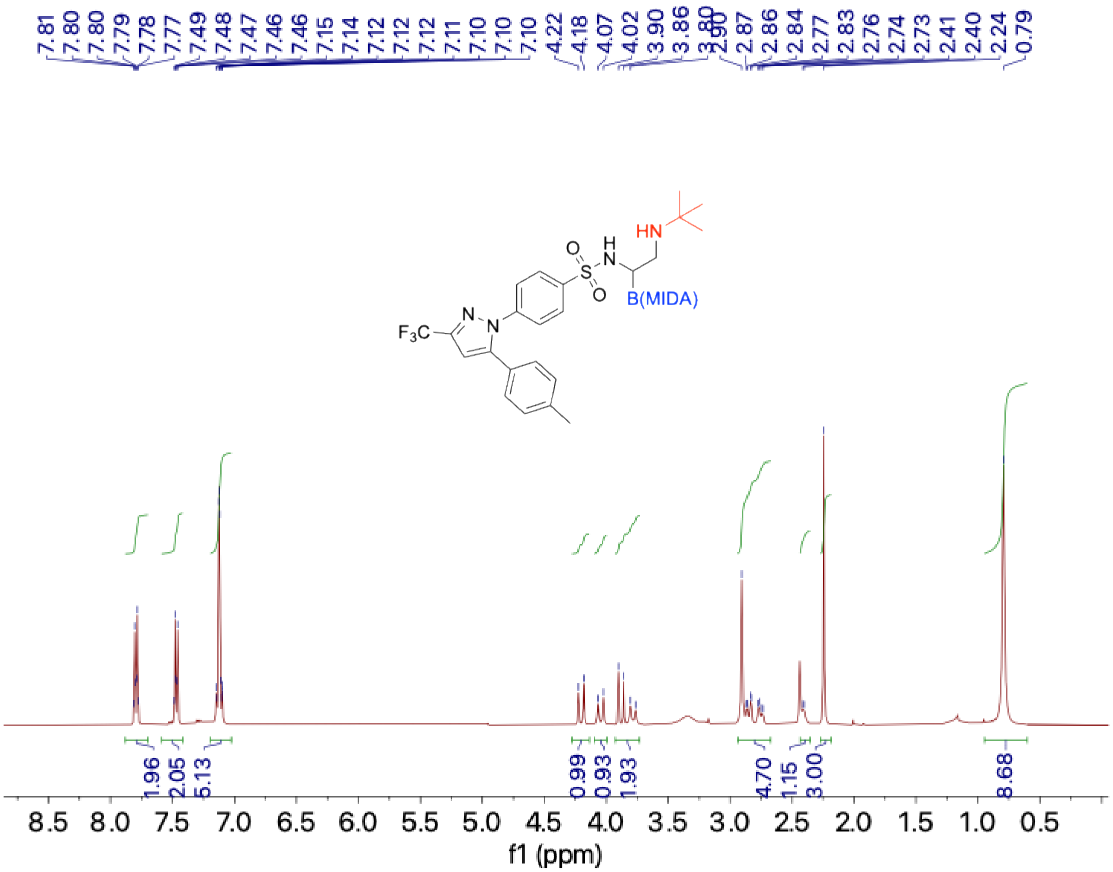

7.81 7.80 7.80 7.79 7.78 7.77 7.49 7.48 7.47 7.46 7.46 7.15 7.14 7.12 7.12 7.12 7.11 7.10 7.10 7.10 4.22 4.18 4.07 4.02 3.90 3.86 3.80 2.90 2.87 2.86 2.84 2.77 2.83 2.76 2.74 2.73 2.41 2.40 2.24 0.79
HN
H
N
O
S
O
B(MIDA)
N
N
F3C
1.96
2.05
5.13
0.99
0.93
1.93
4.70
1.15
3.00
8.68
8.5 8.0 7.5 7.0 6.5 6.0 5.5 5.0 4.5 4.0 3.5 3.0 2.5 2.0 1.5 1.0 0.5
f1 (ppm)

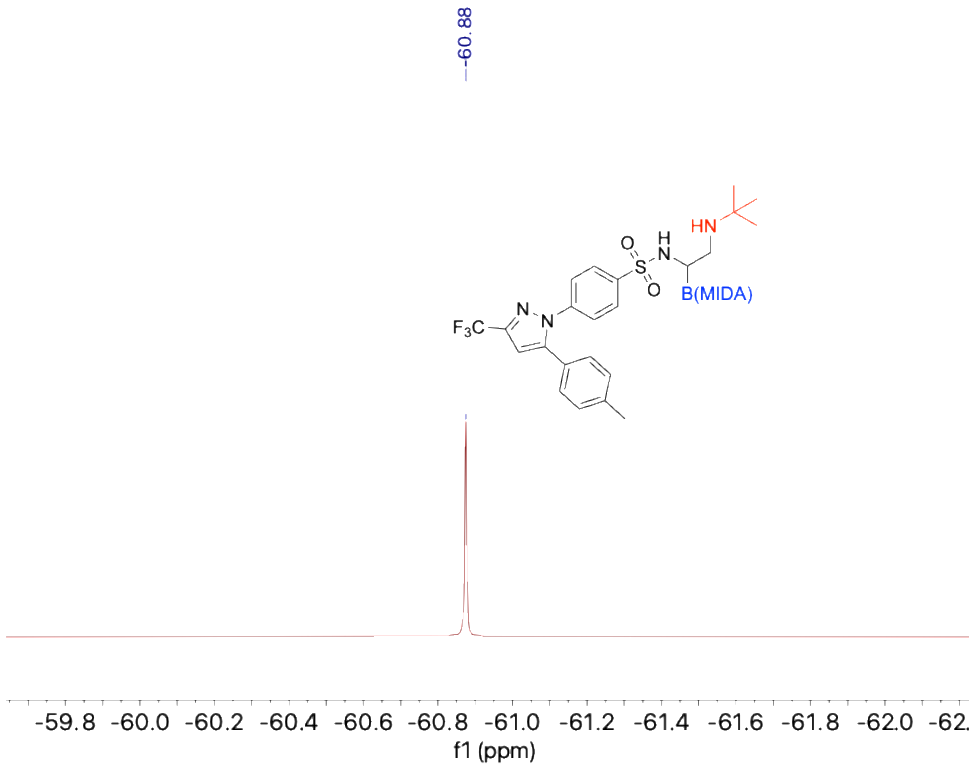

-60.88
HN
H
N
O
S
O
B(MIDA)
N
N
F3C
-59.8 -60.0 -60.2 -60.4 -60.6 -60.8 -61.0 -61.2 -61.4 -61.6 -61.8 -62.0 -62.
f1 (ppm)

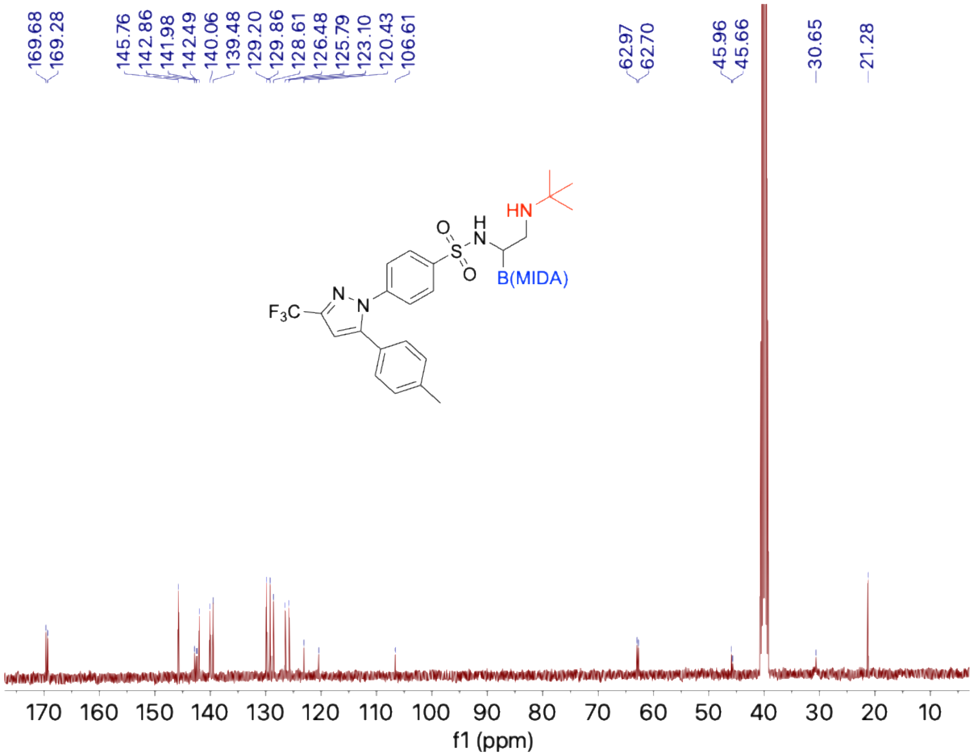
169.68
169.28
145.76
142.86
141.98
142.49
140.06
139.48
129.20
129.86
128.61
126.48
125.79
123.10
120.43
106.61
62.97
62.70
45.96
45.66
30.65
21.28
HN
H
N
O
S
O
B(MIDA)
N
N
F3C
170 160 150 140 130 120 110 100 90 80 70 60 50 40 30 20 10
f1 (ppm)

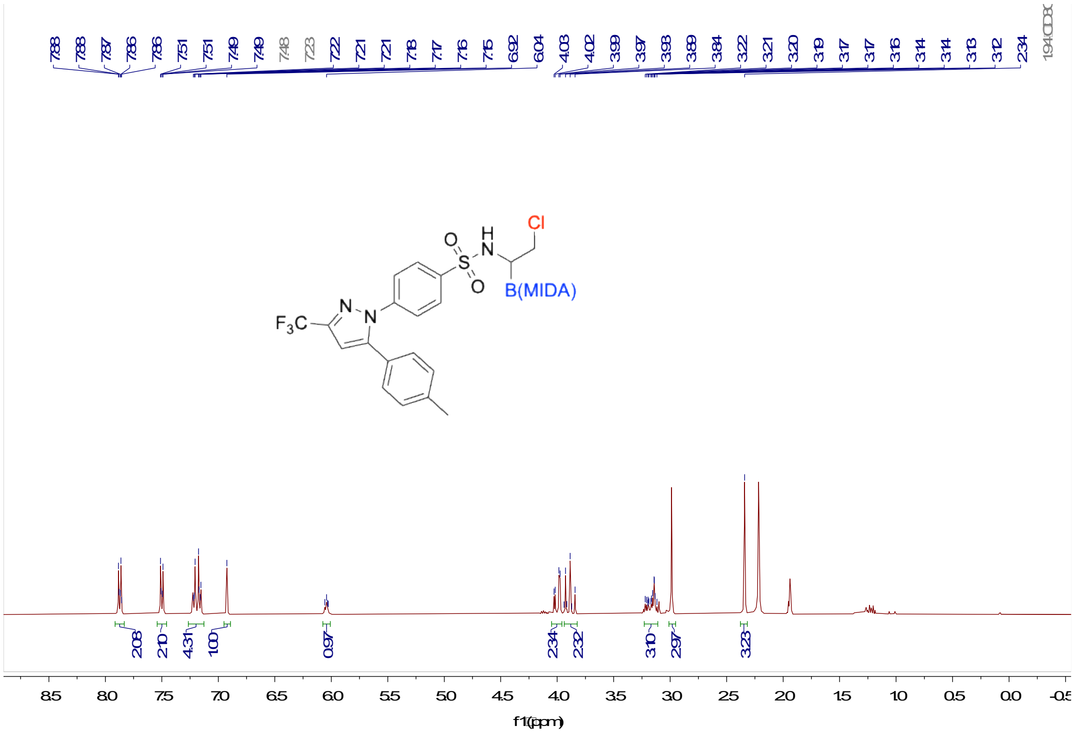

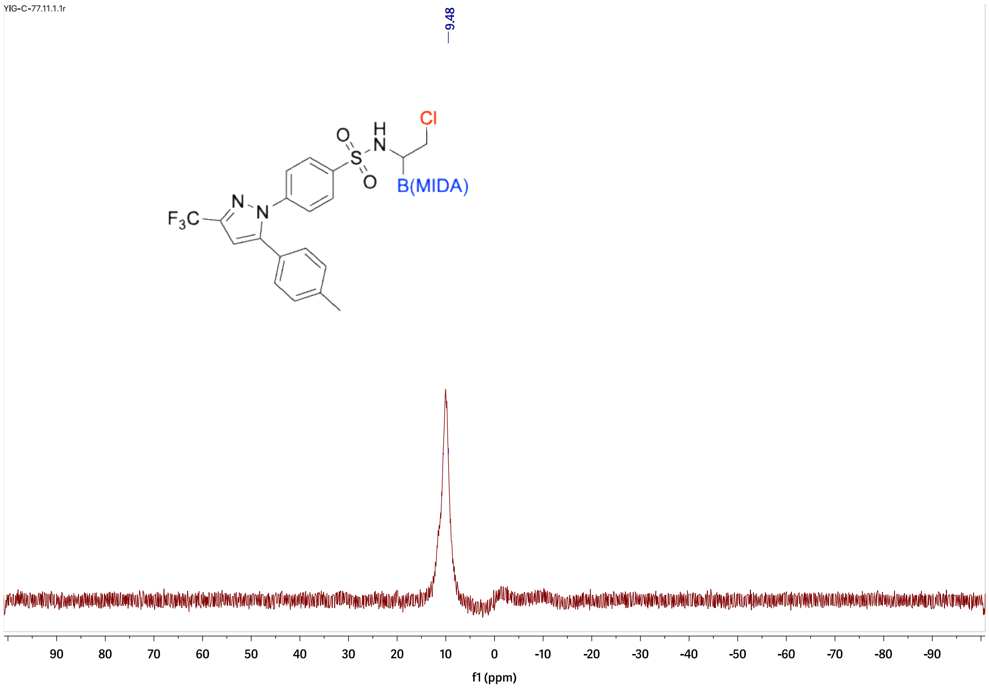

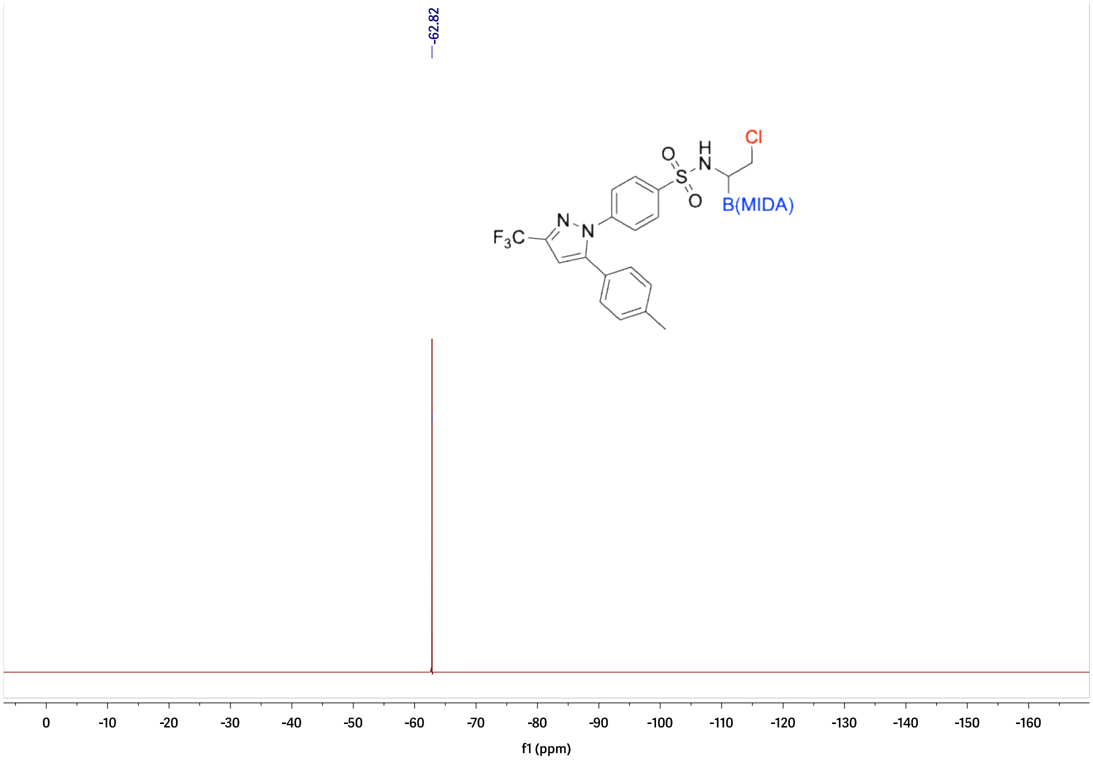

-62.82
Cl
O
H
N
S
O
B(MIDA)
N
N
F3C
0
-10
-20
-30
-40
-50
-60
-70
-80
-90
-100
-110
-120
-130
-140
-150
-160
f1 (ppm)

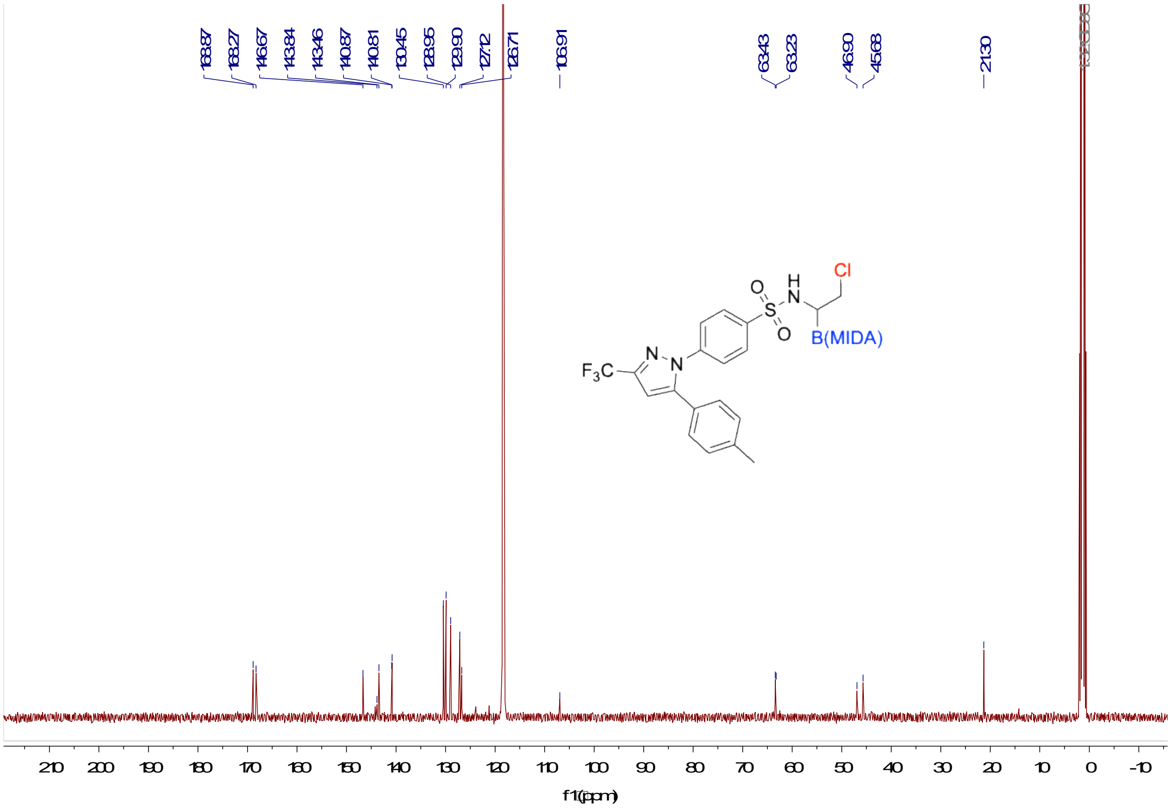

Cl
B(MIDA)
F3C
210
200
190
180
170
160
150
140
130
120
110
100
90
80
70
60
50
40
30
20
10
0
-10
f1 (ppm)

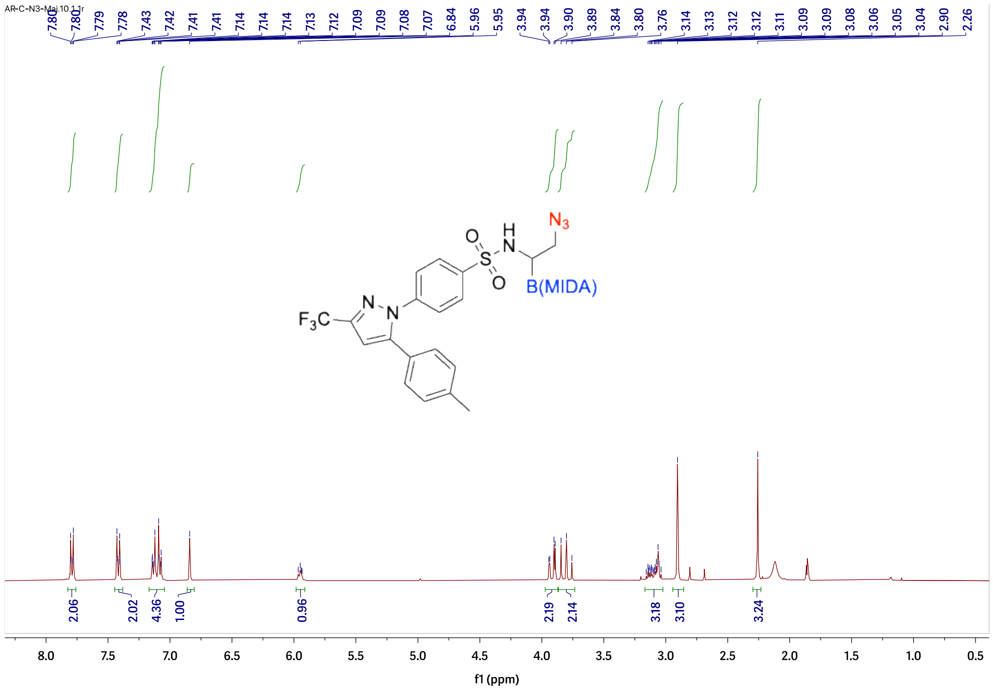
AR-C-N3-Maj.10.1.1r
7.80
7.80
7.79
7.78
7.43
7.42
7.41
7.41
7.14
7.14
7.14
7.13
7.12
7.09
7.09
7.08
7.07
6.84
5.96
5.95
3.94
3.94
3.90
3.89
3.84
3.80
3.76
3.14
3.13
3.12
3.12
3.11
3.09
3.09
3.08
3.06
3.05
3.04
2.90
2.26
N3
O
H
S
N
O
B(MIDA)
N
N
F3C
2.06
2.02
4.36
1.00
0.96
2.19
2.14
3.18
3.10
3.24
8.0
7.5
7.0
6.5
6.0
5.5
5.0
4.5
4.0
3.5
3.0
2.5
2.0
1.5
1.0
0.5
f1 (ppm)

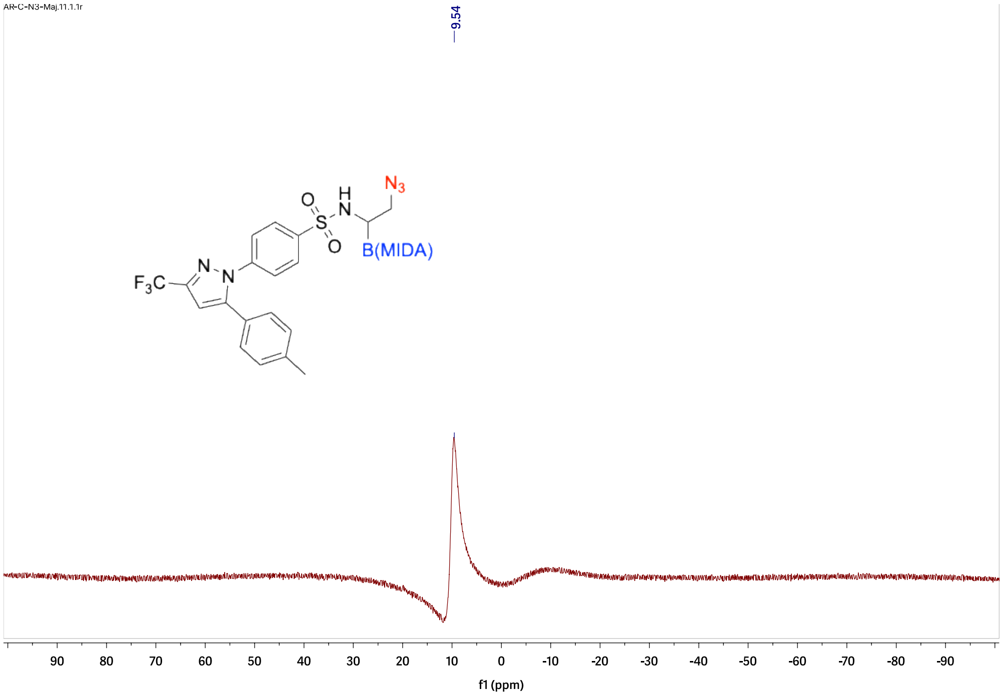
AR-C-N3-Maj.11.1.1r
9.54
N3
O
H
S
N
O
B(MIDA)
N
N
F3C
90
80
70
60
50
40
30
20
10
0
-10
-20
-30
-40
-50
-60
-70
-80
-90
f1 (ppm)

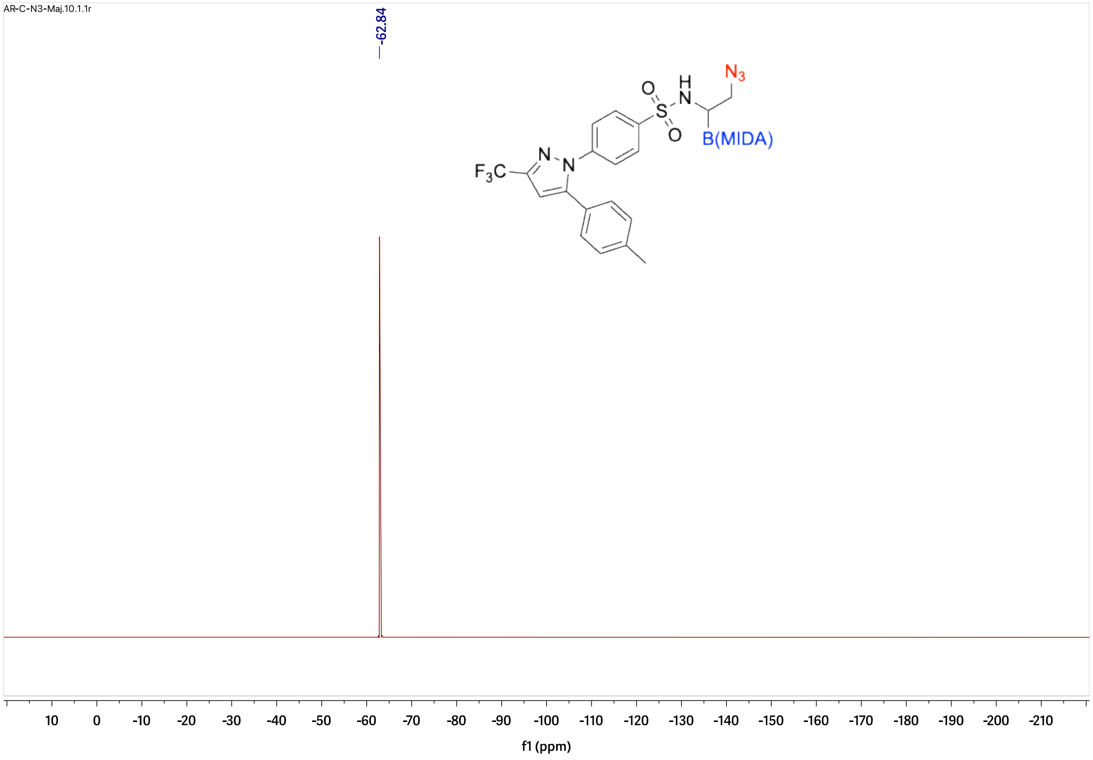
AR-C-N3-Maj.10.1.1r
-62.84
N3
O
H
N
S
O
B(MIDA)
N
N
F3C
10 0 -10 -20 -30 -40 -50 -60 -70 -80 -90 -100 -110 -120 -130 -140 -150 -160 -170 -180 -190 -200 -210
f1 (ppm)

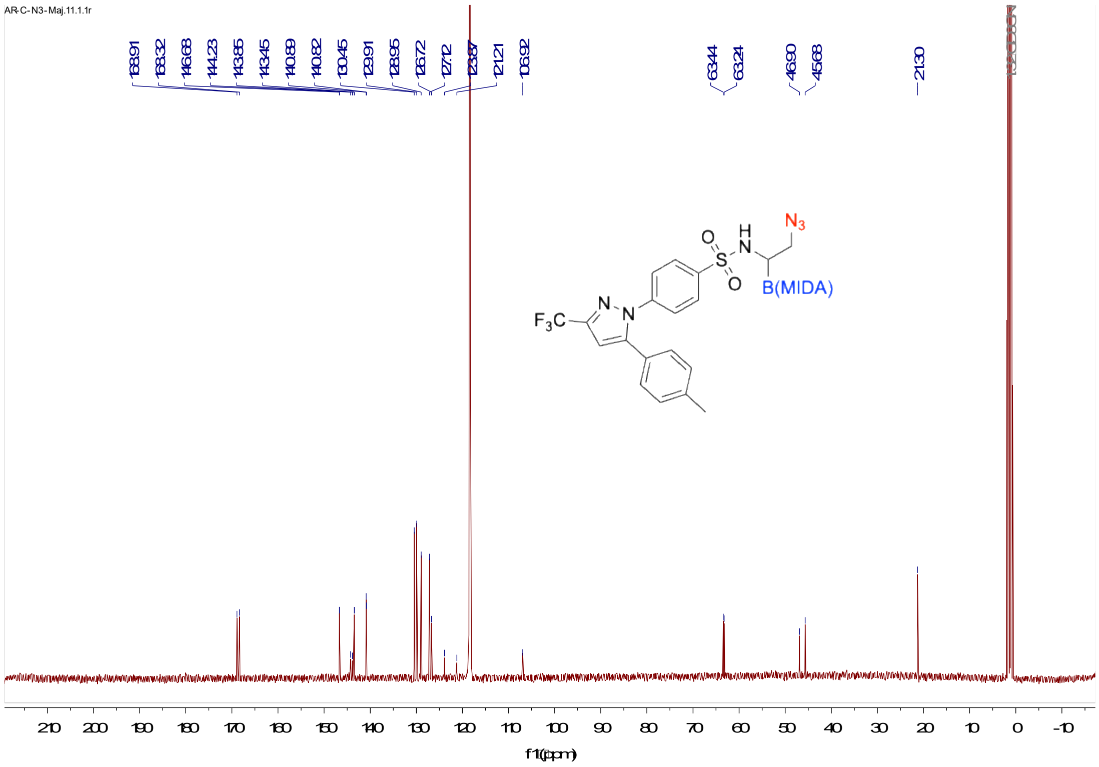
AR-C-N3-Maj.11.1.1r
168.91
168.32
146.68
144.23
143.85
143.45
140.89
140.82
130.45
129.91
128.95
126.72
123.87
121.21
106.92
63.44
63.24
46.90
45.68
21.30
N3
O
H
N
S
O
B(MIDA)
N
N
F3C
210 200 190 180 170 160 150 140 130 120 110 100 90 80 70 60 50 40 30 20 10 0 -10
f1 (ppm)

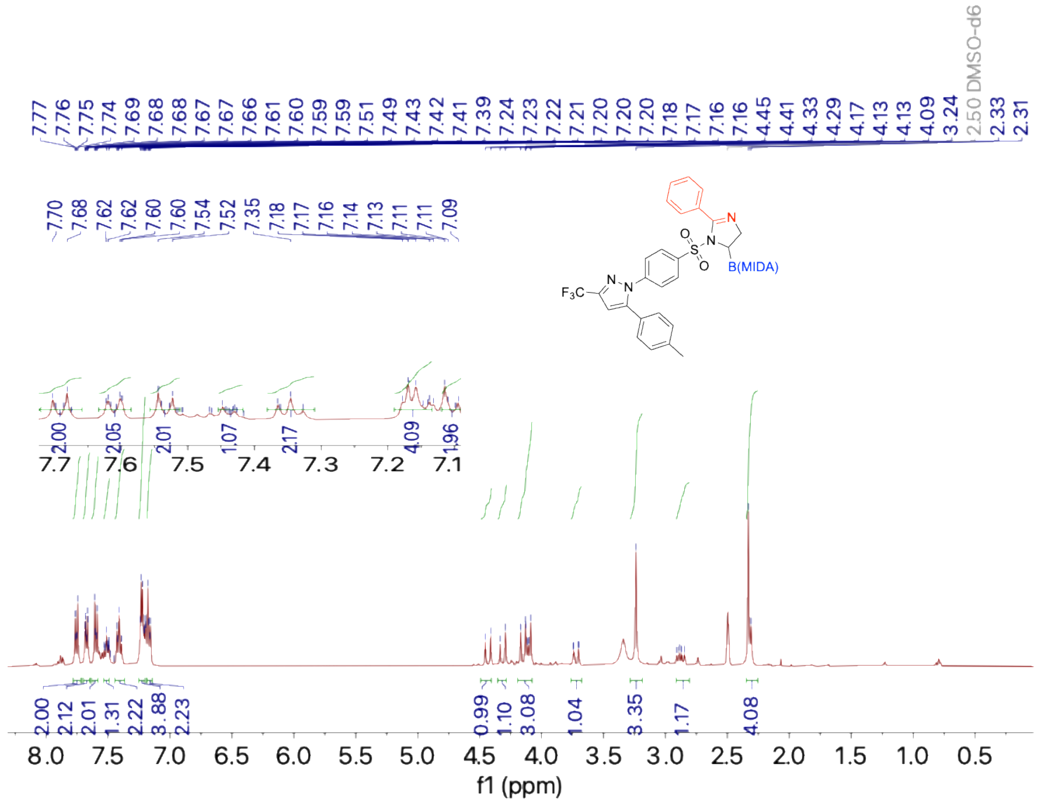

VB-F-182-PHCN-2.11.fid

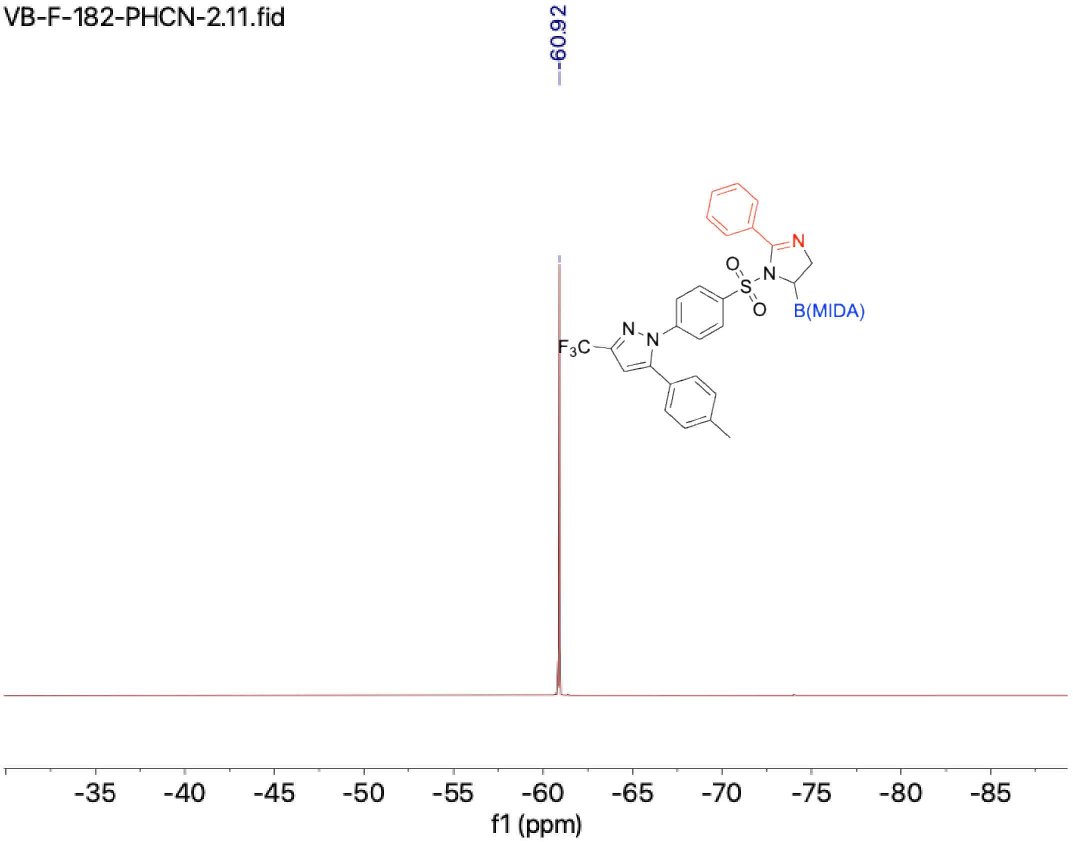

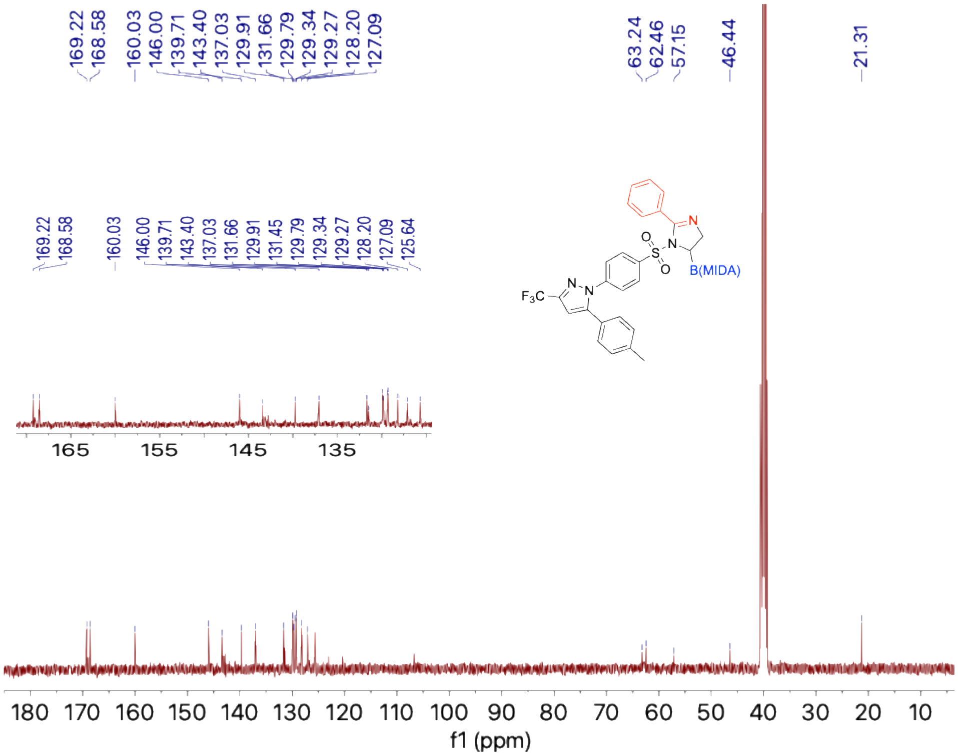
169.22
168.58
160.03
146.00
139.71
143.40
137.03
129.91
131.66
129.79
129.34
129.27
128.20
127.09
63.24
62.46
57.15
46.44
21.31
169.22
168.58
160.03
146.00
139.71
143.40
137.03
131.66
129.91
131.45
129.79
129.34
129.27
128.20
127.09
125.64
165
155
145
135
B(MIDA)
F3C
180 170 160 150 140 130 120 110 100 90 80 70 60 50 40 30 20 10
f1 (ppm)

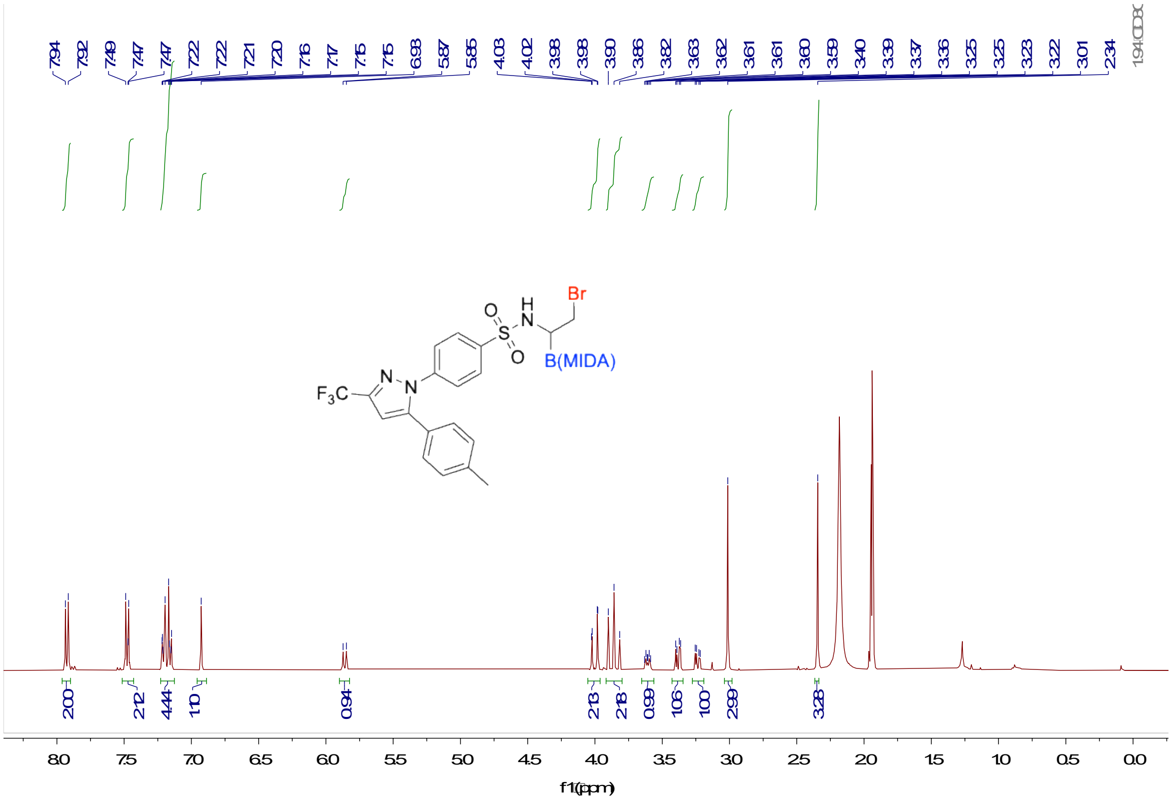

AR-C-72-U2.11.1.1r

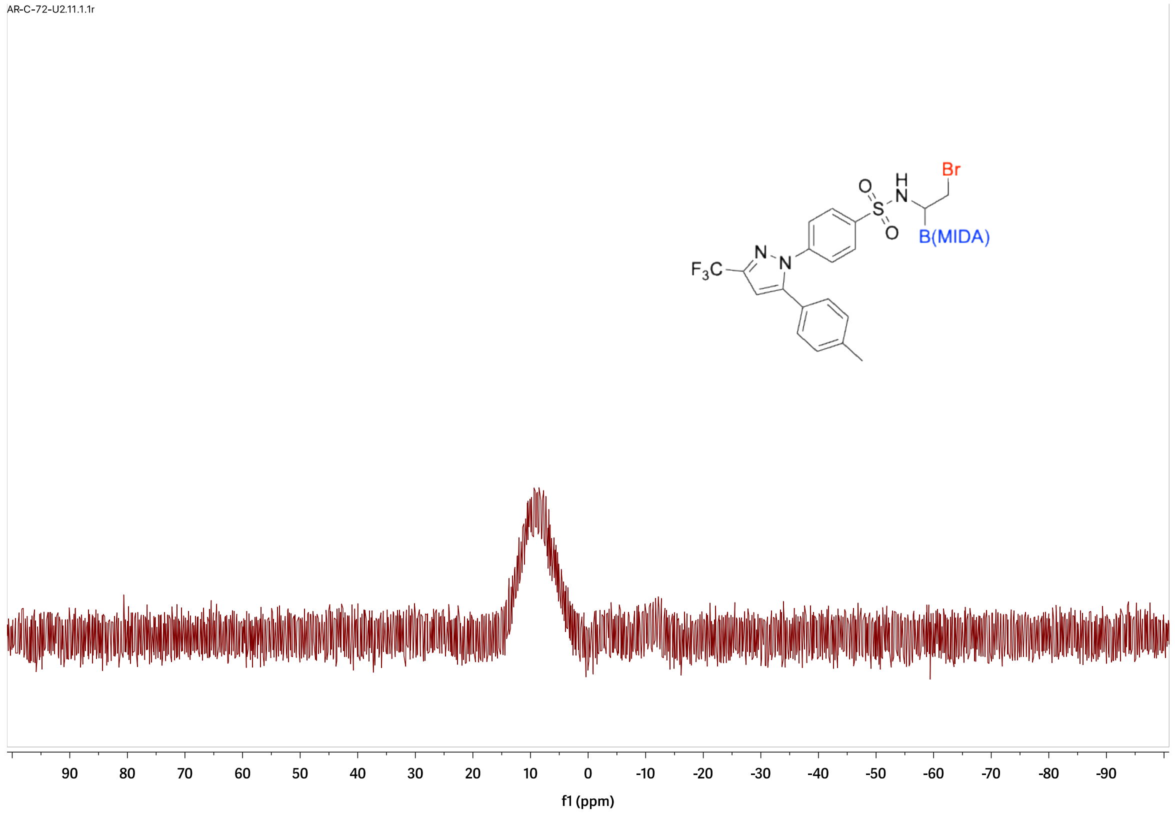

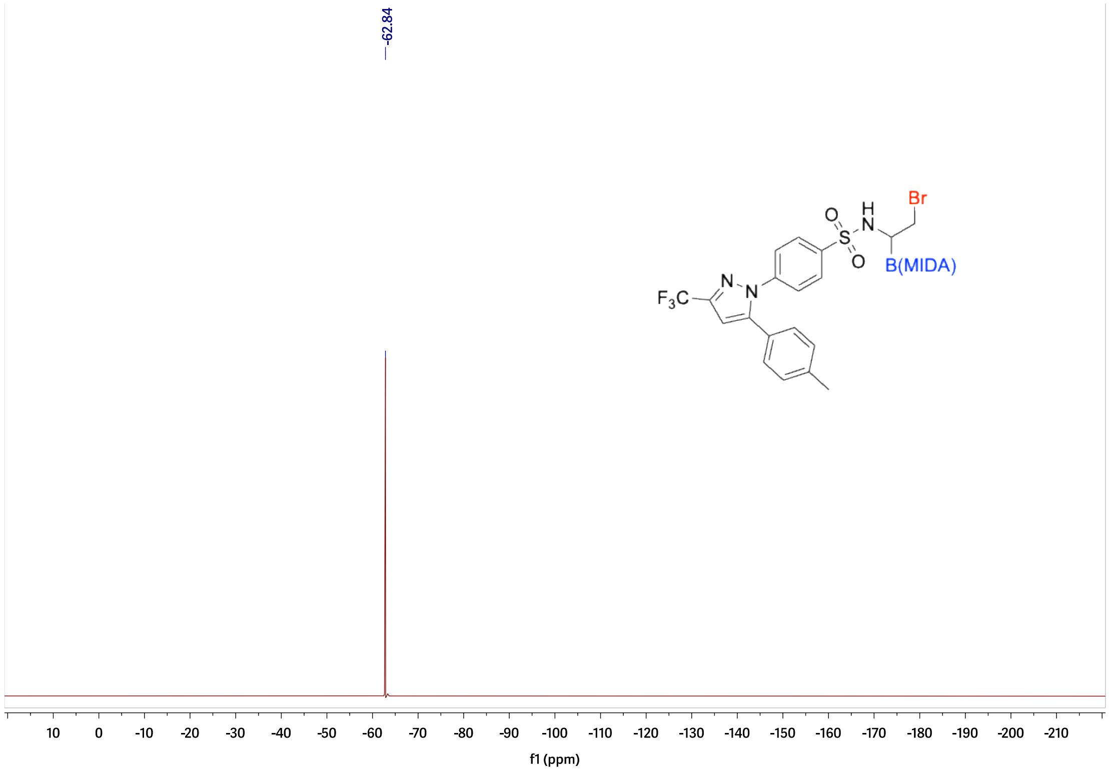
-62.84
Br
O
H
N
S
O
B(MIDA)
F3C
N
N
10 0 -10 -20 -30 -40 -50 -60 -70 -80 -90 -100 -110 -120 -130 -140 -150 -160 -170 -180 -190 -200 -210
f1 (ppm)

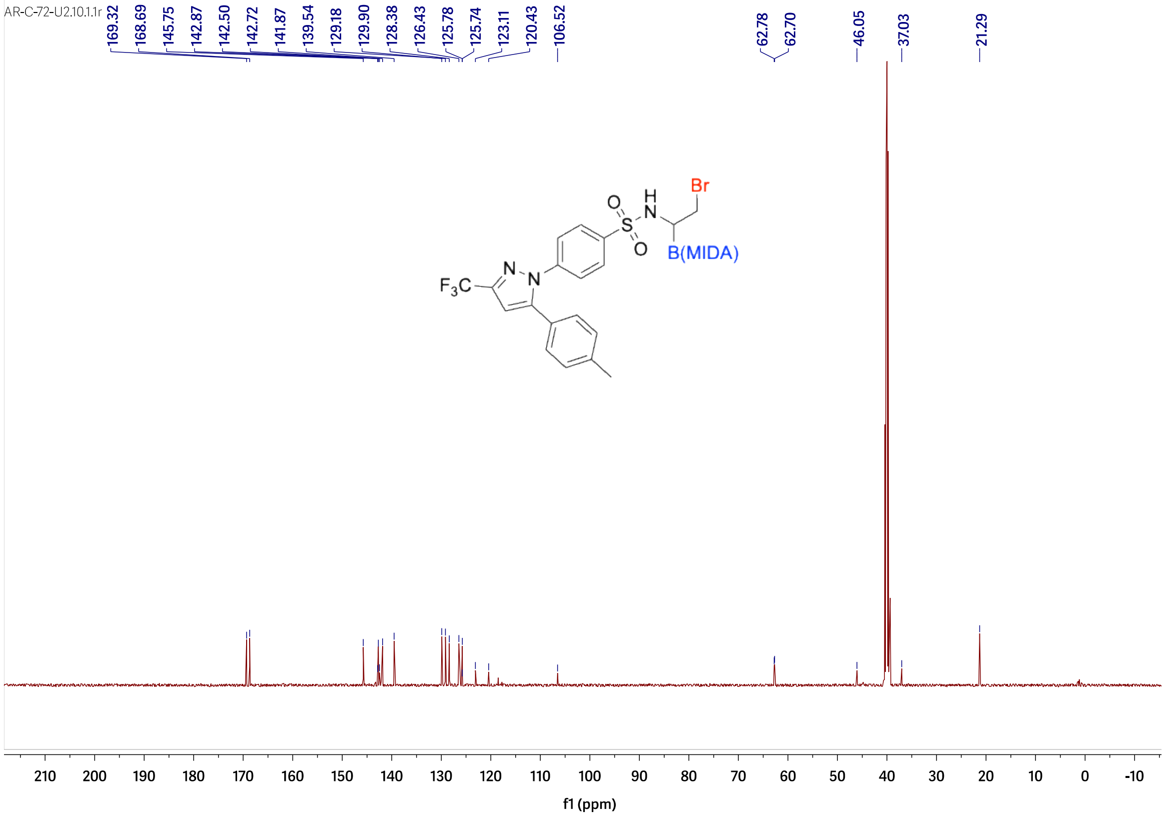
AR-C-72-U2.10.1.1r
169.32
168.69
145.75
142.87
142.50
142.72
141.87
139.54
129.18
129.90
128.38
126.43
125.78
125.74
123.11
120.43
106.52
62.78
62.70
46.05
37.03
21.29
Br
O
H
N
S
O
B(MIDA)
F3C
N
N
210 200 190 180 170 160 150 140 130 120 110 100 90 80 70 60 50 40 30 20 10 0 -10
f1 (ppm)

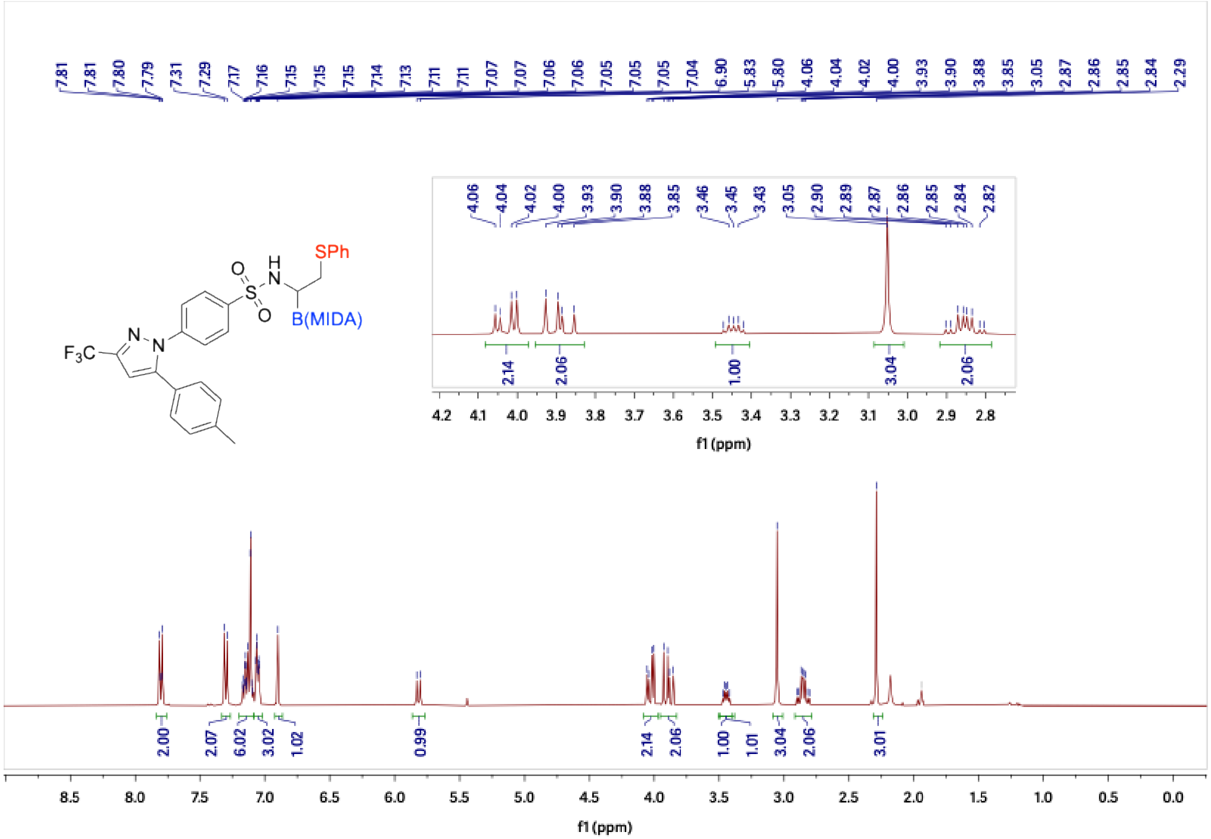

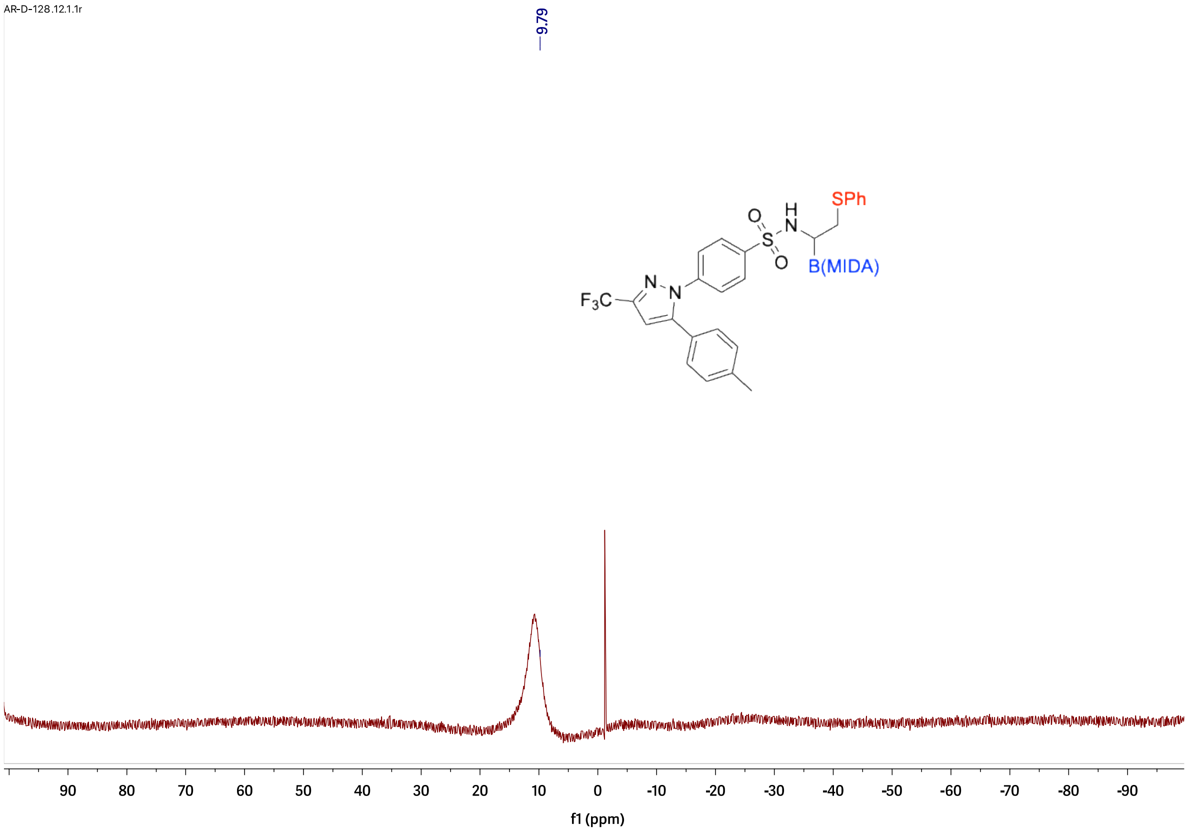

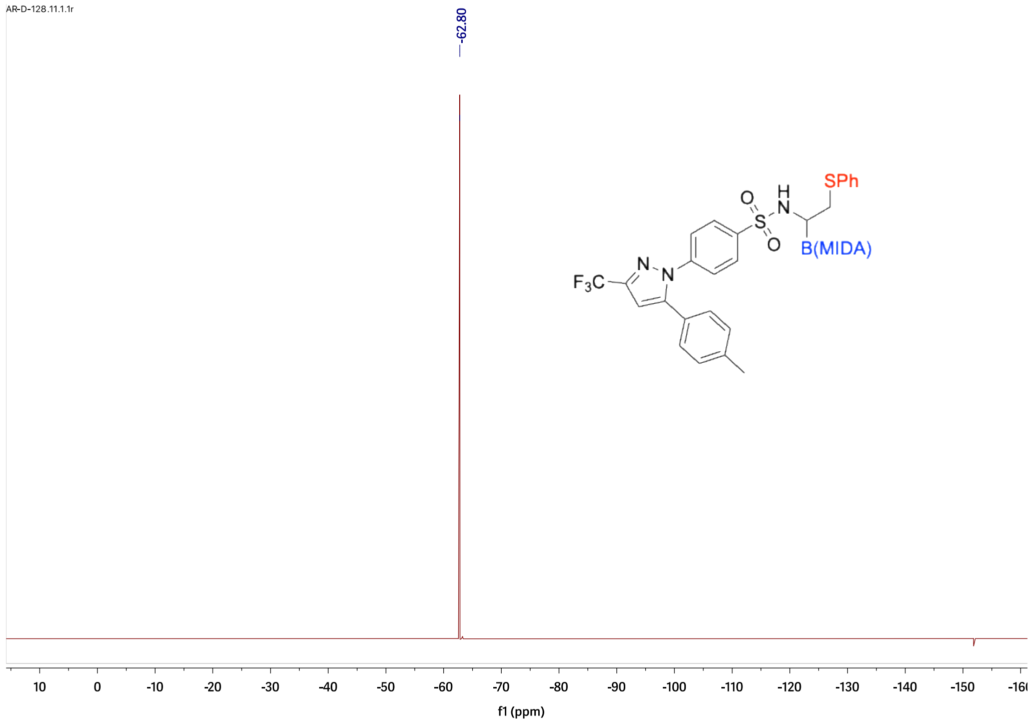
AR-D-128.11.1.1r
-62.80
SPh
B(MIDA)
F_3C
f1 (ppm)

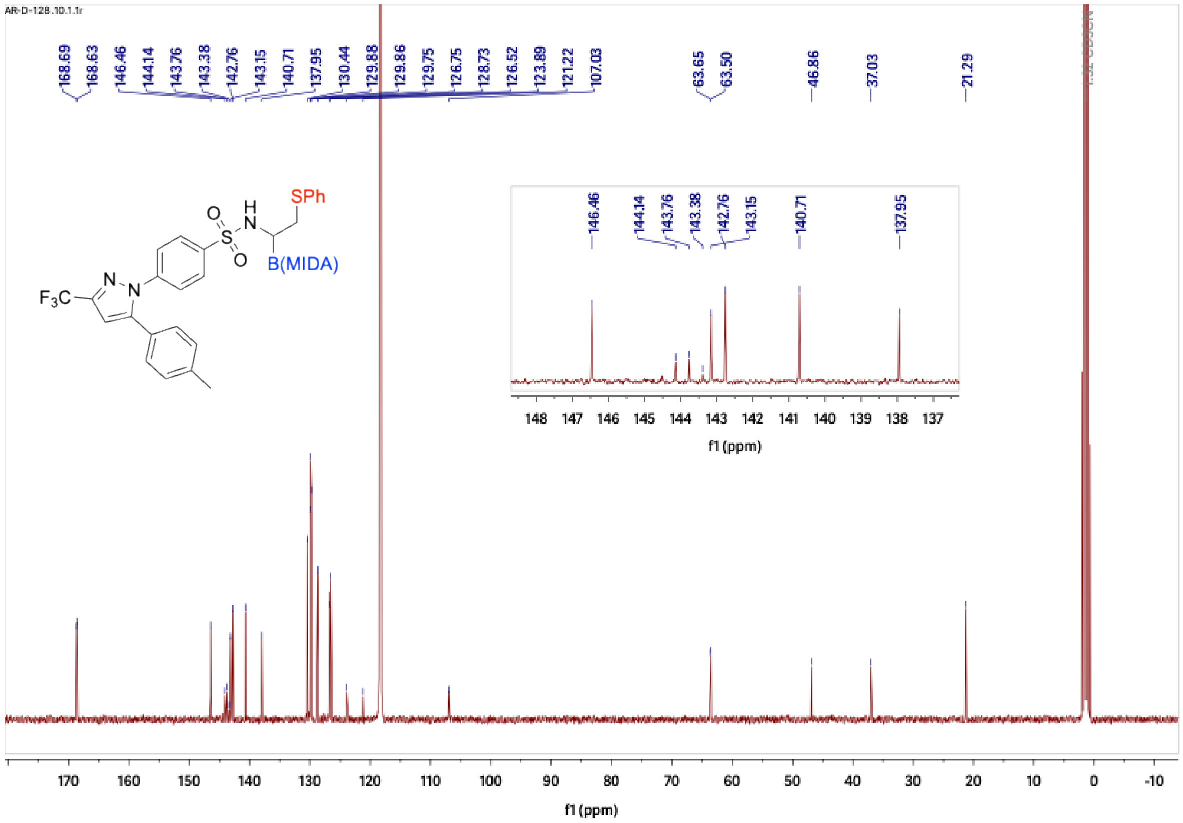
AR-D-128.10.1.1r
168.69
168.63
146.46
144.14
143.76
143.38
142.76
143.15
140.71
137.95
130.44
129.88
129.86
129.75
126.75
128.73
126.52
123.89
121.22
107.03
63.65
63.50
46.86
37.03
21.29
SPh
B(MIDA)
F_3C
f1 (ppm)

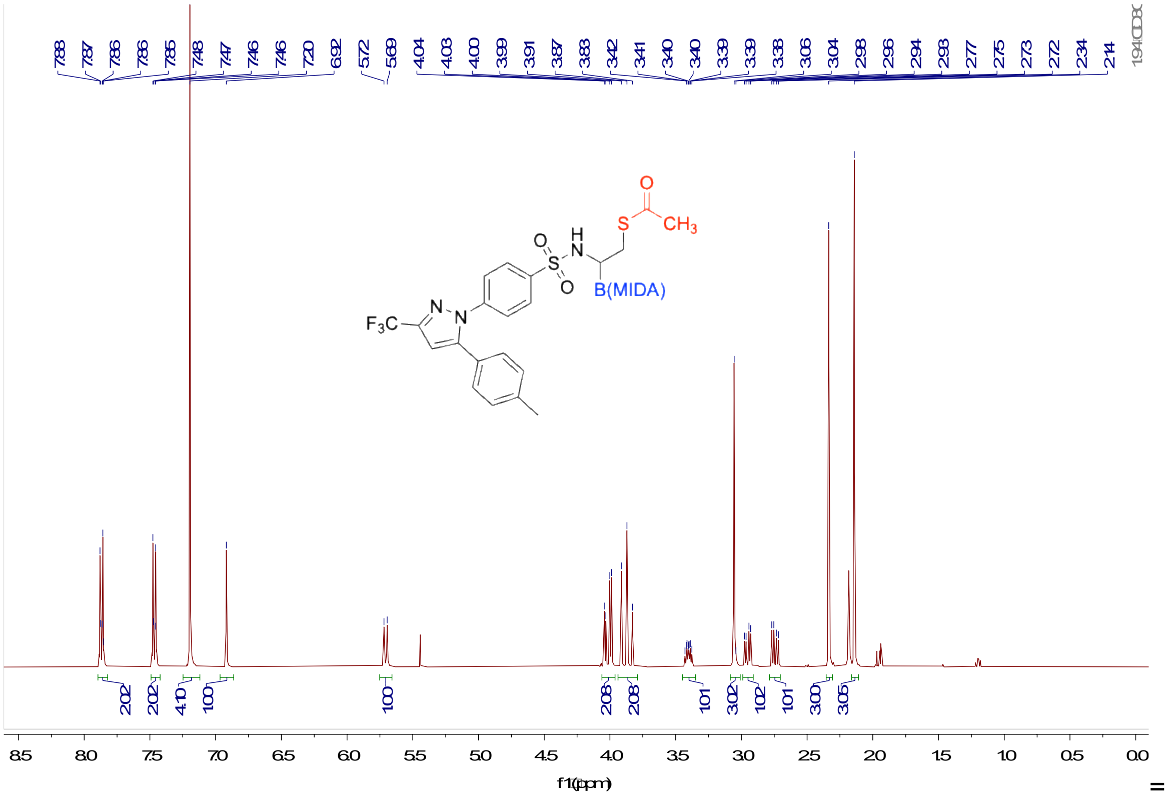

F3C
N
N
S
O
O
H
N
B(MIDA)
S
O
CH3
f1 (ppm)

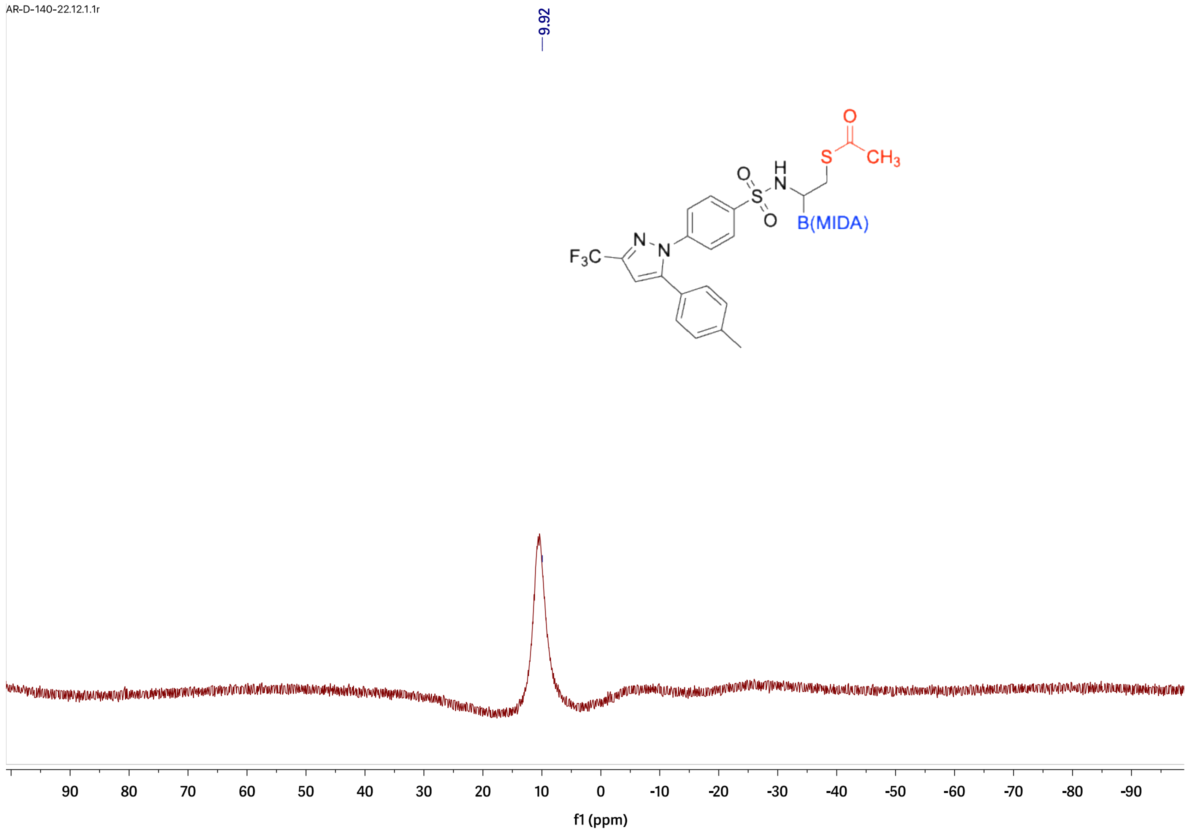
AR-D-140-22.12.1.1r
9.92
B(MIDA)
f1 (ppm)

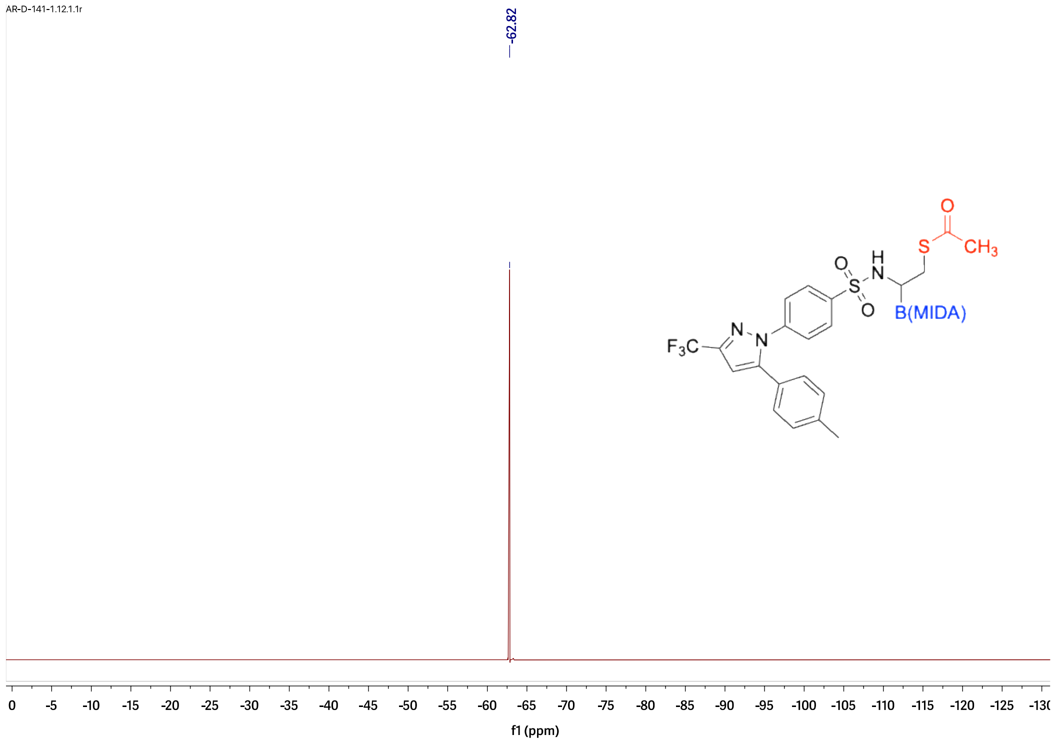
AR-D-141-1.12.1.1r
-62.82
B(MIDA)
f1 (ppm)

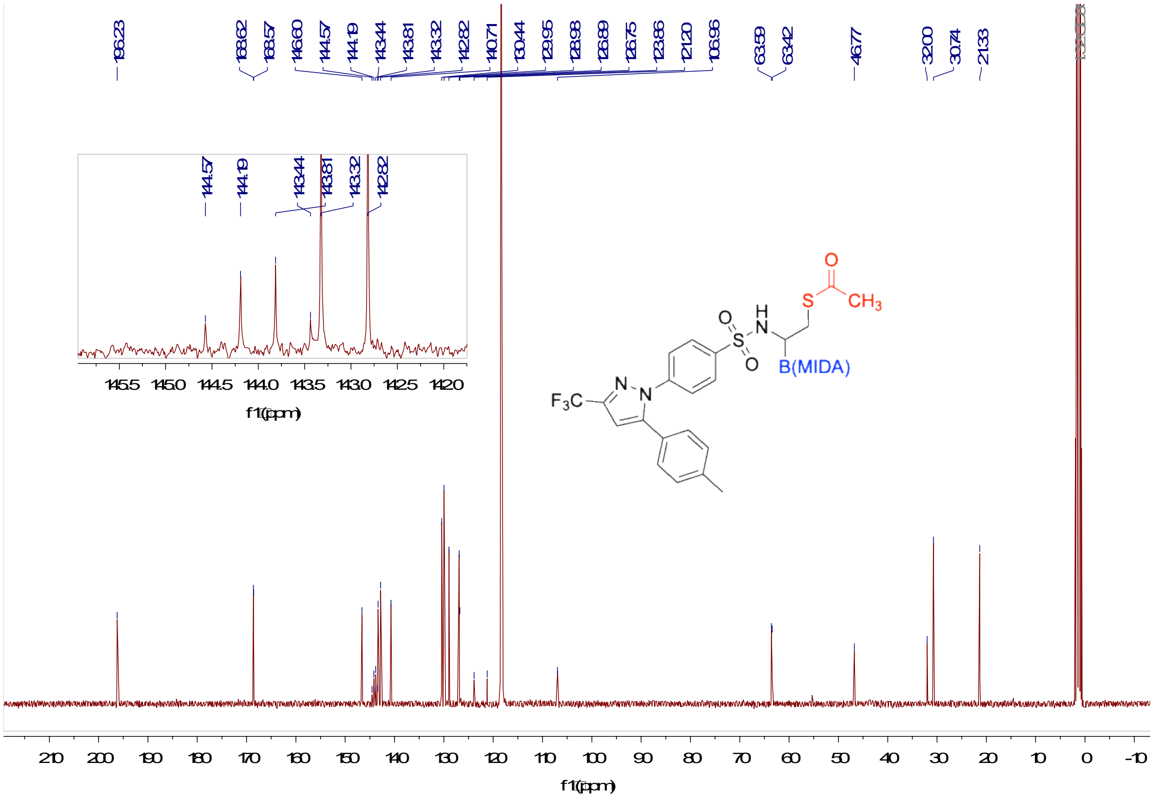

145.5 145.0 144.5 144.0 143.5 143.0 142.5 142.0
f1 (ppm)
O
S
CH3
O
H
N
S
O
N
N
F3C
B(MIDA)
210 200 190 180 170 160 150 140 130 120 110 100 90 80 70 60 50 40 30 20 10 0 -10
f1 (ppm)

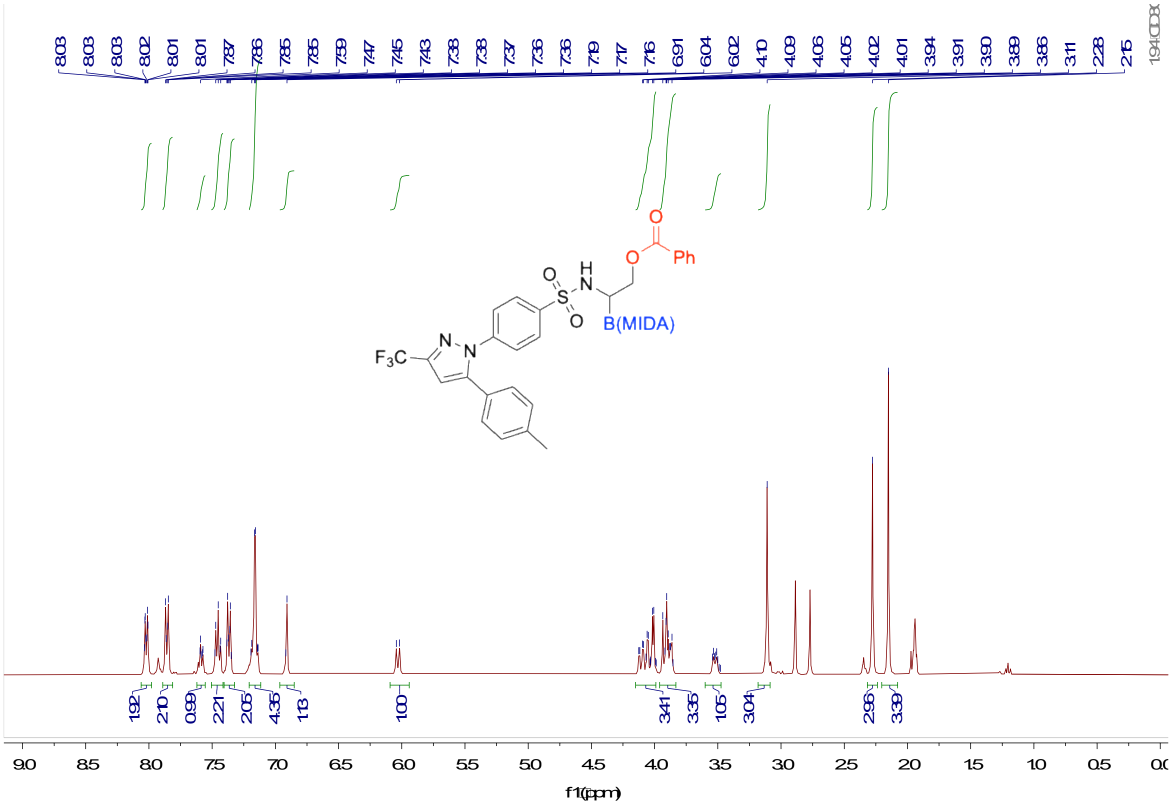

O
O
Ph
O
H
N
S
O
N
N
F3C
B(MIDA)
9.0 8.5 8.0 7.5 7.0 6.5 6.0 5.5 5.0 4.5 4.0 3.5 3.0 2.5 2.0 1.5 1.0 0.5 0.0
f1 (ppm)

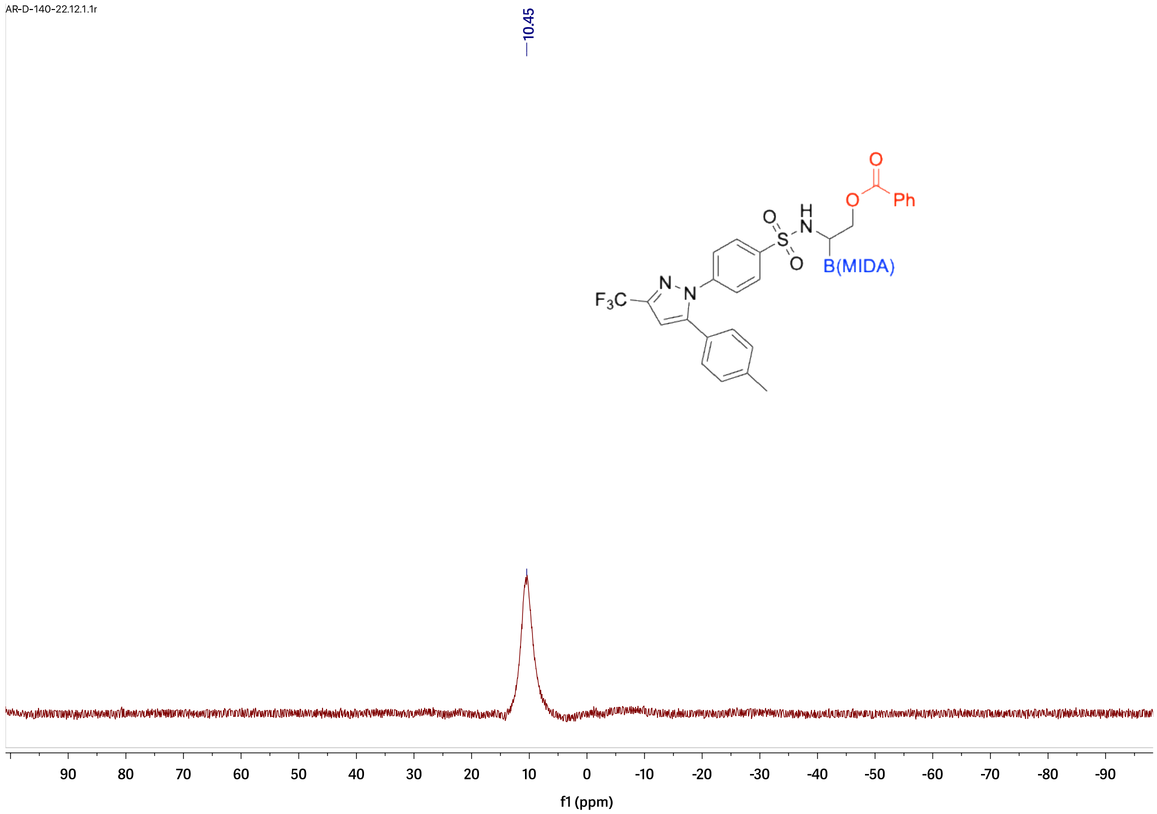
AR-D-140-22.12.1.1r
10.45
F3C
N
N
O
S
O
H
N
O
O
Ph
B(MIDA)
90
80
70
60
50
40
30
20
10
0
-10
-20
-30
-40
-50
-60
-70
-80
-90
f1 (ppm)

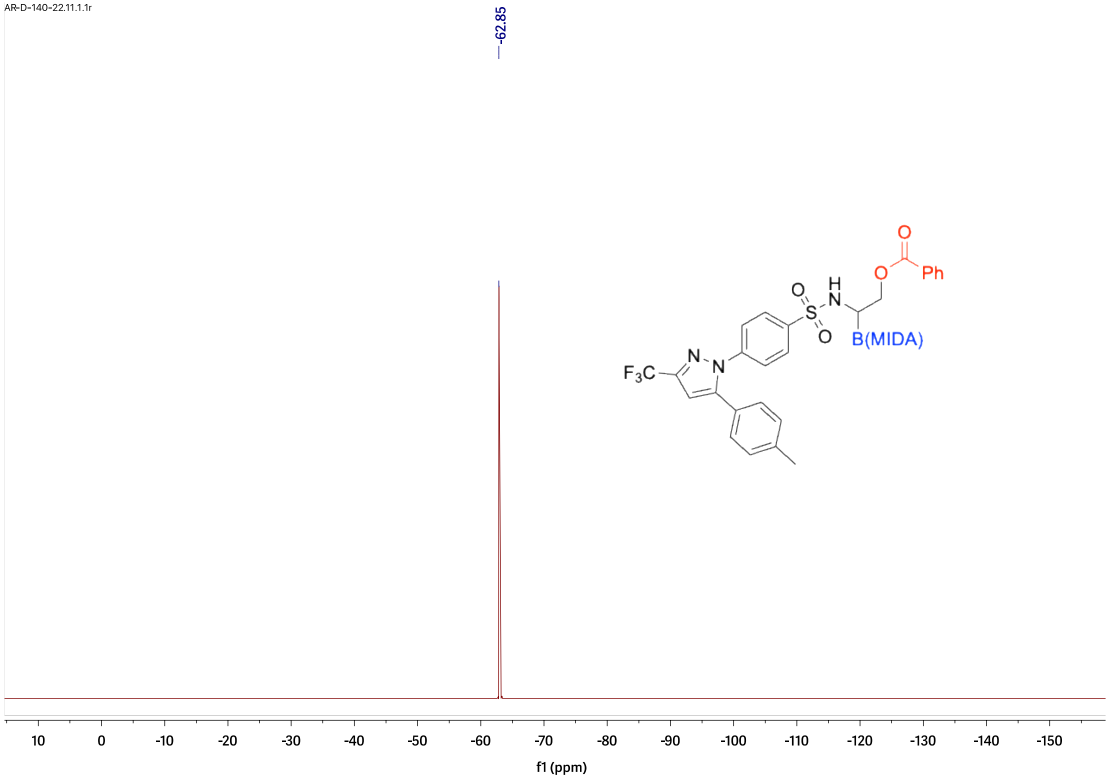
AR-D-140-22.11.1.1r
-62.85
F3C
N
N
O
S
O
H
N
O
O
Ph
B(MIDA)
10
0
-10
-20
-30
-40
-50
-60
-70
-80
-90
-100
-110
-120
-130
-140
-150
f1 (ppm)

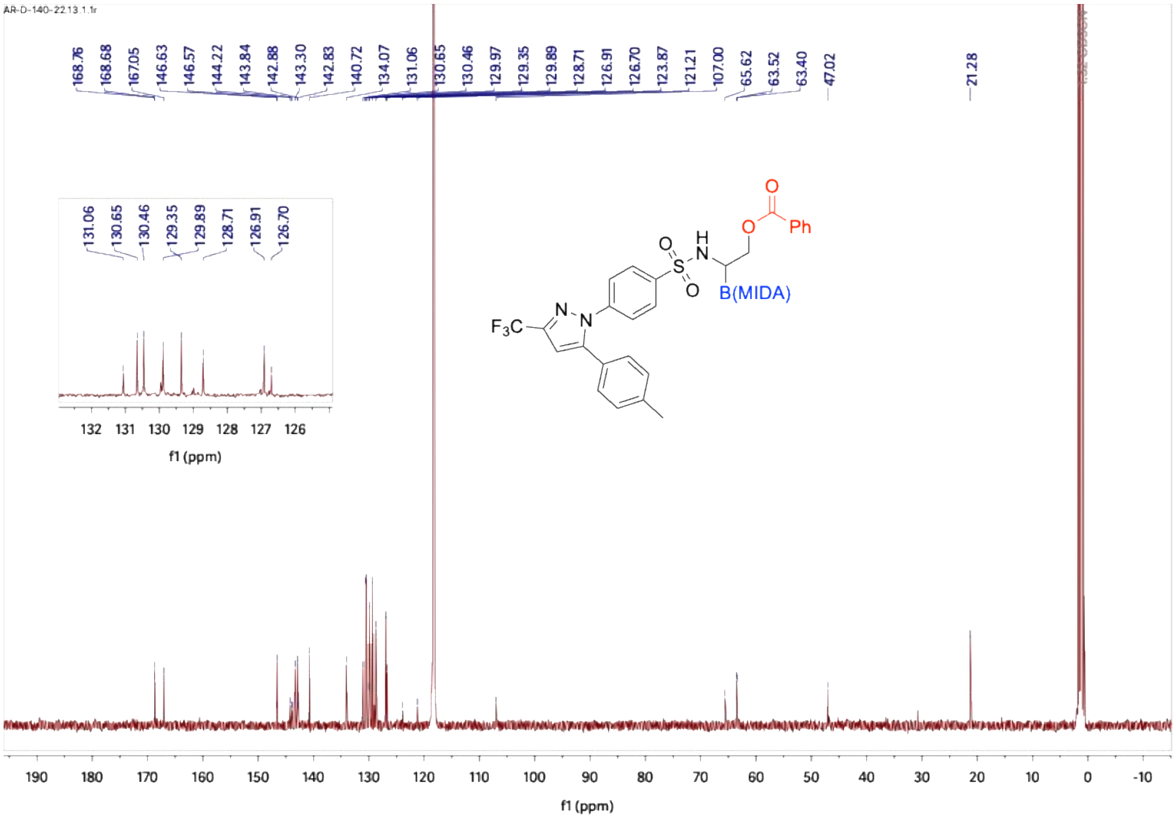
AR-D-140-22.13.1.1r
168.76
168.68
167.05
146.63
146.57
144.22
143.84
142.88
143.30
142.83
140.72
134.07
131.06
130.65
130.46
129.97
129.35
129.89
128.71
126.91
126.70
123.87
121.21
107.00
65.62
63.52
63.40
47.02
21.28
O
Ph
O
H
N
S
B(MIDA)
N
F3C
190 180 170 160 150 140 130 120 110 100 90 80 70 60 50 40 30 20 10 0 -10
f1 (ppm)
132 131 130 129 128 127 126
f1 (ppm)

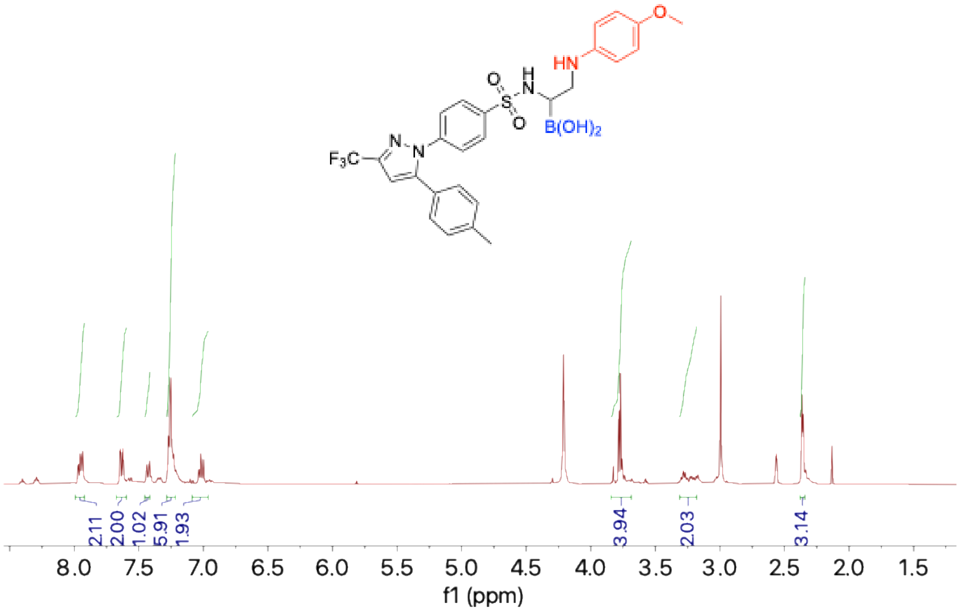
HN
O
H
N
S
B(OH)2
N
F3C
2.11
2.00
1.02
5.91
1.93
3.94
2.03
3.14
8.0 7.5 7.0 6.5 6.0 5.5 5.0 4.5 4.0 3.5 3.0 2.5 2.0 1.5
f1 (ppm)

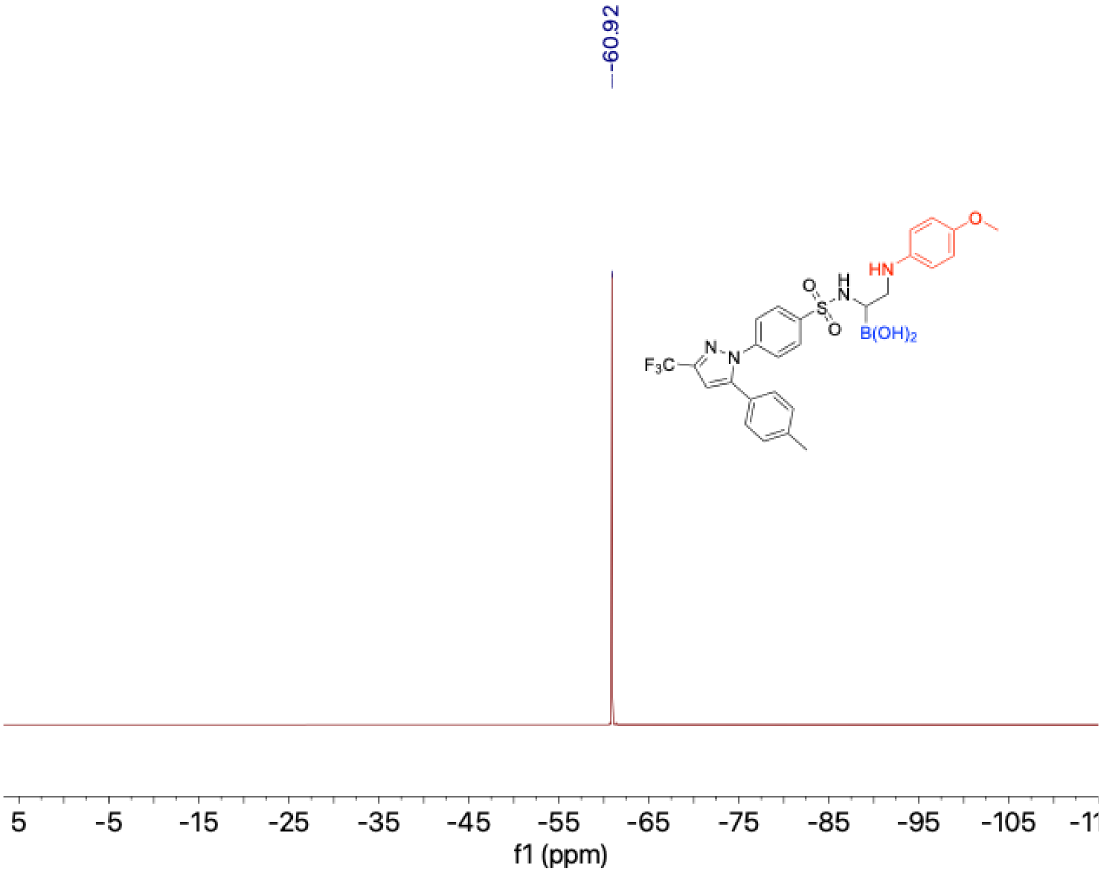

-60.92
HN
O
N
H
O
S
O
B(OH)2
N
N
F3C
5
-5
-15
-25
-35
-45
-55
-65
-75
-85
-95
-105
f1 (ppm)

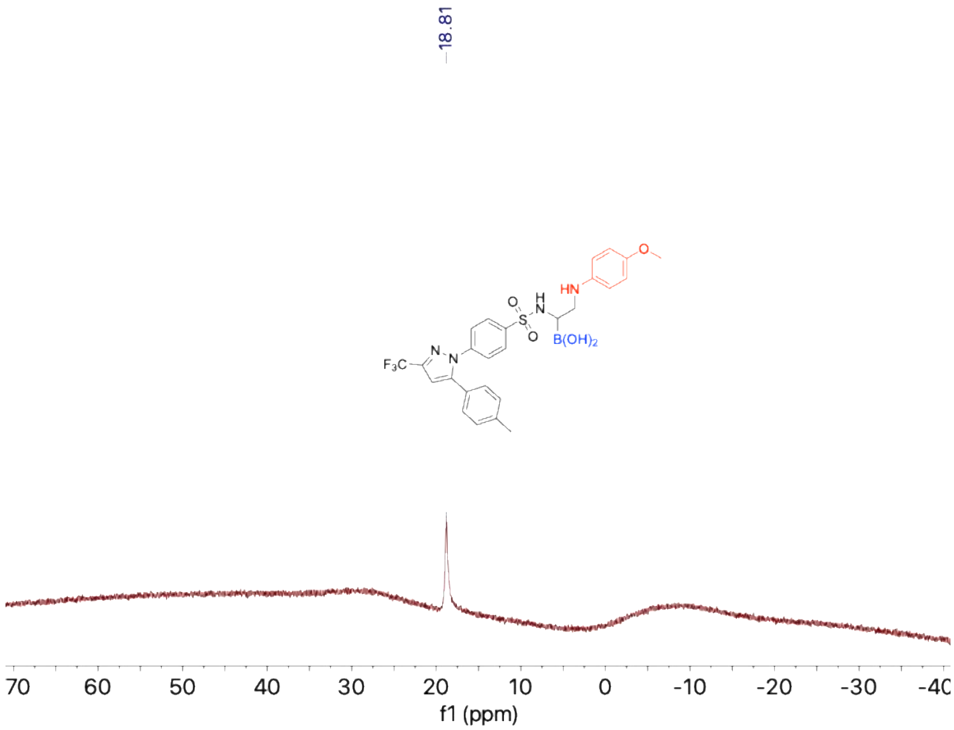

18.81
HN
O
N
H
O
S
O
B(OH)2
N
N
F3C
70
60
50
40
30
20
10
0
-10
-20
-30
f1 (ppm)

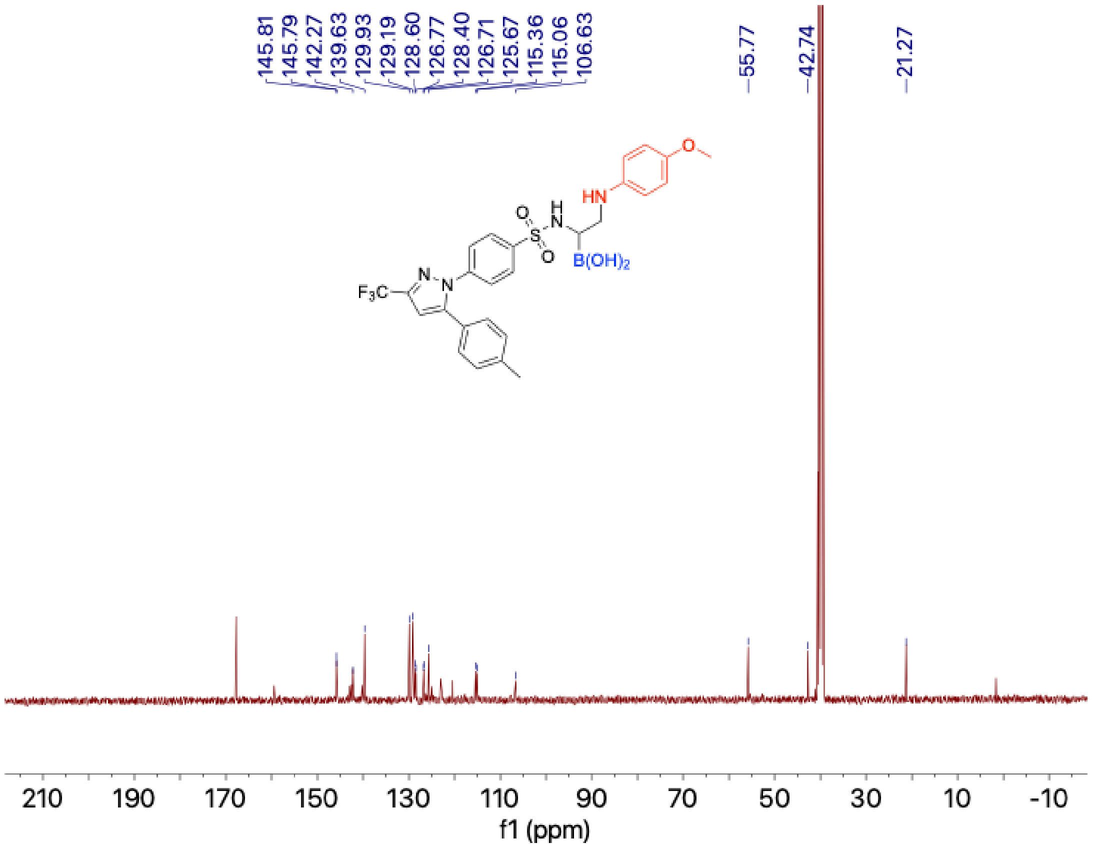

145.81
145.79
142.27
139.63
129.93
129.19
128.60
126.77
128.40
126.71
125.67
115.36
115.06
106.63
55.77
42.74
21.27
HN
O
H
N
S
B(OH)2
N
N
F3C
210
190
170
150
130
110
90
70
50
30
10
-10
f1 (ppm)

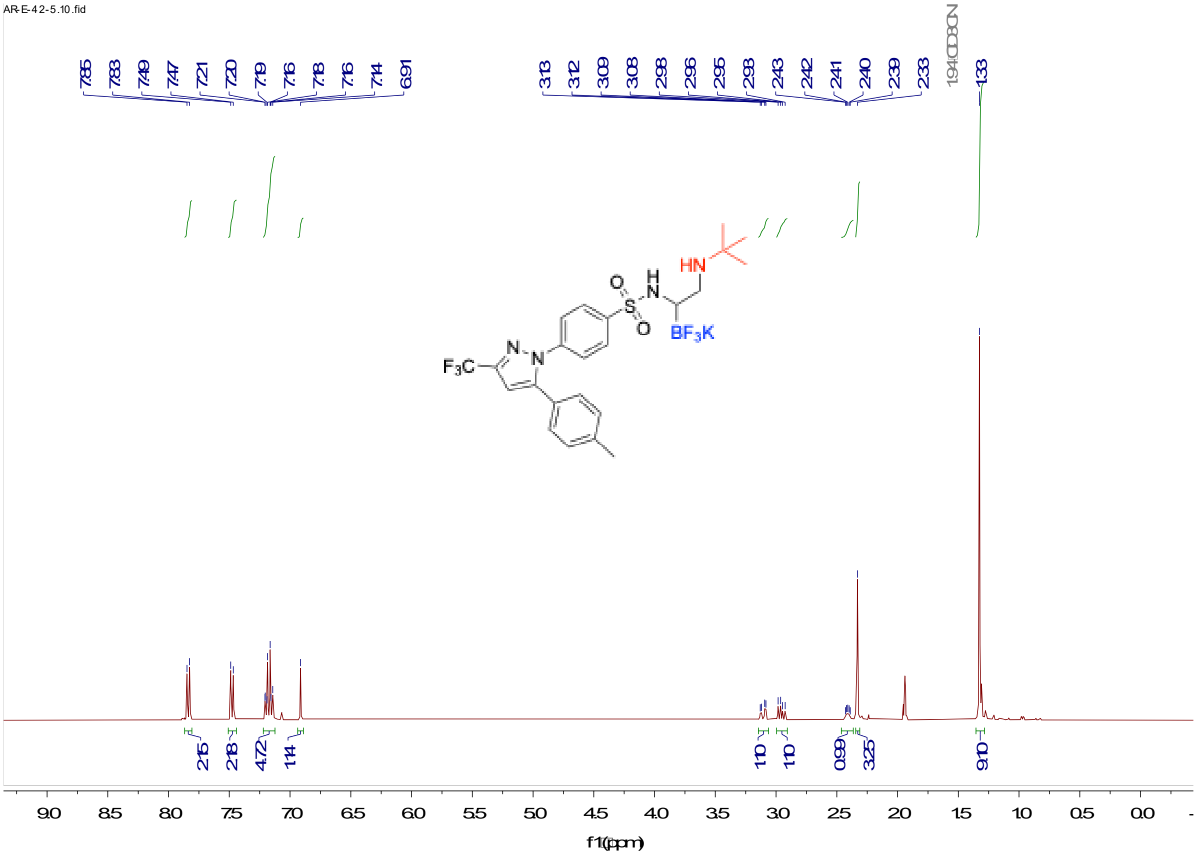
AR-E-42-5.10.fid
7.85
7.83
7.49
7.47
7.21
7.20
7.19
7.16
7.18
7.16
7.14
6.91
3.13
3.12
3.09
3.08
2.98
2.96
2.95
2.93
2.43
2.42
2.41
2.40
2.39
2.33
1.94 CD3CN
1.33
HN
H
N
O
S
O
BF3K
N
N
F3C
2.15
2.18
4.72
1.14
1.10
1.10
0.99
3.25
9.10
f1 (ppm)

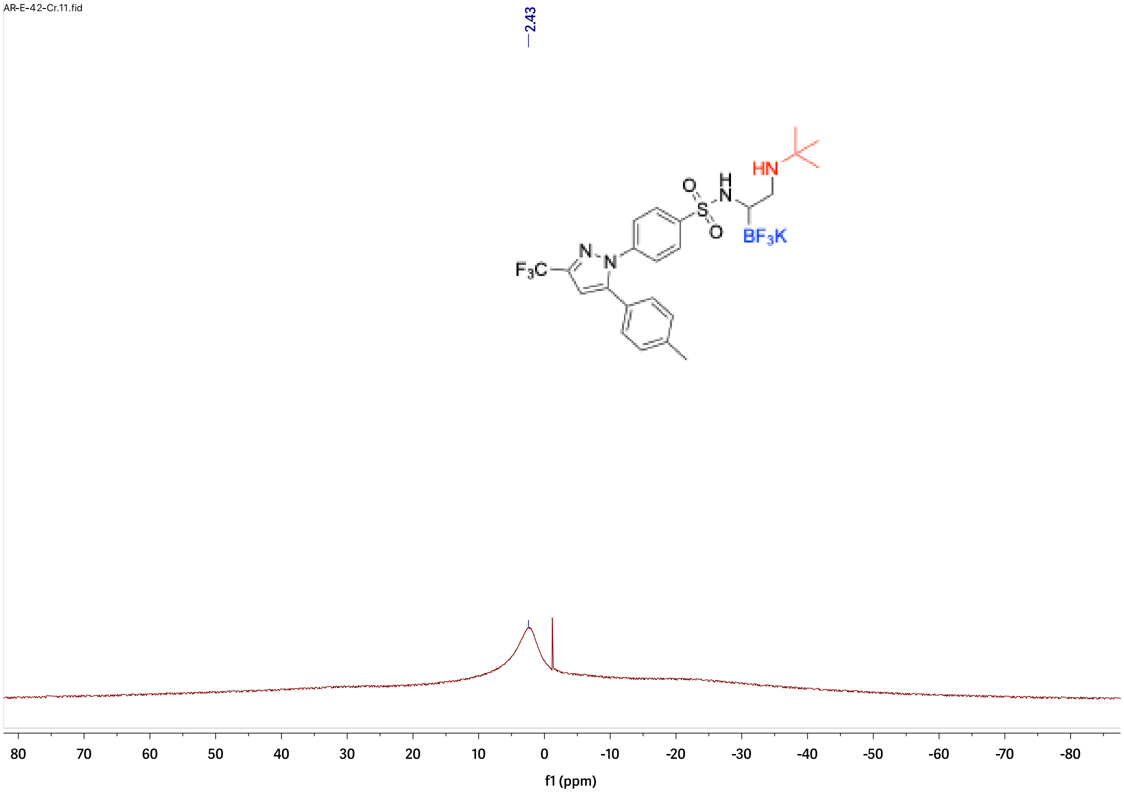
AR-E-42-Cr.11.fid
2.43
HN
H
N
O
S
O
BF3K
N
N
F3C
f1 (ppm)

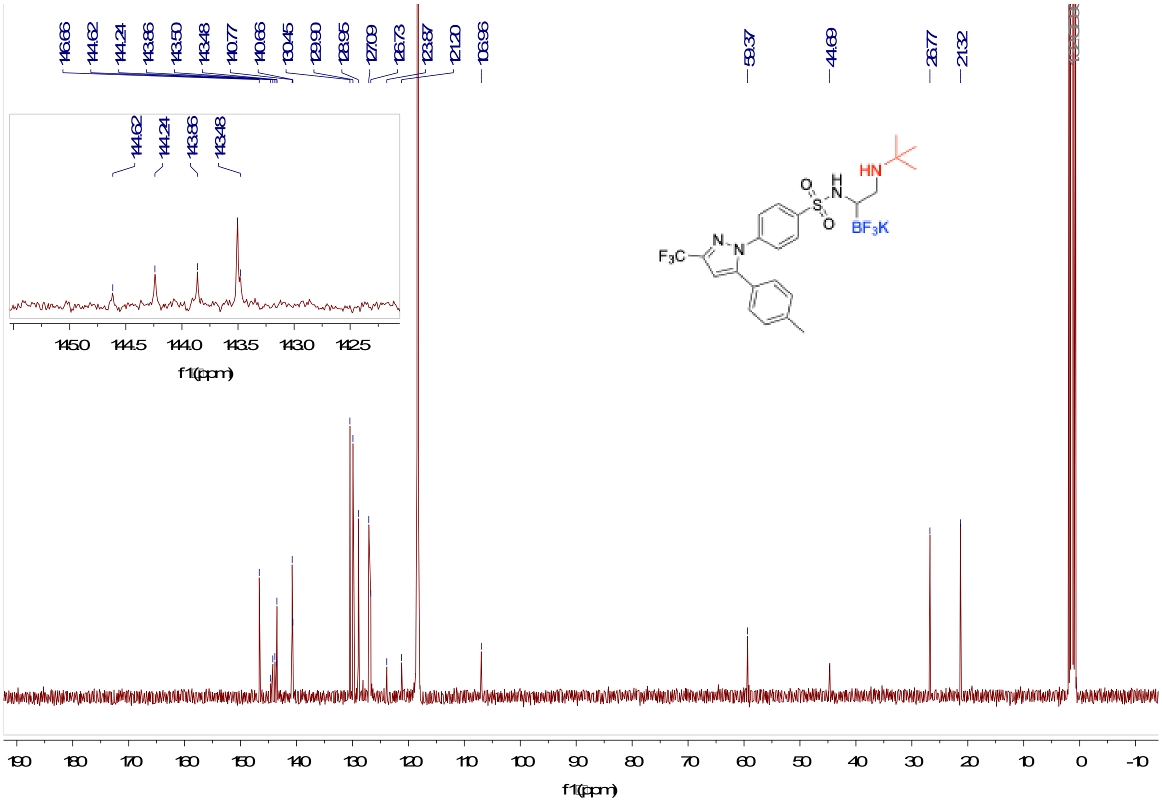
146.66
144.62
144.24
143.86
143.50
143.48
140.77
140.66
130.45
129.90
128.95
127.09
126.73
123.87
121.20
106.96
59.37
44.69
26.77
21.32
F3C
N
N
S
O
O
H
N
HN
BF3K
145.0
144.5
144.0
143.5
143.0
142.5
f1 (ppm)
190
180
170
160
150
140
130
120
110
100
90
80
70
60
50
40
30
20
10
0
-10
f1 (ppm)

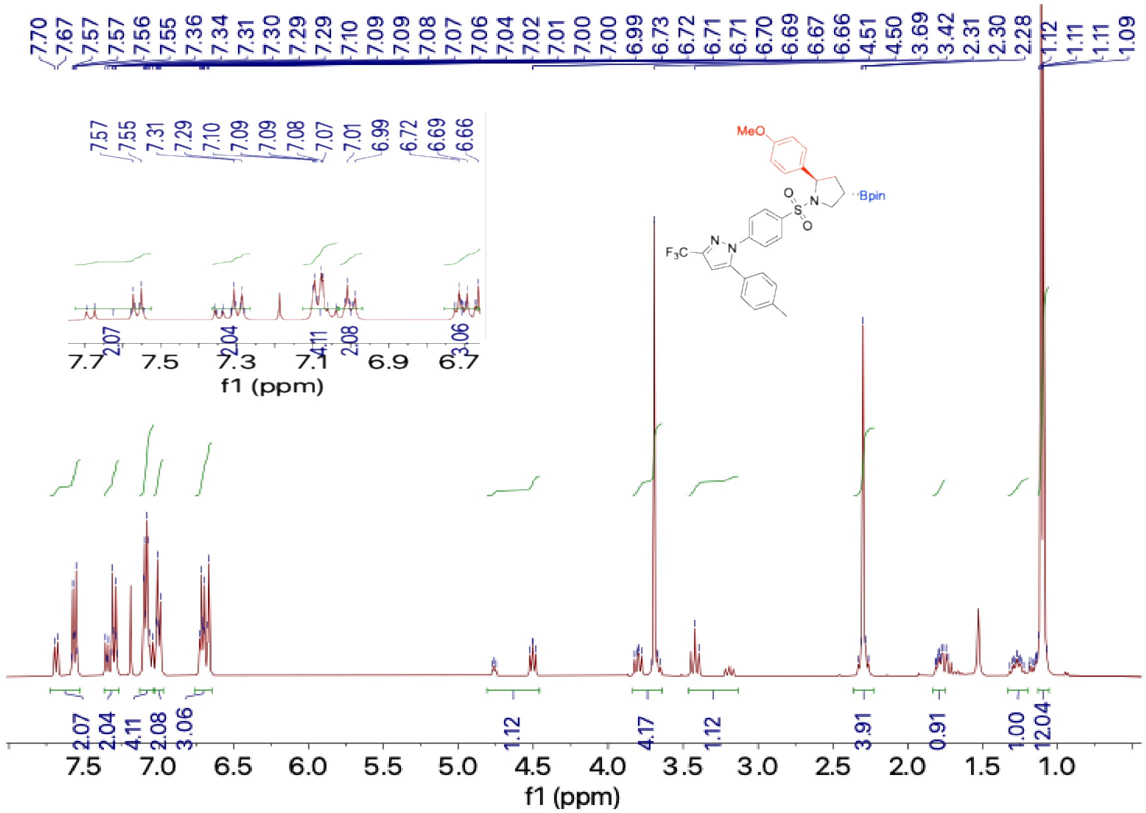

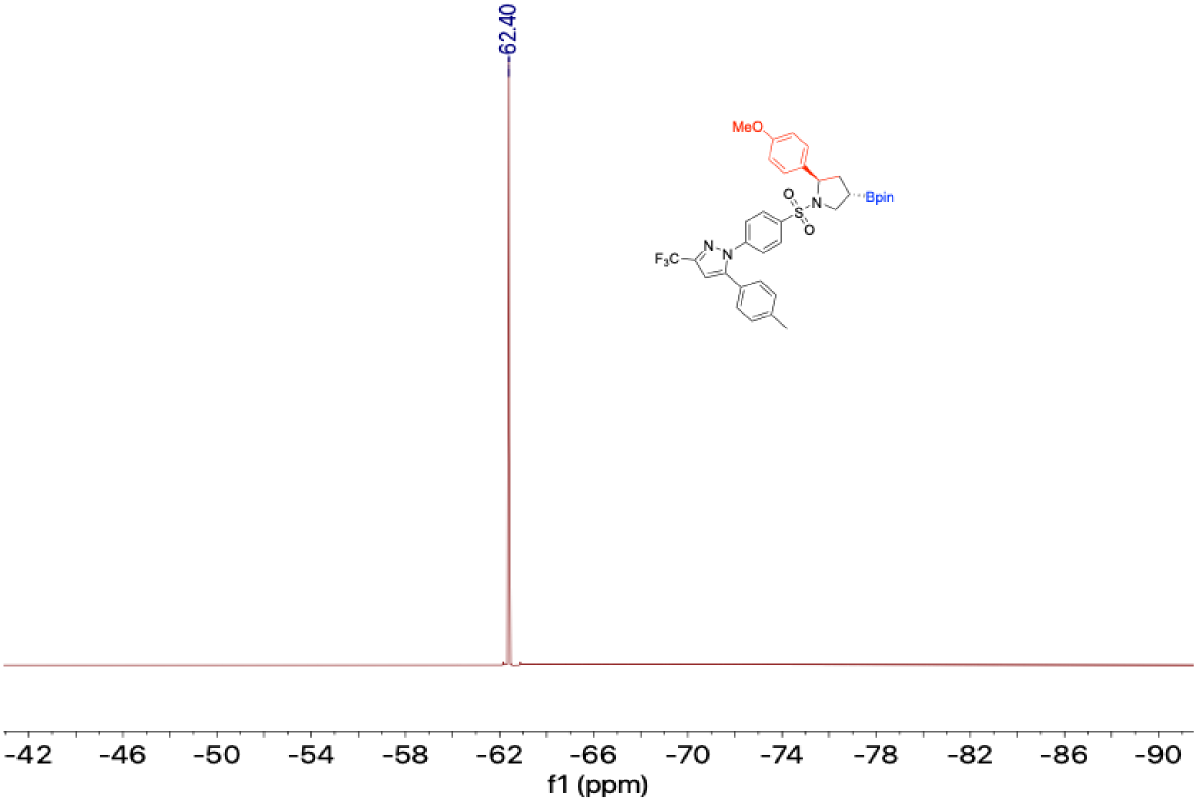

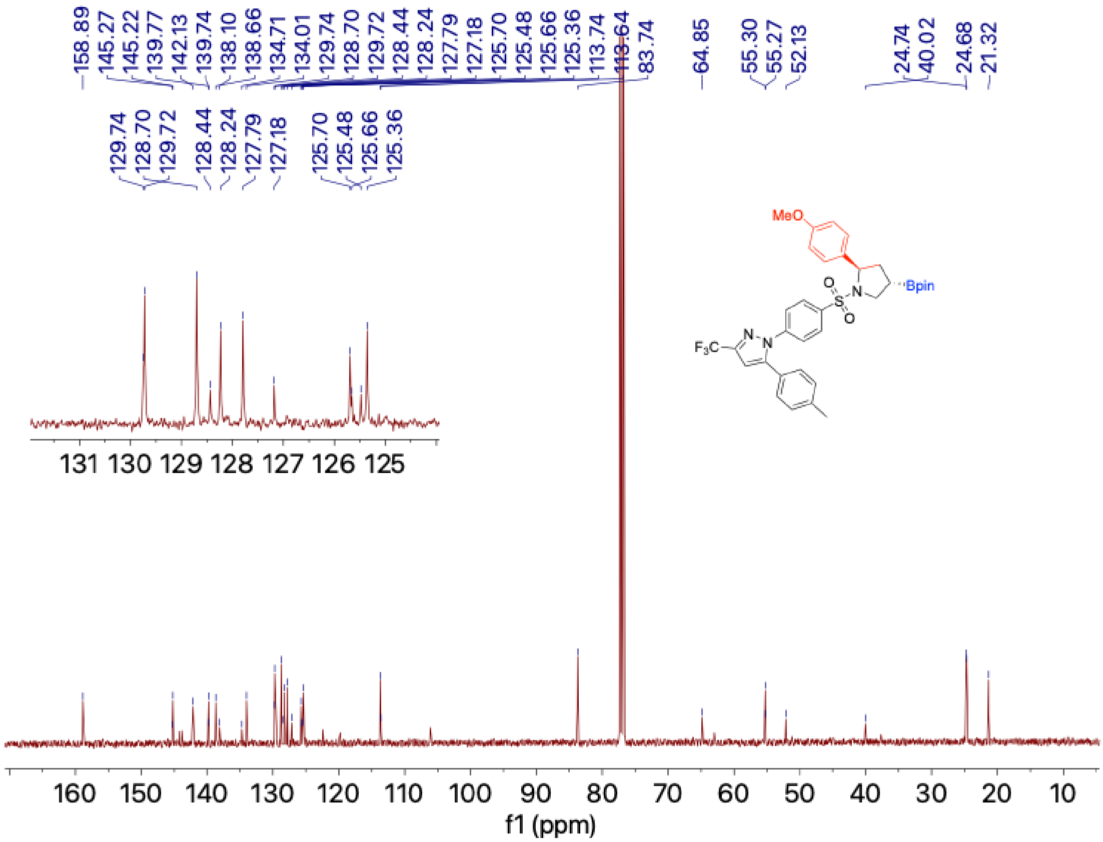

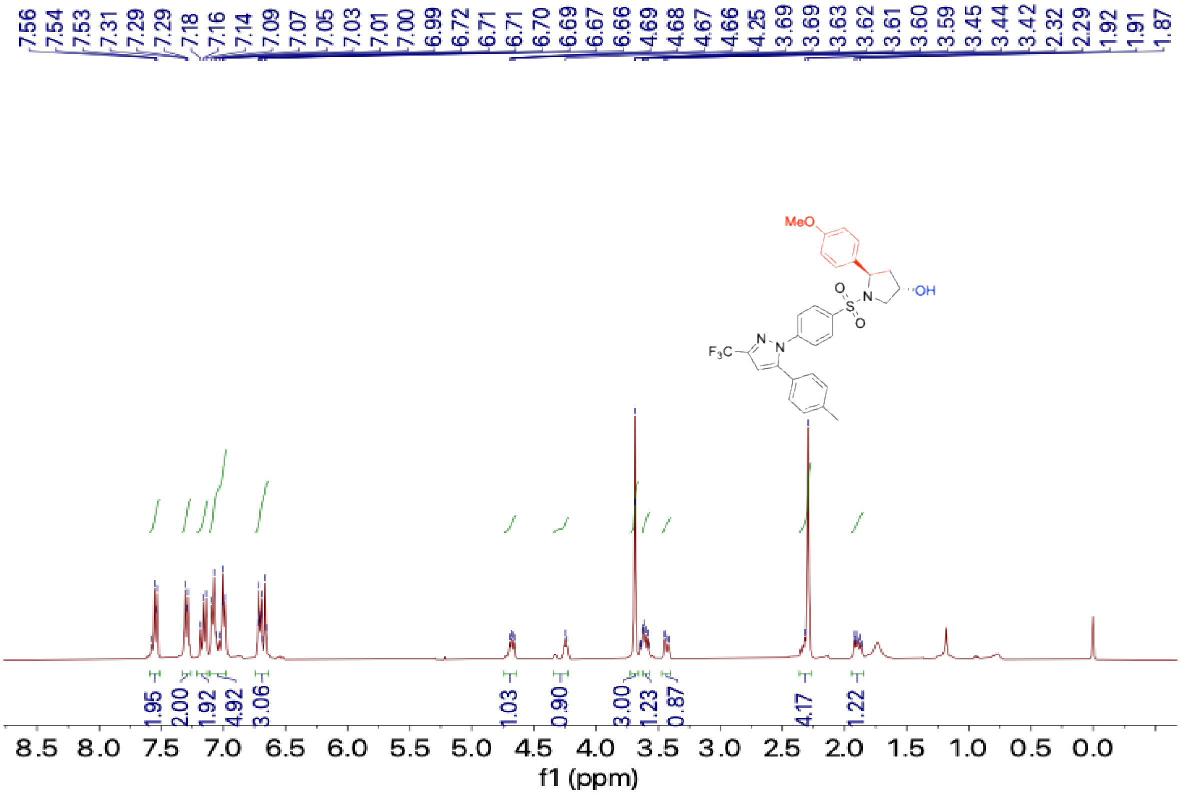

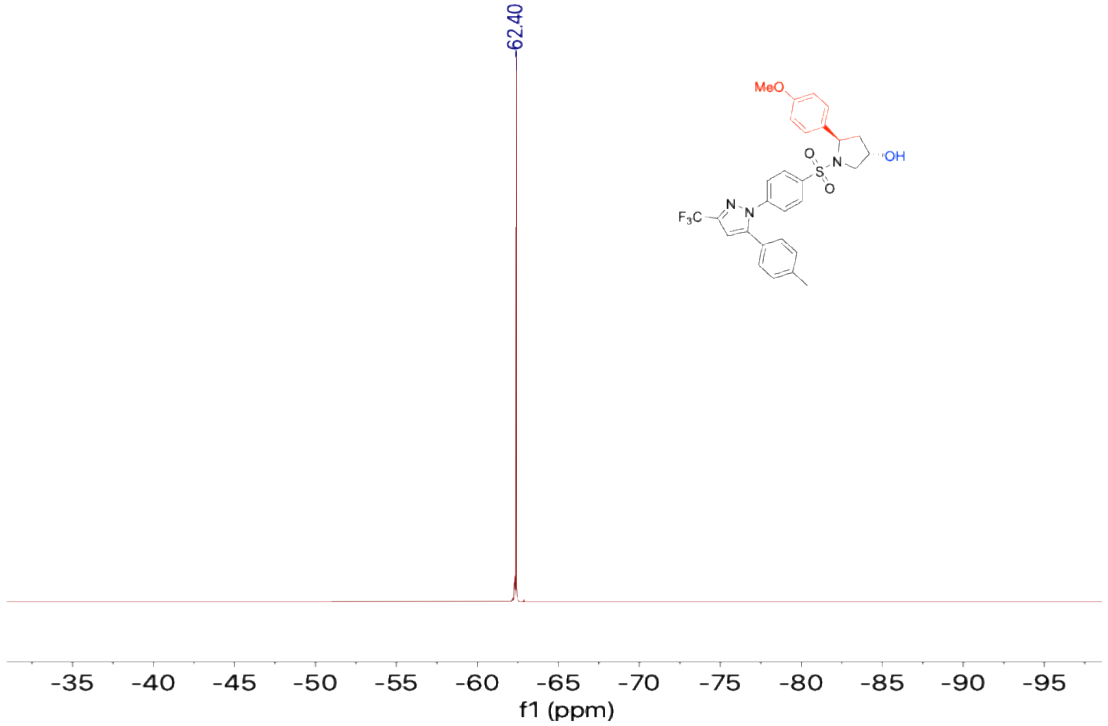
-62.40
MeO
OH
F3C
-35 -40 -45 -50 -55 -60 -65 -70 -75 -80 -85 -90 -95
f1 (ppm)

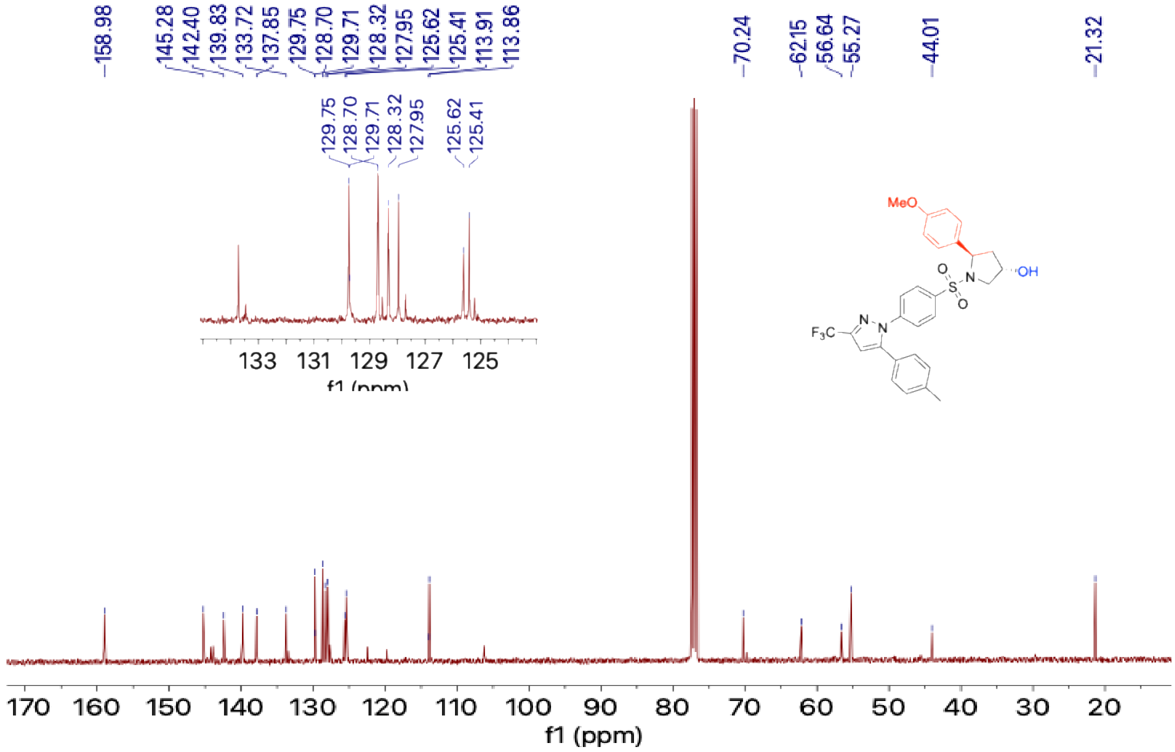
158.98
145.28
142.40
139.83
133.72
137.85
129.75
128.70
129.71
128.32
127.95
125.62
125.41
113.91
113.86
70.24
62.15
56.64
55.27
44.01
21.32
133 131 129 127 125
MeO
OH
F3C
170 160 150 140 130 120 110 100 90 80 70 60 50 40 30 20
f1 (ppm)

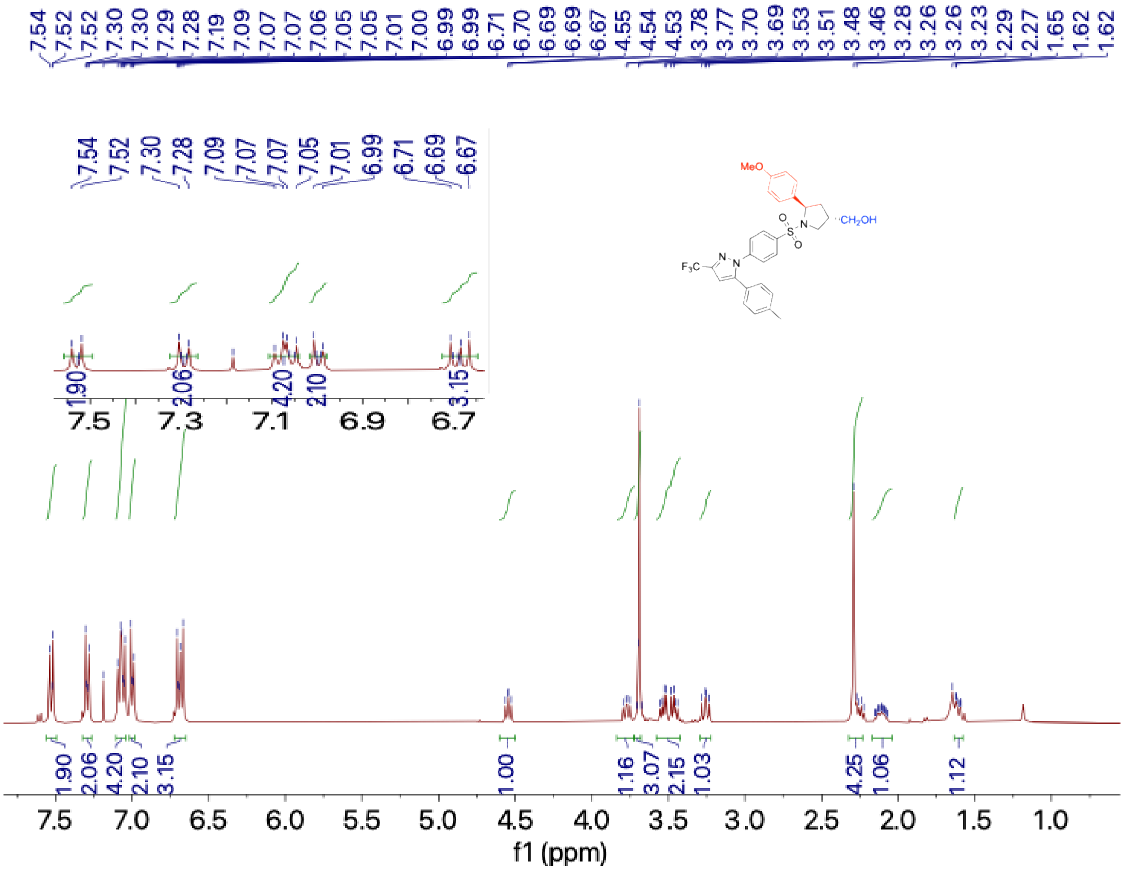

MeO
CH₂OH
F₃C
f1 (ppm)

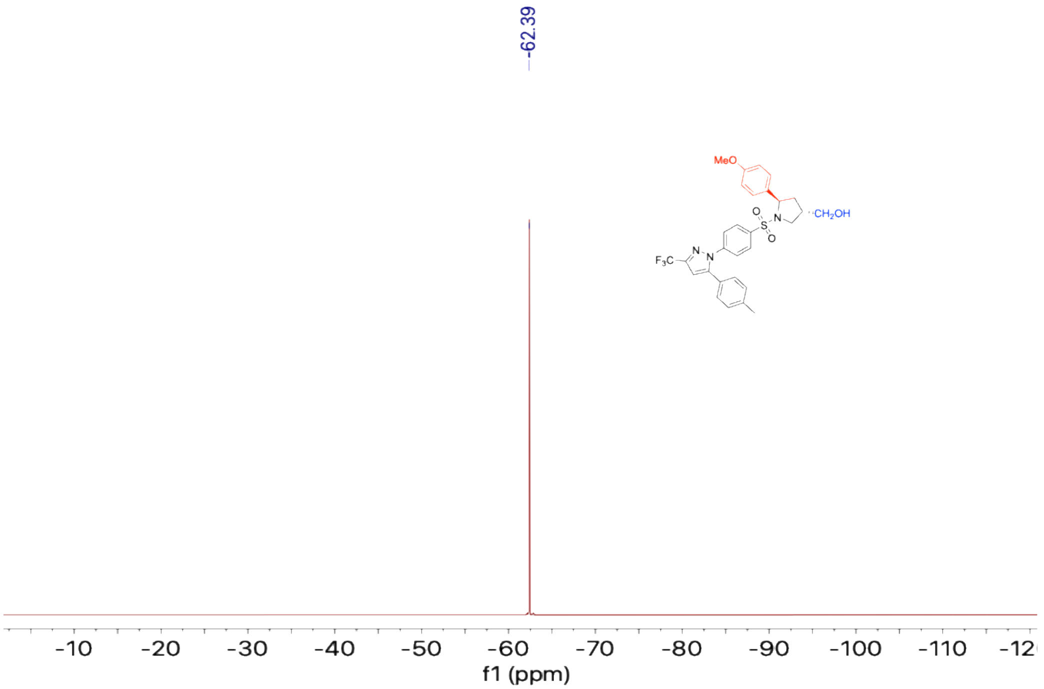

-62.39
MeO
CH₂OH
F₃C
f1 (ppm)

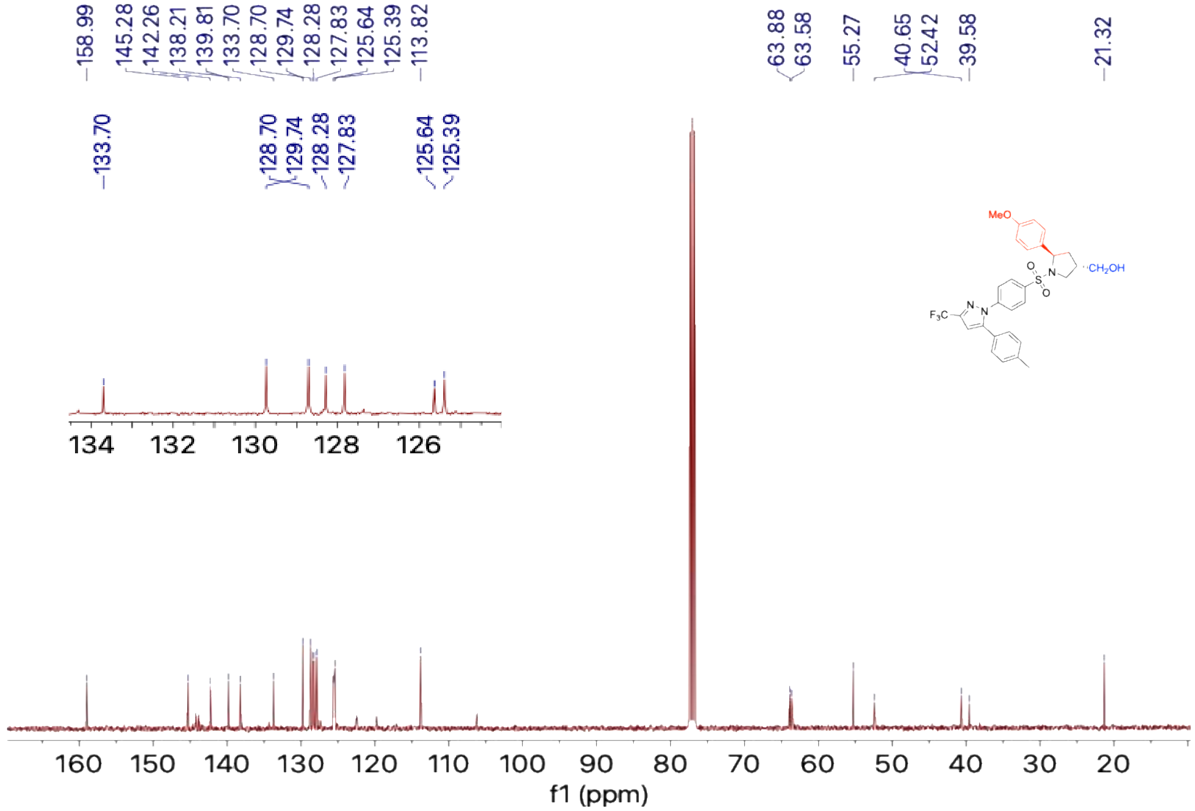

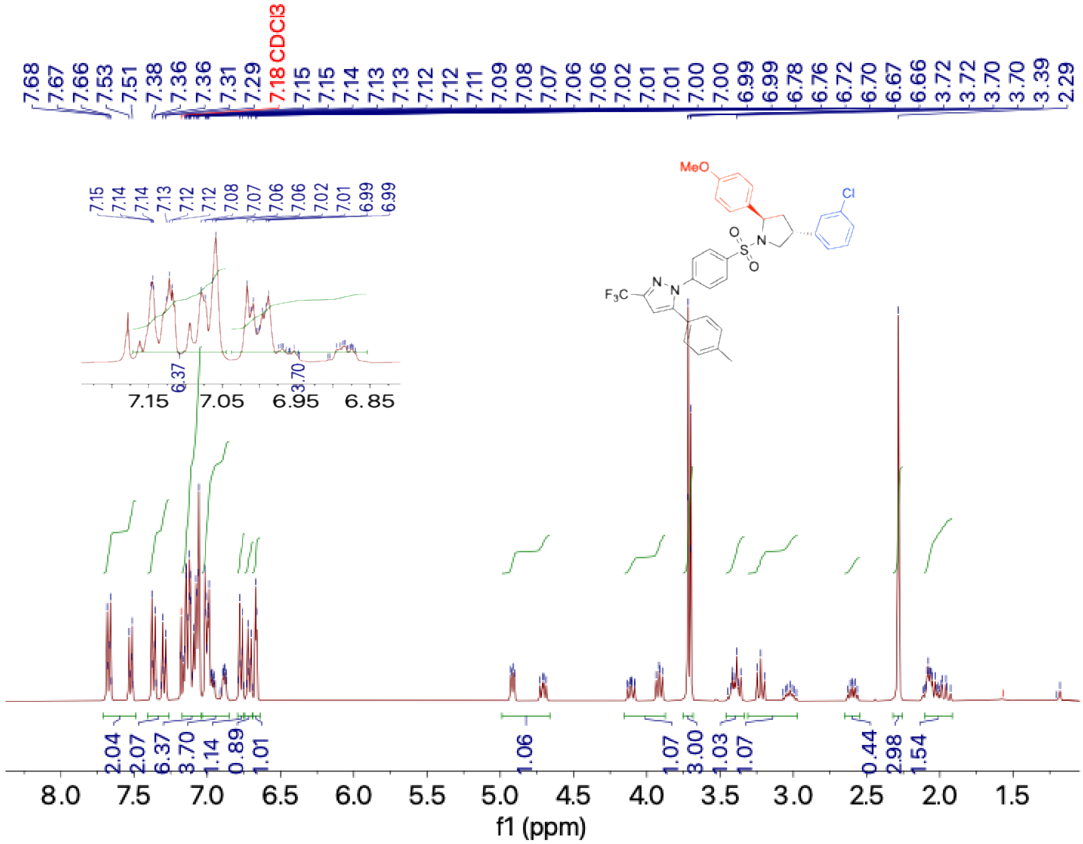

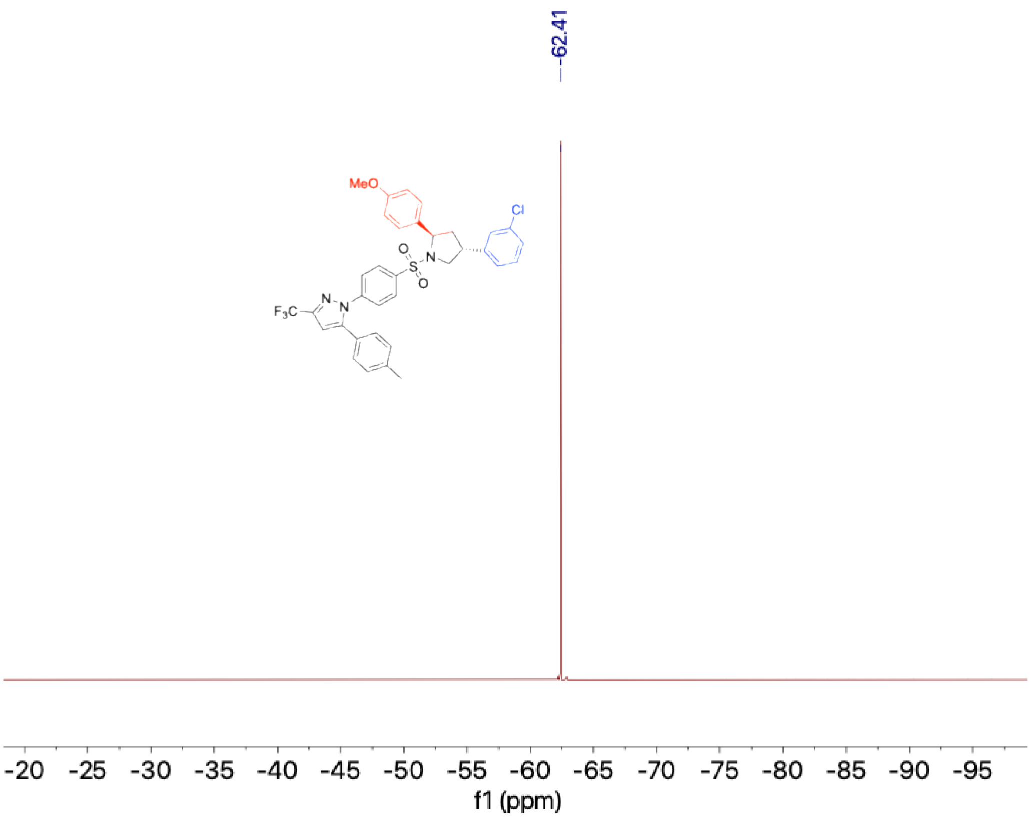
-62.41
MeO
Cl
F3C
N
N
N
O
S
O
-20 -25 -30 -35 -40 -45 -50 -55 -60 -65 -70 -75 -80 -85 -90 -95
f1 (ppm)

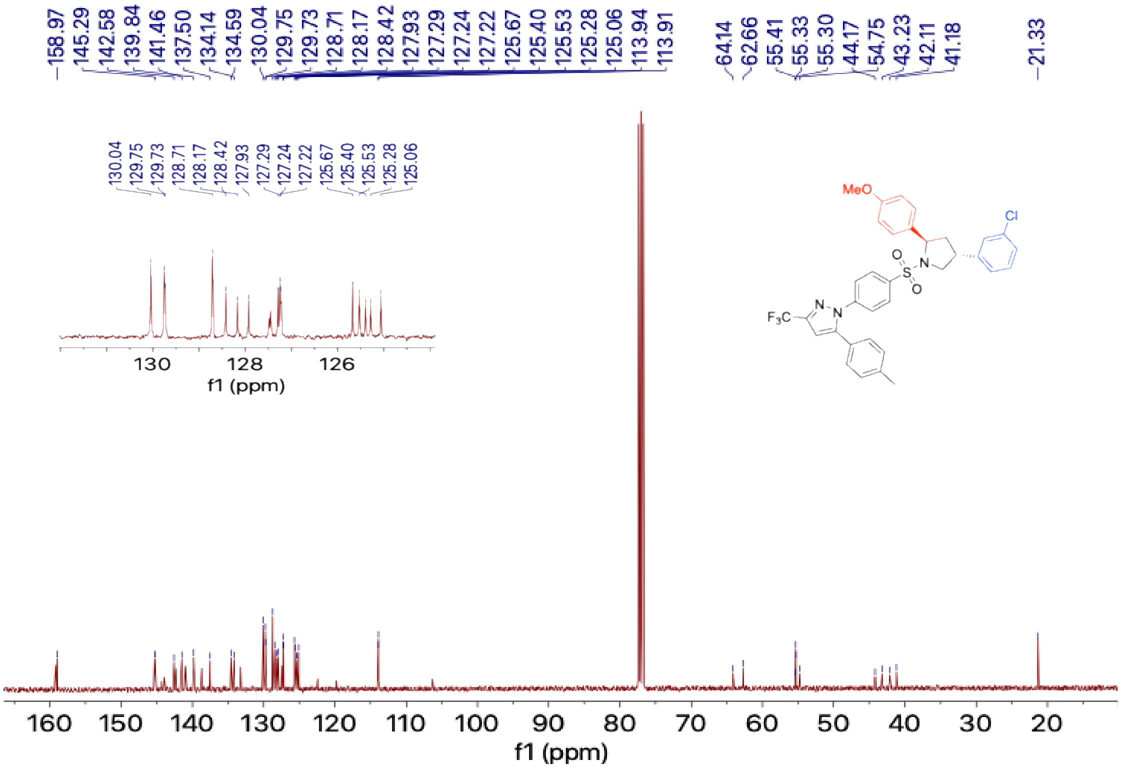
158.97
145.29
142.58
139.84
141.46
137.50
134.14
134.59
130.04
129.75
129.73
128.71
128.17
128.42
127.93
127.29
127.24
127.22
125.67
125.40
125.53
125.28
125.06
113.94
113.91
64.14
62.66
55.41
55.33
55.30
44.17
54.75
43.23
42.11
41.18
21.33
130 128 126
f1 (ppm)
MeO
Cl
F3C
160 150 140 130 120 110 100 90 80 70 60 50 40 30 20
f1 (ppm)

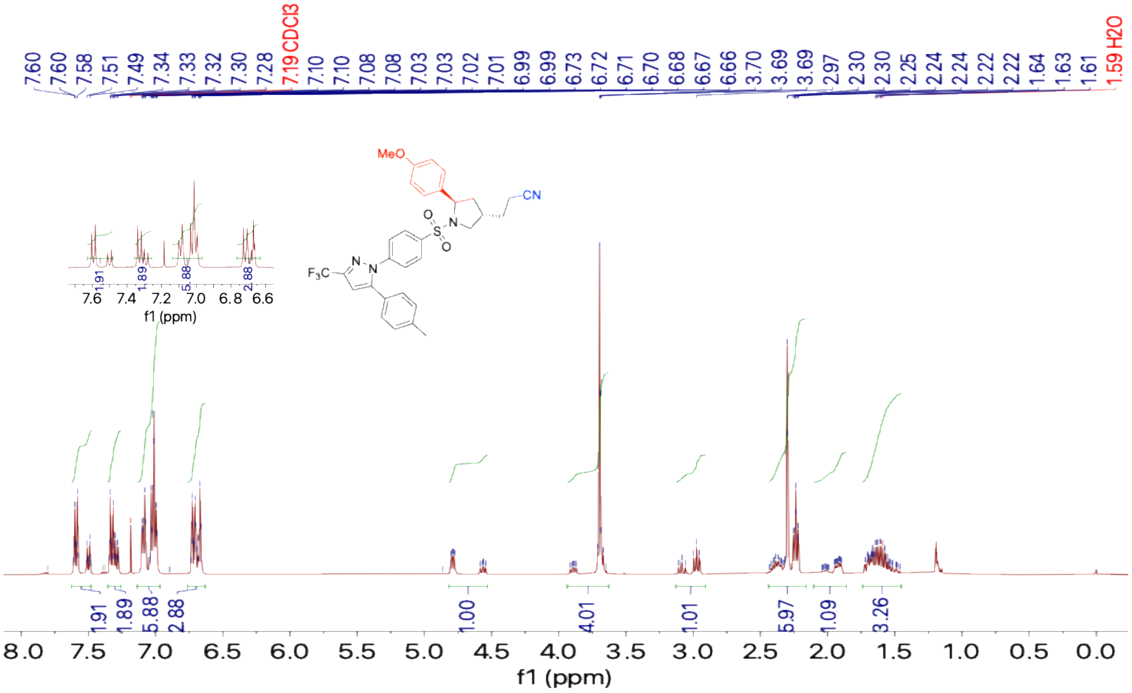
7.60
7.60
7.58
7.51
7.49
7.34
7.33
7.32
7.30
7.28
7.19 CDCl3
7.10
7.10
7.08
7.08
7.03
7.03
7.02
7.01
6.99
6.99
6.73
6.72
6.71
6.70
6.68
6.67
6.66
3.70
3.69
3.69
2.97
2.30
2.30
2.25
2.24
2.24
2.22
2.22
1.64
1.63
1.61
1.59 H2O
MeO
CN
N
O
S
O
F3C
N
N
1.91
1.89
5.88
2.88
1.00
4.01
1.01
5.97
1.09
3.26
f1 (ppm)

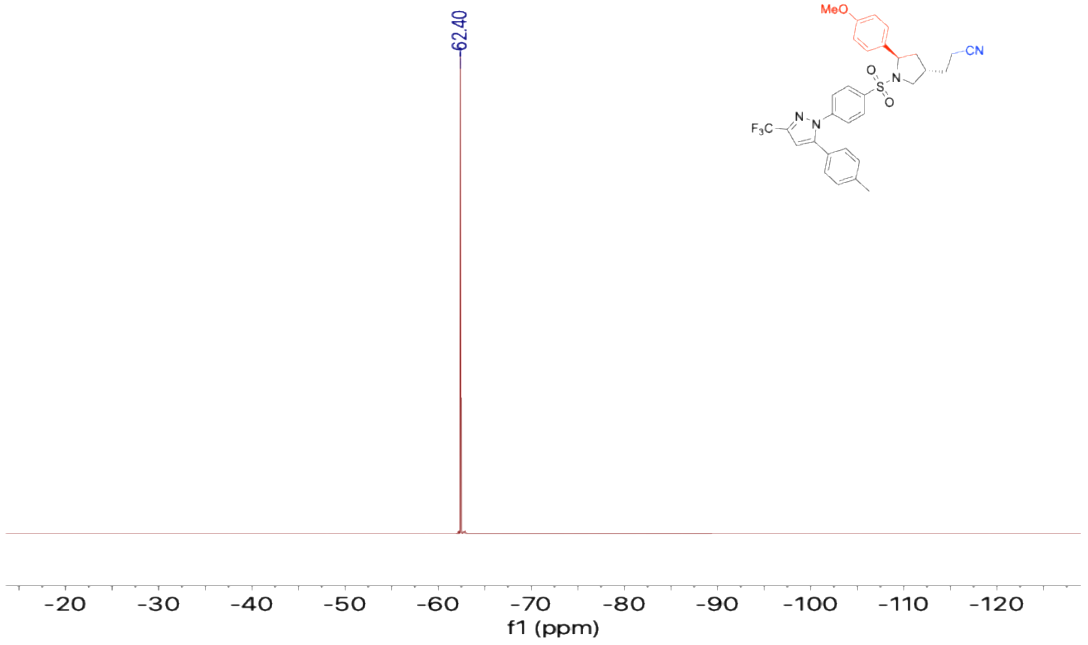
-62.40
MeO
CN
N
O
S
O
F3C
N
N
f1 (ppm)

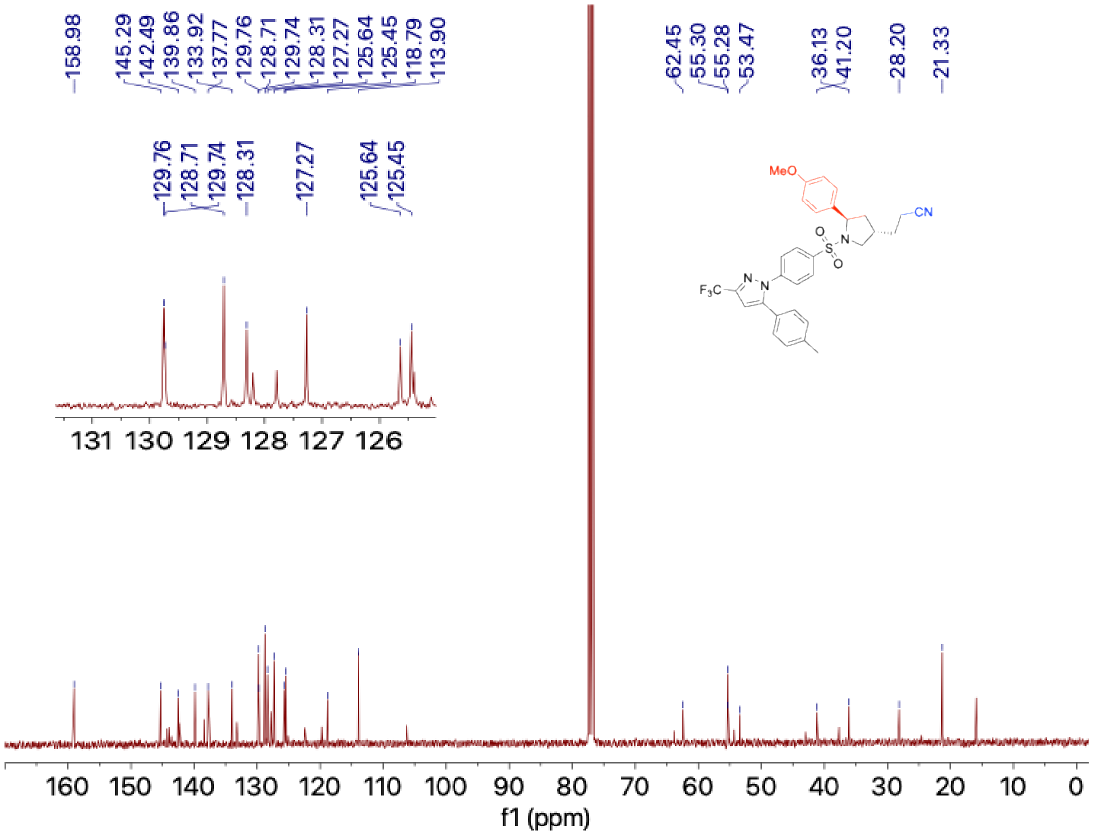

158.98
145.29
142.49
139.86
133.92
137.77
129.76
128.71
129.74
128.31
127.27
125.64
125.45
118.79
113.90
62.45
55.30
55.28
53.47
36.13
41.20
28.20
21.33
129.76
128.71
129.74
128.31
127.27
125.64
125.45
131 130 129 128 127 126
MeO
CN
N
O
S
O
N
N
F3C
160 150 140 130 120 110 100 90 80 70 60 50 40 30 20 10 0
f1 (ppm)

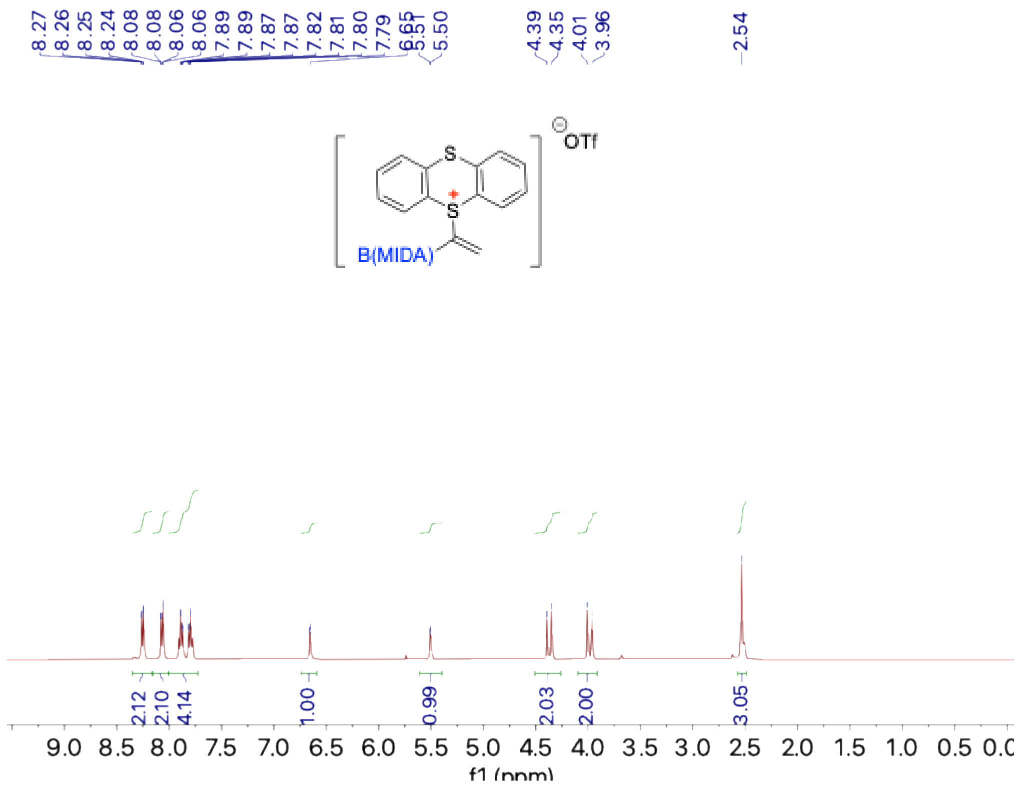
8.27
8.26
8.25
8.24
8.08
8.08
8.06
8.06
7.89
7.89
7.87
7.87
7.82
7.81
7.80
7.79
6.65
5.51
5.50
4.39
4.35
4.01
3.96
2.54
S
S
OTf
B(MIDA)
2.12
2.10
4.14
1.00
0.99
2.03
2.00
3.05
9.0 8.5 8.0 7.5 7.0 6.5 6.0 5.5 5.0 4.5 4.0 3.5 3.0 2.5 2.0 1.5 1.0 0.5 0.0
f1 (ppm)

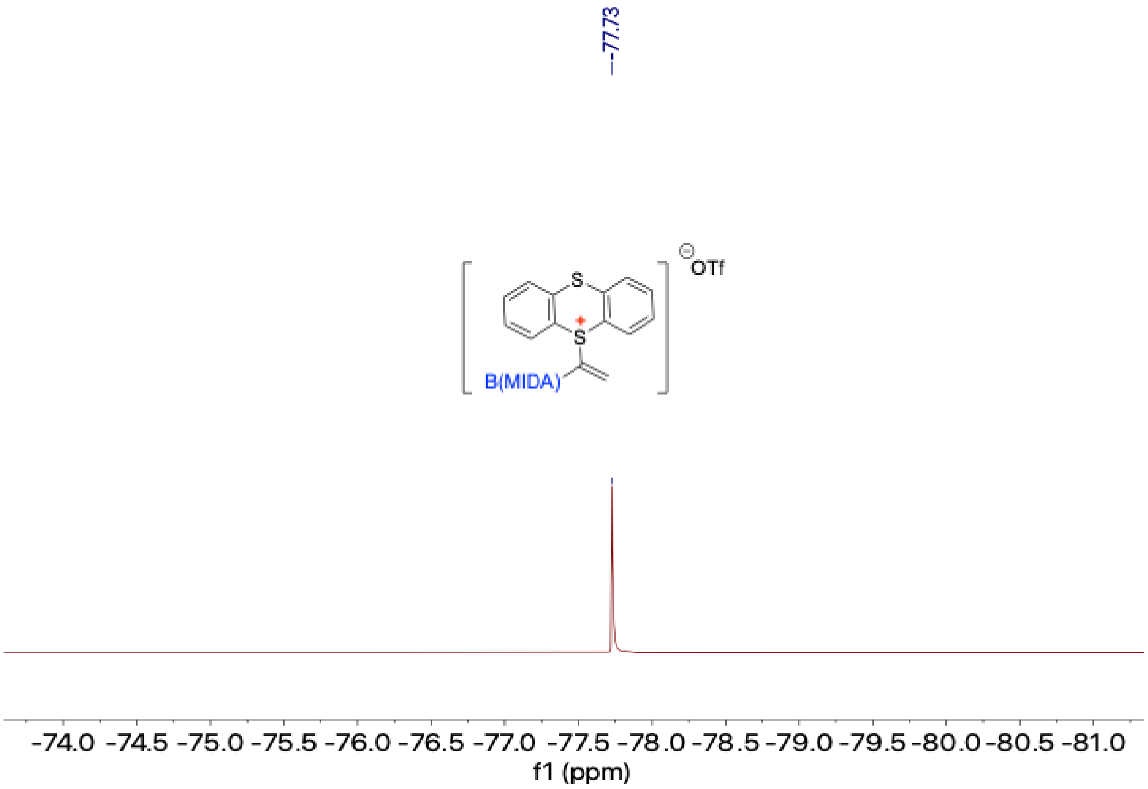
-77.73
S
S
OTf
B(MIDA)
-74.0 -74.5 -75.0 -75.5 -76.0 -76.5 -77.0 -77.5 -78.0 -78.5 -79.0 -79.5 -80.0 -80.5 -81.0
f1 (ppm)

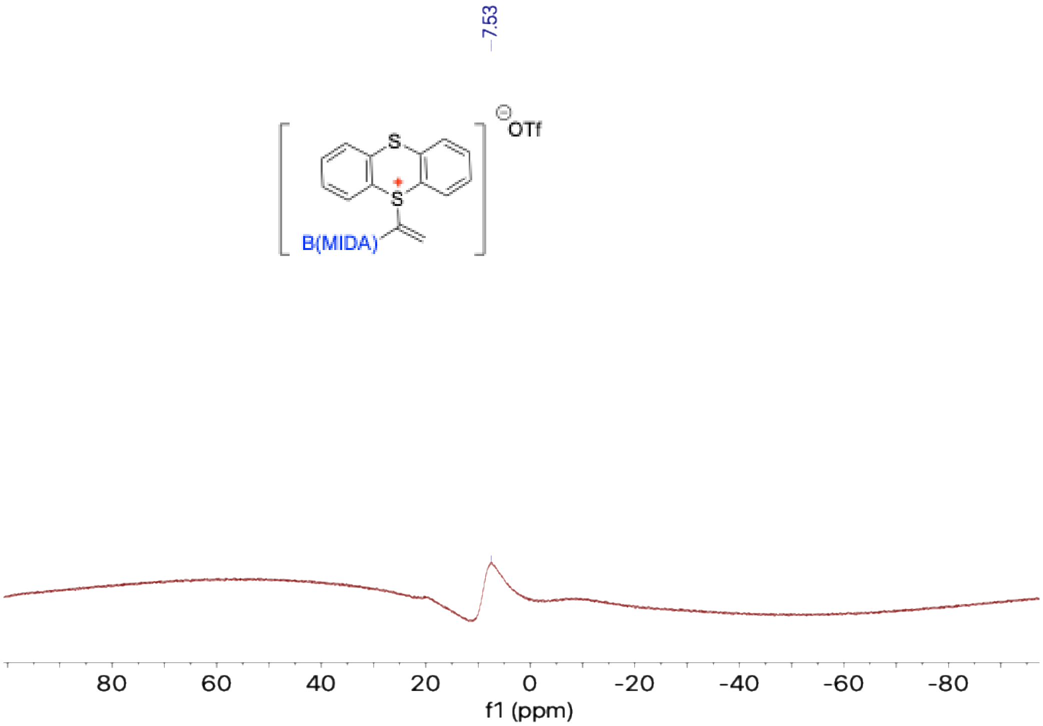

7.53
OTf
S
S
B(MIDA)
80
60
40
20
0
-20
-40
-60
-80
f1 (ppm)

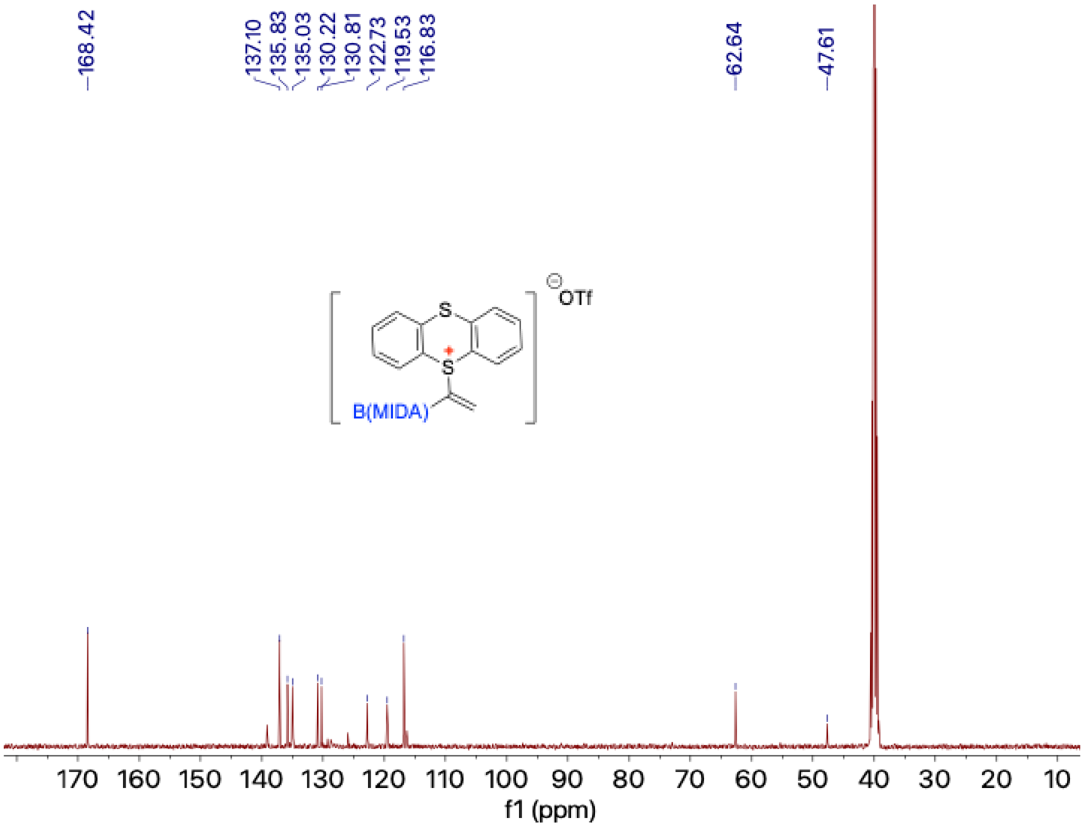

168.42
137.10
135.83
135.03
130.22
130.81
122.73
119.53
116.83
62.64
47.61
OTf
S
S
B(MIDA)
170
160
150
140
130
120
110
100
90
80
70
60
50
40
30
20
10
f1 (ppm)

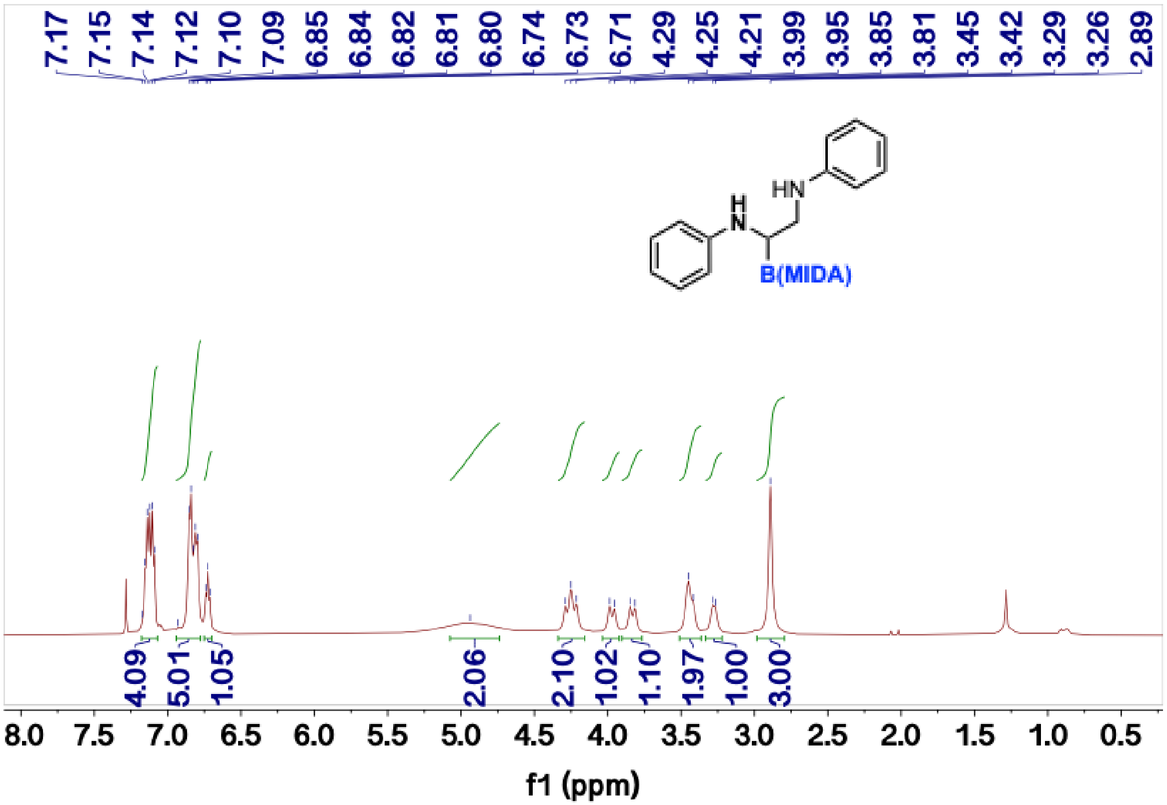
7.17
7.15
7.14
7.12
7.10
7.09
6.85
6.84
6.82
6.81
6.80
6.74
6.73
6.71
4.29
4.25
4.21
3.99
3.95
3.85
3.81
3.45
3.42
3.29
3.26
2.89
HN
H
N
B(MIDA)
4.09
5.01
1.05
2.06
2.10
1.02
1.10
1.97
1.00
3.00
8.0 7.5 7.0 6.5 6.0 5.5 5.0 4.5 4.0 3.5 3.0 2.5 2.0 1.5 1.0 0.5
f1 (ppm)

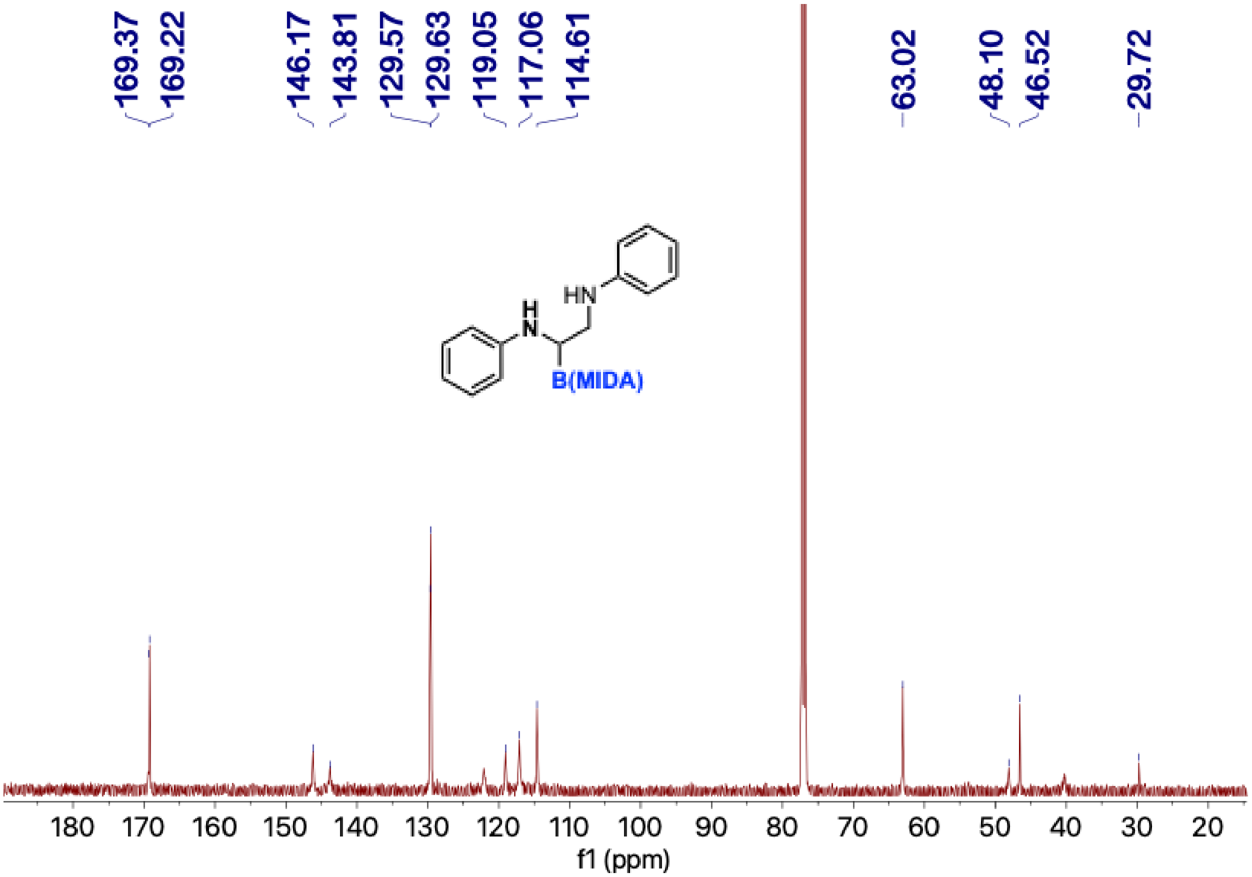
169.37
169.22
146.17
143.81
129.57
129.63
119.05
117.06
114.61
63.02
48.10
46.52
29.72
HN
H
N
B(MIDA)
180 170 160 150 140 130 120 110 100 90 80 70 60 50 40 30 20
f1 (ppm)

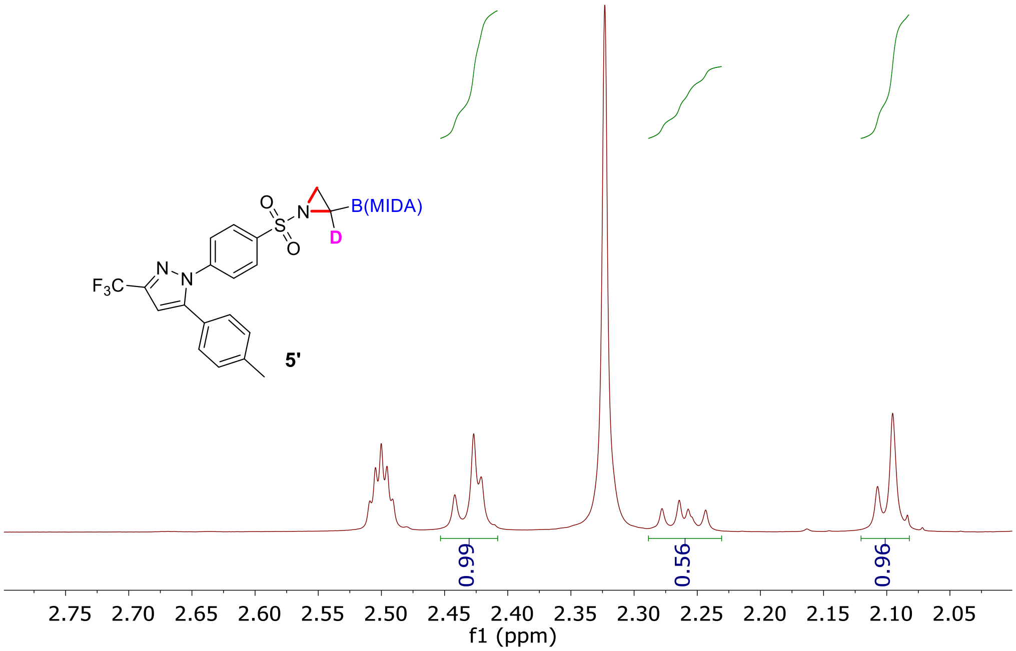
B(MIDA)
D
F3C
5'
0.99
0.56
0.96
2.75 2.70 2.65 2.60 2.55 2.50 2.45 2.40 2.35 2.30 2.25 2.20 2.15 2.10 2.05
f1 (ppm)

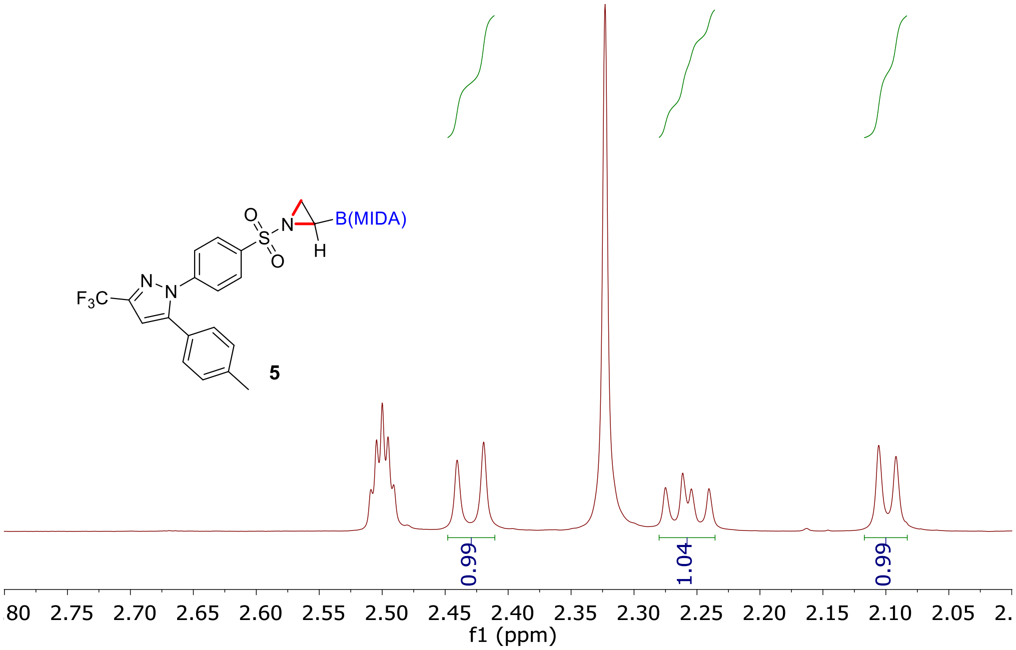
B(MIDA)
H
F3C
5
0.99
1.04
0.99
80 2.75 2.70 2.65 2.60 2.55 2.50 2.45 2.40 2.35 2.30 2.25 2.20 2.15 2.10 2.05 2.
f1 (ppm)
